# Supplementary material for: Structural homology screens reveal poxvirus-encoded proteins impacting inflammasome-mediated defenses
Source: bioRxiv. 2023 Apr 3:2023.02.26.529821. Originally published 2023 Feb 27. Preprint. [Version 2] doi: 10.1101/2023.02.26.529821 (PMC10002665; doi:10.1101/2023.02.26.529821)
Supplement: Supplement 1 [file media-1.pdf]

Vaccinia screen results summary.

Converged = TM-align & FATCAT agreement **Excess Hits** = Large quantity of nonspec

Proteins with <16 secondary structural motifs (as determined by STRIDE) are highlighte

%ID was calculated based on a clustal omega alignment of the indicated uniprot identifi

Some proteins with hits have other hits that are extrememly close to the annotated hit.

Uniprot ID	Orf Name	pLDDT	Length (AA)	ssMotifs	Converged	Excess Hits
P21113	7kDaAtIP	68.74233	65	**9**	N	
P20982	A1	87.2308	150	22	N	
P20642	A10	77.11623	891	122	N	
P20988	A11	56.09341	318	25	N	
P20989	A12	62.00416	192	19	N	
P20990	A13	70.54972	70	**4**	Y	
P20991	A14	69.03298	90	**2**	Y	Y
p0ck28	A14p5	89.63571	53	**2**	Y	Y
P20992	A15	89.0949	94	**15**	N	
P20993	A16	81.98536	378	64	N	
P68592	A17	71.33835	203	22	N	
p20534	A18	86.7	493	74	Y	
P20994	A19	66.21313	77	**15**	N	
P68318	A2	77.66306	224	23	N	
P20995	A20	90.89355	426	58	N	
P20996	A21	85.92326	117	19	N	
P20997	A22	88.70113	176	23	Y	
P20998	A23	91.58571	382	60	N	
P68694	A24	91.11326	1164	213	Y	
P21114	A26	77.11563	322	34	N	
p20535	A27	78.05599	110	**15**	Y	Y
P68632	A28	81.33939	146	25	N	
P21087	A29	95.74789	305	51	N	
p0ck20	A2p5	69.70142	76	**7**	N	
P20643	A3	77.41739	644	97	N	
P68595	A30	78.62309	77	**6**	Y	Y
P21096	A31	80.39635	124	24	N	
P68614	A32	89.23297	270	42	N	
P68616	A33	74.04233	185	20	Y	
P21057	A34	90.60423	168	25	Y	
P21058	A35	88.00402	176	38	N	
P68618	A36	50.56892	221	18	N	
P21060	A37	87.80005	263	50	N	
P21061	A38	87.01837	277	36	Y	
P21062	A39	94.25722	403	85	Y	
P20983	A4	52.38249	281	28	N	
P21063	A40	66.87082	168	27	Y	
P21064	A41	89.74088	219	36	N	
P68695	A42	96.85205	133	25	Y	
P21065	A43	67.30829	194	24	N	
P21097	A44	95.30341	346	50	Y	

P21132	A45	93.24252	125	22	Y	
P21066	A46	71.92699	214	37	N	
P21067	A47	75.06809	244	24	Y	
P68693	A48	96.80824	204	27	Y	
P21068	A49	91.49097	162	18	N	
P68610	A5	76.81659	164	17	N	
p20492	A50	90.70989	552	91	Y	
P21069	A51	83.25104	334	56	N	
P21070	A52	88.23601	190	17	N	
P21071	A53	66.26683	103	20	N	
P68620	A54	58.26713	90	**5**	N	Y
P21073	A55	87.92701	564	106	Y	
P20978	A56	64.19411	315	35	N	
P21074	A57	91.44574	151	20	Y	
P20985	A6	91.34894	372	30	N	
p20635	A7	84.76429	710	134	N	
P20986	A8	79.39829	288	35	N	
P20987	A9	65.02727	99	**4**	Y	
p20510	AorfA	60.24153	119	26	n	
p20511	AorfB	75.7881	86	**1**	N	Y
p20512	AorfC	40.40304	128	**12**	N	
p20513	AorfD	49.14148	88	**10**	Y	
p20514	AorfE	42.43184	166	**13**	N	
p20515	AorfF	65.75827	75	**12**	N	
p20516	AorfG	59.32088	75	**12**	N	
p20517	AorfH	36.84384	128	**13**	N	
p20518	AorfI	55.97135	73	**6**	Y	Y
p20519	AorfJ	48.84006	73	**9**	N	
p20520	AorfK	39.61527	70	**11**	N	
P68624	AorfL	54.92525	88	**7**	Y	
P68626	AorfM	58.78473	76	**3**	Y	Y
p20523	AorfN	70.84175	70	**1**	Y	Y
p20524	AorfO	40.7194	83	**12**	N	
p20525	AorfP	35.78624	89	**14**	N	
p20526	AorfQ	44.01094	77	**7**	N	
p20527	AorfR	61.5128	66	**4**	Y	Y
P68628	AorfS	57.68266	67	**11**	N	
P68630	AorfT	52.02503	81	**8**	N	
p20530	AorfU	63.18684	72	**3**	Y	
p20505	B1	95.97757	300	49	Y	
P68687	B10	89.35938	166	40	Y	
P21007	B11	69.78397	88	**5**	Y	
P21098	B12	94.05945	283	42	Y	
P20841	B13	87.6357	116	20	Y	
P20842	B14	85.83112	222	34	Y	
P21089	B15	93.55426	149	16	Y	
P21116	B16	81.66437	30	**1**	Y	Y
P21075	B17	45.73089	340	61	N	

P21076	B18	87.10394	574	87	Y	
P21077	B19	93.77266	353	70	N	
P20999	B2	91.24447	219	42	N	
P21078	B20	49.01911	127	32	N	
P21099	B21	64.58082	91	**13**	Y	
P21100	B22	80.37308	181	21	N	
P21101	B23	73.59851	386	63	Y	
P21102	B24	81.14964	150	24	Y	
P21103	B25	76.88415	259	33	Y	
P21104	B26	67.3626	103	**10**	Y	
P21105	B27	77.64603	113	**15**	N	
P21106	B28	66.49728	122	24	N	
P21000	B3	75.25853	124	20	Y	
P21001	B4	74.79937	558	67	N	
P21115	B5	85.58255	317	62	N	
P68443	B6	80.86823	173	**11**	Y	
P68445	B7	69.05378	182	32	N	
P21004	B8	92.88861	272	45	N	
P21005	B9	81.06937	77	**9**	N	
P68473	BorfA	46.51293	107	**9**	N	
p20542	BorfB	59.96655	66	**9**	N	
p20543	BorfC	36.44316	95	16	N	
P68475	BorfD	59.85997	71	**5**	Y	
P68477	BorfE	40.35753	91	**13**	N	
p20546	BorfF	50.60222	75	**10**	N	
p20547	BorfG	65.03501	91	**2**	Y	Y
p20548	BorfH	43.73027	70	**13**	N	
p20549	BorfI	56.48816	67	**10**	N	
P21036	C1	82.9674	224	20	N	
P21043	C10	85.73095	331	53	N	
p20494	c11	72.89068	142	24	N	
p20531	C12	86.81473	353	57	Y	
P21044	C13	64.16698	65	**9**	N	
P21045	C14	81.71396	82	**12**	N	
P21037	C2	85.64696	512	93	Y	
P21090	C23	88.57905	244	40	N	
P68639	C3	90.06353	263	56	Y	
P21038	C4	81.96497	316	51	N	
P21039	C5	89.17651	204	20	Y	
P21040	C6	92.6986	151	16	N	
P68599	C7	93.20519	150	29	N	
P21041	C8	83.61364	184	28	N	
P21042	C9	86.34565	634	85	N	
P21119	CorfA	50.19871	69	**12**	N	
P21120	CorfB	60.48731	72	**6**	Y	Y
P21121	CorfC	71.63799	82	**6**	Y	Y
P21122	CorfD	66.64765	70	**3**	Y	Y
P21123	CorfE	84.93081	65	**2**	Y	Y

P20979	D1	90.48484	844	140	N	
P21012	D10	94.27866	248	34	N	
P20637	D11	91.01643	631	100	Y	
P20980	D12	96.66837	287	34	Y	
P68441	D13	95.34617	551	105	N	
P21008	D2	84.00116	146	21	N	
P21009	D3	87.61361	237	38	N	
p20536	D4	96.6796	218	45	Y	
P21010	D5	89.58724	785	147	N	
p20634	D6	86.64423	637	112	Y	
P21034	D7	91.4259	161	34	Y	
p20508	D8	89.6699	304	45	Y	
P21011	D9	91.26687	213	32	N	
p20550	DorfA	59.06901	84	**4**	Y	Y
p20551	DorfB	52.66891	80	**15**	N	
p20552	DorfC	41.73074	69	**10**	N	
P68479	DorfD	55.33893	72	**8**	N	
P68481	DorfE	48.15648	80	17	N	
P68483	DorfF	65.46633	69	**12**	Y	
P68485	DorfG	55.62048	90	**5**	Y	Y
P68487	DorfH	69.0556	61	**1**	Y	Y
p20553	DorfI	72.22042	74	**2**	N	
P21079	E1	94.07629	479	70	N	
P21050	E10	95.66748	95	**10**	Y	
P68449	E11	84.83324	129	22	N	
P21080	E2	89.72825	737	95	N	
P21081	E3	78.20192	190	27	N	
P21082	E4	68.36226	259	29	N	
P21046	E5	74.01381	341	34	N	
P21047	E6	84.78101	567	62	N	
P68447	E7	44.55575	166	20	N	
P21049	E8	80.53922	273	42	N	
p20509	E9	91.44555	1006	146	Y	
P21085	EorfA	49.13843	71	**9**	n	
p20554	EorfB	42.55432	106	**9**	Y	
p20555	EorfC	42.52227	70	**11**	N	
p20556	EorfD	53.83275	66	**15**	N	
p20557	EorfE	63.04056	65	**4**	Y	Y
p20558	EorfF	51.66814	115	**11**	Y	
P68450	F1	68.00448	226	21	N	
P21095	F10	89.65752	439	65	N	
P21052	F11	79.95733	354	45	N	
P21053	F12	88.68075	635	95	N	
P20638	F13	91.70991	372	71	Y	
P21019	F14	65.32265	73	**6**	N	
P21020	F15	81.8579	158	37	N	
P21021	F16	84.14321	231	25	N	
P68454	F17	70.68404	101	21	N	

P68634	F2	94.15347	147	26	Y	
P21013	F3	89.12424	480	94	N	
p20493	F4	88.21558	319	27	Y	
P21014	F5	70.75118	321	51	N	
P68601	F6	66.58151	74	**10**	N	
P21016	F7	50.84788	92	**9**	N	
P21017	F8	68.05785	65	**5**	N	
P68452	F9	83.26356	212	21	N	
p20559	ForfA	59.44491	67	**5**	Y	Y
p20560	ForfB	65.59204	78	**7**	N	
p20561	ForfC	59.57557	96	**7**	Y	Y
p20562	ForfD	67.73666	85	**5**	Y	
p20563	ForfE	58.68662	71	**5**	Y	Y
p20564	ForfF	66.70897	74	**4**	Y	Y
P21022	G1	87.26159	591	85	Y	
P68457	G2	93.47739	220	30	N	
P68459	G3	84.01472	111	**14**	N	
P68461	G4	91.5997	124	22	N	
P21026	G5	93.24123	434	65	N	
P68315	G5p5	77.45979	63	**10**	N	
P21027	G6	90.72357	165	36	N	
P21028	G7	64.33256	371	33	N	
P68612	G8	89.59898	260	50	Y	
P21030	G9	86.37393	340	51	N	
p20565	GorfA	32.24285	132	**13**	N	
p20566	GorfB	72.70645	72	**1**	Y	Y
P20495	H1	97.21163	171	21	Y	
p20496	H2	84.26683	189	28	N	
p20497	H3	86.44234	324	50	N	
P68439	H4	71.77599	795	107	N	
p20538	H5	65.45242	203	**10**	N	
P68697	H6	94.36452	314	41	Y	
p20539	H7	93.55347	146	16	N	
p20567	HorfA	77.6054	71	**2**	Y	Y
p20498	I1	83.96295	312	50	N	
P68604	I2	67.87248	73	**6**	Y	
p20499	I3	81.56212	269	45	N	
P20503	I4	93.1913	771	112	Y	
p20500	I5	83.24622	79	**8**	Y	Y
P68463	I6	85.76244	382	60	N	
p20501	I7	91.60765	423	55	N	
p20502	I8	91.6925	676	120	Y	
p20568	IorfA	57.44336	77	**8**	Y	Y
P21032	J1	73.72111	153	**11**	N	
P68564	J2	92.61234	177	38	Y	
P21033	J3	89.63018	333	37	Y	
P68608	J4	92.21356	185	33	Y	
P21083	J5	80.47185	133	20	N	

p20504	J6	93.89595	1286	208	Y	
p20632	K1	95.64797	284	47	Y	
p20532	K2	89.54373	369	65	Y	
P20639	K3	92.30271	88	18	Y	
p20537	K4	92.23112	424	72	Y	
P21084	K5	73.44188	136	20	Y	
P68465	K6	88.78416	81	**13**	Y	
P68467	K7	88.34052	149	16	Y	
p20569	KorfA	44.21513	81	**12**	N	
p20570	KorfB	61.64135	79	**6**	Y	Y
p20540	L1	78.59391	250	34	N	
P20843	L2	74.66685	87	**2**	Y	
P21031	L3	79.56406	350	43	N	
P20981	L4	79.44113	251	45	N	
P68622	L5	77.47556	128	18	N	
P20640	M1	86.22016	472	59	Y	
P21092	M2	75.51065	220	39	N	
P21054	N1	95.31486	117	**12**	Y	
P20641	N2	75.03608	175	**13**	N	
P21093	O1	82.19695	666	77	N	
P68690	O2	97.4889	108	17	Y	
p0ck22	O3	80.35554	35	**1**	Y	Y
P68489	U7kDa	73.8132	62	**3**	Y	Y
P68491	U8p7kDa	84.77117	72	**2**	Y	Y

:ific (such as groups of unrelated proteins, not a large protein family) observed. This commonly overlaps with va(
d in yellow and excluded as hits. Some select proteins in this category were still further annotated and may be o
ers
. While these are often orthologs of the same gene/protein, they may be of interest. See supplemental figures.

Hit	Hit ID	%ID (Top Hit)
Helicases (top: HELLS)	Q9NRZ9	20.55
Mitochondrial resolvase tefm	Q0P4D6	21.34
Polymerases (Polr2b)	Q8CFI7	24.44
Klrh1	Q3UU75	23.87
Klrb1c	P27814	20.75
CD47	Q61735	27.08
Semaphorin Sema7a	Q9QUR8	27.23
Klra1	P20937	24.2
Profilin (Pfn2)	A0A0R4IPL1	27.27
Steroid dehydrogenases (Top: Hsd3b2)	P26149	32.46

sod1	O73872	18.55	
GSDMD	P57764	20.09	
DTYMK	P23919	18.01	
Ligases (Lig3)	P97386	55.43	
TNFRSF1B	P20333	24	
Kelch proteins (diablo is top)	Q9VUU5	23.49	
Guk1	Q64520	46.36	
Serinc1/drosophila ND-15			
PSME1	Q06323	19.72	
K7DYB1			
Kinases (top: VRK1)	Q99986	40.6	
Kelch proteins (top: Danio kelch-like protein 12)	A0A2R8Q6R0	28.57	
**ambiguous**			
Kinases (top: VRK1)	Q99986	33.33	
Serpins (top: Fly Spn42Dc)	A1Z6R4	33.04	*B13/B14 are fragment
Serpins (top: Serpina3j)	D3Z451	29.03	
Bcl2l2	P70345	13.86	

Ankyrin repeat and SOCS box-containing 15a	A0A2R8PZ70	20.53
Below threshhold: IL1r2		
Ankrd37		
ASB4	Q9Y574	20
Ankyrin proteins (top: ANKRD22)	Q5VYY1	22.76
Ankyrin proteins (top: Danio Ankyrin repeat and SOCS box pr	Q7T386	23.67
ambiguous - ankyrin or protein phosphatase 1?		
Slfn1	Q3UU73	25.21
Buffy	Q8T8Y5	20.96
F3/IFNGR1 (below threshold)		
Tmem91, KANTR		
Strong pyrin domain hits w/ FATCAT alone, some BCL2 domains with TM-align. See main text.		
Serpins (top: Serpinb1c)	Q5SV42	34.2
Keap1	Q14145	22.2
C4BPA	P04003	37.45
Kelch proteins(top: KLHL8)	Q9P2G9	21.03
below thresshold: bcl2l15		

just under thresshold: Nudt2			
Chd3, HELLS, smarcad1 - helicases	O16102	19.44	
Cmtr1, 2 (methyltransferase)	Q9W4N2	19.79	
UNG1 (danio)	A2BIQ2	19.81	
smarcad1b (danio)	E7F1C4	21.98	**look back
POL2RG	P62487	17.39	
Ca5b	Q9QZA0	33.33	
just under thresshold: NudT15, 16, etc			
	A1A750, weak		
lots, ambiguous			
lots, ambiguous			
GFER			
PolD1	P54358	24.26	
TMEM18			
CHIC1	Q5VXU3	18.18	
PLD4	Q96BZ4	25.62	

Dut	Q9CQ43	59.86
Rrm2b	Q6PEE3	75.24
Ifitm10?	Q8BR26	17.11
Ide	P22817	17.9
just below threshhold: FEN1/XRN2/XRN1 nucleases		
just below threshhold: polr2l		
just below threshhold: Plaat1, tmem222 - previously shown to have similarity to bacterial peptidase, NLPC/P6		
PCNA	Q9VIT0	20.4
Phosphatases (top: Danio tyrosine phosphatase Q0P4E3)	Q0P4E3	27.03
top1	E7FE49	19.59
mt-co3		
Rrm1	P07742	76.01
ambiguous, maybe odorant receptors/olfactory receptors?		
helicases (top: DHX35)	Q9H5Z1	20.5
TK1	P04184	66.67
cmtr2	Q9UAS6	19.75
POLR2E	P19388	21.88

POLR2A	P24928	24.07
Ankyrin proteins (top: Nfkbid)	Q2TB02	19.03
Serpins (top: SERPINA3	P01011	24.59
polr2g	P62488	20.48
PLD3	O35405	47.73
Mgll	O35678	32.35
Mgll		
BCL2L10	Q9Z0F3	18.8
ambiguous		
ASB2, other ankyrin	Q96Q27	23.94
MCL1		
Glrx	P35754	46.23

ccinia proteins with few structural motifs.
f interest.

s of a Serpin present as a single ORF in vaccinia strain WR

family, Plaat is a phospholipase/acyltransferase within this family

Synteny Analysis

Genome	Accession
Vaccinia virus	NC_006998
Skunkpox virus	NC_031038
NY014 poxvirus	NC_035469.1
Myxoma virus	NC_001132.2
Eptesipox virus	NC_035460.1

Poxvirus BLAST Database

Genome	Accession
Cotia virus	HQ646181.2
Saltwater crocodilepox	MK903858.1
Deerpox virus	AY689437.1
Fowlpox virus	MH706124.1
Eastern grey kangaroopox virus	MF661791.2
Lumpy skin disease virus	NC_003027.1
Molluscum contagiosum virus	MH320554.1
Orf virus	MG716417.1
Pteropox virus	KU980965.1
Sea otter poxvirus	MH427217.1
Sheeppox virus	MN072631.1
Squirrel poxvirus	HE601899.1
Swinepox virus	MZ682626.1
Yaba monkey tumor virus	AY386371.1

Viral gasdermins

Protein	Accession
Vaccinia virus gasdermin (COP)	M35027.1:c153690-152956
Rabbitpox virus gasdermin	AY484669.1:c159516-158782
Horsepox virus gasdermin	KY349117.1:c166272-165538
Buffalopox virus gasdermin	MG599038.1:c157906-157148
Vaccinia virus gasdermin (WR)	AY243312.1:c154675-153917
Cowpox virus gasdermin	KC813500.1:c153312-152578
Orthopoxvirus abatino gasdermin	NC 055231.1:c163765-163031
Ectromelia virus gasdermin	MN912466.1:c156293-155559
Akhmeta virus gasdermin	MH607143.1:c167386-166652
Taterapox virus gasdermin	DQ437594.1:c153307-152573
Variola virus gasdermin (17CE)	KY358055.1:c145385-144651
Variola virus gasdermin (1947)	DQ441432.1:c146026-145292
Variola virus gasdermin (1969)	DQ441437.1:c146904-146170
Alaskapox virus gasdermin	MN240300.1:c166555-165827
Skunkpox virus gasdermin	KU749310.1:c167631-166873
Volepox virus gasdermin	KU749311.1:c169057-168281
Raccoonpox virus gasdermin	KU749309.1:c145369-144644
Camelpox virus gasdermin (CMS, truncated)	AY009089.1:c154402-154034
Camelpox virus gasdermin (truncated)	MK910851.1:c154204-153836
NY014 poxvirus gasdermin	MF001305.1:c159640-158771
Murmansk poxxvirus gasdermin	MF001304.1:c160603-159728
Yoka poxvirus gasdermin	HQ849551.1:c134784-134035
Skunkpox virus gasdermin (short)	KU749310.1:172437-172985
Racconpox virus gasdermin (short)	KU749309.1:150174-150722
Volepox virus gasdermin (short)	KU749311.1:173861-174445
Murmansk poxxvirus gasdermin (short)	MF001304.1:c165763-165224
NY014 poxvirus gasdermin (short)	MF001305.1:c164808-164269
Myxoma virus gasdermin	NP_051846.1
Rabbit fibroma virus gasdermin	NP_052021.1
Eptesipox virus gasdermin	YP_009408097.1

Vertebrate gasdermins

Protein	Accession
Human gasdermin a	NP_835465.2
Chicken gasdermin a	NP_001026532.1
Bos taurus gasdermin a	NP_001071558.1
Macaca mulatta gasdermin a	XP_014975037.1
Pan troglodytes gasdermin a	XP_001171222.1
Desmodus rotundus gasdermin a	XP_024429158.1
Rousettus aegyptiacus gasdermin a	XP_016020091.2
Pteropus alecto gasdermin a	XP_006924750.1
Halichoerus grypus gasdermin a	XP_035935879.1
Octodon degus gasdermin a	XP_004633937.1
Rhinatrema bivittatum gasdermin a	XP_029428328.1
Amblyraja radiata gasdermin a	XP_032891479.1
Mouse gasdermin a3	Q5Y4Y6
Mouse gasdermin a3	Q9EST1

Mouse gasdermin a2	Q32M21
Human gasdermin b	NP_001159430.1
Bos taurus gasdermin b	NP_001231146.1
Pan troglodytes gasdermin b	XP_009430521.1
Sarcophilus harrisii gasdermin b	XP_023358160.1
Pteropus alecto gasdermin b	XP_024897515.1
Rousettus aegyptiacus gasdermin b	XP_036078993.1
Desmodus rotundus gasdermin b	XP_024429114.1
Macaca mulatta gasdermin b	XP_014975035.1
Halichoerus grypus gasdermin b	XP_035935913.1
Human gasdermin c	NP_113603.1
Macaca mulatta gasdermin c	XP_28708741.1
Bos taurus gasdermin c	NP_001039469.1
Sarcophilus harrisii gasdermin c	XP_31803207.1
Rousettus aegyptiacus gasdermin c	XP_036088919.1
Desmodus rotundus gasdermin c	XP_024427836.1
Felis catus gasdermin c	XP_019678683.1
Pan troglodytes gasdermin c	XP_001153860.1
Mus pahari gasdermin c	XP_021073018.1
Human gasdermin d	NP_079012.3
Mouse gasdermin d	NP_081236.1
Bos taurus gasdermin d	NP_001039625.1
Macaca mulatta gasdermin d	XP_015001615.2
Pteropus alecto gasdermin d	XP_006913014.1
Rousettus aegyptiacus gasdermin d	XP_036089037.1
Desmodus rotundus gasdermin d	XP_024428237.1
Pan troglodytes gasdermin d	XP_009454389.2
Felis catus gasdermin d	XP_023104116.1
Human gasdermin e	NP_001120925.1
Mouse gasdermin e	NP_061239.1
Chicken gasdermin e	NP_001006361.1
Bos taurus gasdermin e	NP_001180041.1
Xenopus tropicalis gasdermin e	XP_002933445.2
Macaca mulatta gasdermin e	XP_001096213.2
Pteropus alecto gasdermin e	XP_006912063.1
Sarcophilus harrisii gasdermin e	XP_031796450.1
Chelonia mydas gasdermin e	XP_027689547.1
Rana temporaria gasdermin e	XP_040208817.1
Chrysemys picta bellii gasdermin e	XP_023962559.1
Rhinatrema bivittatum gasdermin e	XP_029444983.1
Octodon degus gasdermin e	XP_004626528.1
Mus pahari gasdermin e	XP_021046698.1
Pygosceli adeliae gasdermin e	XP_009324004.1
Desmodus rotundus gasdermin e	XP_024418892.1
Rousettus aegyptiacus gasdermin e	XP_016015046.2
Amblyraja radiata gasdermin e	XP_032903802.1
Pan troglodytes gasdermin e	XP_001159685.3

Omitted from tree (severe truncation)

Hypsugopox virus gasdermin (pseudogene)	MK860688.1:c133573-132651

Vertebrate pyrin domain containing proteins

Protein	Accession
POP1 (Human)	NP_690865.1
ASC (Human)	NP_660183.1
Danio PYD protein	NP_001104231.1
ASC (Danio)	Q9I9N6.1
PYDC2 (Human)	NP_001076777.1
AIM2 (Mouse)	NP_001013801.2
IFI203 (Mouse)	BAE43039.1
Danio CASPb	NP_690840.2
Danio CASPa	NP_571580.1

M013L family

Protein	Accession
M013L	NP_051727.1
S013L	NP_051902.1
Deerpox M013L homolog	YP_227401.1
Yaba M013L homolog	NP_073403.1
Tanapox M013L homolog	ABQ43490.1
Swinepox m013L homolog	NP_570174.1

C1L family

Protein	Accession
Vaccinia C1 protein	P21036.1
Buffalopox C1 homolog	QCY53926.1
Cowpox C1 homolog	AGY97559.1
Rabbitpox C1 homolog	AAS49732.1
Horsepox C1 homolog	YP_010509239.1
Orthopox abatino C1 homolog	YP_010085697.1
Akhmeta virus C1 homolog	YP_010085480.1
Variola B22L (C1 homolog)	AAA69361.1
Monkeypox C1 homolog	YP_010377017.1
Alaskapox C1 homolog	QED21215.1
Volepox C1 homolog	YP_009281774.1
Skunkpox C1 homolog	YP_009282720.1
Raccoonpox C1 homolog	YP_009143336.1
NY014 poxvirus C1 homolog	YP_009408409.1
Murmansk poxvirus C1 homolog	YP_009408207.1
Yoka poxvirus C1 homolog	YP_004821365.1

Cloning

OLIGO
pCMV_hsGSDMD_N_R
pCMV_hsGSDMD_C_F
pCMV_hsGSDMD_C_R
pEF-Flag_F
pEF-C1_R
C1_PYD_r
C1_bcl2_fwd1
C1_bcl2_fwd2
cop_donor_c1_5pF
cop_donor_c1_5pR
cop_donor_c1_3pF
cop_donor_c1_3pR

PCR Genotyping for Recombinant Virus

OLIGO
a47_5p_flank_PCRfwd
a47_5p_flank_PCRrev_rec
a47_5p_flank_PCRrev_wt
a47_3p_flank_PCRfwd_rec
a47_3p_flank_PCRfwd_wt
a47_3p_flank_PCRrev
c1_5p_flank_PCRfwd
c1_5p_flank_PCRrev_wt
c1_3p_flank_PCRfwd_wt
c1_3p_flank_PCRrev

**SEQUENCE**
CGTCAActcgagctaATCTGTCAAGAAATTGTGGAG
gacctctagaaccATGGAACAAAAACTCATCTCAGAAGAGGATCTGggttctGGGGTCCCCGCTGAAG
CGTCAACTCGAGctaATGGGGTTCTTGGCTAAGACCGGAC
gaatCCCGGGaccatggactacaaagacg
CCACTAgcggccgcctaTTTGCTCATAAGTTCCTTC
CATAgcggccgcctaGAGTATAGCAAATCTGACTGCTG
gacgacaagGGTGGAGGTGGTACCatgGACAAAATGACCGAGGATATTAAGC
gacctctagaaccatggactacaaagacgatgacgacaagGGTGGAGG
**gctc**aagcttttcatcctttgataattttccaaattc
gctaCAATTGtttttttataaaaaatattgaatatataaacctttac
gcttCTCGAGttatttatttttcaccatatagatcaatcattagatc
gctaGAGCTCttcaaatgtttcattaactacgtg

**SEQUENCE**
cgattaatgccccacgagac
**GTGACCGTTTACGTCGCC**
**gagcttagtccgtttgtccac**
**GAGGACGTGACGGCTG**
ctgagaggtcaatacctaactcc
**cgtctacatgttctgccgtaac**
cggagacgccgtaataatttcc
**cctacgataggacacgtaaaagc**
**catcatctgtaagatttttgagatgtc**
**cgctaagcgaatgatcgc**

NOTES

PCR off of gBlock for full-length GSDMD
PCR off of gBlock for full-length GSDMD
PCR off of gBlock for full-length GSDMD
For all FLAG-tagged constructs (C1)
For C1 full length and C1 BCL2 domain
used with pEF-Flag_F
first PCR for BCL2 domain cloning: used with with pEF_C1_R
second PCR for BCL2 domain cloning: used with pEF_C1_R, had to add a third PCR round (pEF_C1_[illegible]
Used VC2 DNA as template to paste into cut pUC19_muGFP_A47_donor vector
Used VC2 DNA as template to paste into cut pUC19_muGFP_A47_donor vector
Used VC2 DNA as template to paste into cut pUC19_muGFP_A47_donor vector
Used VC2 DNA as template to paste into cut pUC19_muGFP_A47_donor vector

NOTES

expect:250bp
expect: 380bp

expect: 287bp
expect: 191
TM 57, for recomb: use with a47_5p_flank_PCRrev_rec: expect 270bp product, for WT use with c1_5[illegible]
TM56
TM 54
TM 55, for rec use with a47_5p_flank_PCRrev_wt, expect 220bp product, for WT expect 240 (with c1_[illegible]

R) to add correct cut site due to mistake in cloning design

p_flank_PCRfwd, expect 240bp product

_5p_flank_PCRrev_wt)

pCMV_vvGSDM	This Study
pCMV_GSDMD_CTD	This Study
pEF1α_C1	This Study
pEF1α_C1_PYD	This Study
pEF1α_C1_BCL2	This Study
pUC19_muGFP_Donor_A47	This Study
pUC19_muGFP_Donor_C1	This Study
pET_SUMO_eptvGSDM	This Study

pLEX-MCS-ASC-GFP was a gift from Christian Stehlik (Addgene, catalog number: 73957)
pCMV-pro-IL1β-C-Flag was a kind gift from Bruce Beutler (Addgene, catalog number: 75131)
pcDNA3-N-Flag-NLRP3 was a kind gift from Bruce Beutler (Addgene, catalog number: 75127)
pcDNA3-N-Flag-ASC1 was a kind gift from Bruce Beutler (Addgene, catalog number: 75134)
pcDNA3-N-Flag-Caspase-1 was a kind gift from Bruce Beutler (Addgene, catalog number: 75128)
pcDNA3-N-HA-NEK7 was a kind gift from Bruce Beutler (Addgene, catalog number: 75142)

Table S1. Crystallographic statistics, Related to Figure 2 and Figure NN

	EPTV A47L	EPTV A47L (SeMet)
**Data Collection**		
Resolution (Å)[a]	48.65–1.46 (1.48–1.46)	48.39–1.54 (1.57–1.54)
Wavelength (Å)	0.9792	0.9792
Space group	C 2 2 2_1	C 2 2 2_1
Unit cell: a, b, c (Å)	64.46 74.14 95.60	63.51, 74.71, 95.29
Unit cell: α, β, γ (°)	90.00 90.00 90.00	90.00, 90.00, 90.00
Molecules per ASU	1	1
Total reflections	543256	920245
Unique reflections	40114	33934
Completeness (%)[a]	100.0 (99.6)	100.0 (99.9)
Multiplicity[a]	13.5 (13.2)	27.1 (23.4)
I/σI[a]	18.3 (1.6)	15.7 (2.1)
CC(1/2)[b] (%)[a]	99.9 (50.5)	99.9 (88.8)
Rpim[c] (%)[a]	1.9 (71.8)	3.0 (68.9)
Sites		5
**Refinement**		
Resolution (Å)	48.65–1.46	
Free reflections	40033	
R-factor / R-free	19.0 / 21.2	
Bond distance (RMS Å)	0.01	
Bond angles (RMS °)	1.10	
**Structure/Stereochemistry**		
No. atoms: protein	1658	
No. atoms: water	85	
Average B-factor: protein	32.8	
Average B-factor: water	43.1	
Ramachandran plot: favored	99.01%	
Ramachandran plot: allowed	0.99%	
Ramachandran plot: outliers	0.00%	
Rotamer outliers	0.52%	
MolProbity[d] score	1.05	
Protein Data Bank ID	**TBD**	

[a] Highest resolution shell values in parentheses
[b] (Karplus and Diederichs, 2012)
[c] (Weiss, 2001)
[d] (Chen et al., 2010)

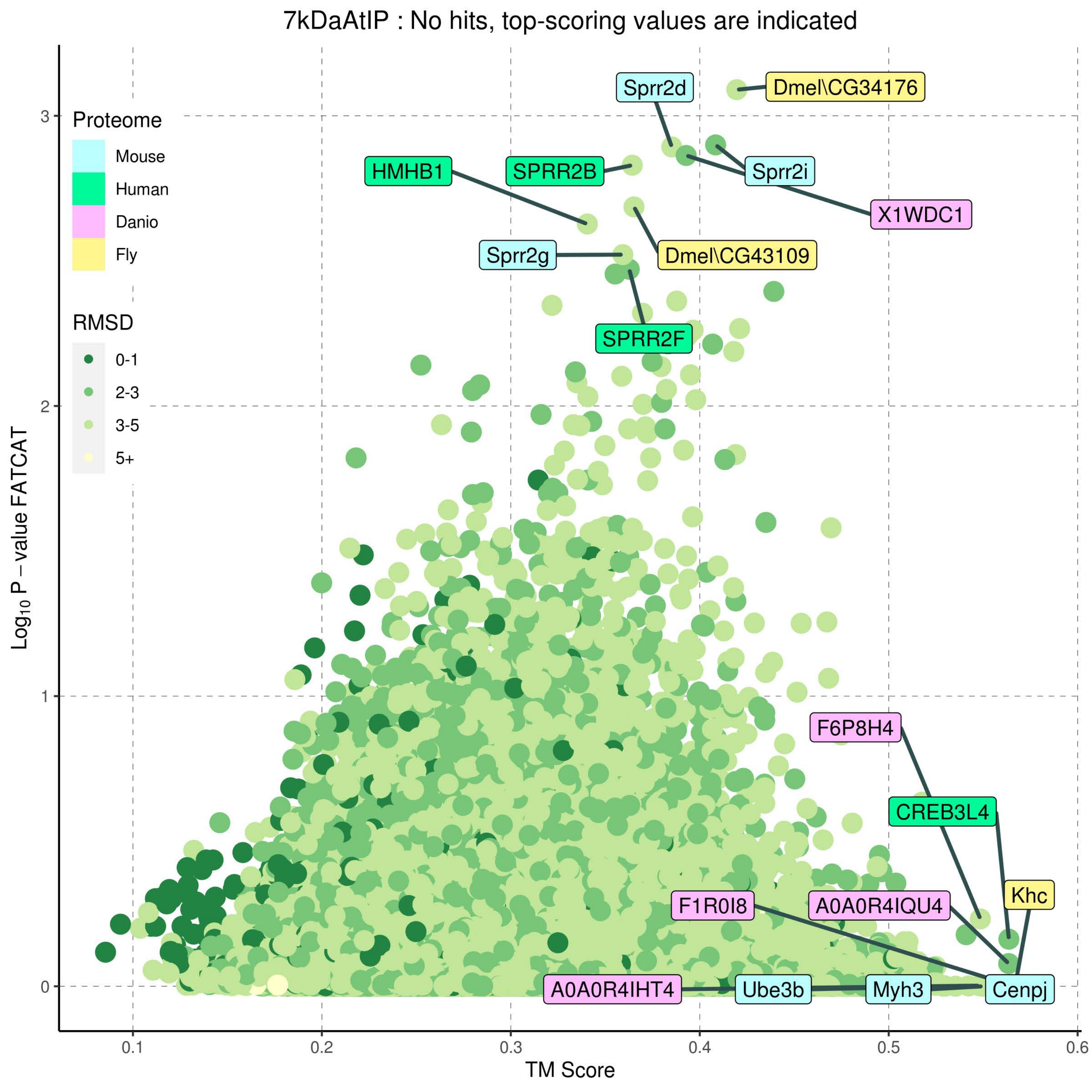

7kDaAtIP : No hits, top-scoring values are indicated
Proteome
Mouse
Human
Danio
Fly
RMSD
0-1
2-3
3-5
5+
Sprr2d
Dmel\CG34176
HMHB1
SPRR2B
Sprr2i
X1WDC1
Sprr2g
Dmel\CG43109
SPRR2F
F6P8H4
CREB3L4
Khc
F1R0I8
A0A0R4IQU4
A0A0R4IHT4
Ube3b
Myh3
Cenpj
0
1
2
3
0.1
0.2
0.3
0.4
0.5
0.6
TM Score
Log10 P − value FATCAT

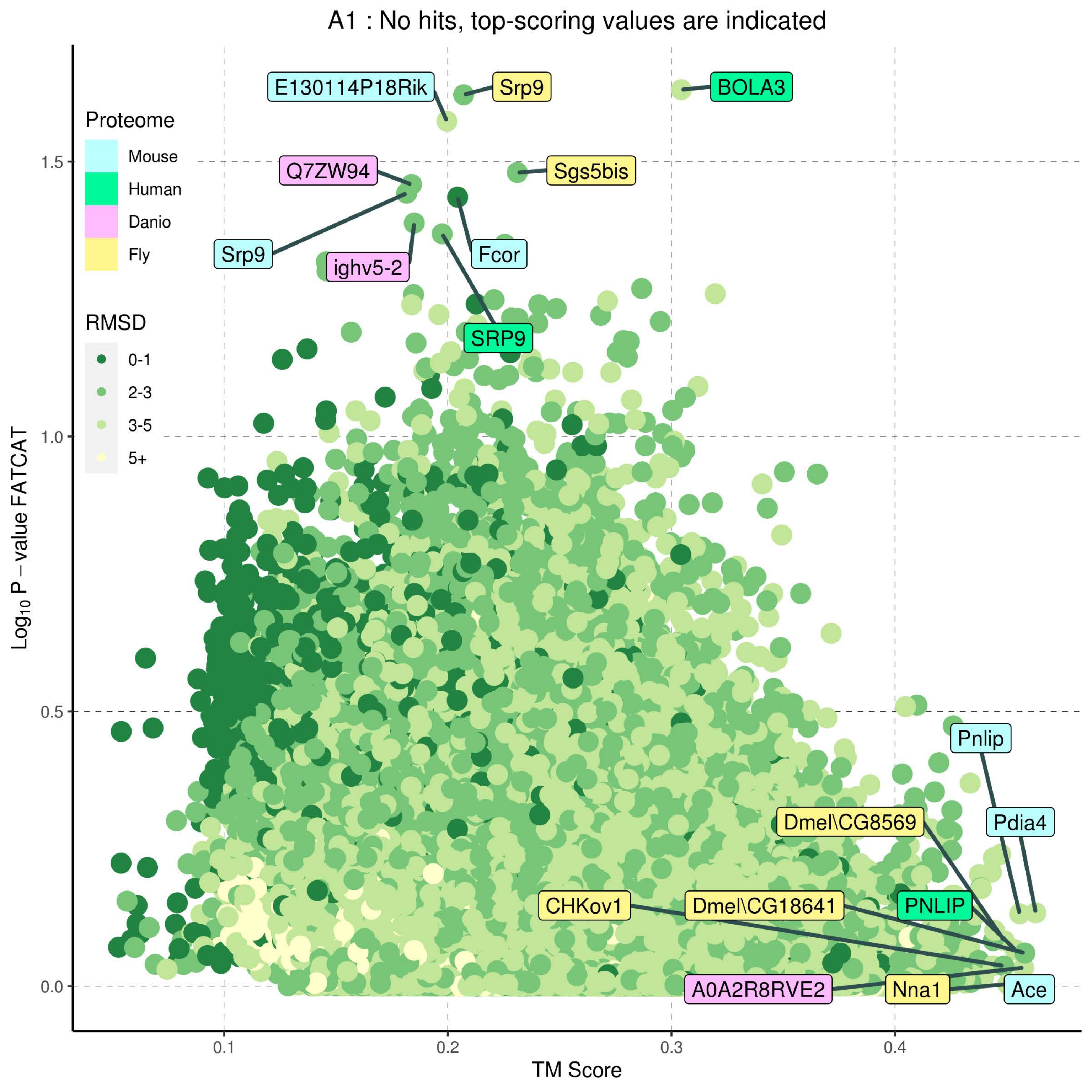

A1 : No hits, top-scoring values are indicated
Proteome
Mouse
Human
Danio
Fly
RMSD
0-1
2-3
3-5
5+
E130114P18Rik
Srp9
BOLA3
Q7ZW94
Sgs5bis
Srp9
ighv5-2
Fcor
SRP9
Pnlip
Pdia4
Dmel\CG8569
CHKov1
Dmel\CG18641
PNLIP
A0A2R8RVE2
Nna1
Ace
1.5
1.0
0.5
0.0
0.1
0.2
0.3
0.4
TM Score
Log_{10} P − value FATCAT

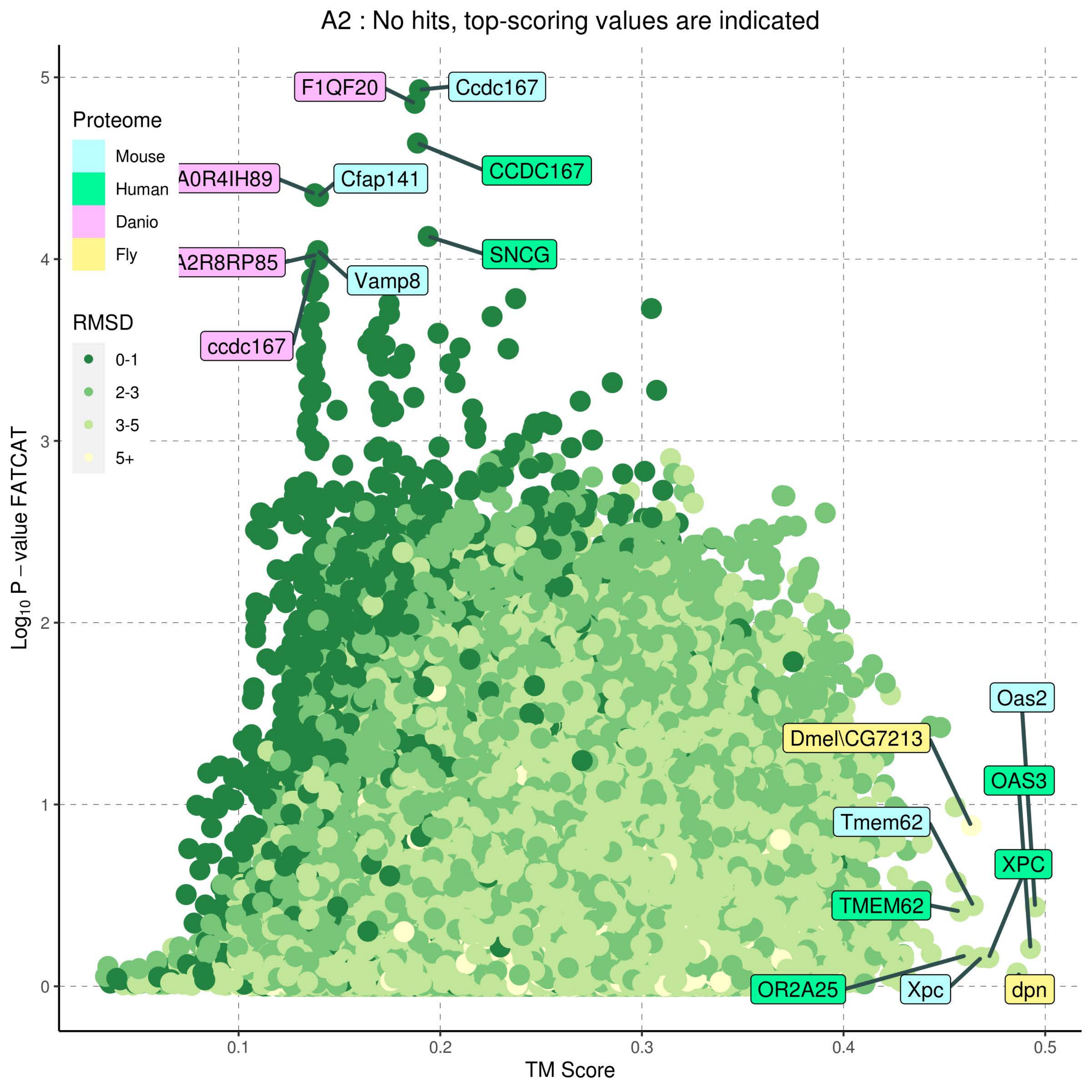
A2 : No hits, top-scoring values are indicated
Proteome
Mouse
Human
Danio
Fly
RMSD
0-1
2-3
3-5
5+
F1QF20
Ccdc167
CCDC167
A0R4IH89
Cfap141
SNCG
A2R8RP85
Vamp8
ccdc167
Oas2
Dmel\CG7213
OAS3
Tmem62
XPC
TMEM62
OR2A25
Xpc
dpn
Log10 P – value FATCAT
TM Score
0
1
2
3
4
5
0.1
0.2
0.3
0.4
0.5

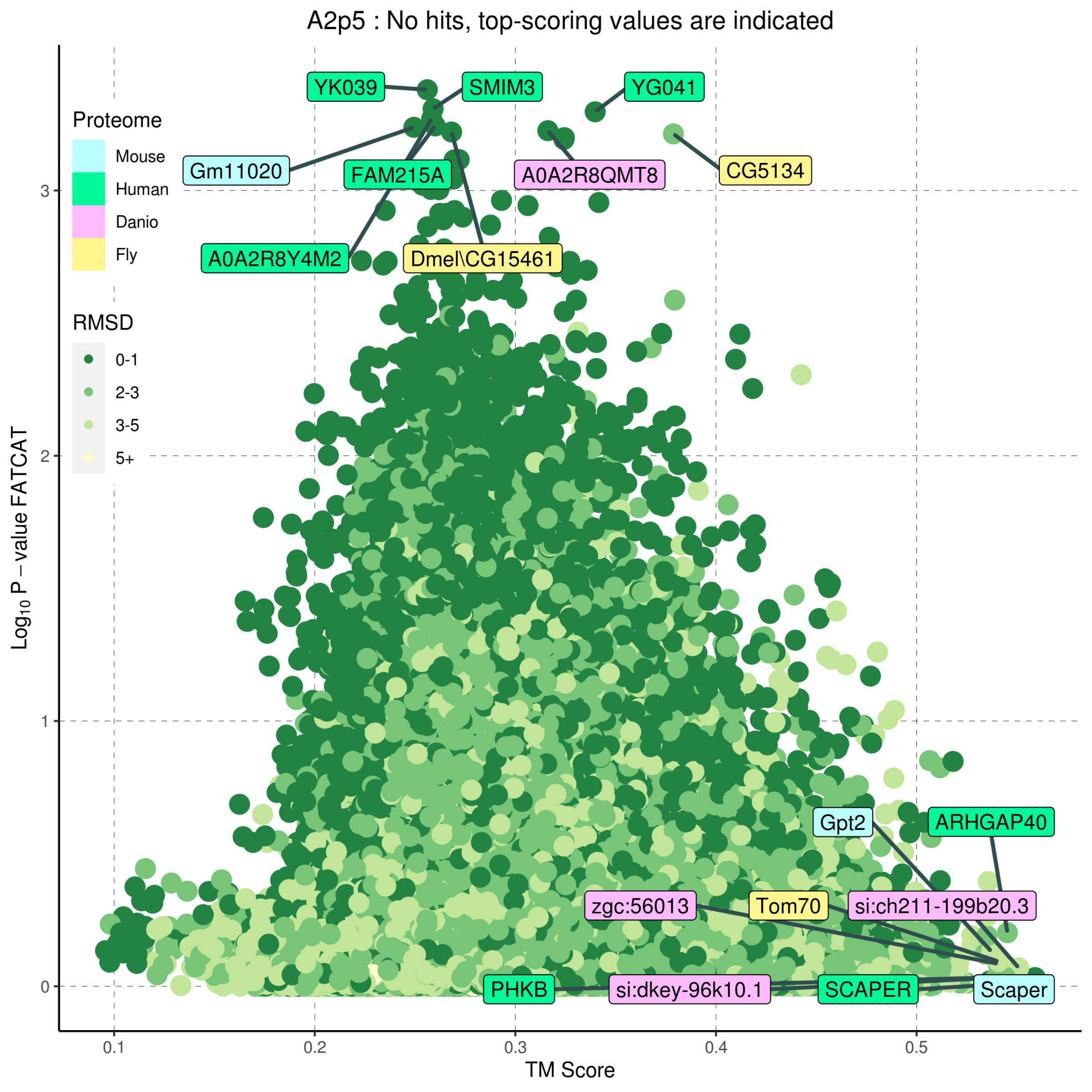

A2p5 : No hits, top-scoring values are indicated
Proteome
Mouse
Human
Danio
Fly
RMSD
0-1
2-3
3-5
5+
YK039
SMIM3
YG041
Gm11020
FAM215A
A0A2R8QMT8
CG5134
A0A2R8Y4M2
Dmel\CG15461
Gpt2
ARHGAP40
zgc:56013
Tom70
si:ch211-199b20.3
PHKB
si:dkey-96k10.1
SCAPER
Scaper
Log_{10} P – value FATCAT
TM Score
0
1
2
3
0.1
0.2
0.3
0.4
0.5

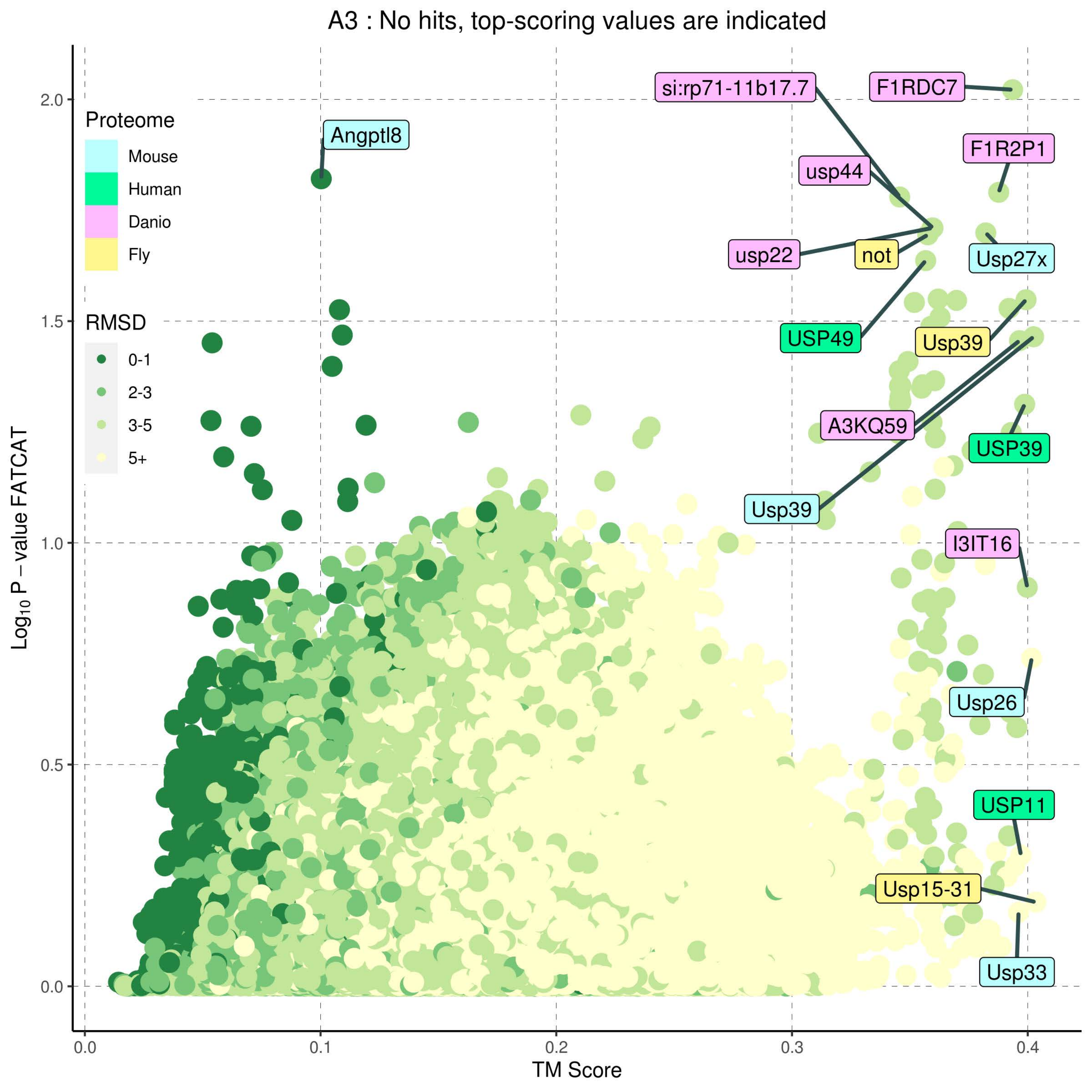
A3 : No hits, top-scoring values are indicated
Proteome
Mouse
Human
Danio
Fly
RMSD
0-1
2-3
3-5
5+
Log10 P − value FATCAT
TM Score
0.0
0.5
1.0
1.5
2.0
0.0
0.1
0.2
0.3
0.4
Angptl8
si:rp71-11b17.7
F1RDC7
F1R2P1
usp44
usp22
not
Usp27x
USP49
Usp39
A3KQ59
USP39
Usp39
I3IT16
Usp26
USP11
Usp15-31
Usp33

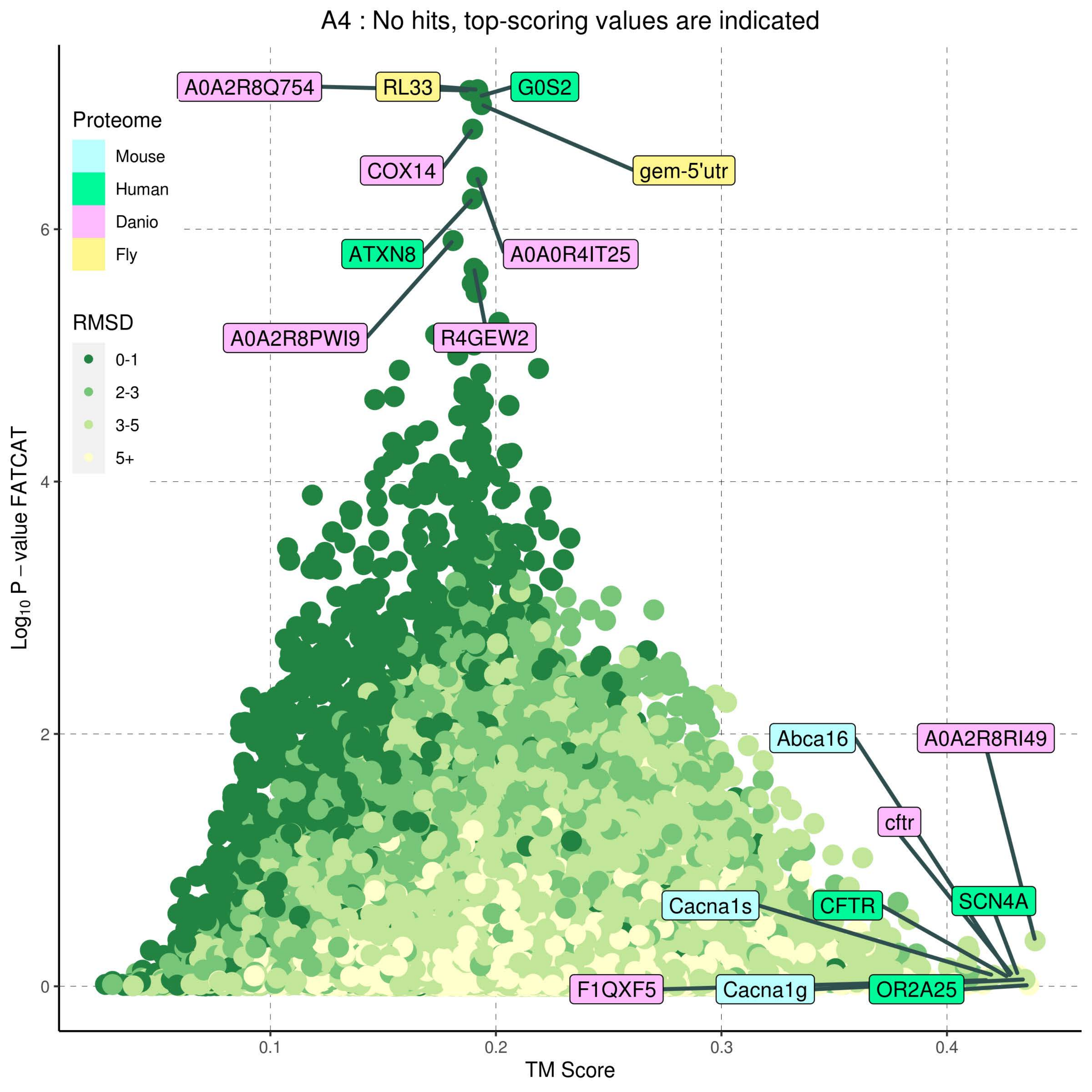
A4 : No hits, top-scoring values are indicated
Proteome
Mouse
Human
Danio
Fly
RMSD
0-1
2-3
3-5
5+
A0A2R8Q754
RL33
G0S2
gem-5'utr
COX14
ATXN8
A0A0R4IT25
A0A2R8PWI9
R4GEW2
Abca16
A0A2R8RI49
cftr
SCN4A
Cacna1s
CFTR
F1QXF5
Cacna1g
OR2A25
Log10 P − value FATCAT
TM Score
0
2
4
6
0.1
0.2
0.3
0.4

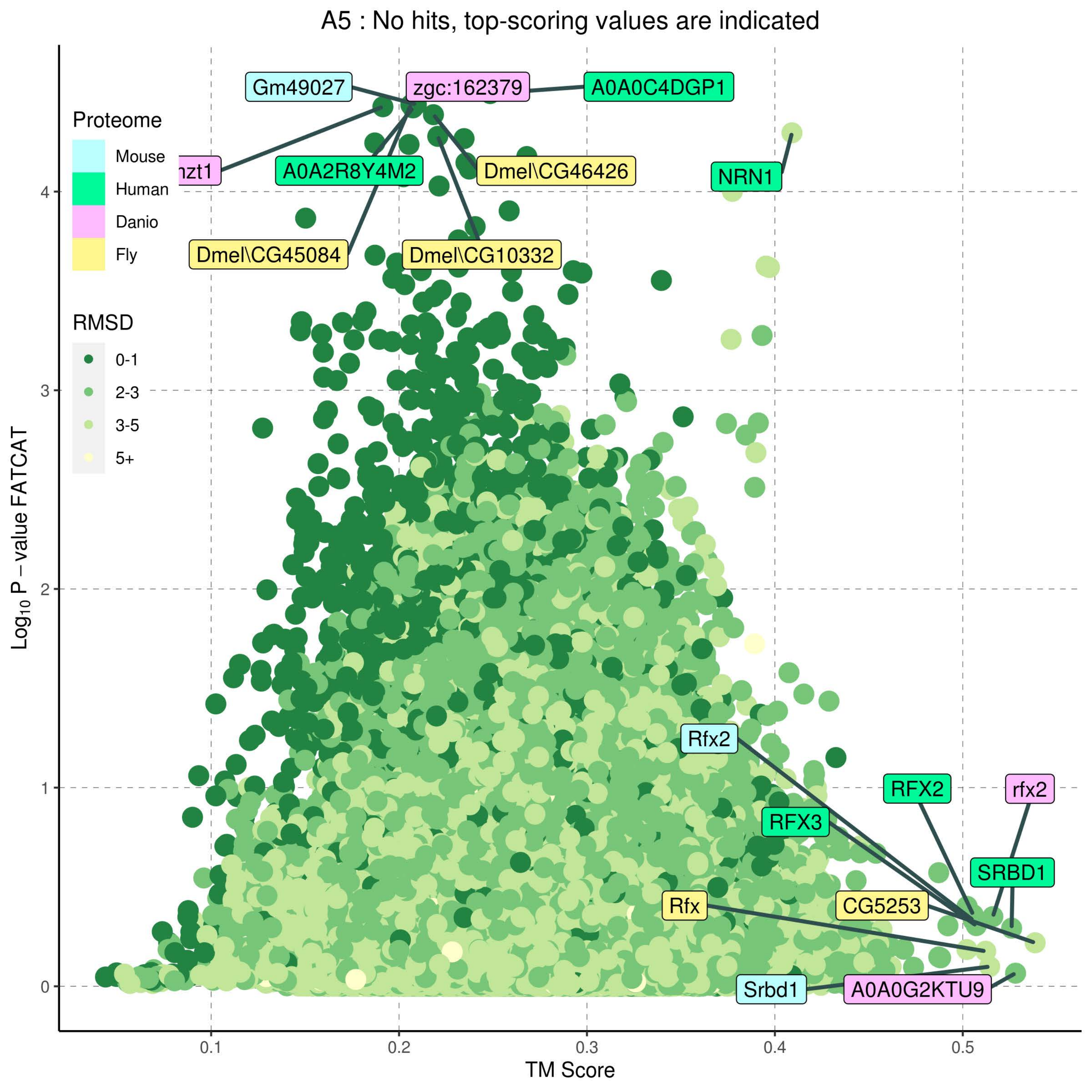

A5 : No hits, top-scoring values are indicated
Proteome
Mouse
Human
Danio
Fly
RMSD
0-1
2-3
3-5
5+
Gm49027
zgc:162379
A0A0C4DGP1
nzt1
A0A2R8Y4M2
Dmel\CG46426
NRN1
Dmel\CG45084
Dmel\CG10332
Rfx2
RFX2
rfx2
RFX3
SRBD1
Rfx
CG5253
Srbd1
A0A0G2KTU9
Log10 P – value FATCAT
TM Score
0
1
2
3
4
0.1
0.2
0.3
0.4
0.5

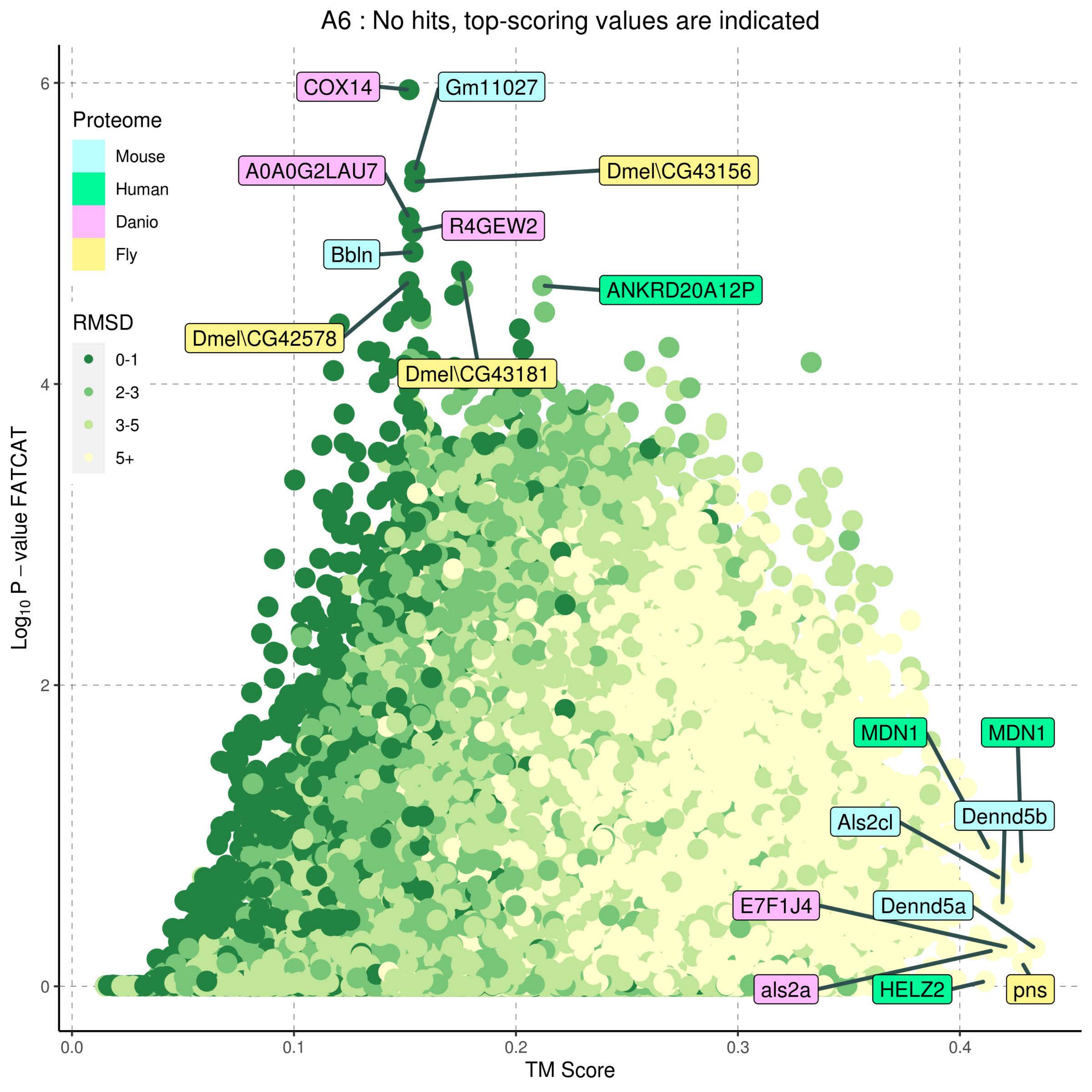

A6 : No hits, top-scoring values are indicated
Proteome
Mouse
Human
Danio
Fly
RMSD
0-1
2-3
3-5
5+
COX14
Gm11027
A0A0G2LAU7
Dmel\CG43156
R4GEW2
Bbln
ANKRD20A12P
Dmel\CG42578
Dmel\CG43181
MDN1
MDN1
Dennd5b
Als2cl
E7F1J4
Dennd5a
als2a
HELZ2
pns
Log10 P – value FATCAT
TM Score
0
2
4
6
0.0
0.1
0.2
0.3
0.4

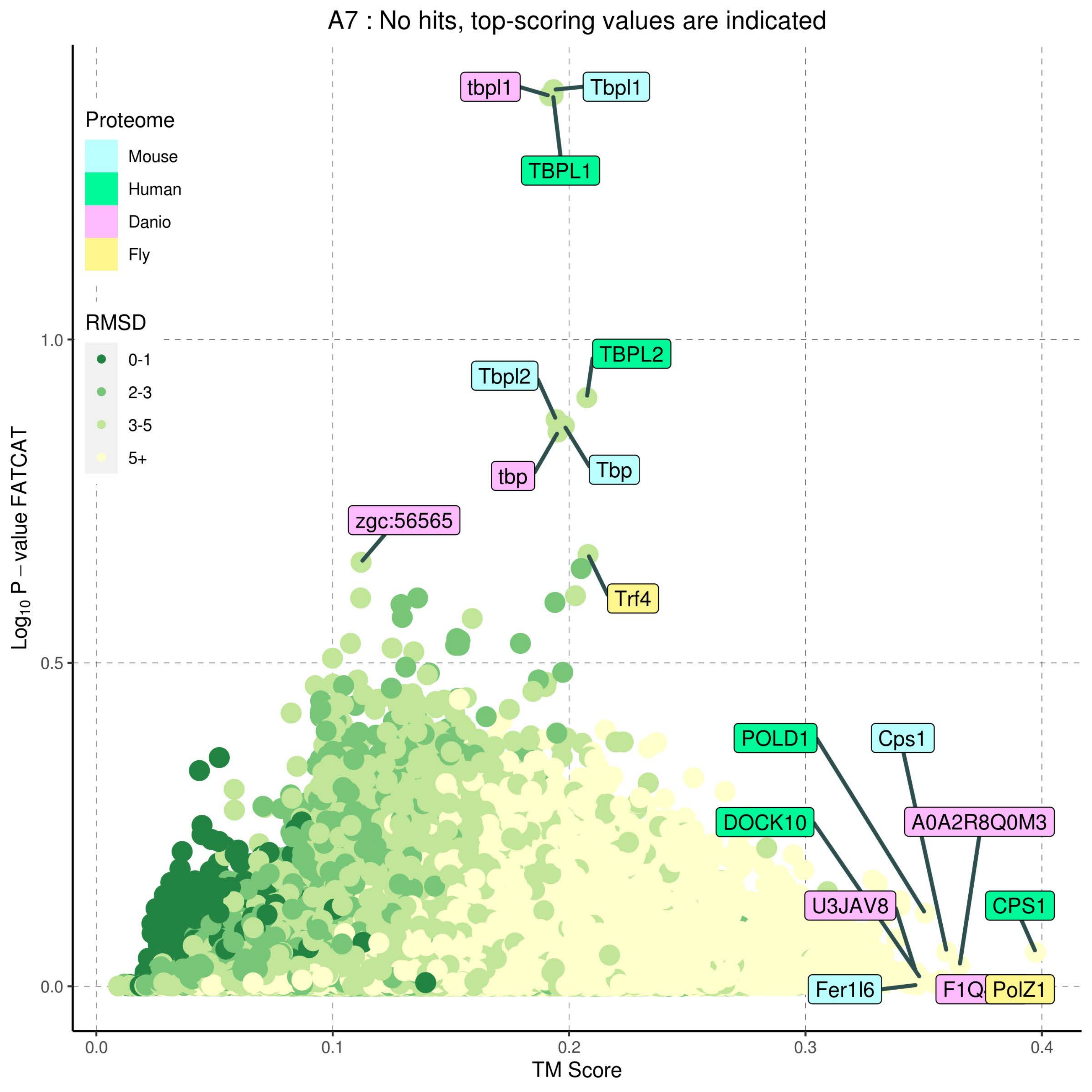

A7 : No hits, top-scoring values are indicated
Proteome
Mouse
Human
Danio
Fly
RMSD
0-1
2-3
3-5
5+
tbpl1
Tbpl1
TBPL1
TBPL2
Tbpl2
tbp
Tbp
zgc:56565
Trf4
POLD1
Cps1
DOCK10
A0A2R8Q0M3
U3JAV8
CPS1
Fer1l6
F1Q
PolZ1
Log10 P – value FATCAT
TM Score
0.0
0.5
1.0
0.0
0.1
0.2
0.3
0.4

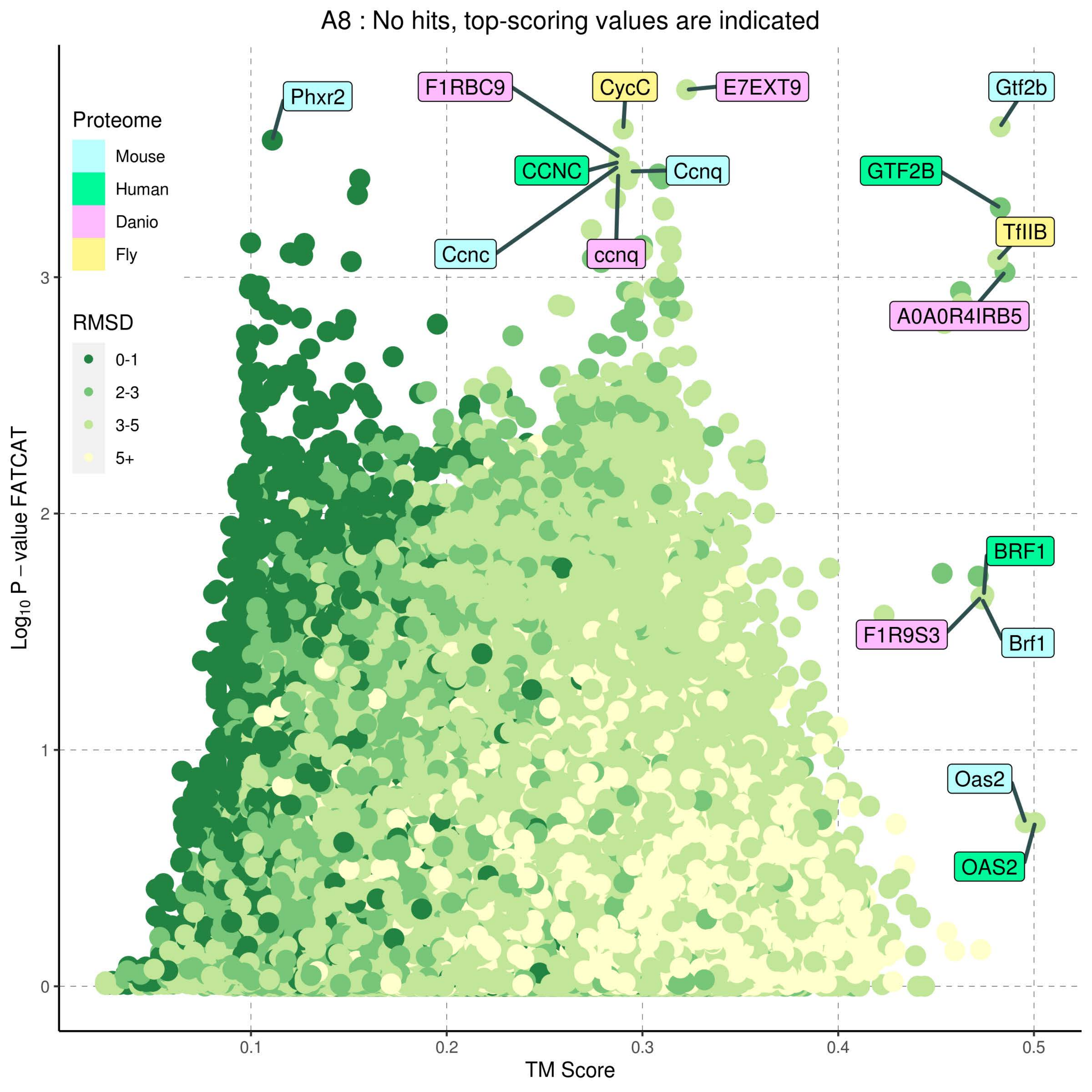
A8 : No hits, top-scoring values are indicated
Proteome
Mouse
Human
Danio
Fly
RMSD
0-1
2-3
3-5
5+
Phxr2
F1RBC9
CycC
E7EXT9
Gtf2b
CCNC
Ccnq
GTF2B
Ccnc
ccnq
TfIIB
A0A0R4IRB5
BRF1
F1R9S3
Brf1
Oas2
OAS2
Log10 P – value FATCAT
0
1
2
3
TM Score
0.1
0.2
0.3
0.4
0.5

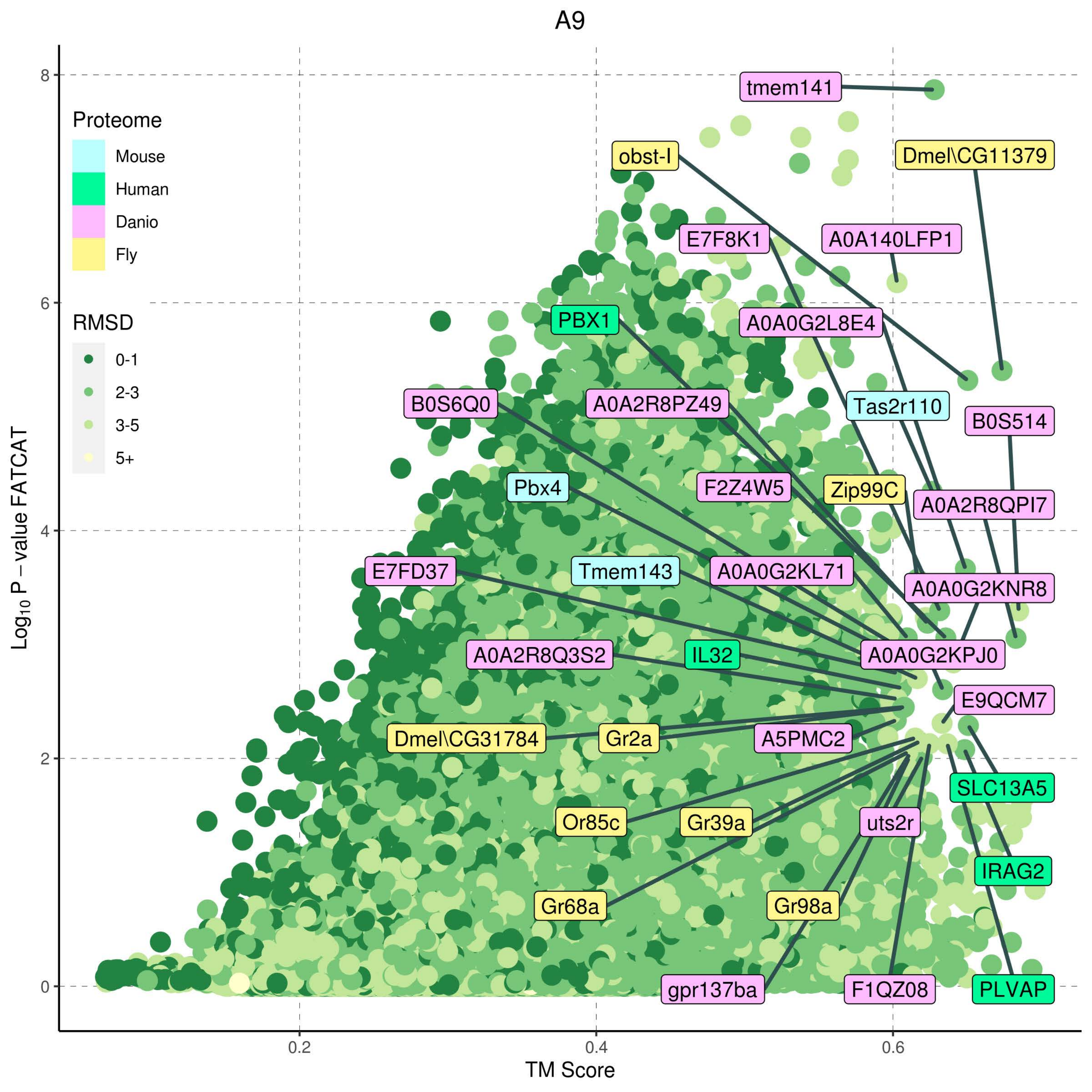
A9
Proteome
Mouse
Human
Danio
Fly
RMSD
0-1
2-3
3-5
5+
tmem141
obst-I
Dmel\CG11379
E7F8K1
A0A140LFP1
PBX1
A0A0G2L8E4
B0S6Q0
A0A2R8PZ49
Tas2r110
B0S514
Pbx4
F2Z4W5
Zip99C
A0A2R8QPI7
E7FD37
Tmem143
A0A0G2KL71
A0A0G2KNR8
A0A2R8Q3S2
IL32
A0A0G2KPJ0
E9QCM7
Dmel\CG31784
Gr2a
A5PMC2
SLC13A5
Or85c
Gr39a
uts2r
IRAG2
Gr68a
Gr98a
gpr137ba
F1QZ08
PLVAP
Log10 P − value FATCAT
TM Score
0
2
4
6
8
0.2
0.4
0.6

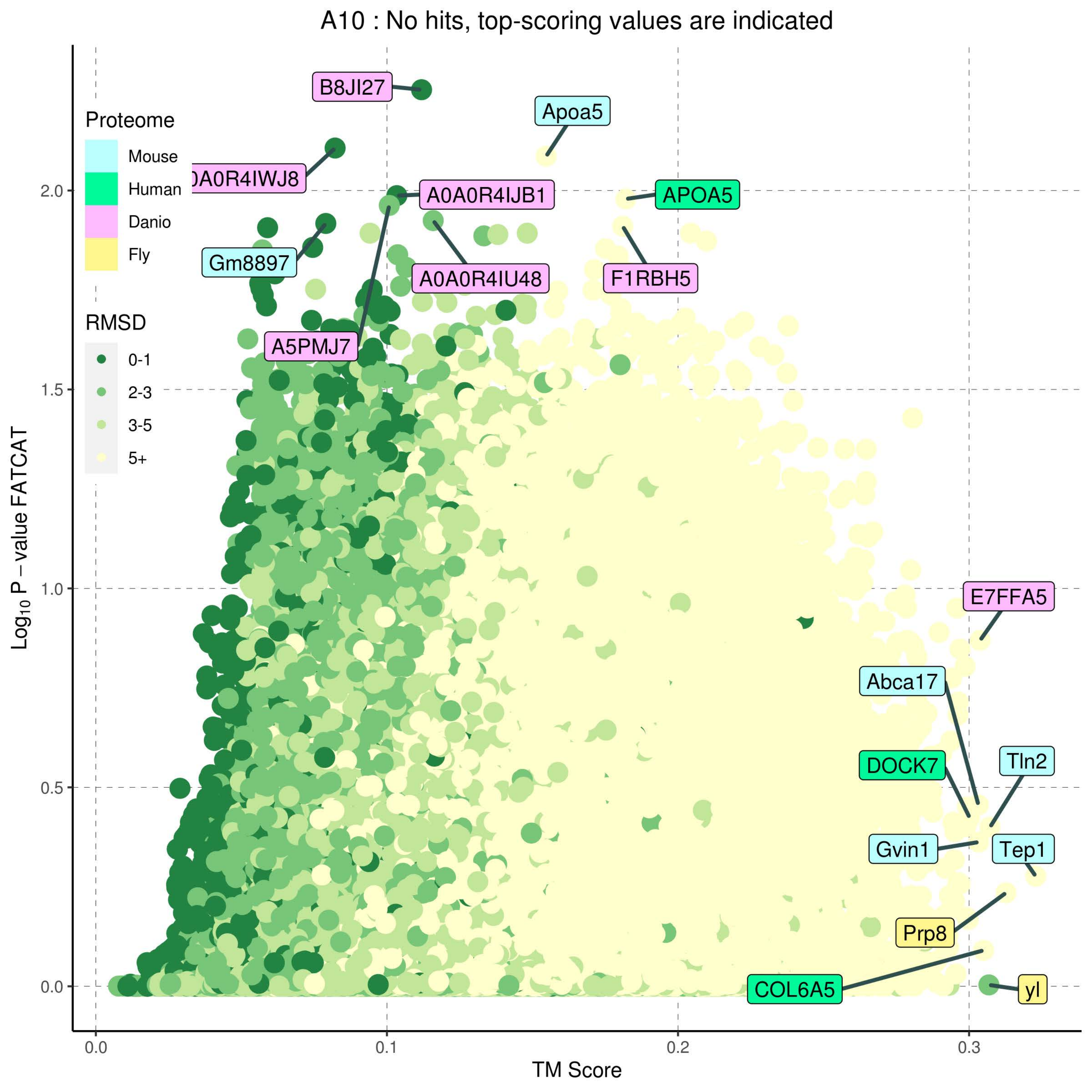

A10 : No hits, top-scoring values are indicated
Proteome
Mouse
Human
Danio
Fly
RMSD
0-1
2-3
3-5
5+
B8JI27
Apoa5
0A0R4IWJ8
A0A0R4IJB1
APOA5
Gm8897
A0A0R4IU48
F1RBH5
A5PMJ7
E7FFA5
Abca17
DOCK7
Tln2
Gvin1
Tep1
Prp8
COL6A5
yl
2.0
1.5
1.0
0.5
0.0
0.1
0.2
0.3
TM Score
Log_{10} P – value FATCAT

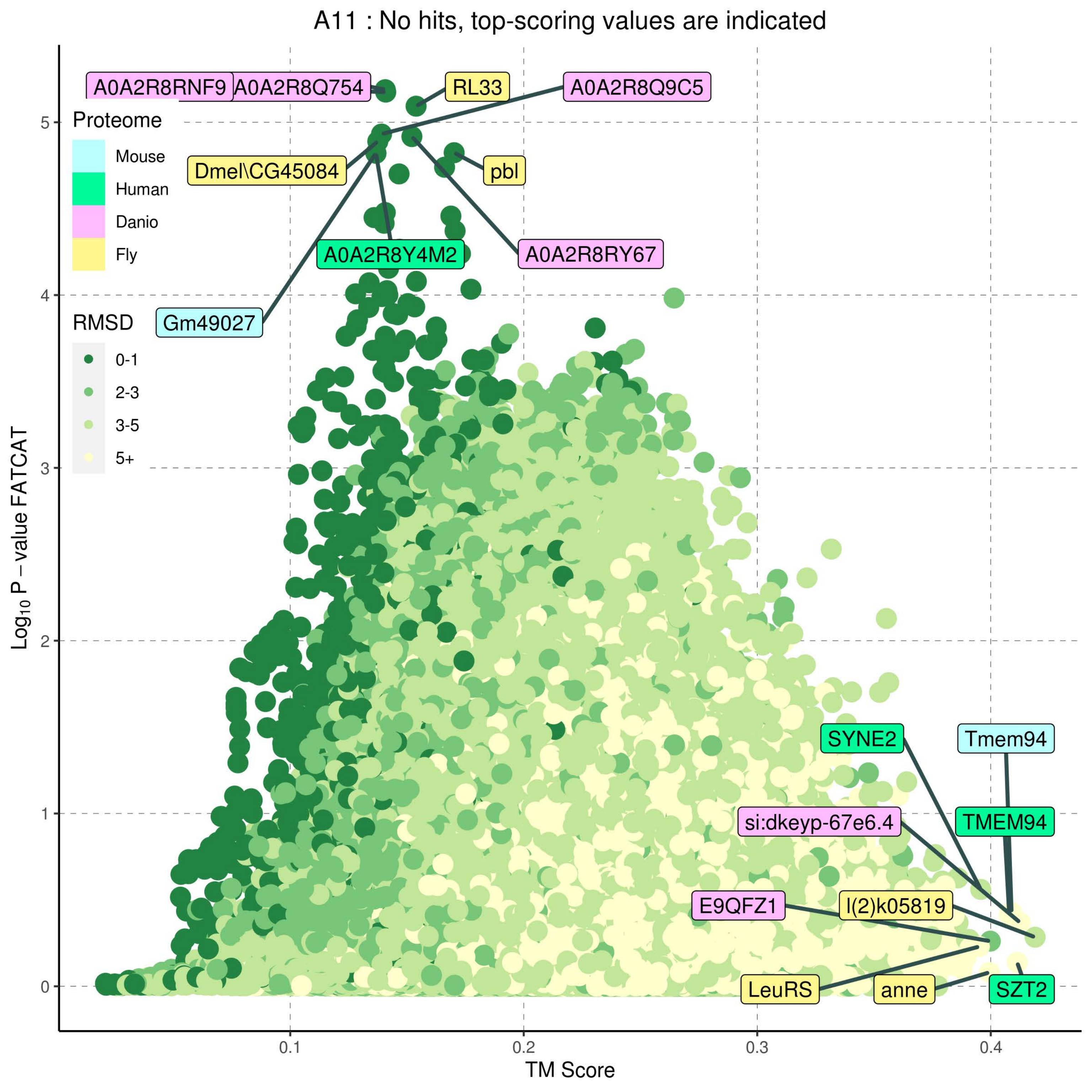
A11 : No hits, top-scoring values are indicated
A0A2R8RNF9
A0A2R8Q754
RL33
A0A2R8Q9C5
Dmel\CG45084
pbl
A0A2R8Y4M2
A0A2R8RY67
Gm49027
SYNE2
Tmem94
si:dkeyp-67e6.4
TMEM94
E9QFZ1
l(2)k05819
LeuRS
anne
SZT2
Proteome
Mouse
Human
Danio
Fly
RMSD
0-1
2-3
3-5
5+
Log_{10} P – value FATCAT
TM Score
0
1
2
3
4
5
0.1
0.2
0.3
0.4

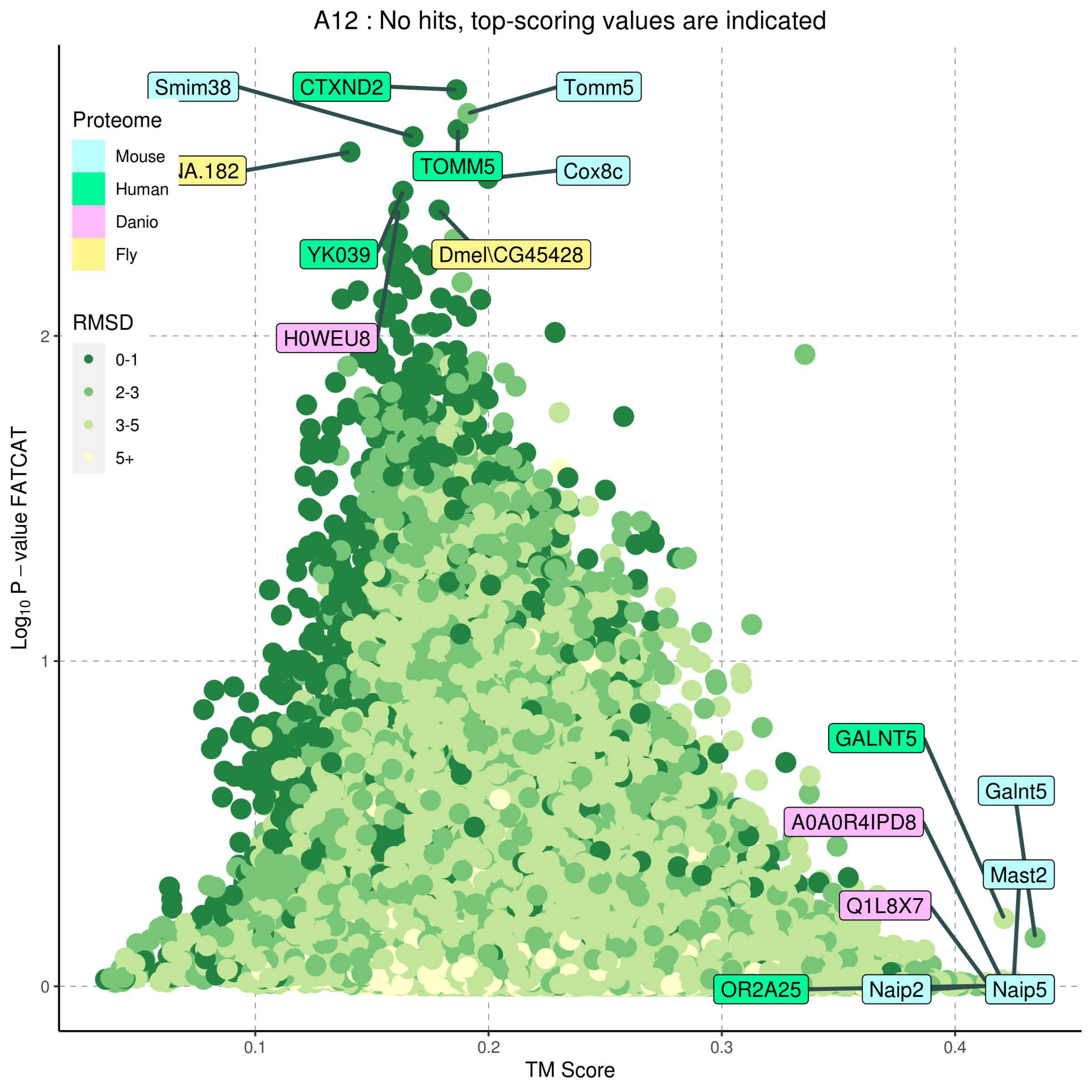

A12 : No hits, top-scoring values are indicated
Proteome
Mouse
Human
Danio
Fly
RMSD
0-1
2-3
3-5
5+
Smim38
CTXND2
Tomm5
NA.182
TOMM5
Cox8c
YK039
Dmel\CG45428
H0WEU8
GALNT5
Galnt5
A0A0R4IPD8
Mast2
Q1L8X7
OR2A25
Naip2
Naip5
Log10 P − value FATCAT
TM Score
0
1
2
0.1
0.2
0.3
0.4

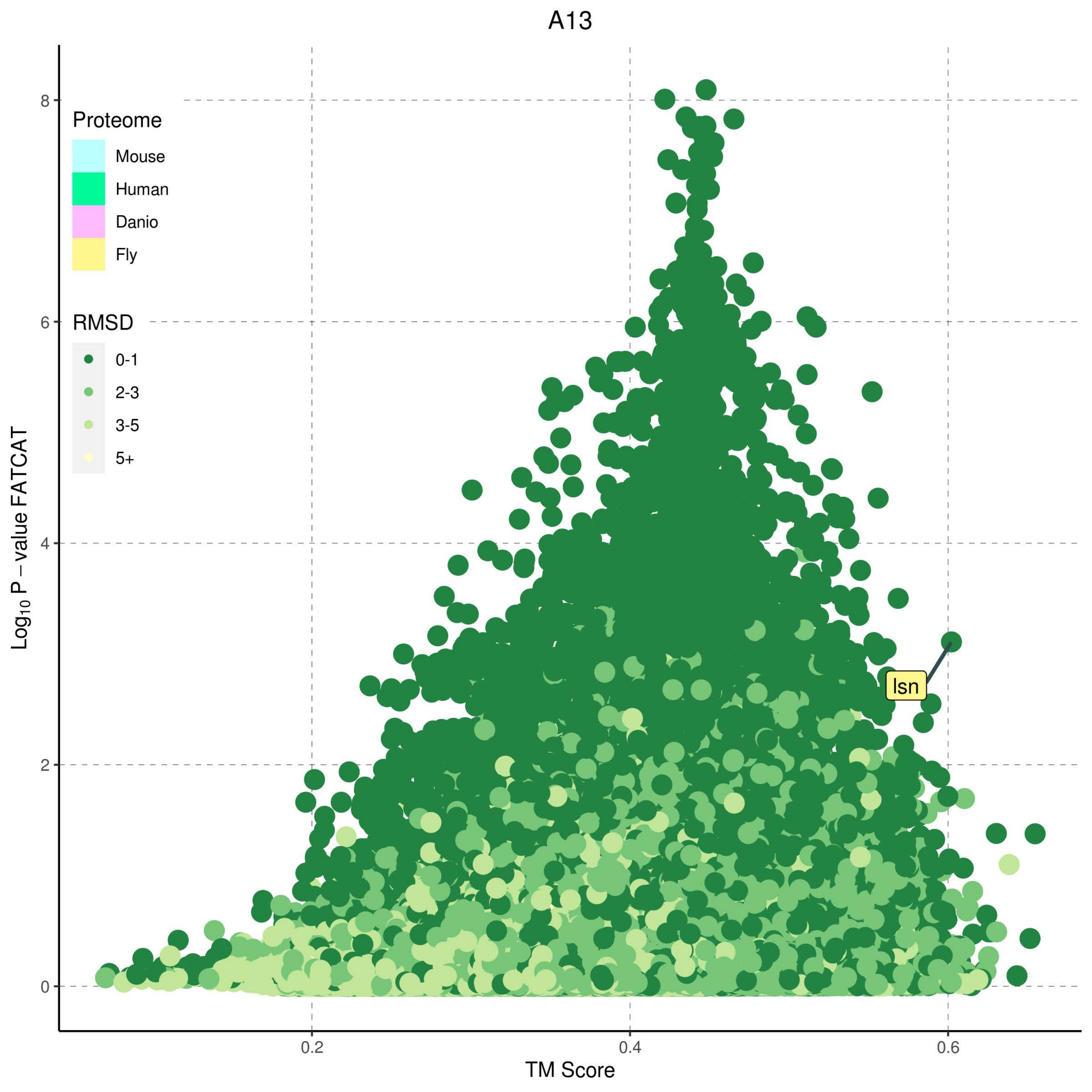

A13
Proteome
Mouse
Human
Danio
Fly
RMSD
0-1
2-3
3-5
5+
Isn
Log10 P − value FATCAT
TM Score
0
2
4
6
8
0.2
0.4
0.6

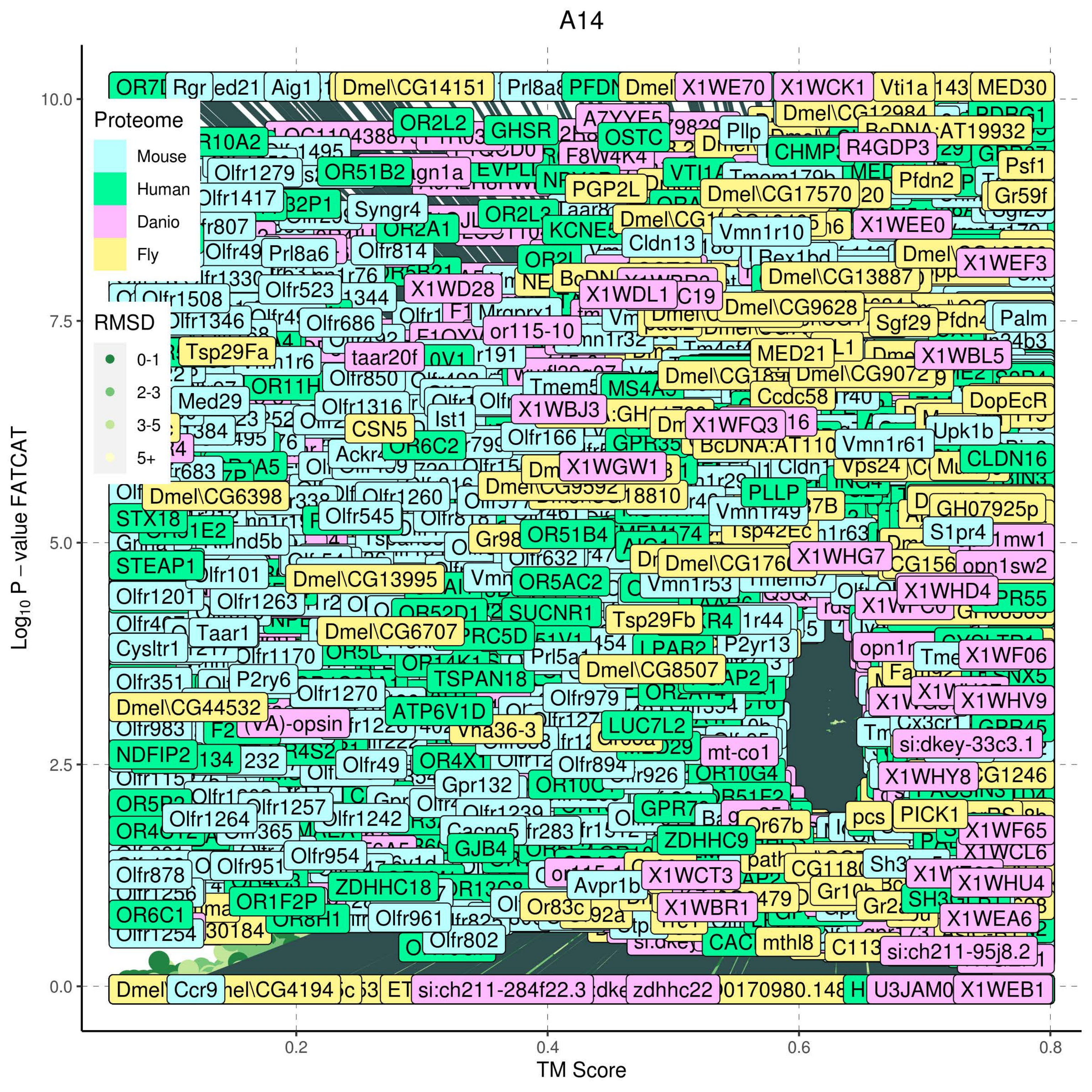

A14
Proteome
Mouse
Human
Danio
Fly
RMSD
0-1
2-3
3-5
5+
Log$_{10}$ P − value FATCAT
TM Score
0.0
2.5
5.0
7.5
10.0
0.2
0.4
0.6
0.8

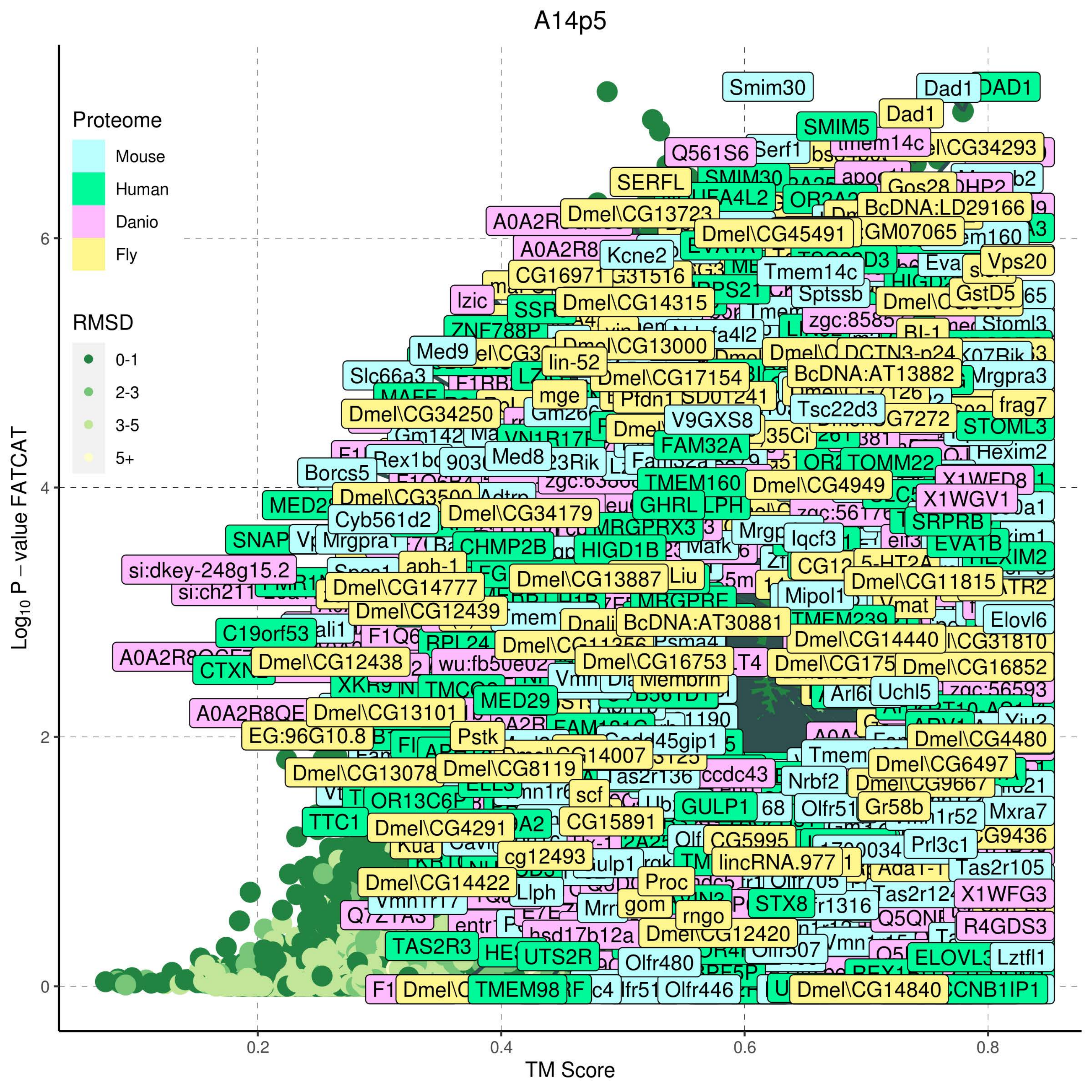

A14p5
Proteome
Mouse
Human
Danio
Fly
RMSD
0-1
2-3
3-5
5+
Log10 P − value FATCAT
0
2
4
6
TM Score
0.2
0.4
0.6
0.8

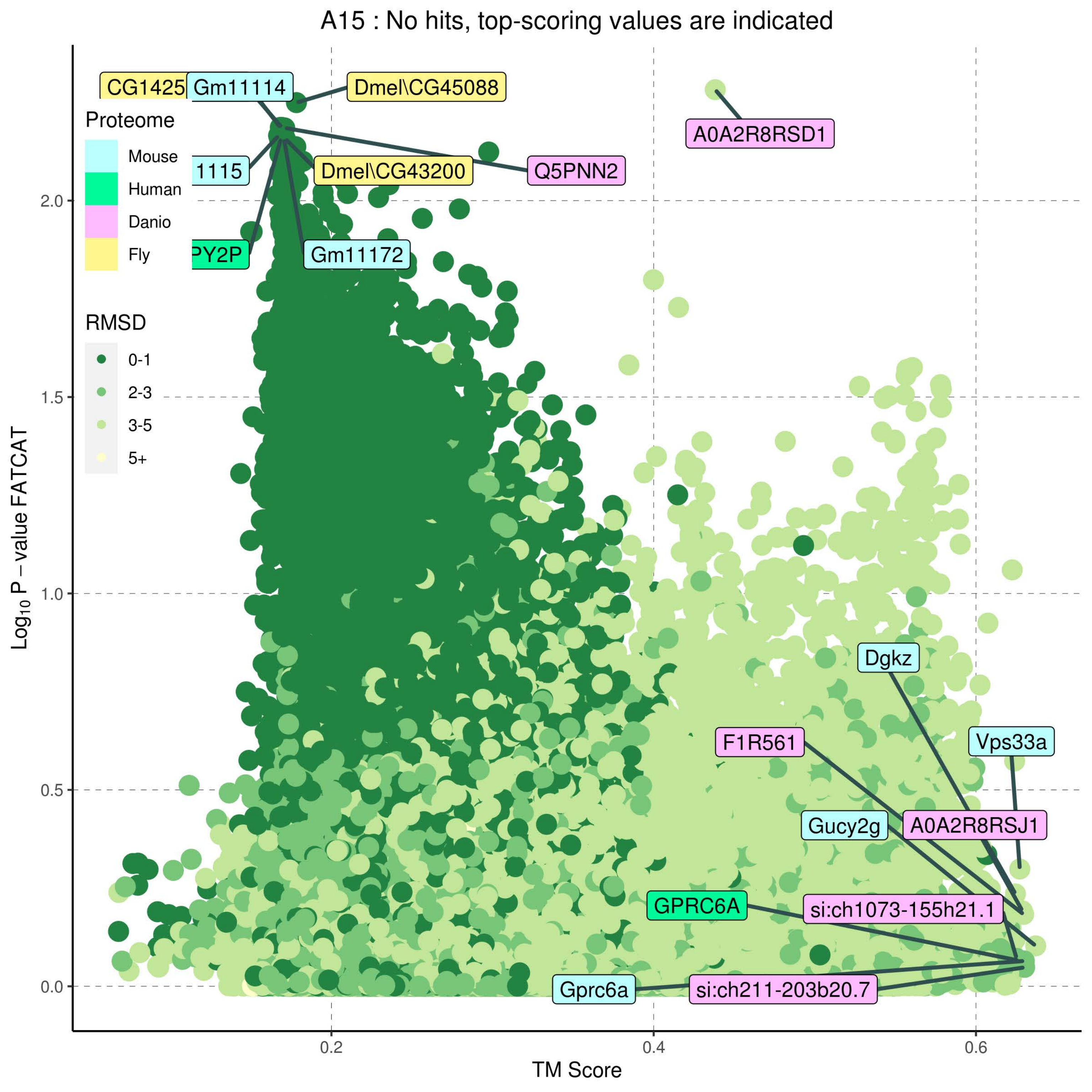

A15 : No hits, top-scoring values are indicated
Proteome
Mouse
Human
Danio
Fly
RMSD
0-1
2-3
3-5
5+
CG1425
Gm11114
Dmel\CG45088
A0A2R8RSD1
1115
Dmel\CG43200
Q5PNN2
PY2P
Gm11172
Dgkz
F1R561
Vps33a
Gucy2g
A0A2R8RSJ1
GPRC6A
si:ch1073-155h21.1
Gprc6a
si:ch211-203b20.7
Log10 P − value FATCAT
TM Score
0.0
0.5
1.0
1.5
2.0
0.2
0.4
0.6

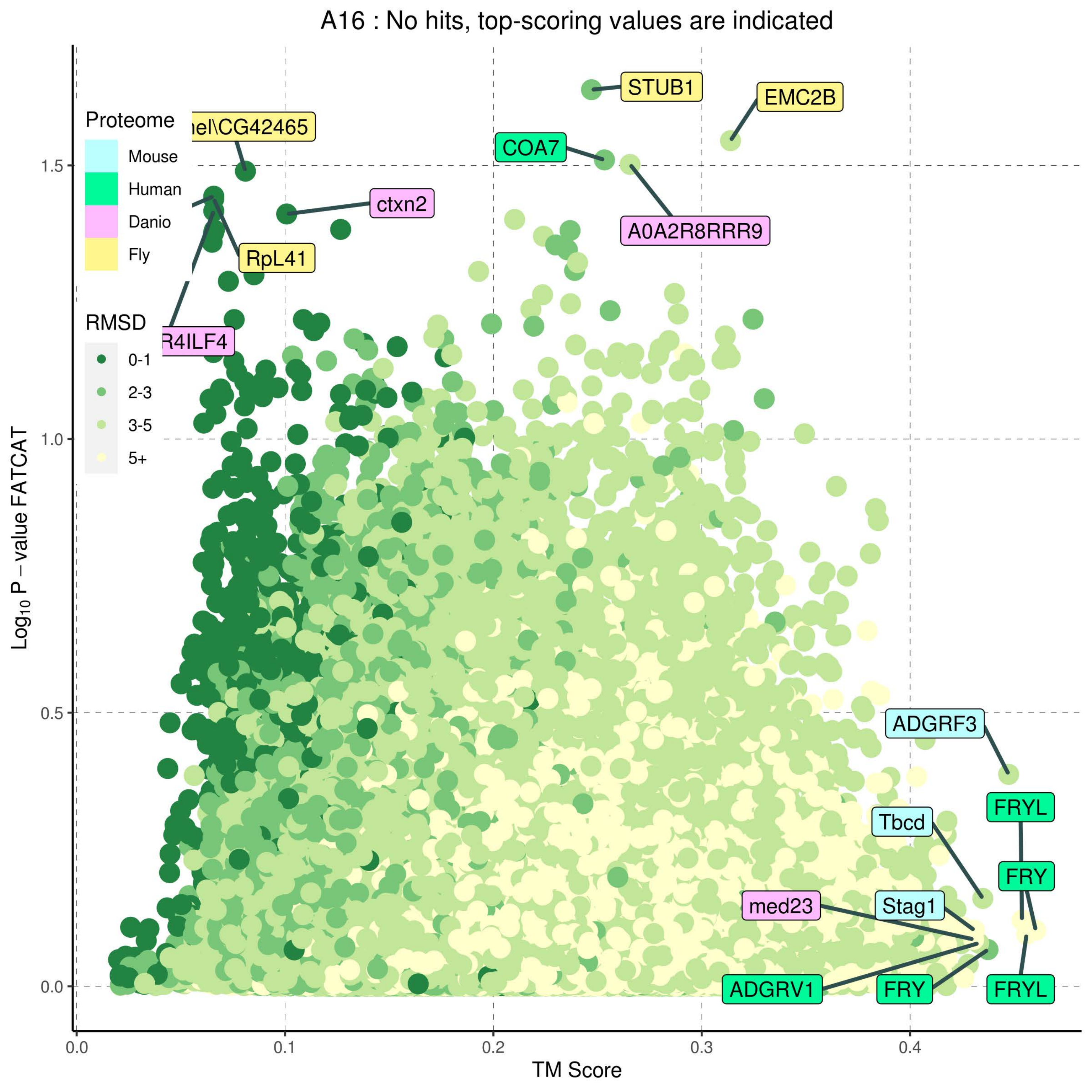

A16 : No hits, top-scoring values are indicated
Proteome
Mouse
Human
Danio
Fly
RMSD
0-1
2-3
3-5
5+
Log10 P − value FATCAT
TM Score
0.0
0.5
1.0
1.5
0.0
0.1
0.2
0.3
0.4
STUB1
EMC2B
nel\CG42465
COA7
ctxn2
A0A2R8RRR9
RpL41
R4ILF4
ADGRF3
FRYL
Tbcd
FRY
med23
Stag1
ADGRV1
FRY
FRYL

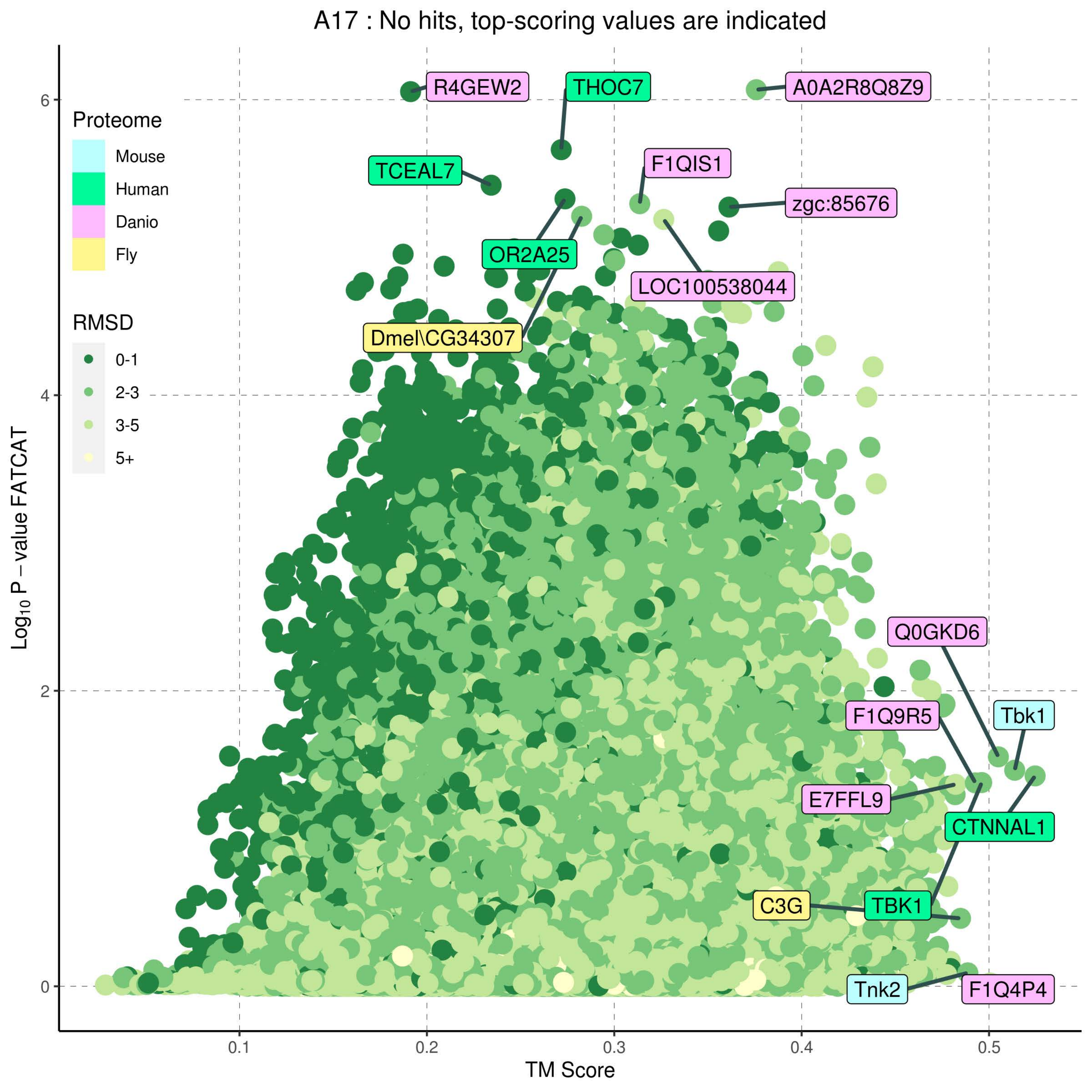

A17 : No hits, top-scoring values are indicated
Proteome
Mouse
Human
Danio
Fly
RMSD
0-1
2-3
3-5
5+
R4GEW2
THOC7
A0A2R8Q8Z9
TCEAL7
F1QIS1
zgc:85676
OR2A25
LOC100538044
Dmel\CG34307
Q0GKD6
F1Q9R5
Tbk1
E7FFL9
CTNNAL1
C3G
TBK1
Tnk2
F1Q4P4
6
4
2
0
0.1
0.2
0.3
0.4
0.5
Log10 P – value FATCAT
TM Score

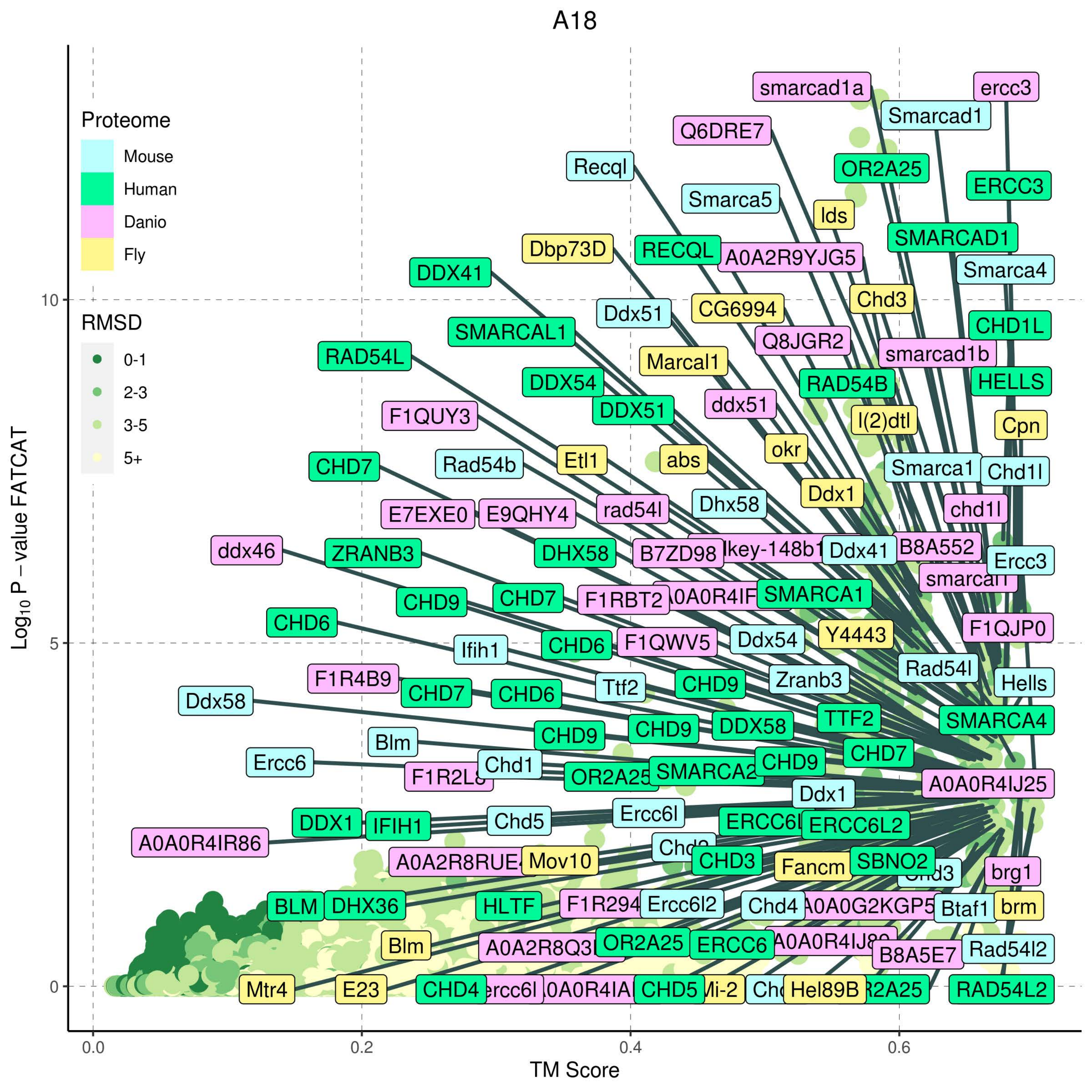
A18
Proteome
Mouse
Human
Danio
Fly
RMSD
0-1
2-3
3-5
5+
Log10 P – value FATCAT
TM Score
0.0
0.2
0.4
0.6
0
5
10
smarcad1a
ercc3
Smarcad1
Q6DRE7
Recql
OR2A25
ERCC3
Smarca5
lds
SMARCAD1
Dbp73D
RECQL
A0A2R9YJG5
Smarca4
DDX41
Chd3
Ddx51
CG6994
CHD1L
SMARCAL1
Q8JGR2
smarcad1b
RAD54L
Marcal1
DDX54
RAD54B
HELLS
DDX51
ddx51
F1QUY3
l(2)dtl
Cpn
CHD7
Rad54b
Etl1
abs
okr
Smarca1
Chd1l
Dhx58
Ddx1
E7EXE0
E9QHY4
rad54l
chd1l
ddx46
ZRANB3
DHX58
B7ZD98
Ddx41
B8A552
Ercc3
CHD9
CHD7
F1RBT2
SMARCA1
CHD6
Y4443
F1QJP0
Ifih1
CHD6
F1QWV5
Ddx54
Rad54l
F1R4B9
CHD7
CHD6
Ttf2
CHD9
Zranb3
Hells
Ddx58
TTF2
SMARCA4
Blm
CHD9
CHD9
DDX58
CHD7
Ercc6
Chd1
CHD9
A0A0R4IJ25
Ddx1
Ercc6l
DDX1
IFIH1
Chd5
ERCC6L2
A0A0R4IR86
CHD3
Fancm
SBNO2
brg1
BLM
DHX36
HLTF
Ercc6l2
Chd4
Btaf1
brm
Blm
OR2A25
ERCC6
B8A5E7
Rad54l2
Mtr4
E23
CHD4
CHD5
Hel89B
RAD54L2

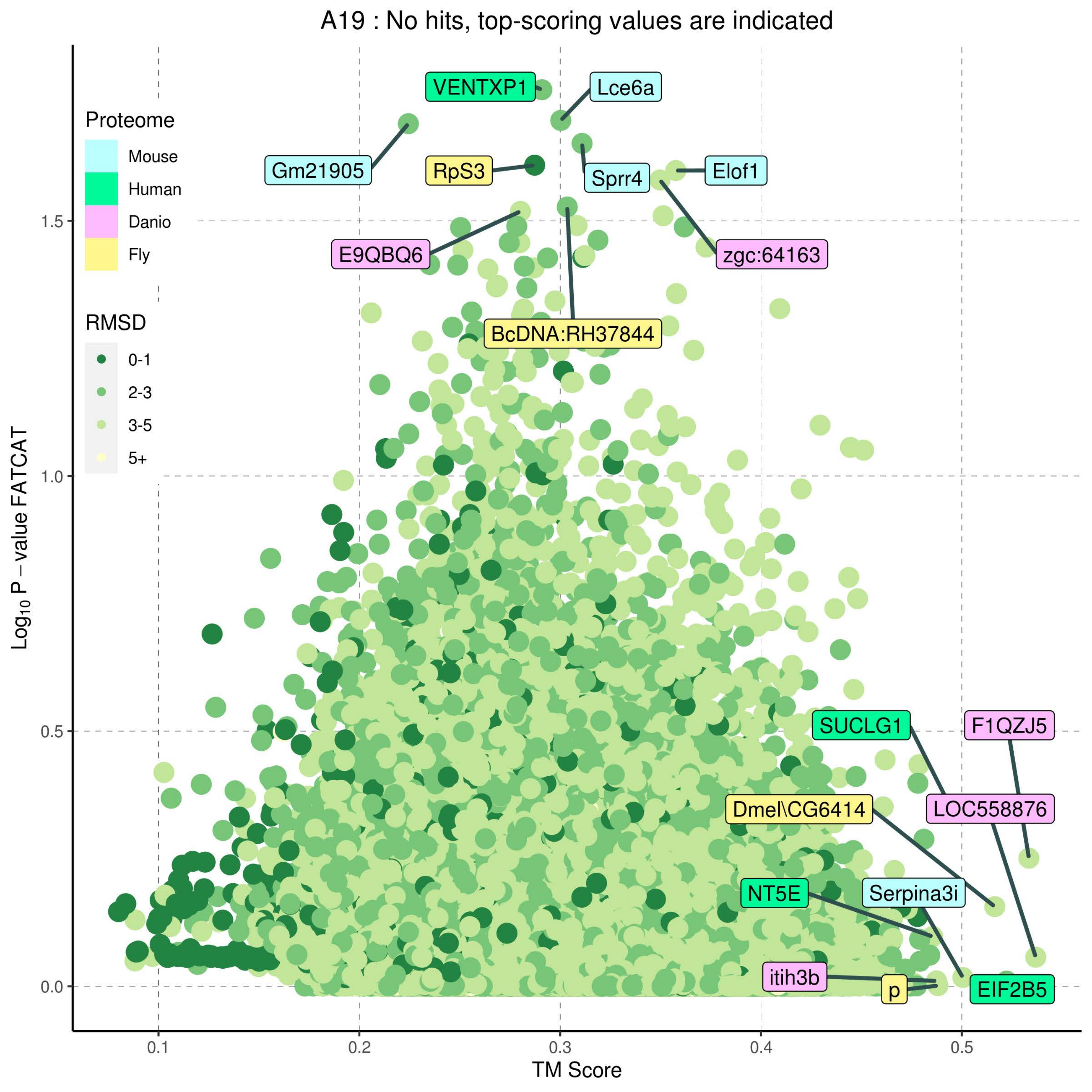

A19 : No hits, top-scoring values are indicated
Proteome
Mouse
Human
Danio
Fly
RMSD
0-1
2-3
3-5
5+
Log10 P − value FATCAT
TM Score
0.0
0.5
1.0
1.5
0.1
0.2
0.3
0.4
0.5
VENTXP1
Lce6a
Gm21905
RpS3
Sprr4
Elof1
E9QBQ6
zgc:64163
BcDNA:RH37844
SUCLG1
F1QZJ5
Dmel\CG6414
LOC558876
NT5E
Serpina3i
itih3b
p
EIF2B5

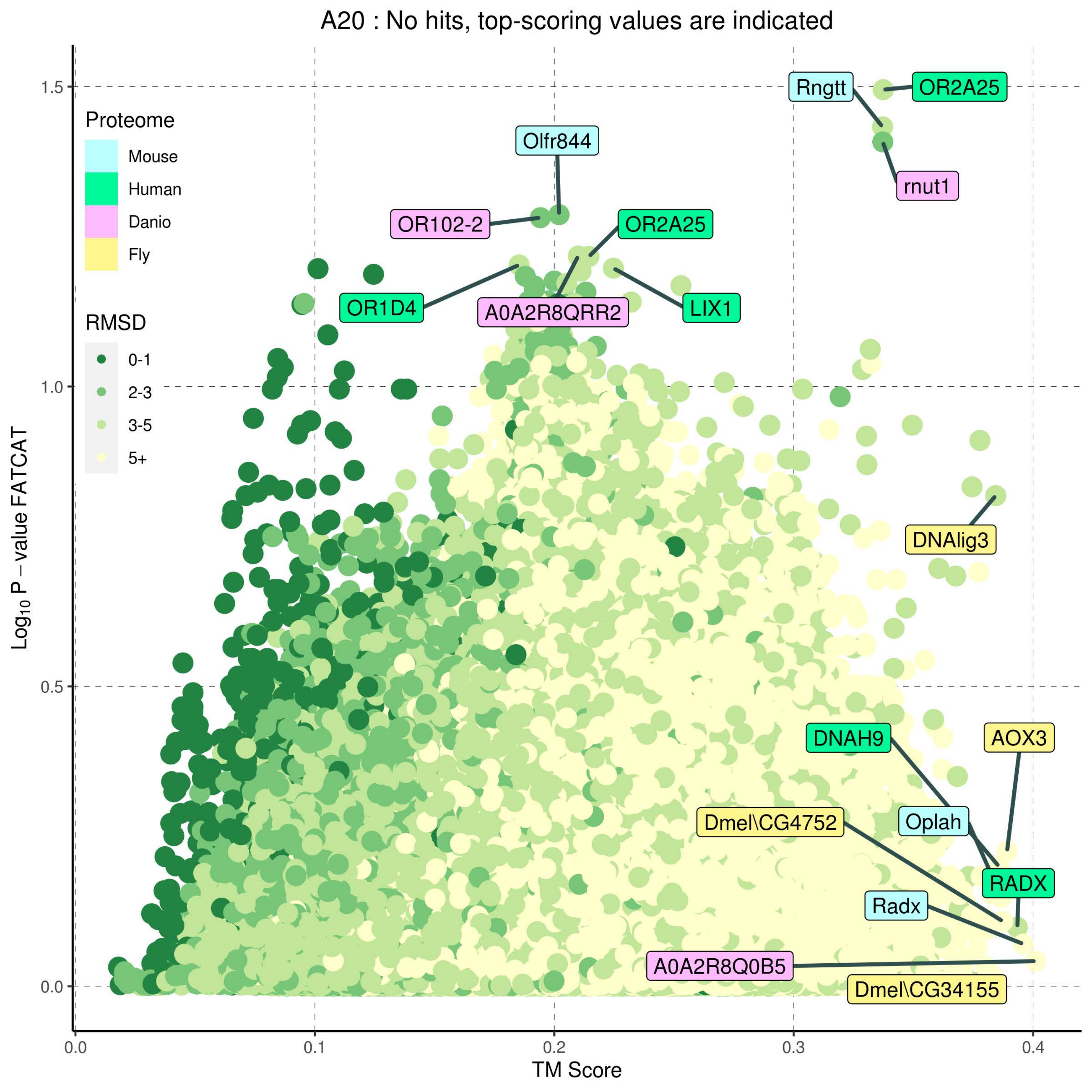
A20 : No hits, top-scoring values are indicated
Proteome
Mouse
Human
Danio
Fly
RMSD
0-1
2-3
3-5
5+
Log10 P – value FATCAT
TM Score
0.0
0.5
1.0
1.5
0.0
0.1
0.2
0.3
0.4
Rngtt
OR2A25
Olfr844
rnut1
OR102-2
OR2A25
OR1D4
A0A2R8QRR2
LIX1
DNAlig3
DNAH9
AOX3
Oplah
Dmel\CG4752
RADX
Radx
A0A2R8Q0B5
Dmel\CG34155

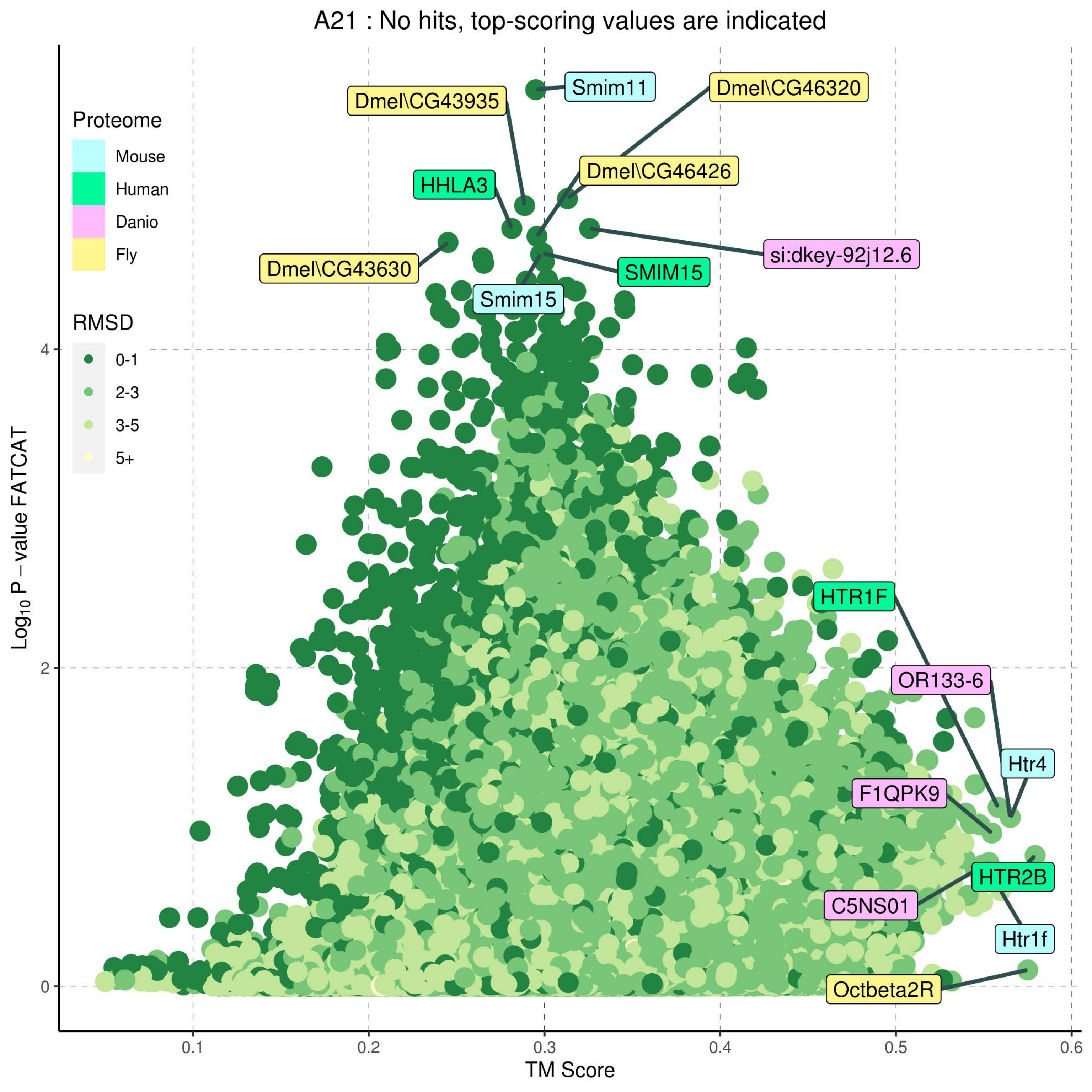

A21 : No hits, top-scoring values are indicated
Proteome
Mouse
Human
Danio
Fly
RMSD
0-1
2-3
3-5
5+
Smim11
Dmel\CG43935
Dmel\CG46320
Dmel\CG46426
HHLA3
si:dkey-92j12.6
Dmel\CG43630
SMIM15
Smim15
HTR1F
OR133-6
Htr4
F1QPK9
HTR2B
C5NS01
Htr1f
Octbeta2R
Log10 P – value FATCAT
TM Score
0
2
4
0.1
0.2
0.3
0.4
0.5
0.6

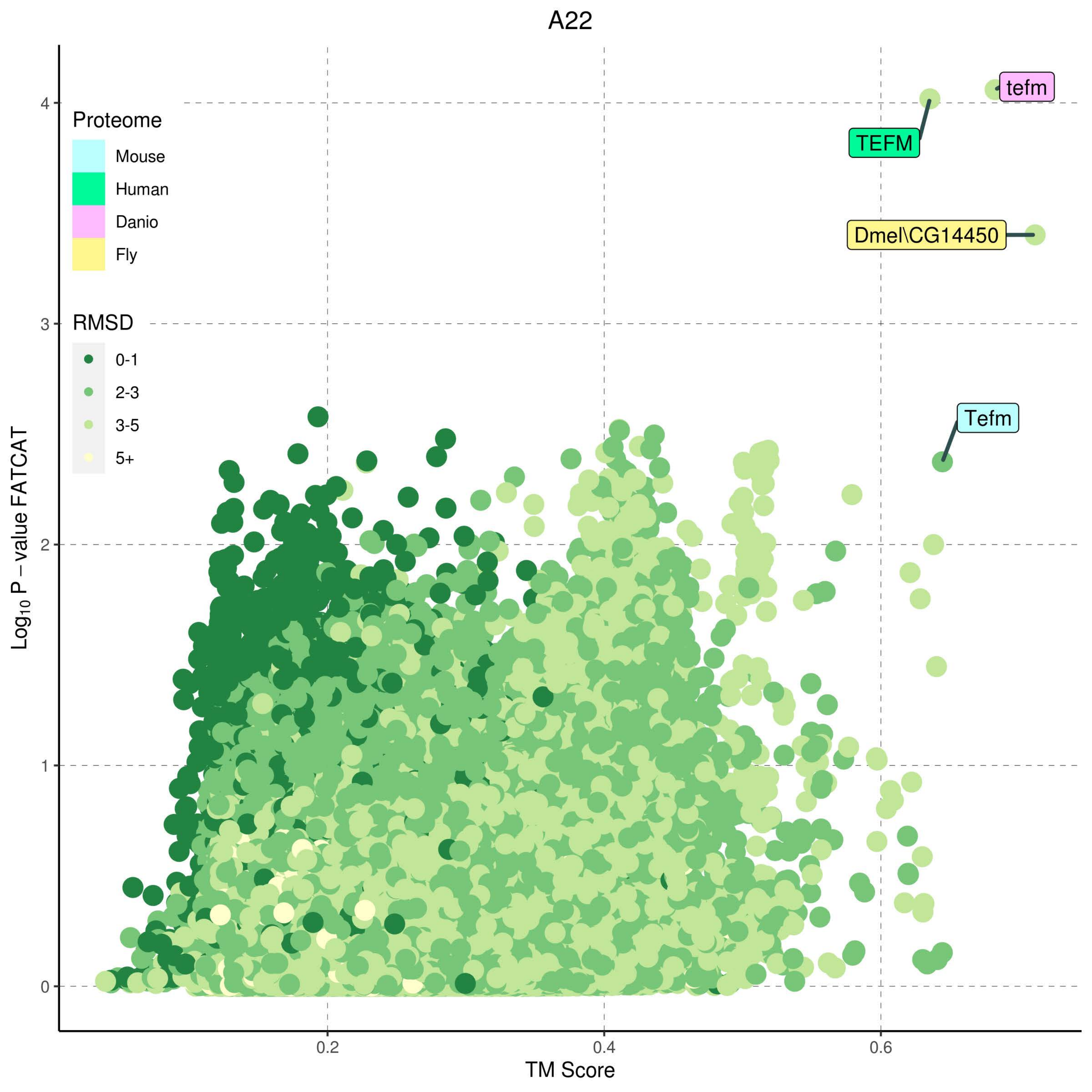

A22
Proteome
Mouse
Human
Danio
Fly
RMSD
0-1
2-3
3-5
5+
tefm
TEFM
Dmel\CG14450
Tefm
Log_{10} P − value FATCAT
TM Score
0
1
2
3
4
0.2
0.4
0.6

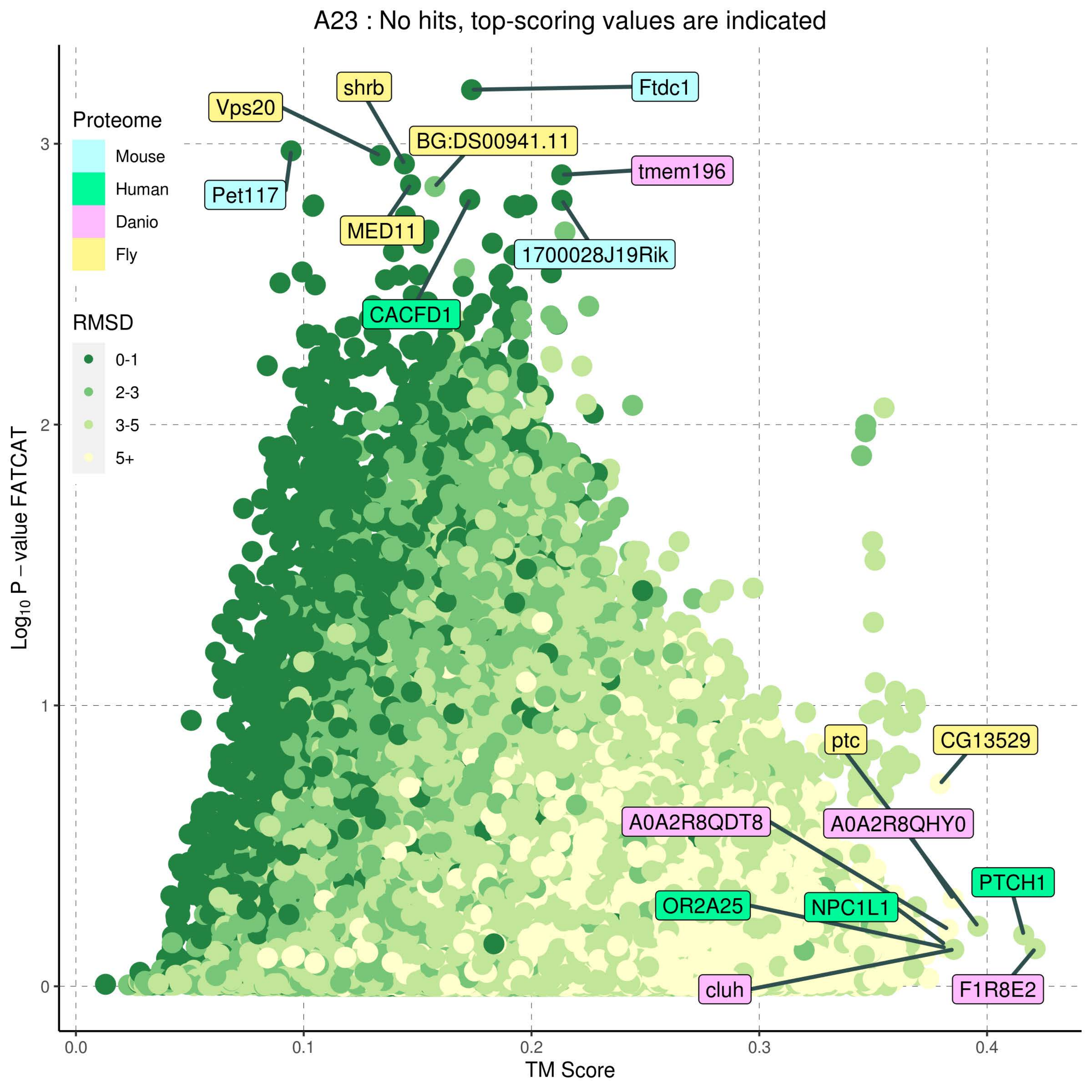
A23 : No hits, top-scoring values are indicated
Proteome
Mouse
Human
Danio
Fly
RMSD
0-1
2-3
3-5
5+
Ftdc1
shrb
Vps20
BG:DS00941.11
tmem196
Pet117
MED11
1700028J19Rik
CACFD1
ptc
CG13529
A0A2R8QDT8
A0A2R8QHY0
PTCH1
OR2A25
NPC1L1
cluh
F1R8E2
Log10 P – value FATCAT
TM Score
0.0
0.1
0.2
0.3
0.4
0
1
2
3

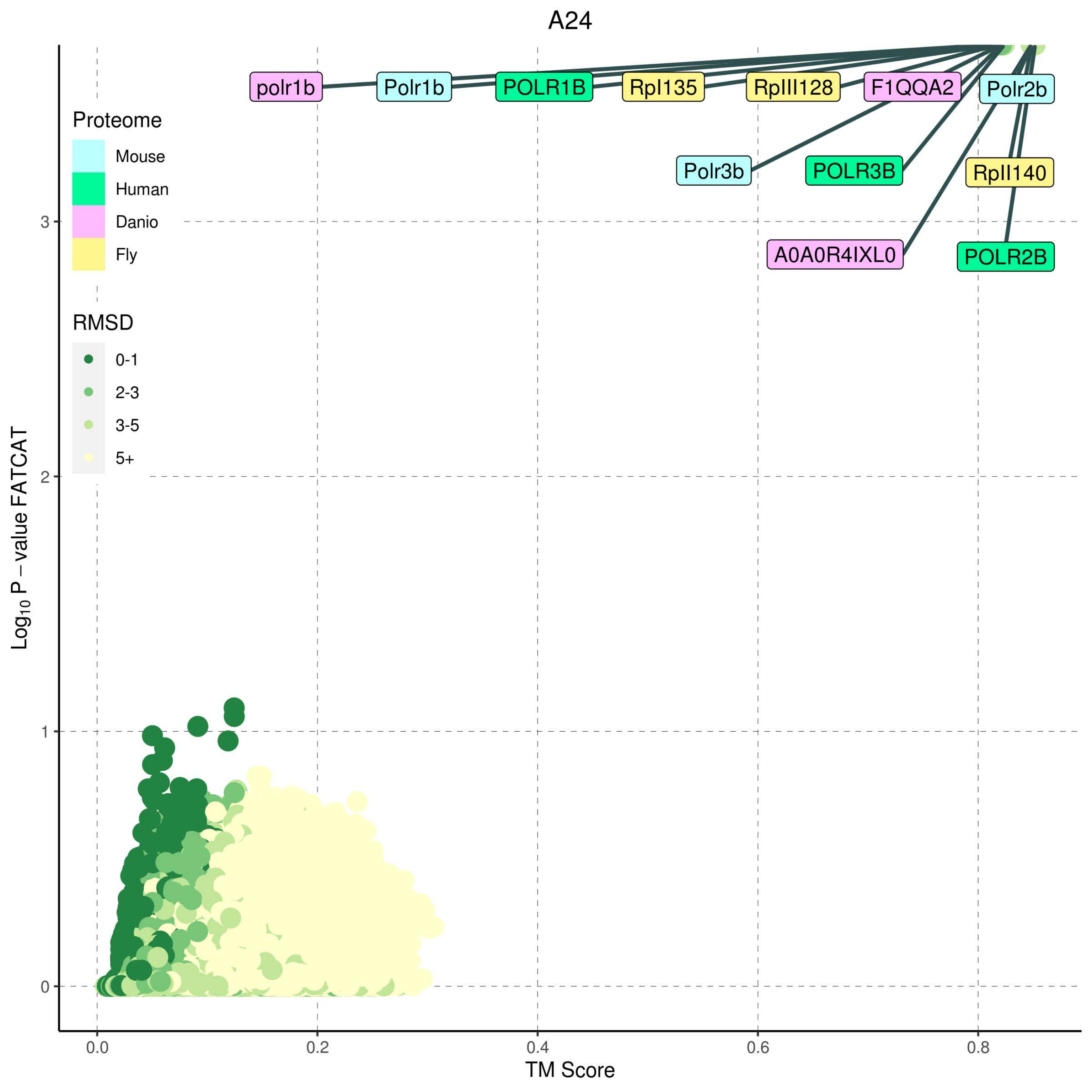

A24
polr1b
Polr1b
POLR1B
Rpl135
RpIII128
F1QQA2
Polr2b
Polr3b
POLR3B
RpII140
A0A0R4IXL0
POLR2B
Proteome
Mouse
Human
Danio
Fly
RMSD
0-1
2-3
3-5
5+
Log_{10} P − value FATCAT
TM Score
0.0
0.2
0.4
0.6
0.8
0
1
2
3

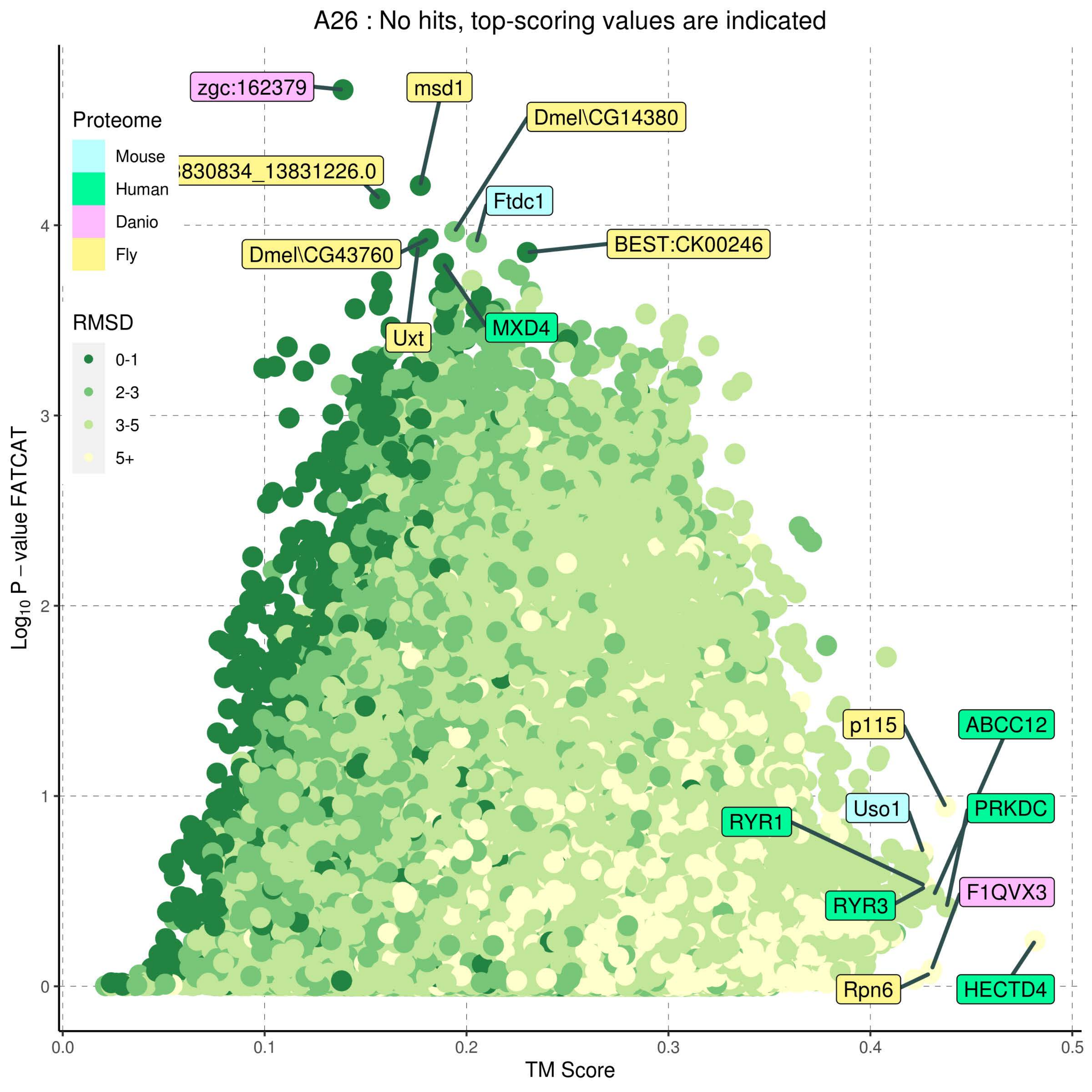
A26 : No hits, top-scoring values are indicated
Proteome
Mouse
Human
Danio
Fly
RMSD
0-1
2-3
3-5
5+
zgc:162379
msd1
Dmel\CG14380
830834_13831226.0
Ftdc1
BEST:CK00246
Dmel\CG43760
MXD4
Uxt
p115
ABCC12
Uso1
PRKDC
RYR1
RYR3
F1QVX3
Rpn6
HECTD4
Log10 P − value FATCAT
TM Score
0
1
2
3
4
0.0
0.1
0.2
0.3
0.4
0.5

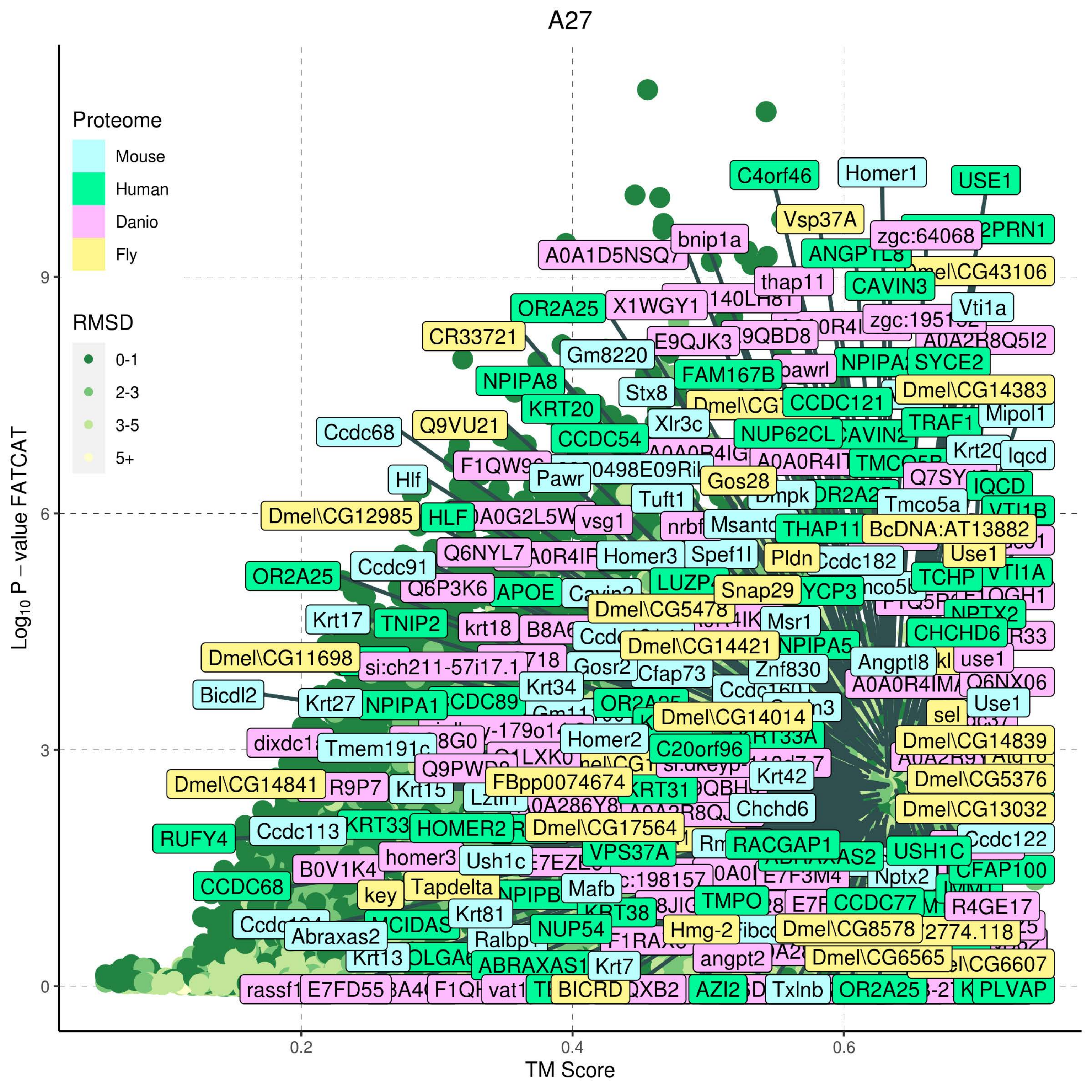
A27
Proteome
Mouse
Human
Danio
Fly
RMSD
0-1
2-3
3-5
5+
Log10 P – value FATCAT
TM Score
0
3
6
9
0.2
0.4
0.6
C4orf46
Homer1
USE1
Vsp37A
bnip1a
zgc:64068
A0A1D5NSQ7
thap11
CAVIN3
OR2A25
X1WGY1
Vti1a
CR33721
E9QJK3
A0A2R8Q5I2
Gm8220
SYCE2
NPIPA8
FAM167B
Dmel\CG14383
Stx8
CCDC121
KRT20
TRAF1
Mipol1
Ccdc68
Q9VU21
Xlr3c
NUP62CL
CCDC54
Iqcd
Hlf
Pawr
Gos28
IQCD
Tmco5a
Tuft1
Dmel\CG12985
HLF
vsg1
THAP11
BcDNA:AT13882
Q6NYL7
Homer3
Spef1l
Pldn
Ccdc182
Use1
OR2A25
Ccdc91
TCHP
VTI1A
Q6P3K6
APOE
Snap29
Dmel\CG5478
Krt17
TNIP2
krt18
Msr1
CHCHD6
Dmel\CG11698
Dmel\CG14421
si:ch211-57i17.1
Gosr2
Cfap73
Znf830
Angptl8
use1
Q6NX06
Bicdl2
Krt27
NPIPA1
Krt34
Dmel\CG14014
Use1
sel
Homer2
C20orf96
Dmel\CG14839
Q9PWD8
FBpp0074674
Krt42
Dmel\CG5376
Dmel\CG14841
R9P7
Krt15
Chchd6
Dmel\CG13032
RUFY4
Ccdc113
KRT33
HOMER2
Dmel\CG17564
RACGAP1
Ccdc122
homer3
Ush1c
VPS37A
USH1C
B0V1K4
Nptx2
CFAP100
CCDC68
key
Tapdelta
Mafb
TMPO
CCDC77
R4GE17
Krt81
Abraxas2
NUP54
Hmg-2
Dmel\CG8578
Krt13
ABRAXAS1
Krt7
angpt2
Dmel\CG6565
rassf1
E7FD55
BICRD
AZI2
Txlnb
OR2A25
PLVAP

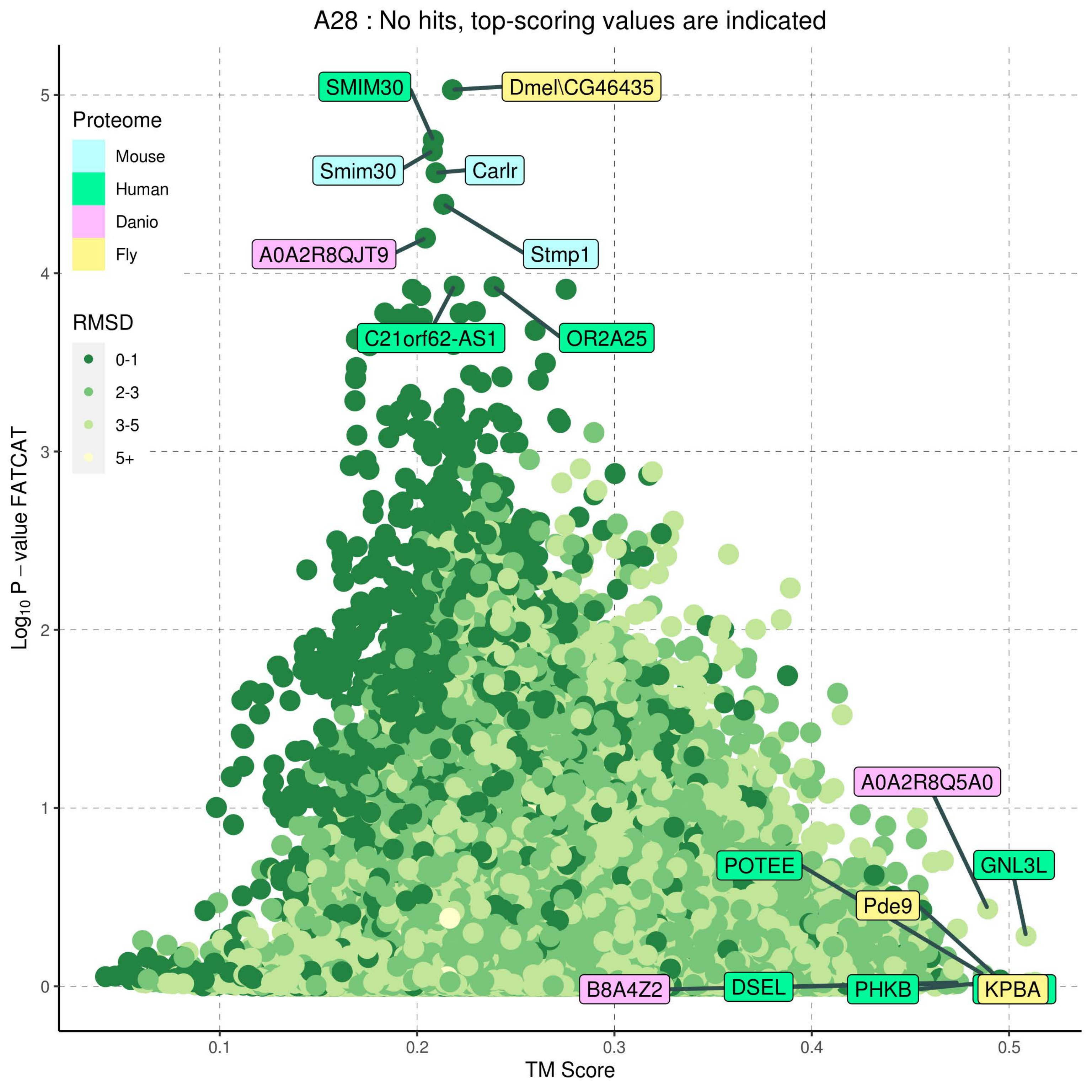
A28 : No hits, top-scoring values are indicated
Proteome
Mouse
Human
Danio
Fly
RMSD
0-1
2-3
3-5
5+
SMIM30
Dmel\CG46435
Smim30
Carlr
A0A2R8QJT9
Stmp1
C21orf62-AS1
OR2A25
A0A2R8Q5A0
POTEE
GNL3L
Pde9
B8A4Z2
DSEL
PHKB
KPBA
Log10 P – value FATCAT
TM Score
0
1
2
3
4
5
0.1
0.2
0.3
0.4
0.5

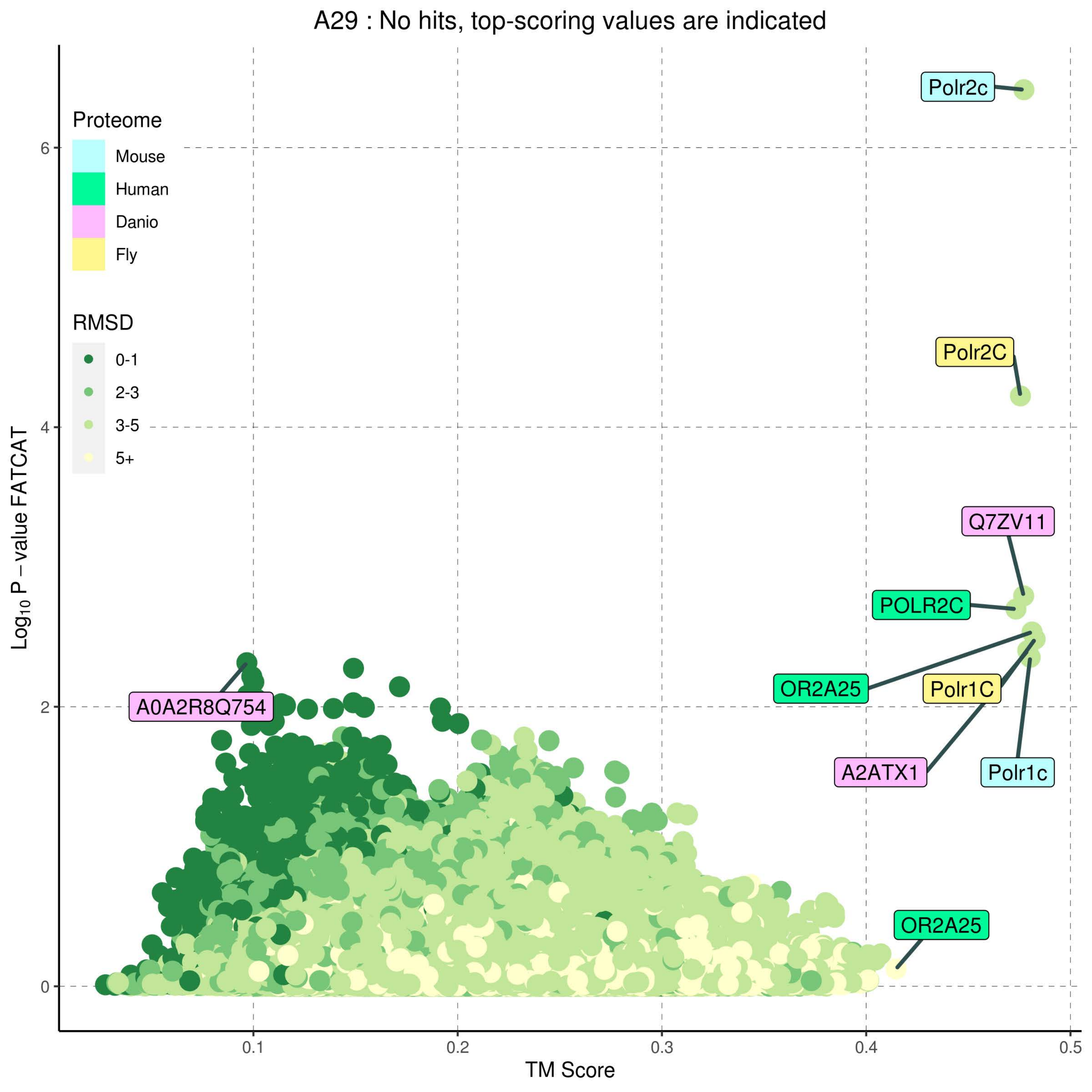

A29 : No hits, top-scoring values are indicated
Proteome
Mouse
Human
Danio
Fly
RMSD
0-1
2-3
3-5
5+
Polr2c
Polr2C
Q7ZV11
POLR2C
OR2A25
Polr1C
A2ATX1
Polr1c
A0A2R8Q754
OR2A25
0
2
4
6
0.1
0.2
0.3
0.4
0.5
TM Score
Log10 P − value FATCAT

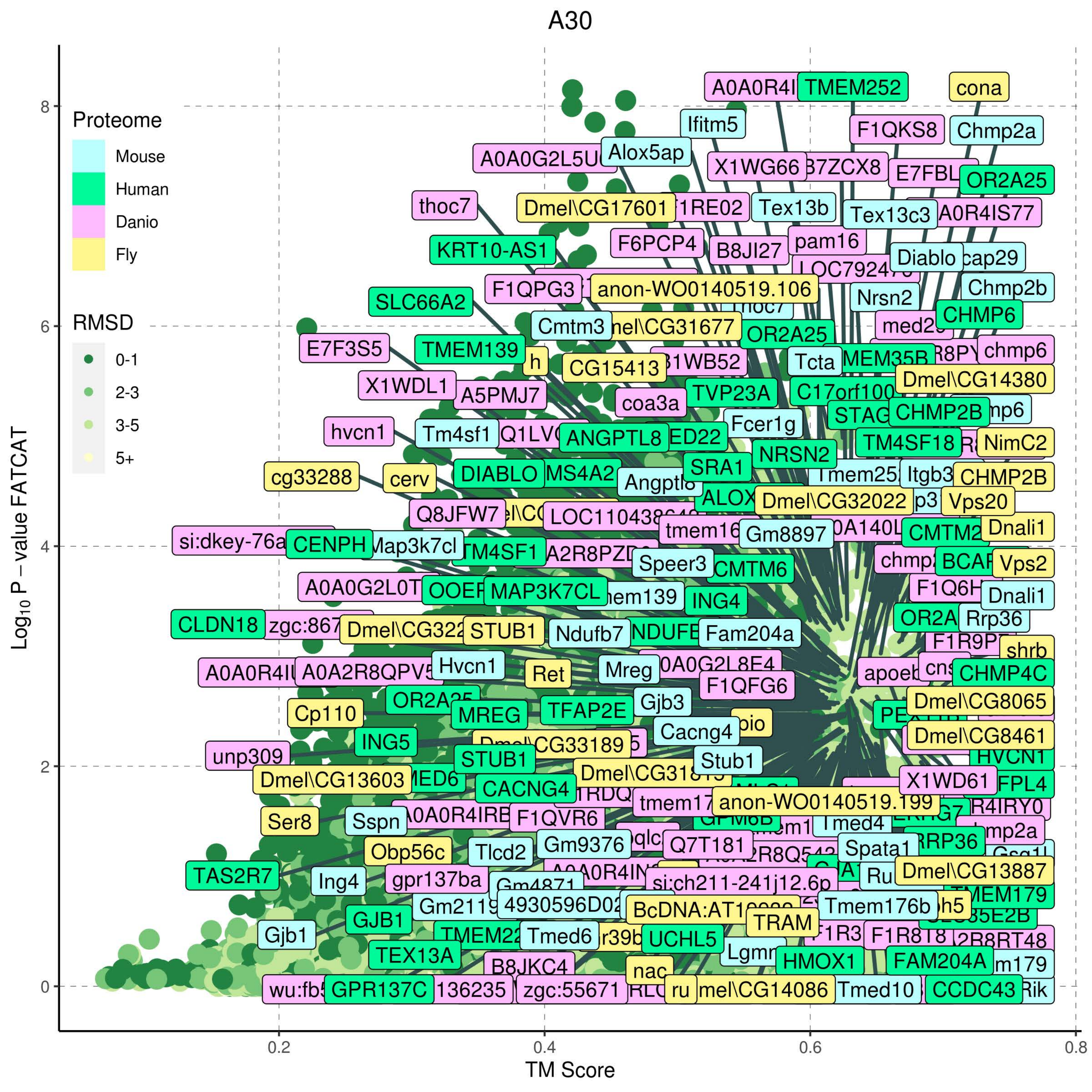

A30
Proteome
Mouse
Human
Danio
Fly
RMSD
0-1
2-3
3-5
5+
Log10 P − value FATCAT
TM Score
0
2
4
6
8
0.2
0.4
0.6
0.8
TMEM252
cona
Ifitm5
F1QKS8
Chmp2a
Alox5ap
X1WG66
OR2A25
thoc7
Dmel\CG17601
Tex13b
Tex13c3
KRT10-AS1
F6PCP4
B8JI27
pam16
Diablo
F1QPG3
anon-WO0140519.106
Chmp2b
SLC66A2
Nrsn2
CHMP6
Cmtm3
E7F3S5
TMEM139
CG15413
Tcta
chmp6
Dmel\CG14380
X1WDL1
A5PMJ7
coa3a
TVP23A
hvcn1
Tm4sf1
ANGPTL8
Fcer1g
CHMP2B
TM4SF18
NimC2
NRSN2
SRA1
cg33288
cerv
DIABLO
MS4A2
Itgb3
Dmel\CG32022
Vps20
Q8JFW7
Gm8897
Dnali1
CENPH
TM4SF1
Speer3
CMTM6
MAP3K7CL
ING4
Rrp36
CLDN18
STUB1
Ndufb7
Fam204a
shrb
Hvcn1
Ret
Mreg
F1QFG6
CHMP4C
Dmel\CG8065
Cp110
MREG
TFAP2E
Gjb3
Cacng4
Dmel\CG8461
ING5
unp309
Stub1
HVCN1
Dmel\CG13603
CACNG4
X1WD61
anon-WO0140519.199
Ser8
Sspn
F1QVR6
Tmed4
Obp56c
Tlcd2
Gm9376
Q7T181
Spata1
RRP36
TAS2R7
Ing4
gpr137ba
Dmel\CG13887
si:ch211-241j12.6p
TMEM179
4930596D02
Tmem176b
GJB1
TRAM
Gjb1
Tmed6
UCHL5
F1R8T8
TEX13A
HMOX1
FAM204A
B8JKC4
nac
GPR137C
zgc:55671
ru
Tmed10
CCDC43

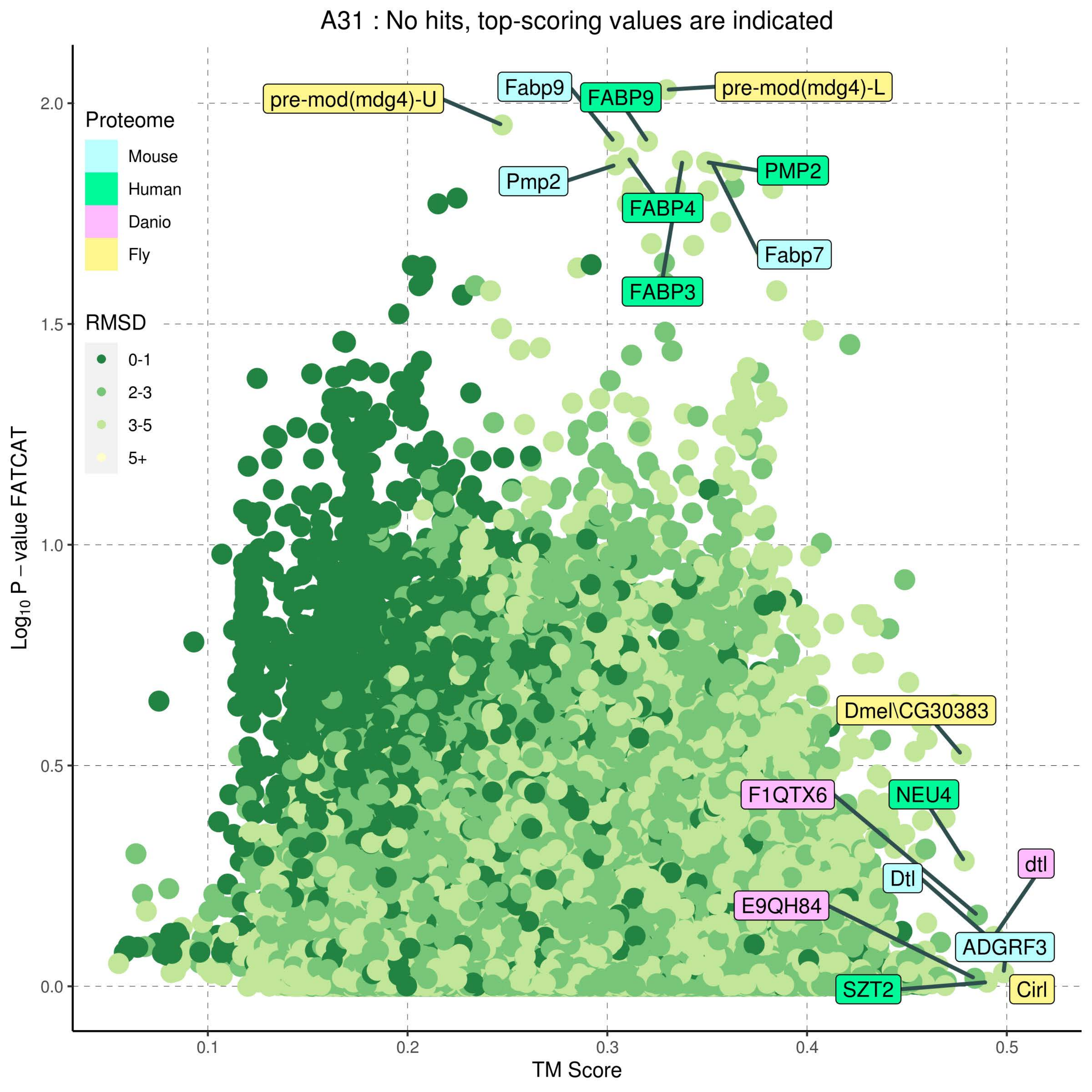

A31 : No hits, top-scoring values are indicated
Proteome
Mouse
Human
Danio
Fly
RMSD
0-1
2-3
3-5
5+
Log10 P – value FATCAT
TM Score
0.0
0.5
1.0
1.5
2.0
0.1
0.2
0.3
0.4
0.5
pre-mod(mdg4)-U
Fabp9
FABP9
pre-mod(mdg4)-L
Pmp2
PMP2
FABP4
Fabp7
FABP3
Dmel\CG30383
F1QTX6
NEU4
dtl
Dtl
E9QH84
ADGRF3
SZT2
Cirl

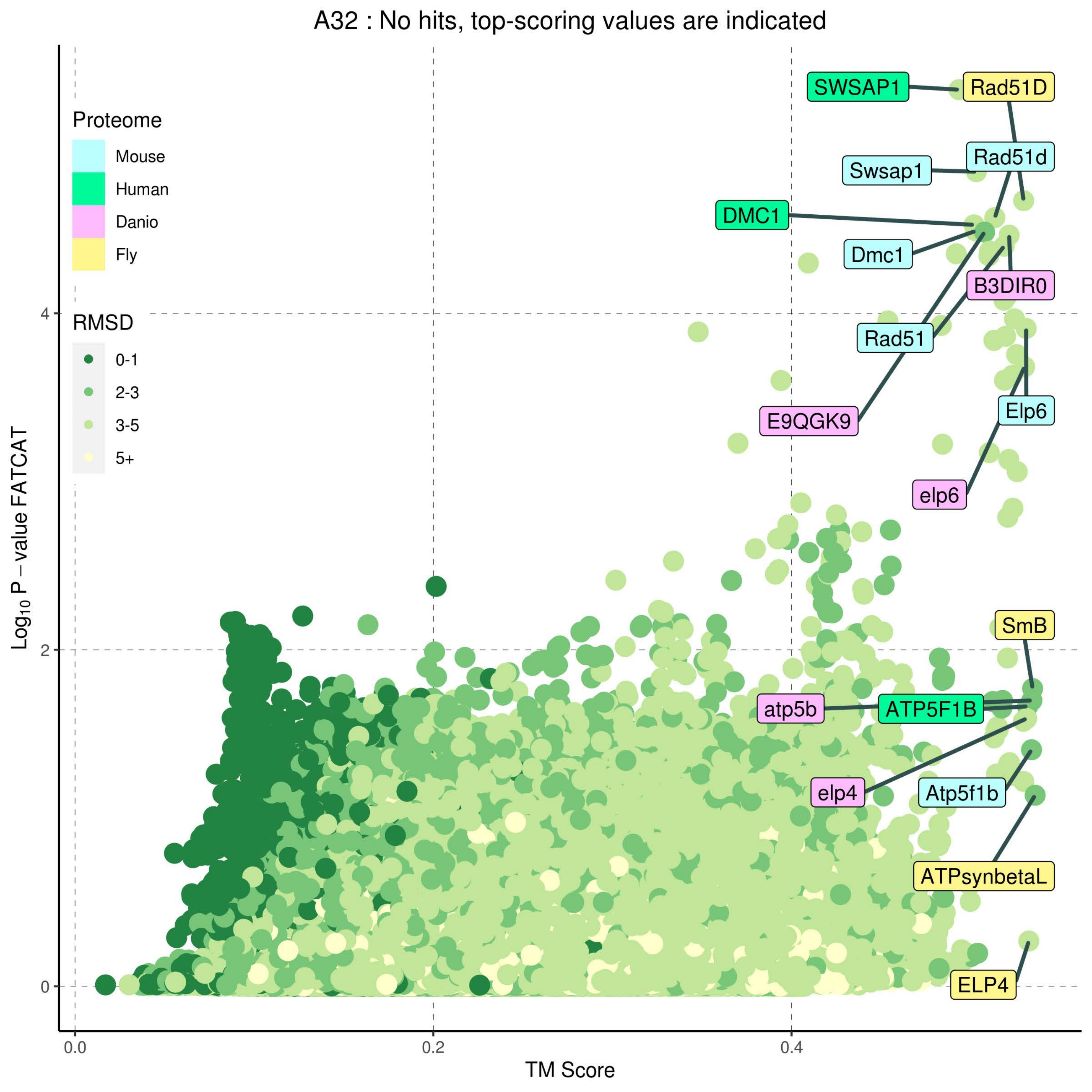

A32 : No hits, top-scoring values are indicated
Proteome
Mouse
Human
Danio
Fly
RMSD
0-1
2-3
3-5
5+
Log10 P − value FATCAT
TM Score
0.0
0.2
0.4
0
2
4
SWSAP1
Rad51D
Rad51d
Swsap1
DMC1
Dmc1
B3DIR0
Rad51
E9QGK9
Elp6
elp6
SmB
atp5b
ATP5F1B
elp4
Atp5f1b
ATPsynbetaL
ELP4

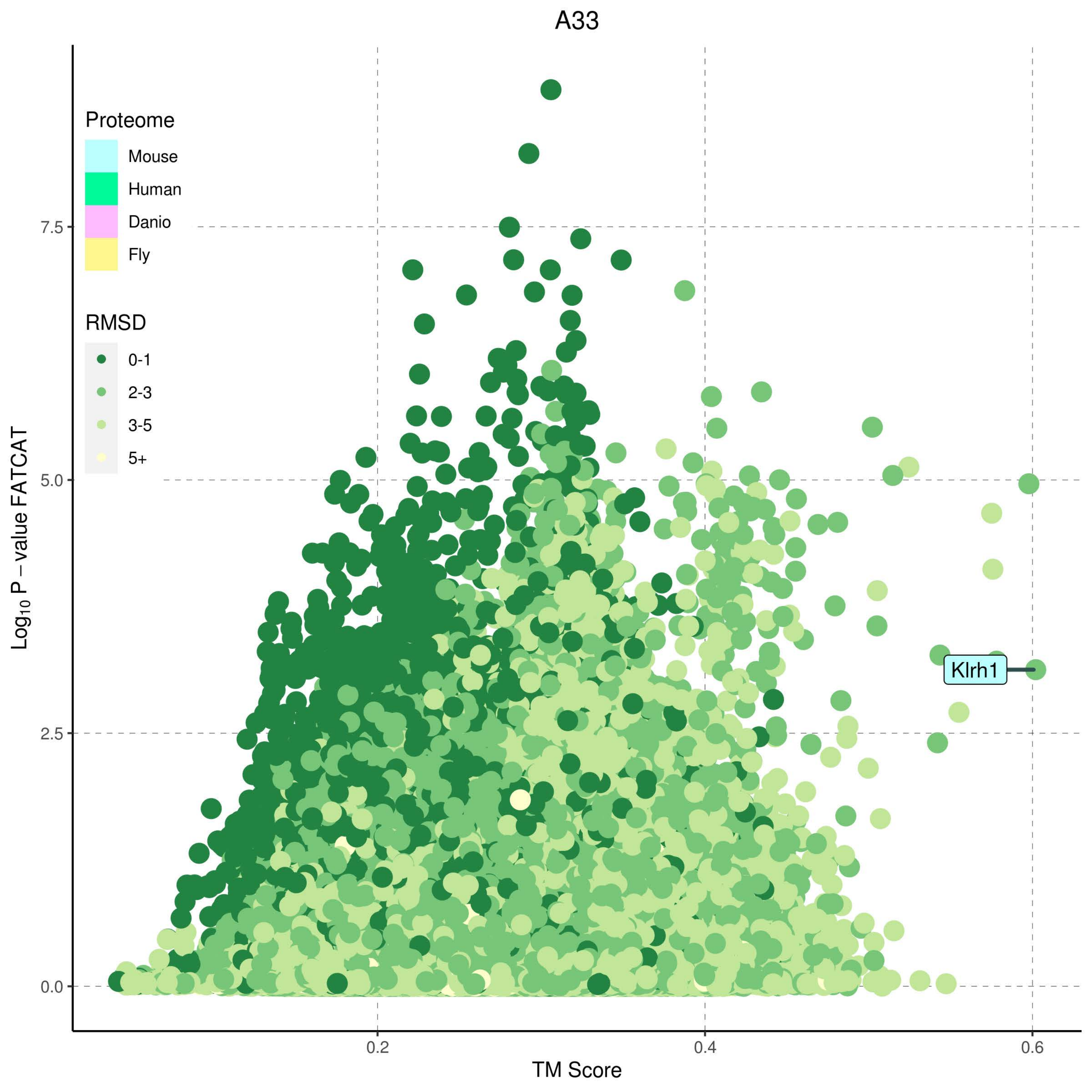

A33
Proteome
Mouse
Human
Danio
Fly
RMSD
0-1
2-3
3-5
5+
7.5
5.0
2.5
0.0
Log10 P − value FATCAT
0.2
0.4
0.6
TM Score
Klrh1

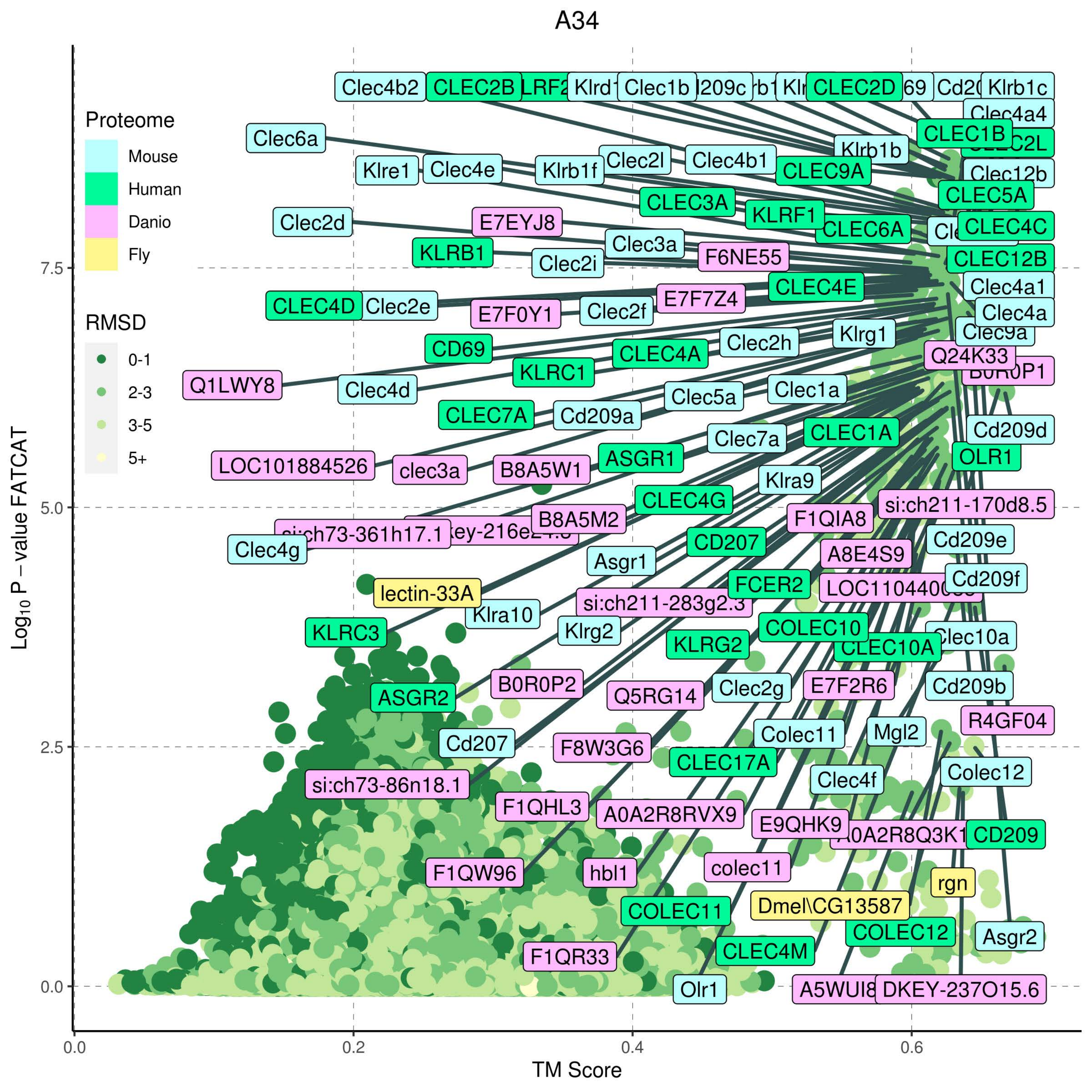

A34
Proteome
Mouse
Human
Danio
Fly
RMSD
0-1
2-3
3-5
5+
Log10 P – value FATCAT
TM Score
0.0
0.2
0.4
0.6
0.0
2.5
5.0
7.5
Clec4b2
CLEC2B
Clec6a
Klre1
Clec4e
Klrb1f
Clec2l
Clec4b1
CLEC9A
Klrb1b
CLEC1B
Clec4a4
Klrb1c
CLEC2D
Clec1b
Clec12b
CLEC5A
CLEC3A
KLRF1
CLEC6A
CLEC4C
Clec2d
E7EYJ8
Clec3a
KLRB1
Clec2i
F6NE55
CLEC12B
CLEC4E
Clec4a1
CLEC4D
Clec2e
E7F0Y1
Clec2f
E7F7Z4
Clec4a
Klrg1
Clec2h
CD69
CLEC4A
KLRC1
Q24K33
Q1LWY8
Clec4d
Clec1a
Clec5a
CLEC7A
Cd209a
Clec7a
CLEC1A
Cd209d
LOC101884526
clec3a
B8A5W1
ASGR1
OLR1
Klra9
CLEC4G
si:ch211-170d8.5
B8A5M2
F1QIA8
CD207
Cd209e
Clec4g
Asgr1
A8E4S9
FCER2
Cd209f
lectin-33A
Klra10
KLRC3
Klrg2
COLEC10
KLRG2
Clec10a
CLEC10A
B0R0P2
ASGR2
Q5RG14
Clec2g
E7F2R6
Cd209b
Mgl2
R4GF04
Colec11
Cd207
F8W3G6
CLEC17A
Colec12
si:ch73-86n18.1
Clec4f
F1QHL3
A0A2R8RVX9
E9QHK9
CD209
F1QW96
hbl1
colec11
rgn
Dmel\CG13587
COLEC11
COLEC12
Asgr2
F1QR33
CLEC4M
Olr1
DKEY-237O15.6

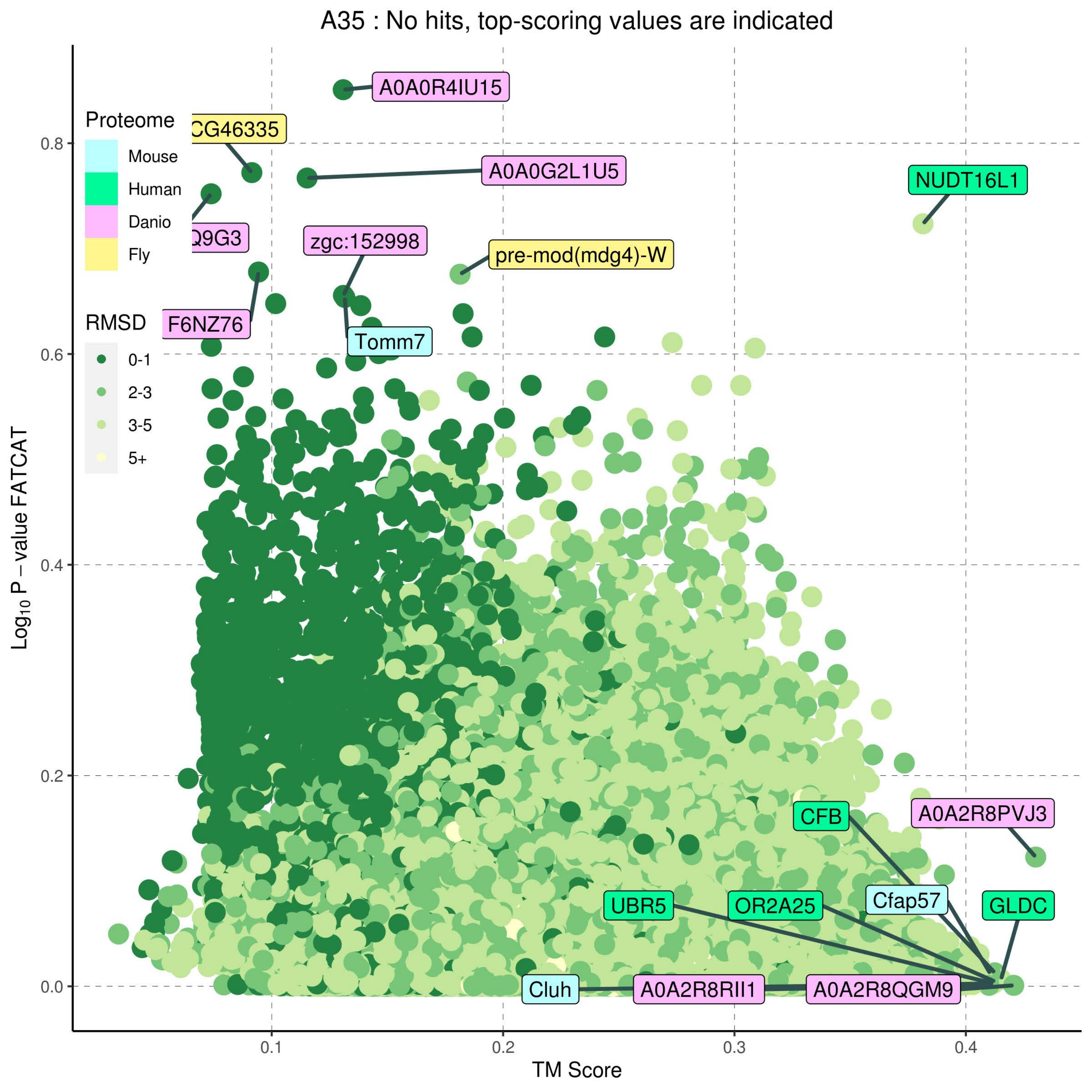

A35 : No hits, top-scoring values are indicated
Proteome
Mouse
Human
Danio
Fly
RMSD
0-1
2-3
3-5
5+
A0A0R4IU15
CG46335
A0A0G2L1U5
NUDT16L1
Q9G3
zgc:152998
pre-mod(mdg4)-W
F6NZ76
Tomm7
CFB
A0A2R8PVJ3
UBR5
OR2A25
Cfap57
GLDC
Cluh
A0A2R8RII1
A0A2R8QGM9
0.0
0.2
0.4
0.6
0.8
0.1
0.2
0.3
0.4
TM Score
Log10 P − value FATCAT

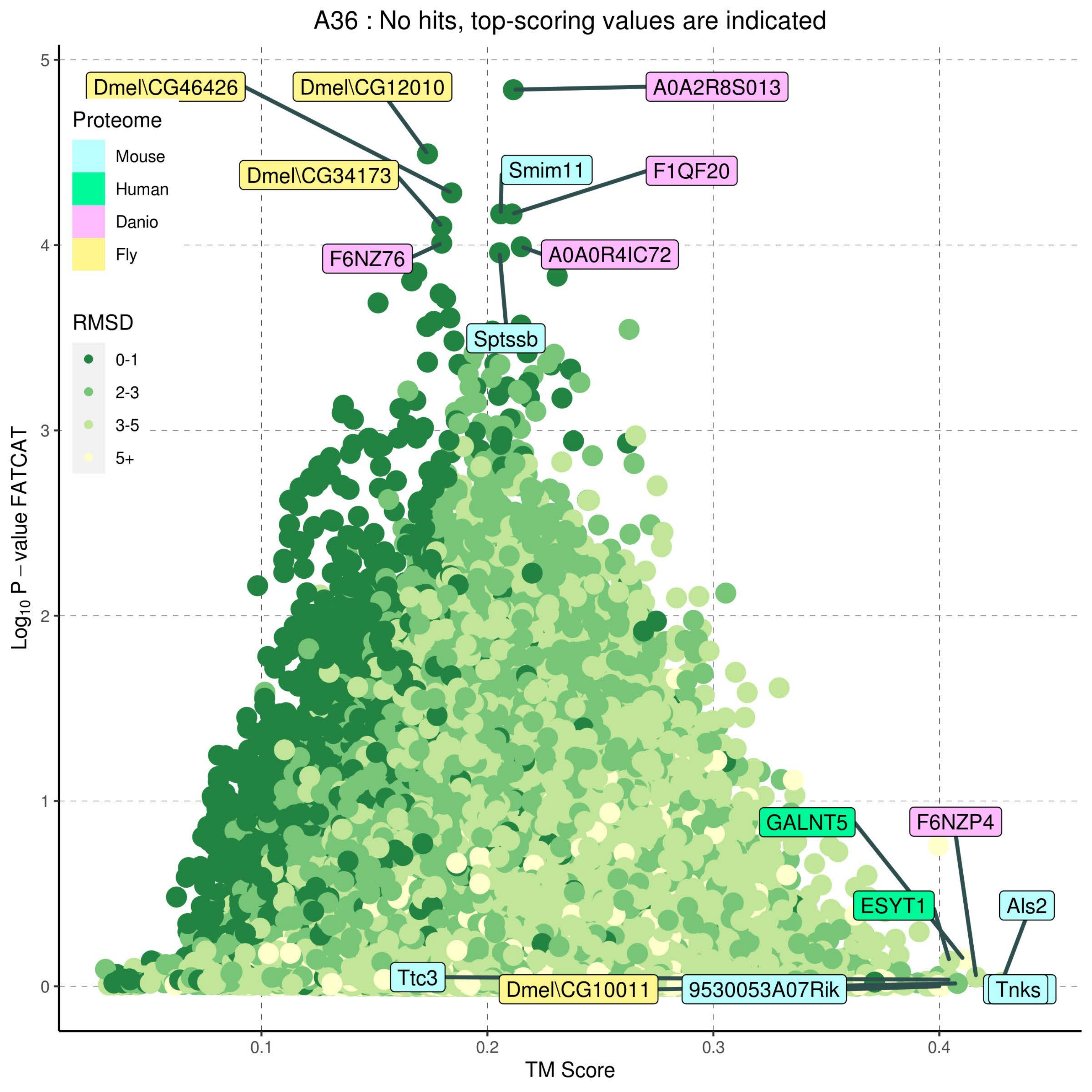

A36 : No hits, top-scoring values are indicated
Proteome
Mouse
Human
Danio
Fly
RMSD
0-1
2-3
3-5
5+
Dmel\CG46426
Dmel\CG12010
A0A2R8S013
Dmel\CG34173
Smim11
F1QF20
F6NZ76
A0A0R4IC72
Sptssb
GALNT5
F6NZP4
ESYT1
Als2
Ttc3
Dmel\CG10011
9530053A07Rik
Tnks
Log10 P – value FATCAT
TM Score
0.1
0.2
0.3
0.4
0
1
2
3
4
5

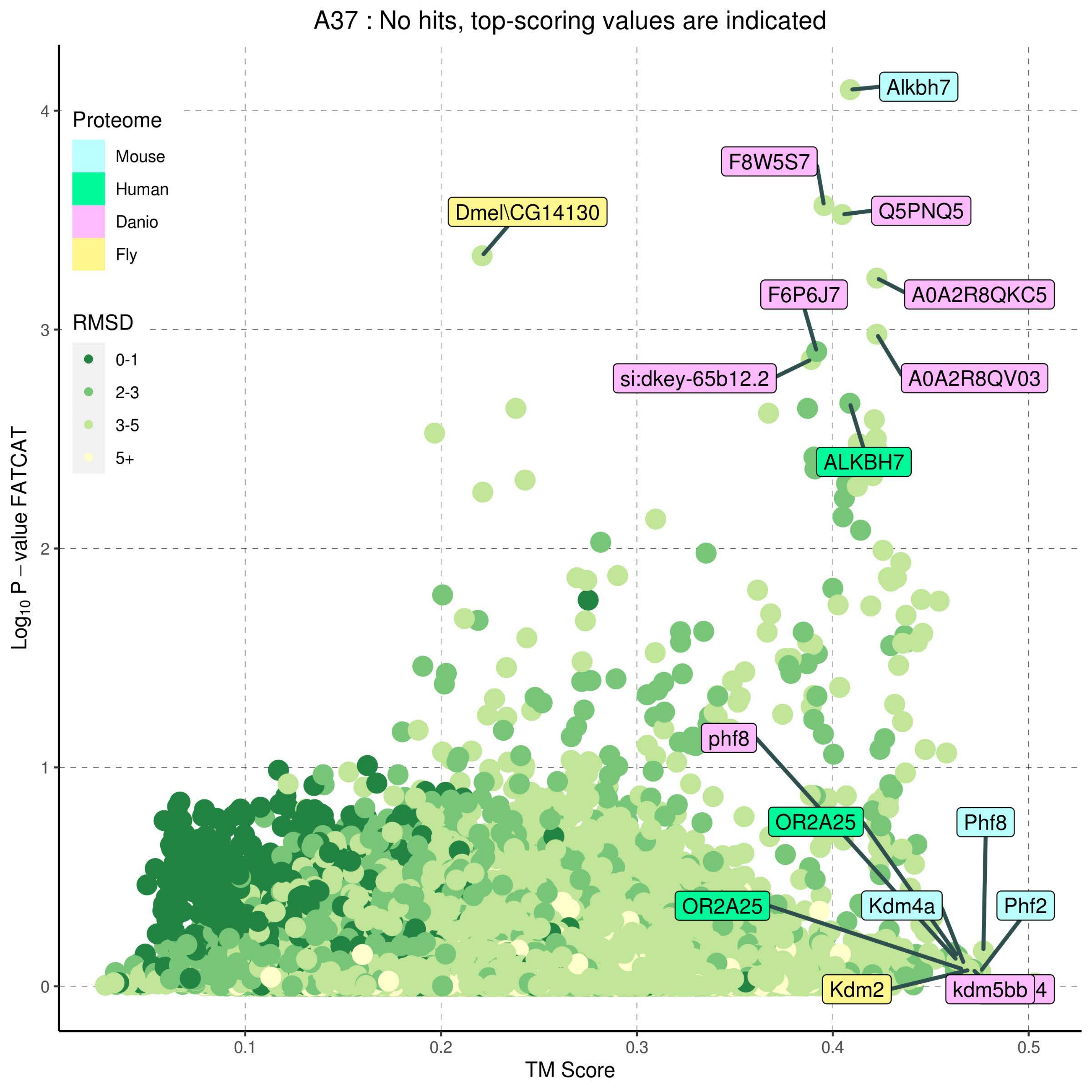
A37 : No hits, top-scoring values are indicated
Proteome
Mouse
Human
Danio
Fly
RMSD
0-1
2-3
3-5
5+
Alkbh7
F8W5S7
Q5PNQ5
Dmel\CG14130
F6P6J7
A0A2R8QKC5
si:dkey-65b12.2
A0A2R8QV03
ALKBH7
phf8
OR2A25
Phf8
OR2A25
Kdm4a
Phf2
Kdm2
kdm5bb
4
Log10 P − value FATCAT
TM Score
0
1
2
3
4
0.1
0.2
0.3
0.4
0.5

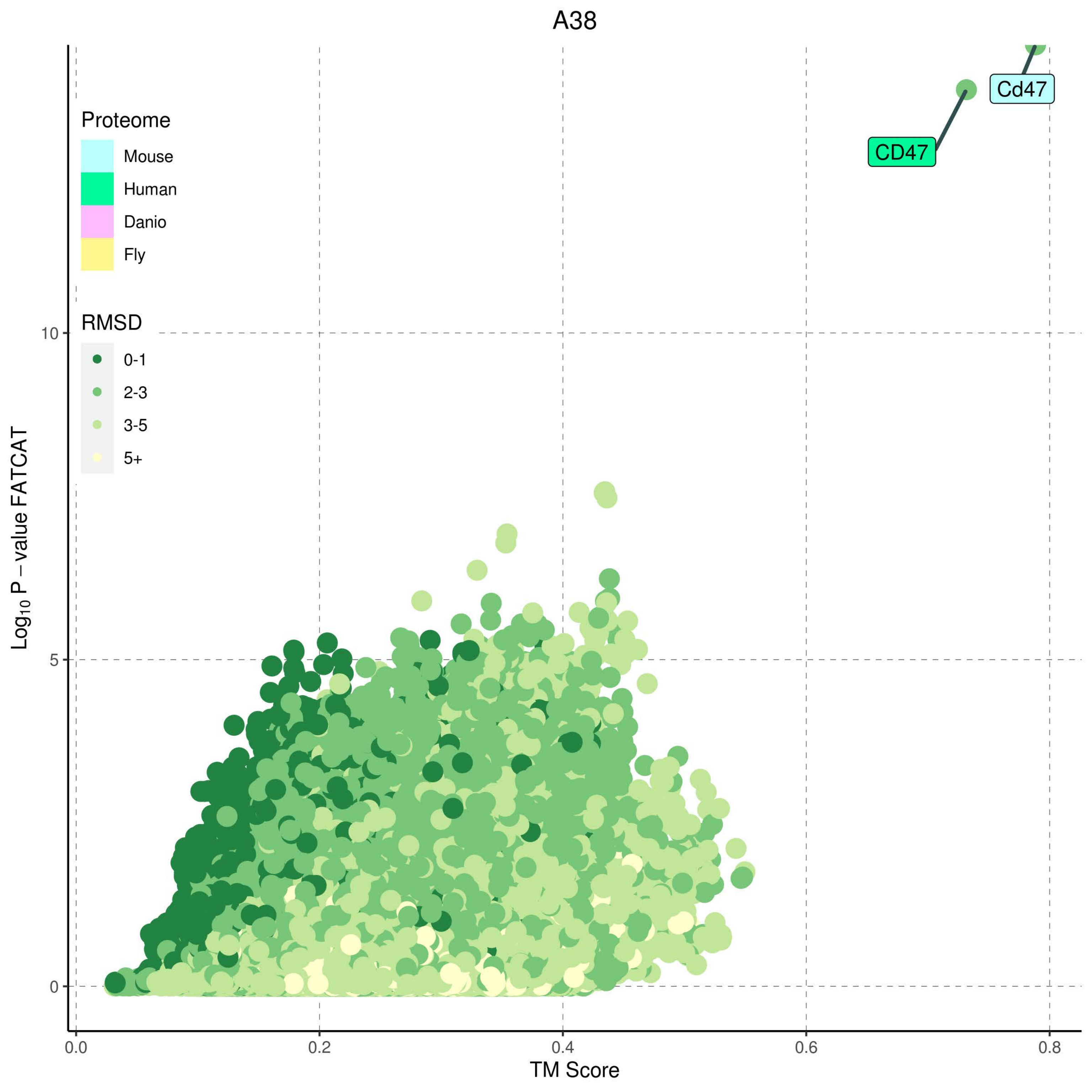

A38
Proteome
Mouse
Human
Danio
Fly
RMSD
0-1
2-3
3-5
5+
CD47
Cd47
Log10 P − value FATCAT
TM Score
0
5
10
0.0
0.2
0.4
0.6
0.8

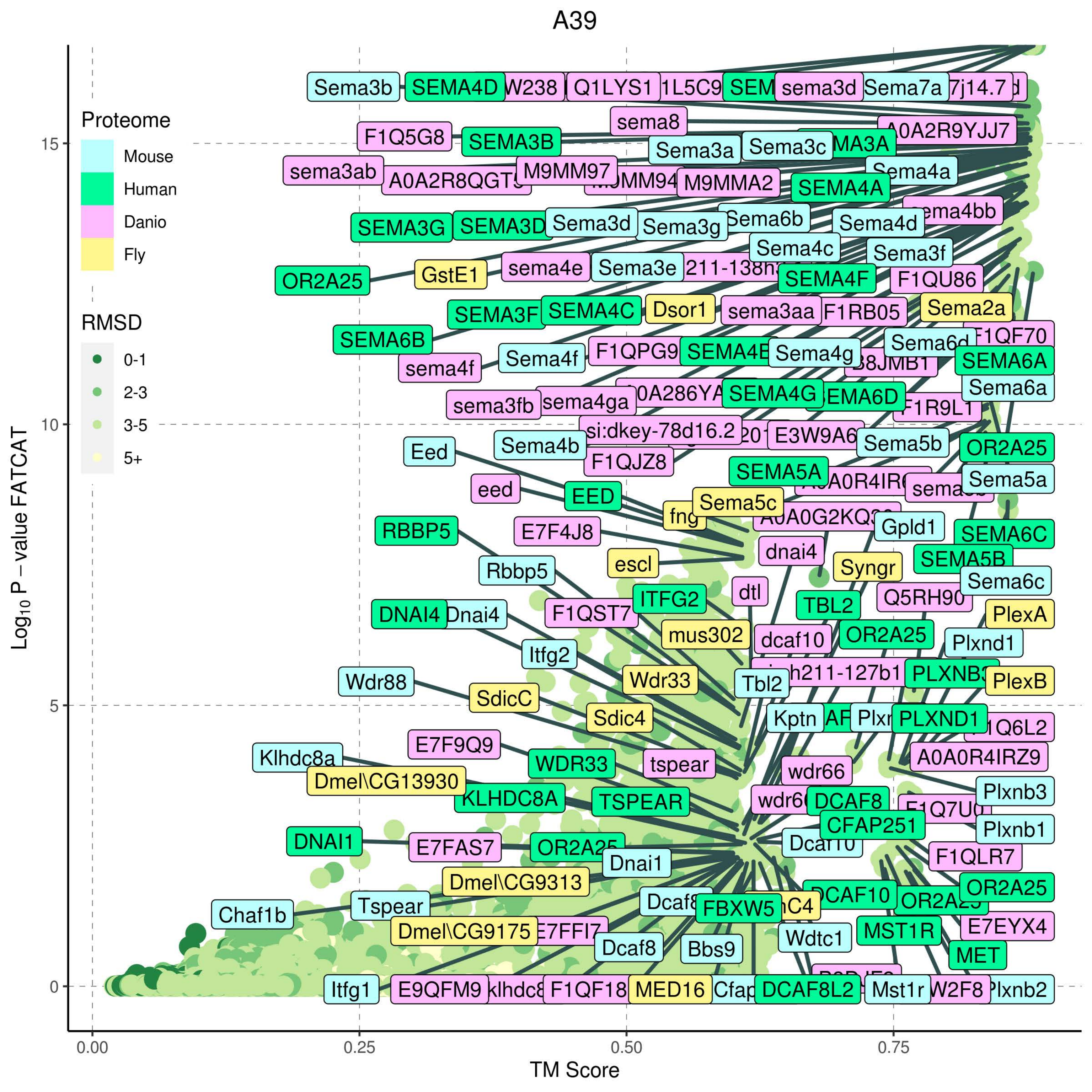
A39
Proteome
Mouse
Human
Danio
Fly
RMSD
0-1
2-3
3-5
5+
Log10 P – value FATCAT
TM Score
0.00
0.25
0.50
0.75
0
5
10
15

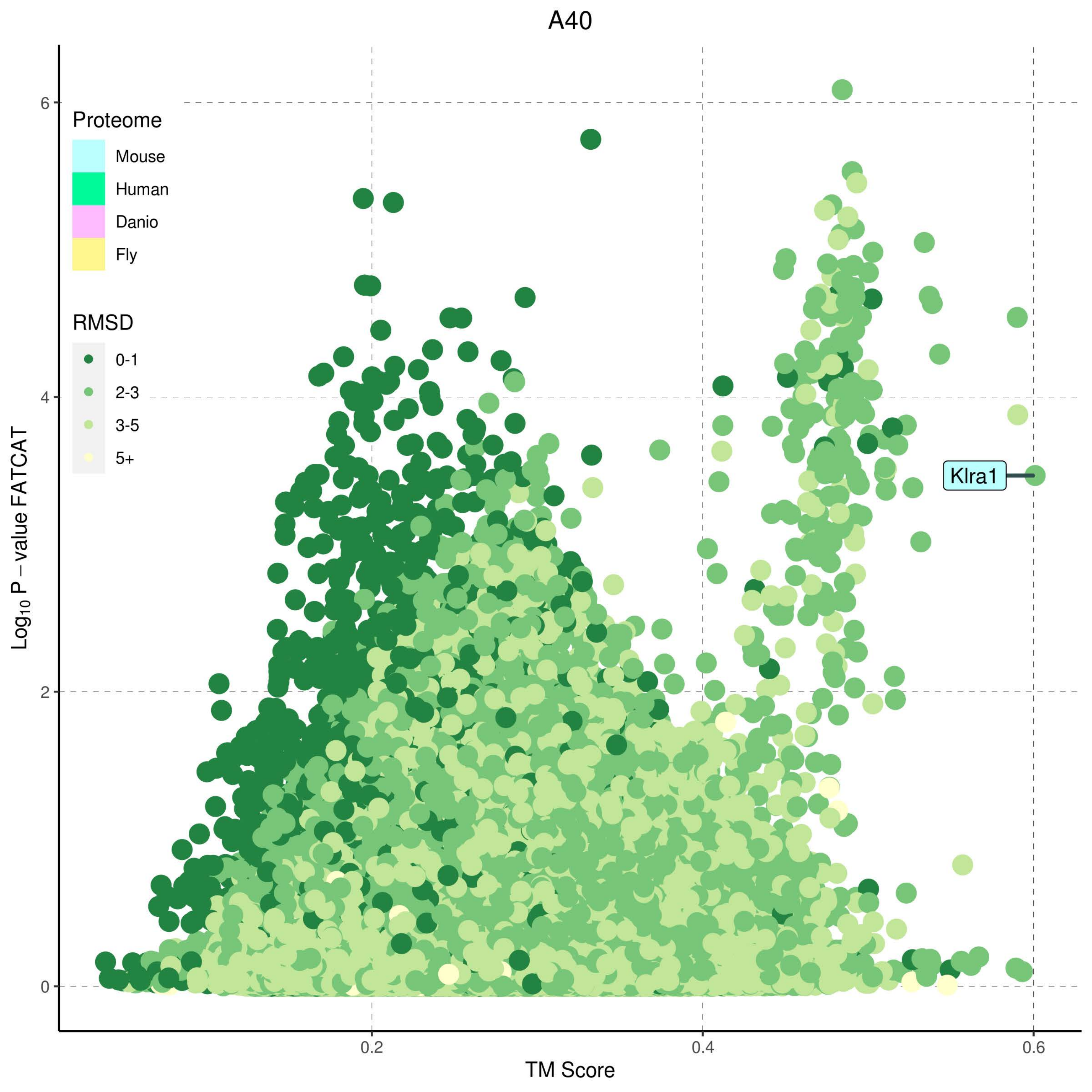
A40
Proteome
Mouse
Human
Danio
Fly
RMSD
0-1
2-3
3-5
5+
Klra1
6
4
2
0
0.2
0.4
0.6
Log10 P − value FATCAT
TM Score

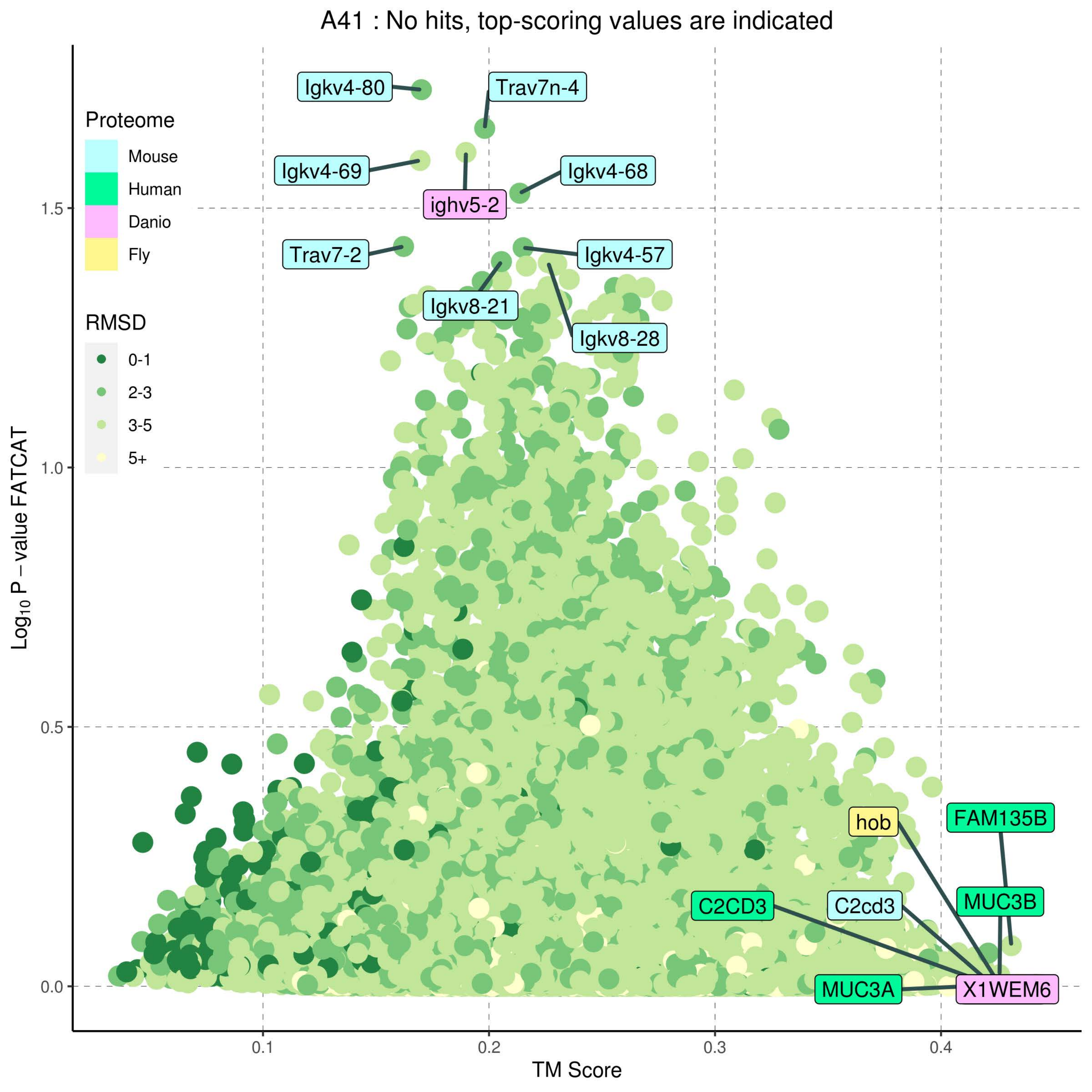

A41 : No hits, top-scoring values are indicated
Proteome
Mouse
Human
Danio
Fly
RMSD
0-1
2-3
3-5
5+
Igkv4-80
Trav7n-4
Igkv4-69
ighv5-2
Igkv4-68
Trav7-2
Igkv4-57
Igkv8-21
Igkv8-28
hob
FAM135B
C2CD3
C2cd3
MUC3B
MUC3A
X1WEM6
Log10 P − value FATCAT
TM Score
0.0
0.5
1.0
1.5
0.1
0.2
0.3
0.4

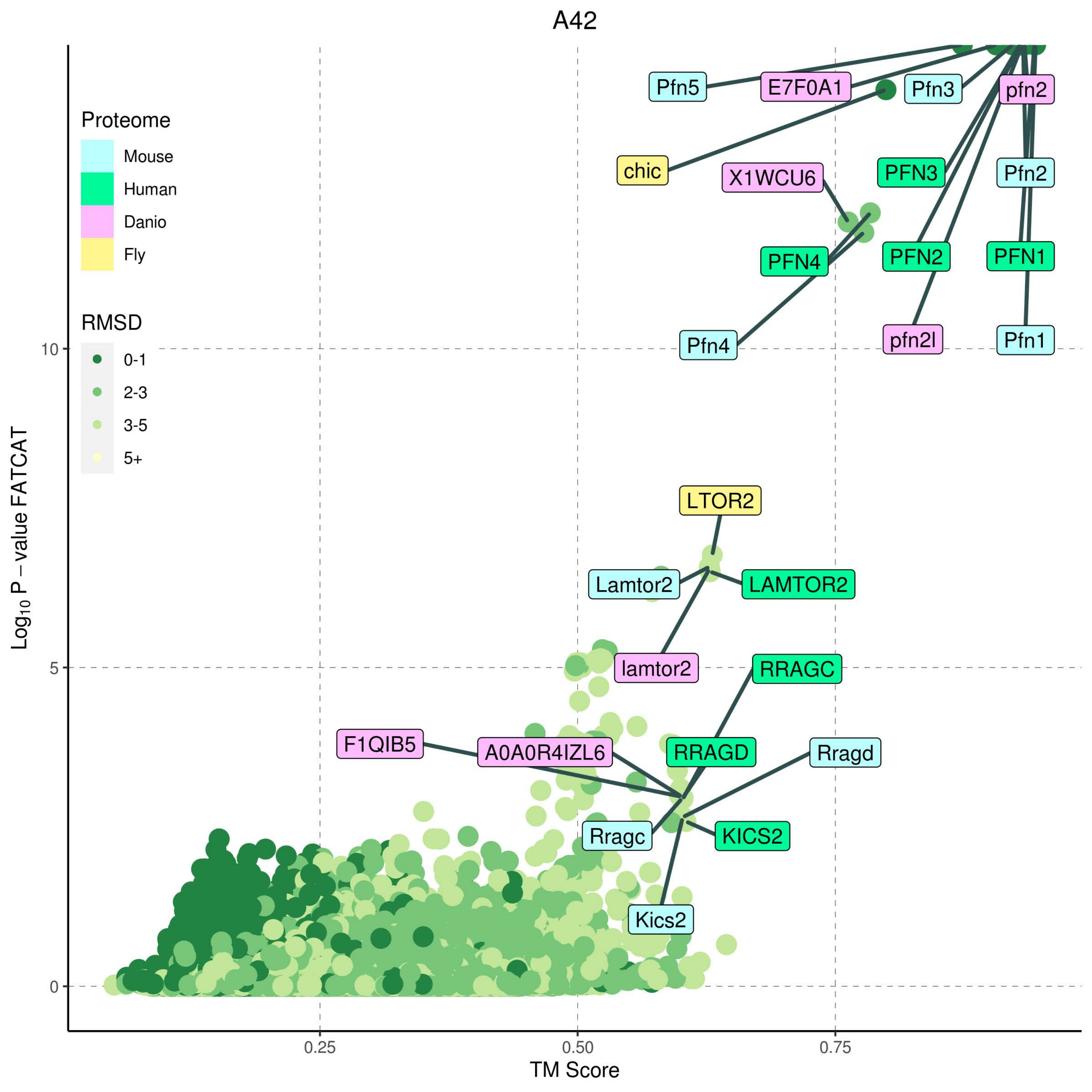

A42
Proteome
Mouse
Human
Danio
Fly
RMSD
0-1
2-3
3-5
5+
Pfn5
E7F0A1
Pfn3
pfn2
chic
X1WCU6
PFN3
Pfn2
PFN4
PFN2
PFN1
Pfn4
pfn2l
Pfn1
LTOR2
Lamtor2
LAMTOR2
lamtor2
RRAGC
F1QIB5
A0A0R4IZL6
RRAGD
Rragd
Rragc
KICS2
Kics2
10
5
0
0.25
0.50
0.75
TM Score
Log_{10} P – value FATCAT

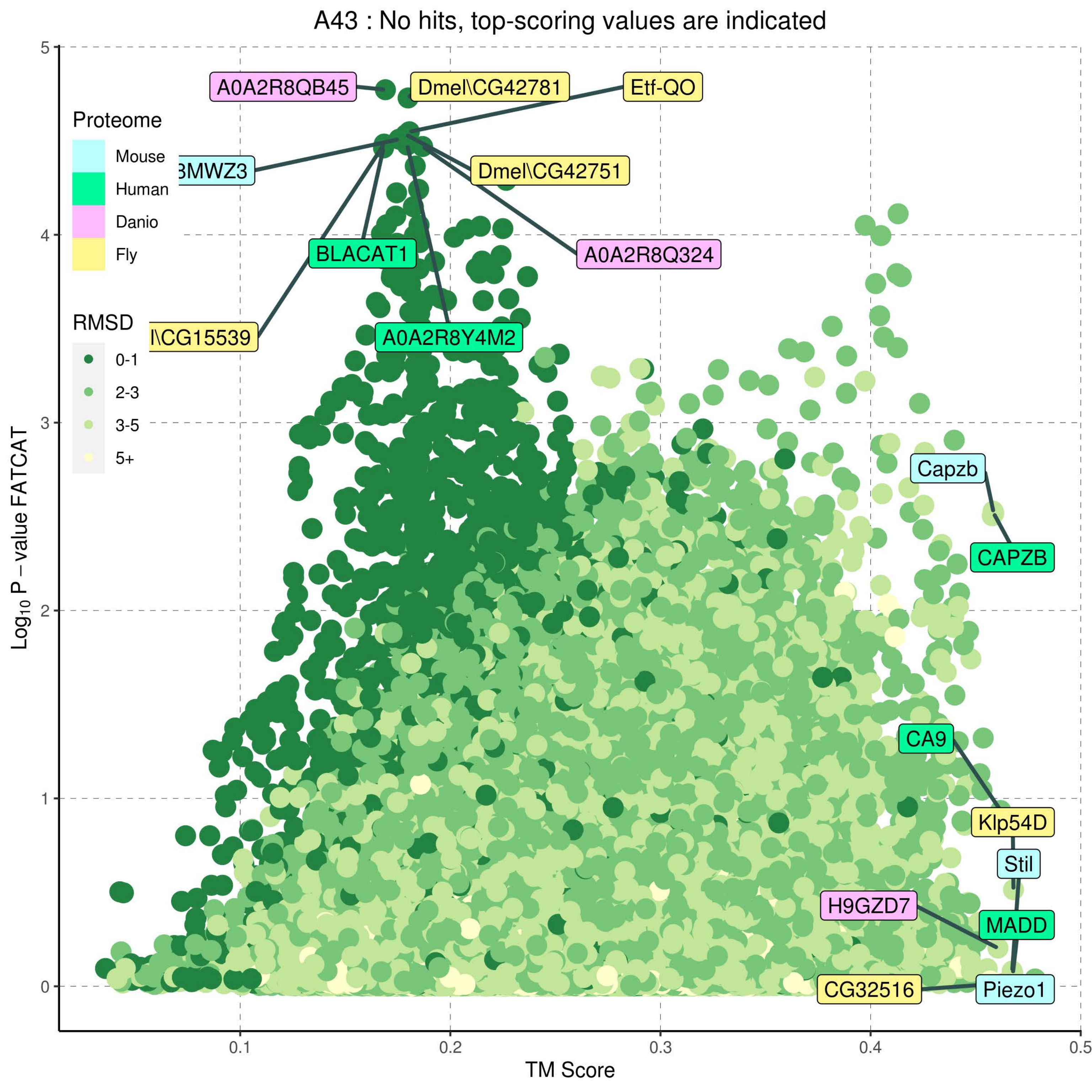

A43 : No hits, top-scoring values are indicated
Proteome
Mouse
Human
Danio
Fly
RMSD
0-1
2-3
3-5
5+
A0A2R8QB45
Dmel\CG42781
Etf-QO
3MWZ3
Dmel\CG42751
BLACAT1
A0A2R8Q324
l\CG15539
A0A2R8Y4M2
Capzb
CAPZB
CA9
Klp54D
Stil
H9GZD7
MADD
CG32516
Piezo1
Log10 P – value FATCAT
TM Score
0
1
2
3
4
5
0.1
0.2
0.3
0.4
0.5

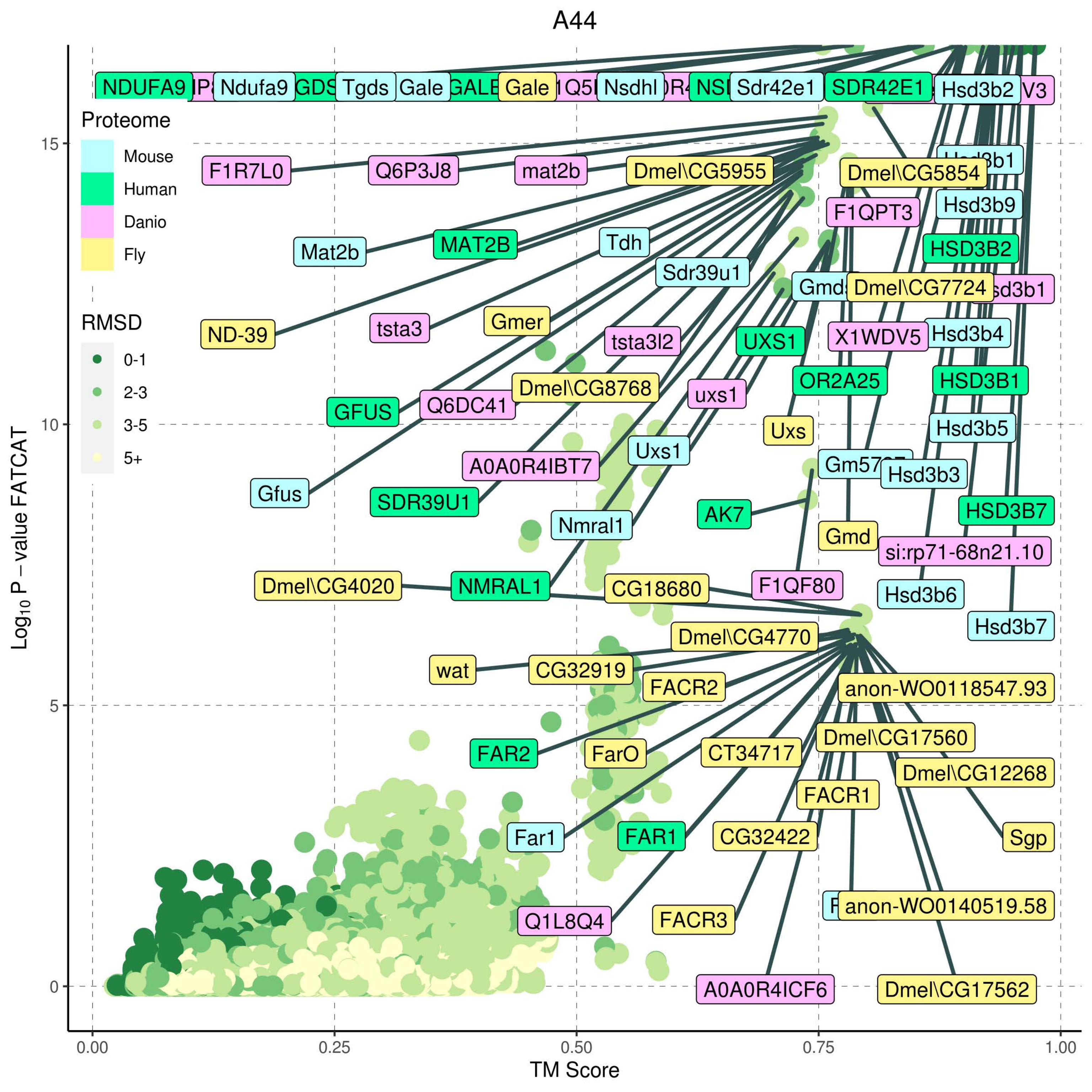
A44
Proteome
Mouse
Human
Danio
Fly
RMSD
0-1
2-3
3-5
5+
Log10 P − value FATCAT
TM Score
0.00
0.25
0.50
0.75
1.00
0
5
10
15
NDUFA9
Ndufa9
Tgds
Gale
Gale
Nsdhl
Sdr42e1
SDR42E1
Hsd3b2
F1R7L0
Q6P3J8
mat2b
Dmel\CG5955
Dmel\CG5854
Hsd3b9
F1QPT3
Mat2b
MAT2B
Tdh
HSD3B2
Sdr39u1
Dmel\CG7724
ND-39
tsta3
Gmer
tsta3l2
UXS1
X1WDV5
Hsd3b4
GFUS
Q6DC41
Dmel\CG8768
uxs1
OR2A25
HSD3B1
Uxs
Hsd3b5
Uxs1
A0A0R4IBT7
Hsd3b3
Gfus
SDR39U1
AK7
HSD3B7
Nmral1
Gmd
si:rp71-68n21.10
Dmel\CG4020
NMRAL1
CG18680
F1QF80
Hsd3b6
Hsd3b7
Dmel\CG4770
wat
CG32919
FACR2
anon-WO0118547.93
FAR2
FarO
CT34717
Dmel\CG17560
Dmel\CG12268
FACR1
Far1
FAR1
CG32422
Sgp
anon-WO0140519.58
Q1L8Q4
FACR3
A0A0R4ICF6
Dmel\CG17562

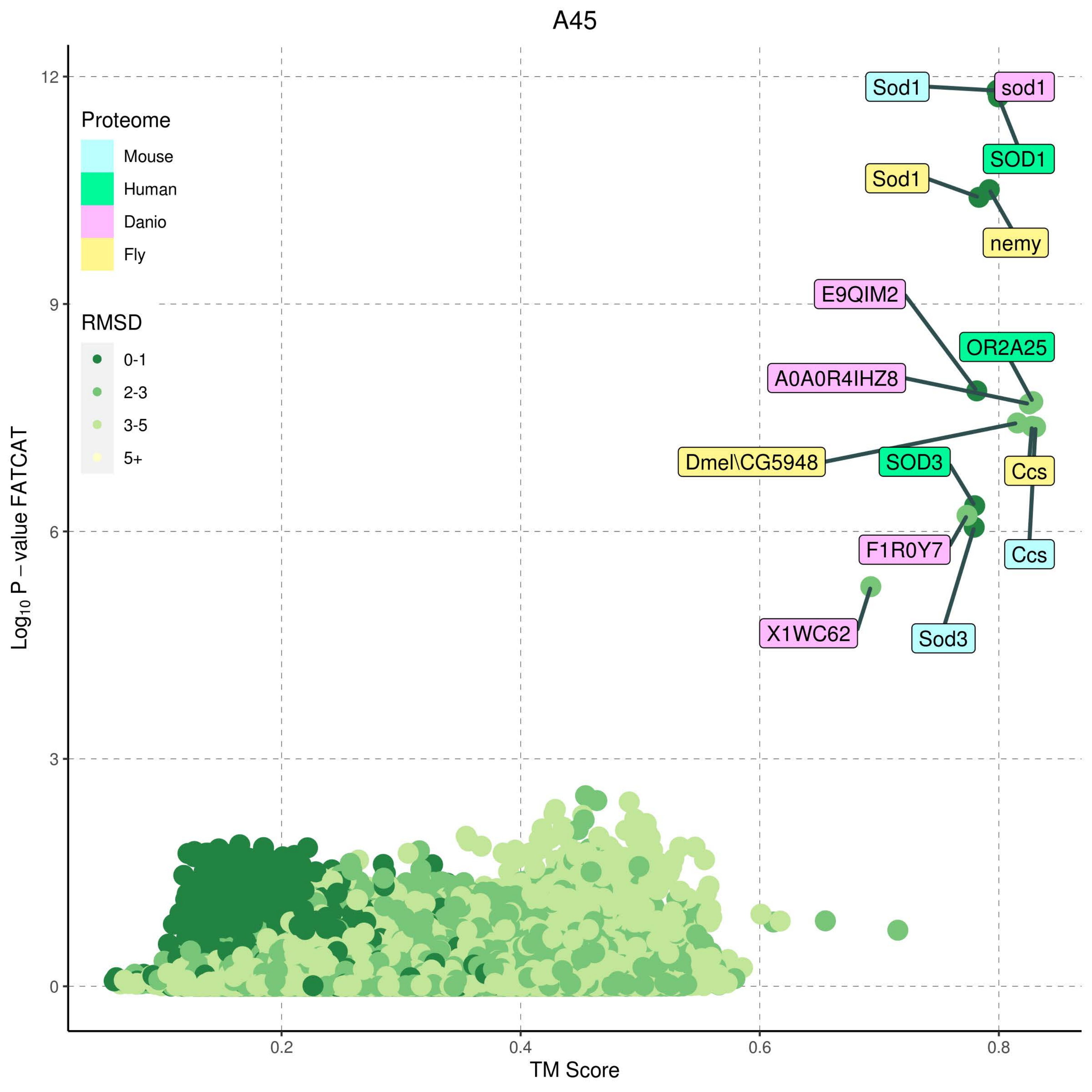

A45
Proteome
Mouse
Human
Danio
Fly
RMSD
0-1
2-3
3-5
5+
Sod1
sod1
SOD1
Sod1
nemy
E9QIM2
OR2A25
A0A0R4IHZ8
Dmel\CG5948
SOD3
Ccs
F1R0Y7
Ccs
X1WC62
Sod3
12
9
6
3
0
0.2
0.4
0.6
0.8
TM Score
Log10 P – value FATCAT

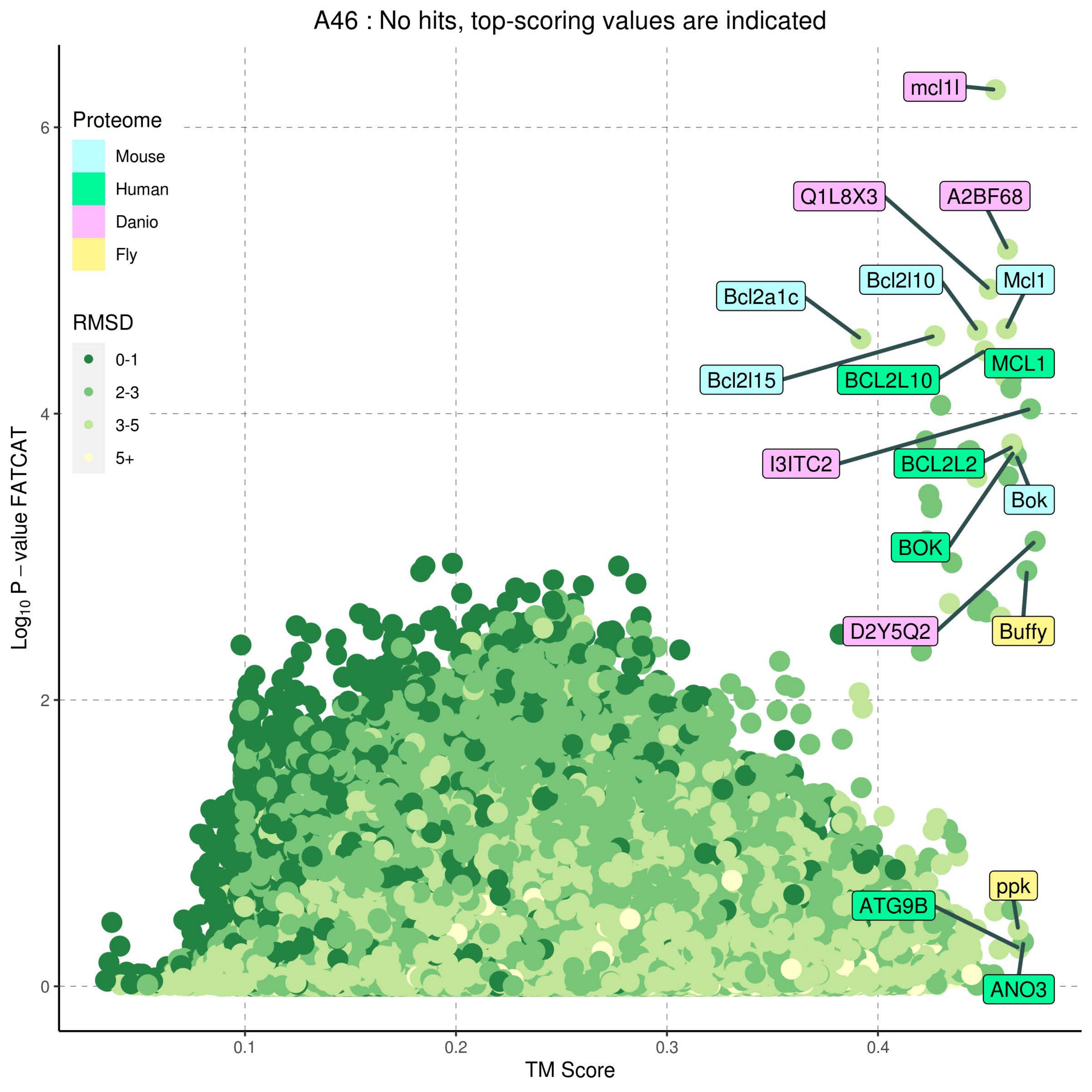

A46 : No hits, top-scoring values are indicated
Proteome
Mouse
Human
Danio
Fly
RMSD
0-1
2-3
3-5
5+
Log10 P – value FATCAT
TM Score
0
2
4
6
0.1
0.2
0.3
0.4
mcl1l
Q1L8X3
A2BF68
Bcl2l10
Mcl1
Bcl2a1c
MCL1
Bcl2l15
BCL2L10
I3ITC2
BCL2L2
Bok
BOK
D2Y5Q2
Buffy
ppk
ATG9B
ANO3

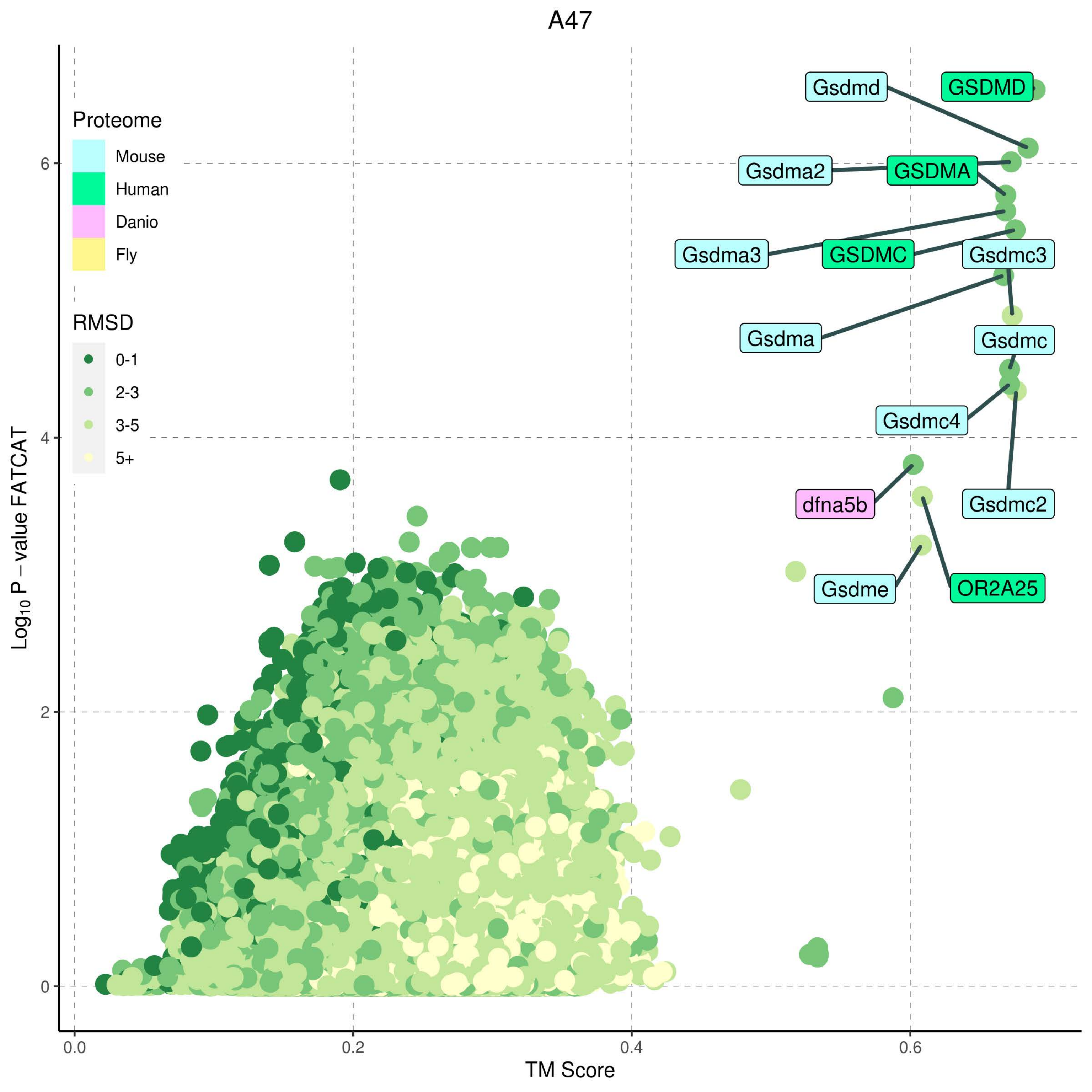
A47
Proteome
Mouse
Human
Danio
Fly
RMSD
0-1
2-3
3-5
5+
Gsdmd
GSDMD
Gsdma2
GSDMA
Gsdma3
GSDMC
Gsdmc3
Gsdma
Gsdmc
Gsdmc4
dfna5b
Gsdmc2
Gsdme
OR2A25
Log10 P – value FATCAT
TM Score
0
2
4
6
0.0
0.2
0.4
0.6

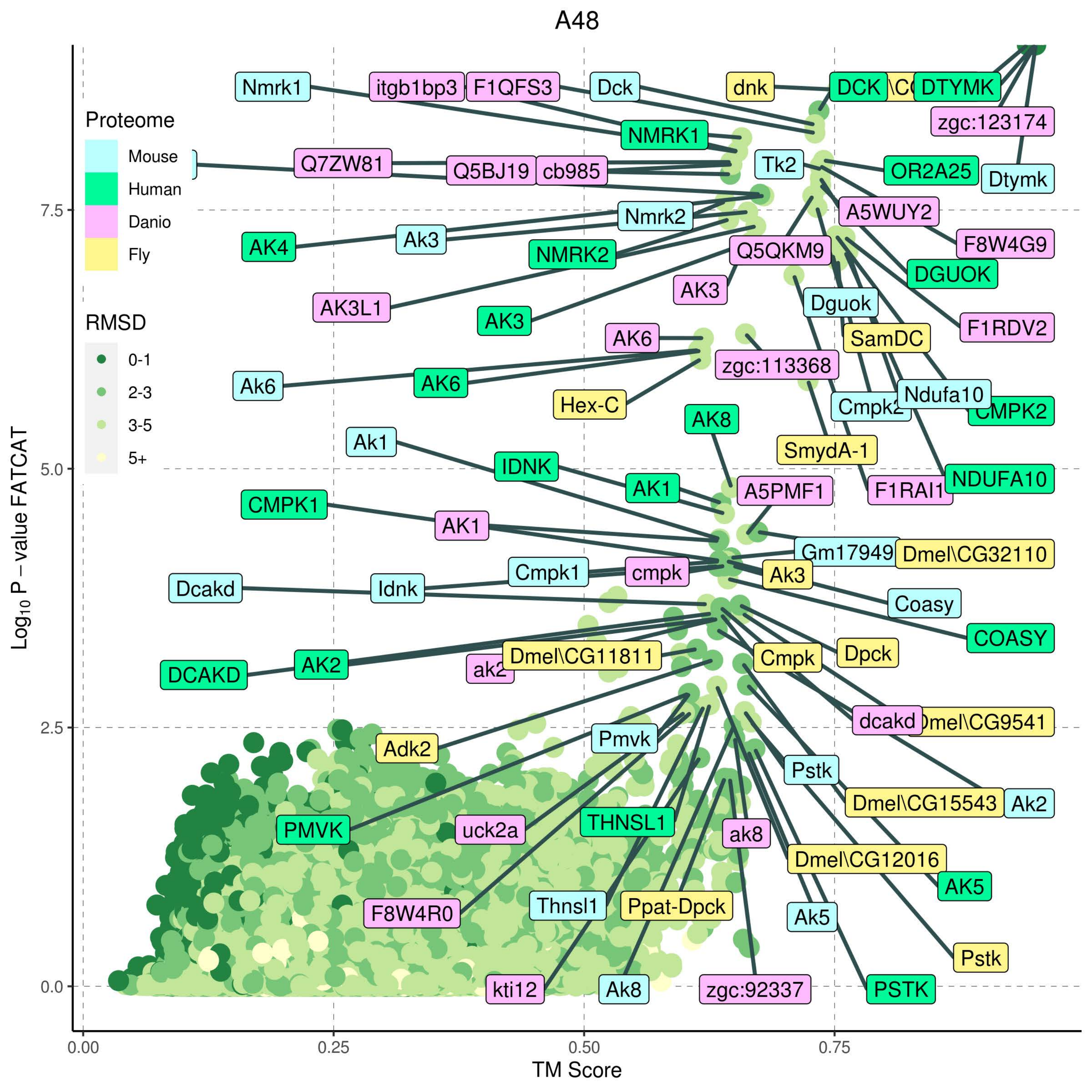

A48
Proteome
Mouse
Human
Danio
Fly
RMSD
0-1
2-3
3-5
5+
Log10 P − value FATCAT
TM Score
0.00
0.25
0.50
0.75
0.0
2.5
5.0
7.5
Nmrk1
itgb1bp3
F1QFS3
Dck
dnk
DCK
DTYMK
zgc:123174
NMRK1
Q7ZW81
Q5BJ19
cb985
Tk2
OR2A25
Dtymk
Nmrk2
A5WUY2
AK4
Ak3
NMRK2
Q5QKM9
F8W4G9
DGUOK
AK3
AK3L1
AK3
Dguok
F1RDV2
AK6
SamDC
zgc:113368
Ak6
AK6
Hex-C
Ndufa10
AK8
Ak1
SmydA-1
IDNK
NDUFA10
AK1
A5PMF1
F1RAI1
CMPK1
AK1
Gm17949
Dmel\CG32110
Cmpk1
cmpk
Ak3
Dcakd
Idnk
Coasy
COASY
Dmel\CG11811
Dpck
Cmpk
DCAKD
AK2
dcakd
Adk2
Pmvk
Pstk
Dmel\CG15543
Ak2
PMVK
uck2a
THNSL1
ak8
Dmel\CG12016
AK5
F8W4R0
Thnsl1
Ppat-Dpck
Ak5
Pstk
kti12
Ak8
zgc:92337
PSTK

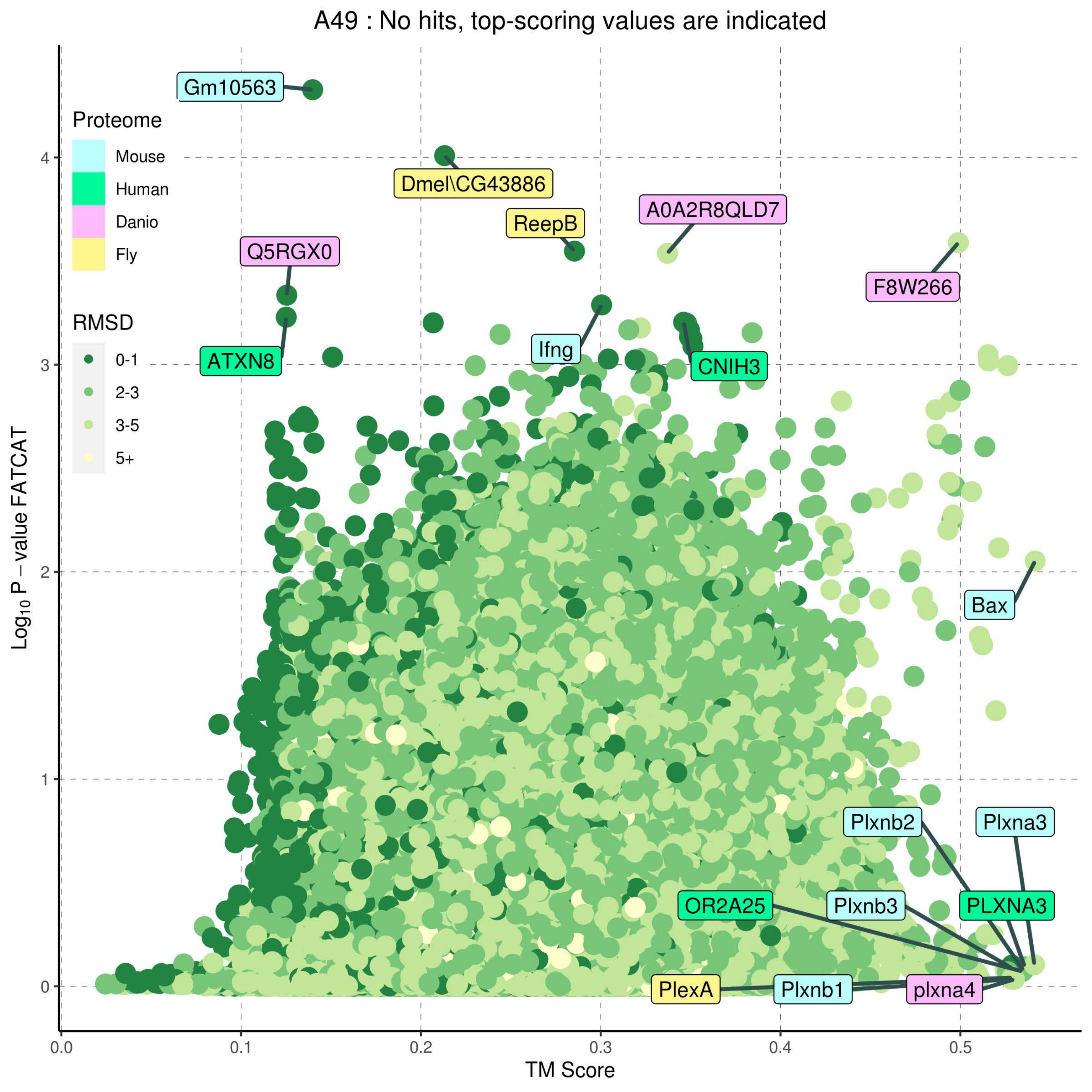

A49 : No hits, top-scoring values are indicated
Proteome
Mouse
Human
Danio
Fly
RMSD
0-1
2-3
3-5
5+
Gm10563
Dmel\CG43886
ReepB
A0A2R8QLD7
Q5RGX0
F8W266
Ifng
CNIH3
ATXN8
Bax
Plxnb2
Plxna3
OR2A25
Plxnb3
PLXNA3
PlexA
Plxnb1
plxna4
Log_{10} P – value FATCAT
TM Score
0
1
2
3
4
0.0
0.1
0.2
0.3
0.4
0.5

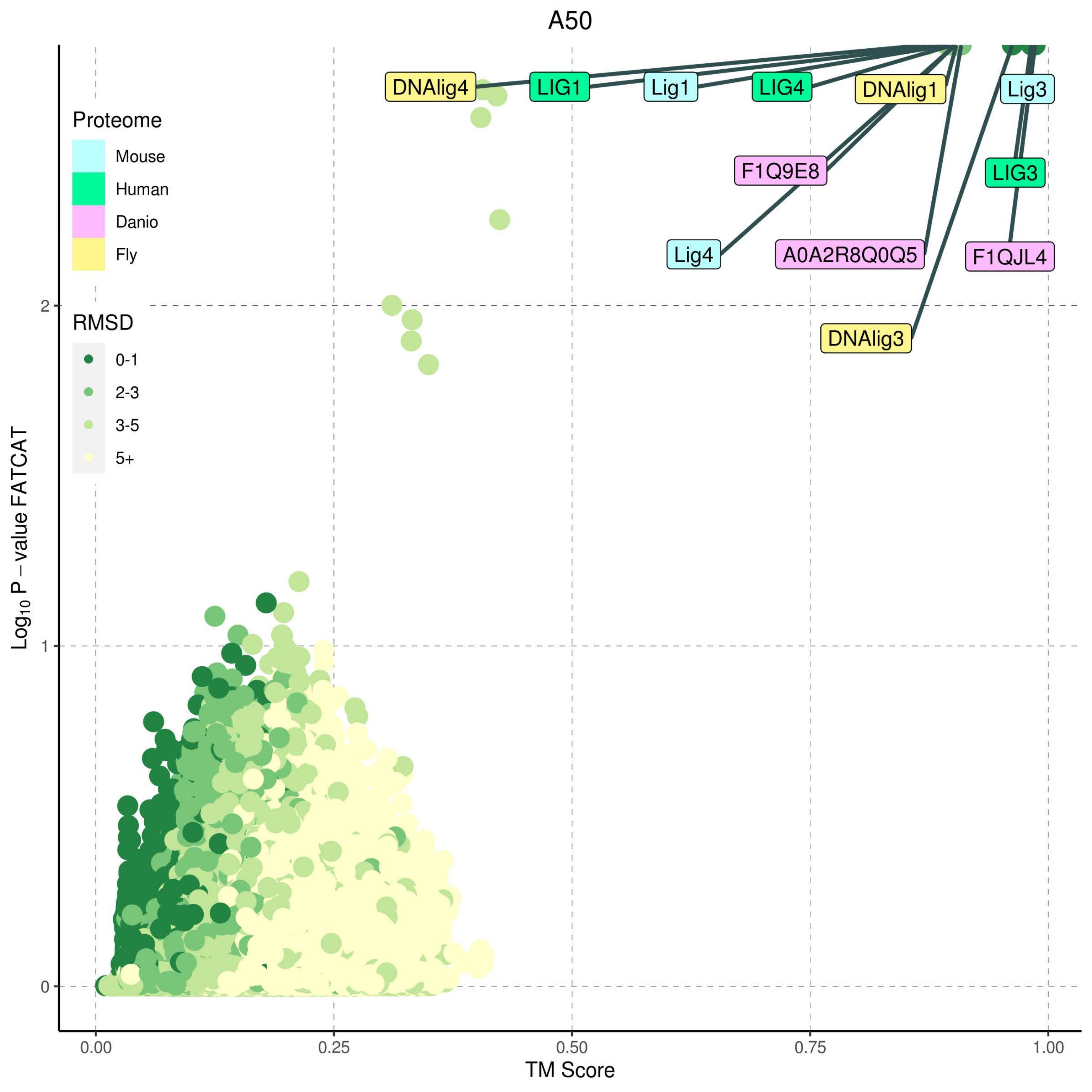
A50
Proteome
Mouse
Human
Danio
Fly
RMSD
0-1
2-3
3-5
5+
DNAlig4
LIG1
Lig1
LIG4
DNAlig1
Lig3
F1Q9E8
LIG3
Lig4
A0A2R8Q0Q5
F1QJL4
DNAlig3
Log10 P – value FATCAT
TM Score
0
1
2
0.00
0.25
0.50
0.75
1.00

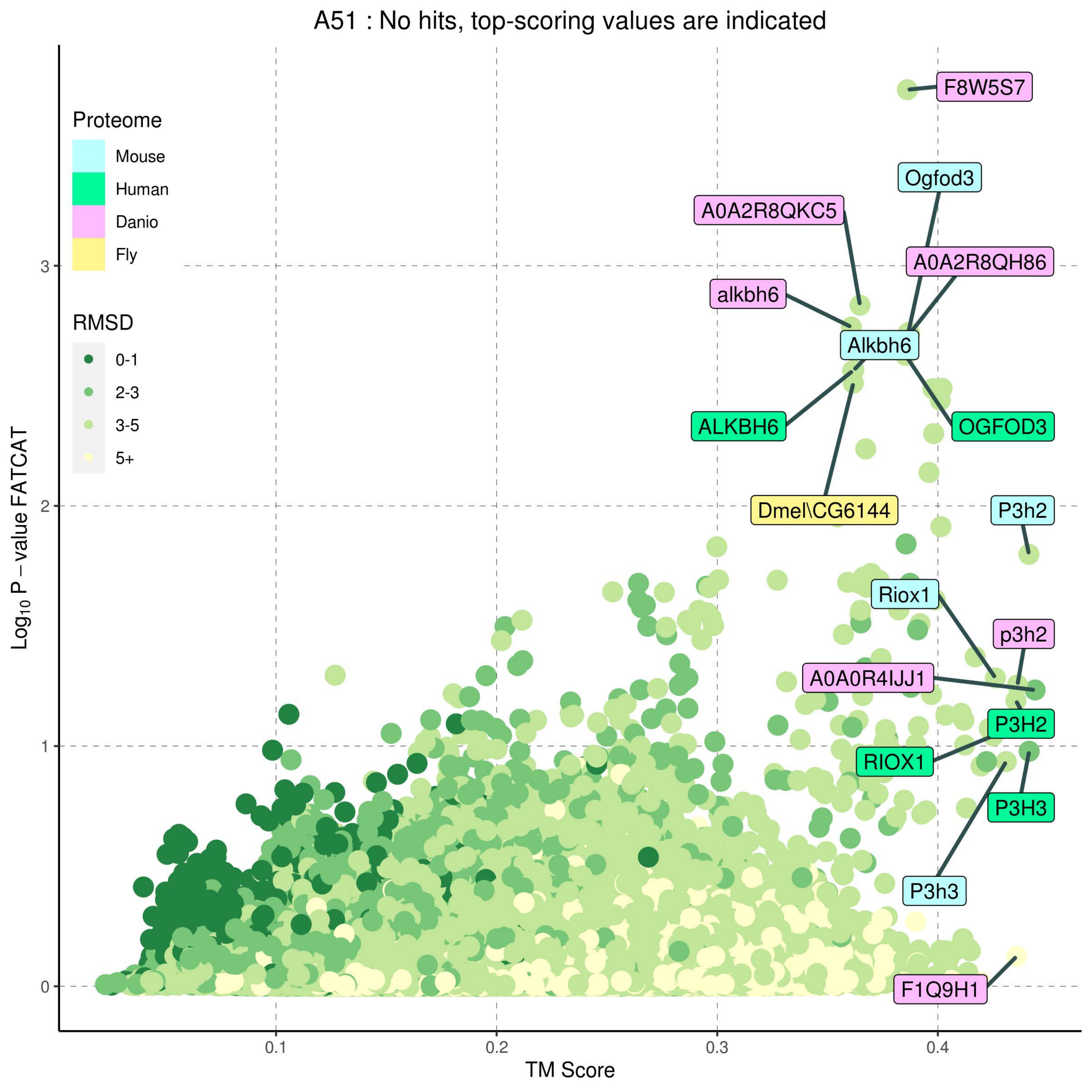
A51 : No hits, top-scoring values are indicated
Proteome
Mouse
Human
Danio
Fly
RMSD
0-1
2-3
3-5
5+
F8W5S7
Ogfod3
A0A2R8QKC5
A0A2R8QH86
alkbh6
Alkbh6
ALKBH6
OGFOD3
Dmel\CG6144
P3h2
Riox1
p3h2
A0A0R4IJJ1
P3H2
RIOX1
P3H3
P3h3
F1Q9H1
Log_{10} P − value FATCAT
TM Score
0
1
2
3
0.1
0.2
0.3
0.4

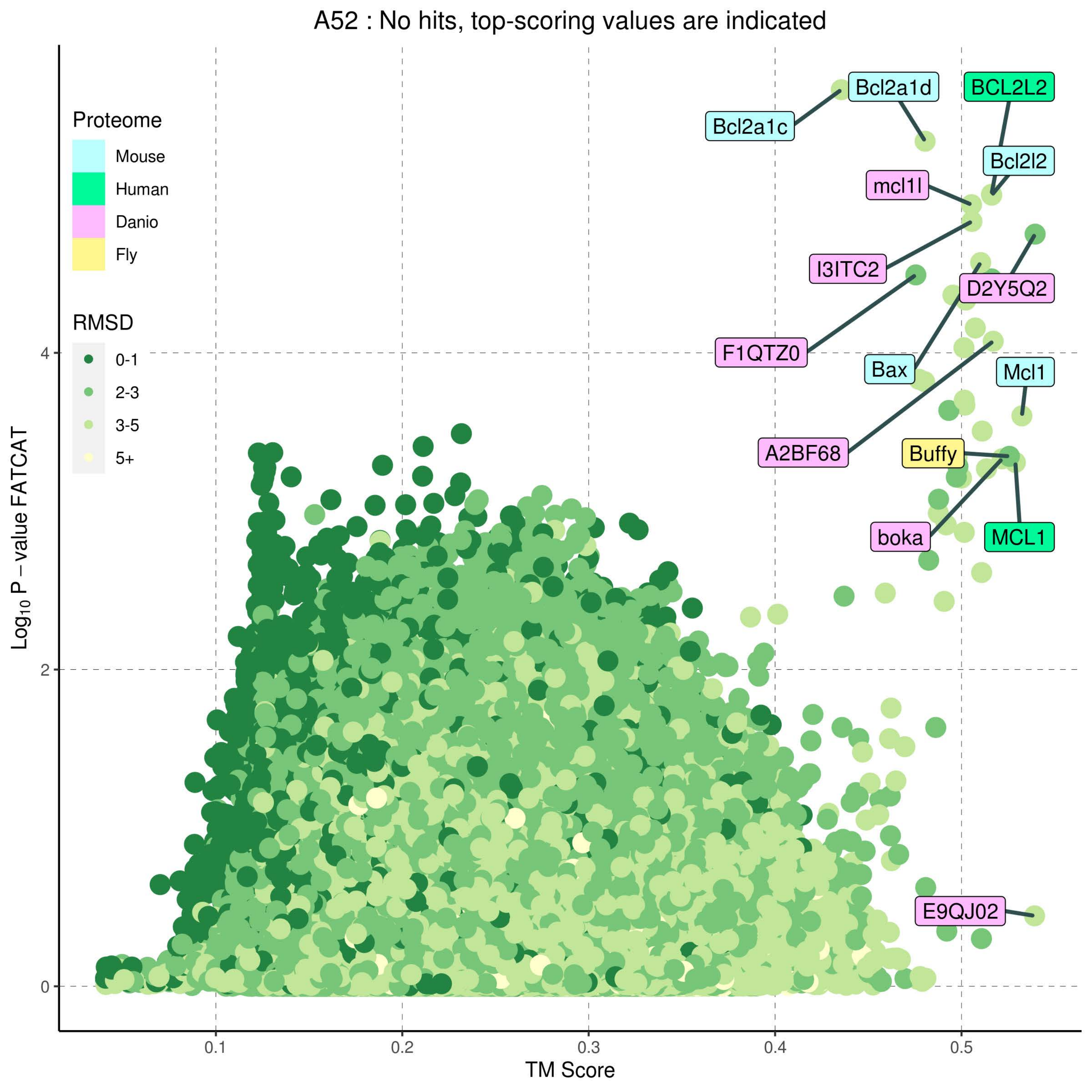

A52 : No hits, top-scoring values are indicated
Proteome
Mouse
Human
Danio
Fly
RMSD
0-1
2-3
3-5
5+
Bcl2a1d
BCL2L2
Bcl2a1c
Bcl2l2
mcl1l
I3ITC2
D2Y5Q2
F1QTZ0
Bax
Mcl1
A2BF68
Buffy
boka
MCL1
E9QJ02
Log10 P – value FATCAT
TM Score
0
2
4
0.1
0.2
0.3
0.4
0.5

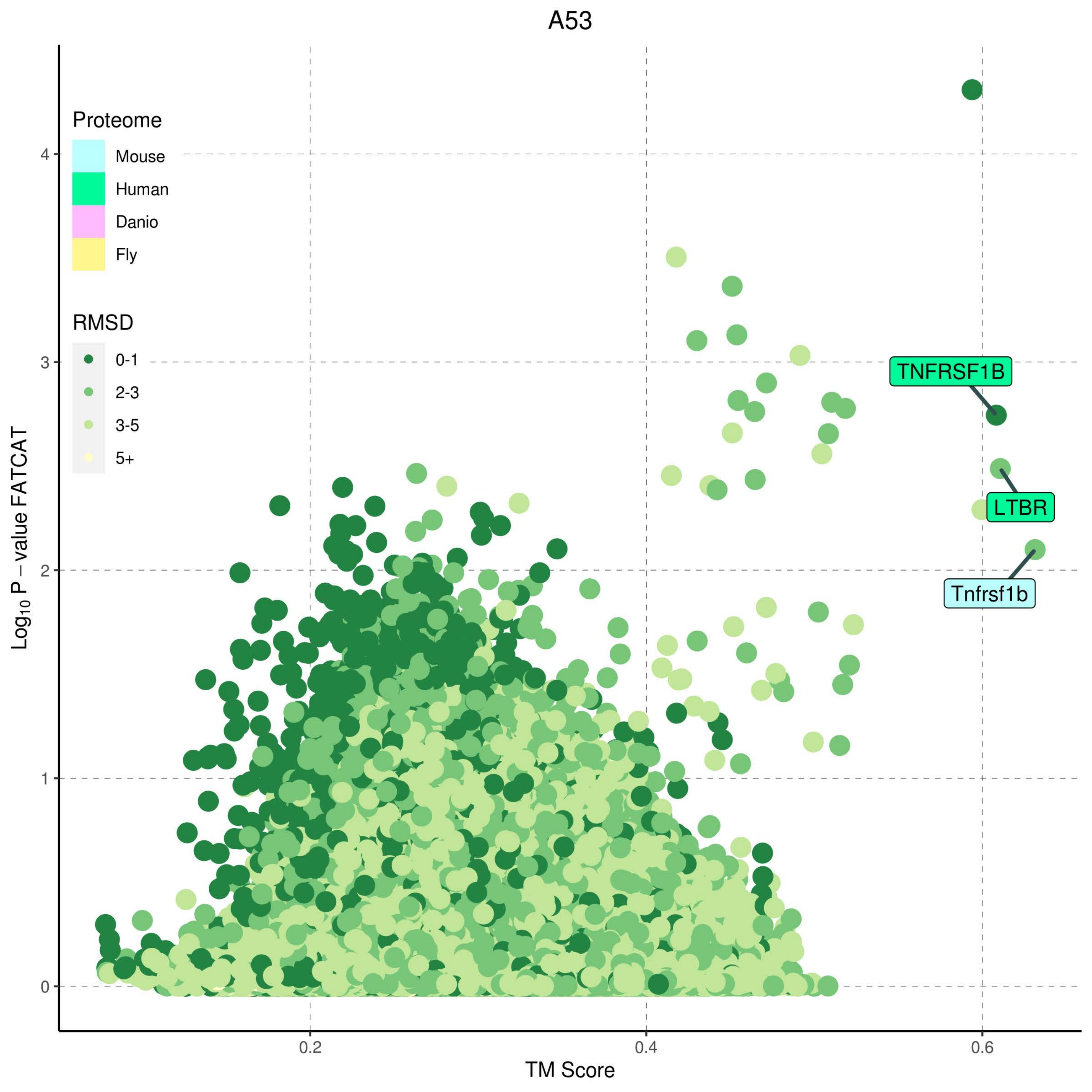

A53
Proteome
Mouse
Human
Danio
Fly
RMSD
0-1
2-3
3-5
5+
TNFRSF1B
LTBR
Tnfrsf1b
Log10 P − value FATCAT
TM Score
0
1
2
3
4
0.2
0.4
0.6

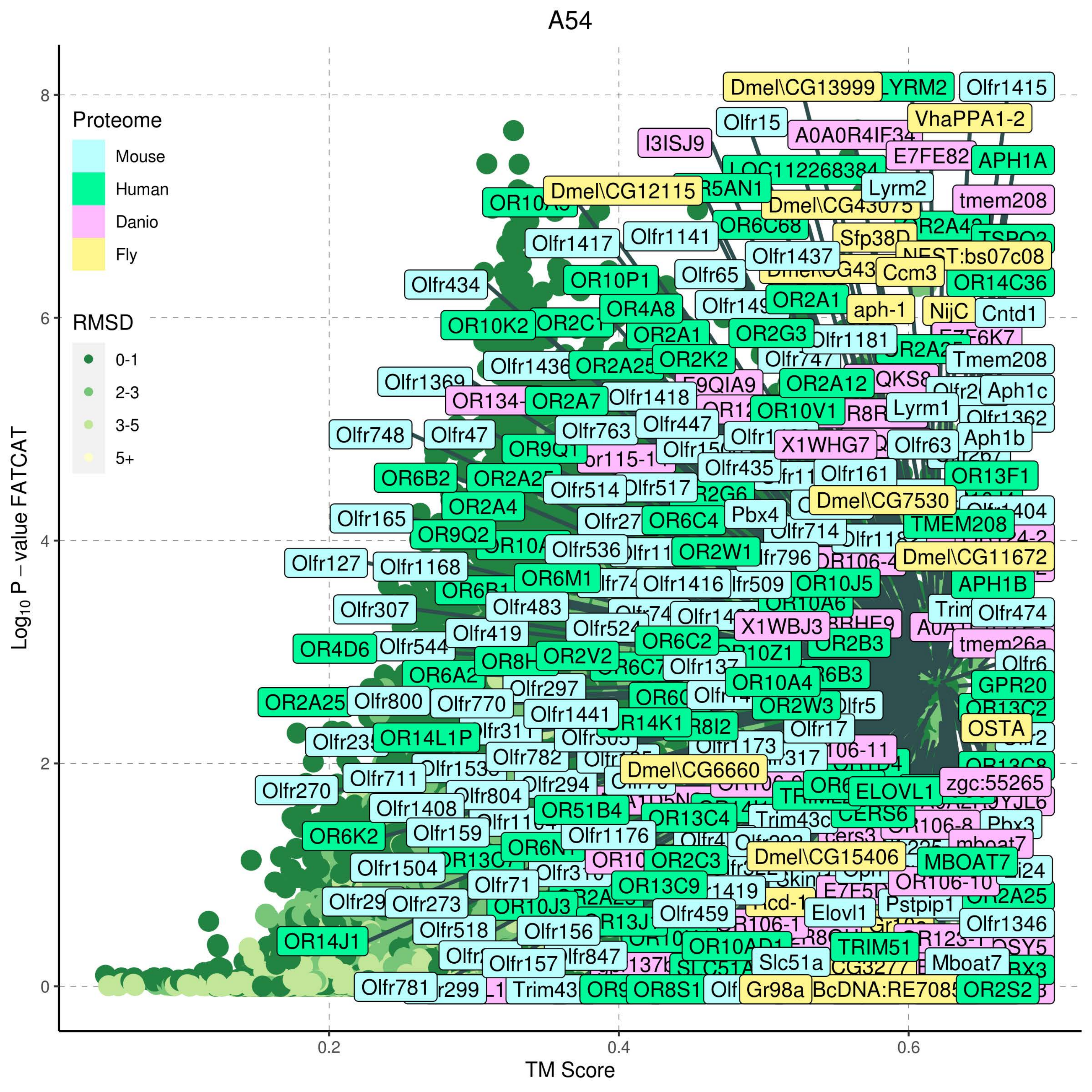
A54
Proteome
Mouse
Human
Danio
Fly
RMSD
0-1
2-3
3-5
5+
Log10 P – value FATCAT
TM Score
0
2
4
6
8
0.2
0.4
0.6
Dmel\CG13999
LYRM2
Olfr1415
Olfr15
VhaPPA1-2
I3ISJ9
E7FE82
APH1A
Dmel\CG12115
Lyrm2
tmem208
Olfr1417
Olfr1141
Olfr1437
Ccm3
Olfr434
OR10P1
Olfr65
OR14C36
OR10K2
OR4A8
OR2A1
aph-1
NijC
Cntd1
OR2G3
Olfr1181
Olfr1436
OR2K2
Tmem208
Olfr1369
OR2A7
Olfr1418
OR2A12
Aph1c
OR10V1
Lyrm1
Olfr748
Olfr47
Olfr763
Olfr447
X1WHG7
Olfr63
Aph1b
Olfr435
Olfr161
OR6B2
Olfr514
OR13F1
Dmel\CG7530
OR2A4
Pbx4
Olfr165
OR6C4
Olfr714
TMEM208
OR9Q2
Olfr536
OR2W1
Dmel\CG11672
Olfr127
Olfr1168
OR6M1
Olfr1416
OR10J5
APH1B
Olfr307
Olfr483
Olfr474
Olfr419
Olfr524
X1WBJ3
OR4D6
Olfr544
OR2V2
OR6C2
OR2B3
OR10A4
GPR20
OR2A25
Olfr800
Olfr770
Olfr297
OR2W3
Olfr1441
OSTA
OR14L1P
Olfr17
Olfr782
Olfr1173
Olfr270
Olfr711
Dmel\CG6660
zgc:55265
Olfr804
Olfr294
ELOVL1
Olfr1408
OR51B4
OR13C4
CERS6
Pbx3
OR6K2
Olfr159
Olfr1176
Dmel\CG15406
MBOAT7
Olfr1504
OR2C3
OR13C9
Olfr71
Elovl1
Pstpip1
Olfr273
OR10J3
Olfr459
Olfr1346
Olfr518
Olfr156
OR14J1
OR10AD1
TRIM51
Olfr157
Olfr847
Slc51a
Mboat7
Olfr781
Trim43
OR8S1
Gr98a
OR2S2

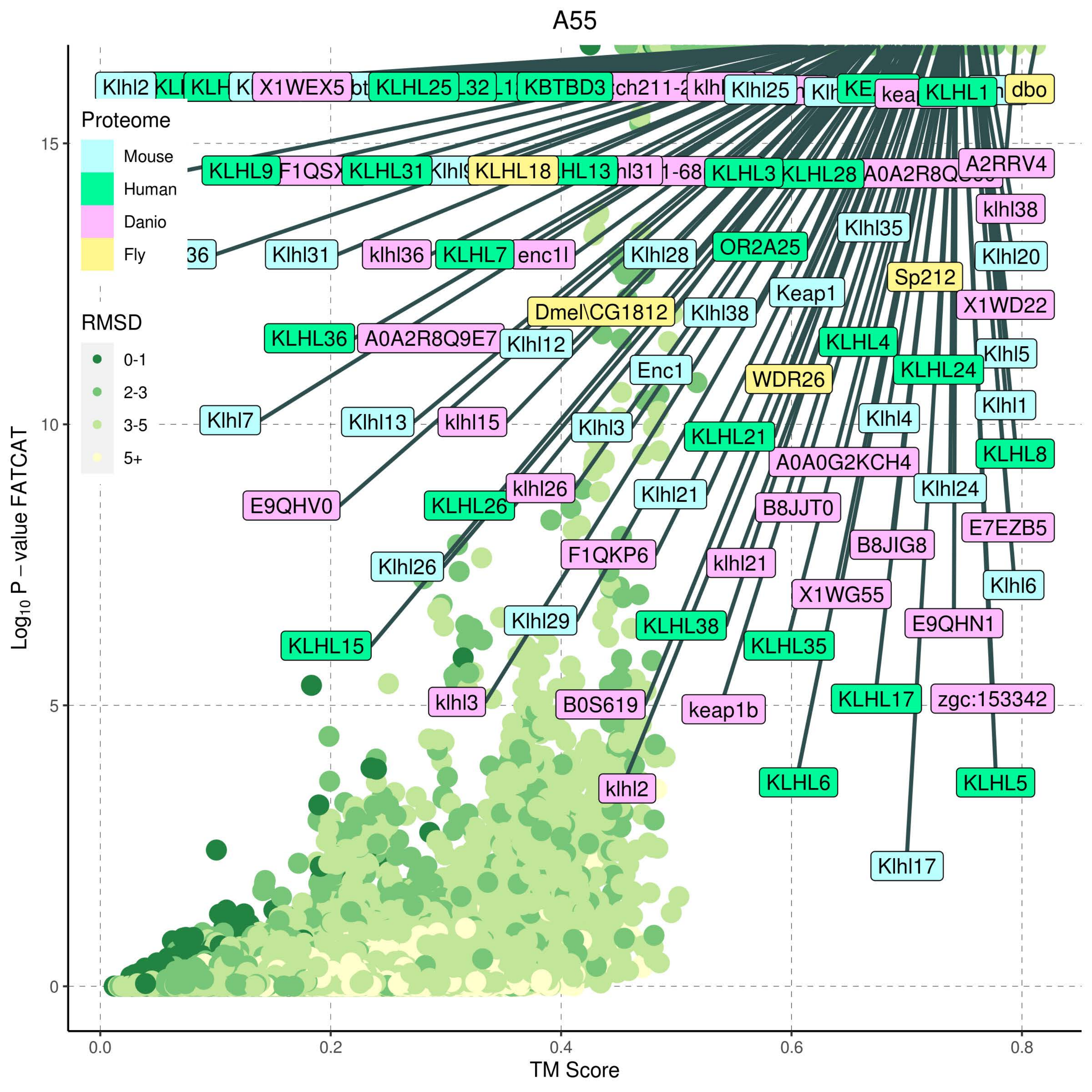

A55
Proteome
Mouse
Human
Danio
Fly
RMSD
0-1
2-3
3-5
5+
Log10 P − value FATCAT
TM Score
0.0
0.2
0.4
0.6
0.8
0
5
10
15
Klhl2
KBTBD3
KLHL25
X1WEX5
Klhl25
keap
KLHL1
dbo
KLHL9
KLHL31
KLHL18
KLHL3
KLHL28
A2RRV4
klhl38
Klhl35
Klhl20
Klhl31
klhl36
KLHL7
enc1l
Klhl28
OR2A25
Sp212
Keap1
X1WD22
Dmel\CG1812
Klhl38
KLHL36
A0A2R8Q9E7
Klhl12
KLHL4
Klhl5
Enc1
WDR26
KLHL24
Klhl1
Klhl7
Klhl13
klhl15
Klhl3
Klhl4
KLHL21
KLHL8
A0A0G2KCH4
klhl26
Klhl21
Klhl24
E9QHV0
KLHL26
B8JJT0
E7EZB5
B8JIG8
F1QKP6
Klhl26
klhl21
X1WG55
Klhl6
Klhl29
KLHL38
E9QHN1
KLHL15
KLHL35
klhl3
B0S619
keap1b
KLHL17
zgc:153342
klhl2
KLHL6
KLHL5
Klhl17

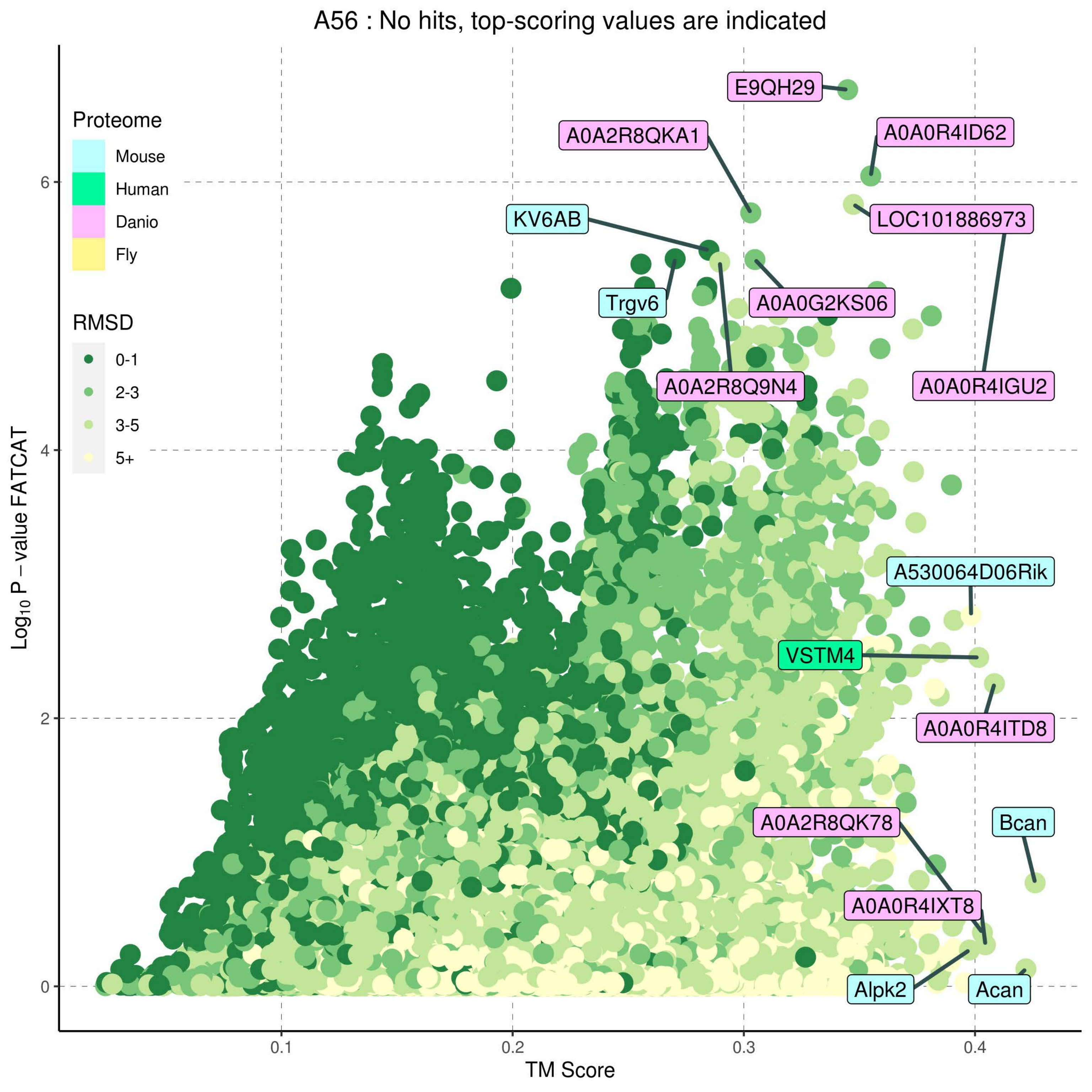
A56 : No hits, top-scoring values are indicated
Proteome
Mouse
Human
Danio
Fly
RMSD
0-1
2-3
3-5
5+
Log10 P – value FATCAT
TM Score
0
2
4
6
0.1
0.2
0.3
0.4
E9QH29
A0A2R8QKA1
A0A0R4ID62
KV6AB
LOC101886973
Trgv6
A0A0G2KS06
A0A2R8Q9N4
A0A0R4IGU2
A530064D06Rik
VSTM4
A0A0R4ITD8
A0A2R8QK78
Bcan
A0A0R4IXT8
Alpk2
Acan

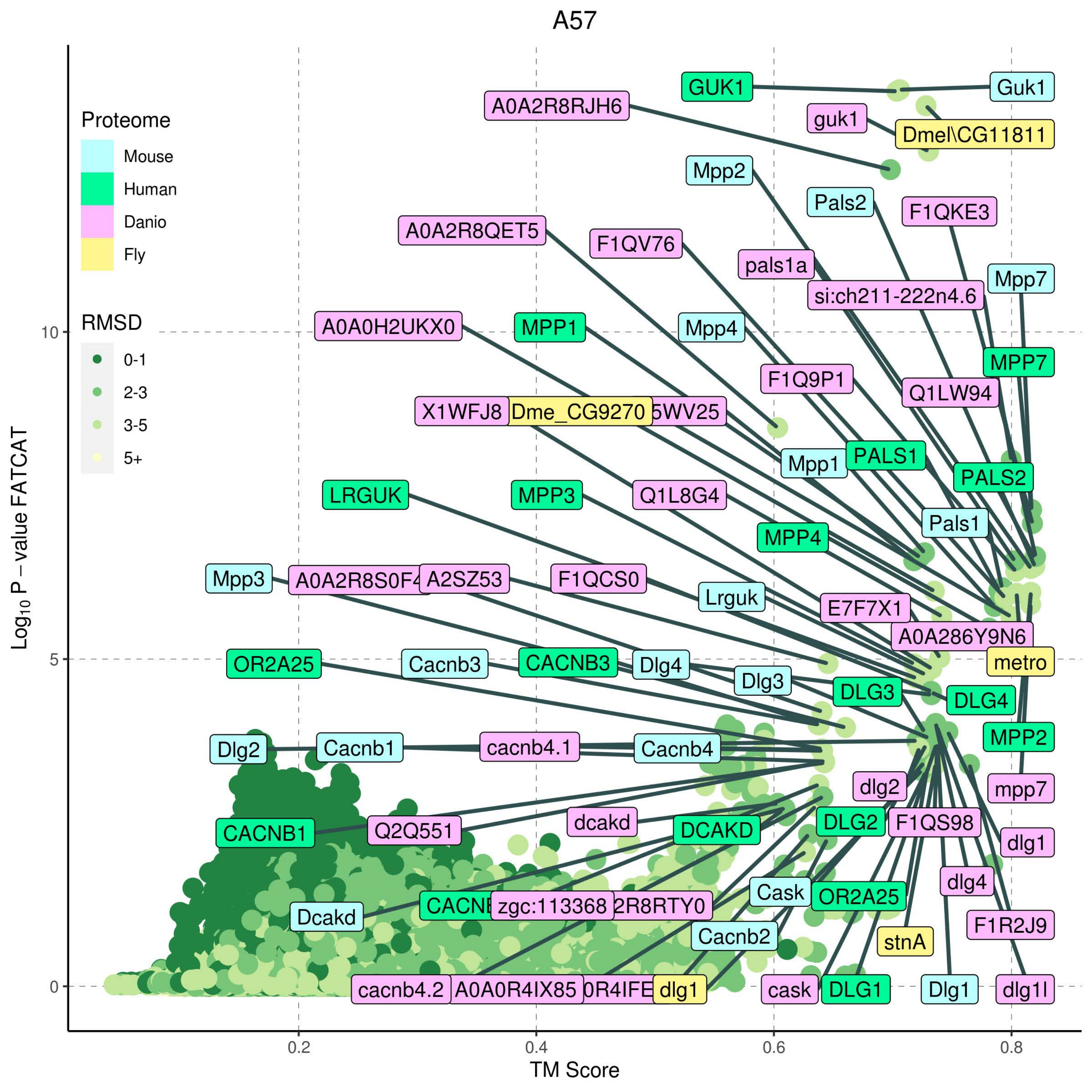

A57
Proteome
Mouse
Human
Danio
Fly
RMSD
0-1
2-3
3-5
5+
Log10 P – value FATCAT
TM Score
0
5
10
0.2
0.4
0.6
0.8
GUK1
Guk1
A0A2R8RJH6
guk1
Dmel\CG11811
Mpp2
Pals2
F1QKE3
A0A2R8QET5
F1QV76
pals1a
Mpp7
si:ch211-222n4.6
A0A0H2UKX0
MPP1
Mpp4
MPP7
F1Q9P1
Q1LW94
X1WFJ8
Dme_CG9270
5WV25
Mpp1
PALS1
PALS2
LRGUK
MPP3
Q1L8G4
Pals1
MPP4
Mpp3
A0A2R8S0F4
A2SZ53
F1QCS0
Lrguk
E7F7X1
A0A286Y9N6
OR2A25
Cacnb3
CACNB3
Dlg4
metro
Dlg3
DLG3
DLG4
MPP2
Dlg2
Cacnb1
cacnb4.1
Cacnb4
dlg2
mpp7
CACNB1
Q2Q551
dcakd
DCAKD
DLG2
F1QS98
dlg1
dlg4
Cask
OR2A25
Dcakd
CACNE
zgc:113368
2R8RTY0
F1R2J9
Cacnb2
stnA
cacnb4.2
A0A0R4IX85
0R4IFE
dlg1
cask
DLG1
Dlg1
dlg1l

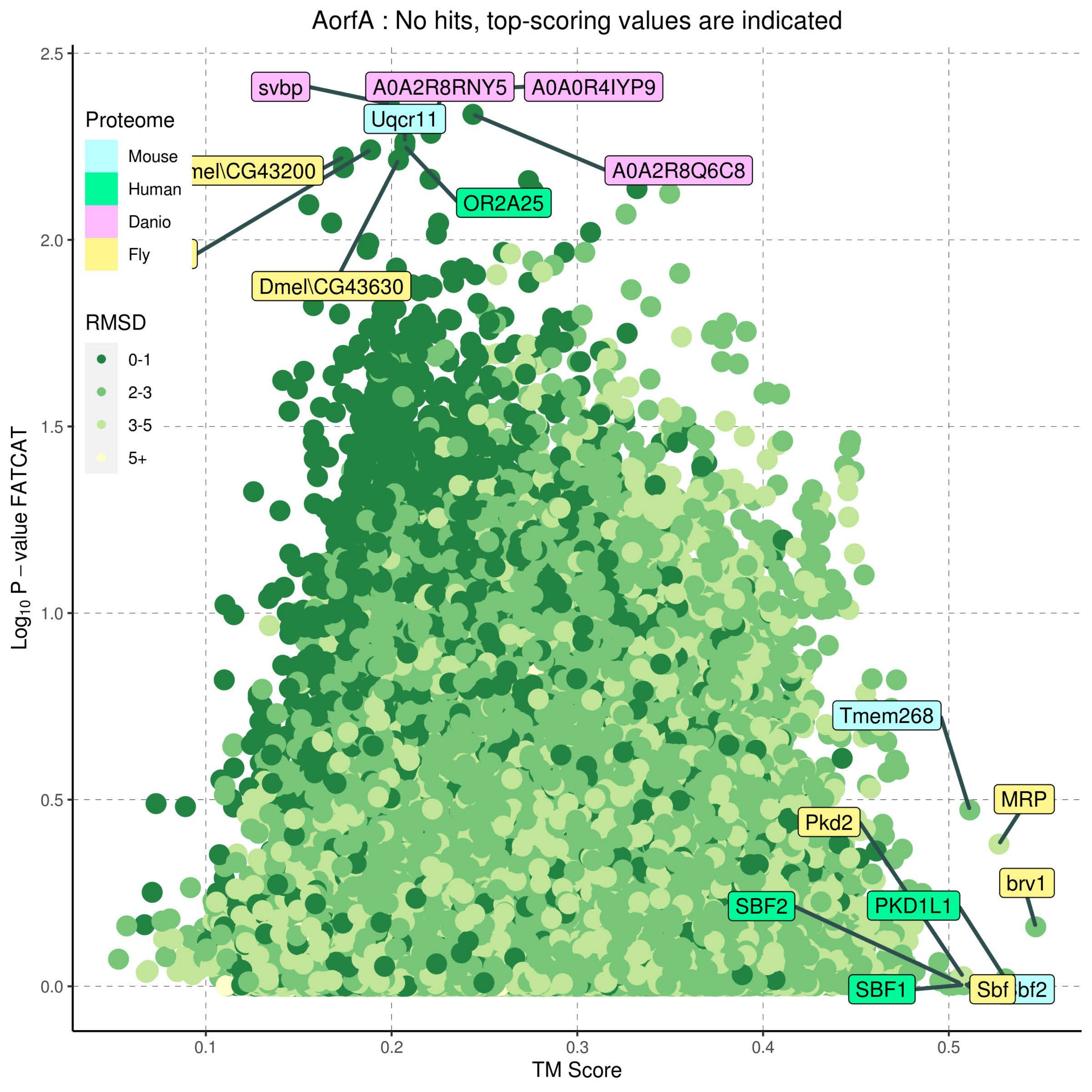
AorfA : No hits, top-scoring values are indicated
Proteome
Mouse
Human
Danio
Fly
RMSD
0-1
2-3
3-5
5+
svbp
A0A2R8RNY5
A0A0R4IYP9
Uqcr11
mel\CG43200
A0A2R8Q6C8
OR2A25
Dmel\CG43630
Tmem268
MRP
Pkd2
brv1
SBF2
PKD1L1
SBF1
Sbf
bf2
2.5
2.0
1.5
1.0
0.5
0.0
0.1
0.2
0.3
0.4
0.5
Log10 P – value FATCAT
TM Score

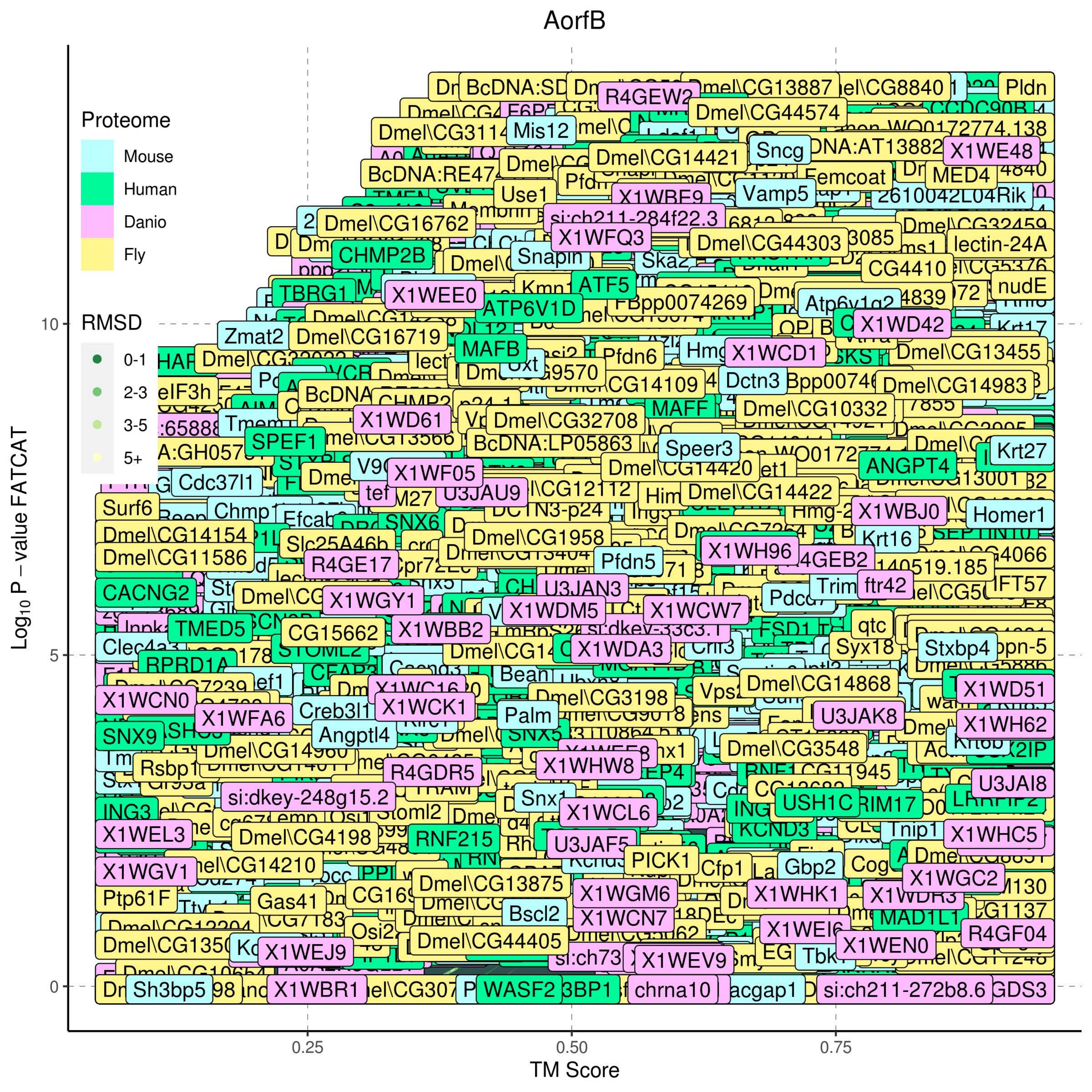
AorfB
Proteome
Mouse
Human
Danio
Fly
RMSD
0-1
2-3
3-5
5+
Log10 P − value FATCAT
0
5
10
TM Score
0.25
0.50
0.75

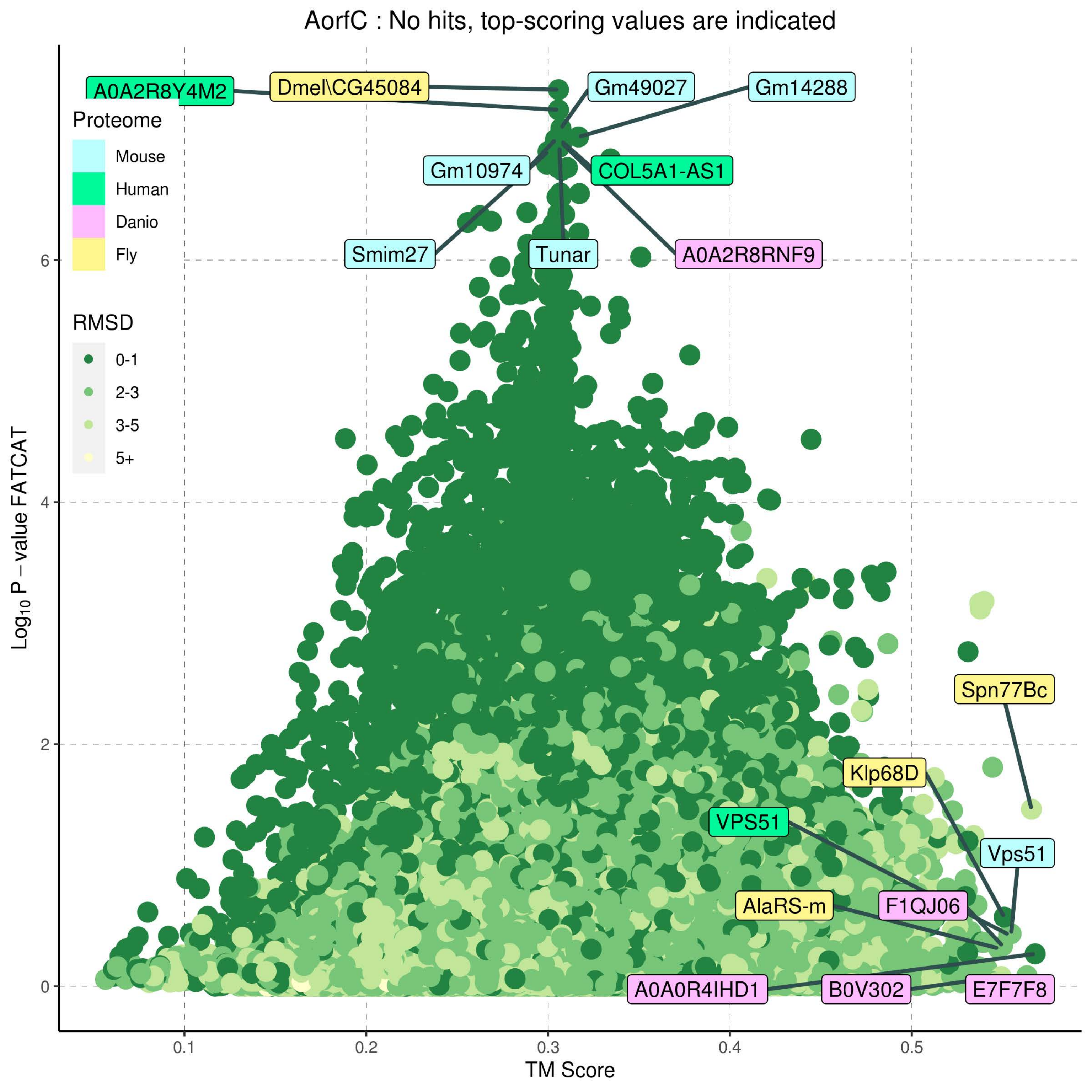
AorfC : No hits, top-scoring values are indicated
Proteome
Mouse
Human
Danio
Fly
RMSD
0-1
2-3
3-5
5+
A0A2R8Y4M2
Dmel\CG45084
Gm49027
Gm14288
Gm10974
COL5A1-AS1
Smim27
Tunar
A0A2R8RNF9
Spn77Bc
Klp68D
VPS51
Vps51
AlaRS-m
F1QJ06
A0A0R4IHD1
B0V302
E7F7F8
Log10 P – value FATCAT
TM Score
0
2
4
6
0.1
0.2
0.3
0.4
0.5

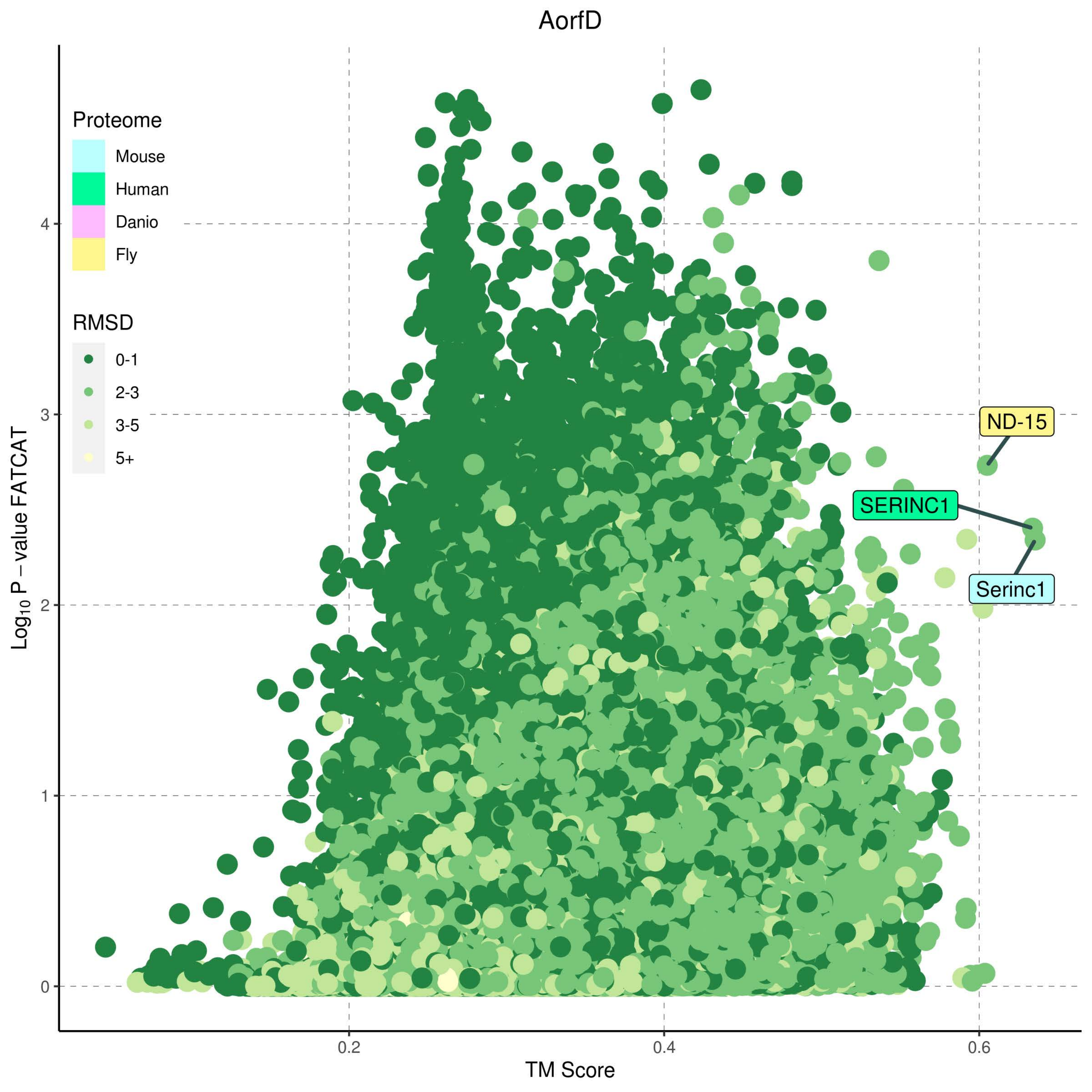
AorfD
Proteome
Mouse
Human
Danio
Fly
RMSD
0-1
2-3
3-5
5+
ND-15
SERINC1
Serinc1
Log10 P − value FATCAT
TM Score
0
1
2
3
4
0.2
0.4
0.6

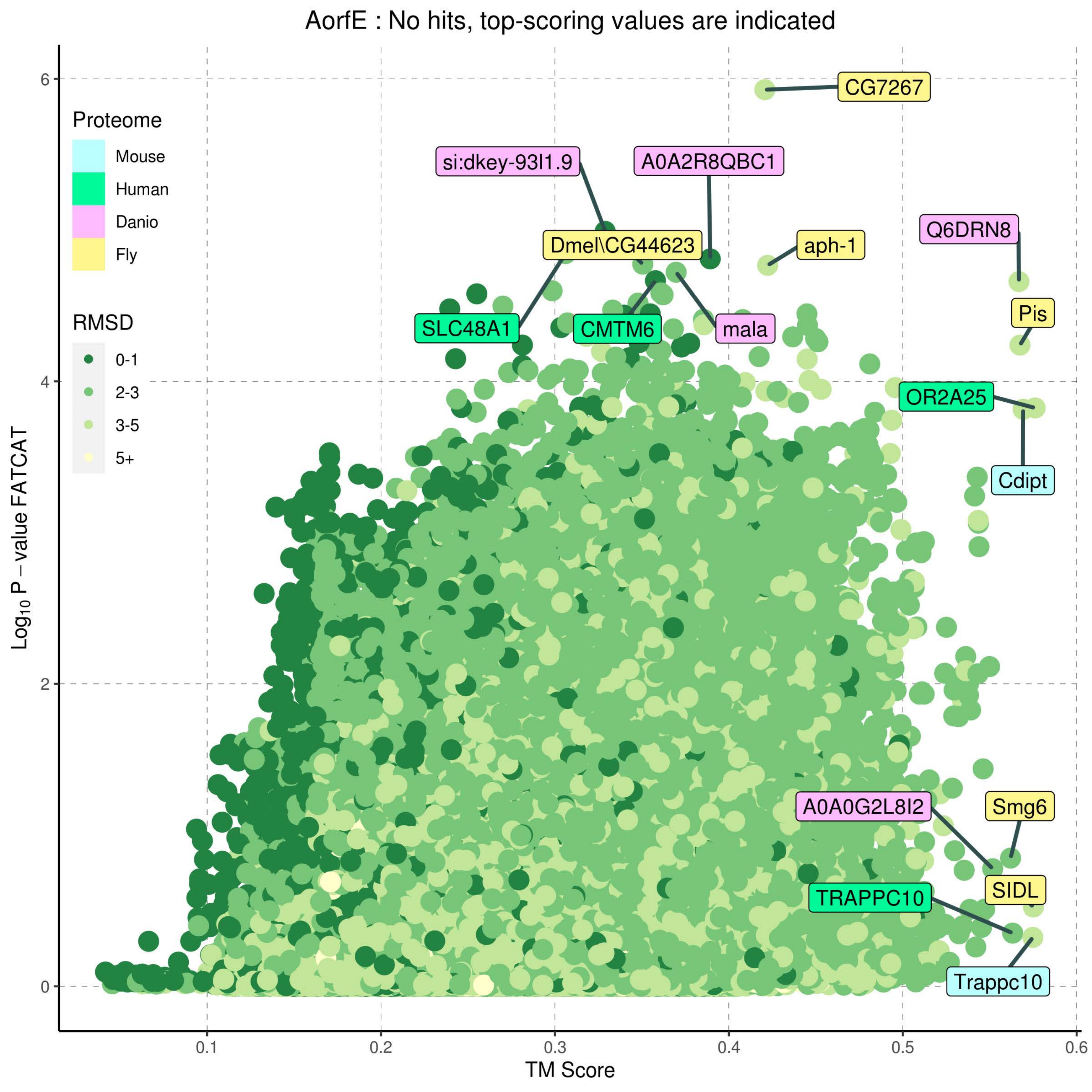
AorfE : No hits, top-scoring values are indicated
Proteome
Mouse
Human
Danio
Fly
RMSD
0-1
2-3
3-5
5+
CG7267
si:dkey-93l1.9
A0A2R8QBC1
Dmel\CG44623
aph-1
Q6DRN8
Pis
SLC48A1
CMTM6
mala
OR2A25
Cdipt
A0A0G2L8I2
Smg6
SIDL
TRAPPC10
Trappc10
Log10 P − value FATCAT
TM Score
0
2
4
6
0.1
0.2
0.3
0.4
0.5
0.6

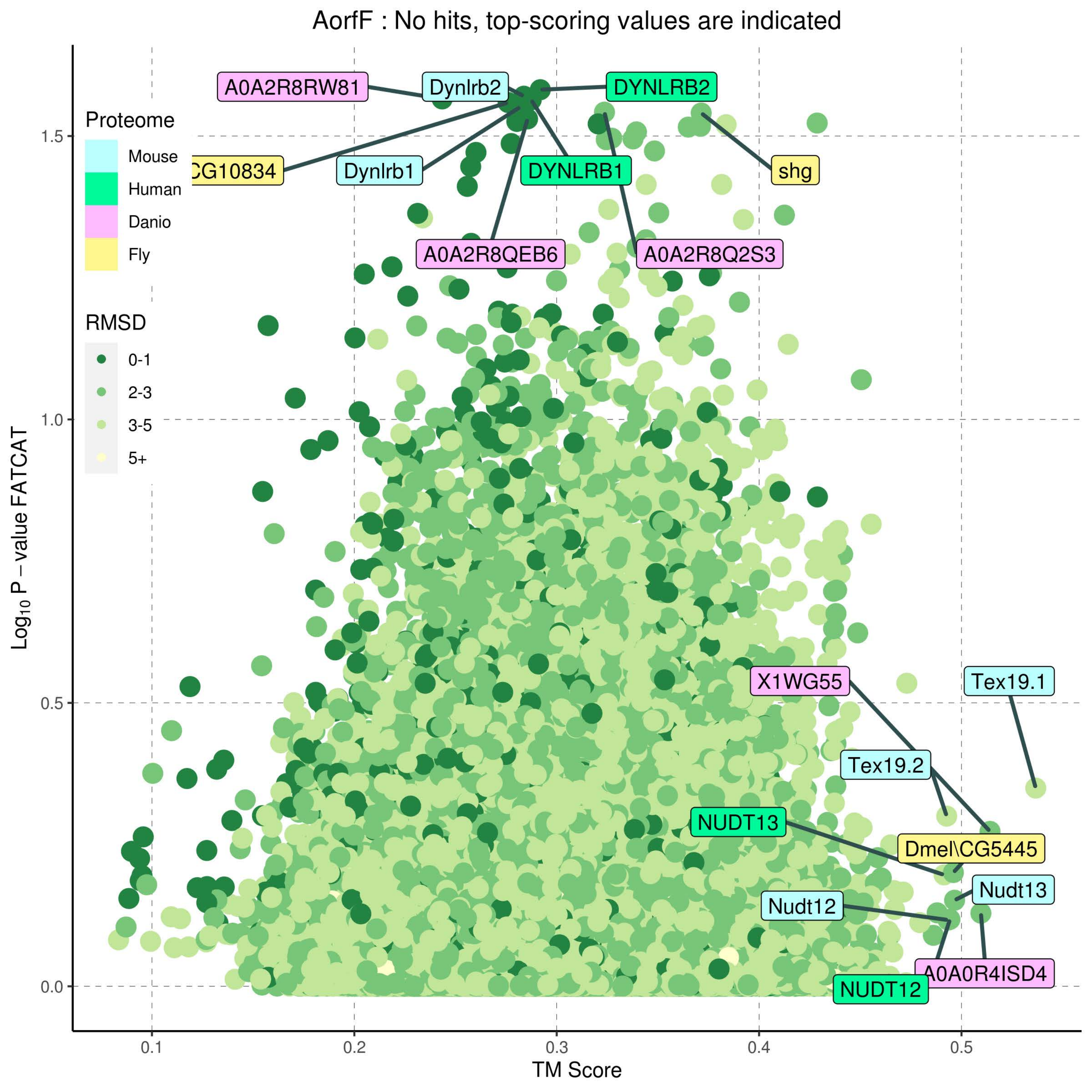
AorfF : No hits, top-scoring values are indicated
Proteome
Mouse
Human
Danio
Fly
RMSD
0-1
2-3
3-5
5+
A0A2R8RW81
Dynlrb2
DYNLRB2
CG10834
Dynlrb1
DYNLRB1
shg
A0A2R8QEB6
A0A2R8Q2S3
X1WG55
Tex19.1
Tex19.2
NUDT13
Dmel\CG5445
Nudt13
Nudt12
A0A0R4ISD4
NUDT12
Log10 P − value FATCAT
TM Score
0.0
0.5
1.0
1.5
0.1
0.2
0.3
0.4
0.5

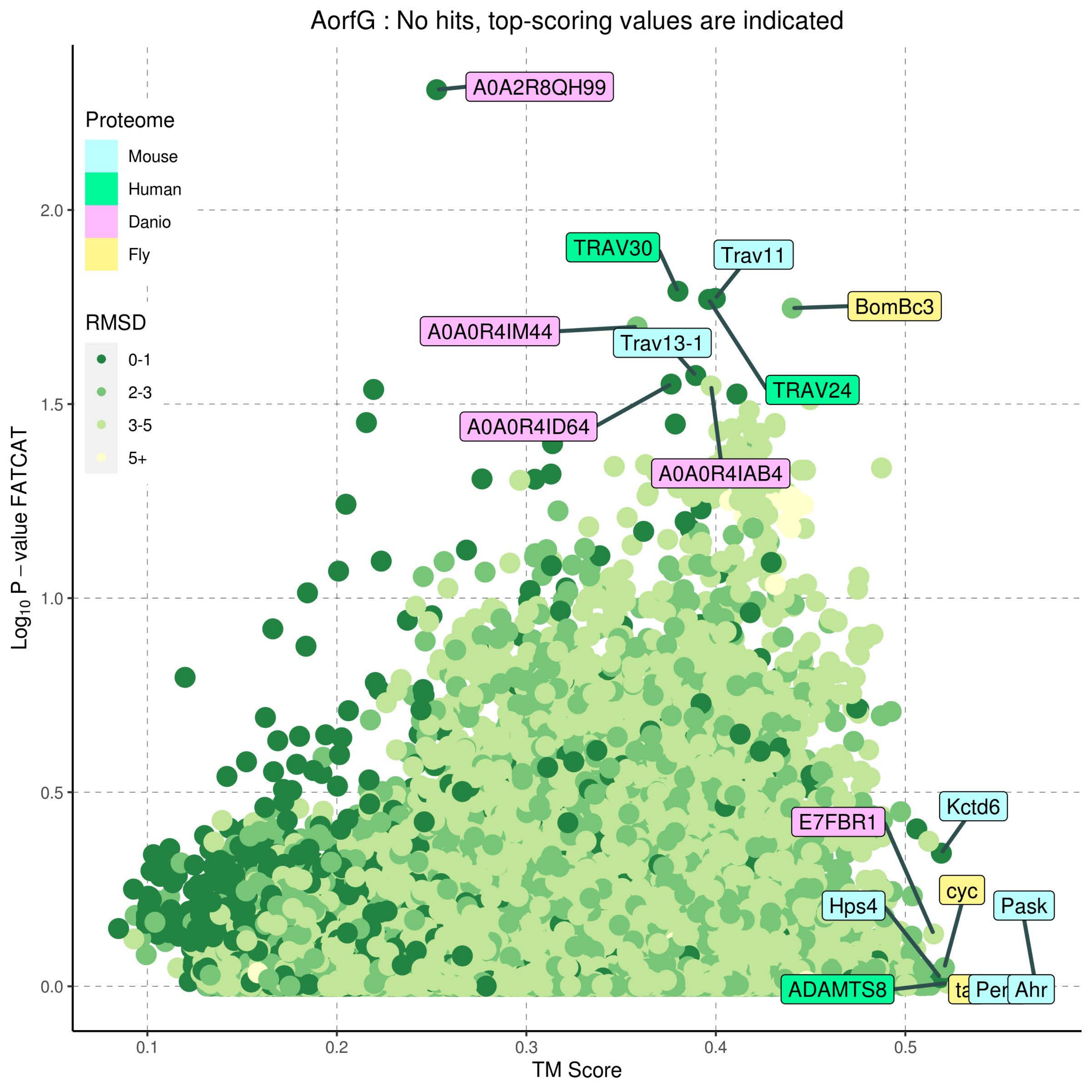

AorfG : No hits, top-scoring values are indicated
Proteome
Mouse
Human
Danio
Fly
RMSD
0-1
2-3
3-5
5+
A0A2R8QH99
TRAV30
Trav11
BomBc3
A0A0R4IM44
Trav13-1
TRAV24
A0A0R4ID64
A0A0R4IAB4
Kctd6
E7FBR1
cyc
Hps4
Pask
ADAMTS8
Ahr
Log10 P − value FATCAT
TM Score
2.0
1.5
1.0
0.5
0.0
0.1
0.2
0.3
0.4
0.5

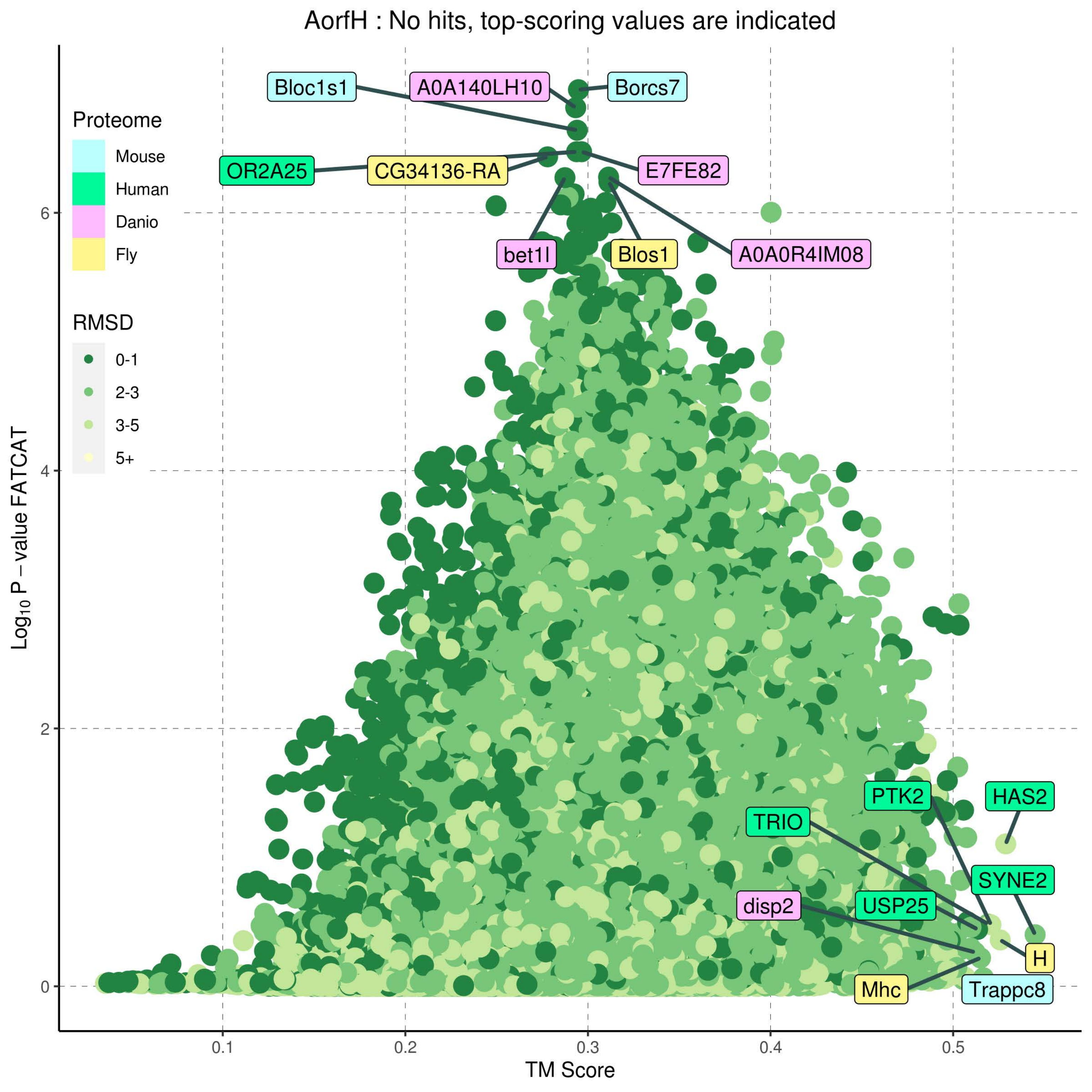

AorfH : No hits, top-scoring values are indicated
Proteome
Mouse
Human
Danio
Fly
RMSD
0-1
2-3
3-5
5+
Bloc1s1
A0A140LH10
Borcs7
OR2A25
CG34136-RA
E7FE82
bet1l
Blos1
A0A0R4IM08
PTK2
HAS2
TRIO
SYNE2
disp2
USP25
H
Mhc
Trappc8
Log10 P − value FATCAT
TM Score
0
2
4
6
0.1
0.2
0.3
0.4
0.5

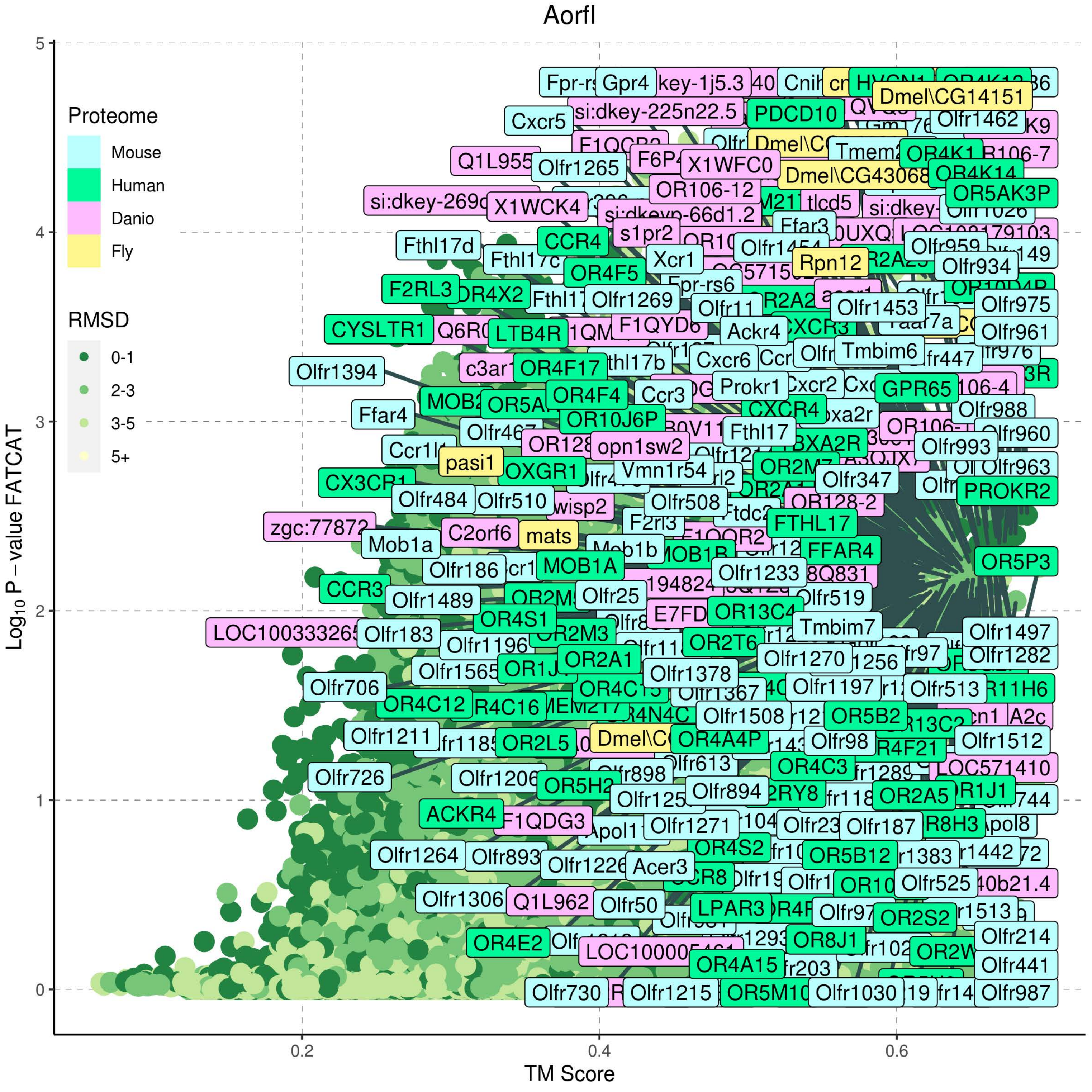

Aorfl
Proteome
Mouse
Human
Danio
Fly
RMSD
0-1
2-3
3-5
5+
Log10 P − value FATCAT
TM Score
0
1
2
3
4
5
0.2
0.4
0.6

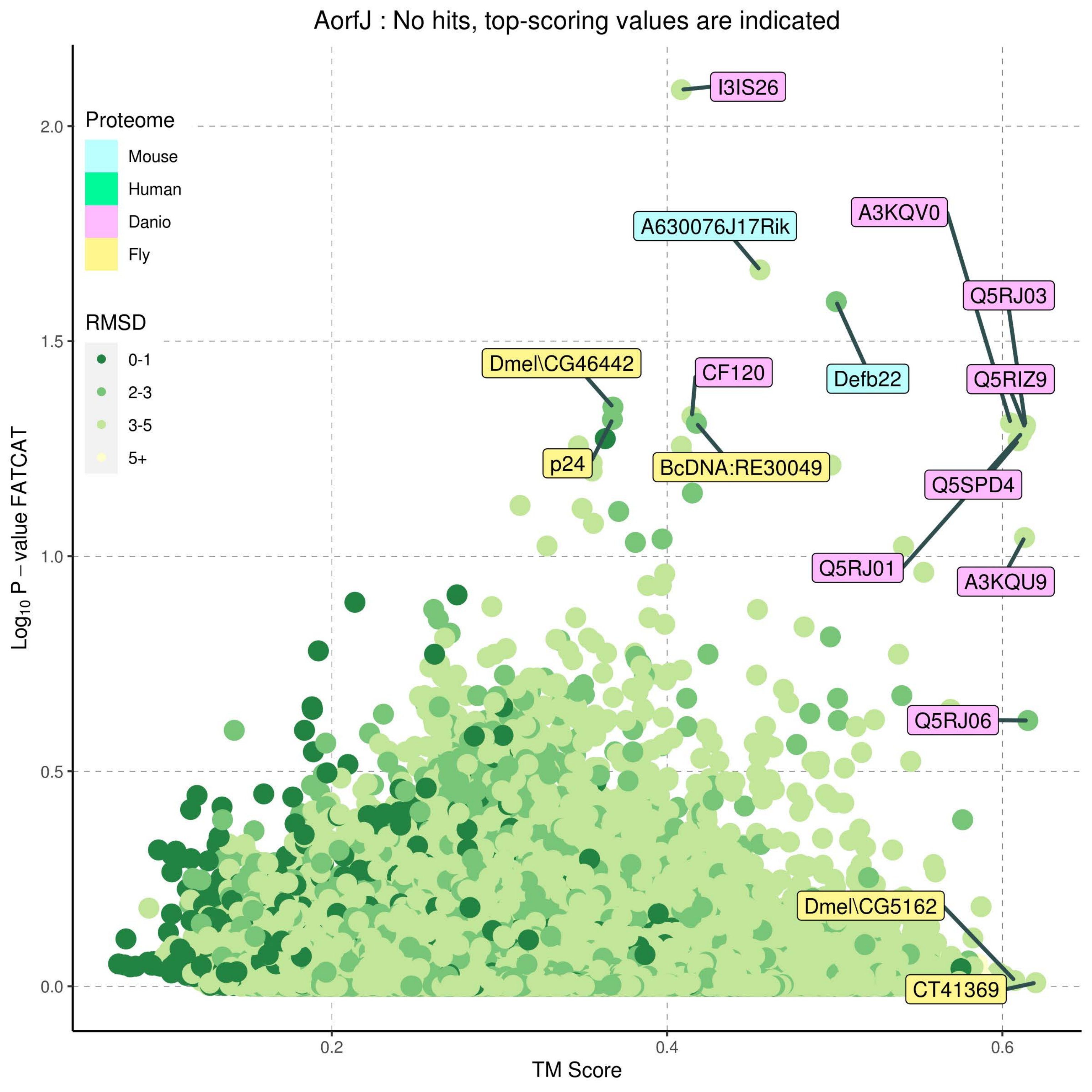

AorfJ : No hits, top-scoring values are indicated
Proteome
Mouse
Human
Danio
Fly
RMSD
0-1
2-3
3-5
5+
I3IS26
A630076J17Rik
A3KQV0
Q5RJ03
Defb22
Q5RIZ9
CF120
Dmel\CG46442
p24
BcDNA:RE30049
Q5SPD4
Q5RJ01
A3KQU9
Q5RJ06
Dmel\CG5162
CT41369
Log10 P − value FATCAT
TM Score
2.0
1.5
1.0
0.5
0.0
0.2
0.4
0.6

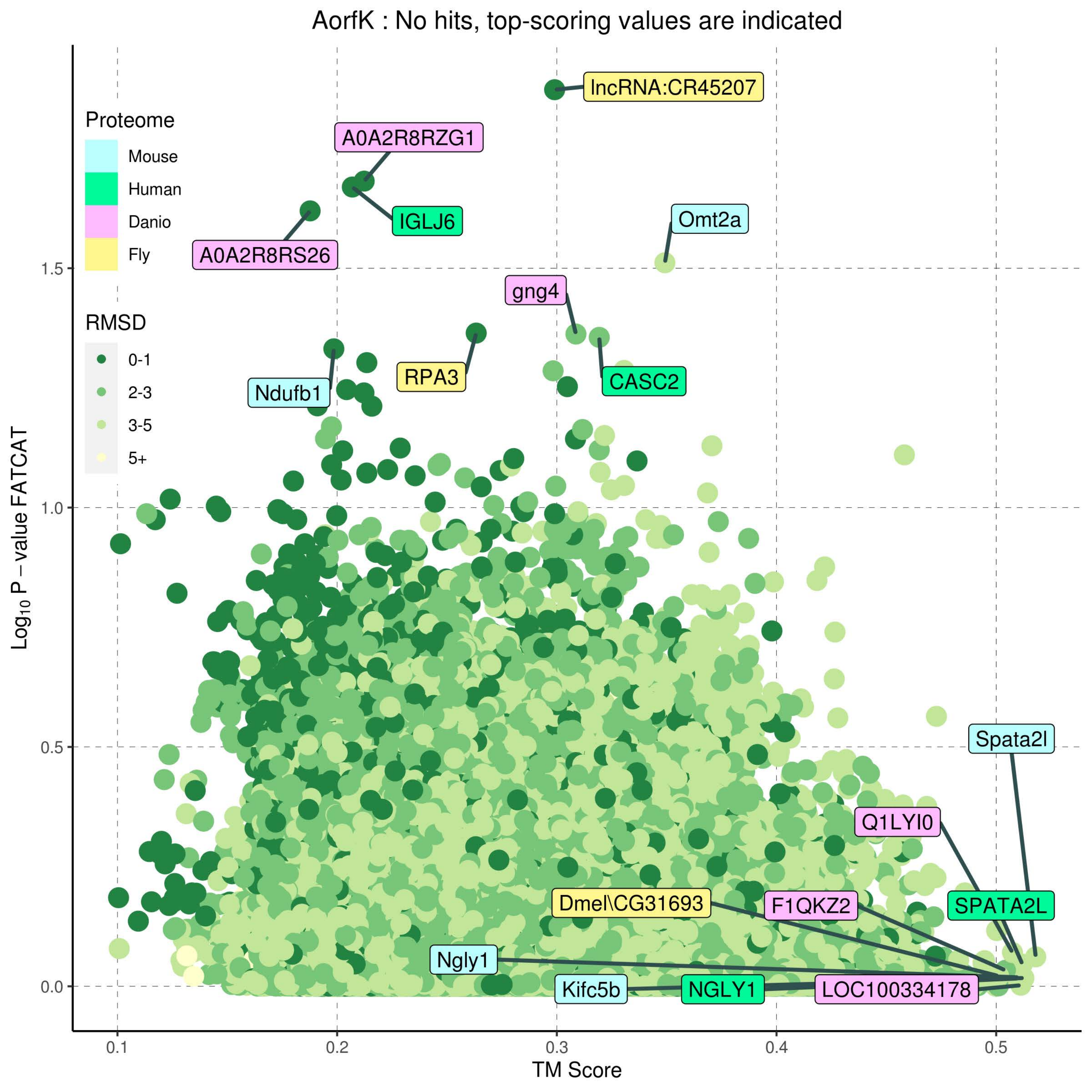
AorfK : No hits, top-scoring values are indicated
Proteome
Mouse
Human
Danio
Fly
RMSD
0-1
2-3
3-5
5+
lncRNA:CR45207
A0A2R8RZG1
IGLJ6
A0A2R8RS26
Omt2a
gng4
RPA3
Ndufb1
CASC2
Spata2l
Q1LYI0
Dmel\CG31693
F1QKZ2
SPATA2L
Ngly1
Kifc5b
NGLY1
LOC100334178
Log10 P − value FATCAT
TM Score
0.0
0.5
1.0
1.5
0.1
0.2
0.3
0.4
0.5

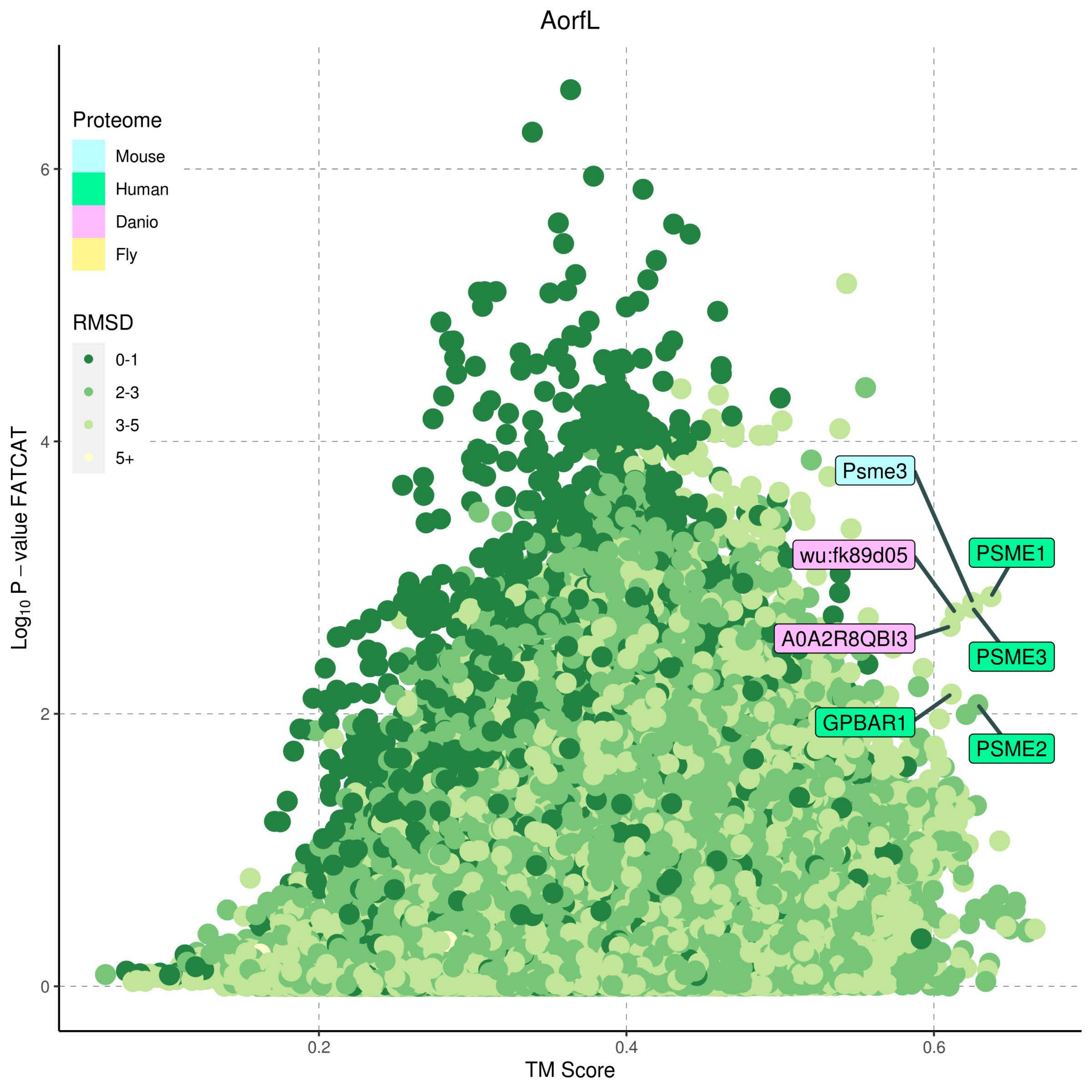

AorfL
Proteome
Mouse
Human
Danio
Fly
RMSD
0-1
2-3
3-5
5+
Psme3
wu:fk89d05
PSME1
A0A2R8QBI3
PSME3
GPBAR1
PSME2
6
4
2
0
0.2
0.4
0.6
TM Score
Log10 P − value FATCAT

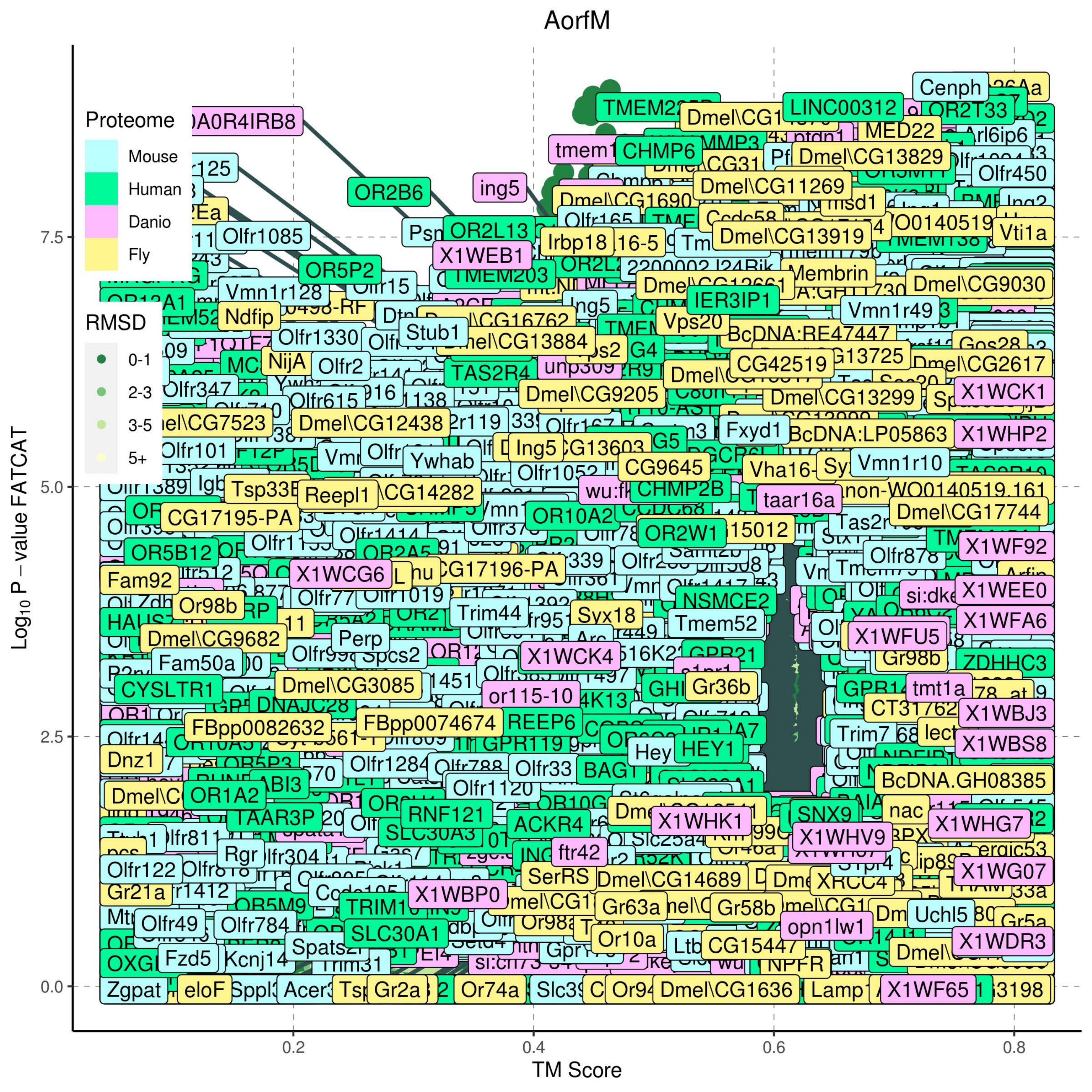

AorfM
Proteome
Mouse
Human
Danio
Fly
RMSD
0-1
2-3
3-5
5+
7.5
5.0
2.5
0.0
Log_{10} P – value FATCAT
0.2
0.4
0.6
0.8
TM Score

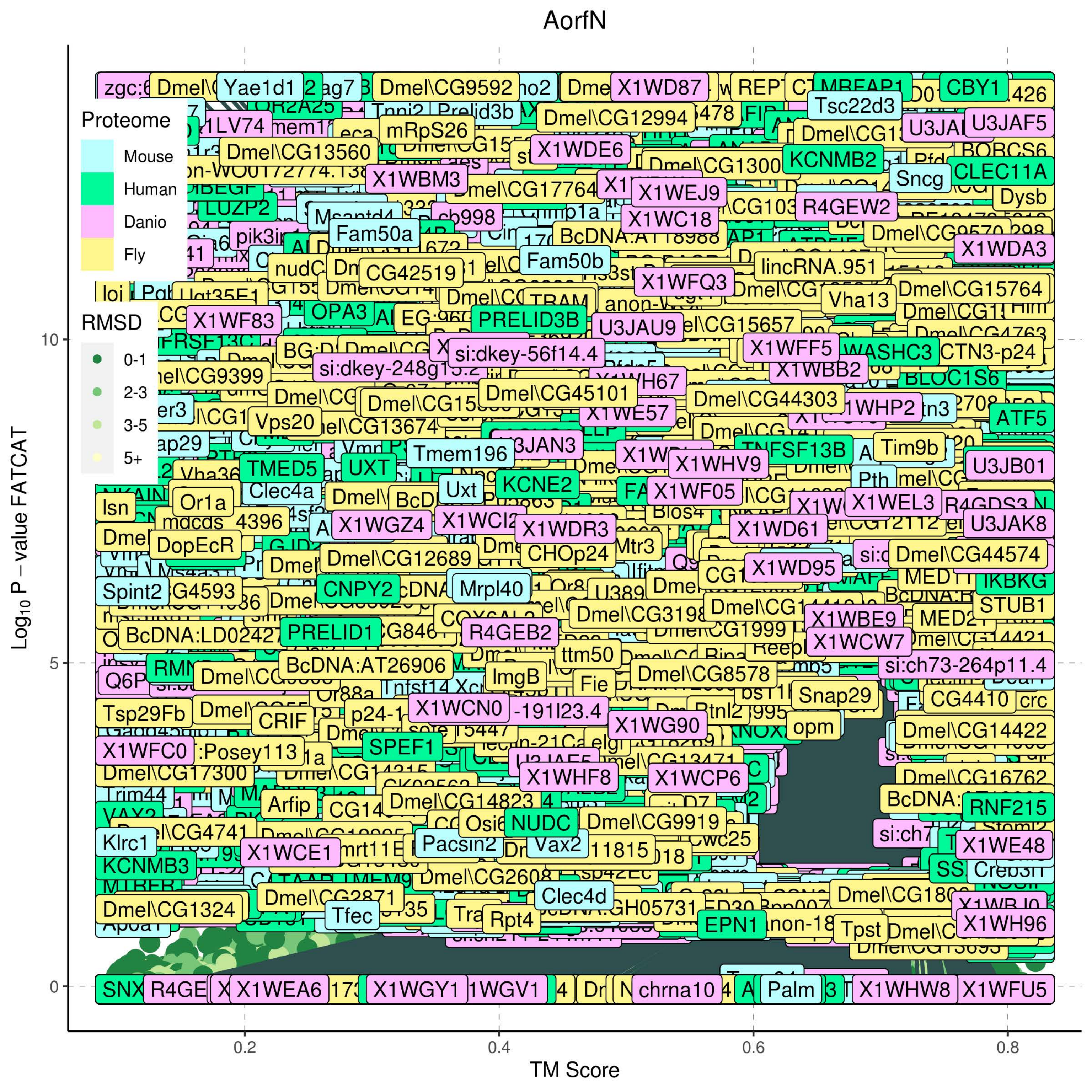
AorfN
Proteome
Mouse
Human
Danio
Fly
RMSD
0-1
2-3
3-5
5+
Log10 P – value FATCAT
TM Score
0
5
10
0.2
0.4
0.6
0.8
Yae1d1
Dmel\CG9592
X1WD87
CBY1
Tsc22d3
U3JAF5
Dmel\CG12994
mRpS26
Dmel\CG13560
X1WDE6
KCNMB2
CLEC11A
X1WBM3
Sncg
X1WEJ9
LUZP2
R4GEW2
Dysb
X1WC18
Fam50a
X1WDA3
Fam50b
lincRNA.951
CG42519
X1WFQ3
Dmel\CG15764
Vha13
OPA3
PRELID3B
X1WF83
U3JAU9
X1WFF5
si:dkey-56f14.4
WASHC3
CTN3-p24
X1WBB2
si:dkey-248g15.2
X1WH67
BLOC1S6
Dmel\CG45101
Dmel\CG44303
X1WHP2
X1WE57
ATF5
Vps20
Tmem196
U3JAN3
Tim9b
TMED5
UXT
X1WHV9
U3JB01
Uxt
KCNE2
X1WF05
Or1a
X1WEL3
Isn
X1WGZ4
X1WDR3
X1WD61
U3JAK8
DopEcR
CHOp24
Mtr3
Dmel\CG12689
Dmel\CG44574
X1WD95
Spint2
CNPY2
Mrpl40
IKBKG
STUB1
X1WBE9
PRELID1
R4GEB2
X1WCW7
BcDNA:AT26906
ttm50
Dmel\CG8578
si:ch73-264p11.4
ImgB
Fie
Snap29
Tsp29Fb
CRIF
X1WCN0
X1WG90
CG4410
Dmel\CG14422
X1WFC0
SPEF1
opm
Dmel\CG17300
X1WHF8
X1WCP6
Dmel\CG16762
Arfip
Dmel\CG14823
RNF215
NUDC
Dmel\CG9919
Klrc1
Pacsin2
Vax2
X1WE48
KCNMB3
X1WCE1
Dmel\CG2608
Clec4d
Dmel\CG1324
Dmel\CG2871
Tfec
Rpt4
EPN1
Tpst
X1WH96
X1WEA6
X1WGY1
chrna10
Palm
X1WHW8
X1WFU5

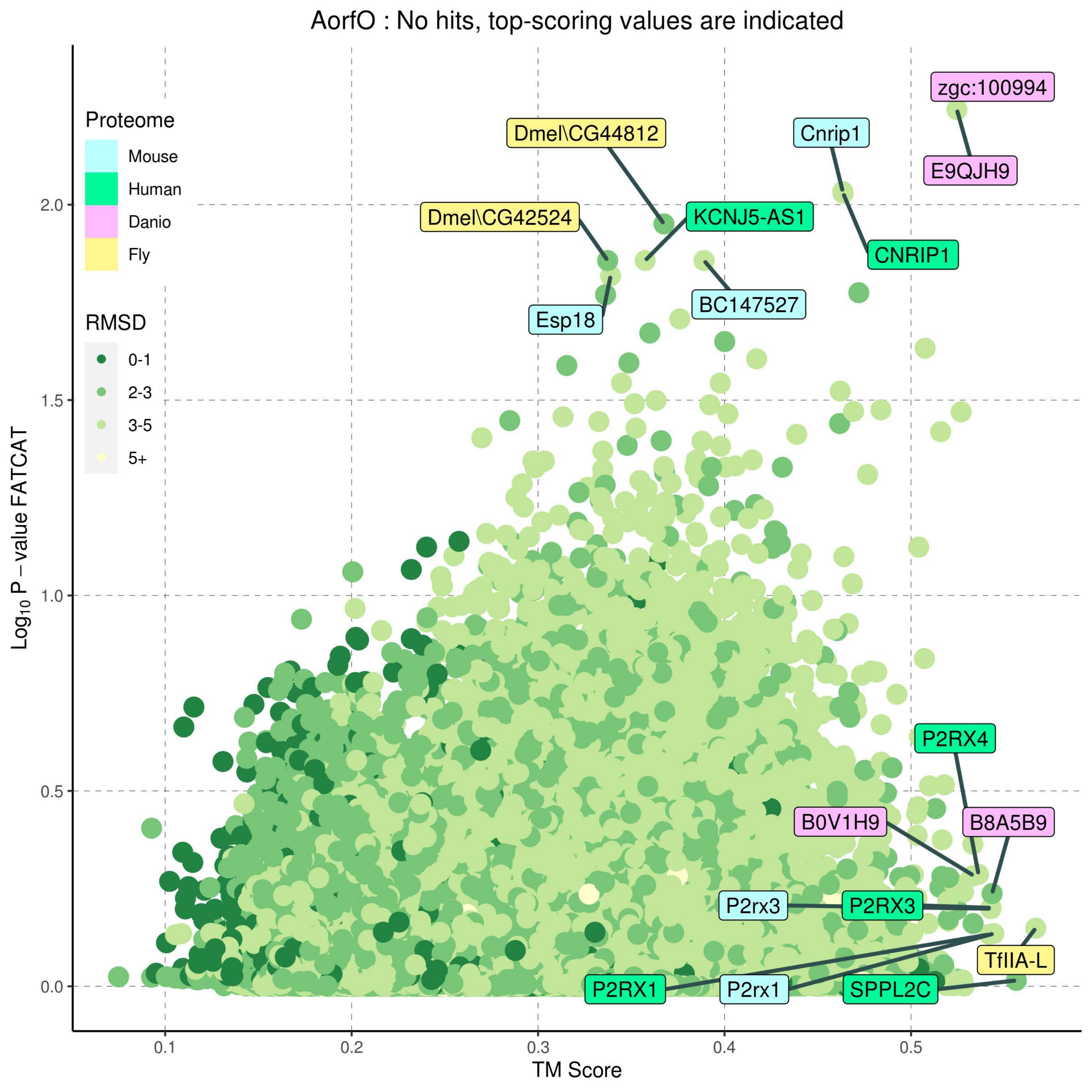
AorfO : No hits, top-scoring values are indicated
Proteome
Mouse
Human
Danio
Fly
RMSD
0-1
2-3
3-5
5+
zgc:100994
E9QJH9
Dmel\CG44812
Cnrip1
Dmel\CG42524
KCNJ5-AS1
CNRIP1
Esp18
BC147527
P2RX4
B0V1H9
B8A5B9
P2rx3
P2RX3
TfIIA-L
P2RX1
P2rx1
SPPL2C
Log10 P − value FATCAT
TM Score
0.0
0.5
1.0
1.5
2.0
0.1
0.2
0.3
0.4
0.5

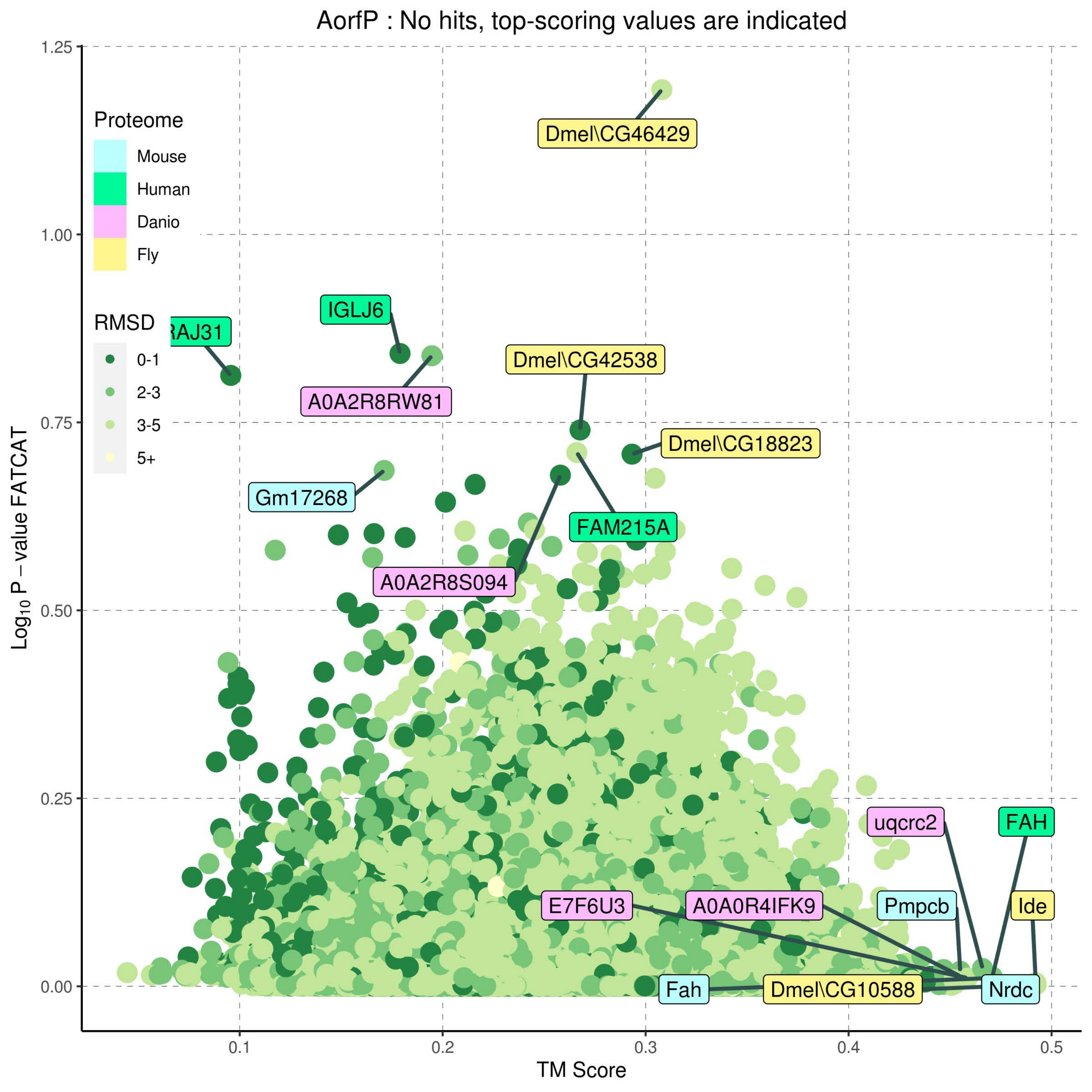
AorfP : No hits, top-scoring values are indicated
Proteome
Mouse
Human
Danio
Fly
RMSD
0-1
2-3
3-5
5+
Dmel\CG46429
IGLJ6
RAJ31
A0A2R8RW81
Dmel\CG42538
Dmel\CG18823
Gm17268
FAM215A
A0A2R8S094
uqcrc2
FAH
E7F6U3
A0A0R4IFK9
Pmpcb
Ide
Fah
Dmel\CG10588
Nrdc
Log10 P – value FATCAT
TM Score
1.25
1.00
0.75
0.50
0.25
0.00
0.1
0.2
0.3
0.4
0.5

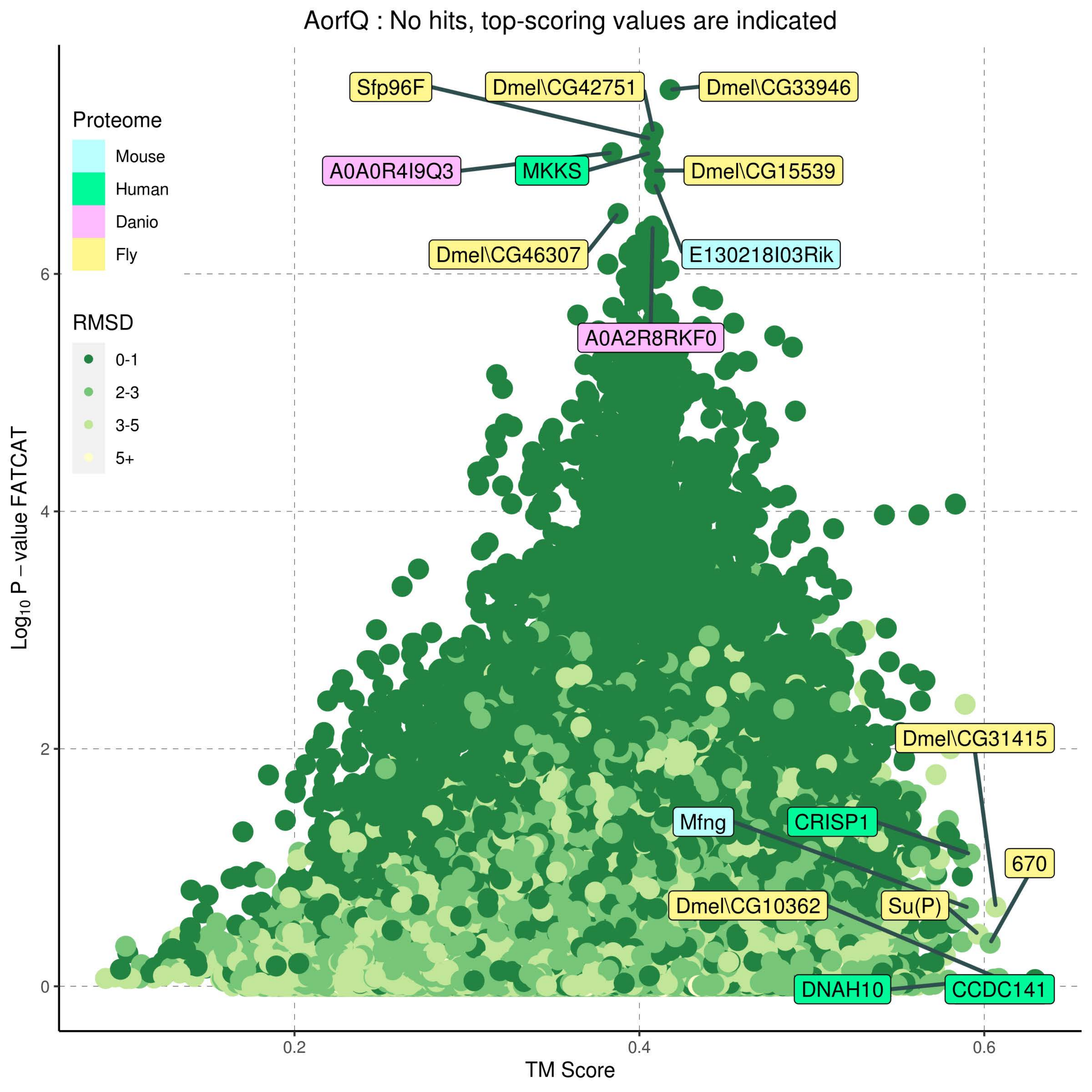

AorfQ : No hits, top-scoring values are indicated
Proteome
Mouse
Human
Danio
Fly
RMSD
0-1
2-3
3-5
5+
Sfp96F
Dmel\CG42751
Dmel\CG33946
A0A0R4I9Q3
MKKS
Dmel\CG15539
Dmel\CG46307
E130218I03Rik
A0A2R8RKF0
Dmel\CG31415
Mfng
CRISP1
670
Dmel\CG10362
Su(P)
DNAH10
CCDC141
Log10 P − value FATCAT
TM Score
0
2
4
6
0.2
0.4
0.6

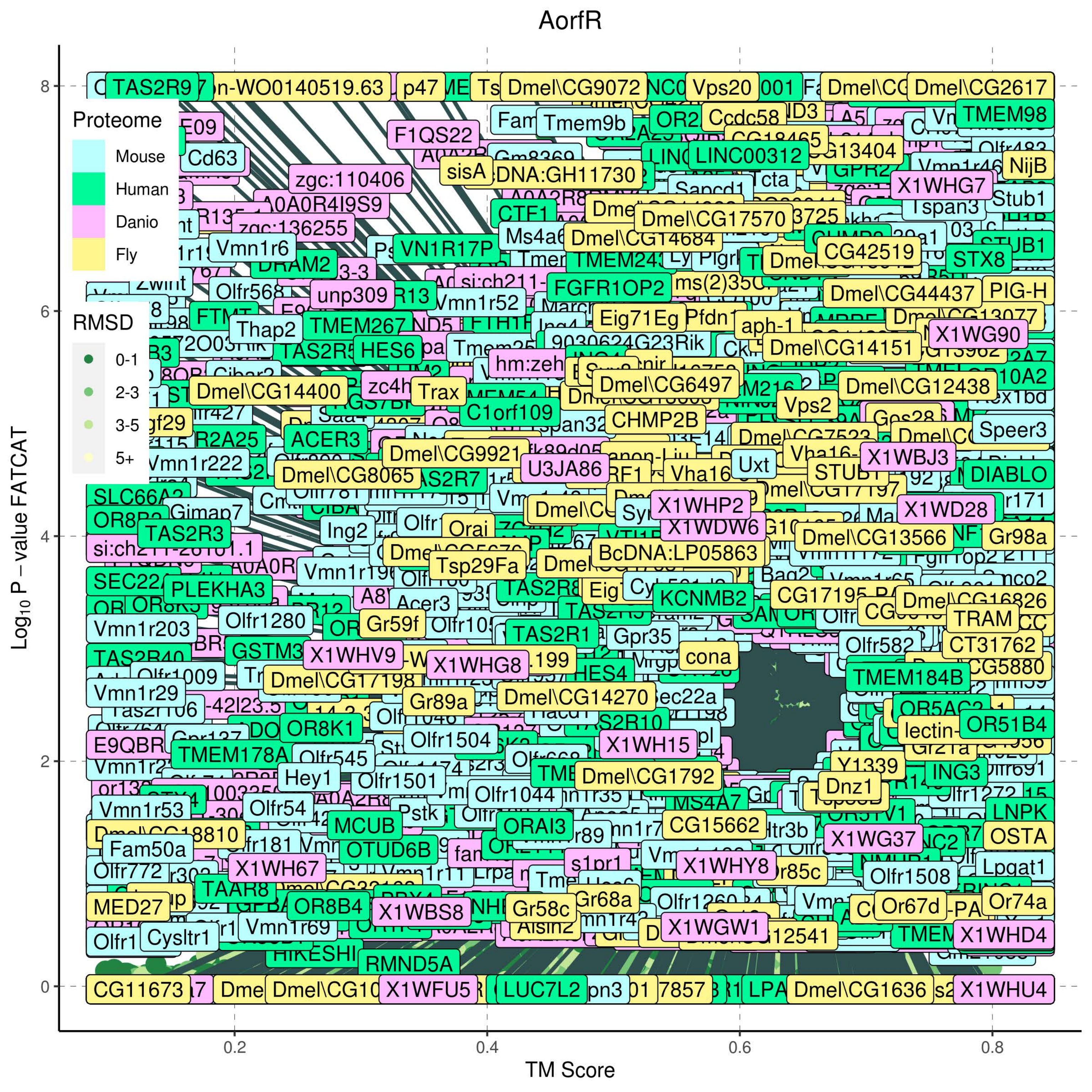

AorfR
Proteome
Mouse
Human
Danio
Fly
RMSD
0-1
2-3
3-5
5+
Log10 P − value FATCAT
TM Score
0
2
4
6
8
0.2
0.4
0.6
0.8
TAS2R9
p47
Dmel\CG9072
Vps20
Dmel\CG2617
TMEM98
Tmem9b
Ccdc58
F1QS22
Cd63
LINC00312
NijB
X1WHG7
zgc:110406
sisA
Stub1
Dmel\CG17570
Dmel\CG14684
CG42519
STX8
Vmn1r6
VN1R17P
FGFR1OP2
Dmel\CG44437
PIG-H
unp309
Vmn1r52
Olfr568
Thap2
TMEM267
HES6
Eig71Eg
X1WG90
Dmel\CG14151
Dmel\CG14400
Trax
Dmel\CG6497
Dmel\CG12438
C1orf109
CHMP2B
Vps2
Speer3
ACER3
Dmel\CG9921
U3JA86
Uxt
X1WBJ3
DIABLO
Dmel\CG8065
SLC66A2
X1WHP2
X1WD28
TAS2R3
Ing2
X1WDW6
Dmel\CG13566
Gr98a
BcDNA:LP05863
Tsp29Fa
PLEKHA3
KCNMB2
Dmel\CG16826
Vmn1r203
Olfr1280
Gr59f
TRAM
TAS2R1
Gpr35
Olfr582
CT31762
X1WHV9
X1WHG8
cona
TMEM184B
Dmel\CG17198
Vmn1r29
Gr89a
Dmel\CG14270
OR8K1
OR51B4
E9QBR
Olfr1504
X1WH15
TMEM178A
Hey1
Olfr1501
Dmel\CG1792
Y1339
ING3
Dnz1
Vmn1r53
Olfr54
Olfr1044
MCUB
ORAI3
CG15662
LNPK
OSTA
X1WG37
Fam50a
OTUD6B
X1WH67
X1WHY8
Olfr772
Olfr1508
Lpgat1
TAAR8
MED27
OR8B4
X1WBS8
Gr58c
Gr68a
Or67d
Or74a
Cysltr1
Vmn1r69
X1WGW1
X1WHD4
HIKESHI
RMND5A
CG11673
X1WFU5
LUC7L2
Dmel\CG1636
X1WHU4

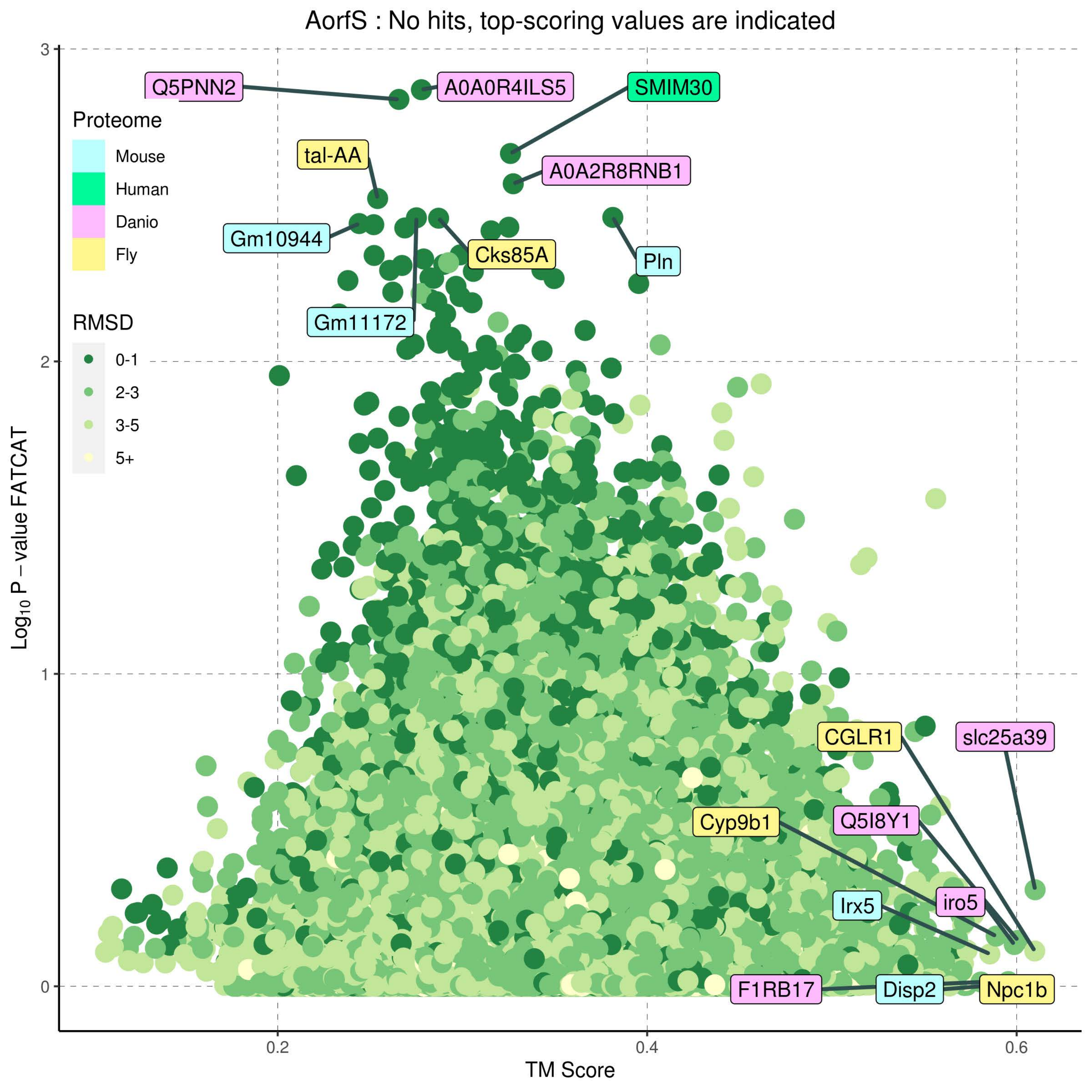

AorfS : No hits, top-scoring values are indicated
Proteome
Mouse
Human
Danio
Fly
RMSD
0-1
2-3
3-5
5+
Q5PNN2
A0A0R4ILS5
SMIM30
tal-AA
A0A2R8RNB1
Gm10944
Cks85A
Pln
Gm11172
CGLR1
slc25a39
Cyp9b1
Q5I8Y1
iro5
Irx5
F1RB17
Disp2
Npc1b
Log_{10} P − value FATCAT
TM Score
0
1
2
3
0.2
0.4
0.6

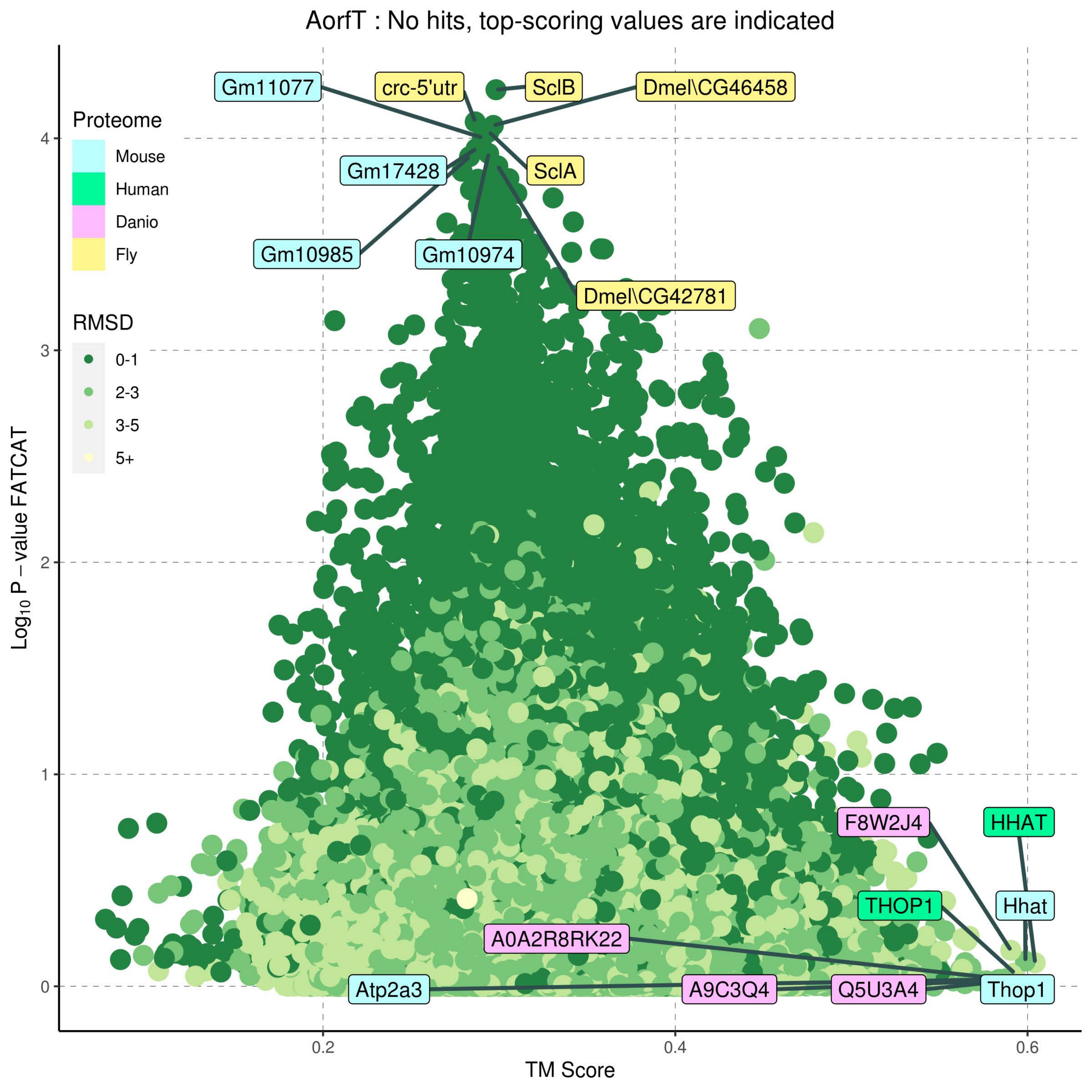

AorfT : No hits, top-scoring values are indicated
Proteome
Mouse
Human
Danio
Fly
RMSD
0-1
2-3
3-5
5+
Gm11077
crc-5'utr
SclB
Dmel\CG46458
Gm17428
SclA
Gm10985
Gm10974
Dmel\CG42781
F8W2J4
HHAT
THOP1
Hhat
A0A2R8RK22
Atp2a3
A9C3Q4
Q5U3A4
Thop1
0
1
2
3
4
0.2
0.4
0.6
Log10 P − value FATCAT
TM Score

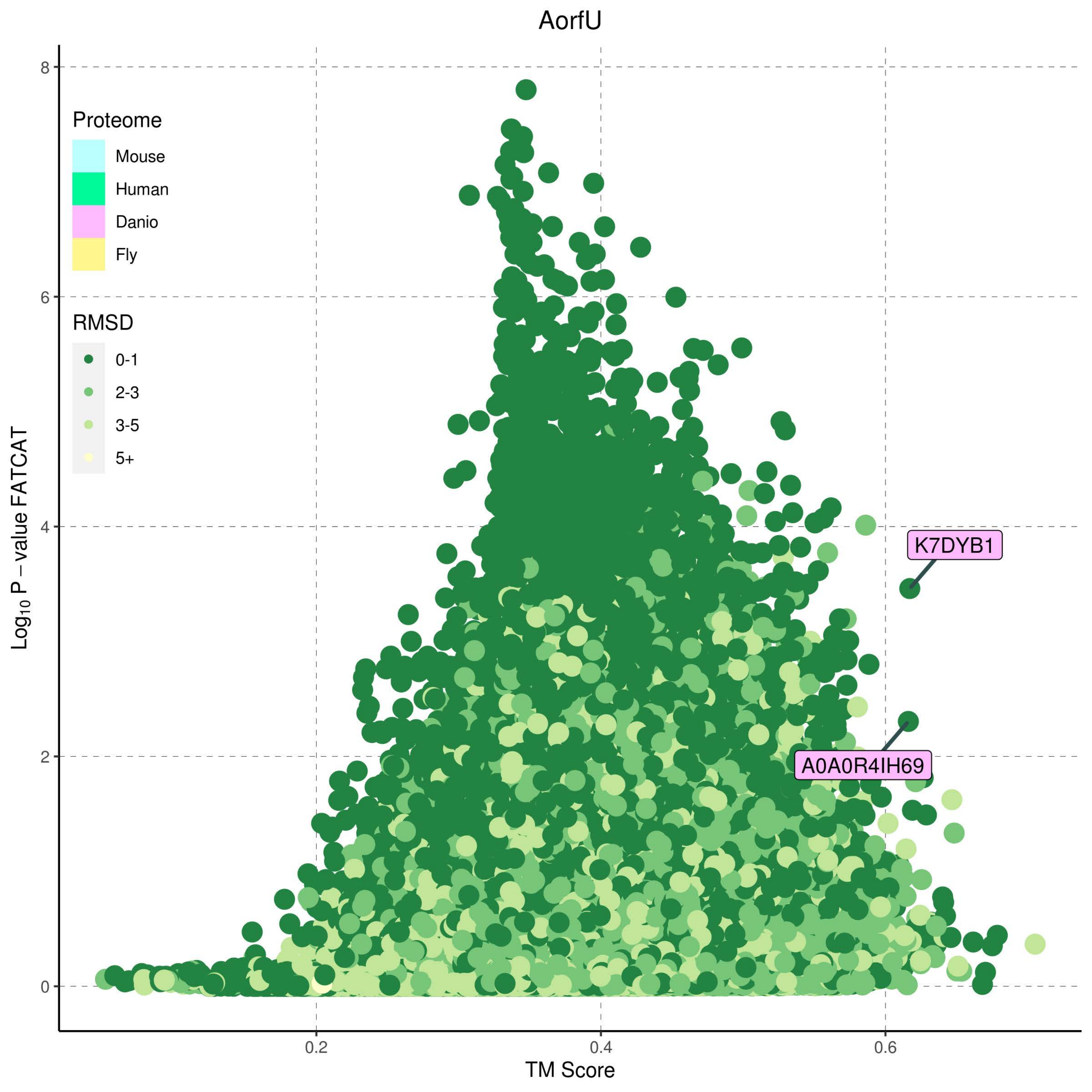

AorfU
Proteome
Mouse
Human
Danio
Fly
RMSD
0-1
2-3
3-5
5+
K7DYB1
A0A0R4IH69
Log₁₀ P − value FATCAT
TM Score
0
2
4
6
8
0.2
0.4
0.6

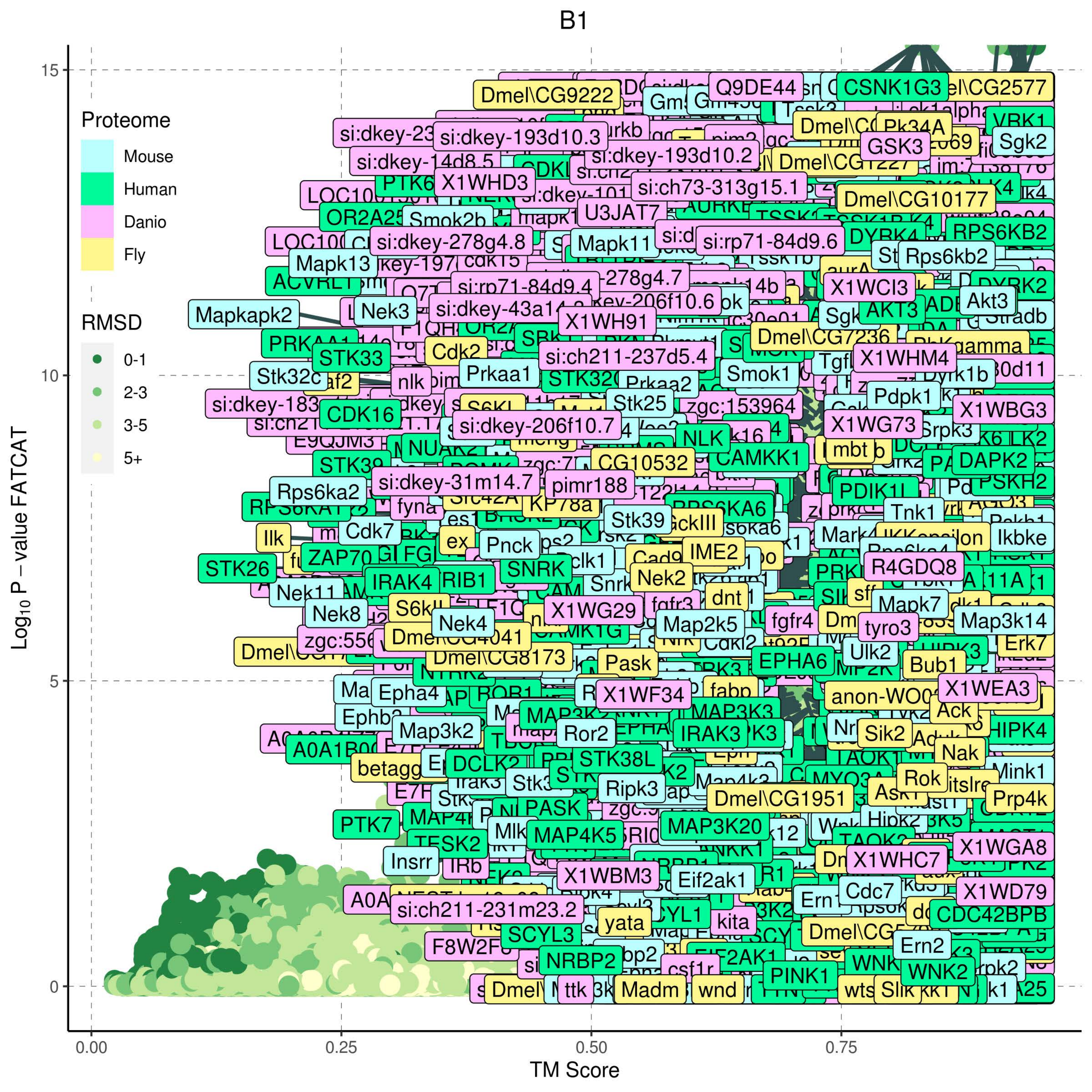
B1
Proteome
Mouse
Human
Danio
Fly
RMSD
0-1
2-3
3-5
5+
Log_{10} P − value FATCAT
15
10
5
0
0.00
0.25
0.50
0.75
TM Score

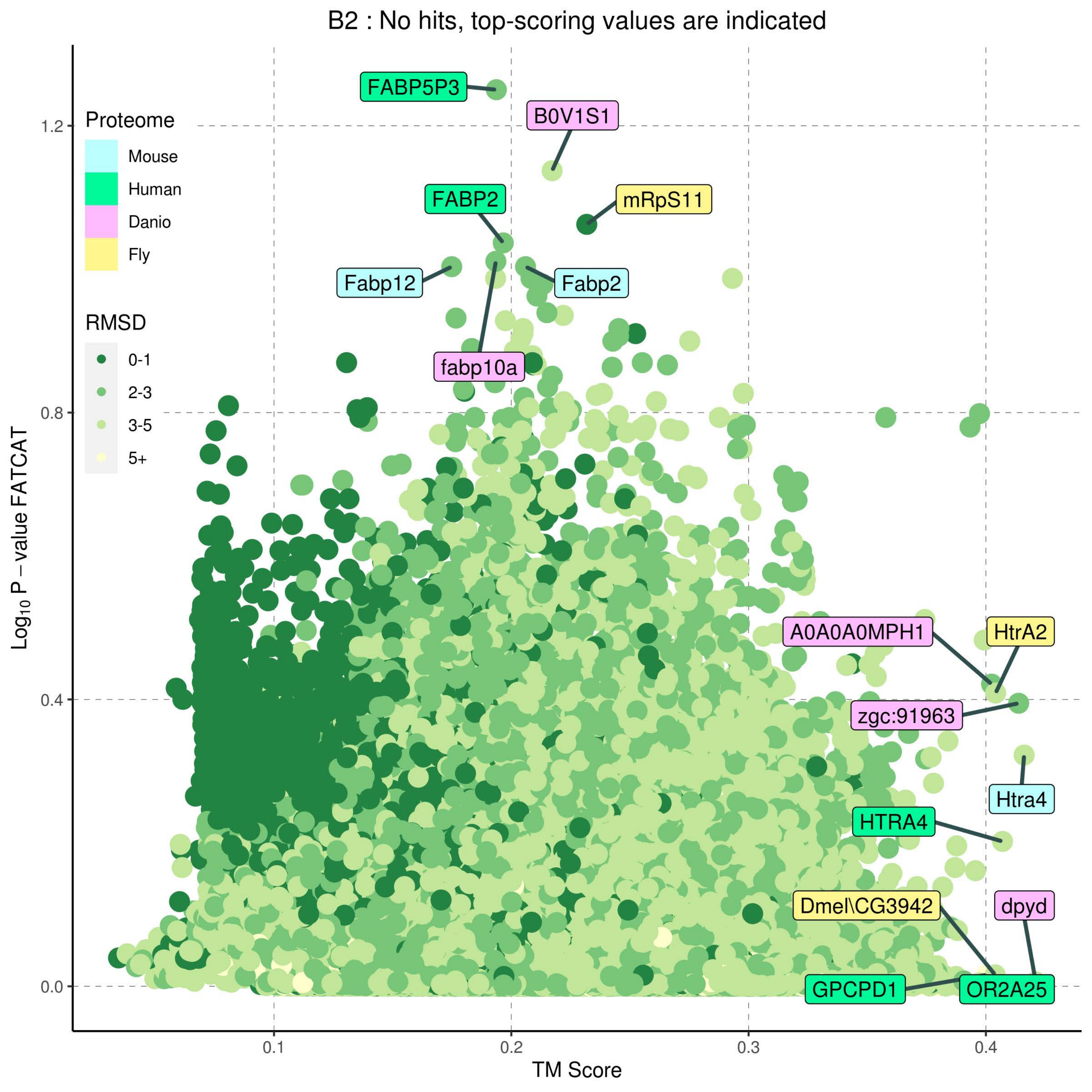

B2 : No hits, top-scoring values are indicated
Proteome
Mouse
Human
Danio
Fly
RMSD
0-1
2-3
3-5
5+
FABP5P3
B0V1S1
FABP2
mRpS11
Fabp12
Fabp2
fabp10a
A0A0A0MPH1
HtrA2
zgc:91963
Htra4
HTRA4
Dmel\CG3942
dpyd
GPCPD1
OR2A25
1.2
0.8
0.4
0.0
0.1
0.2
0.3
0.4
Log10 P – value FATCAT
TM Score

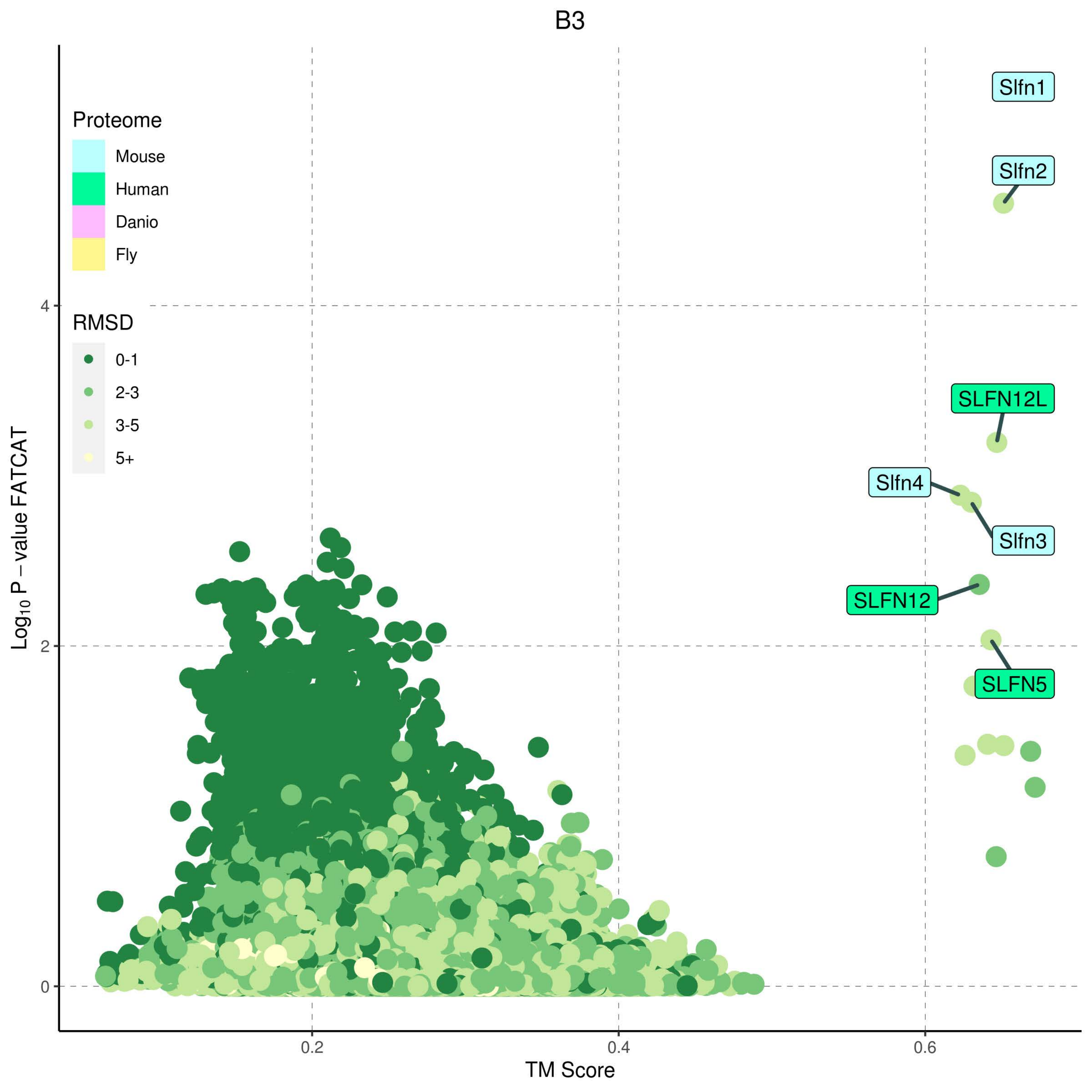

B3
Proteome
Mouse
Human
Danio
Fly
RMSD
0-1
2-3
3-5
5+
Slfn1
Slfn2
SLFN12L
Slfn4
Slfn3
SLFN12
SLFN5
Log10 P − value FATCAT
TM Score
0
2
4
0.2
0.4
0.6

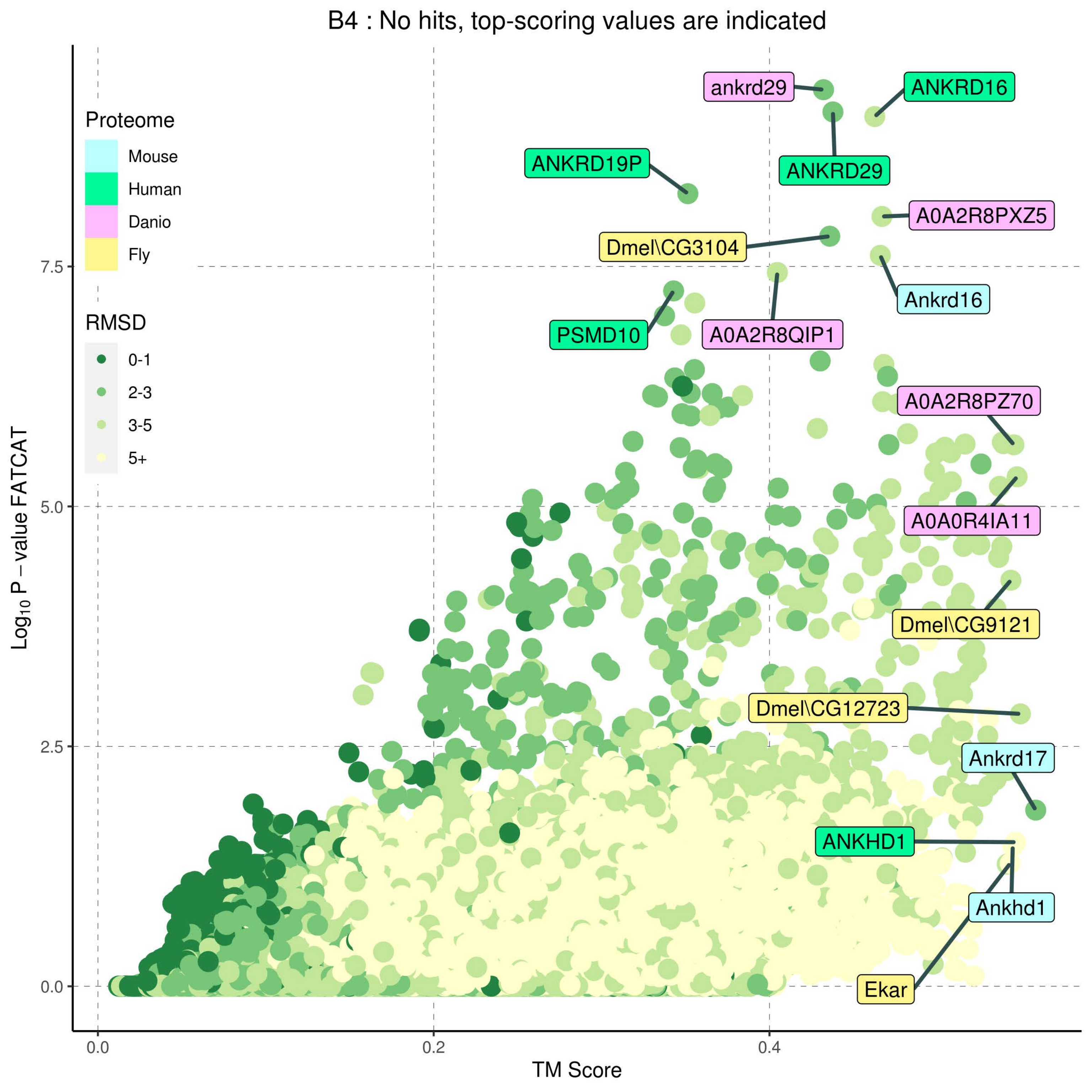
B4 : No hits, top-scoring values are indicated
Proteome
Mouse
Human
Danio
Fly
RMSD
0-1
2-3
3-5
5+
ankrd29
ANKRD16
ANKRD29
ANKRD19P
A0A2R8PXZ5
Dmel\CG3104
Ankrd16
PSMD10
A0A2R8QIP1
A0A2R8PZ70
A0A0R4IA11
Dmel\CG9121
Dmel\CG12723
Ankrd17
ANKHD1
Ankhd1
Ekar
7.5
5.0
2.5
0.0
0.0
0.2
0.4
Log10 P − value FATCAT
TM Score

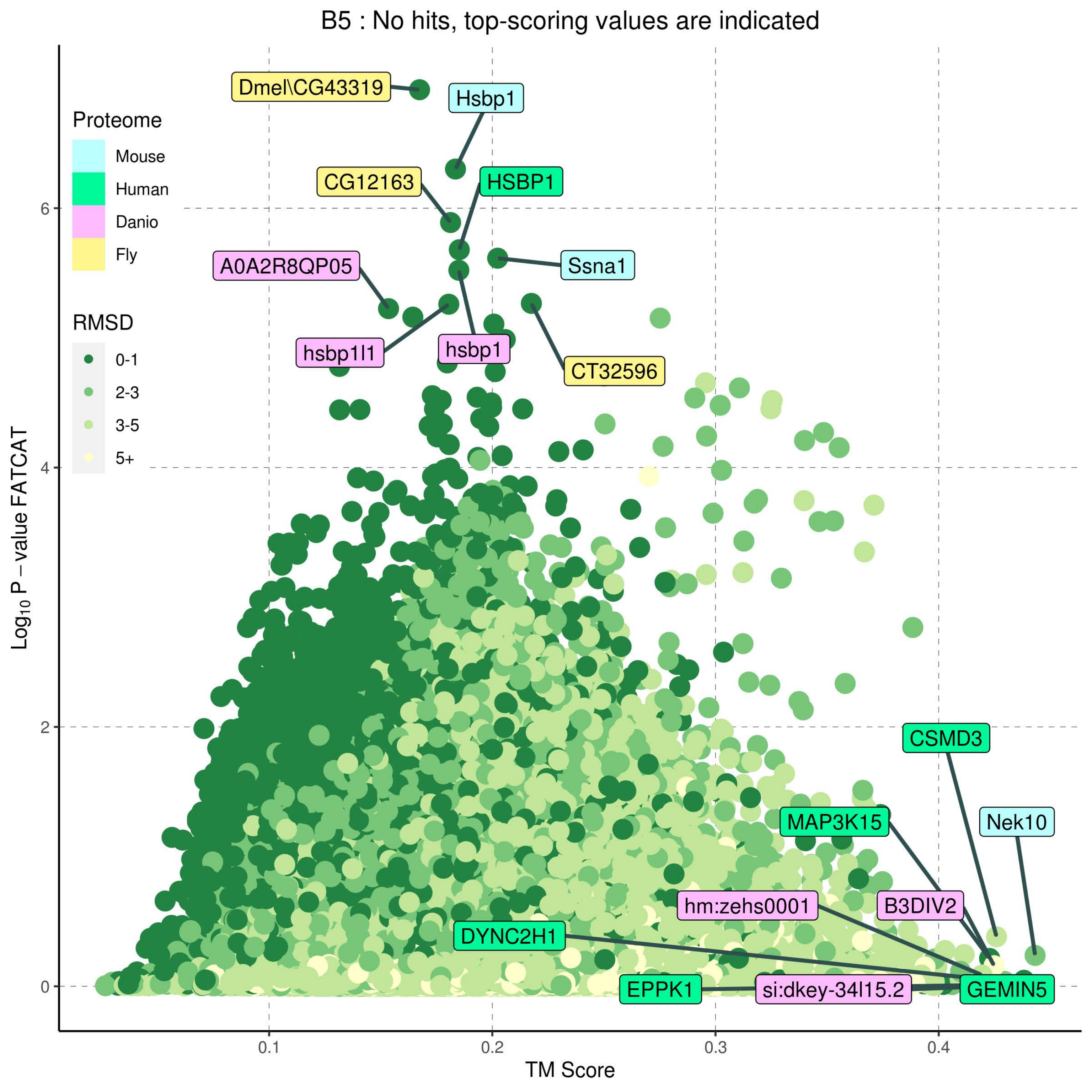

B5 : No hits, top-scoring values are indicated
Proteome
Mouse
Human
Danio
Fly
RMSD
0-1
2-3
3-5
5+
Dmel\CG43319
Hsbp1
CG12163
HSBP1
A0A2R8QP05
Ssna1
hsbp1l1
hsbp1
CT32596
CSMD3
MAP3K15
Nek10
hm:zehs0001
B3DIV2
DYNC2H1
EPPK1
si:dkey-34l15.2
GEMIN5
Log10 P − value FATCAT
TM Score
0
2
4
6
0.1
0.2
0.3
0.4

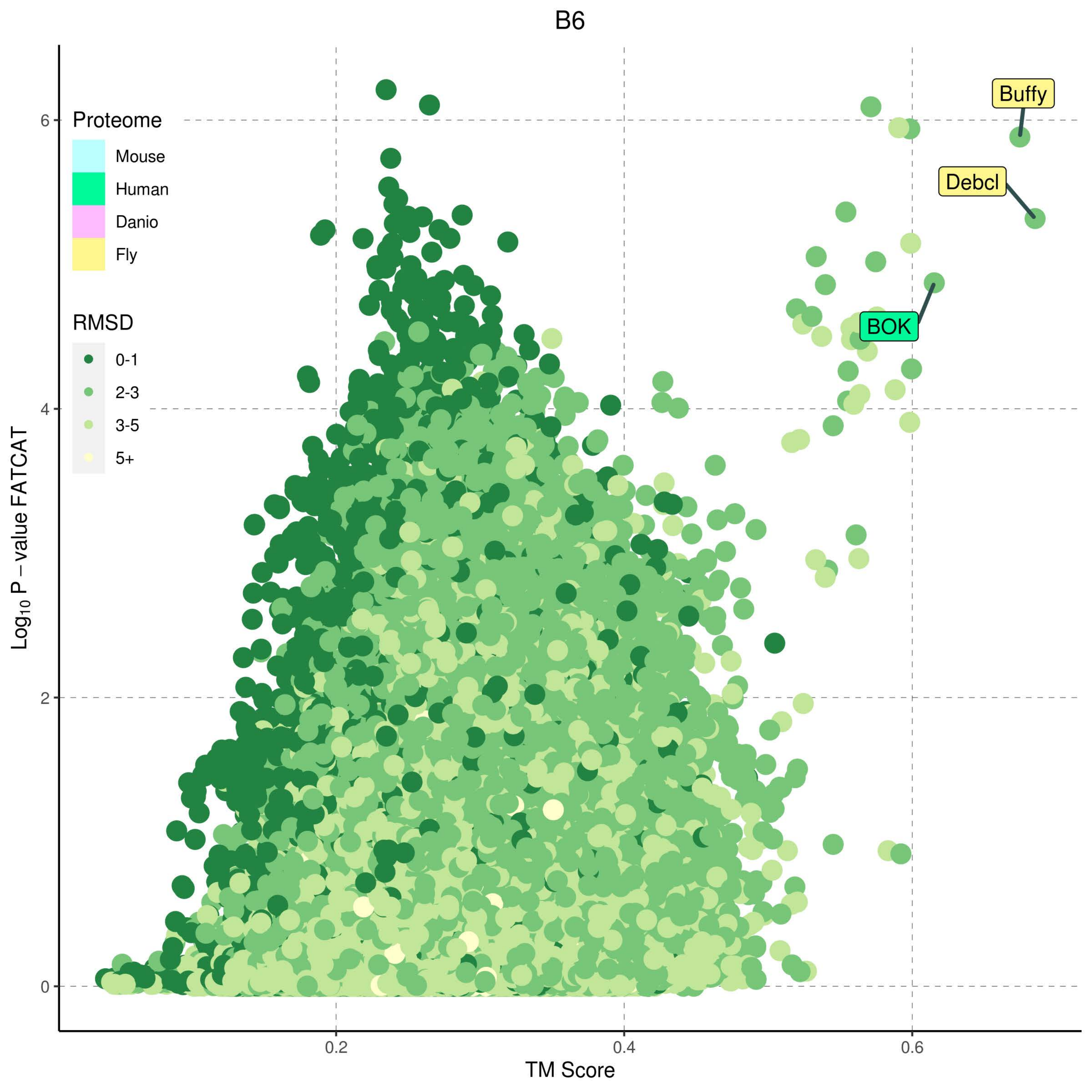
B6
Proteome
Mouse
Human
Danio
Fly
RMSD
0-1
2-3
3-5
5+
Buffy
Debcl
BOK
6
4
2
0
Log10 P − value FATCAT
0.2
0.4
0.6
TM Score

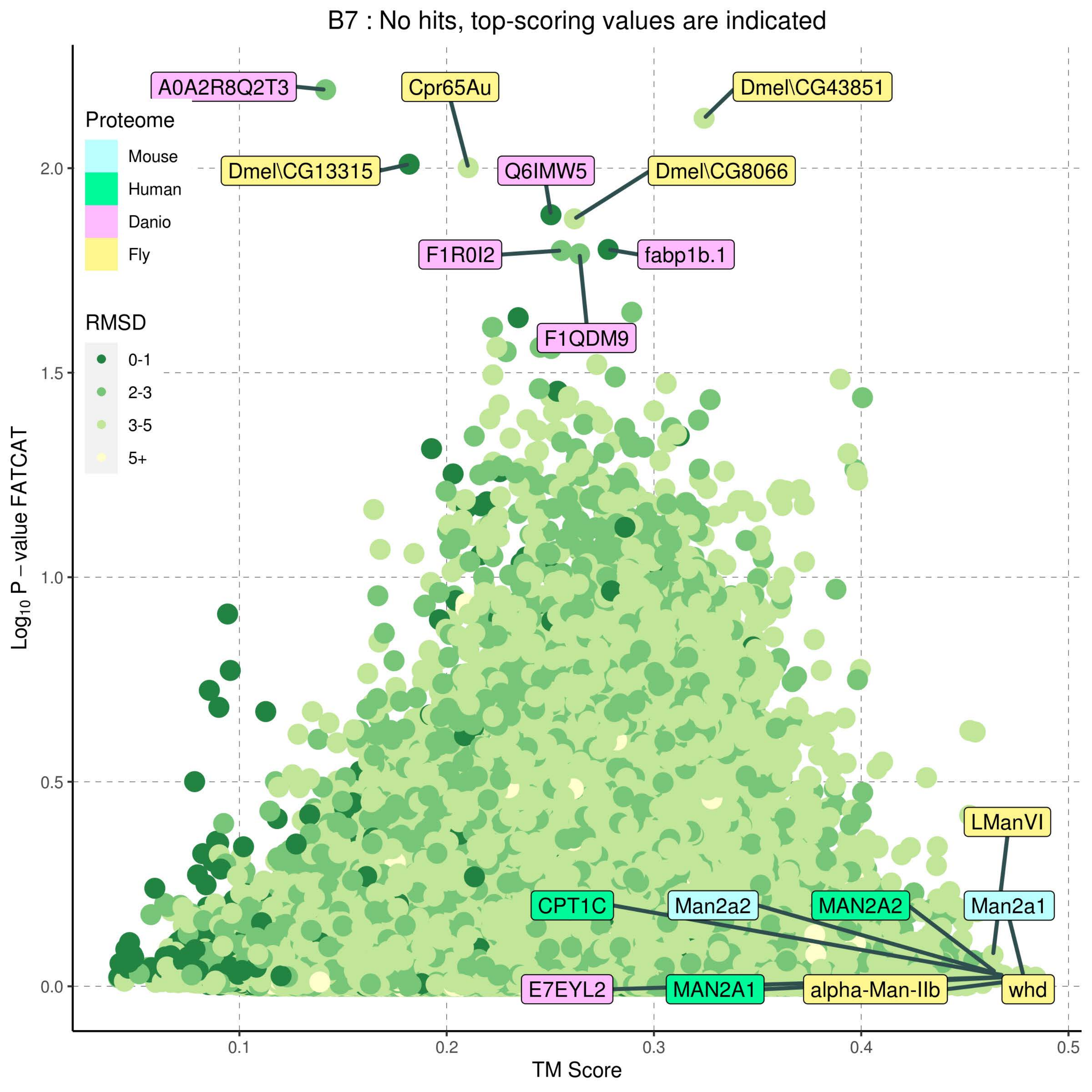

B7 : No hits, top-scoring values are indicated
Proteome
Mouse
Human
Danio
Fly
RMSD
0-1
2-3
3-5
5+
A0A2R8Q2T3
Cpr65Au
Dmel\CG43851
Dmel\CG13315
Q6IMW5
Dmel\CG8066
F1R0I2
fabp1b.1
F1QDM9
LManVI
CPT1C
Man2a2
MAN2A2
Man2a1
E7EYL2
MAN2A1
alpha-Man-IIb
whd
Log10 P – value FATCAT
TM Score
0.0
0.5
1.0
1.5
2.0
0.1
0.2
0.3
0.4
0.5

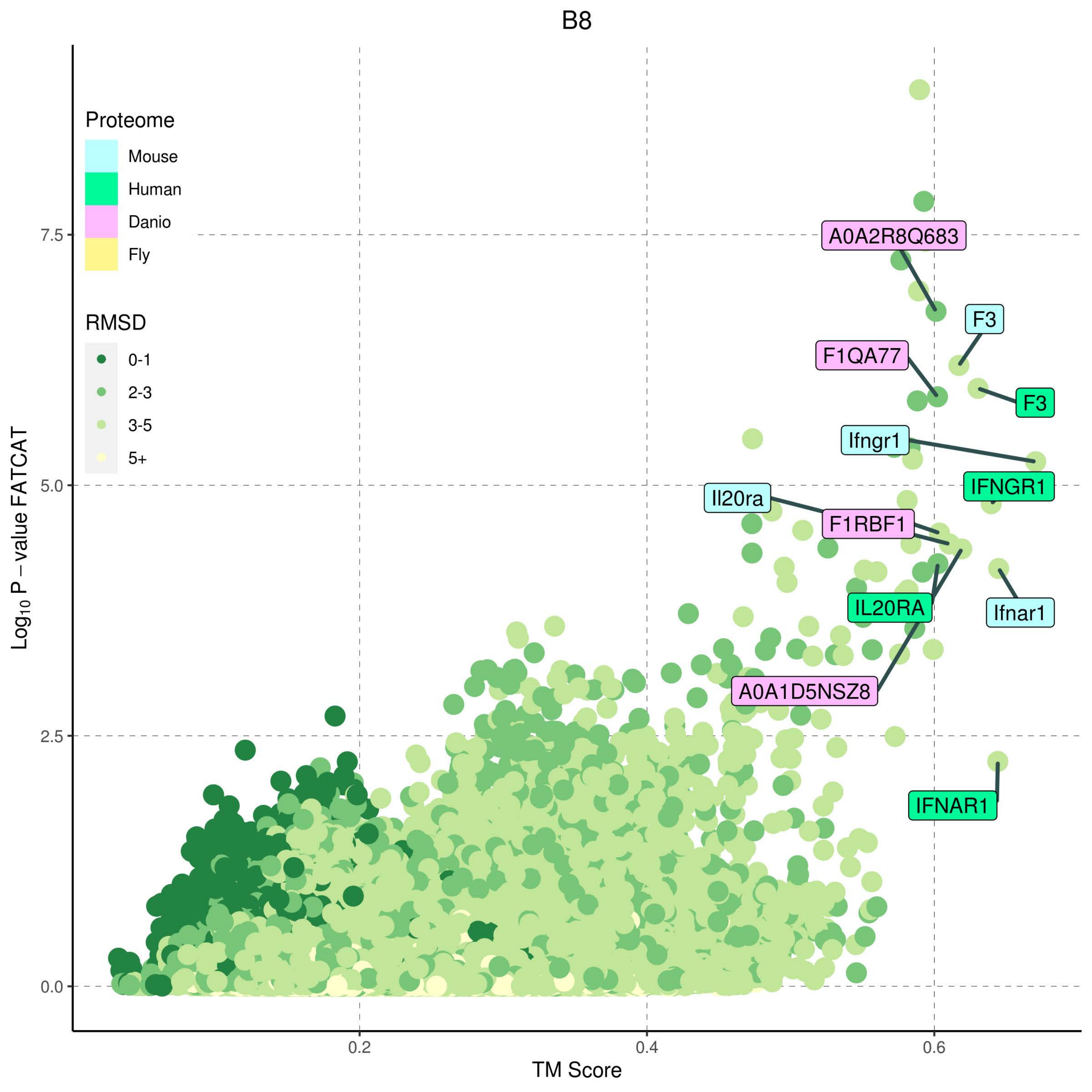

B8
Proteome
Mouse
Human
Danio
Fly
RMSD
0-1
2-3
3-5
5+
7.5
5.0
2.5
0.0
Log_{10} P − value FATCAT
0.2
0.4
0.6
TM Score
A0A2R8Q683
F3
F1QA77
F3
Ifngr1
IFNGR1
Il20ra
F1RBF1
IL20RA
Ifnar1
A0A1D5NSZ8
IFNAR1

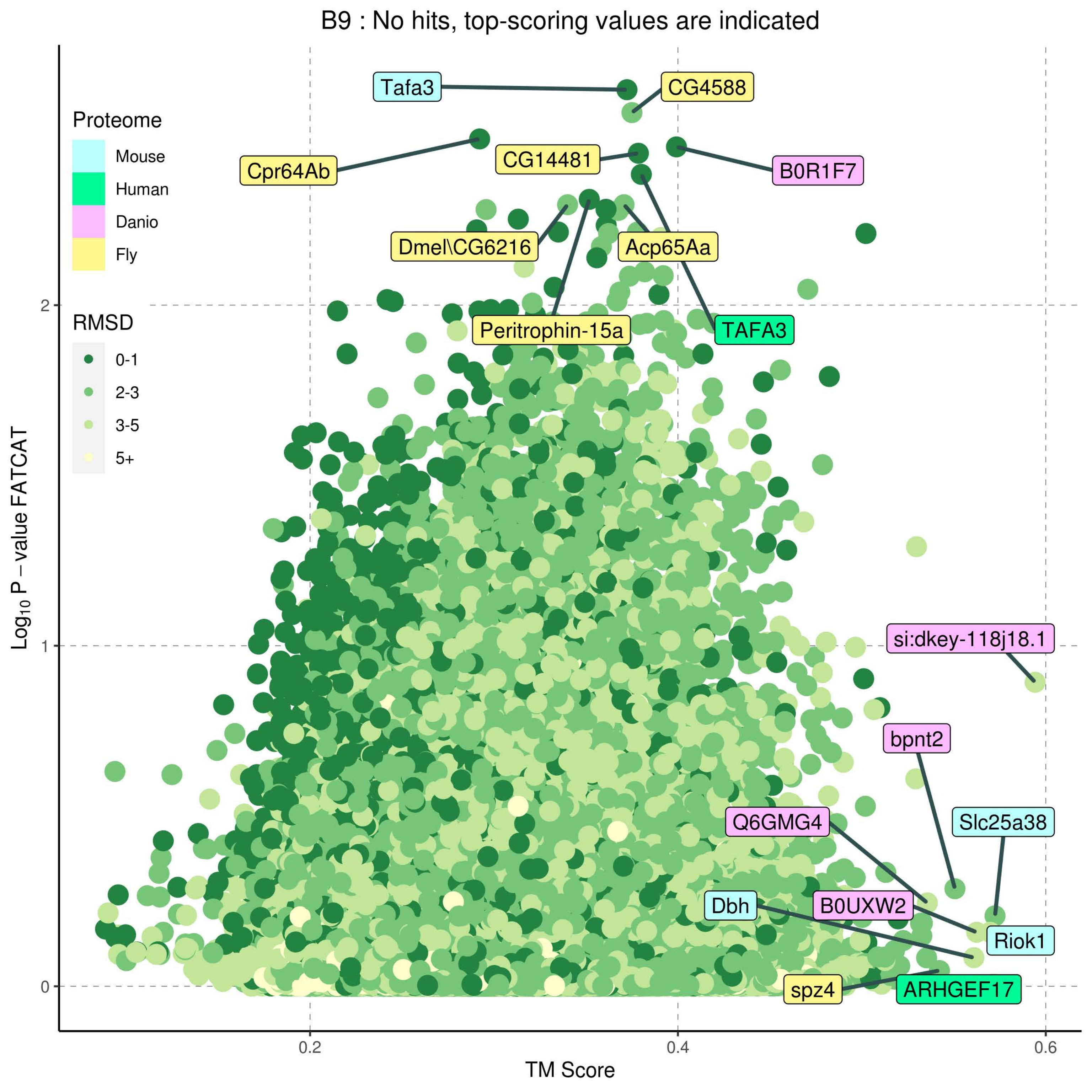

B9 : No hits, top-scoring values are indicated
Proteome
Mouse
Human
Danio
Fly
RMSD
0-1
2-3
3-5
5+
Tafa3
CG4588
Cpr64Ab
CG14481
B0R1F7
Dmel\CG6216
Acp65Aa
Peritrophin-15a
TAFA3
si:dkey-118j18.1
bpnt2
Q6GMG4
Slc25a38
Dbh
B0UXW2
Riok1
spz4
ARHGEF17
Log_{10} P – value FATCAT
TM Score
0.2
0.4
0.6
0
1
2

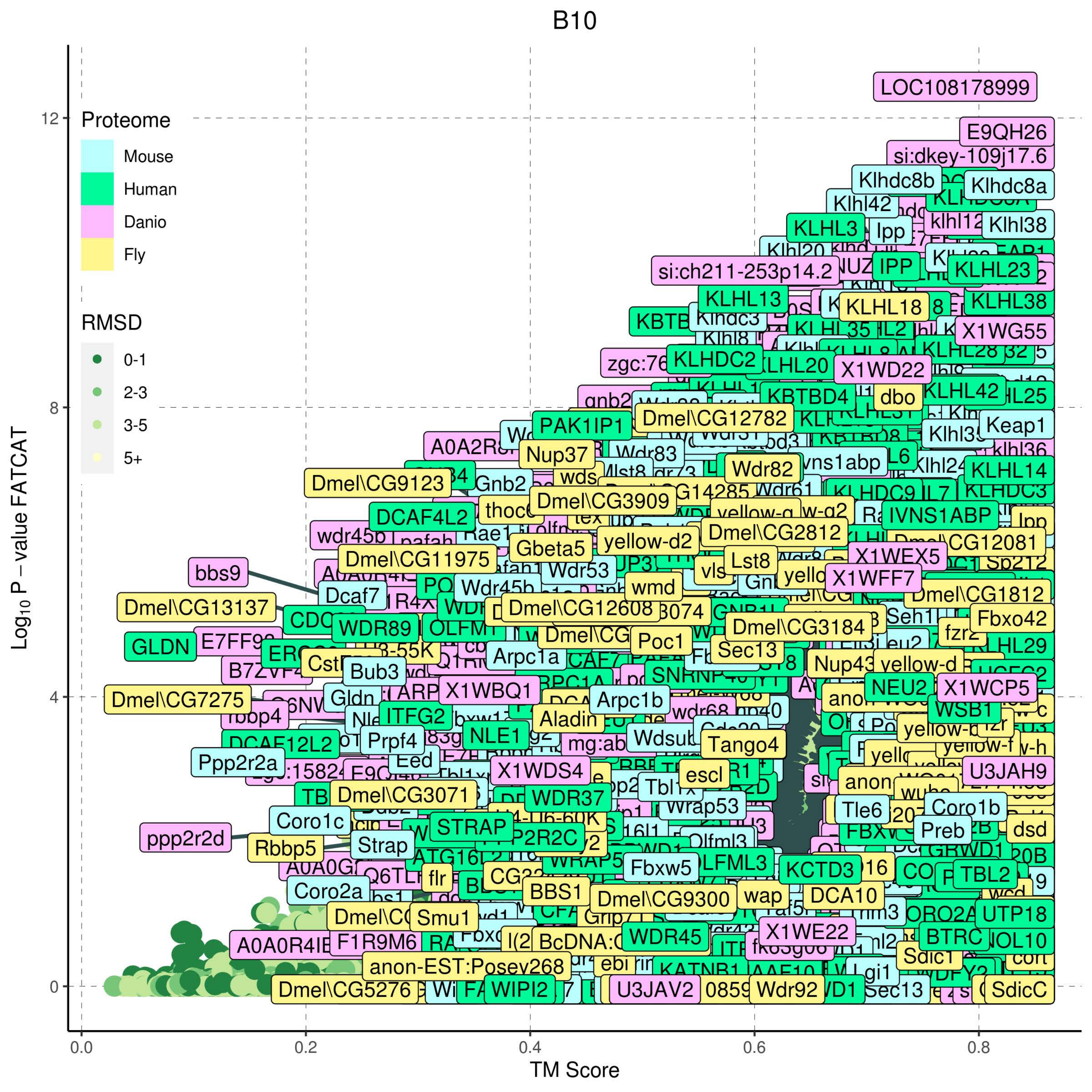
B10
Proteome
Mouse
Human
Danio
Fly
RMSD
0-1
2-3
3-5
5+
Log10 P – value FATCAT
TM Score
0
4
8
12
0.0
0.2
0.4
0.6
0.8
LOC108178999
E9QH26
si:dkey-109j17.6
Klhdc8b
Klhdc8a
Klhl42
KLHL3
Ipp
Klhl38
si:ch211-253p14.2
IPP
KLHL23
KLHL13
KLHL18
KLHL38
X1WG55
KLHDC2
KLHL20
X1WD22
KBTBD4
dbo
KLHL42
Dmel\CG12782
PAK1IP1
Keap1
Nup37
Wdr83
Wdr82
KLHL14
Dmel\CG9123
Gnb2
Dmel\CG3909
DCAF4L2
IVNS1ABP
wdr45b
yellow-d2
Dmel\CG2812
Gbeta5
Dmel\CG11975
X1WEX5
bbs9
Wdr53
Lst8
X1WFF7
Dcaf7
wmd
Dmel\CG13137
Dmel\CG12608
WDR89
Dmel\CG3184
Fbxo42
GLDN
Poc1
Arpc1a
Sec13
Bub3
Nup43
X1WBQ1
NEU2
X1WCP5
Dmel\CG7275
Arpc1b
ITFG2
Aladin
NLE1
Tango4
Prpf4
Ppp2r2a
X1WDS4
escl
U3JAH9
Dmel\CG3071
WDR37
Coro1c
Tle6
Coro1b
STRAP
Preb
dsd
ppp2r2d
Rbbp5
Strap
Fbxw5
KCTD3
TBL2
Coro2a
BBS1
Dmel\CG9300
wap
UTP18
Smu1
X1WE22
F1R9M6
WDR45
BTRC
anon-EST:Posev268
Lgi1
Dmel\CG5276
WIPI2
U3JAV2
Wdr92
Sec13
SdicC

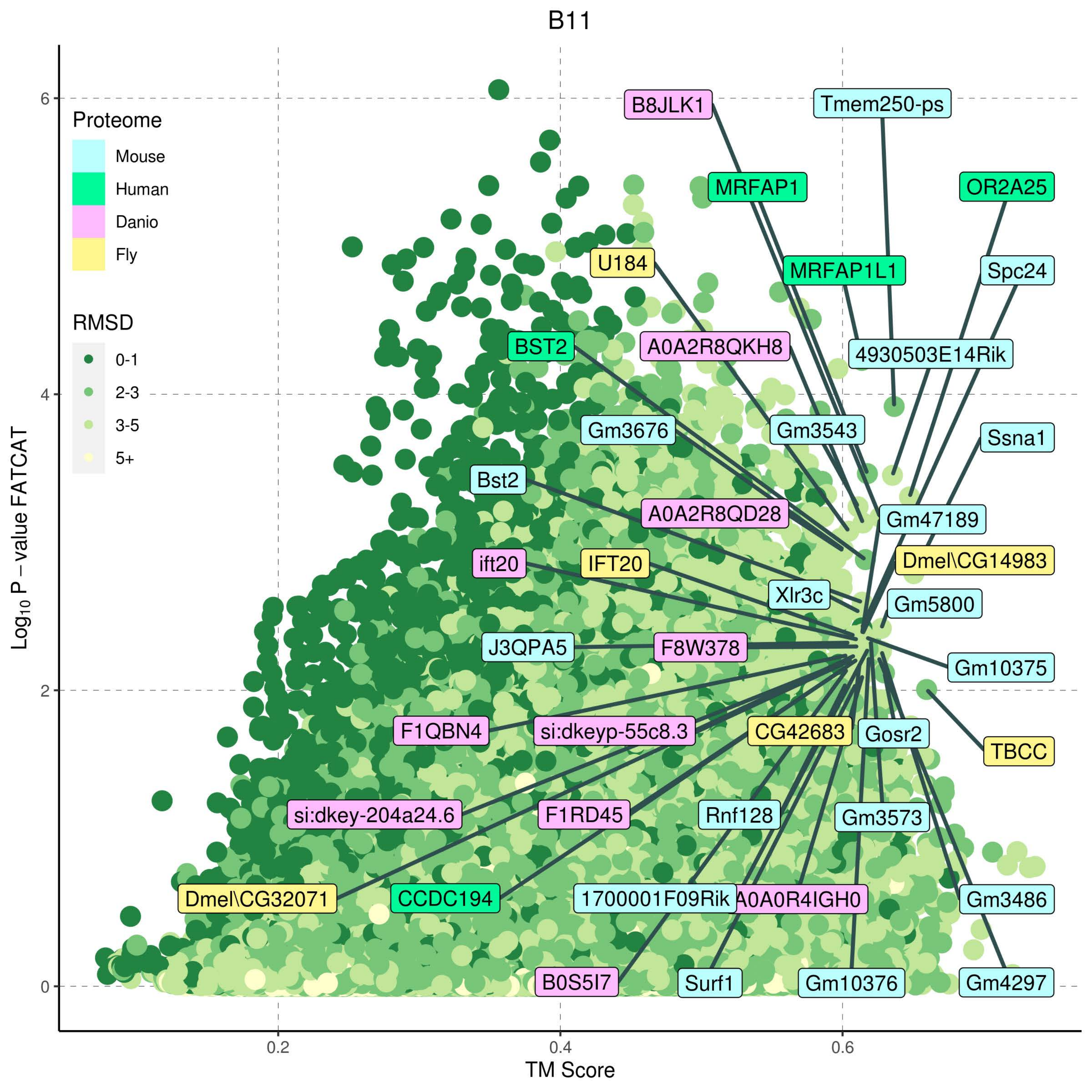
B11
Proteome
Mouse
Human
Danio
Fly
RMSD
0-1
2-3
3-5
5+
Log10 P − value FATCAT
TM Score
0
2
4
6
0.2
0.4
0.6
B8JLK1
Tmem250-ps
MRFAP1
OR2A25
MRFAP1L1
Spc24
U184
BST2
A0A2R8QKH8
4930503E14Rik
Gm3676
Gm3543
Ssna1
Bst2
A0A2R8QD28
Gm47189
ift20
IFT20
Dmel\CG14983
Xlr3c
Gm5800
J3QPA5
F8W378
Gm10375
F1QBN4
si:dkeyp-55c8.3
CG42683
Gosr2
TBCC
si:dkey-204a24.6
F1RD45
Rnf128
Gm3573
Dmel\CG32071
CCDC194
1700001F09Rik
A0A0R4IGH0
Gm3486
B0S5I7
Surf1
Gm10376
Gm4297

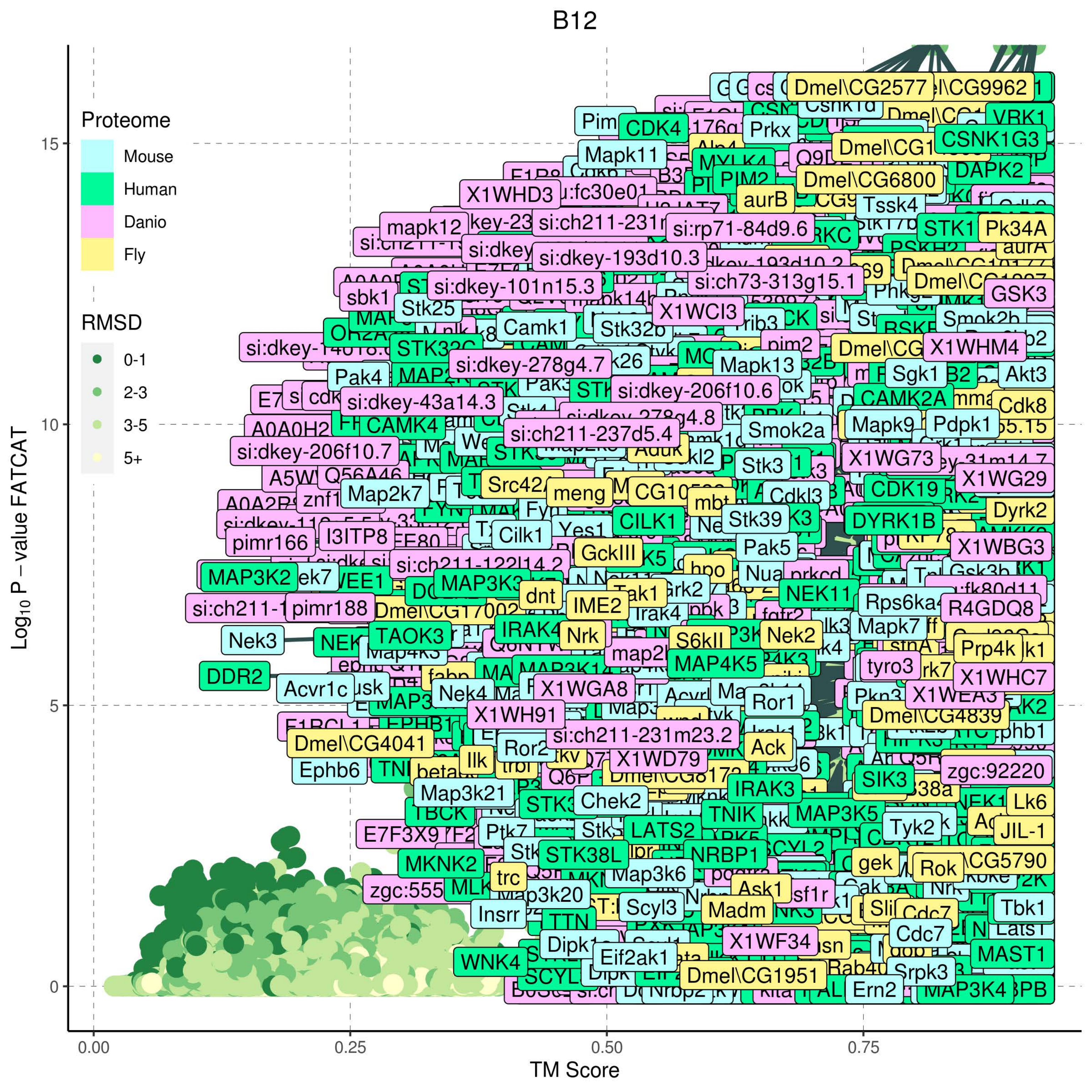

B12
Proteome
Mouse
Human
Danio
Fly
RMSD
0-1
2-3
3-5
5+
Log10 P – value FATCAT
TM Score
0.00
0.25
0.50
0.75
0
5
10
15
Dmel\CG2577
VRK1
CSNK1G3
DAPK2
Dmel\CG6800
CDK4
Prkx
Mapk11
PIM2
X1WHD3
aurB
Tssk4
mapk12
si:rp71-84d9.6
Pk34A
si:dkey-193d10.3
si:ch73-313g15.1
GSK3
sbk1
si:dkey-101n15.3
Stk25
X1WCI3
Camk1
Stk32b
Smok2b
X1WHM4
STK32C
si:dkey-278g4.7
Mapk13
Pak4
Sgk1
Akt3
si:dkey-206f10.6
si:dkey-43a14.3
CAMK2A
Cdk8
CAMK4
Mapk9
Pdpk1
si:ch211-237d5.4
Smok2a
si:dkey-206f10.7
X1WG73
Stk3
X1WG29
Map2k7
Src42A
meng
Cdkl3
CDK19
Stk39
CILK1
Dyrk2
DYRK1B
pimr166
I3ITP8
Cilk1
Yes1
GckIII
Pak5
X1WBG3
MAP3K2
hpo
dnt
IME2
Tak1
NEK11
R4GDQ8
si:ch211-1
pimr188
Rps6ka4
Nek3
TAOK3
IRAK4
Nrk
S6kII
Nek2
Mapk7
Prp4k
tyro3
MAP4K5
X1WHC7
DDR2
Acvr1c
Nek4
X1WGA8
Ror1
X1WEA3
X1WH91
Dmel\CG4839
si:ch211-231m23.2
Dmel\CG4041
Ilk
Ror2
X1WD79
Ack
Ephb6
zgc:92220
SIK3
Map3k21
Chek2
IRAK3
Lk6
TNIK
MAP3K5
Tyk2
JIL-1
E7F3X9
LATS2
STK38L
NRBP1
MKNK2
gek
Rok
trc
Map3k6
Ask1
Map3k20
Scyl3
Madm
Tbk1
Insrr
TTN
Cdc7
X1WF34
WNK4
Eif2ak1
MAST1
Dmel\CG1951
Srpk3
Ern2
MAP3K4

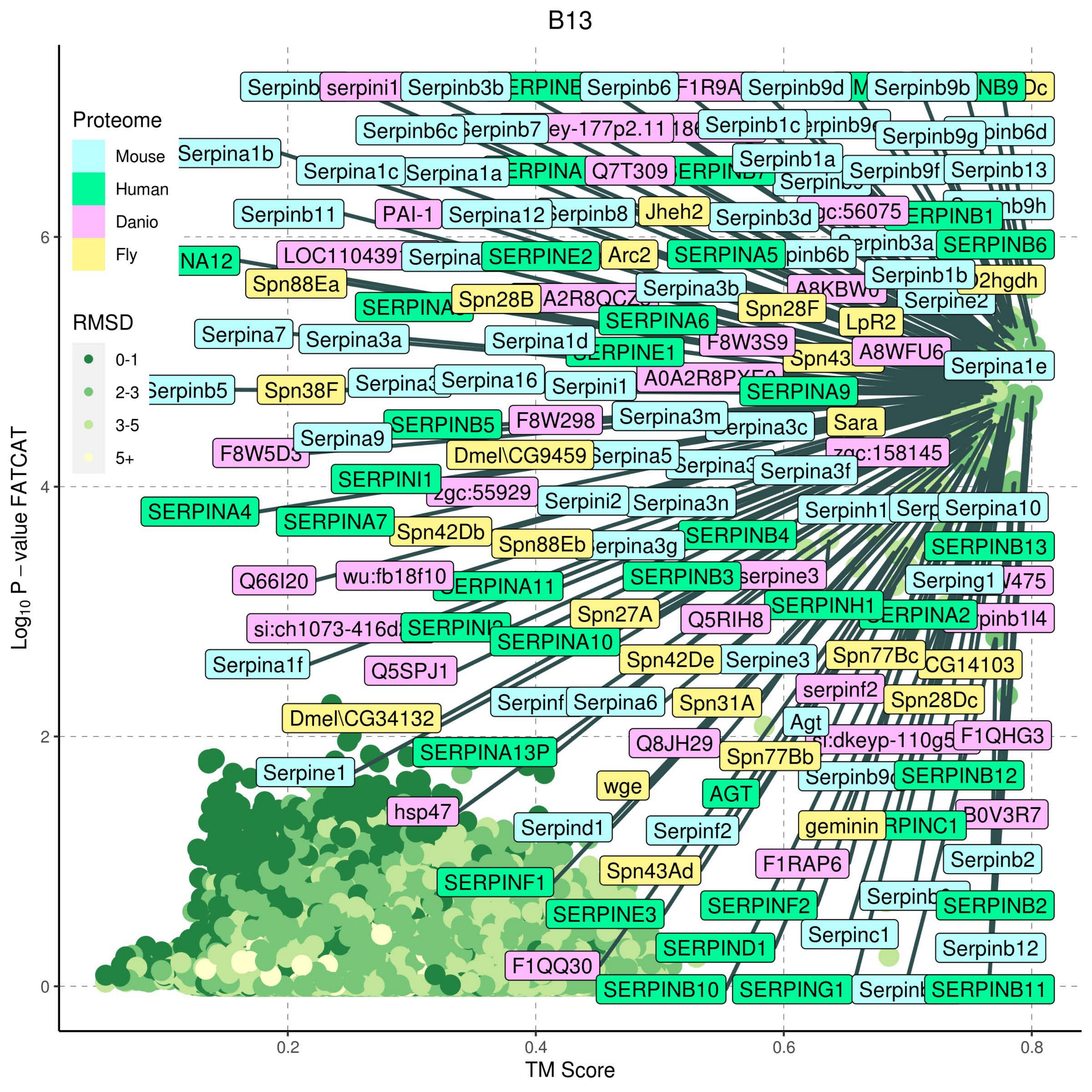
B13
Proteome
Mouse
Human
Danio
Fly
RMSD
0-1
2-3
3-5
5+
Log10 P − value FATCAT
TM Score
0
2
4
6
0.2
0.4
0.6
0.8
serpini1
Serpinb3b
Serpinb6
Serpinb9d
Serpinb9b
Serpinb6c
Serpinb1c
Serpinb9g
Serpina1b
Serpina1c
Serpina1a
Q7T309
Serpinb1a
Serpinb9f
Serpinb13
Serpinb11
PAI-1
Serpina12
Jheh2
Serpinb3d
SERPINB6
SERPINE2
Arc2
SERPINA5
Serpinb1b
Spn88Ea
Serpina3b
Serpine2
Spn28B
Spn28F
LpR2
SERPINA6
Serpina7
Serpina3a
Serpina1d
F8W3S9
A8WFU6
Serpina1e
Spn38F
Serpina16
Serpini1
SERPINA9
SERPINB5
F8W298
Serpina3m
Sara
Serpina9
Dmel\CG9459
Serpina3f
SERPINI1
zgc:55929
Serpini2
Serpina3n
Serpinh1
Serpina10
SERPINA4
SERPINA7
Spn42Db
Spn88Eb
SERPINB4
SERPINB13
Q66I20
wu:fb18f10
SERPINB3
serpine3
Serping1
SERPINH1
Spn27A
Q5RIH8
SERPINA10
Serpina1f
Q5SPJ1
Spn42De
Serpine3
Spn77Bc
CG14103
serpinf2
Serpina6
Spn31A
Spn28Dc
Dmel\CG34132
Agt
F1QHG3
Q8JH29
SERPINA13P
Spn77Bb
Serpine1
SERPINB12
wge
AGT
hsp47
B0V3R7
Serpind1
Serpinf2
geminin
Serpinb2
Spn43Ad
F1RAP6
SERPINF1
SERPINF2
SERPINB2
SERPINE3
Serpinc1
SERPIND1
Serpinb12
F1QQ30
SERPINB10
SERPING1
SERPINB11

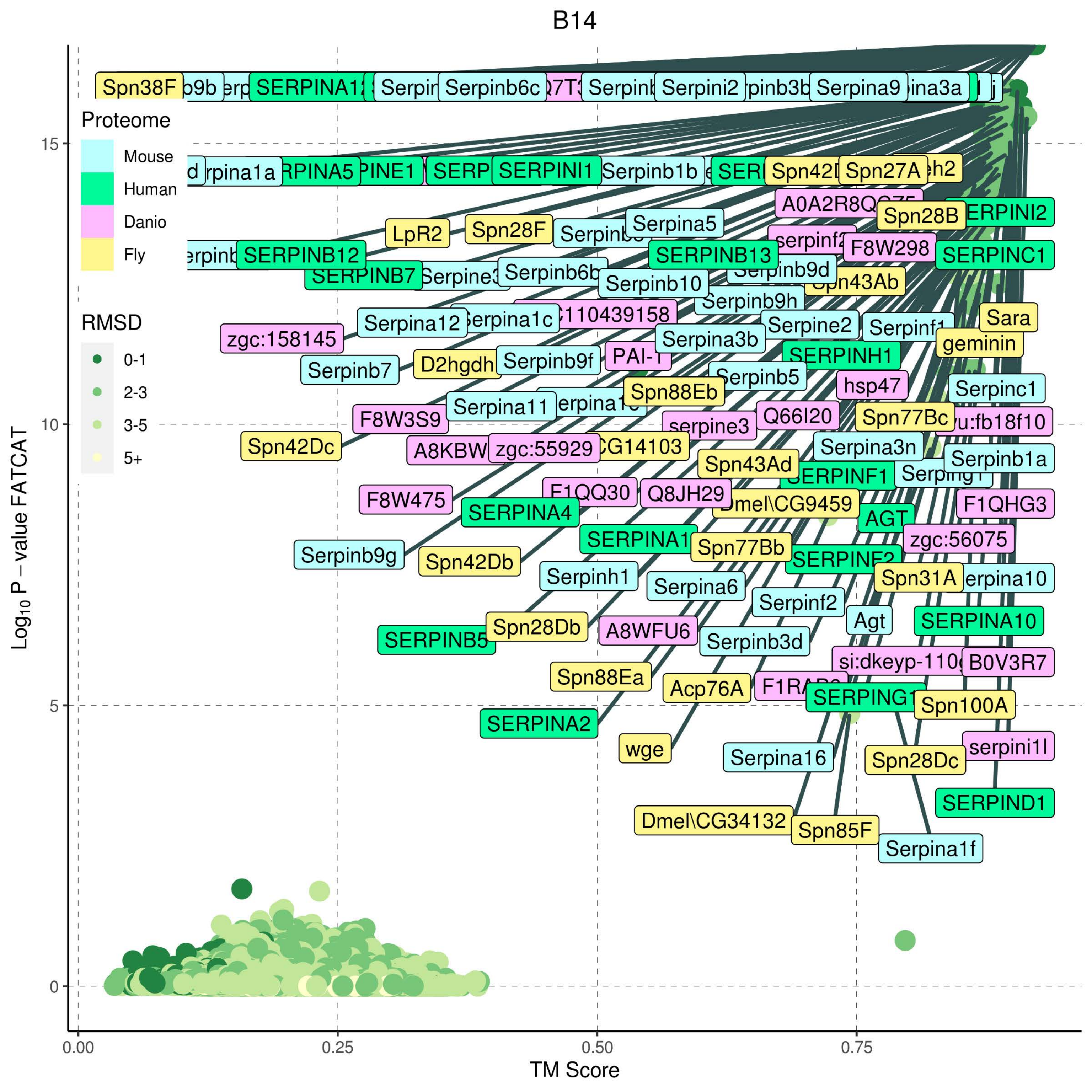

B14
Proteome
Mouse
Human
Danio
Fly
RMSD
0-1
2-3
3-5
5+
Log10 P – value FATCAT
TM Score
0
5
10
15
0.00
0.25
0.50
0.75
Spn38F
Serpinb6c
Serpini2
Serpina9
Serpinb1b
SERPINI1
Spn27A
Spn28B
LpR2
Spn28F
Serpina5
SERPINB13
F8W298
SERPINC1
SERPINB12
Serpinb6b
Serpinb10
Serpinb9h
Serpina12
Serpine2
Sara
zgc:158145
Serpina3b
geminin
Serpinb7
D2hgdh
Serpinb9f
PAI-1
SERPINH1
hsp47
Spn88Eb
Serpinc1
Serpina11
Q66I20
Spn77Bc
F8W3S9
serpine3
Spn42Dc
zgc:55929
Serpina3n
Serpinb1a
Spn43Ad
SERPINF1
Q8JH29
F8W475
F1QHG3
SERPINA4
AGT
zgc:56075
Serpinb9g
Spn77Bb
Spn42Db
Serpinh1
Spn31A
Serpina6
Serpinf2
Agt
SERPINA10
SERPINB5
Spn28Db
A8WFU6
Serpinb3d
B0V3R7
Spn88Ea
Acp76A
SERPING1
Spn100A
SERPINA2
wge
serpini1l
Serpina16
Spn28Dc
SERPIND1
Dmel\CG34132
Spn85F
Serpina1f

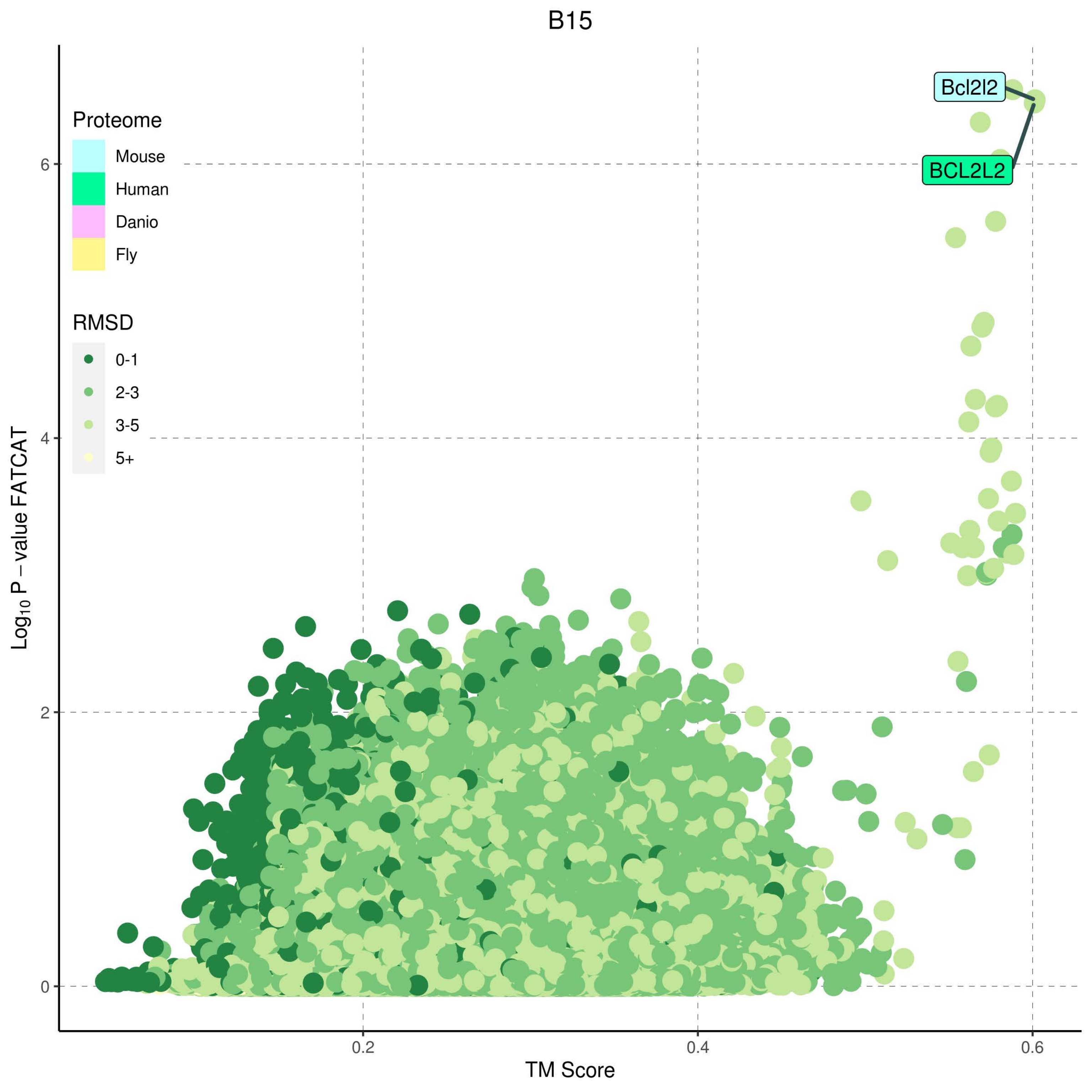

B15
Proteome
Mouse
Human
Danio
Fly
RMSD
0-1
2-3
3-5
5+
Bcl2l2
BCL2L2
Log10 P − value FATCAT
TM Score
0
2
4
6
0.2
0.4
0.6

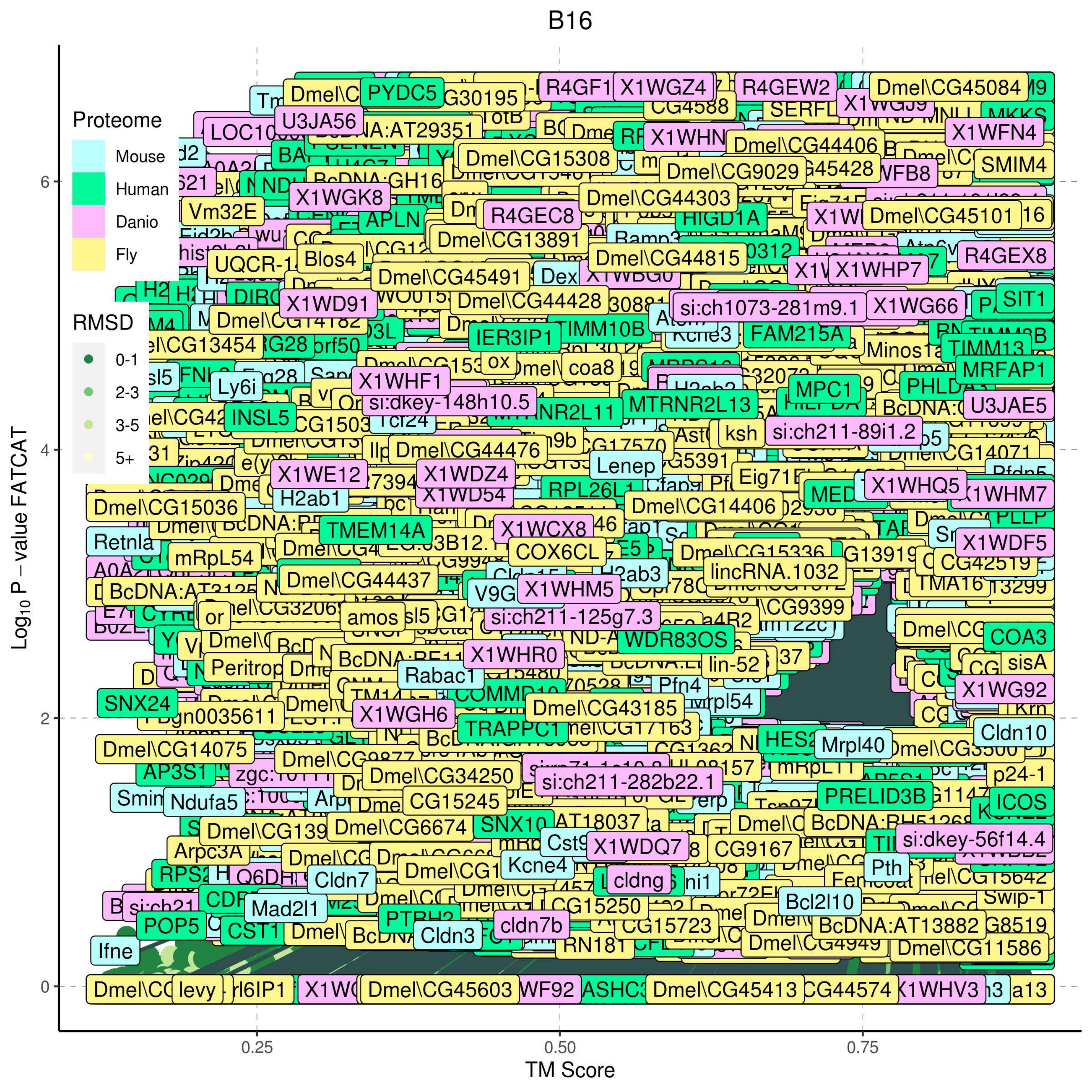
B16
Proteome
Mouse
Human
Danio
Fly
RMSD
0-1
2-3
3-5
5+
Log_{10} P – value FATCAT
0
2
4
6
0.25
0.50
0.75
TM Score

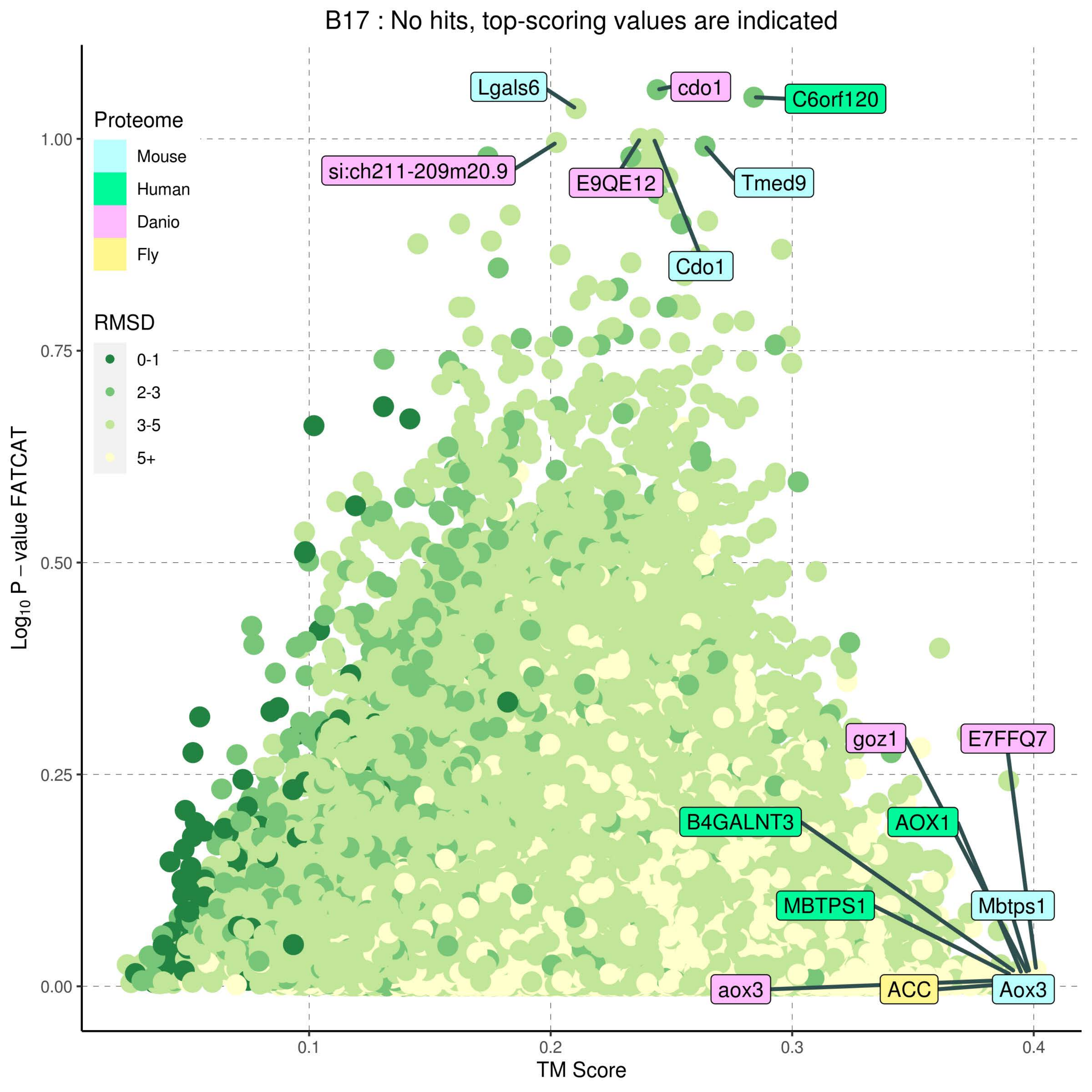
B17 : No hits, top-scoring values are indicated
Proteome
Mouse
Human
Danio
Fly
RMSD
0-1
2-3
3-5
5+
Lgals6
cdo1
C6orf120
si:ch211-209m20.9
E9QE12
Tmed9
Cdo1
goz1
E7FFQ7
B4GALNT3
AOX1
MBTPS1
Mbtps1
aox3
ACC
Aox3
1.00
0.75
0.50
0.25
0.00
0.1
0.2
0.3
0.4
Log10 P − value FATCAT
TM Score

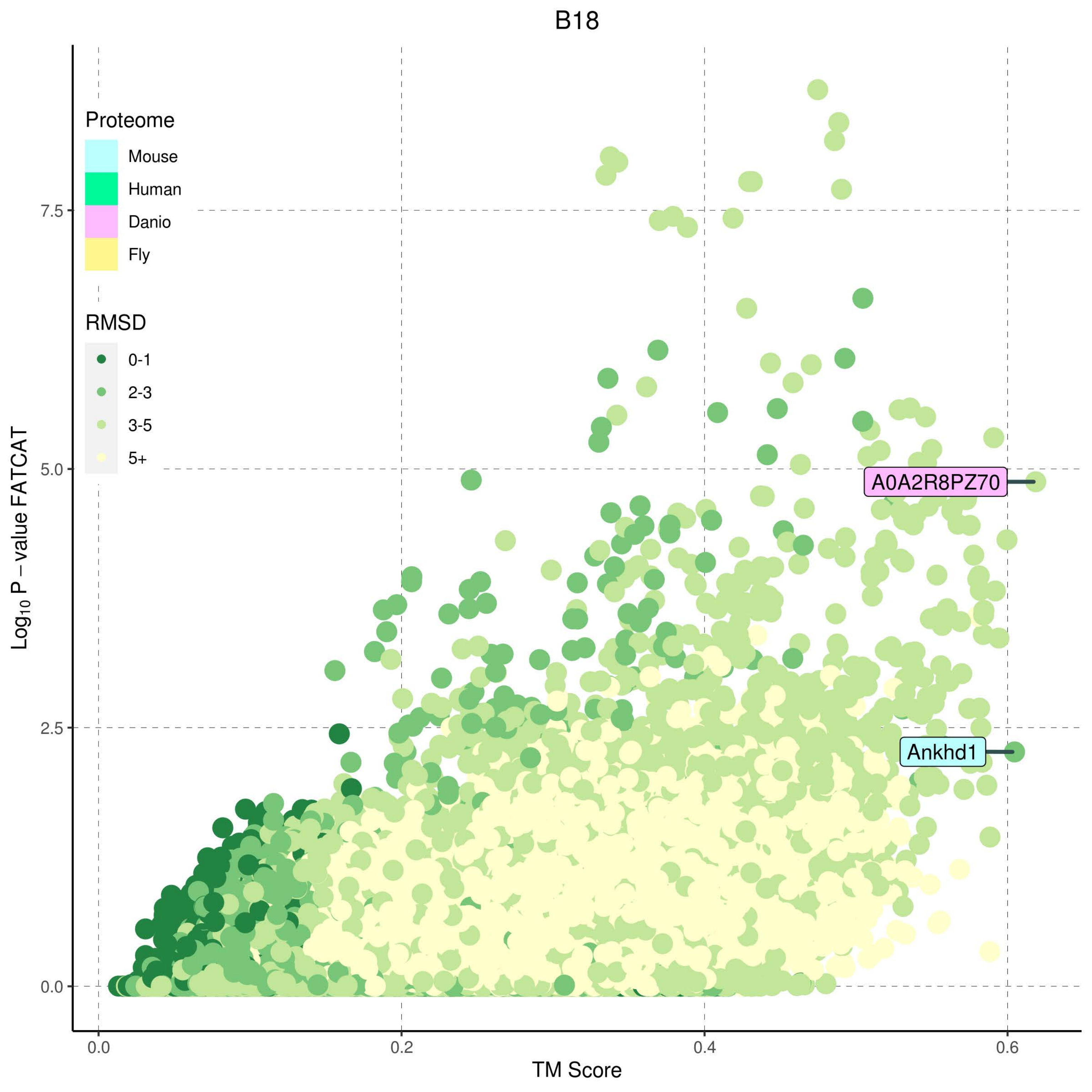

B18
Proteome
Mouse
Human
Danio
Fly
RMSD
0-1
2-3
3-5
5+
A0A2R8PZ70
Ankhd1
7.5
5.0
2.5
0.0
Log10 P − value FATCAT
0.0
0.2
0.4
0.6
TM Score

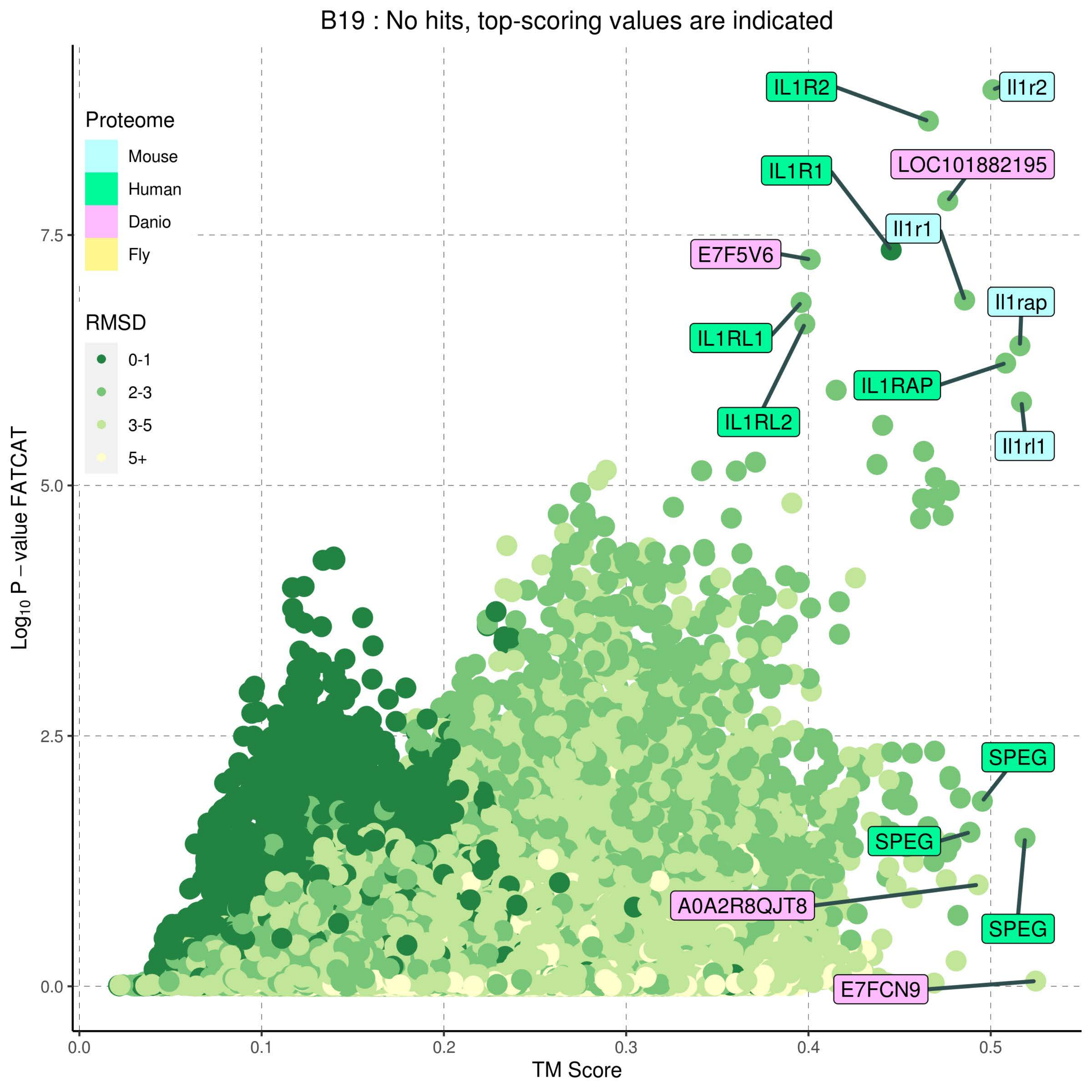

B19 : No hits, top-scoring values are indicated
Proteome
Mouse
Human
Danio
Fly
RMSD
0-1
2-3
3-5
5+
IL1R2
Il1r2
LOC101882195
IL1R1
Il1r1
E7F5V6
IL1RL1
Il1rap
IL1RAP
IL1RL2
Il1rl1
SPEG
SPEG
A0A2R8QJT8
SPEG
E7FCN9
0.0
2.5
5.0
7.5
0.0
0.1
0.2
0.3
0.4
0.5
TM Score
Log_{10} P − value FATCAT

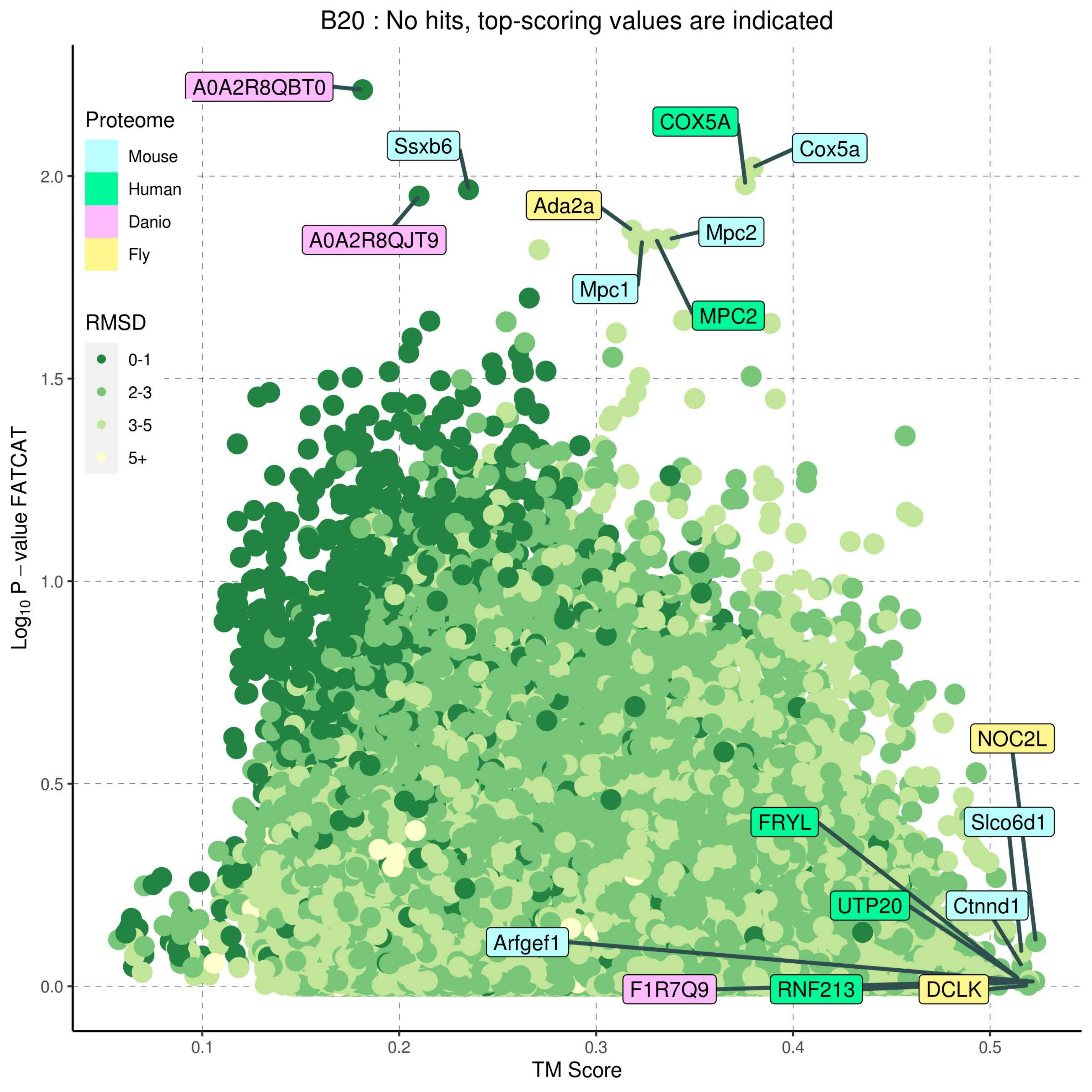

B20 : No hits, top-scoring values are indicated
Proteome
Mouse
Human
Danio
Fly
RMSD
0-1
2-3
3-5
5+
A0A2R8QBT0
Ssxb6
A0A2R8QJT9
COX5A
Cox5a
Ada2a
Mpc2
Mpc1
MPC2
NOC2L
Slco6d1
FRYL
UTP20
Ctnnd1
Arfgef1
F1R7Q9
RNF213
DCLK
Log_{10} P – value FATCAT
TM Score
2.0
1.5
1.0
0.5
0.0
0.1
0.2
0.3
0.4
0.5

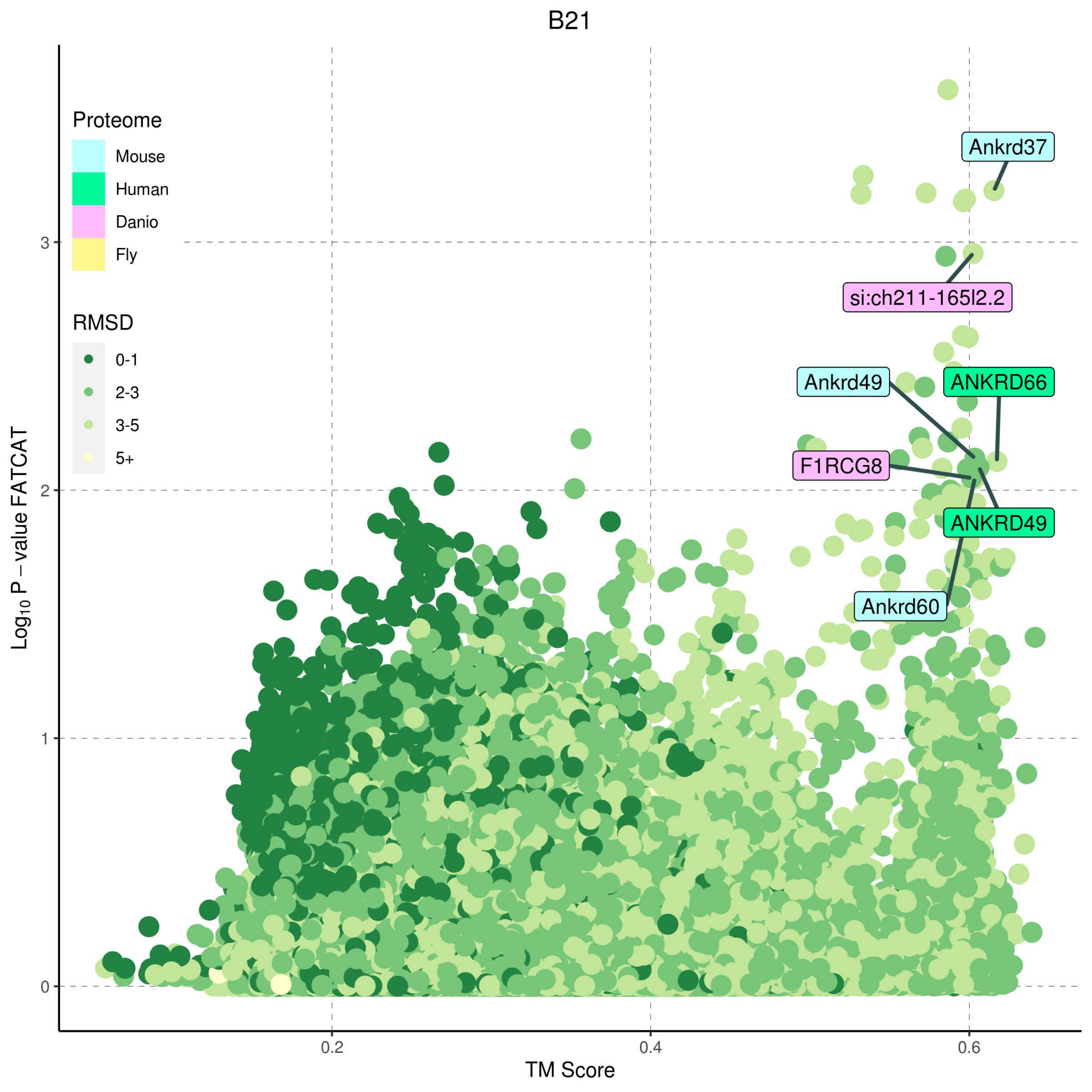

B21
Proteome
Mouse
Human
Danio
Fly
RMSD
0-1
2-3
3-5
5+
Ankrd37
si:ch211-165l2.2
Ankrd49
ANKRD66
F1RCG8
ANKRD49
Ankrd60
$\log_{10}$ P − value FATCAT
TM Score
0
1
2
3
0.2
0.4
0.6

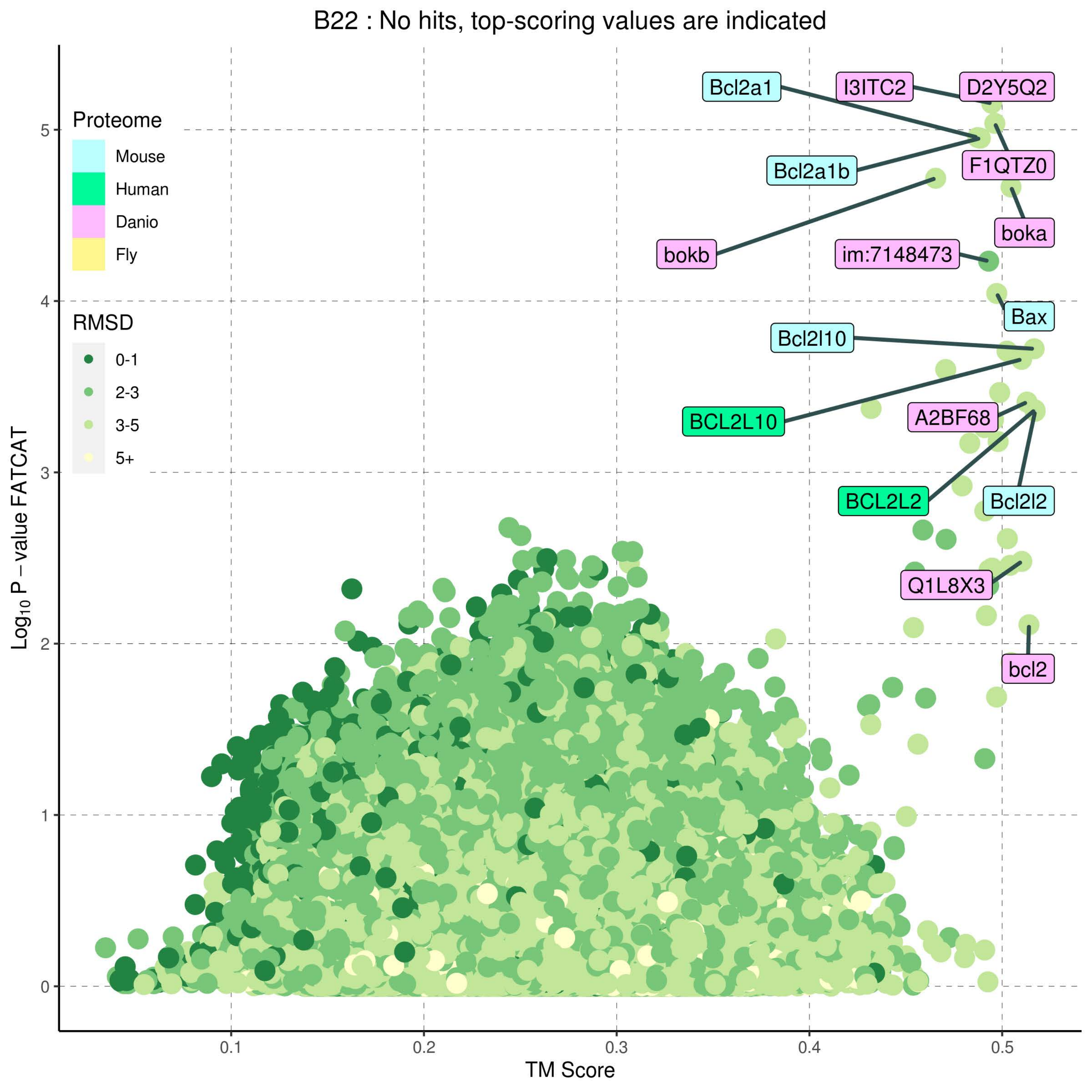

B22 : No hits, top-scoring values are indicated
Proteome
Mouse
Human
Danio
Fly
RMSD
0-1
2-3
3-5
5+
Bcl2a1
I3ITC2
D2Y5Q2
F1QTZ0
Bcl2a1b
boka
bokb
im:7148473
Bax
Bcl2l10
BCL2L10
A2BF68
BCL2L2
Bcl2l2
Q1L8X3
bcl2
Log10 P − value FATCAT
TM Score
0
1
2
3
4
5
0.1
0.2
0.3
0.4
0.5

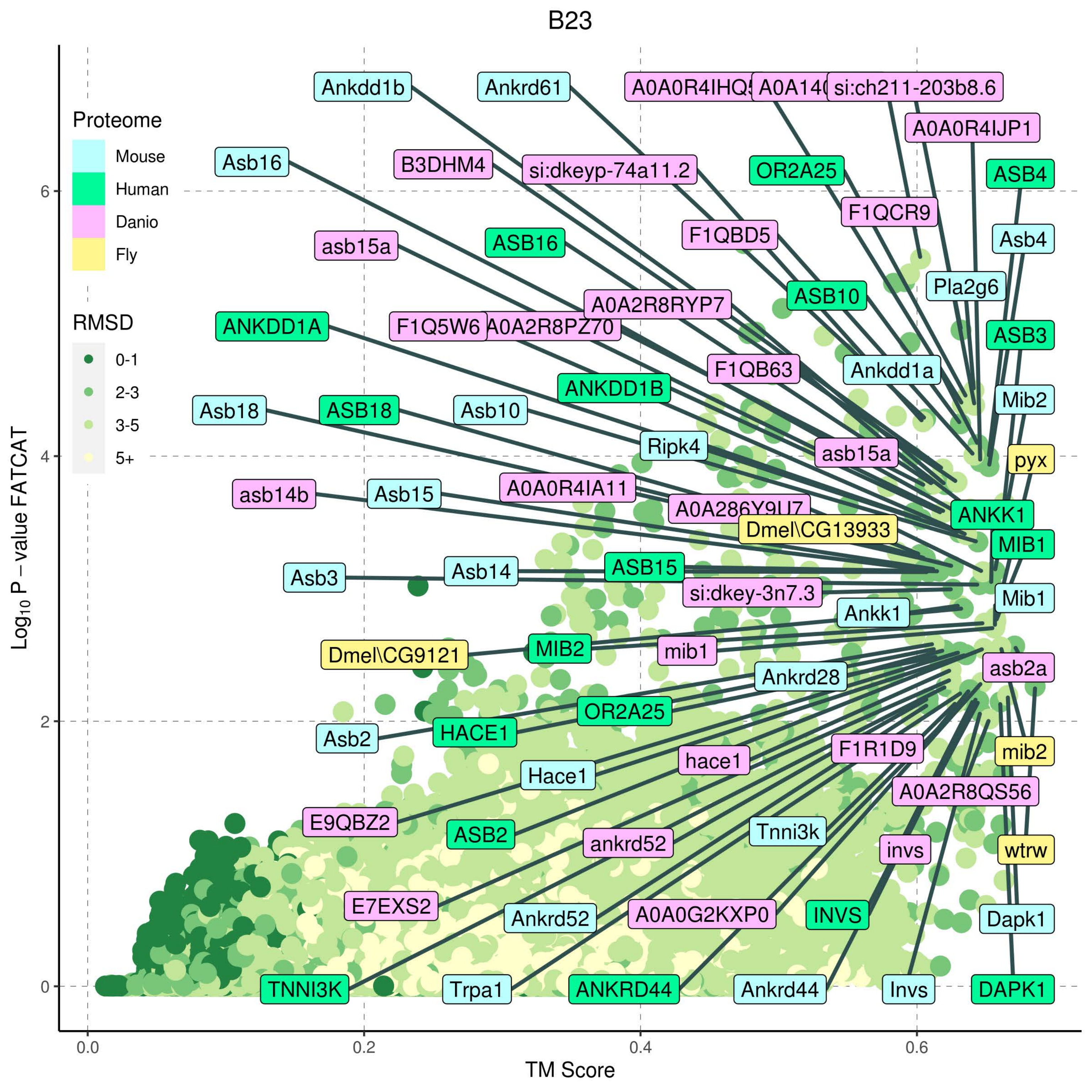

B23
Proteome
Mouse
Human
Danio
Fly
RMSD
0-1
2-3
3-5
5+
Log_{10} P − value FATCAT
TM Score
0.0
0.2
0.4
0.6
0
2
4
6
Ankdd1b
Ankrd61
A0A0R4IHQ5
A0A140
si:ch211-203b8.6
A0A0R4IJP1
Asb16
B3DHM4
si:dkeyp-74a11.2
OR2A25
ASB4
F1QCR9
F1QBD5
Asb4
asb15a
ASB16
Pla2g6
ASB10
A0A2R8RYP7
ANKDD1A
F1Q5W6
A0A2R8PZ70
ASB3
F1QB63
Ankdd1a
ANKDD1B
Mib2
Asb18
ASB18
Asb10
Ripk4
asb15a
pyx
asb14b
Asb15
A0A0R4IA11
A0A286Y9U7
ANKK1
Dmel\CG13933
MIB1
Asb3
Asb14
ASB15
si:dkey-3n7.3
Mib1
Ankk1
Dmel\CG9121
MIB2
mib1
asb2a
Ankrd28
OR2A25
Asb2
HACE1
F1R1D9
mib2
hace1
Hace1
A0A2R8QS56
E9QBZ2
ASB2
Tnni3k
ankrd52
invs
wtrw
E7EXS2
Ankrd52
A0A0G2KXP0
INVS
Dapk1
TNNI3K
Trpa1
ANKRD44
Ankrd44
Invs
DAPK1

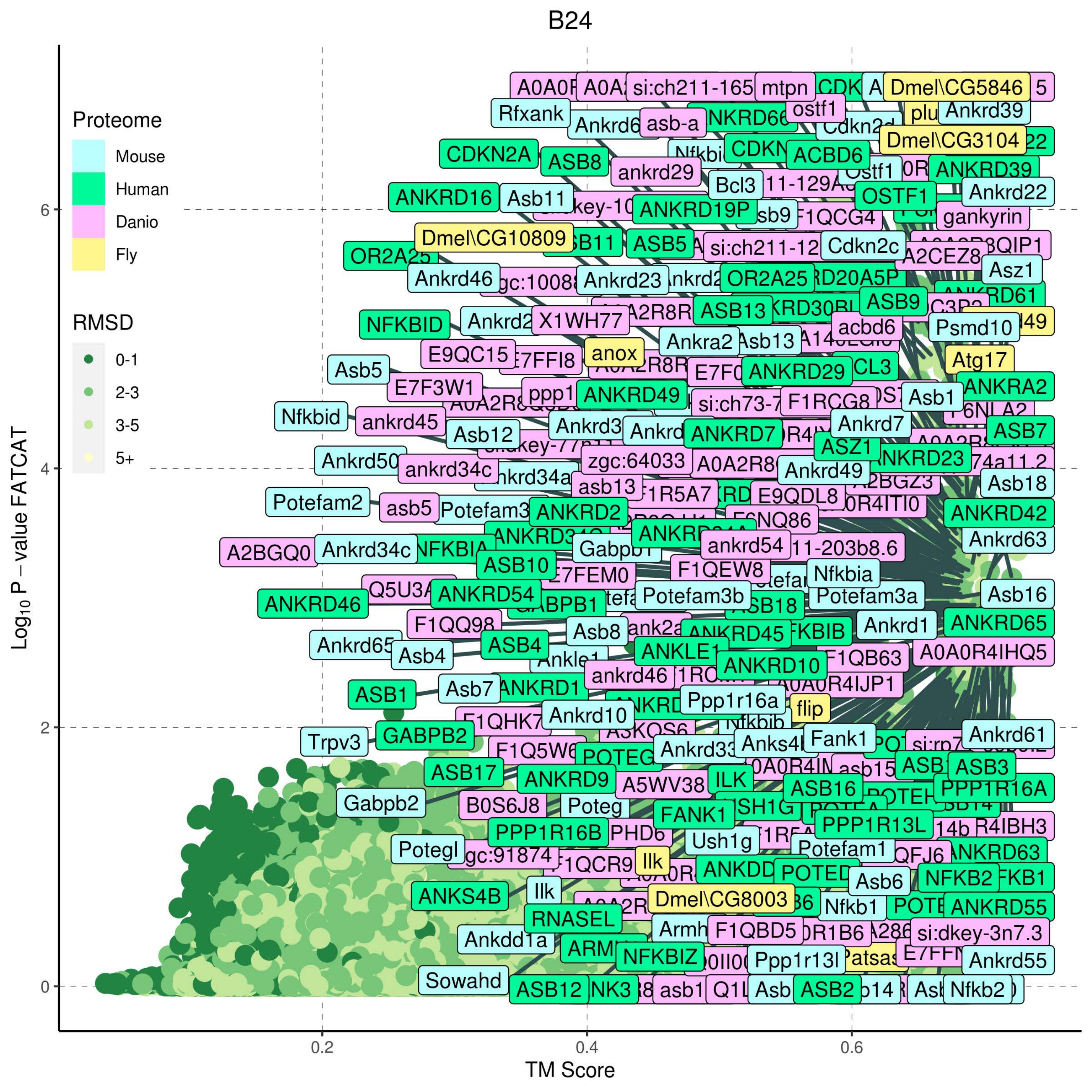

B24
Proteome
Mouse
Human
Danio
Fly
RMSD
0-1
2-3
3-5
5+
Log10 P − value FATCAT
TM Score
0
2
4
6
0.2
0.4
0.6
si:ch211-165
mtpn
Dmel\CG5846
Rfxank
asb-a
ostf1
Ankrd39
Dmel\CG3104
CDKN2A
ASB8
ACBD6
ankrd29
ANKRD39
Bcl3
ANKRD16
Asb11
ANKRD19P
OSTF1
Ankrd22
gankyrin
Dmel\CG10809
ASB5
Cdkn2c
Ankrd46
Ankrd23
OR2A25
Asz1
ASB9
NFKBID
X1WH77
ASB13
acbd6
Psmd10
Ankra2
Asb13
anox
Atg17
E9QC15
Asb5
E7F3W1
ANKRD29
ANKRD49
ANKRA2
Nfkbid
ankrd45
F1RCG8
Asb1
Asb12
ANKRD7
Ankrd7
ASB7
ASZ1
Ankrd50
ankrd34c
zgc:64033
Ankrd49
Asb18
asb13
E9QDL8
Potefam2
asb5
ANKRD2
ANKRD42
A2BGQ0
Ankrd34c
ankrd54
Ankrd63
ASB10
F1QEW8
Nfkbia
ANKRD46
ANKRD54
Potefam3b
Potefam3a
Asb16
F1QQ98
Asb8
Ankrd1
ANKRD65
Asb4
ASB4
ANKLE1
ANKRD10
F1QB63
A0A0R4IHQ5
ankrd46
A0A0R4IJP1
Asb7
ASB1
Ppp1r16a
flip
Ankrd10
Fank1
Ankrd61
Trpv3
GABPB2
ASB17
ANKRD9
A5WV38
ILK
ASB16
ASB3
PPP1R16A
Gabpb2
B0S6J8
Poteg
FANK1
PPP1R16B
PPP1R13L
Ushg1g
Potegl
Potefam1
Ilk
POTED
Asb6
NFKB2
ANKS4B
Dmel\CG8003
Nfkb1
ANKRD55
RNASEL
F1QBD5
si:dkey-3n7.3
Ankdd1a
NFKBIZ
Ppp1r13l
Ankrd55
Sowahd
ASB12
ASB2
Nfkb2

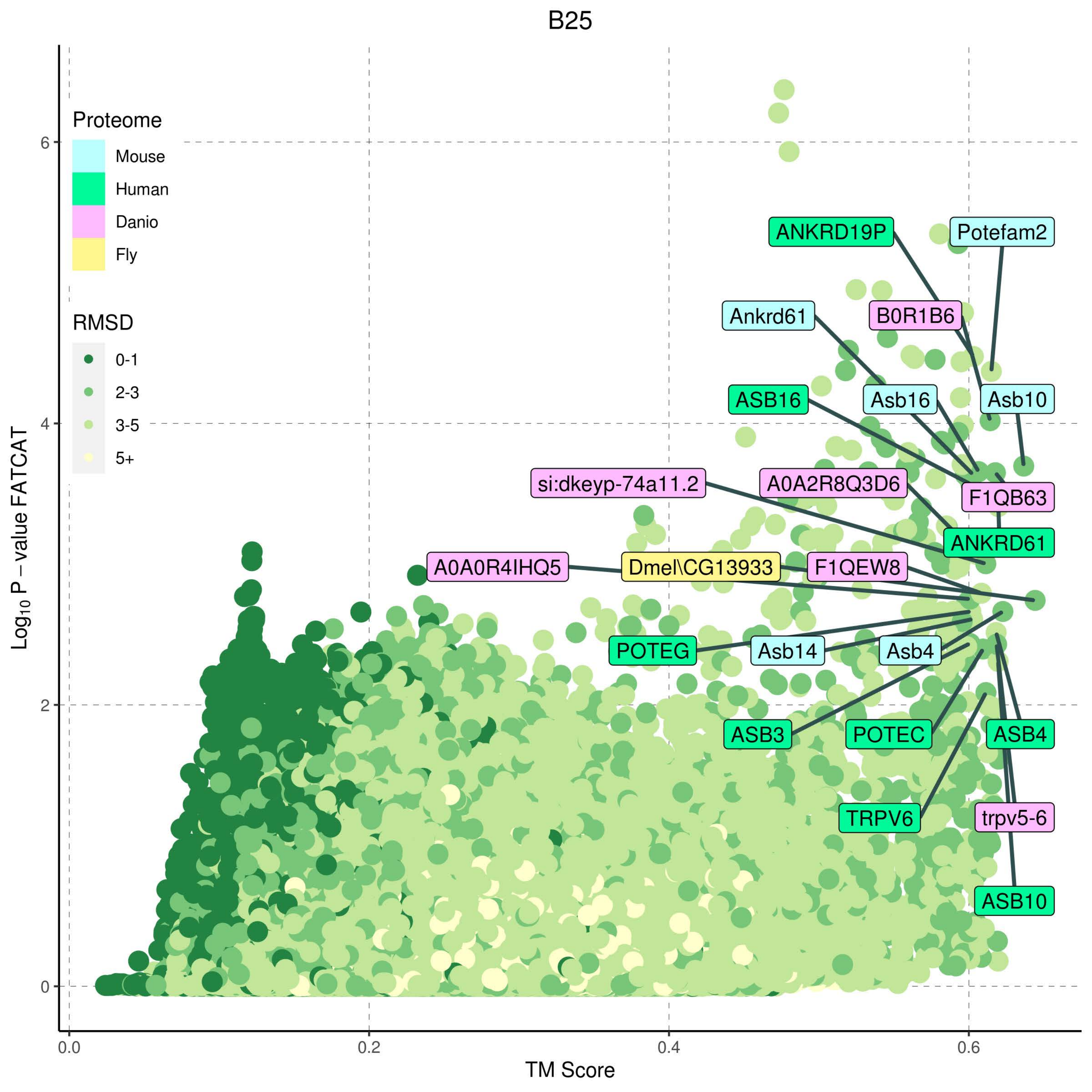

B25
Proteome
Mouse
Human
Danio
Fly
RMSD
0-1
2-3
3-5
5+
ANKRD19P
Potefam2
Ankrd61
B0R1B6
ASB16
Asb16
Asb10
si:dkeyp-74a11.2
A0A2R8Q3D6
F1QB63
ANKRD61
A0A0R4IHQ5
Dmel\CG13933
F1QEW8
POTEG
Asb14
Asb4
ASB3
POTEC
ASB4
TRPV6
trpv5-6
ASB10
Log10 P − value FATCAT
TM Score
0
2
4
6
0.0
0.2
0.4
0.6

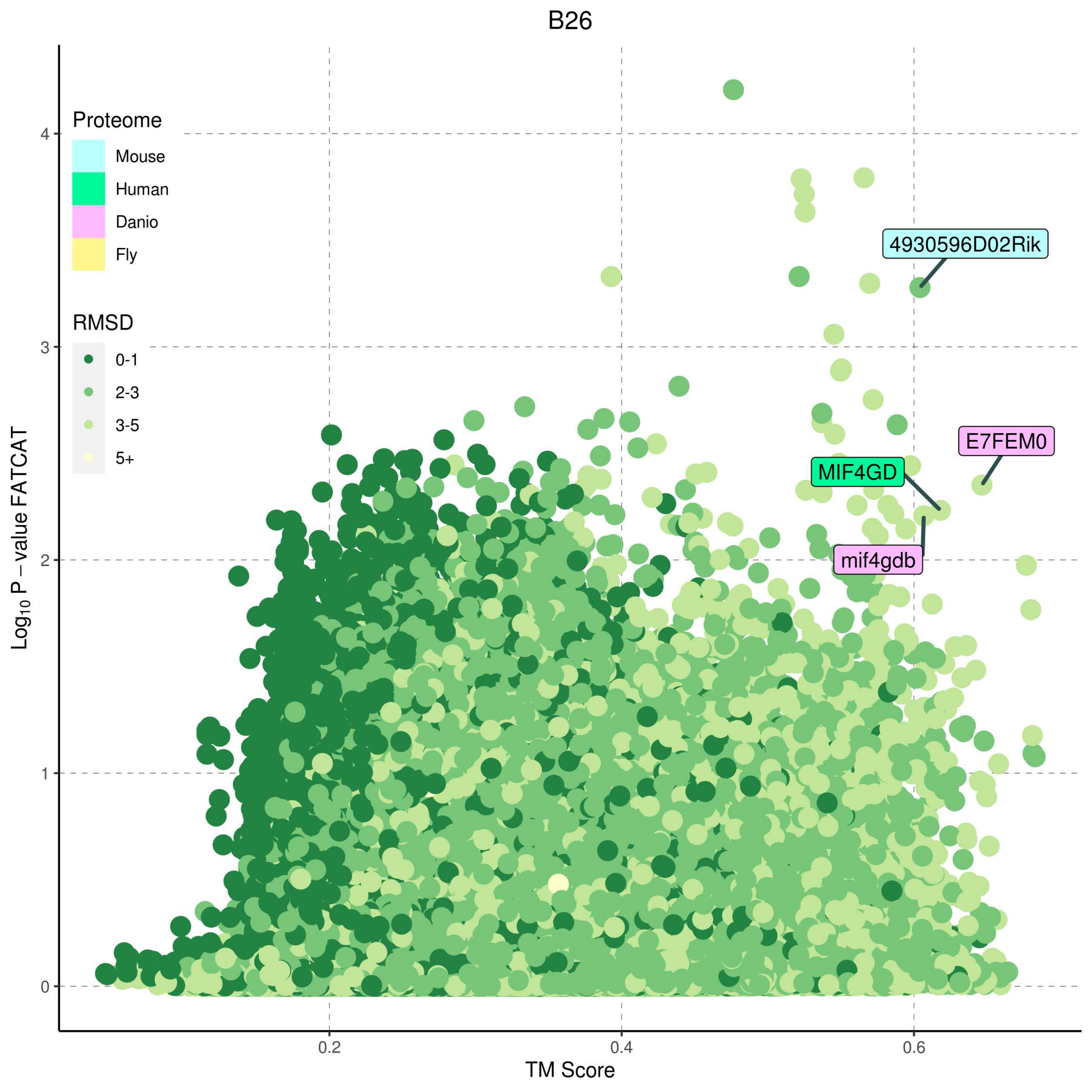
B26
Proteome
Mouse
Human
Danio
Fly
RMSD
0-1
2-3
3-5
5+
4930596D02Rik
E7FEM0
MIF4GD
mif4gdb
Log10 P − value FATCAT
TM Score
0
1
2
3
4
0.2
0.4
0.6

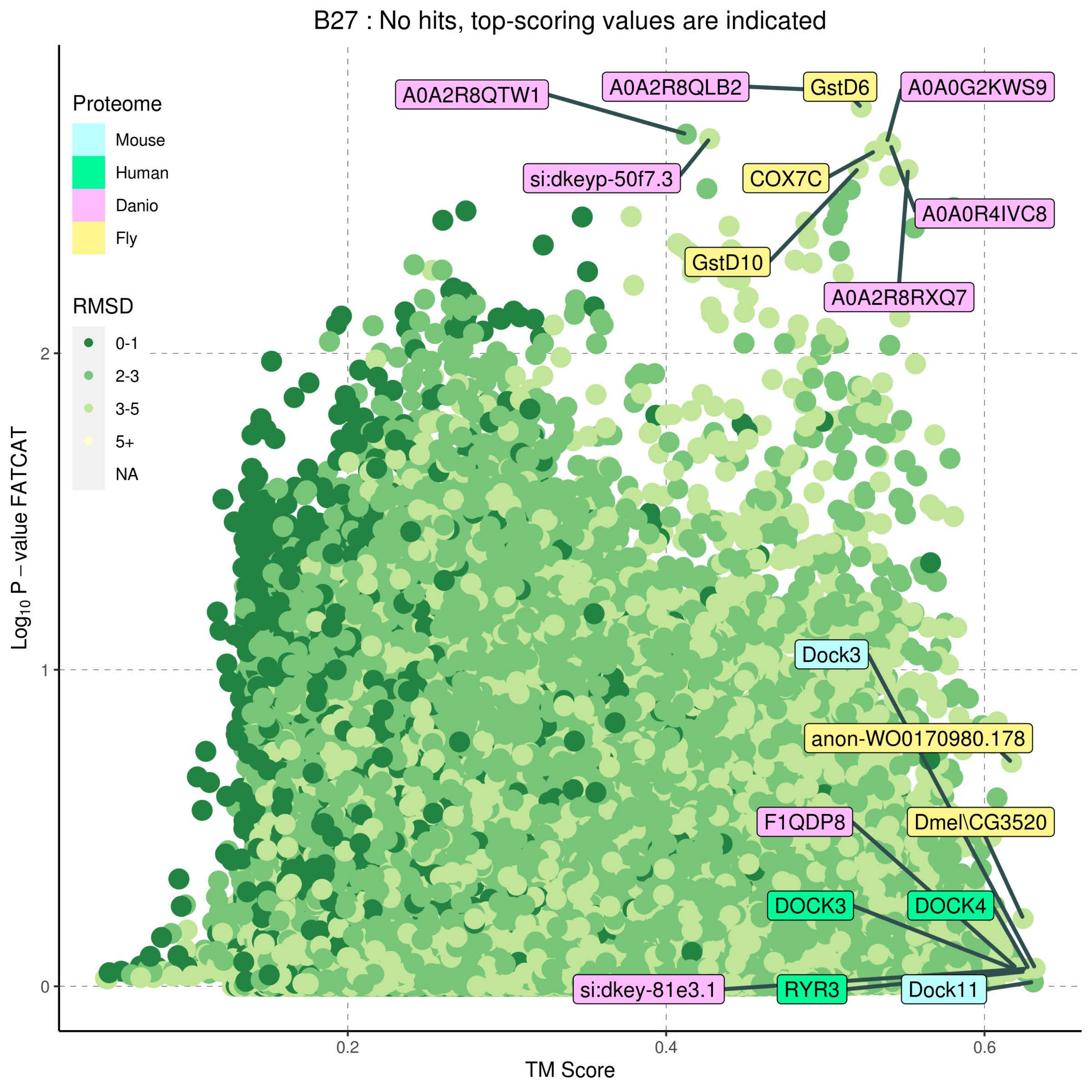
B27 : No hits, top-scoring values are indicated
Proteome
Mouse
Human
Danio
Fly
RMSD
0-1
2-3
3-5
5+
NA
A0A2R8QTW1
A0A2R8QLB2
GstD6
A0A0G2KWS9
si:dkeyp-50f7.3
COX7C
A0A0R4IVC8
GstD10
A0A2R8RXQ7
Dock3
anon-WO0170980.178
F1QDP8
Dmel\CG3520
DOCK3
DOCK4
si:dkey-81e3.1
RYR3
Dock11
Log10 P – value FATCAT
TM Score
0
1
2
0.2
0.4
0.6

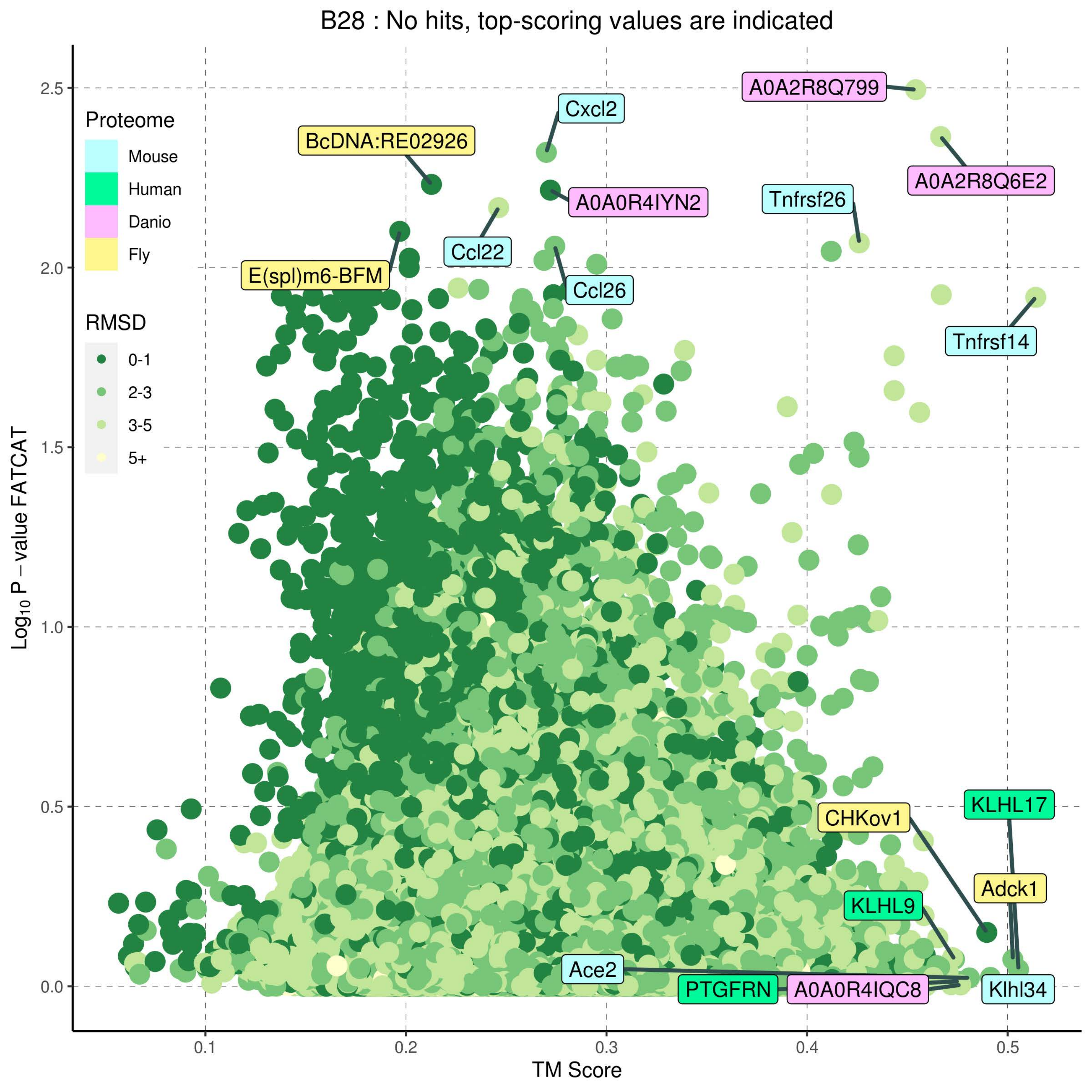
B28 : No hits, top-scoring values are indicated
Proteome
Mouse
Human
Danio
Fly
RMSD
0-1
2-3
3-5
5+
A0A2R8Q799
Cxcl2
BcDNA:RE02926
A0A2R8Q6E2
A0A0R4IYN2
Tnfrsf26
Ccl22
E(spl)m6-BFM
Ccl26
Tnfrsf14
KLHL17
CHKov1
Adck1
KLHL9
Ace2
PTGFRN
A0A0R4IQC8
Klhl34
Log10 P − value FATCAT
TM Score
0.0
0.5
1.0
1.5
2.0
2.5
0.1
0.2
0.3
0.4
0.5

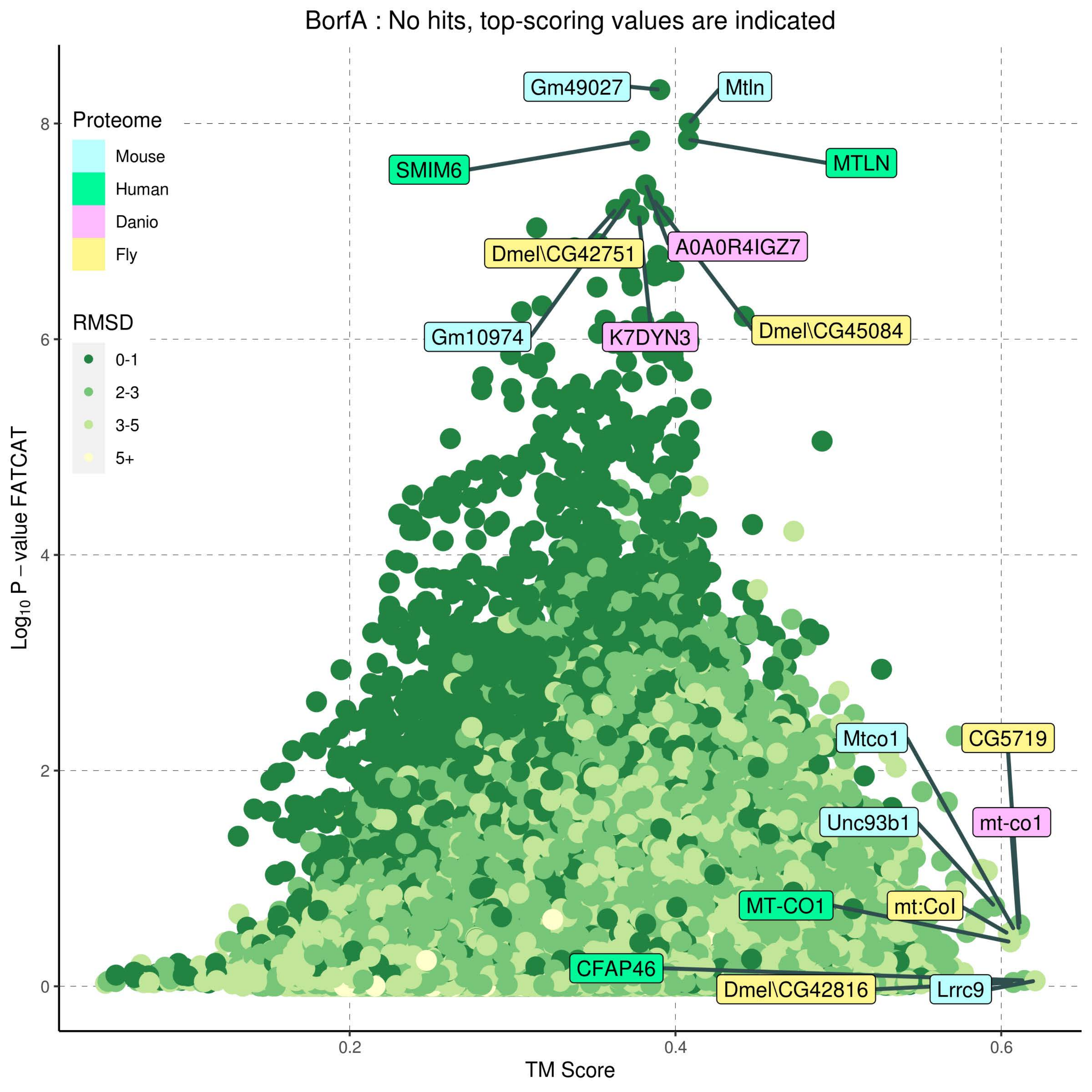

BorfA : No hits, top-scoring values are indicated
Proteome
Mouse
Human
Danio
Fly
RMSD
0-1
2-3
3-5
5+
Gm49027
Mtln
SMIM6
MTLN
Dmel\CG42751
A0A0R4IGZ7
Gm10974
K7DYN3
Dmel\CG45084
Mtco1
CG5719
Unc93b1
mt-co1
MT-CO1
mt:CoI
CFAP46
Dmel\CG42816
Lrrc9
Log10 P – value FATCAT
TM Score
0
2
4
6
8
0.2
0.4
0.6

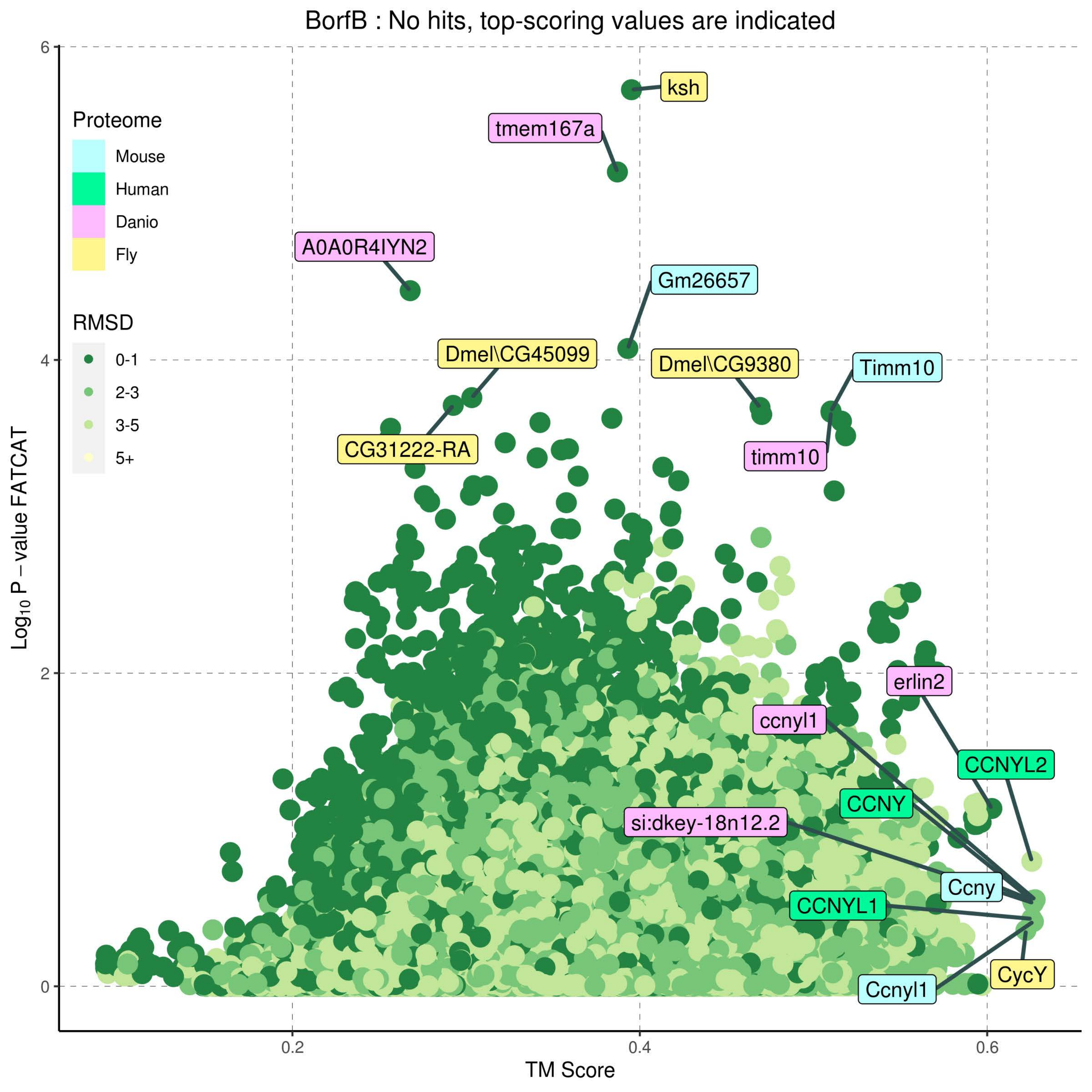
BorfB : No hits, top-scoring values are indicated
Proteome
Mouse
Human
Danio
Fly
RMSD
0-1
2-3
3-5
5+
ksh
tmem167a
A0A0R4IYN2
Gm26657
Dmel\CG45099
Dmel\CG9380
Timm10
CG31222-RA
timm10
erlin2
ccnyl1
CCNYL2
CCNY
si:dkey-18n12.2
Ccny
CCNYL1
CycY
Ccnyl1
Log_{10} P – value FATCAT
TM Score
0
2
4
6
0.2
0.4
0.6

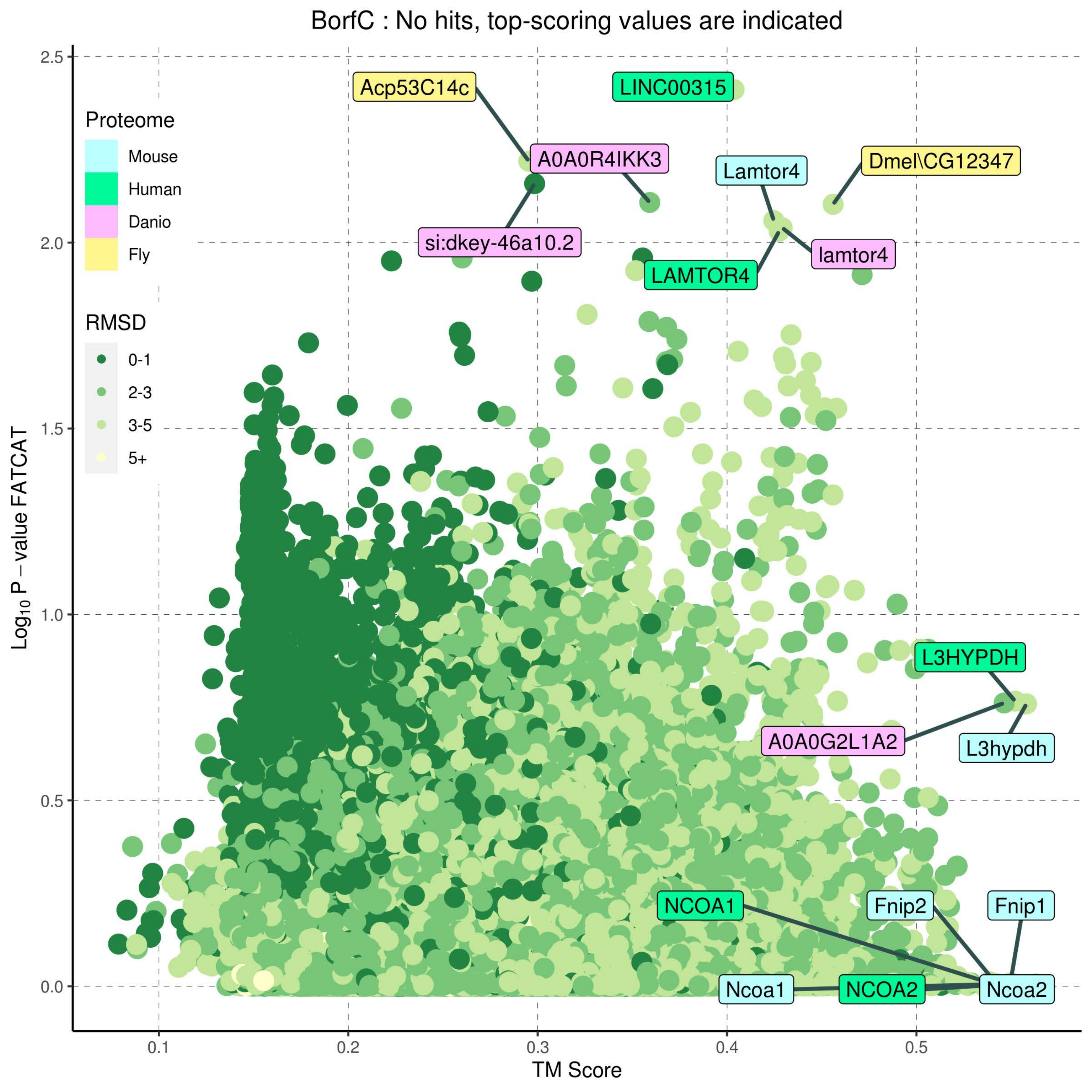
BorfC : No hits, top-scoring values are indicated
Proteome
Mouse
Human
Danio
Fly
RMSD
0-1
2-3
3-5
5+
Acp53C14c
LINC00315
A0A0R4IKK3
Lamtor4
Dmel\CG12347
si:dkey-46a10.2
lamtor4
LAMTOR4
L3HYPDH
A0A0G2L1A2
L3hypdh
NCOA1
Fnip2
Fnip1
Ncoa1
NCOA2
Ncoa2
Log10 P − value FATCAT
TM Score
0.0
0.5
1.0
1.5
2.0
2.5
0.1
0.2
0.3
0.4
0.5

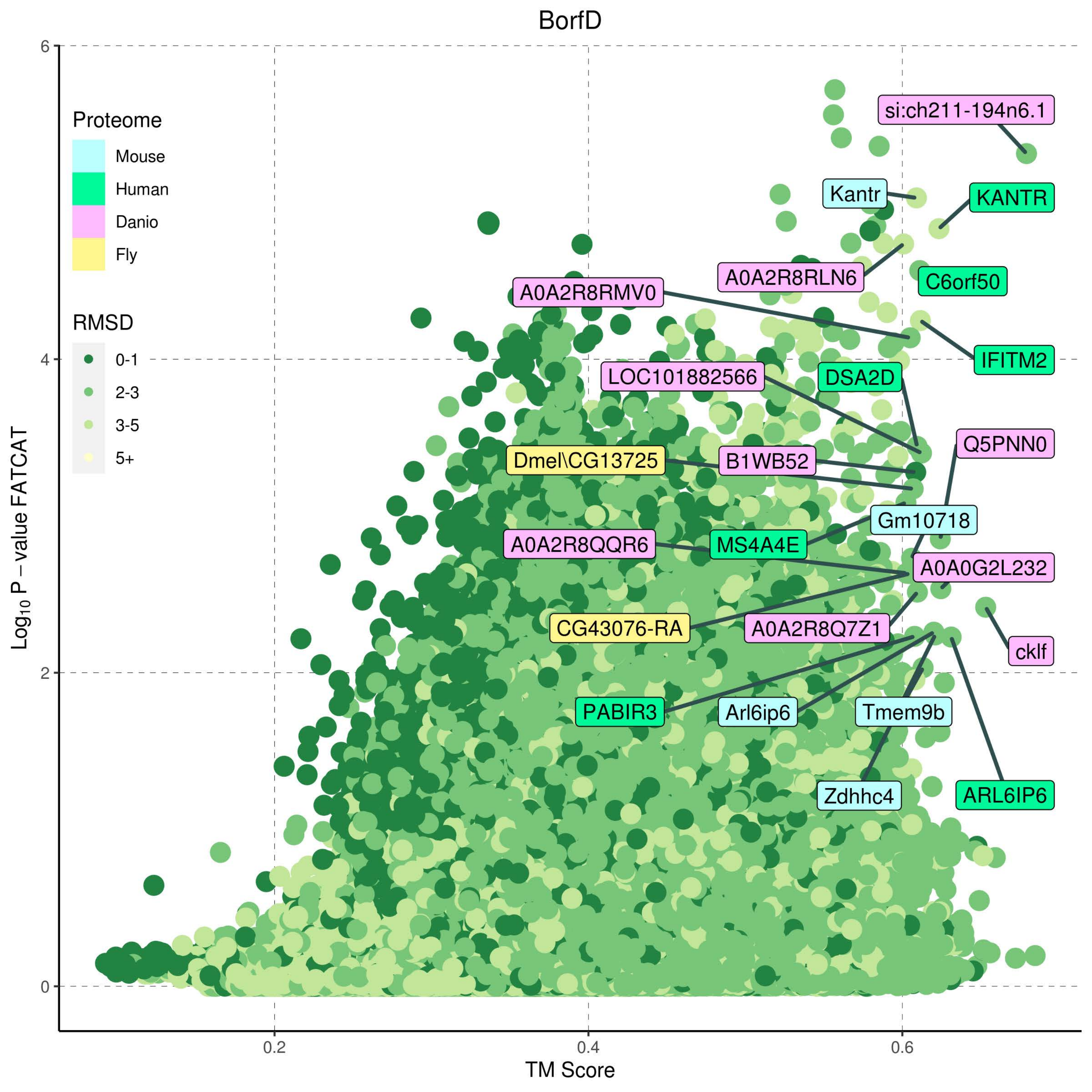
BorfD
Proteome
Mouse
Human
Danio
Fly
RMSD
0-1
2-3
3-5
5+
si:ch211-194n6.1
Kantr
KANTR
A0A2R8RLN6
C6orf50
A0A2R8RMV0
IFITM2
LOC101882566
DSA2D
Q5PNN0
Dmel\CG13725
B1WB52
Gm10718
A0A2R8QQR6
MS4A4E
A0A0G2L232
CG43076-RA
A0A2R8Q7Z1
cklf
PABIR3
Arl6ip6
Tmem9b
Zdhhc4
ARL6IP6
Log_{10} P − value FATCAT
TM Score
0
2
4
6
0.2
0.4
0.6

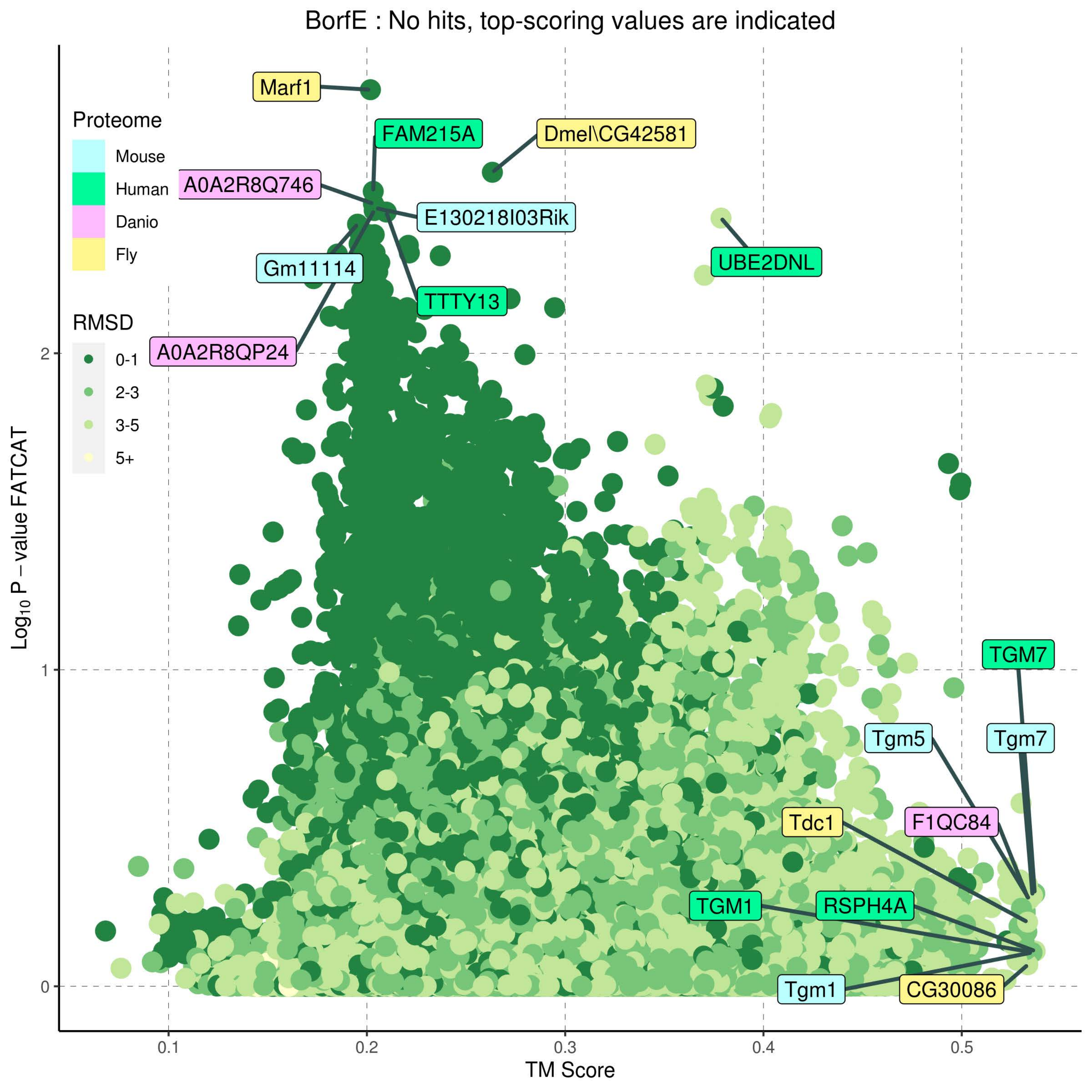

BorfE : No hits, top-scoring values are indicated
Proteome
Mouse
Human
Danio
Fly
RMSD
0-1
2-3
3-5
5+
Marf1
FAM215A
Dmel\CG42581
A0A2R8Q746
E130218I03Rik
Gm11114
UBE2DNL
TTTY13
A0A2R8QP24
TGM7
Tgm5
Tgm7
Tdc1
F1QC84
TGM1
RSPH4A
Tgm1
CG30086
Log10 P − value FATCAT
TM Score
0
1
2
0.1
0.2
0.3
0.4
0.5

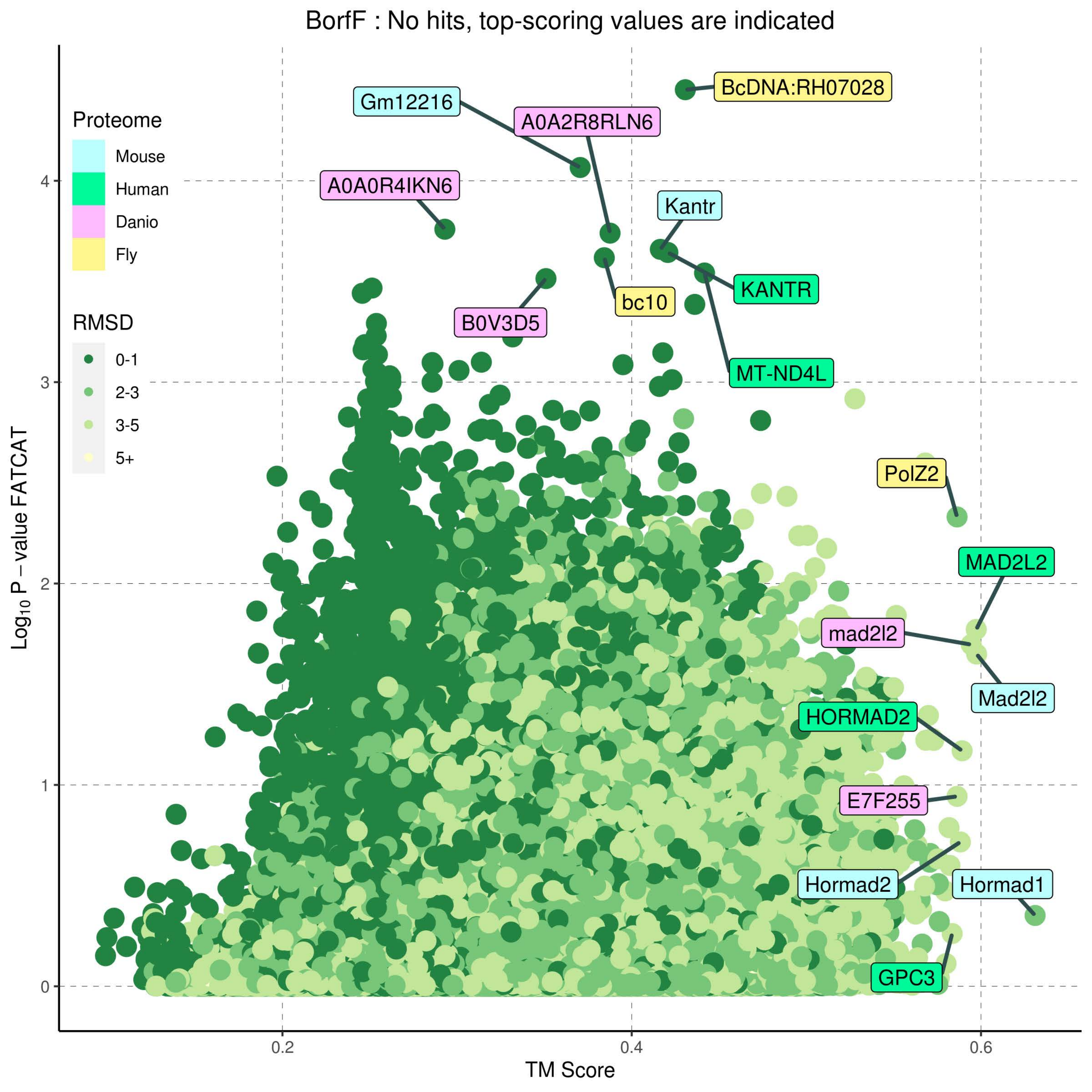

BorfF : No hits, top-scoring values are indicated
Proteome
Mouse
Human
Danio
Fly
RMSD
0-1
2-3
3-5
5+
BcDNA:RH07028
Gm12216
A0A2R8RLN6
A0A0R4IKN6
Kantr
KANTR
bc10
B0V3D5
MT-ND4L
PolZ2
MAD2L2
mad2l2
Mad2l2
HORMAD2
E7F255
Hormad2
Hormad1
GPC3
Log10 P – value FATCAT
TM Score
0
1
2
3
4
0.2
0.4
0.6

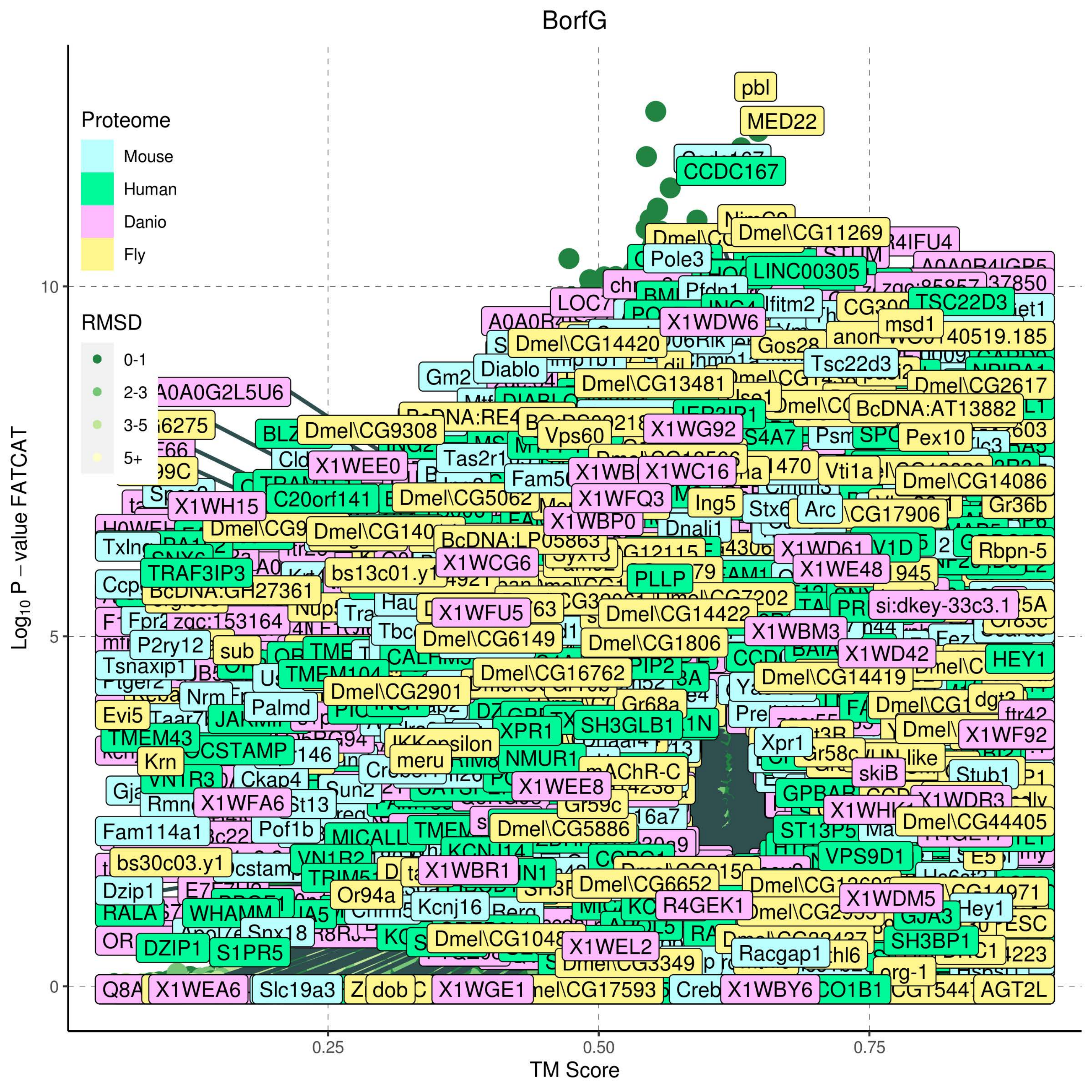
BorfG
Proteome
Mouse
Human
Danio
Fly
RMSD
0-1
2-3
3-5
5+
Log10 P − value FATCAT
TM Score
0
5
10
0.25
0.50
0.75
pbl
MED22
CCDC167
Dmel\CG11269
Pole3
LINC00305
Pfdn1
Ifitm2
TSC22D3
X1WDW6
msd1
Gos28
Dmel\CG14420
Tsc22d3
Diablo
Dmel\CG13481
Dmel\CG2617
BcDNA:AT13882
A0A0G2L5U6
Dmel\CG9308
X1WG92
Vps60
Pex10
X1WEE0
X1WC16
Vti1a
Dmel\CG14086
C20orf141
Dmel\CG5062
X1WFQ3
Ing5
Arc
Gr36b
X1WH15
X1WBP0
Rbpn-5
TRAF3IP3
bs13c01.y1
X1WCG6
X1WE48
PLLP
BcDNA:GH27361
si:dkey-33c3.1
zgc:153164
X1WFU5
Dmel\CG14422
X1WBM3
P2ry12
sub
Dmel\CG6149
Dmel\CG1806
X1WD42
HEY1
Tsnaxip1
Dmel\CG16762
Dmel\CG14419
Dmel\CG2901
Palmd
Evi5
SH3GLB1
XPR1
TMEM43
Xpr1
X1WF92
Krn
meru
NMUR1
mAChR-C
skiB
Stub1
Ckap4
X1WEE8
X1WFA6
X1WDR3
Gr59c
Dmel\CG44405
Fam114a1
Pof1b
Dmel\CG5886
ST13P5
bs30c03.y1
VPS9D1
X1WBR1
Dmel\CG6652
Dzip1
Or94a
Kcnj16
R4GEK1
X1WDM5
WHAMM
Hey1
Snx18
SH3BP1
Dmel\CG1048
X1WEL2
Racgap1
DZIP1
S1PR5
org-1
X1WEA6
Slc19a3
dob
X1WGE1
X1WBY6
AGT2L

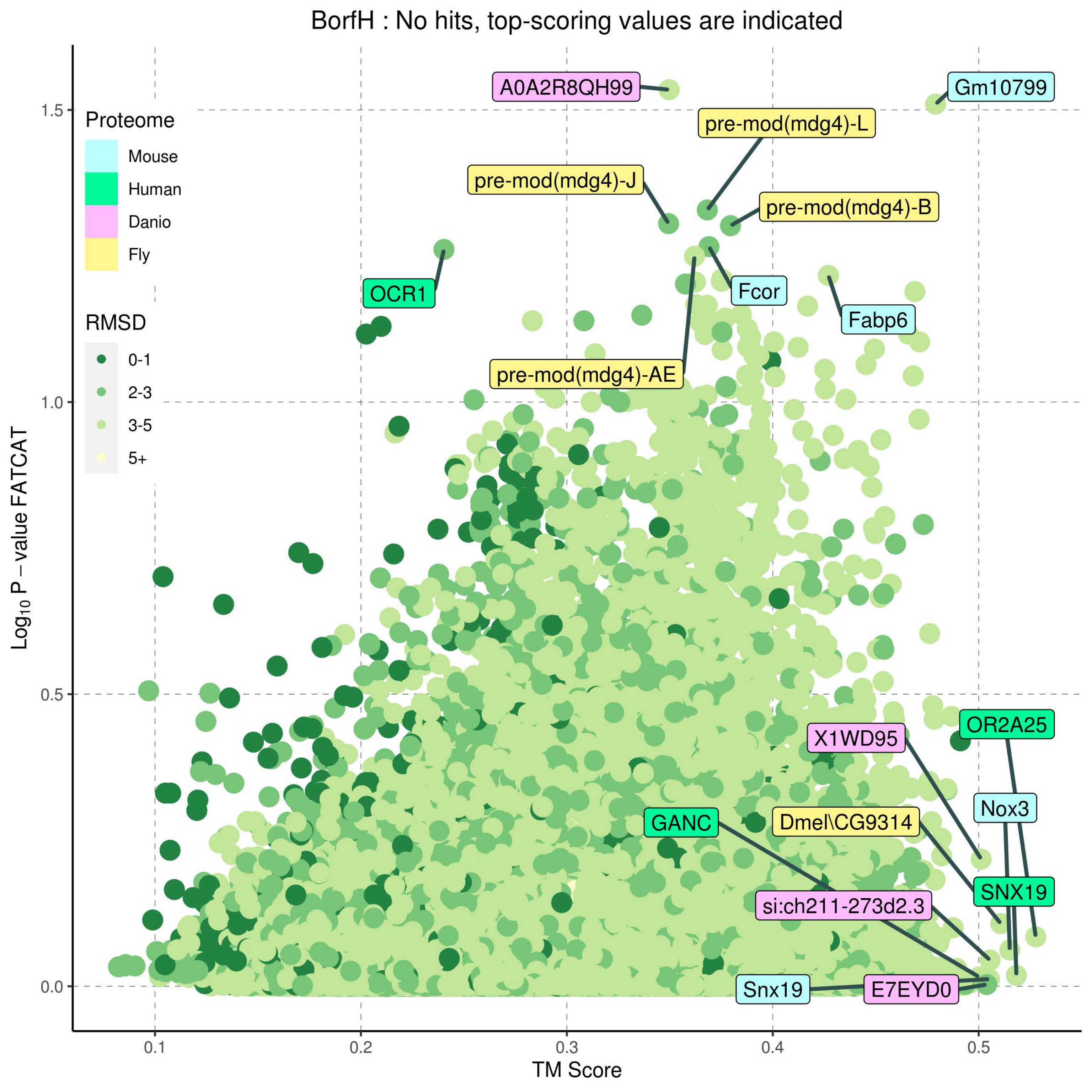

BorfH : No hits, top-scoring values are indicated
Proteome
Mouse
Human
Danio
Fly
RMSD
0-1
2-3
3-5
5+
A0A2R8QH99
Gm10799
pre-mod(mdg4)-L
pre-mod(mdg4)-J
pre-mod(mdg4)-B
OCR1
Fcor
Fabp6
pre-mod(mdg4)-AE
X1WD95
OR2A25
Nox3
GANC
Dmel\CG9314
SNX19
si:ch211-273d2.3
Snx19
E7EYD0
Log10 P − value FATCAT
TM Score
0.0
0.5
1.0
1.5
0.1
0.2
0.3
0.4
0.5

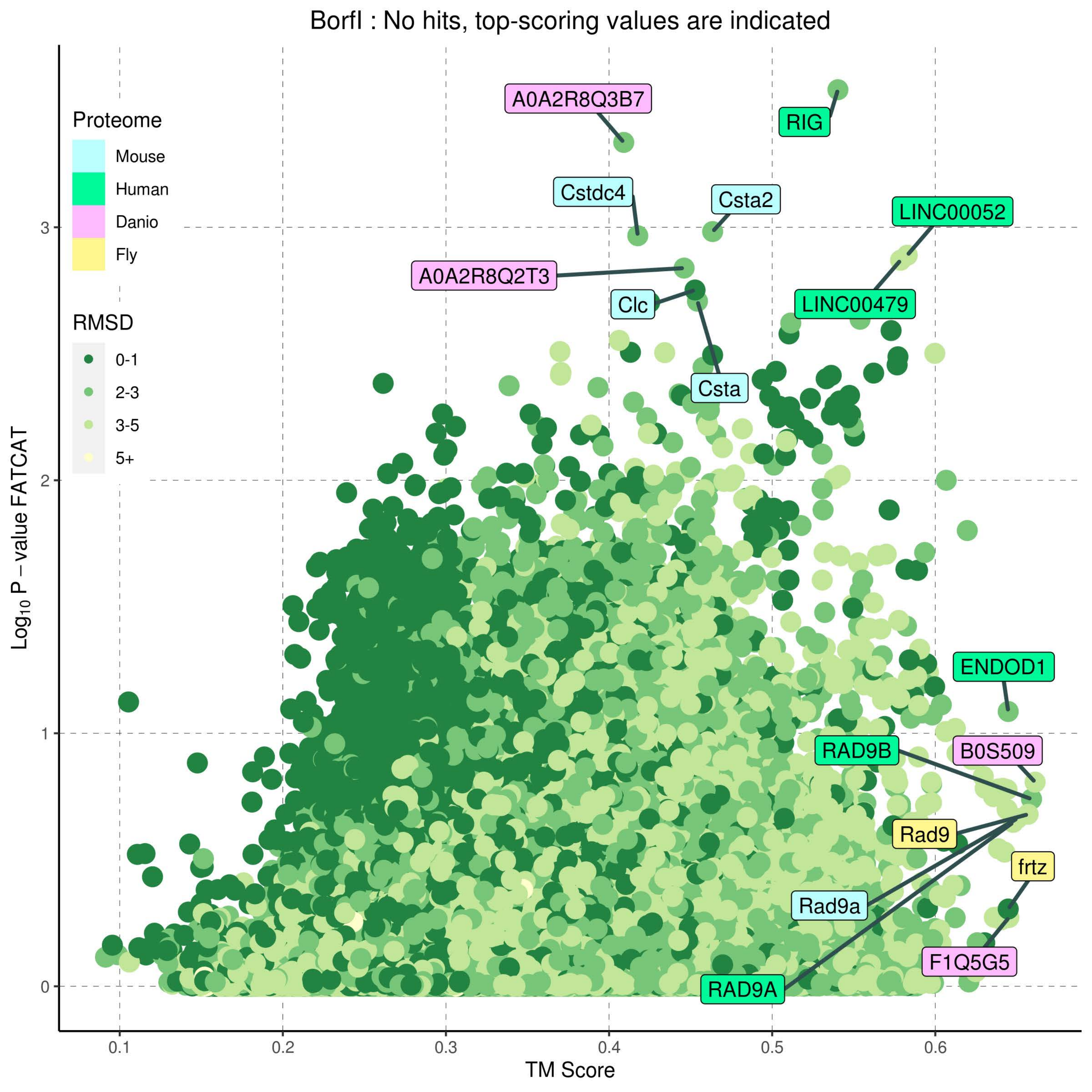
BorfI : No hits, top-scoring values are indicated
Proteome
Mouse
Human
Danio
Fly
RMSD
0-1
2-3
3-5
5+
A0A2R8Q3B7
RIG
Cstdc4
Csta2
LINC00052
A0A2R8Q2T3
Clc
LINC00479
Csta
ENDOD1
RAD9B
B0S509
Rad9
frtz
Rad9a
F1Q5G5
RAD9A
Log10 P − value FATCAT
TM Score
0
1
2
3
0.1
0.2
0.3
0.4
0.5
0.6

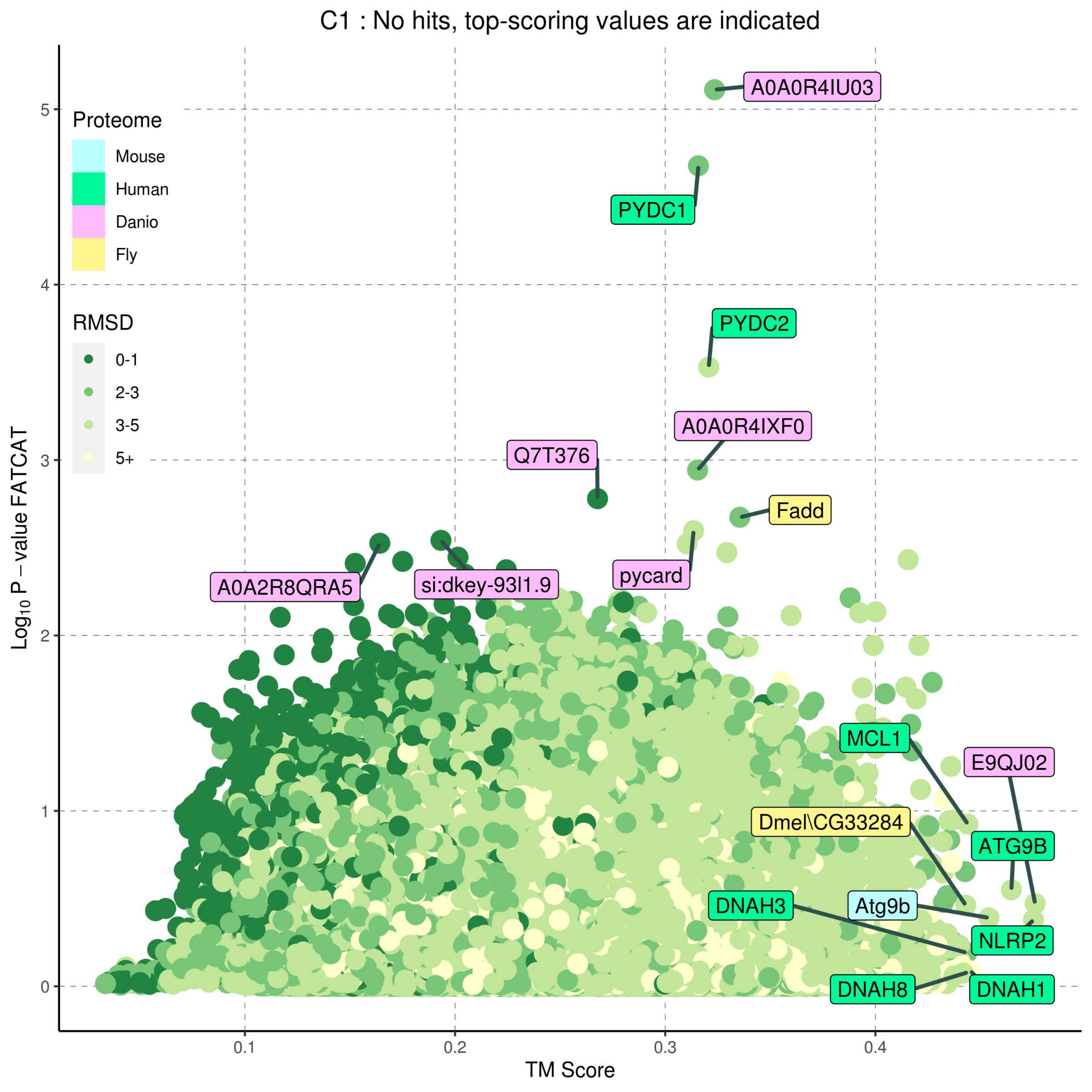

C1 : No hits, top-scoring values are indicated
Proteome
Mouse
Human
Danio
Fly
RMSD
0-1
2-3
3-5
5+
Log10 P − value FATCAT
TM Score
0
1
2
3
4
5
0.1
0.2
0.3
0.4
A0A0R4IU03
PYDC1
PYDC2
A0A0R4IXF0
Q7T376
Fadd
pycard
A0A2R8QRA5
si:dkey-93l1.9
MCL1
E9QJ02
Dmel\CG33284
ATG9B
DNAH3
Atg9b
NLRP2
DNAH8
DNAH1

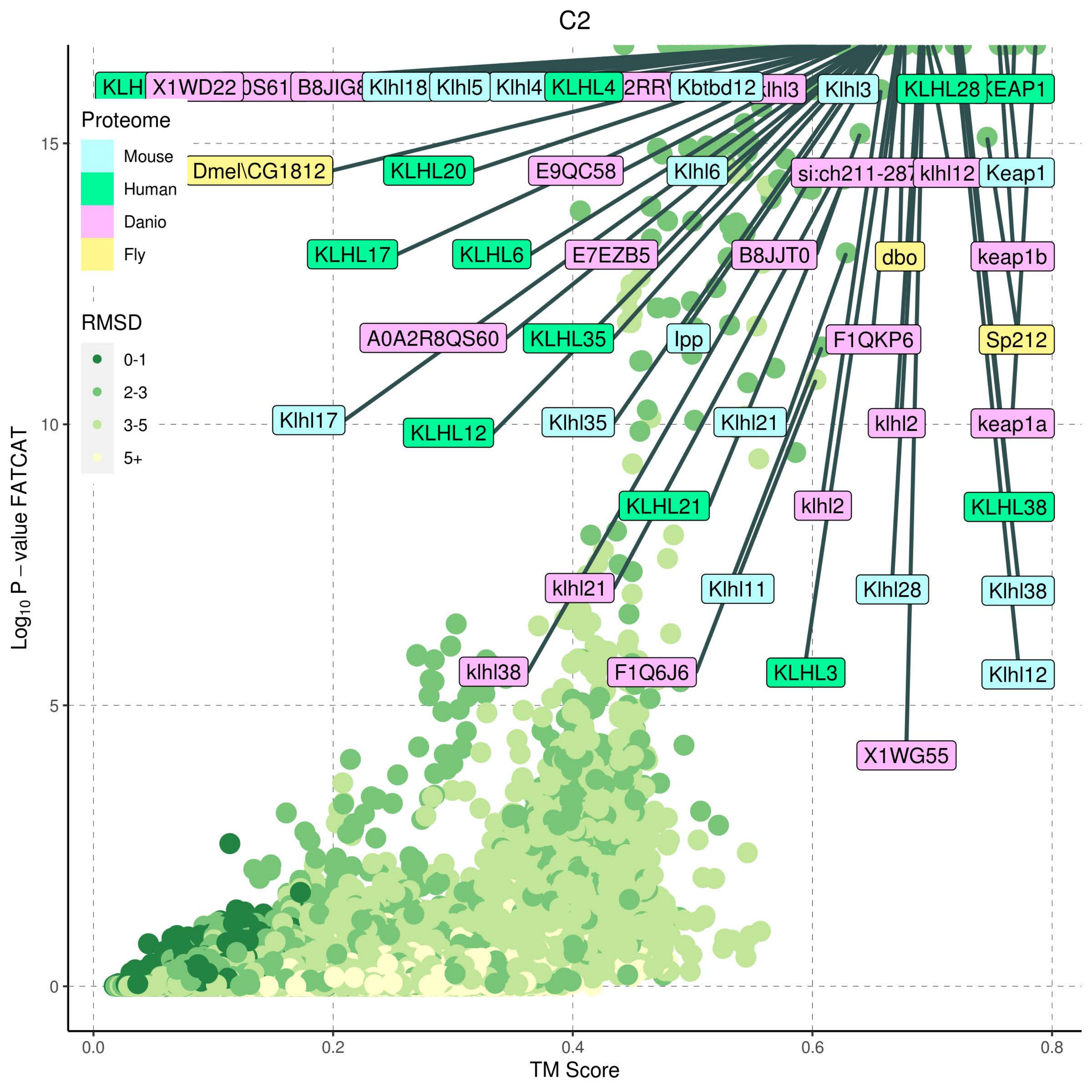
C2
Proteome
Mouse
Human
Danio
Fly
RMSD
0-1
2-3
3-5
5+
Log10 P − value FATCAT
TM Score
0.0
0.2
0.4
0.6
0.8
0
5
10
15
X1WD22
B8JIG8
Klhl18
Klhl5
Klhl4
KLHL4
Kbtbd12
Klhl3
KLHL28
Dmel\CG1812
KLHL20
E9QC58
Klhl6
klhl12
Keap1
KLHL17
KLHL6
E7EZB5
B8JJT0
dbo
keap1b
A0A2R8QS60
KLHL35
lpp
F1QKP6
Sp212
Klhl17
KLHL12
Klhl35
Klhl21
klhl2
keap1a
KLHL21
klhl2
KLHL38
klhl21
Klhl11
Klhl28
Klhl38
klhl38
F1Q6J6
KLHL3
Klhl12
X1WG55

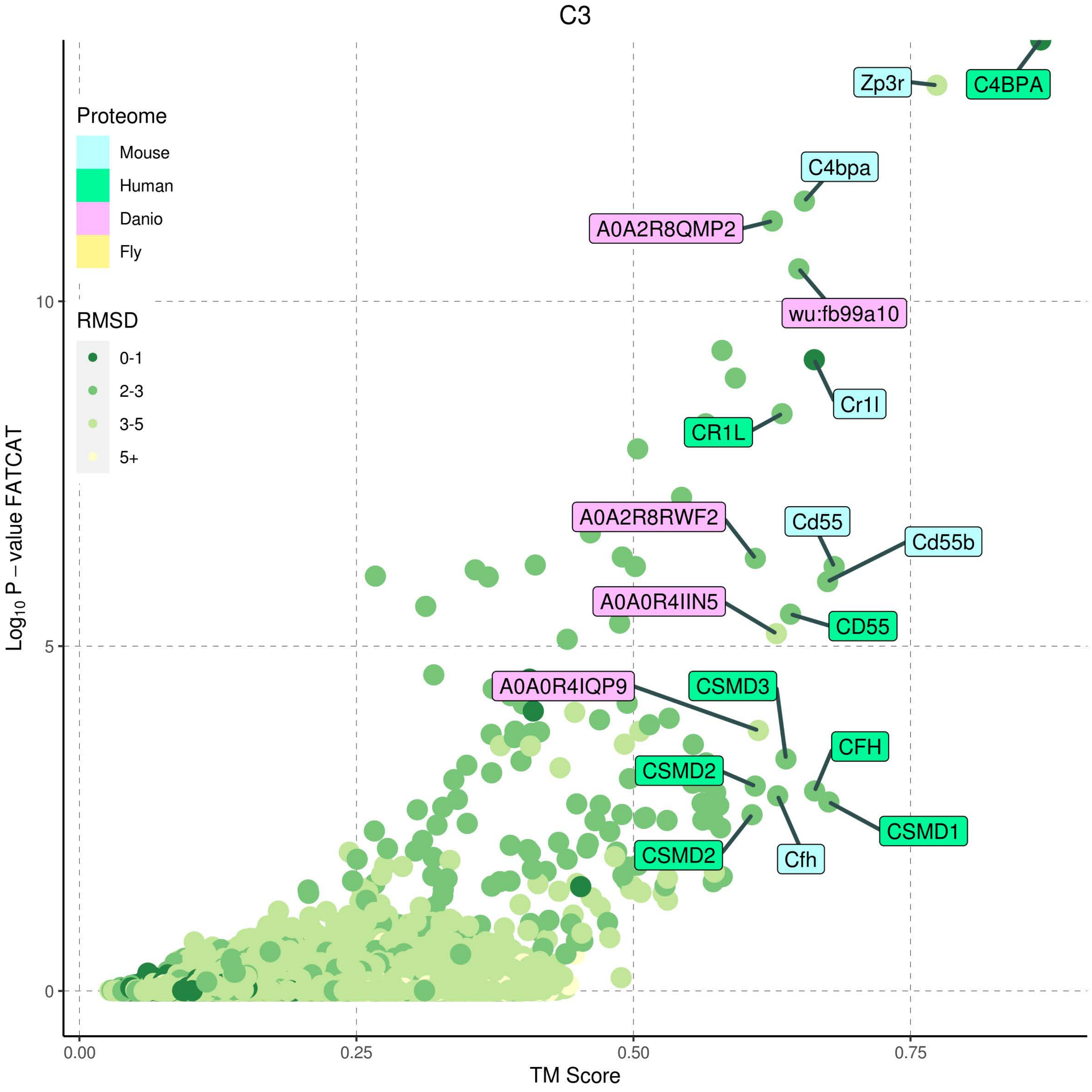

C3
Proteome
Mouse
Human
Danio
Fly
RMSD
0-1
2-3
3-5
5+
Zp3r
C4BPA
C4bpa
A0A2R8QMP2
wu:fb99a10
Cr1l
CR1L
A0A2R8RWF2
Cd55
Cd55b
A0A0R4IIN5
CD55
A0A0R4IQP9
CSMD3
CFH
CSMD2
CSMD1
CSMD2
Cfh
Log10 P − value FATCAT
10
5
0
0.00
0.25
0.50
0.75
TM Score

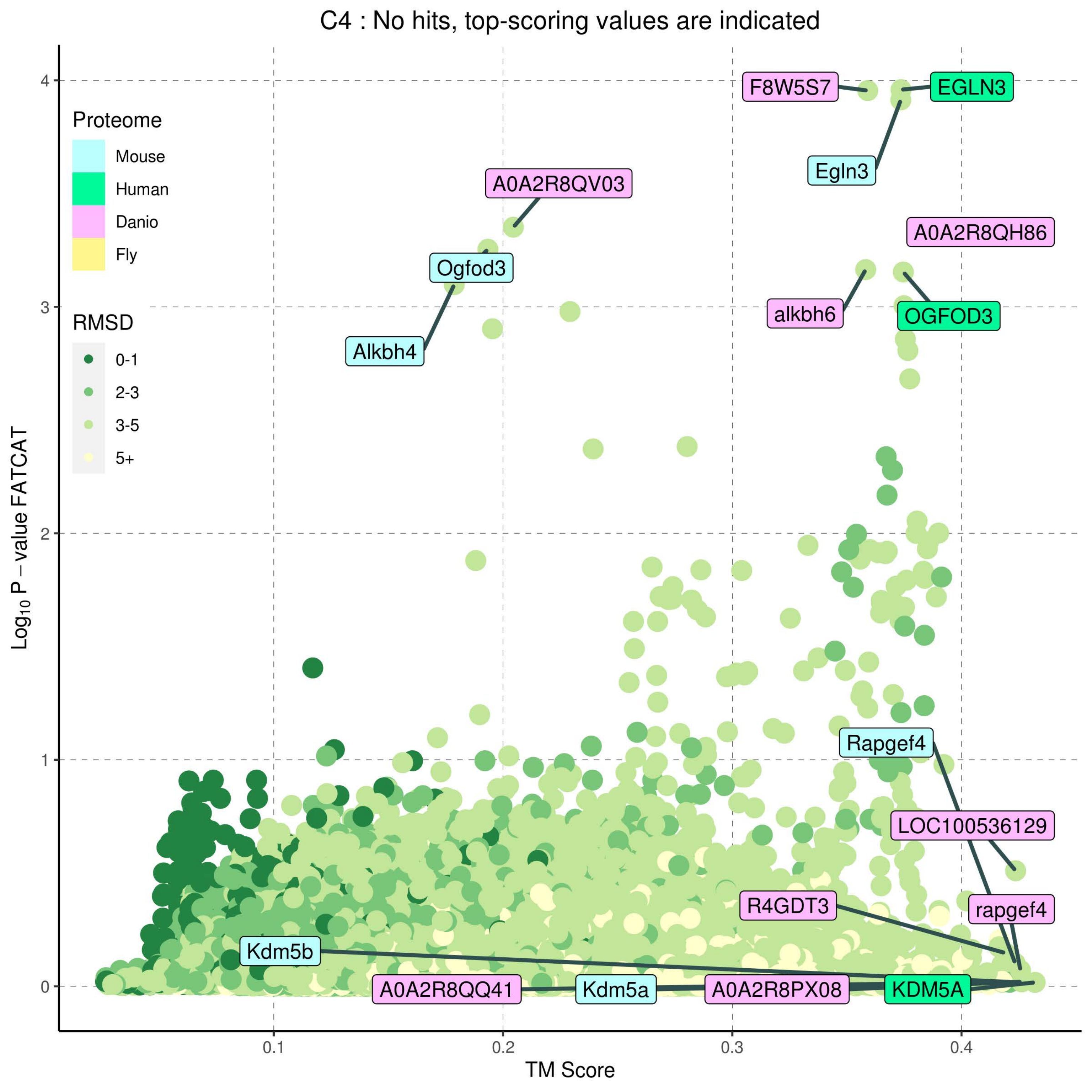

C4 : No hits, top-scoring values are indicated
Proteome
Mouse
Human
Danio
Fly
RMSD
0-1
2-3
3-5
5+
F8W5S7
EGLN3
Egln3
A0A2R8QV03
A0A2R8QH86
Ogfod3
alkbh6
OGFOD3
Alkbh4
Rapgef4
LOC100536129
R4GDT3
rapgef4
Kdm5b
A0A2R8QQ41
Kdm5a
A0A2R8PX08
KDM5A
Log10 P – value FATCAT
TM Score
0
1
2
3
4
0.1
0.2
0.3
0.4

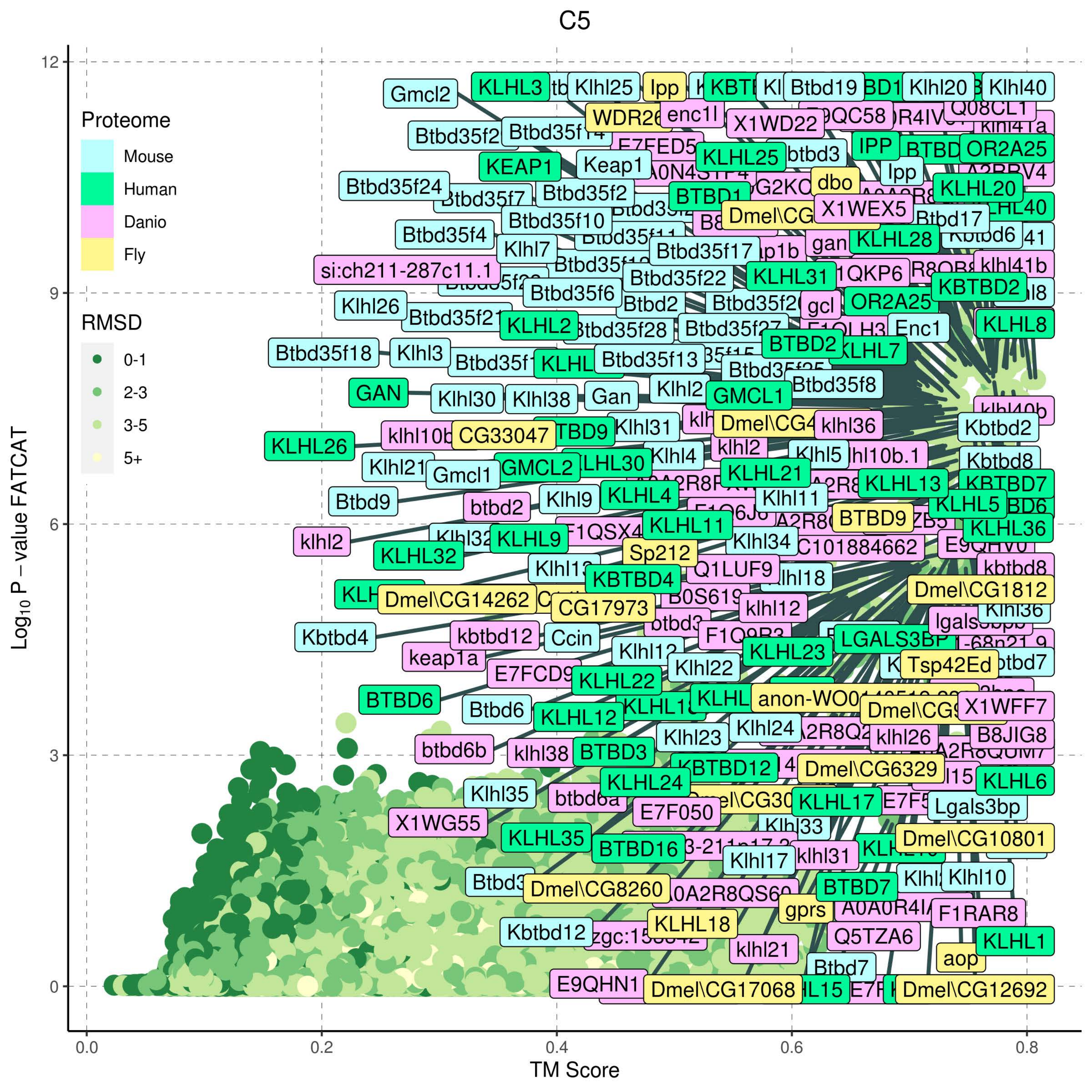

C5
Proteome
Mouse
Human
Danio
Fly
RMSD
0-1
2-3
3-5
5+
Log10 P – value FATCAT
TM Score
0.0
0.2
0.4
0.6
0.8
0
3
6
9
12
Gmcl2
KLHL3
Klhl25
Ipp
Btbd19
Klhl20
Klhl40
WDR26
enc1l
X1WD22
Q08CL1
Btbd35f2
E7FED5
KLHL25
btbd3
IPP
OR2A25
KEAP1
Keap1
Ipp
Btbd35f24
Btbd35f7
Btbd35f2
BTBD1
dbo
KLHL20
X1WEX5
Btbd35f10
Btbd17
Btbd35f4
KLHL28
Klhl7
gan
Btbd35f17
si:ch211-287c11.1
KLHL31
klhl41b
Btbd35f22
KBTBD2
Btbd35f6
Klhl26
Btbd2
gcl
OR2A25
Btbd35f21
KLHL2
Btbd35f28
Enc1
KLHL8
Btbd35f18
Klhl3
BTBD2
KLHL7
Btbd35f13
Btbd35f8
GAN
Klhl30
Klhl38
Gan
Klhl2
GMCL1
klhl10b
CG33047
Klhl31
klhl36
Kbtbd2
KLHL26
Klhl4
klhl2
Klhl5
Klhl21
Gmcl1
GMCL2
KLHL30
KLHL21
KLHL13
Kbtbd8
KBTBD7
Btbd9
btbd2
Klhl9
KLHL4
Klhl11
KLHL5
BTBD9
KLHL11
KLHL36
klhl2
KLHL9
Klhl34
KLHL32
Sp212
C101884662
Q1LUF9
KBTBD4
kbtbd8
Dmel\CG14262
CG17973
klhl12
Dmel\CG1812
Klhl18
Klhl36
Kbtbd4
kbtbd12
Ccin
keap1a
LGALS3BP
KLHL23
Klhl22
E7FCD9
KLHL22
Tsp42Ed
BTBD6
Btbd6
KLHL12
X1WFF7
klhl26
B8JIG8
btbd6b
klhl38
BTBD3
Klhl23
Klhl24
KBTBD12
Dmel\CG6329
KLHL24
KLHL6
Klhl35
btbd6a
E7F050
KLHL17
Lgals3bp
X1WG55
Klhl33
KLHL35
BTBD16
Dmel\CG10801
Klhl17
klhl31
Klhl10
Dmel\CG8260
BTBD7
gprs
F1RAR8
Kbtbd12
KLHL18
Q5TZA6
KLHL1
klhl21
aop
Btbd7
E9QHN1
Dmel\CG17068
Dmel\CG12692

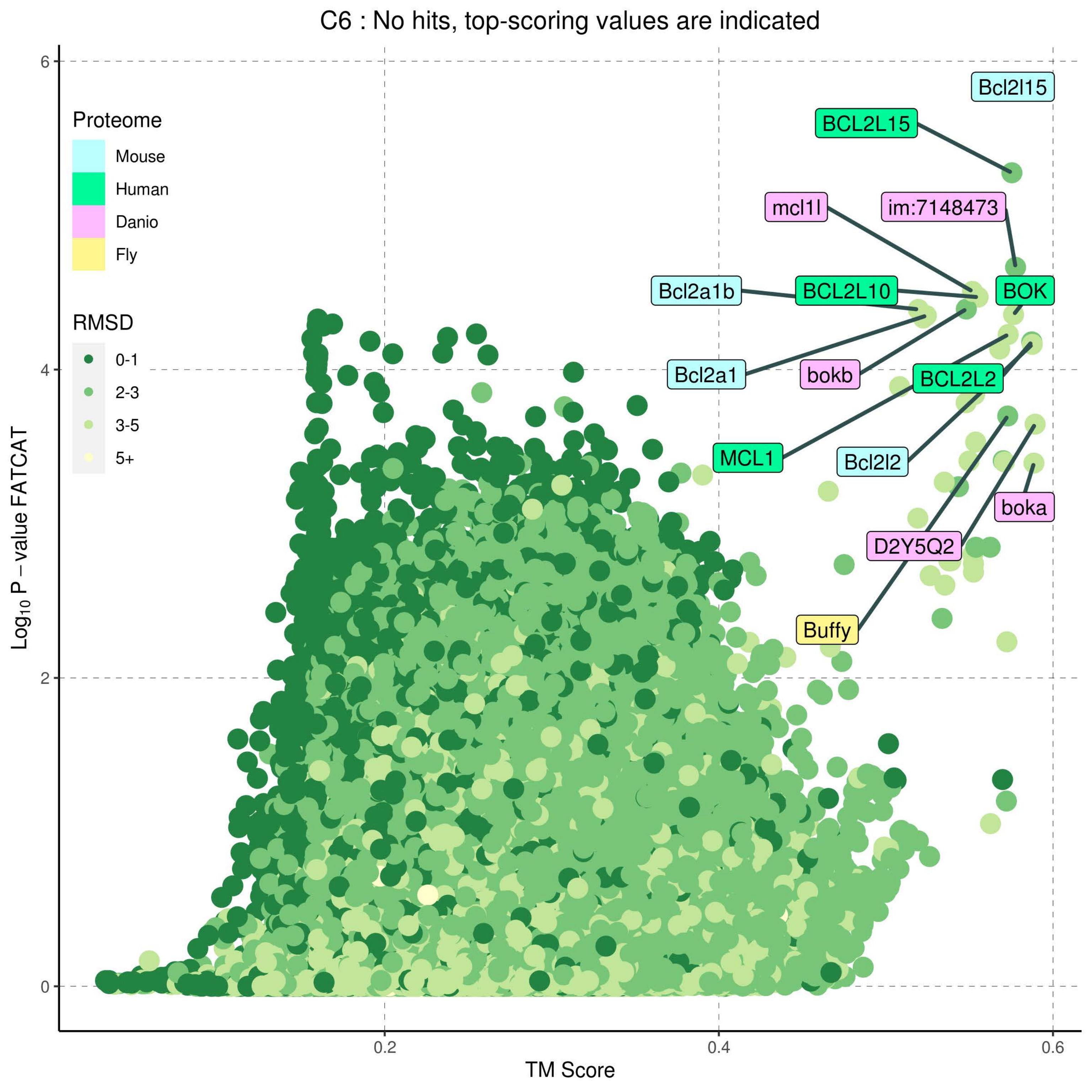

C6 : No hits, top-scoring values are indicated
Proteome
Mouse
Human
Danio
Fly
RMSD
0-1
2-3
3-5
5+
Bcl2l15
BCL2L15
mcl1l
im:7148473
Bcl2a1b
BCL2L10
BOK
Bcl2a1
bokb
BCL2L2
MCL1
Bcl2l2
boka
D2Y5Q2
Buffy
Log_{10} P – value FATCAT
TM Score
0
2
4
6
0.2
0.4
0.6

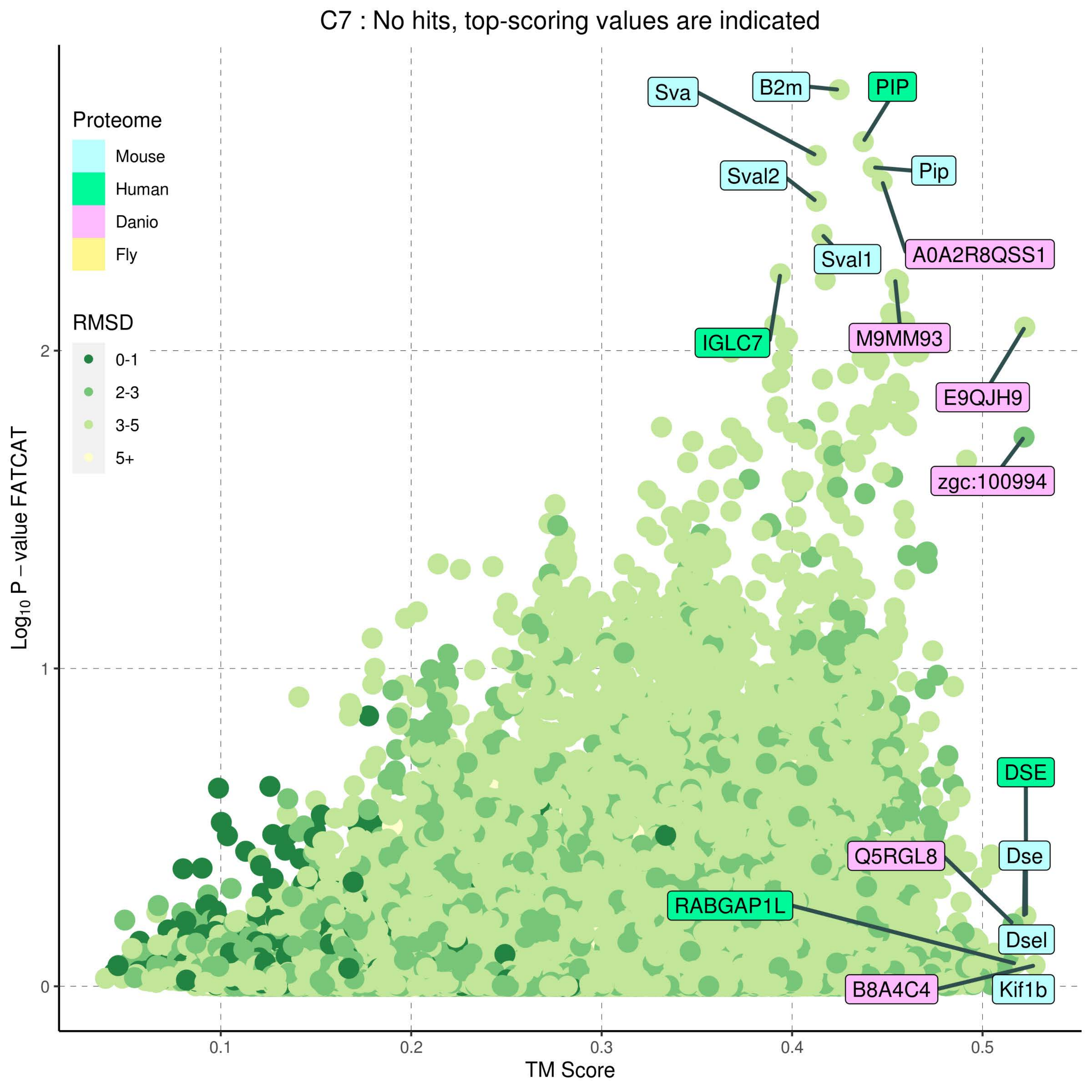

C7 : No hits, top-scoring values are indicated
Proteome
Mouse
Human
Danio
Fly
RMSD
0-1
2-3
3-5
5+
Log10 P – value FATCAT
TM Score
0
1
2
0.1
0.2
0.3
0.4
0.5
Sva
B2m
PIP
Pip
Sval2
Sval1
A0A2R8QSS1
IGLC7
M9MM93
E9QJH9
zgc:100994
DSE
Dse
Q5RGL8
RABGAP1L
Dsel
B8A4C4
Kif1b

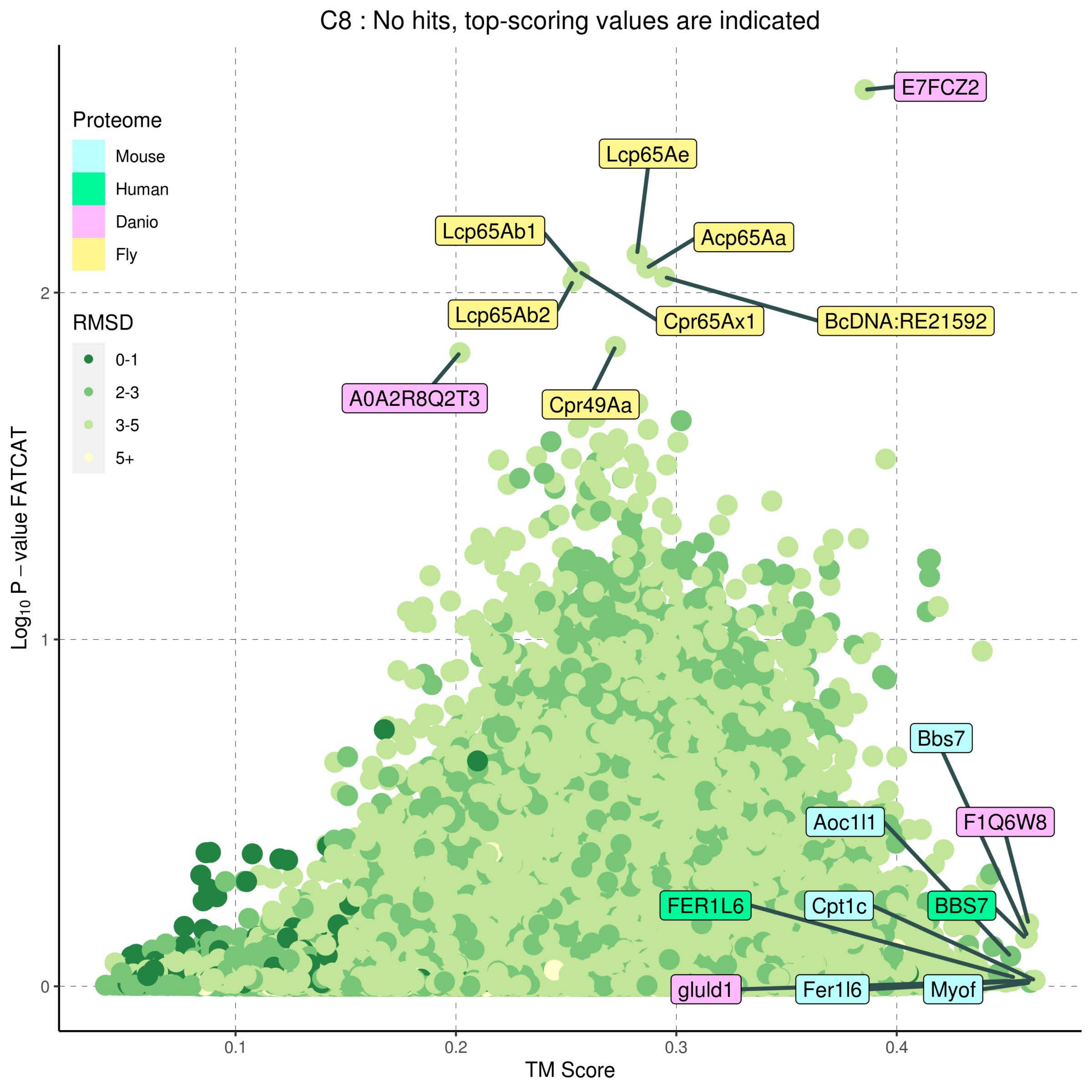

C8 : No hits, top-scoring values are indicated
Proteome
Mouse
Human
Danio
Fly
RMSD
0-1
2-3
3-5
5+
E7FCZ2
Lcp65Ae
Lcp65Ab1
Acp65Aa
Lcp65Ab2
Cpr65Ax1
BcDNA:RE21592
A0A2R8Q2T3
Cpr49Aa
Bbs7
Aoc1l1
F1Q6W8
FER1L6
Cpt1c
BBS7
gluld1
Fer1l6
Myof
2
1
0
0.1
0.2
0.3
0.4
TM Score
Log10 P – value FATCAT

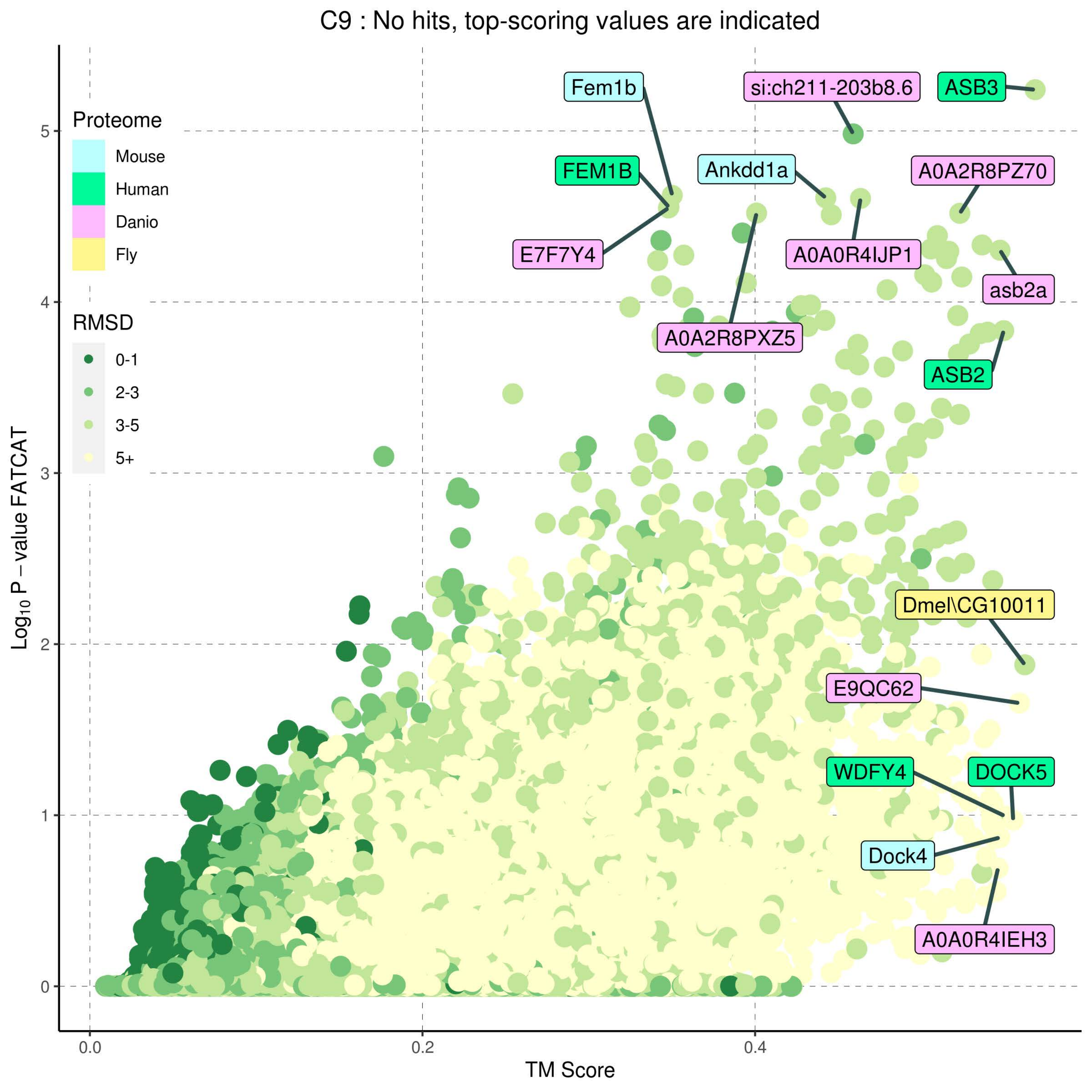
C9 : No hits, top-scoring values are indicated
Proteome
Mouse
Human
Danio
Fly
RMSD
0-1
2-3
3-5
5+
Fem1b
si:ch211-203b8.6
ASB3
FEM1B
Ankdd1a
A0A2R8PZ70
E7F7Y4
A0A0R4IJP1
asb2a
A0A2R8PXZ5
ASB2
Dmel\CG10011
E9QC62
WDFY4
DOCK5
Dock4
A0A0R4IEH3
Log_{10} P − value FATCAT
TM Score
0.0
0.2
0.4
0
1
2
3
4
5

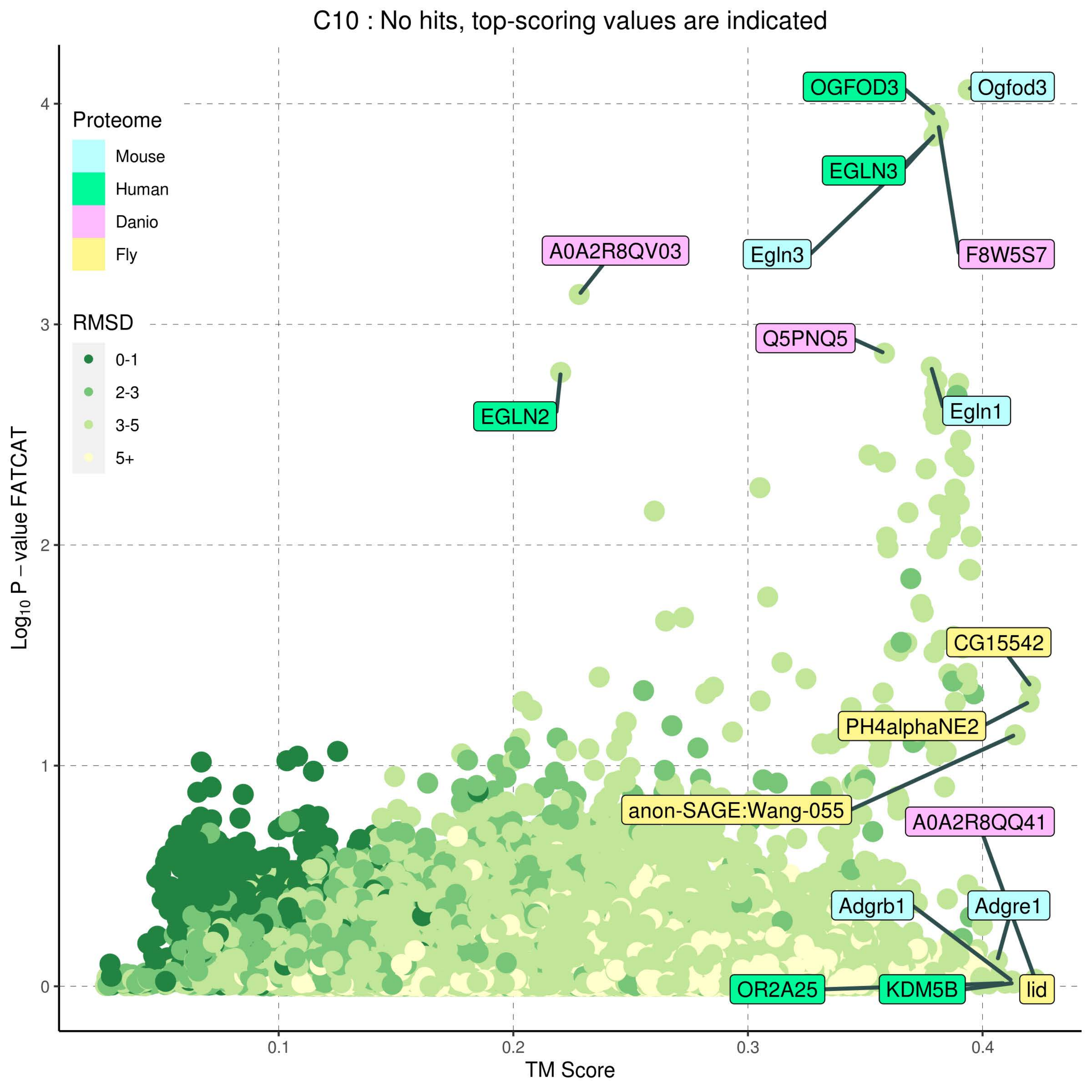

C10 : No hits, top-scoring values are indicated
Proteome
Mouse
Human
Danio
Fly
RMSD
0-1
2-3
3-5
5+
OGFOD3
Ogfod3
EGLN3
Egln3
F8W5S7
A0A2R8QV03
Q5PNQ5
EGLN2
Egln1
CG15542
PH4alphaNE2
anon-SAGE:Wang-055
A0A2R8QQ41
Adgrb1
Adgre1
OR2A25
KDM5B
lid
Log10 P − value FATCAT
TM Score
0
1
2
3
4
0.1
0.2
0.3
0.4

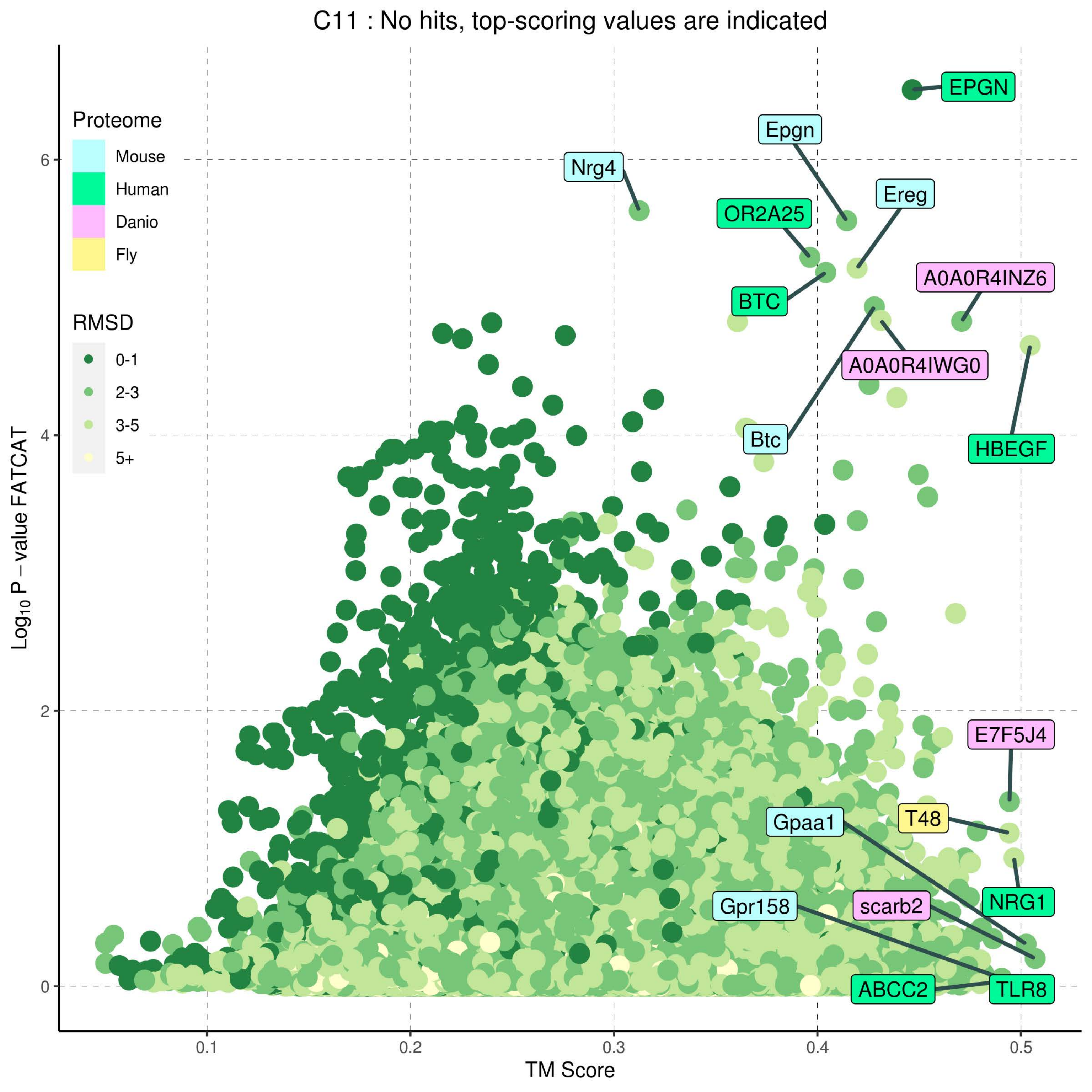

C11 : No hits, top-scoring values are indicated
Proteome
Mouse
Human
Danio
Fly
RMSD
0-1
2-3
3-5
5+
EPGN
Epgn
Nrg4
Ereg
OR2A25
A0A0R4INZ6
BTC
A0A0R4IWG0
Btc
HBEGF
E7F5J4
T48
Gpaa1
NRG1
Gpr158
scarb2
ABCC2
TLR8
6
4
2
0
0.1
0.2
0.3
0.4
0.5
TM Score
Log_{10} P − value FATCAT

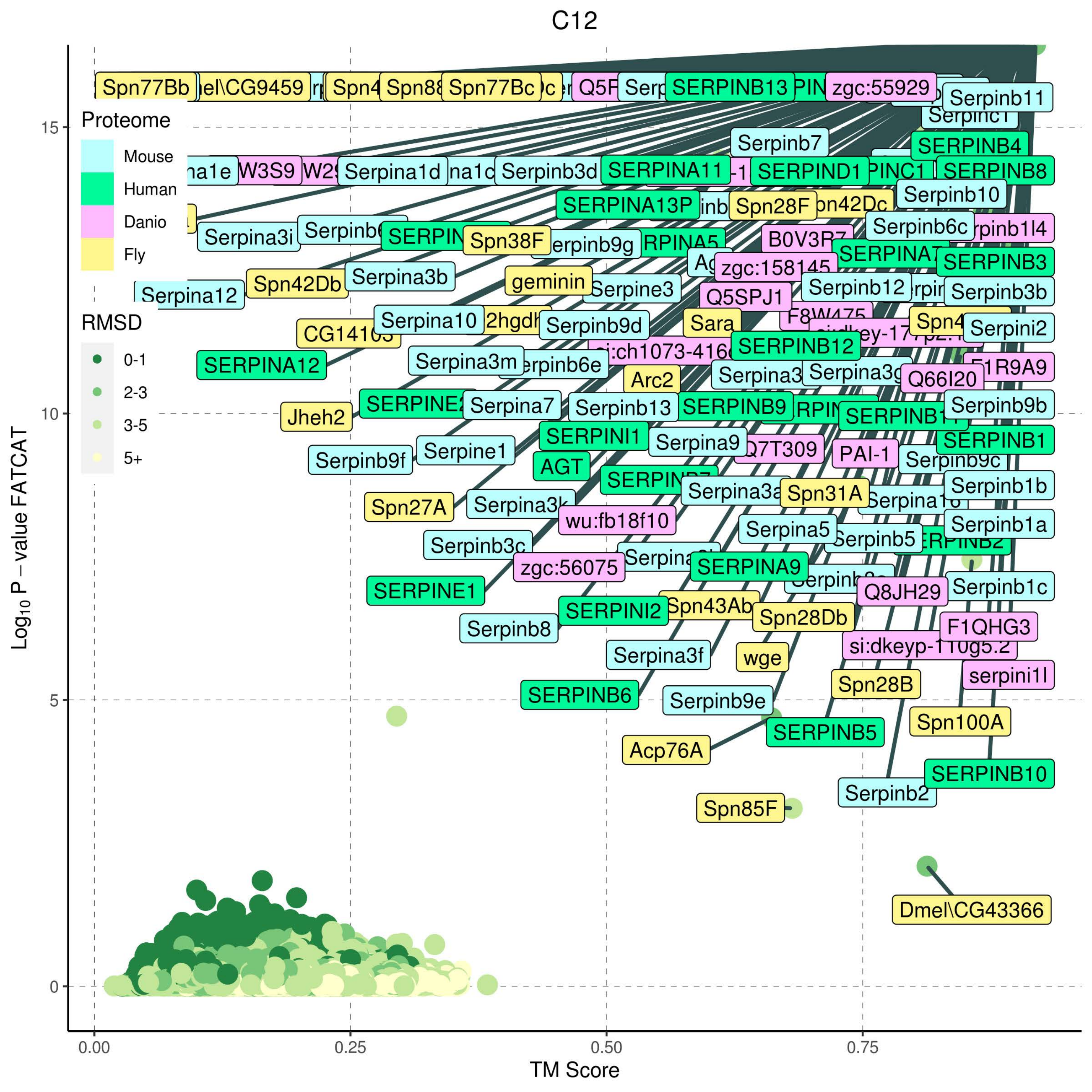

C12
Proteome
Mouse
Human
Danio
Fly
RMSD
0-1
2-3
3-5
5+
Log10 P – value FATCAT
TM Score
0.00
0.25
0.50
0.75
0
5
10
15
Spn77Bb
Spn77Bc
SERPINB13
zgc:55929
Serpinb11
SERPINB4
Serpinb7
Serpina1d
Serpinb3d
SERPINA11
SERPIND1
SERPINB8
Serpinb10
SERPINA13P
Spn28F
Serpinb6c
Serpina3i
Spn38F
SERPINB3
Serpinb12
Serpinb3b
Serpina12
Spn42Db
Serpina3b
geminin
Serpine3
Q5SPJ1
Serpini2
Serpina10
Serpinb9d
Sara
SERPINB12
SERPINA12
Serpina3m
Arc2
Q66I20
Serpina7
Serpinb13
SERPINB9
Serpinb9b
Jheh2
SERPINI1
Serpina9
SERPINB1
Serpinb9f
Serpine1
AGT
PAI-1
Serpinb1b
Spn27A
Spn31A
wu:fb18f10
Serpinb1a
Serpina5
Serpinb5
Serpinb3c
zgc:56075
SERPINA9
SERPINE1
Q8JH29
Serpinb1c
SERPINI2
Spn28Db
Serpinb8
F1QHG3
Serpina3f
wge
serpini1l
Spn28B
SERPINB6
Serpinb9e
Spn100A
SERPINB5
Acp76A
SERPINB10
Serpinb2
Spn85F
Dmel\CG43366

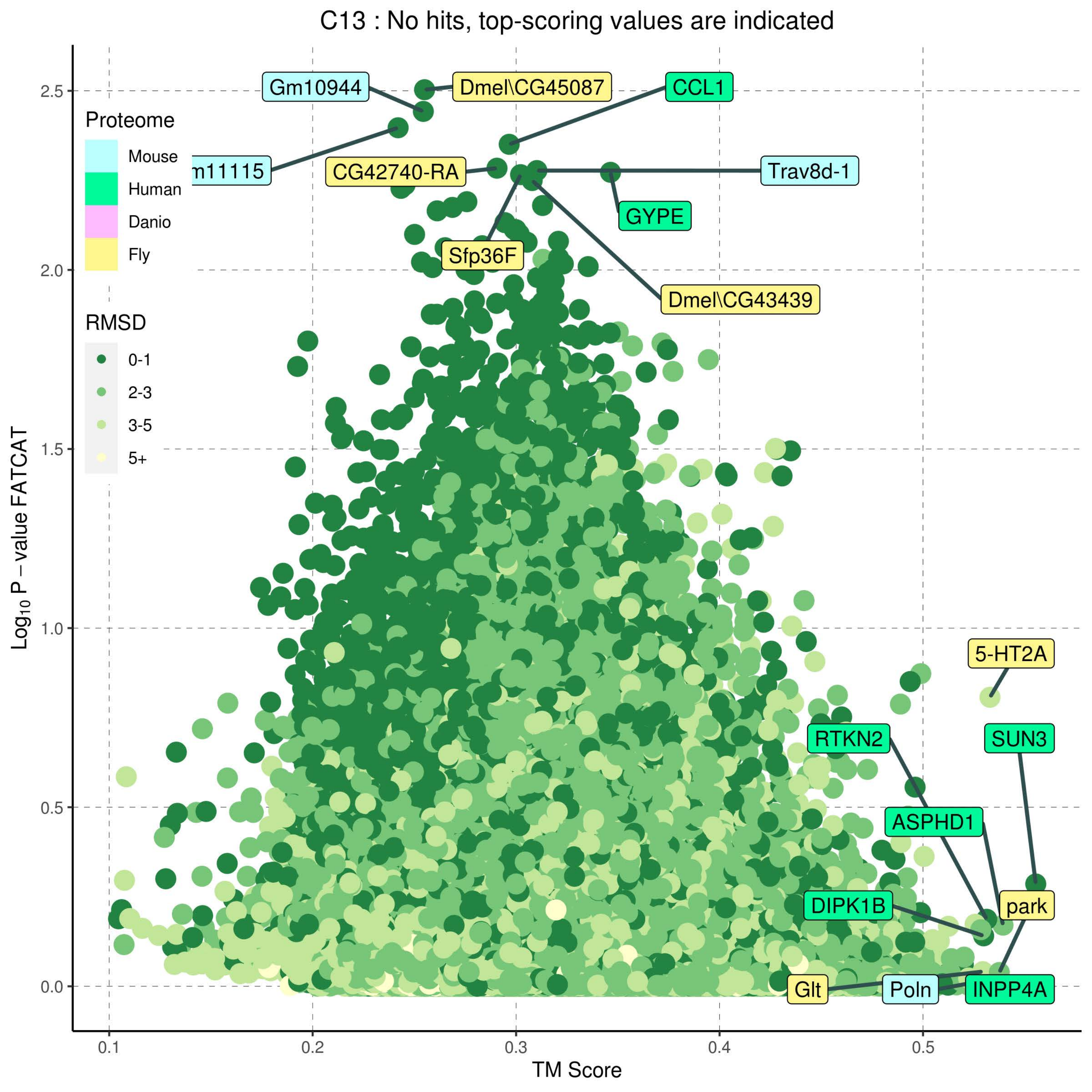

C13 : No hits, top-scoring values are indicated
Proteome
Mouse
Human
Danio
Fly
RMSD
0-1
2-3
3-5
5+
Gm10944
Dmel\CG45087
CCL1
m11115
CG42740-RA
Trav8d-1
GYPE
Sfp36F
Dmel\CG43439
5-HT2A
RTKN2
SUN3
ASPHD1
DIPK1B
park
Glt
Poln
INPP4A
Log10 P − value FATCAT
TM Score
0.0
0.5
1.0
1.5
2.0
2.5
0.1
0.2
0.3
0.4
0.5

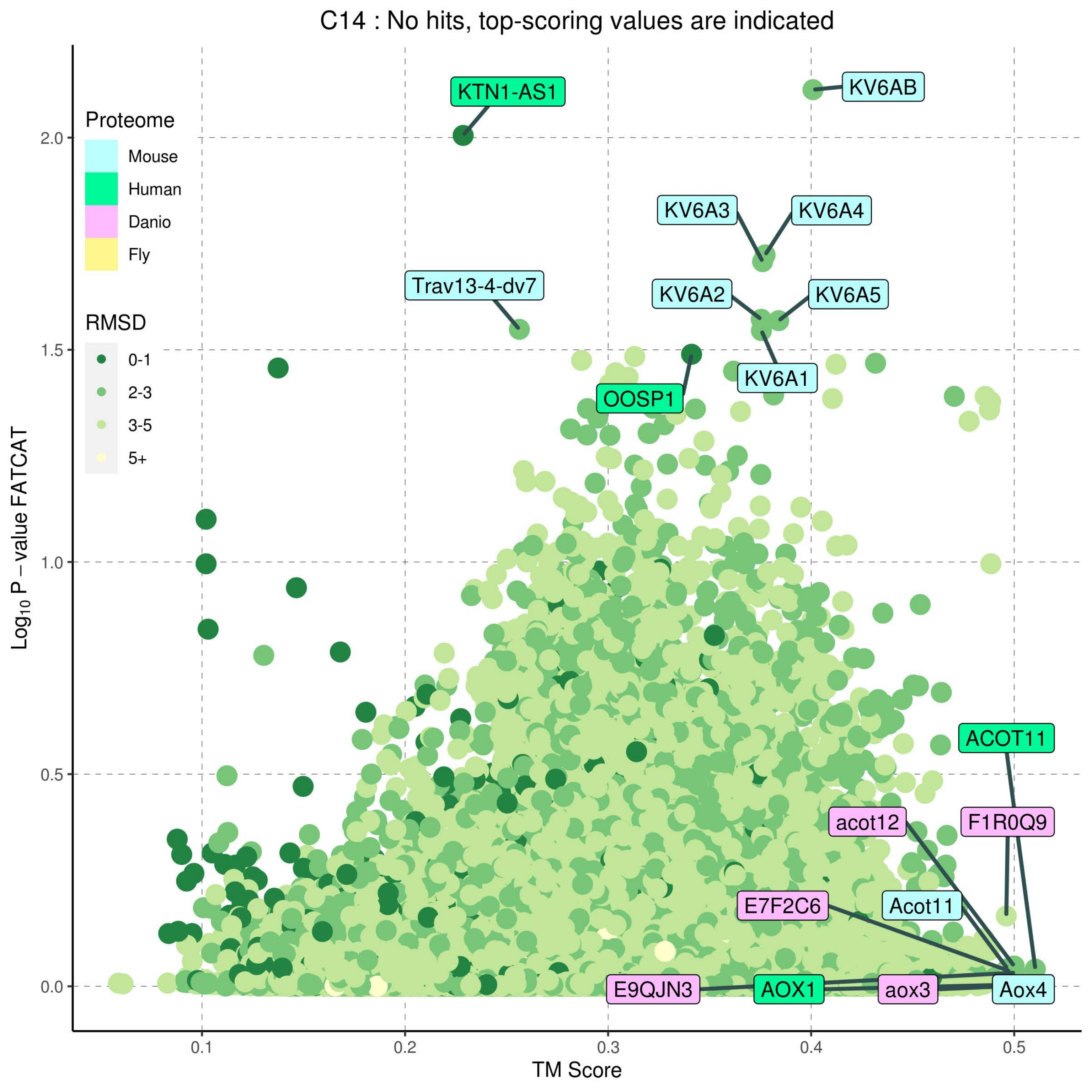

C14 : No hits, top-scoring values are indicated
Proteome
Mouse
Human
Danio
Fly
RMSD
0-1
2-3
3-5
5+
KTN1-AS1
KV6AB
KV6A3
KV6A4
Trav13-4-dv7
KV6A2
KV6A5
KV6A1
OOSP1
ACOT11
acot12
F1R0Q9
E7F2C6
Acot11
E9QJN3
AOX1
aox3
Aox4
Log10 P − value FATCAT
TM Score
0.0
0.5
1.0
1.5
2.0
0.1
0.2
0.3
0.4
0.5

C23 : No hits, top-scoring values are indicated

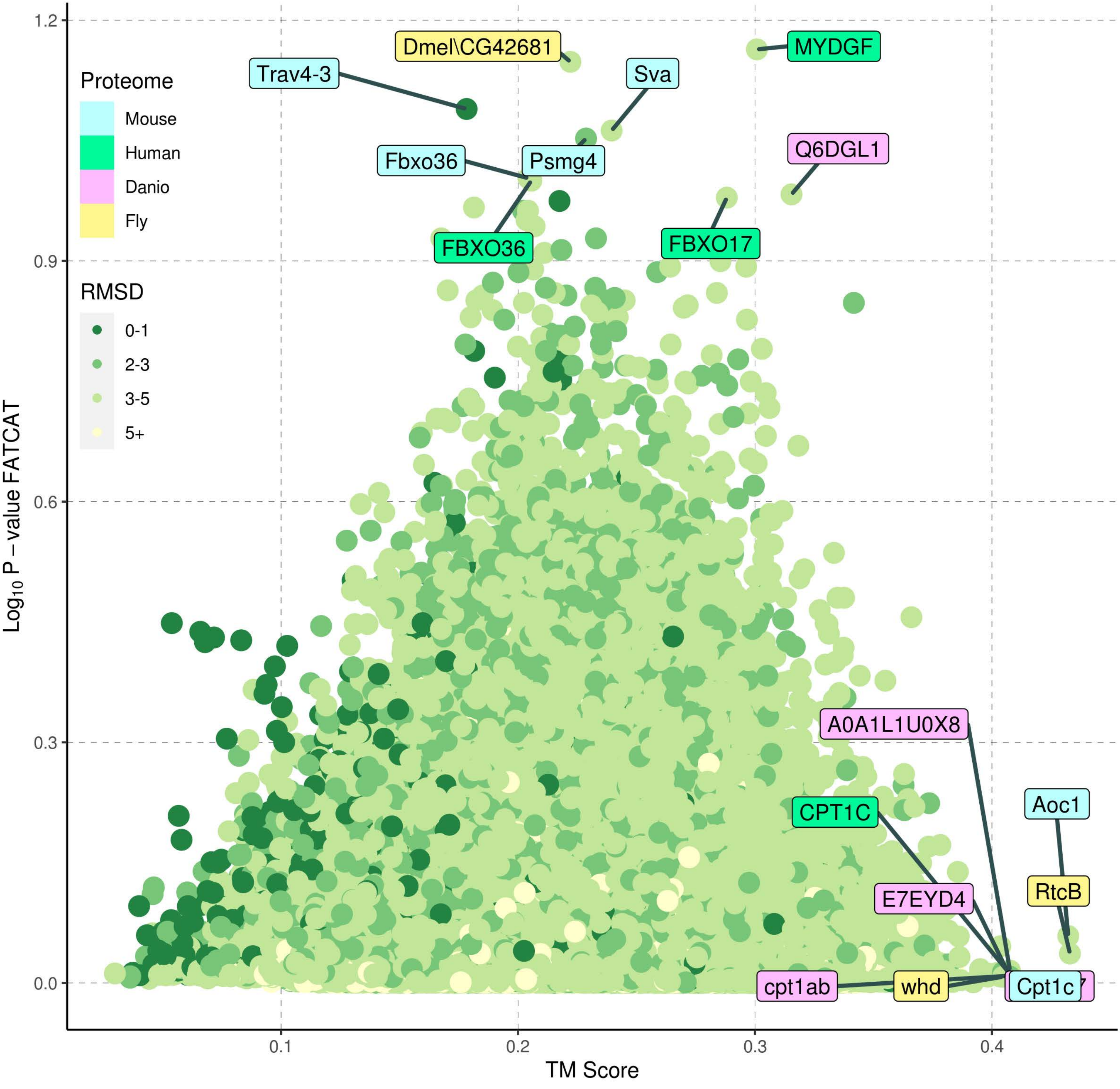

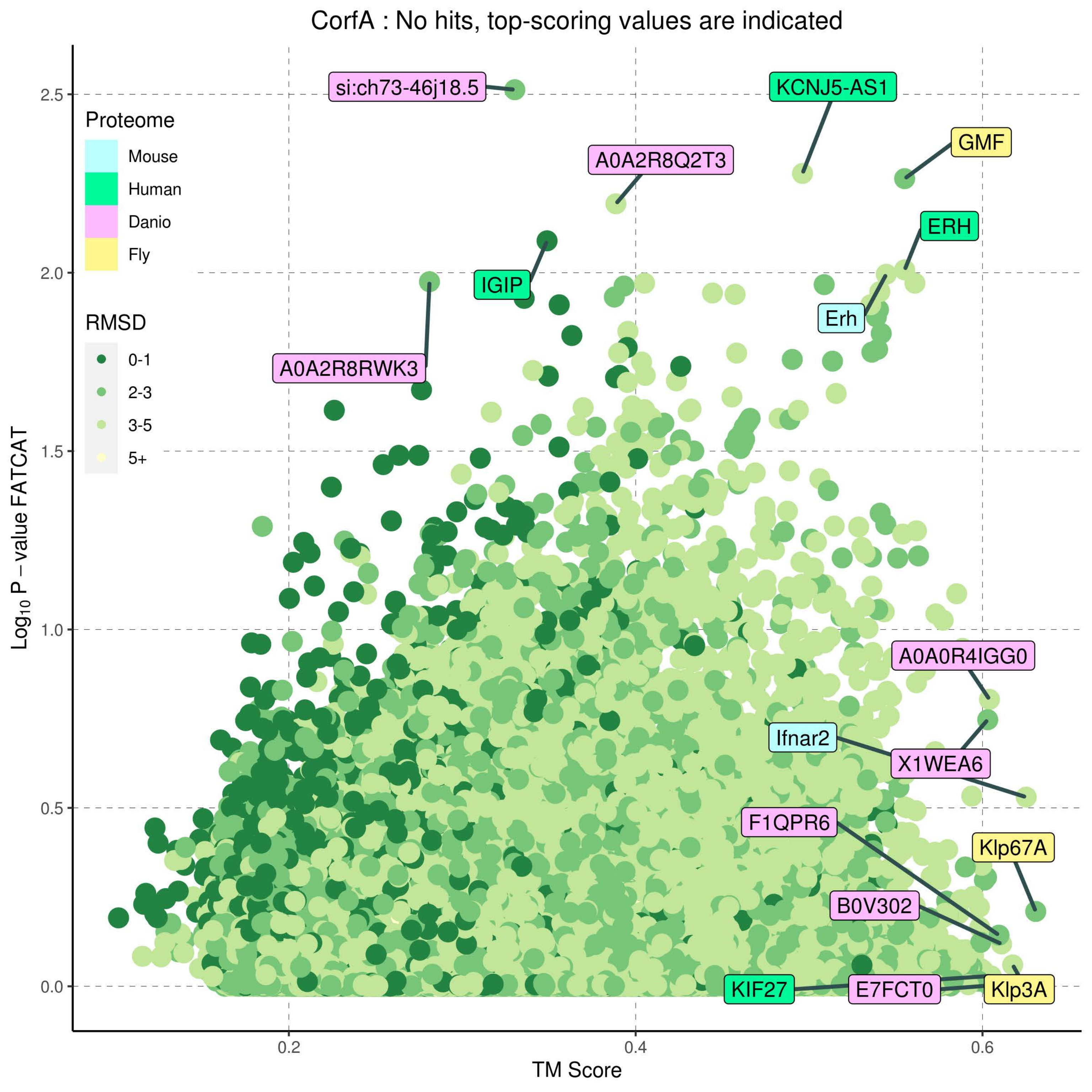
CorfA : No hits, top-scoring values are indicated
Proteome
Mouse
Human
Danio
Fly
RMSD
0-1
2-3
3-5
5+
si:ch73-46j18.5
KCNJ5-AS1
GMF
A0A2R8Q2T3
ERH
IGIP
Erh
A0A2R8RWK3
A0A0R4IGG0
Ifnar2
X1WEA6
F1QPR6
Klp67A
B0V302
KIF27
E7FCT0
Klp3A
2.5
2.0
1.5
1.0
0.5
0.0
0.2
0.4
0.6
TM Score
Log10 P – value FATCAT

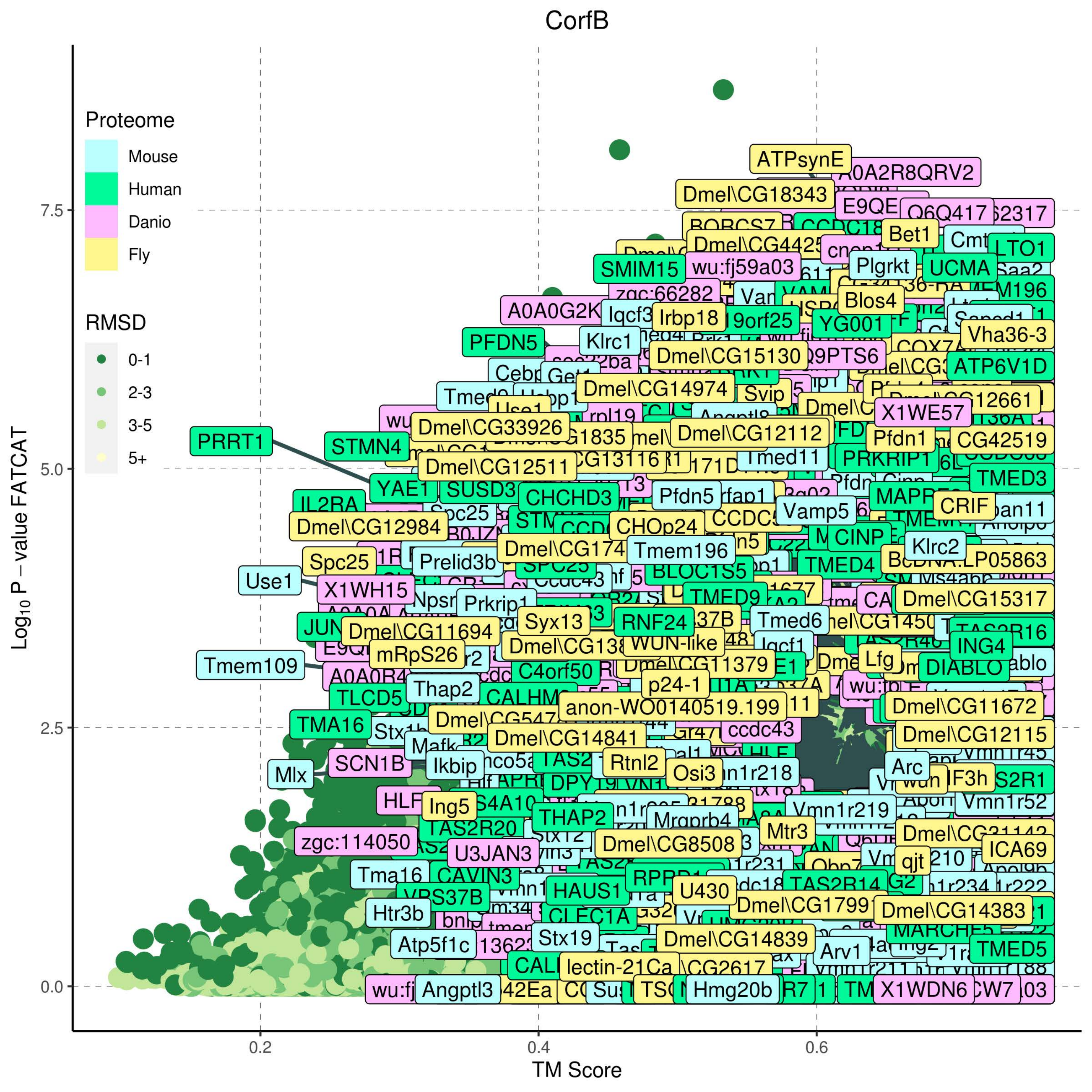
CorfB
Proteome
Mouse
Human
Danio
Fly
RMSD
0-1
2-3
3-5
5+
Log10 P − value FATCAT
TM Score
0.0
2.5
5.0
7.5
0.2
0.4
0.6
ATPsynE
A0A2R8QRV2
Dmel\CG18343
E9QE
Q6Q417
Bet1
SMIM15
wu:fj59a03
Plgrkt
UCMA
LTO1
zgc:66282
Blos4
A0A0G2K
Iqcf3
Irbp18
YG001
PFDN5
Klrc1
Dmel\CG15130
Q9PTS6
Vha36-3
ATP6V1D
Dmel\CG14974
Svip
X1WE57
PRRT1
STMN4
Dmel\CG33926
Dmel\CG12112
Pfdn1
CG42519
Dmel\CG12511
Tmed11
PRKRIP1
TMED3
YAE1
SUSD3
CHCHD3
Pfdn5
MAPRE
CRIF
Dmel\CG12984
Spc25
Vamp5
CHOp24
CINP
Dmel\CG174
Tmem196
Klrc2
Prelid3b
TMED4
BcDNA:LP05863
Use1
X1WH15
BLOC1S5
TMED9
Dmel\CG15317
Prkrip1
Syx13
RNF24
Tmed6
JUN
Dmel\CG11694
ING4
mRpS26
Tmem109
C4orf50
Lfg
DIABLO
Thap2
p24-1
TLCD5
anon-WO0140519.199
Dmel\CG11672
TMA16
Dmel\CG14841
ccdc43
Dmel\CG12115
Mafk
SCN1B
Ikbip
Rtnl2
Osi3
Arc
Mlx
Vmn1r52
HLF
Ing5
THAP2
Vmn1r219
Mrgprb4
Mtr3
zgc:114050
U3JAN3
Dmel\CG8508
ICA69
Tma16
CAVIN3
HAUS1
TAS2R14
qjt
U430
Htr3b
CLEC1A
Dmel\CG14383
Stx19
Dmel\CG14839
Atp5f1c
Arv1
TMED5
lectin-21Ca
Angptl3
Hmg20b
X1WDN6

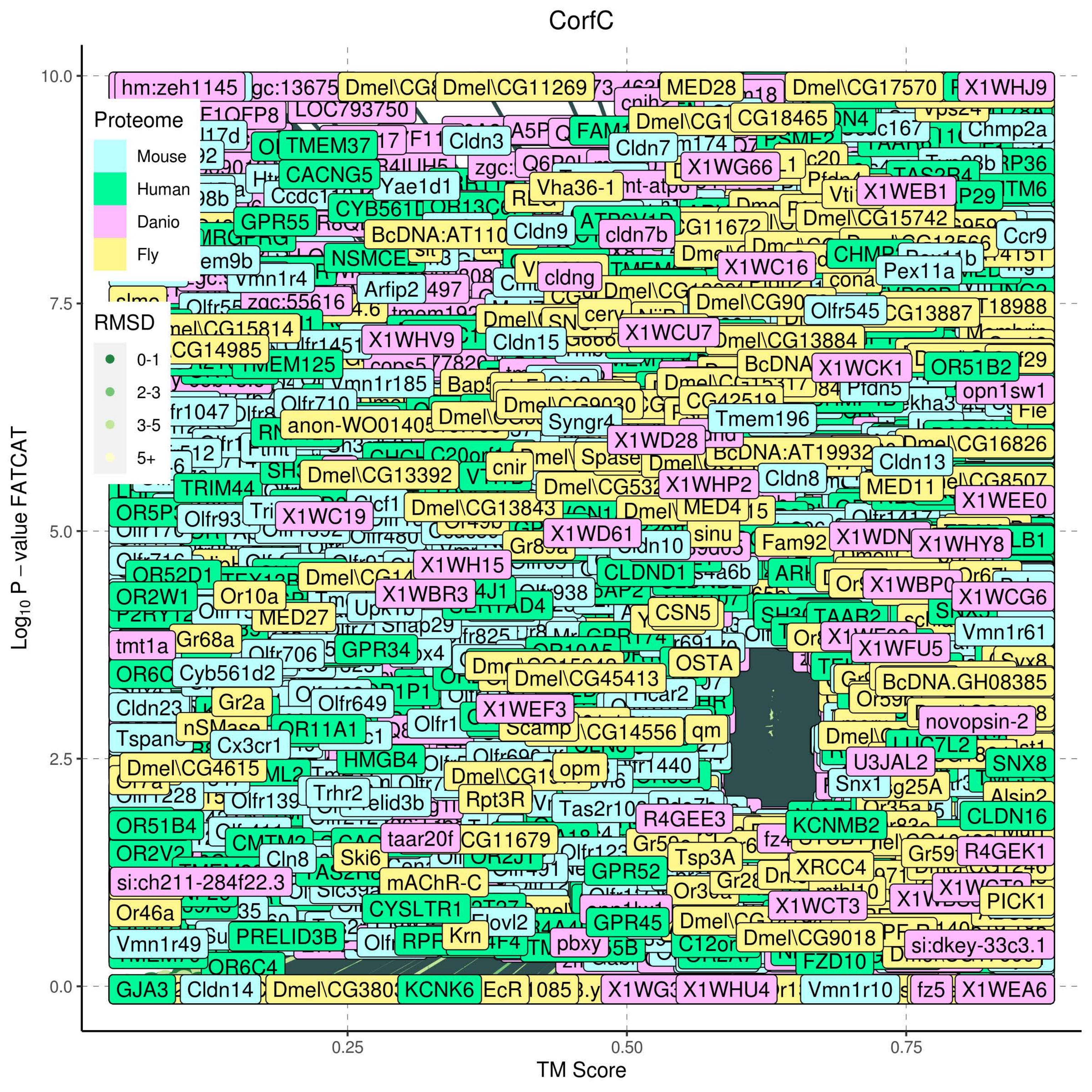
CorfC
Proteome
Mouse
Human
Danio
Fly
RMSD
0-1
2-3
3-5
5+
Log_{10} P – value FATCAT
10.0
7.5
5.0
2.5
0.0
0.25
0.50
0.75
TM Score

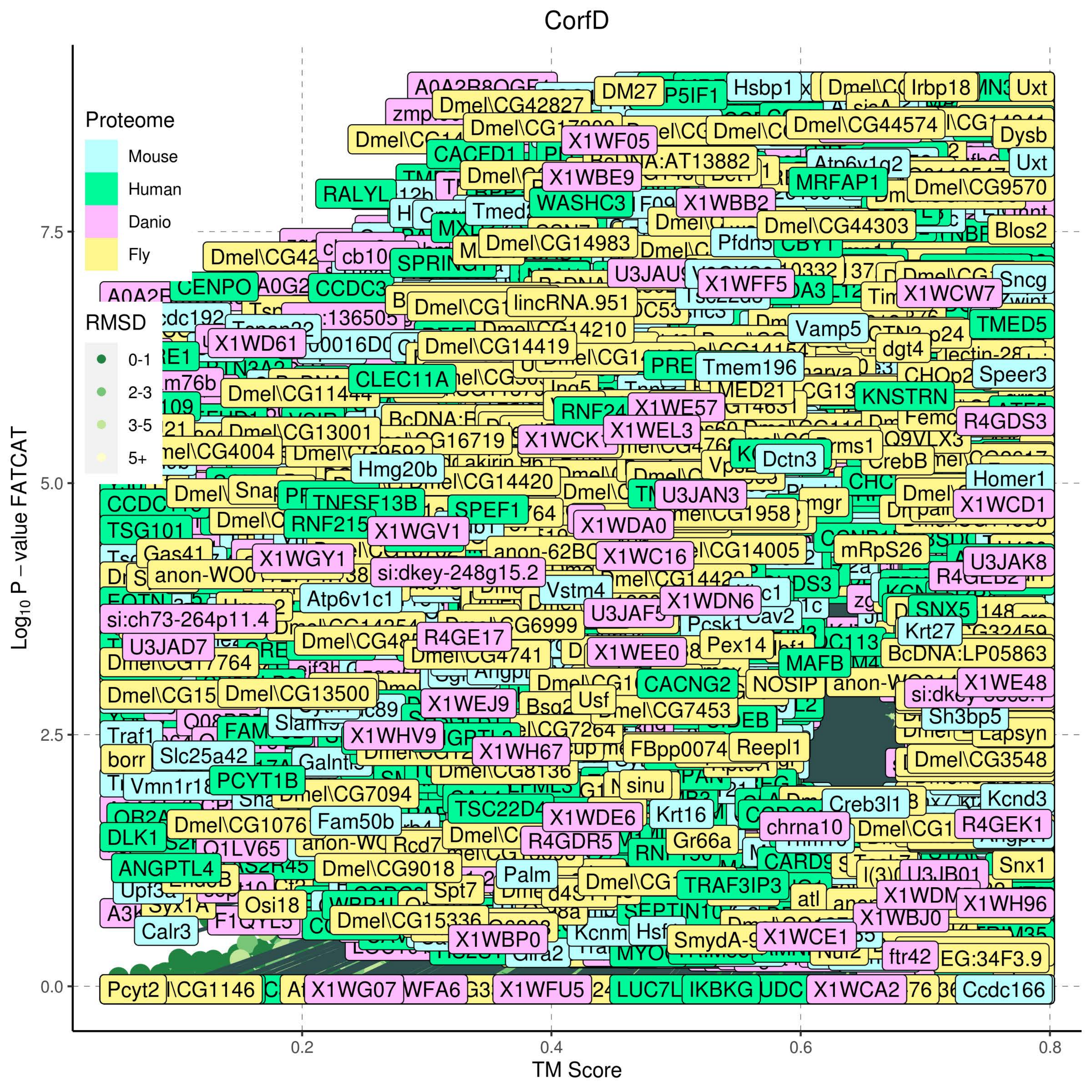
CorfD
Proteome
Mouse
Human
Danio
Fly
RMSD
0-1
2-3
3-5
5+
Log_{10} P – value FATCAT
7.5
5.0
2.5
0.0
TM Score
0.2
0.4
0.6
0.8

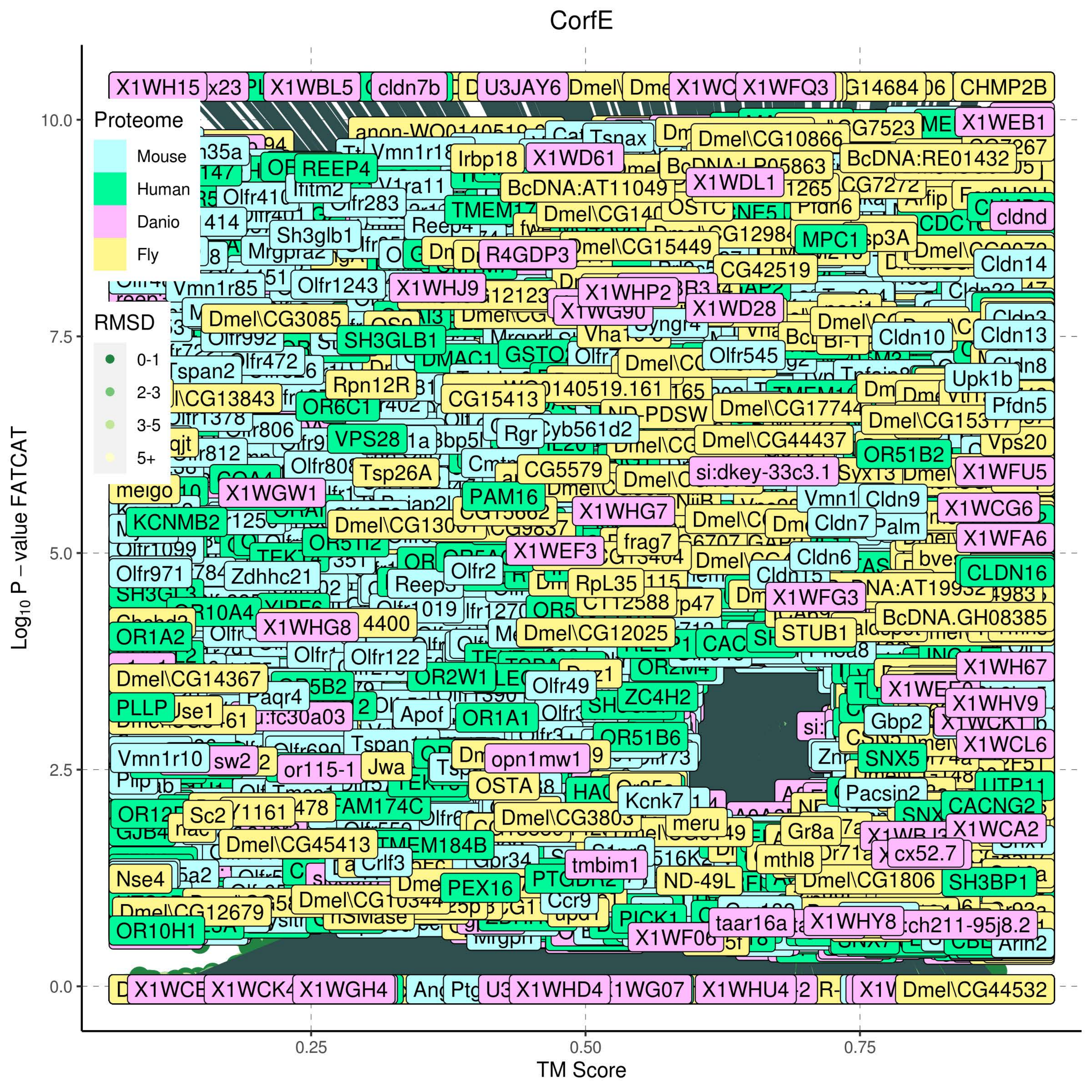

CorfE
Proteome
Mouse
Human
Danio
Fly
RMSD
0-1
2-3
3-5
5+
10.0
7.5
5.0
2.5
0.0
Log10 P – value FATCAT
0.25
0.50
0.75
TM Score

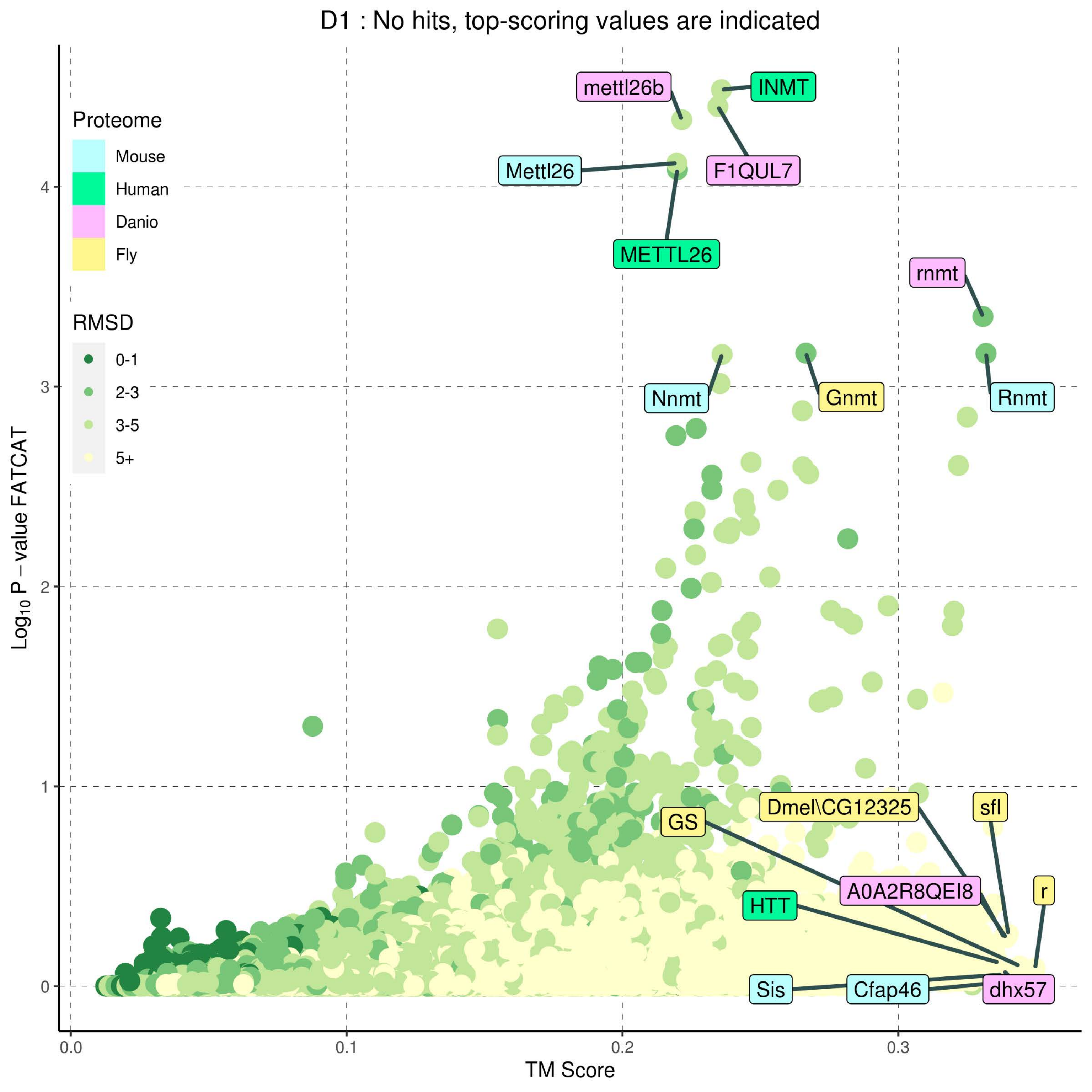
D1 : No hits, top-scoring values are indicated
Proteome
Mouse
Human
Danio
Fly
RMSD
0-1
2-3
3-5
5+
mettl26b
INMT
Mettl26
F1QUL7
METTL26
rnmt
Nnmt
Gnmt
Rnmt
GS
Dmel\CG12325
sfl
A0A2R8QEI8
r
HTT
Sis
Cfap46
dhx57
Log10 P − value FATCAT
TM Score
0.0
0.1
0.2
0.3
0
1
2
3
4

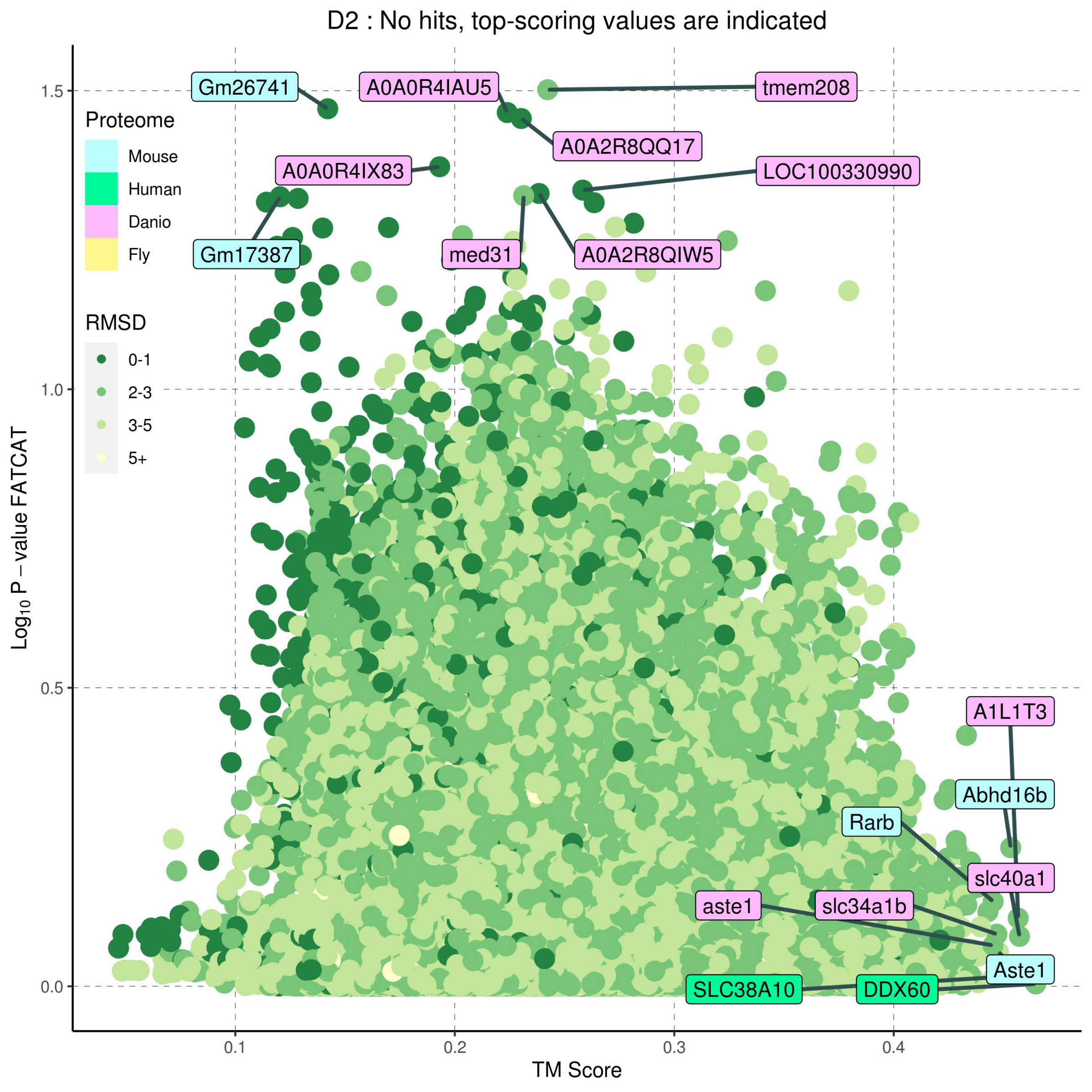

D2 : No hits, top-scoring values are indicated
Proteome
Mouse
Human
Danio
Fly
RMSD
0-1
2-3
3-5
5+
Gm26741
A0A0R4IAU5
tmem208
A0A2R8QQ17
A0A0R4IX83
LOC100330990
Gm17387
med31
A0A2R8QIW5
A1L1T3
Abhd16b
Rarb
slc40a1
aste1
slc34a1b
Aste1
SLC38A10
DDX60
1.5
1.0
0.5
0.0
0.1
0.2
0.3
0.4
TM Score
Log10 P – value FATCAT

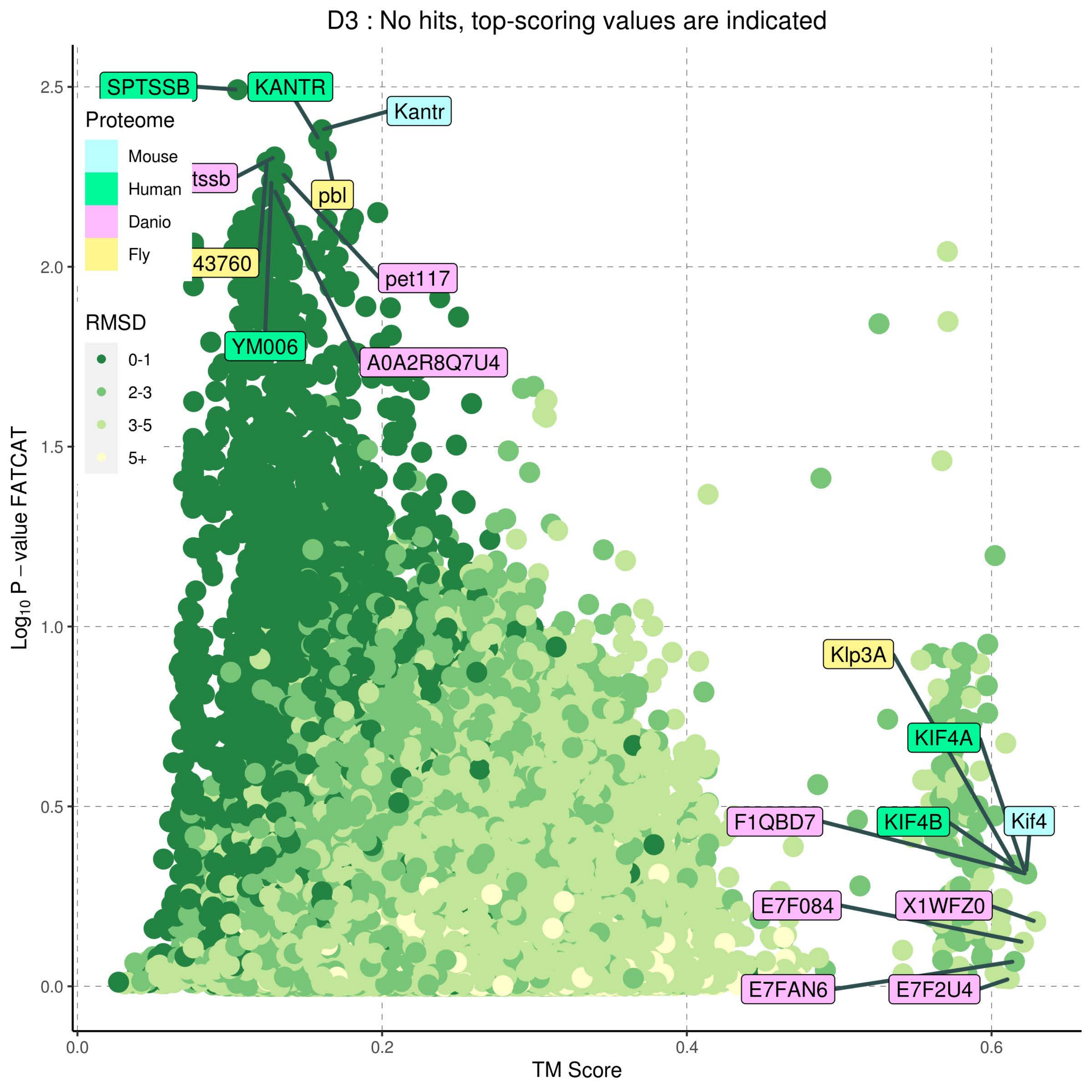
D3 : No hits, top-scoring values are indicated
Proteome
Mouse
Human
Danio
Fly
RMSD
0-1
2-3
3-5
5+
SPTSSB
KANTR
Kantr
tssb
pbl
43760
pet117
YM006
A0A2R8Q7U4
Klp3A
KIF4A
F1QBD7
KIF4B
Kif4
E7F084
X1WFZ0
E7FAN6
E7F2U4
Log_{10} P – value FATCAT
TM Score
0.0
0.2
0.4
0.6
0.0
0.5
1.0
1.5
2.0
2.5

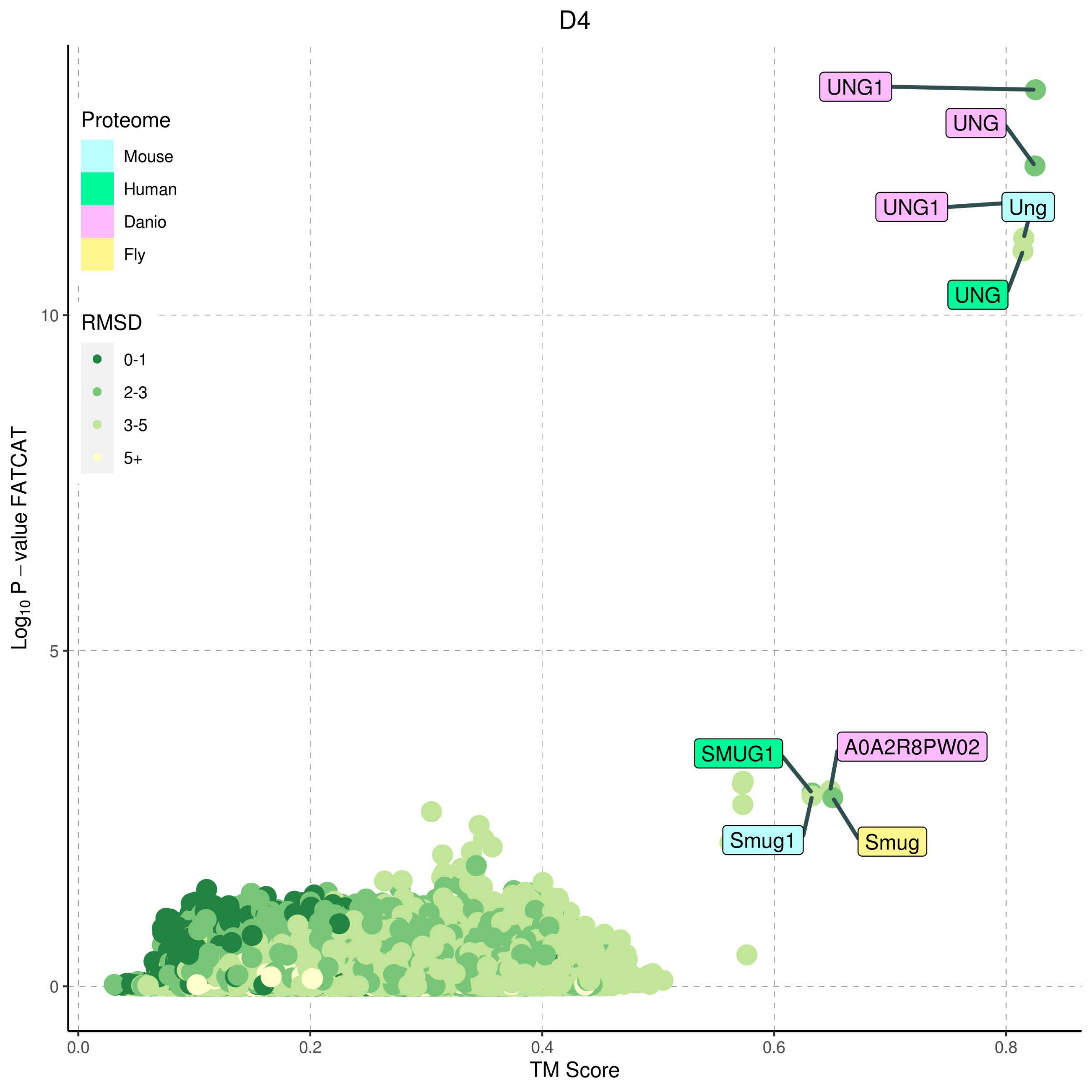

D4
Proteome
Mouse
Human
Danio
Fly
RMSD
0-1
2-3
3-5
5+
UNG1
UNG
UNG1
Ung
UNG
SMUG1
A0A2R8PW02
Smug1
Smug
Log10 P – value FATCAT
TM Score
0
5
10
0.0
0.2
0.4
0.6
0.8

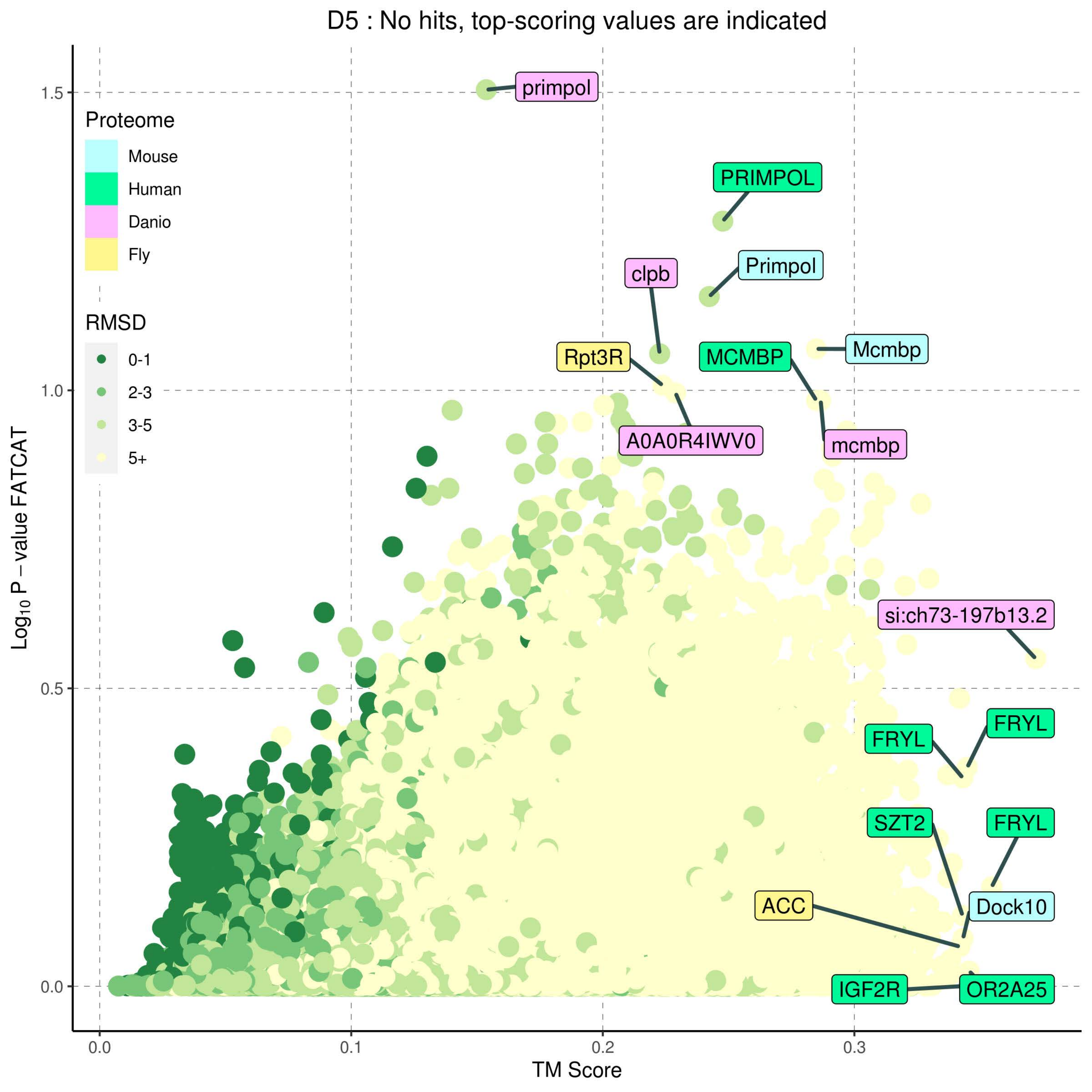

D5 : No hits, top-scoring values are indicated
Proteome
Mouse
Human
Danio
Fly
RMSD
0-1
2-3
3-5
5+
primpol
PRIMPOL
clpb
Primpol
Rpt3R
MCMBP
Mcmbp
A0A0R4IWV0
mcmbp
si:ch73-197b13.2
FRYL
FRYL
SZT2
FRYL
ACC
Dock10
IGF2R
OR2A25
1.5
1.0
0.5
0.0
Log10 P − value FATCAT
0.0
0.1
0.2
0.3
TM Score

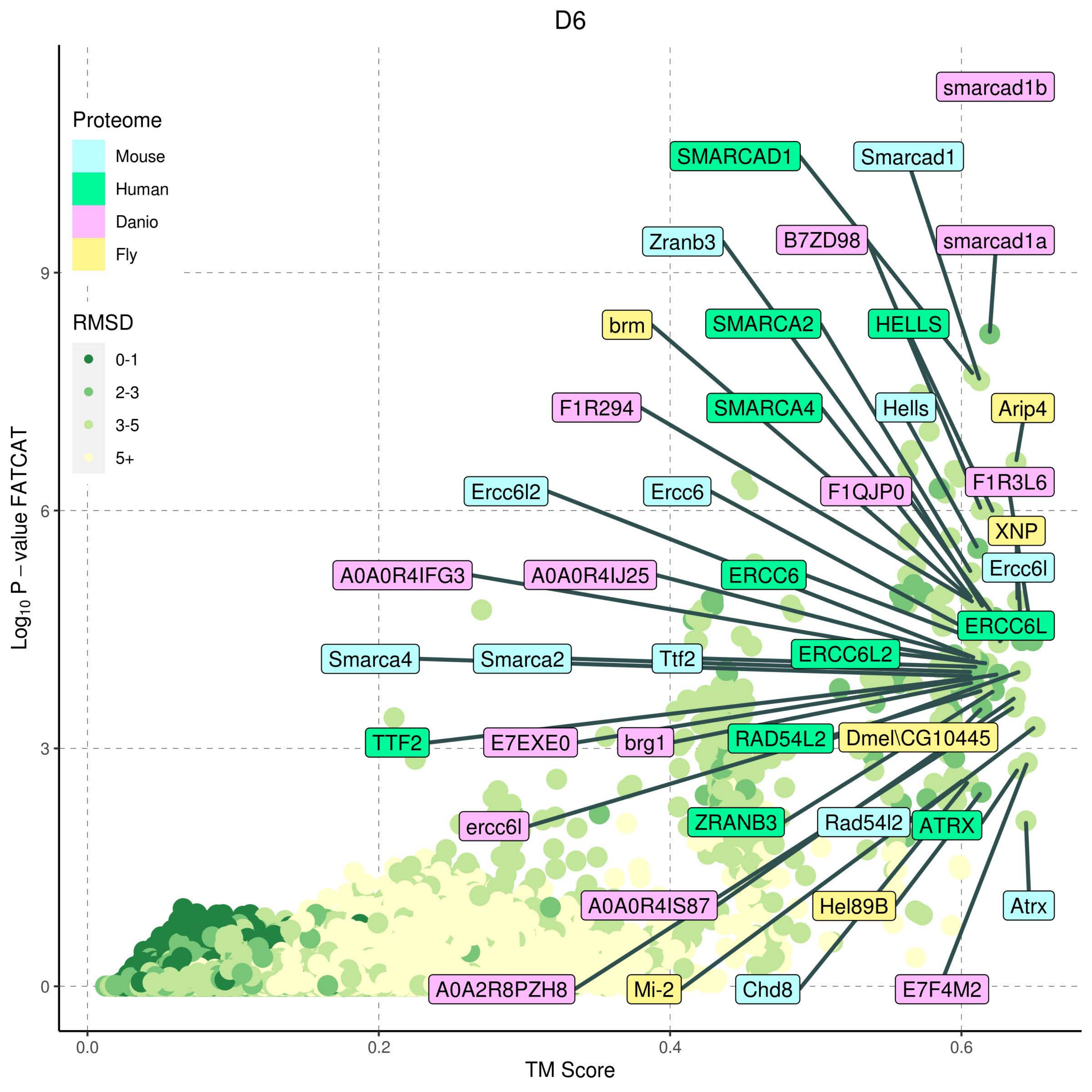
D6
Proteome
Mouse
Human
Danio
Fly
RMSD
0-1
2-3
3-5
5+
smarcad1b
SMARCAD1
Smarcad1
Zranb3
B7ZD98
smarcad1a
brm
SMARCA2
HELLS
F1R294
SMARCA4
Hells
Arip4
Ercc6l2
Ercc6
F1QJP0
F1R3L6
XNP
A0A0R4IFG3
A0A0R4IJ25
ERCC6
Ercc6l
ERCC6L
Smarca4
Smarca2
Ttf2
ERCC6L2
TTF2
E7EXE0
brg1
RAD54L2
Dmel\CG10445
ercc6l
ZRANB3
Rad54l2
ATRX
A0A0R4IS87
Hel89B
Atrx
A0A2R8PZH8
Mi-2
Chd8
E7F4M2
Log10 P − value FATCAT
TM Score
0
3
6
9
0.0
0.2
0.4
0.6

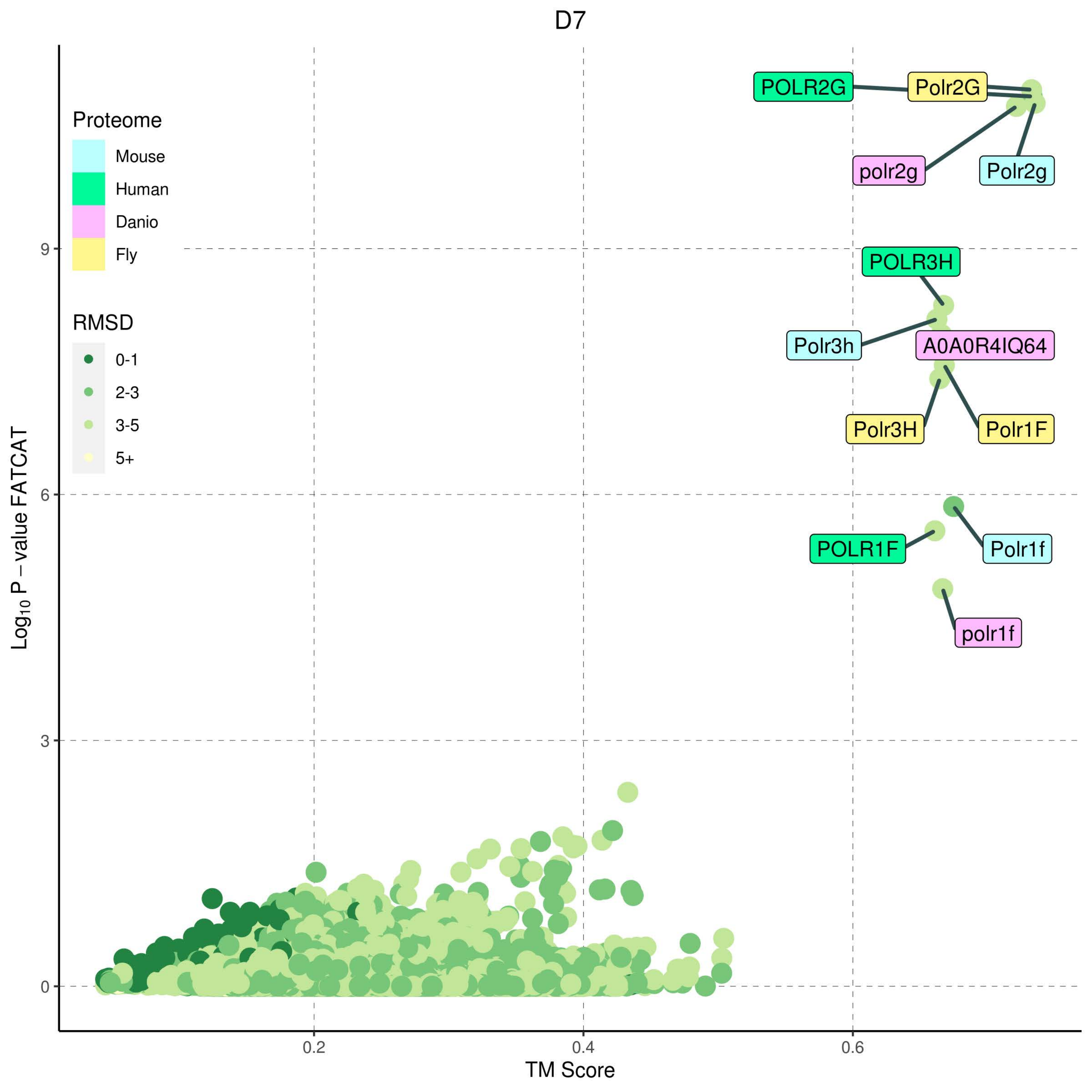

D7
Proteome
Mouse
Human
Danio
Fly
RMSD
0-1
2-3
3-5
5+
POLR2G
Polr2G
polr2g
Polr2g
POLR3H
Polr3h
A0A0R4IQ64
Polr3H
Polr1F
POLR1F
Polr1f
polr1f
9
6
3
0
0.2
0.4
0.6
TM Score
Log10 P − value FATCAT

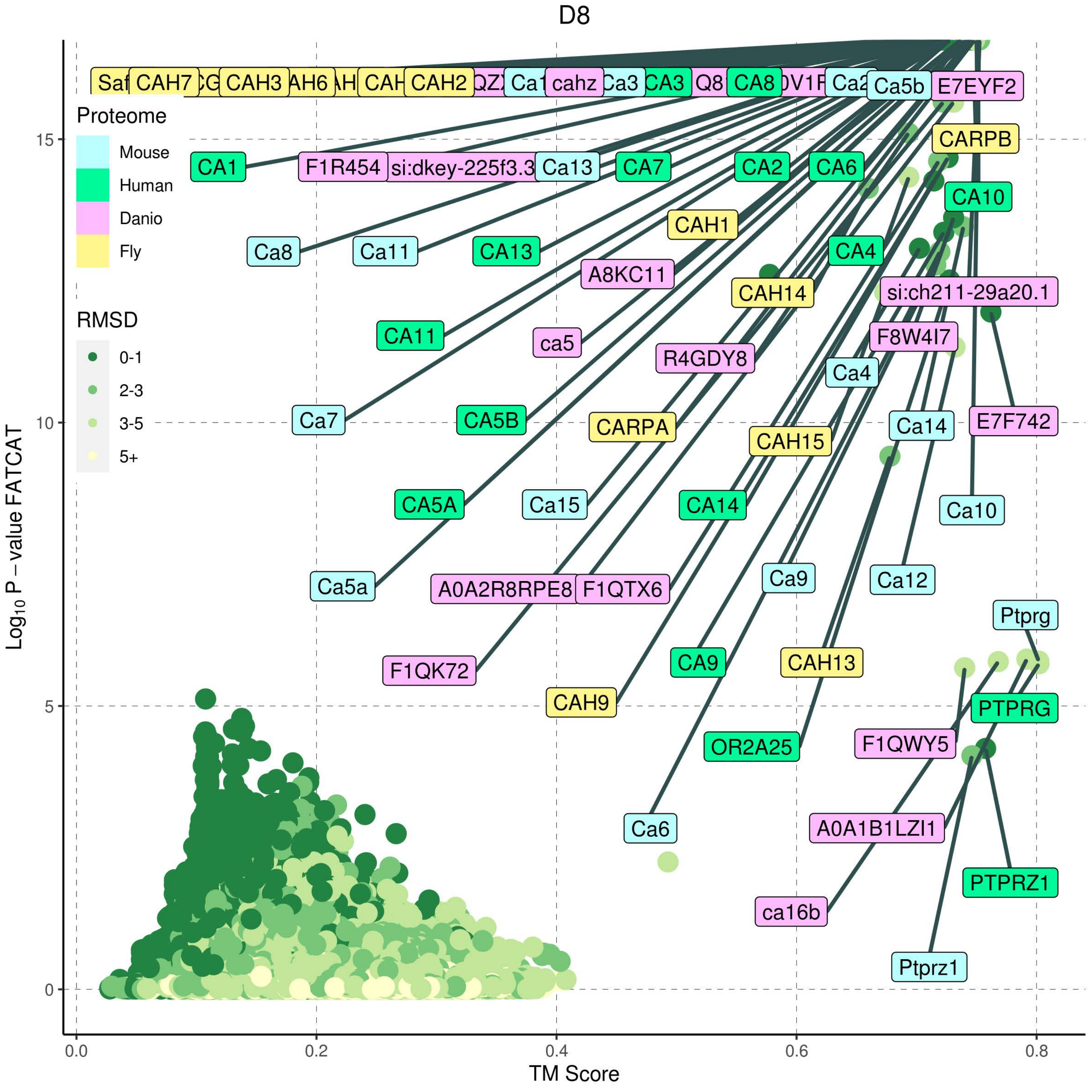

D8
Proteome
Mouse
Human
Danio
Fly
RMSD
0-1
2-3
3-5
5+
Log10 P − value FATCAT
TM Score
0.0
0.2
0.4
0.6
0.8
0
5
10
15
CAH7
CAH3
CAH2
cahz
Ca3
CA3
CA8
Ca5b
E7EYF2
CARPB
CA1
F1R454
si:dkey-225f3.3
Ca13
CA7
CA2
CA6
CA10
CAH1
Ca8
Ca11
CA13
CA4
A8KC11
CAH14
si:ch211-29a20.1
CA11
ca5
R4GDY8
F8W4I7
Ca4
Ca7
CA5B
CARPA
CAH15
Ca14
E7F742
CA5A
Ca15
CA14
Ca10
Ca5a
A0A2R8RPE8
F1QTX6
Ca9
Ca12
Ptprg
F1QK72
CA9
CAH13
CAH9
PTPRG
OR2A25
F1QWY5
Ca6
A0A1B1LZI1
PTPRZ1
ca16b
Ptprz1

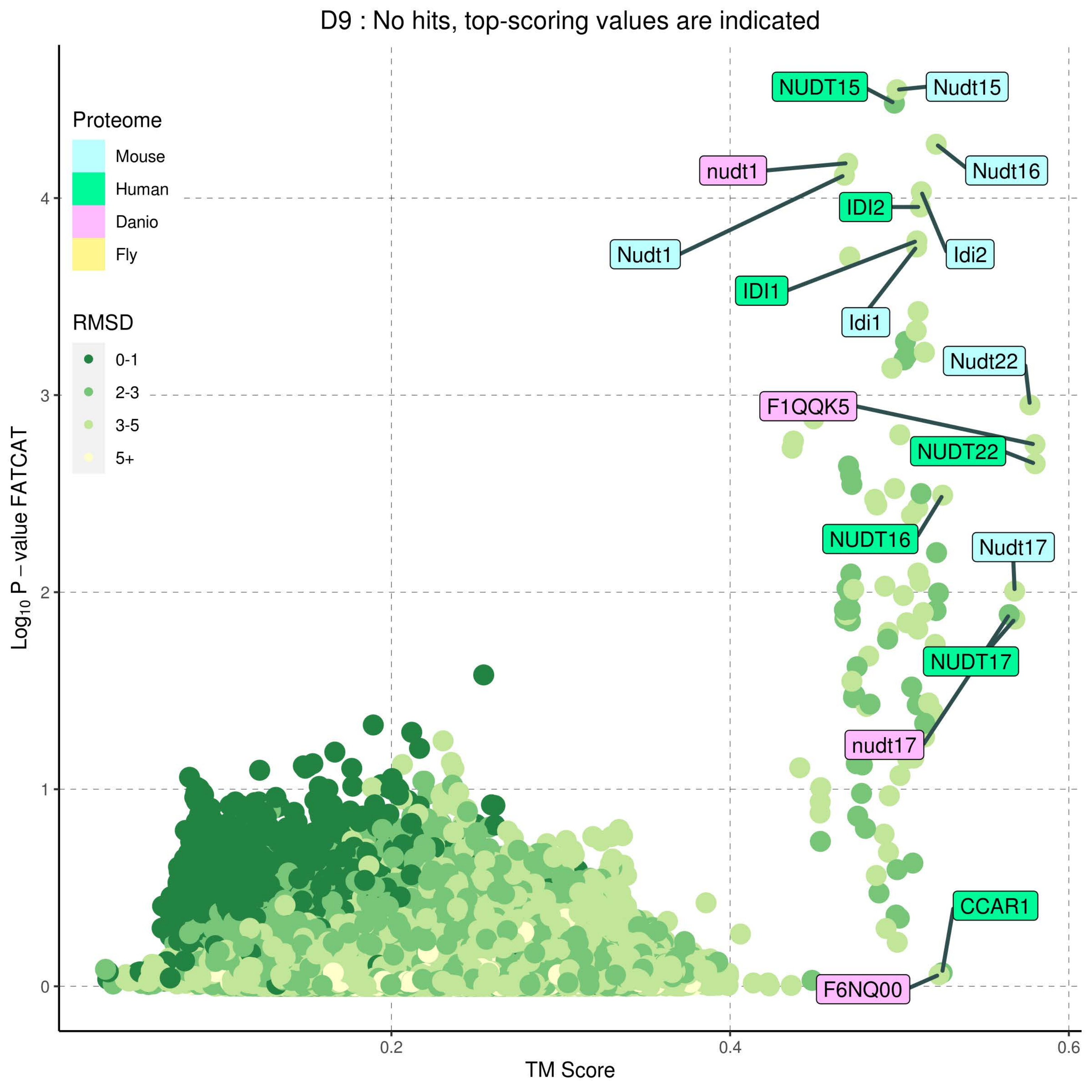

D9 : No hits, top-scoring values are indicated
Proteome
Mouse
Human
Danio
Fly
RMSD
0-1
2-3
3-5
5+
NUDT15
Nudt15
nudt1
Nudt16
IDI2
Nudt1
Idi2
IDI1
Idi1
Nudt22
F1QQK5
NUDT22
NUDT16
Nudt17
NUDT17
nudt17
CCAR1
F6NQ00
Log10 P – value FATCAT
TM Score
0
1
2
3
4
0.2
0.4
0.6

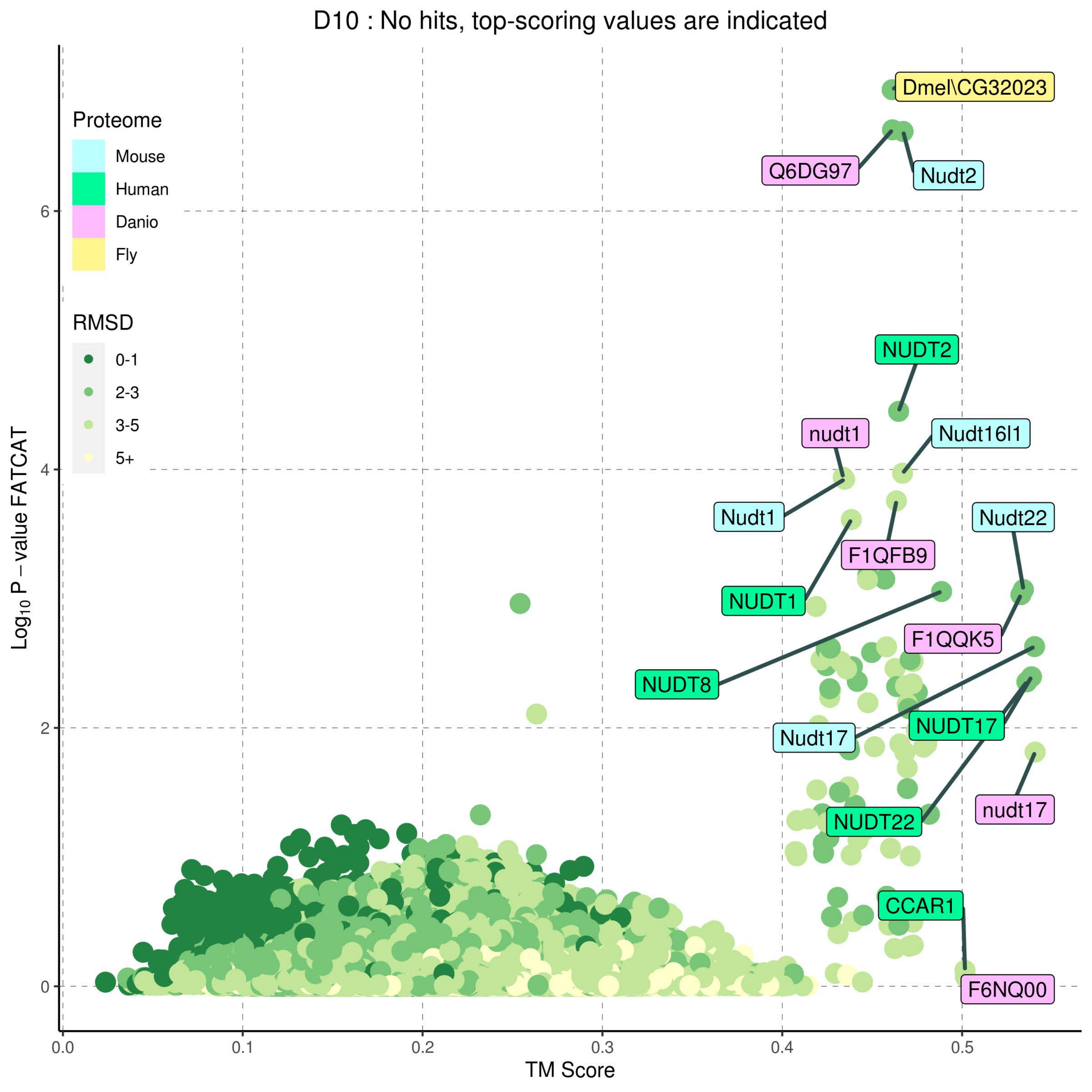

D10 : No hits, top-scoring values are indicated
Proteome
Mouse
Human
Danio
Fly
RMSD
0-1
2-3
3-5
5+
Dmel\CG32023
Q6DG97
Nudt2
NUDT2
nudt1
Nudt16l1
Nudt1
Nudt22
F1QFB9
NUDT1
NUDT8
F1QQK5
Nudt17
NUDT17
nudt17
NUDT22
CCAR1
F6NQ00
Log10 P – value FATCAT
TM Score
0
2
4
6
0.0
0.1
0.2
0.3
0.4
0.5

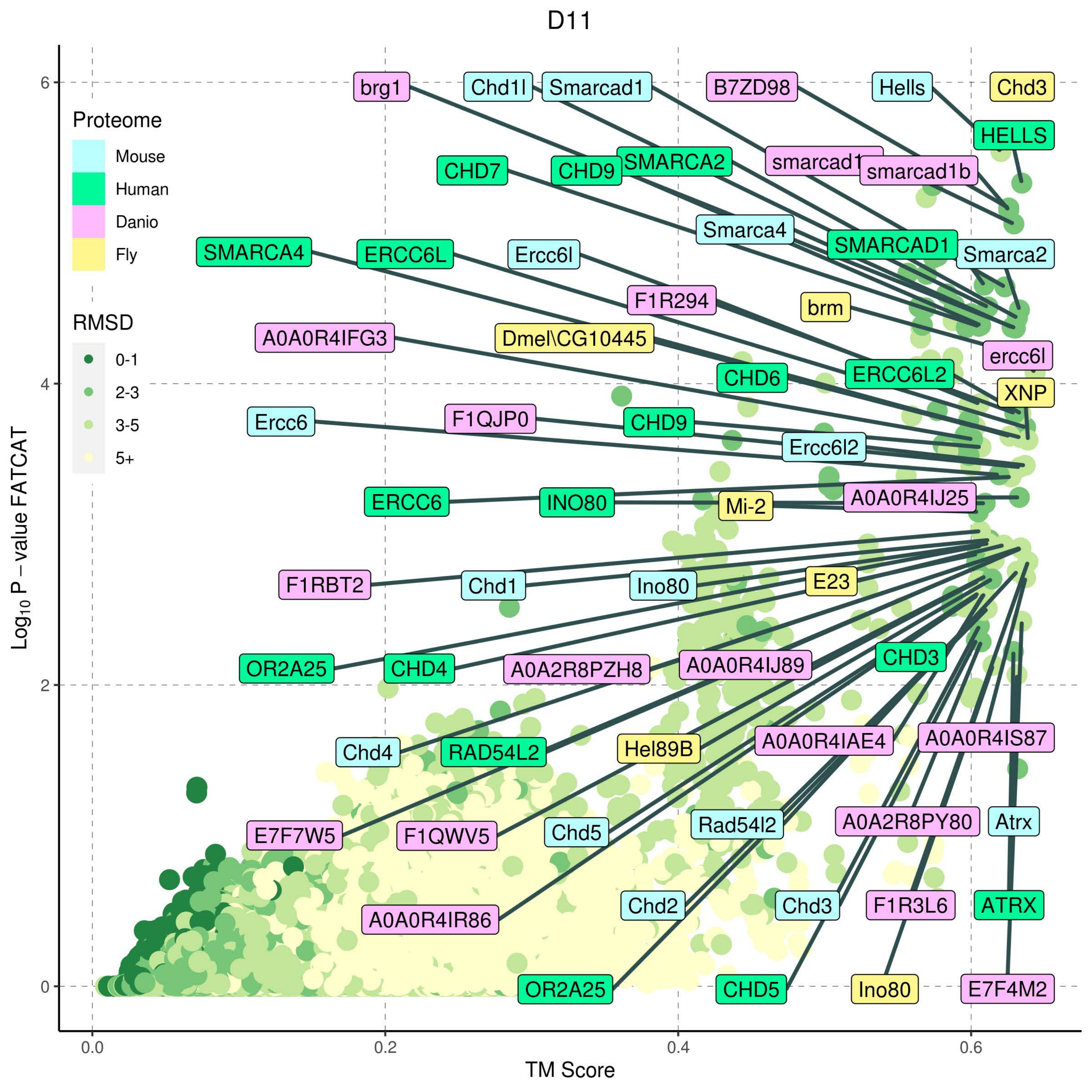

D11
Proteome
Mouse
Human
Danio
Fly
RMSD
0-1
2-3
3-5
5+
brg1
Chd1l
Smarcad1
B7ZD98
Hells
Chd3
HELLS
CHD7
CHD9
SMARCA2
smarcad1
smarcad1b
Smarca4
SMARCAD1
Smarca2
SMARCA4
ERCC6L
Ercc6l
F1R294
brm
A0A0R4IFG3
Dmel\CG10445
ercc6l
CHD6
ERCC6L2
XNP
Ercc6
F1QJP0
CHD9
Ercc6l2
ERCC6
INO80
Mi-2
A0A0R4IJ25
F1RBT2
Chd1
Ino80
E23
OR2A25
CHD4
A0A2R8PZH8
A0A0R4IJ89
CHD3
Chd4
RAD54L2
Hel89B
A0A0R4IAE4
A0A0R4IS87
E7F7W5
F1QWV5
Chd5
Rad54l2
A0A2R8PY80
Atrx
Chd2
Chd3
F1R3L6
ATRX
A0A0R4IR86
OR2A25
CHD5
Ino80
E7F4M2
Log10 P − value FATCAT
TM Score
0.0
0.2
0.4
0.6
0
2
4
6

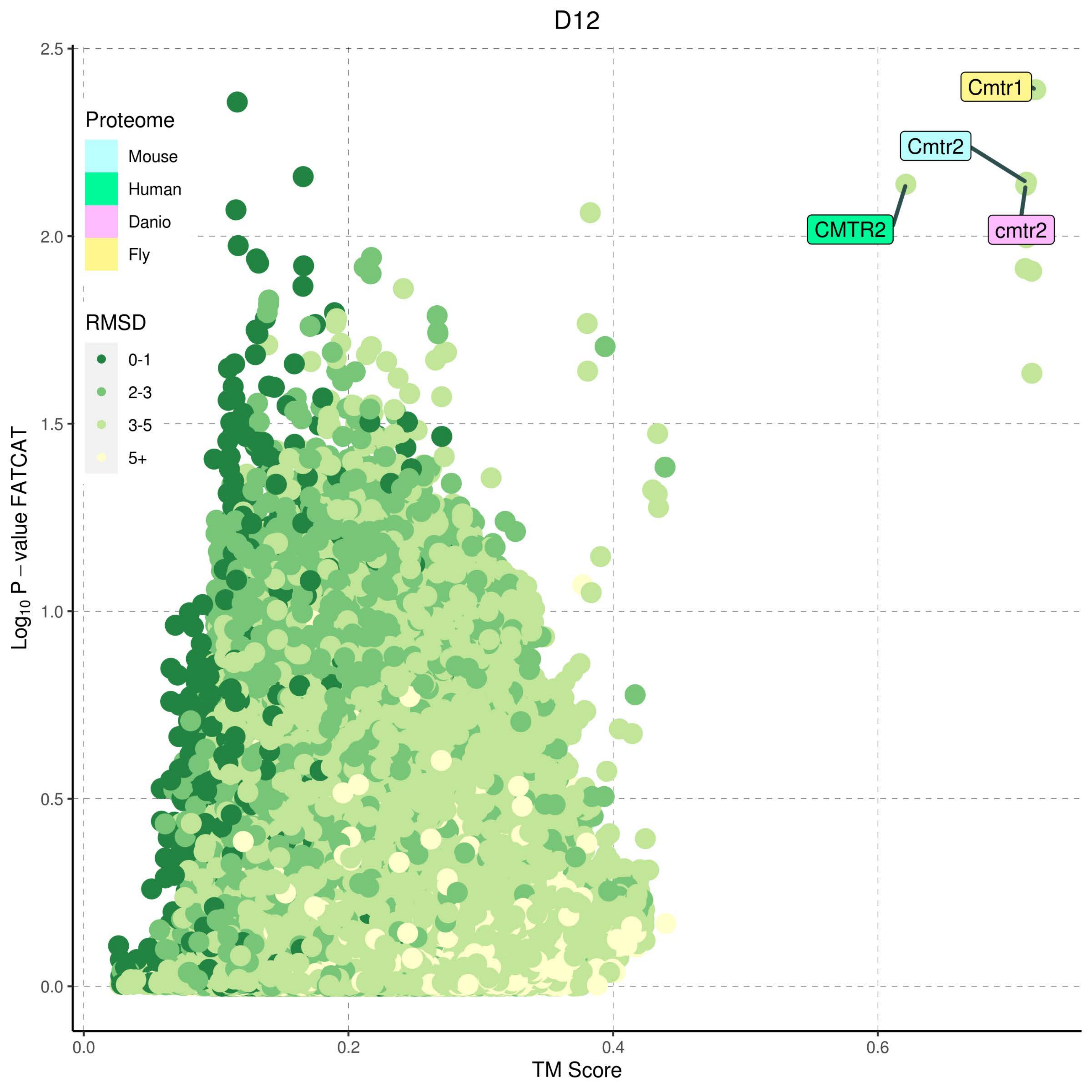

D12
Proteome
Mouse
Human
Danio
Fly
RMSD
0-1
2-3
3-5
5+
Cmtr1
Cmtr2
CMTR2
cmtr2
2.5
2.0
1.5
1.0
0.5
0.0
Log10 P − value FATCAT
0.0
0.2
0.4
0.6
TM Score

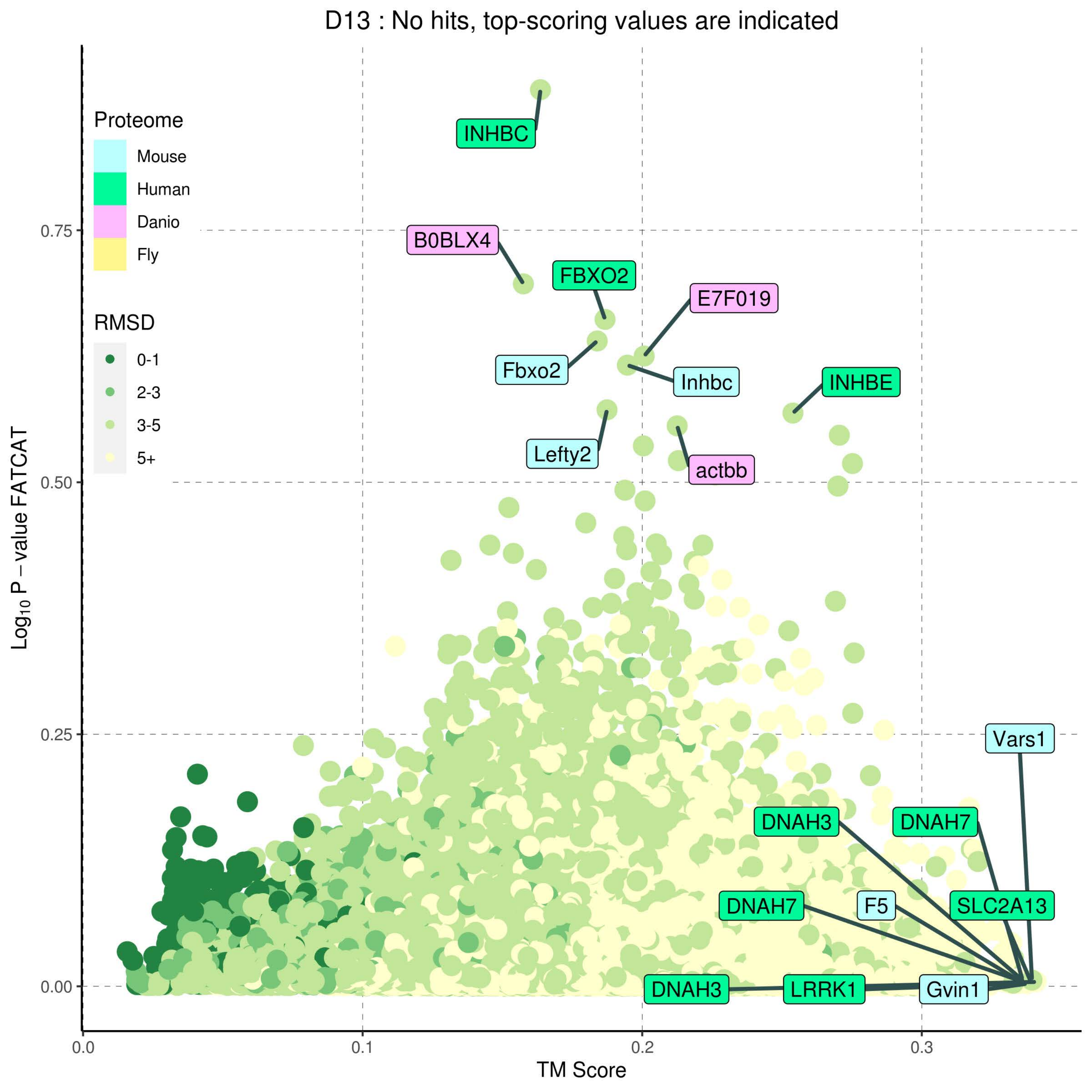

D13 : No hits, top-scoring values are indicated
Proteome
Mouse
Human
Danio
Fly
RMSD
0-1
2-3
3-5
5+
INHBC
B0BLX4
FBXO2
E7F019
Fbxo2
Inhbc
INHBE
Lefty2
actbb
Vars1
DNAH3
DNAH7
DNAH7
F5
SLC2A13
DNAH3
LRRK1
Gvin1
0.75
0.50
0.25
0.00
0.0
0.1
0.2
0.3
Log_{10} P − value FATCAT
TM Score

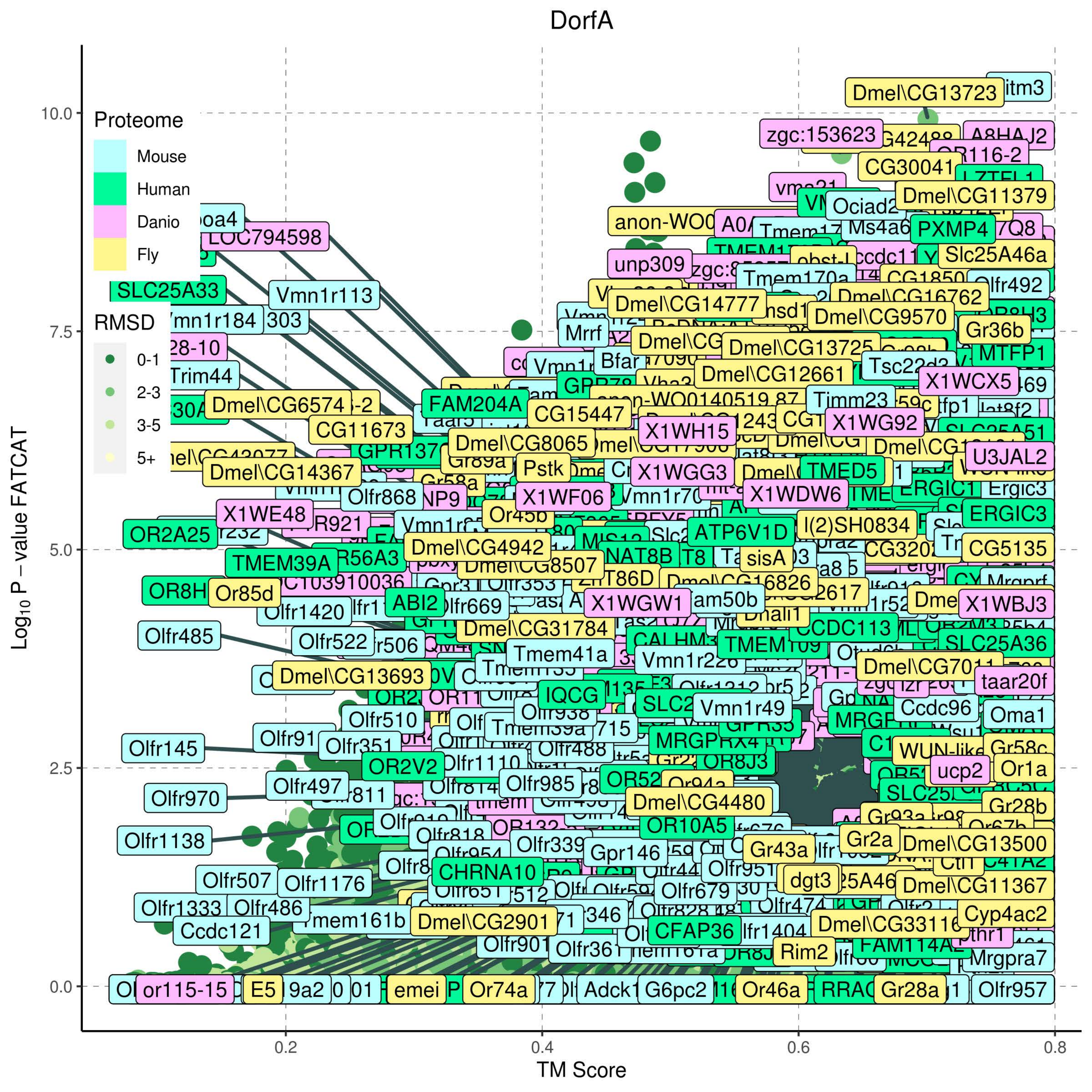
DorfA
Proteome
Mouse
Human
Danio
Fly
RMSD
0-1
2-3
3-5
5+
Log10 P – value FATCAT
TM Score
10.0
7.5
5.0
2.5
0.0
0.2
0.4
0.6
0.8
Dmel\CG13723
zgc:153623
CG30041
Dmel\CG11379
LOC794598
SLC25A33
Vmn1r113
Dmel\CG14777
Dmel\CG16762
Dmel\CG9570
Gr36b
Dmel\CG13725
Dmel\CG12661
MTFP1
X1WCX5
Timm23
FAM204A
CG15447
X1WH15
X1WG92
CG11673
Dmel\CG8065
U3JAL2
Dmel\CG14367
X1WGG3
TMED5
Olfr868
X1WF06
X1WDW6
ERGIC1
Ergic3
X1WE48
ERGIC3
OR2A25
ATP6V1D
l(2)SH0834
Dmel\CG4942
CG5135
TMEM39A
Or85d
X1WGW1
X1WBJ3
ABI2
Olfr669
Olfr1420
Olfr485
Dmel\CG31784
CCDC113
Olfr522
Tmem41a
SLC25A36
Dmel\CG13693
taar20f
IQCG
Vmn1r49
Ccdc96
Oma1
Olfr510
Olfr145
MRGPRX4
OR2V2
Olfr488
ucp2
Or1a
Olfr970
Olfr497
Olfr985
Dmel\CG4480
Gr28b
Olfr1138
OR10A5
Gr43a
Gr2a
Dmel\CG13500
Olfr339
Gpr146
Olfr507
Olfr1176
CHRNA10
dgt3
Dmel\CG11367
Olfr679
Olfr1333
Olfr486
Ccdc121
Dmel\CG2901
CFAP36
Cyp4ac2
Olfr901
Olfr361
Rim2
Mrgpra7
or115-15
emei
Or74a
Adck1
G6pc2
Or46a
Gr28a
Olfr957

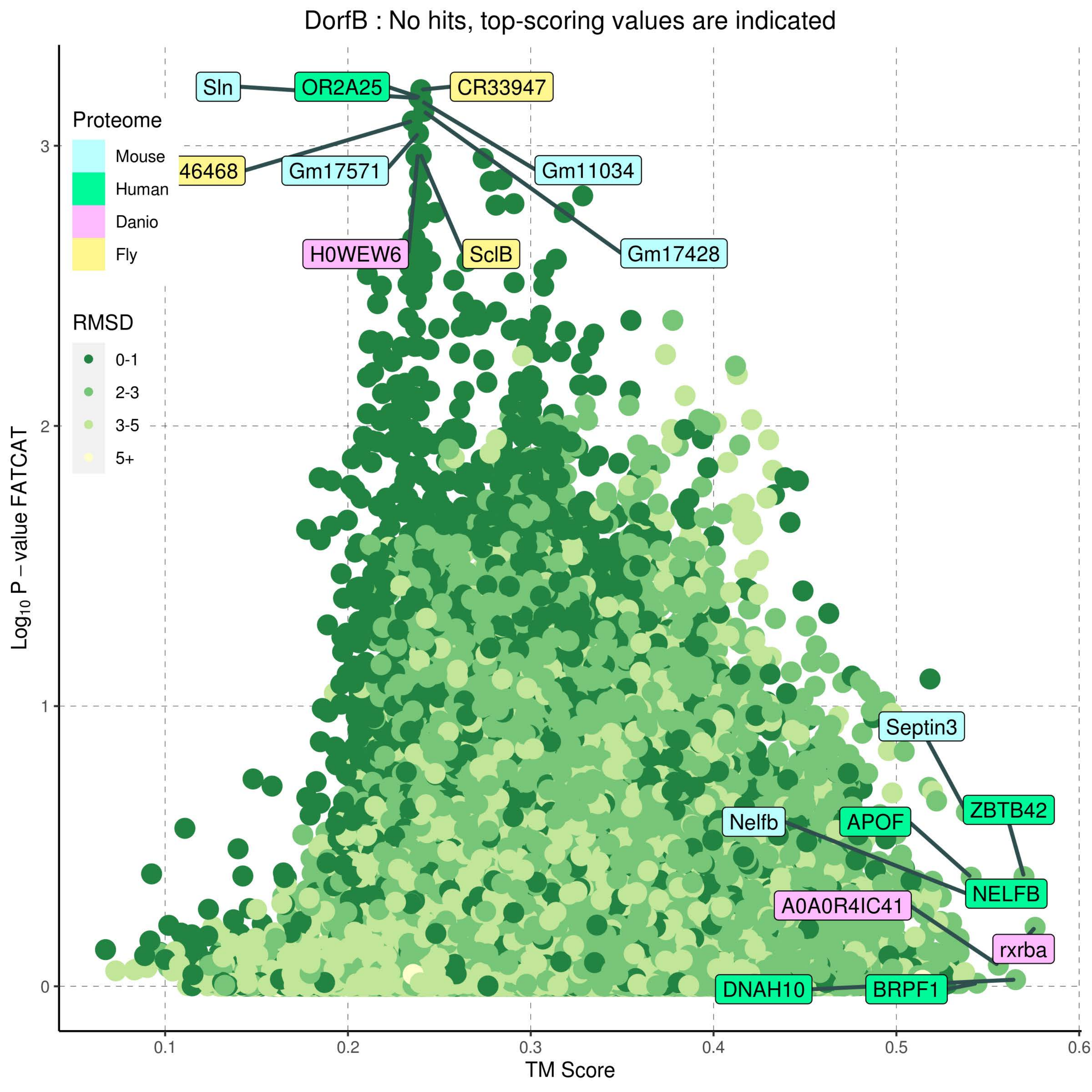

DorfB : No hits, top-scoring values are indicated
Sln
OR2A25
CR33947
46468
Gm17571
Gm11034
H0WEW6
SclB
Gm17428
Septin3
ZBTB42
Nelfb
APOF
NELFB
A0A0R4IC41
rxrba
DNAH10
BRPF1
Proteome
Mouse
Human
Danio
Fly
RMSD
0-1
2-3
3-5
5+
Log10 P − value FATCAT
TM Score
0
1
2
3
0.1
0.2
0.3
0.4
0.5
0.6

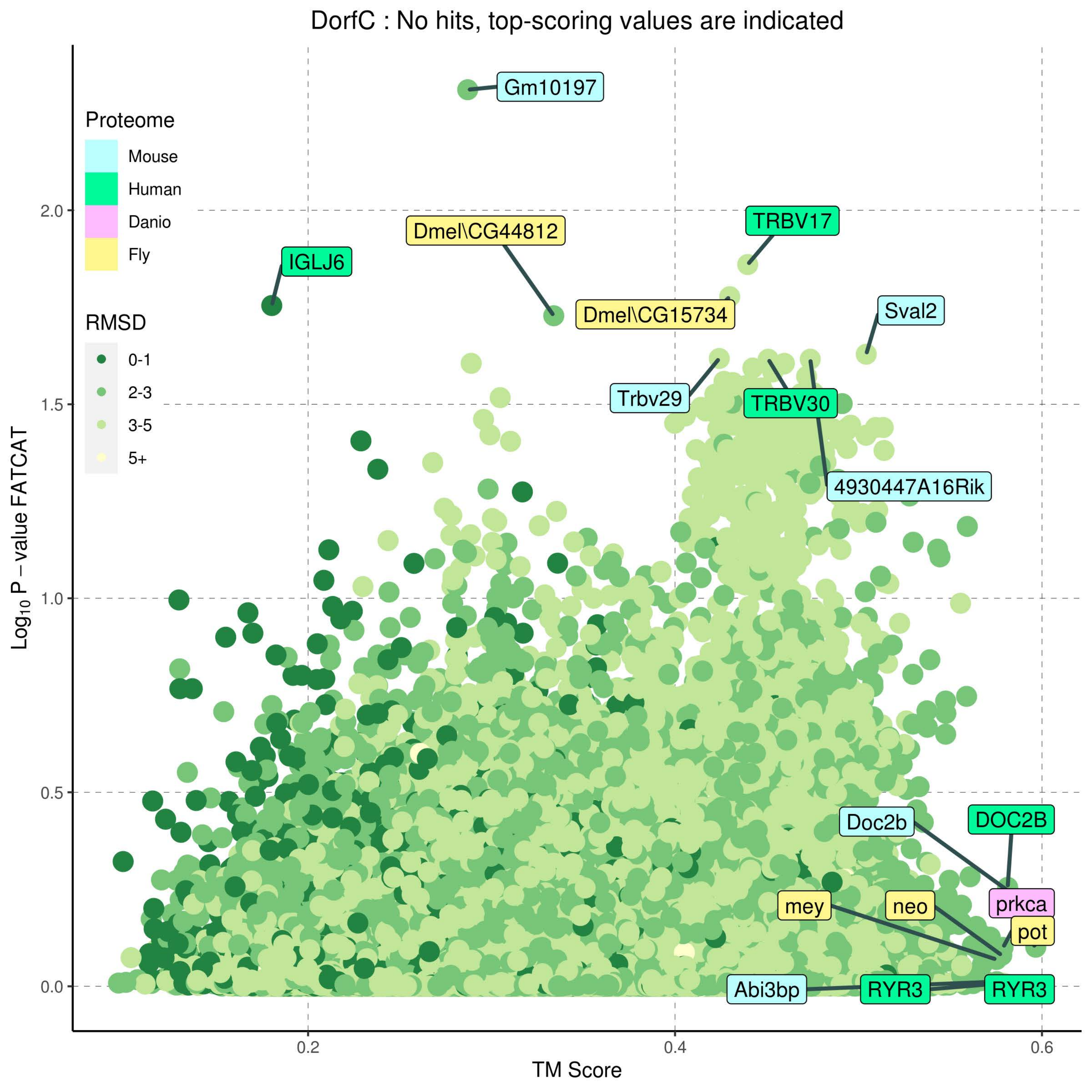

DorfC : No hits, top-scoring values are indicated
Proteome
Mouse
Human
Danio
Fly
RMSD
0-1
2-3
3-5
5+
Gm10197
Dmel\CG44812
TRBV17
IGLJ6
Dmel\CG15734
Sval2
Trbv29
TRBV30
4930447A16Rik
Doc2b
DOC2B
mey
neo
prkca
pot
Abi3bp
RYR3
RYR3
Log10 P − value FATCAT
TM Score
0.0
0.5
1.0
1.5
2.0
0.2
0.4
0.6

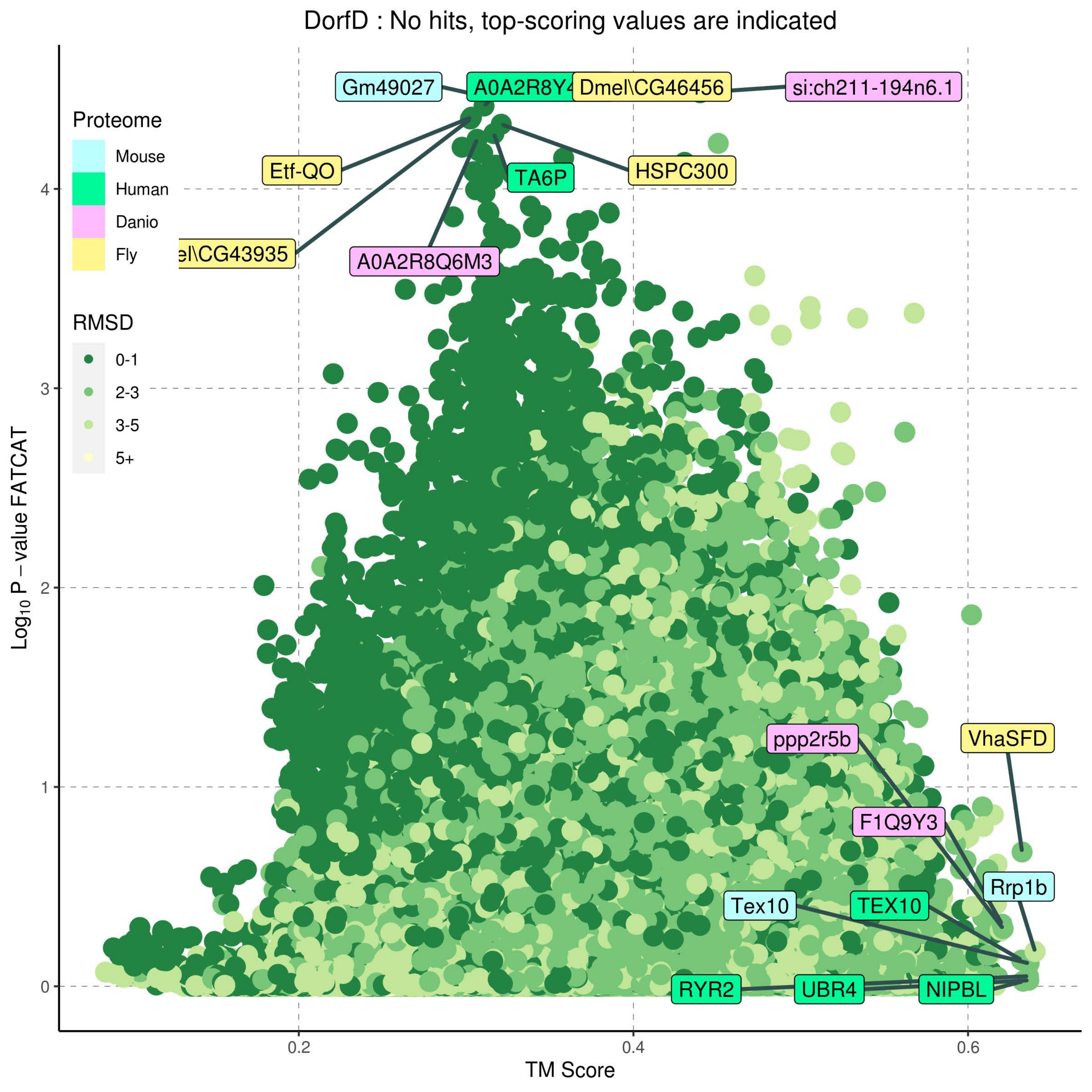

DorfD : No hits, top-scoring values are indicated
Gm49027
A0A2R8Y4
Dmel\CG46456
si:ch211-194n6.1
Etf-QO
TA6P
HSPC300
el\CG43935
A0A2R8Q6M3
Proteome
Mouse
Human
Danio
Fly
RMSD
0-1
2-3
3-5
5+
ppp2r5b
VhaSFD
F1Q9Y3
Rrp1b
Tex10
TEX10
RYR2
UBR4
NIPBL
Log10 P − value FATCAT
TM Score
0
1
2
3
4
0.2
0.4
0.6

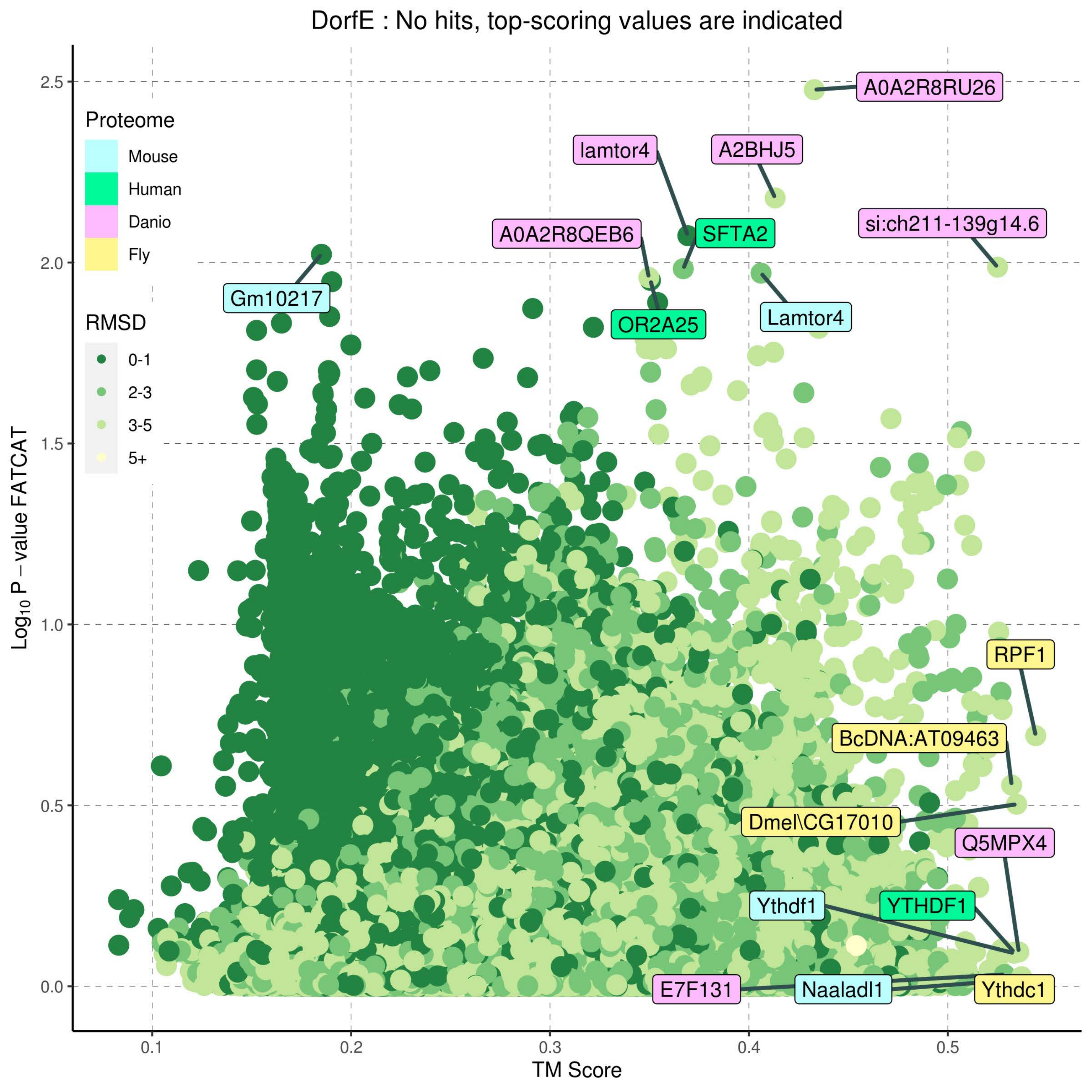
DorfE : No hits, top-scoring values are indicated
Proteome
Mouse
Human
Danio
Fly
RMSD
0-1
2-3
3-5
5+
A0A2R8RU26
lamtor4
A2BHJ5
A0A2R8QEB6
SFTA2
si:ch211-139g14.6
Gm10217
OR2A25
Lamtor4
RPF1
BcDNA:AT09463
Dmel\CG17010
Q5MPX4
Ythdf1
YTHDF1
E7F131
Naaladl1
Ythdc1
Log10 P – value FATCAT
TM Score
2.5
2.0
1.5
1.0
0.5
0.0
0.1
0.2
0.3
0.4
0.5

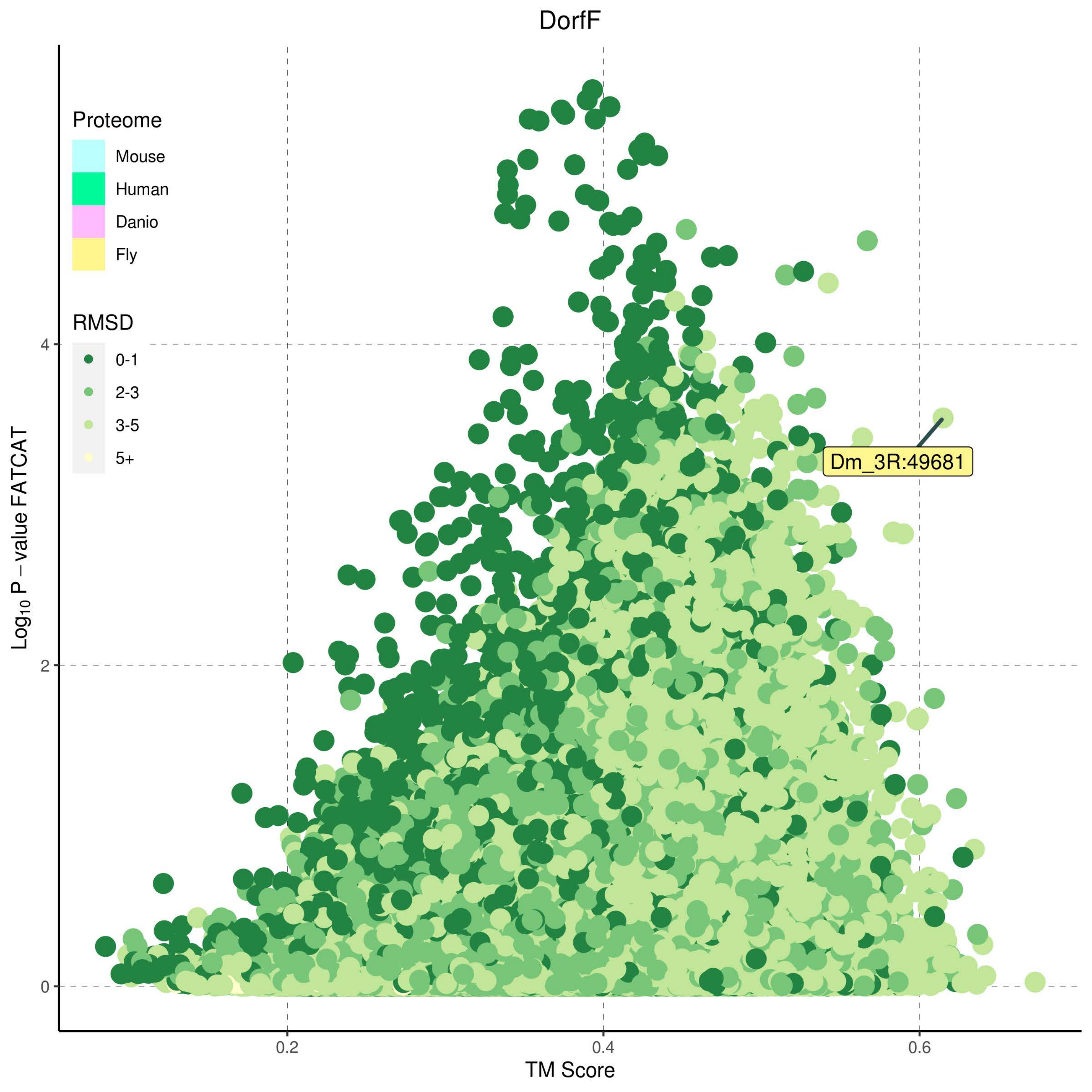
DorfF
Proteome
Mouse
Human
Danio
Fly
RMSD
0-1
2-3
3-5
5+
Dm_3R:49681
Log10 P − value FATCAT
TM Score
0
2
4
0.2
0.4
0.6

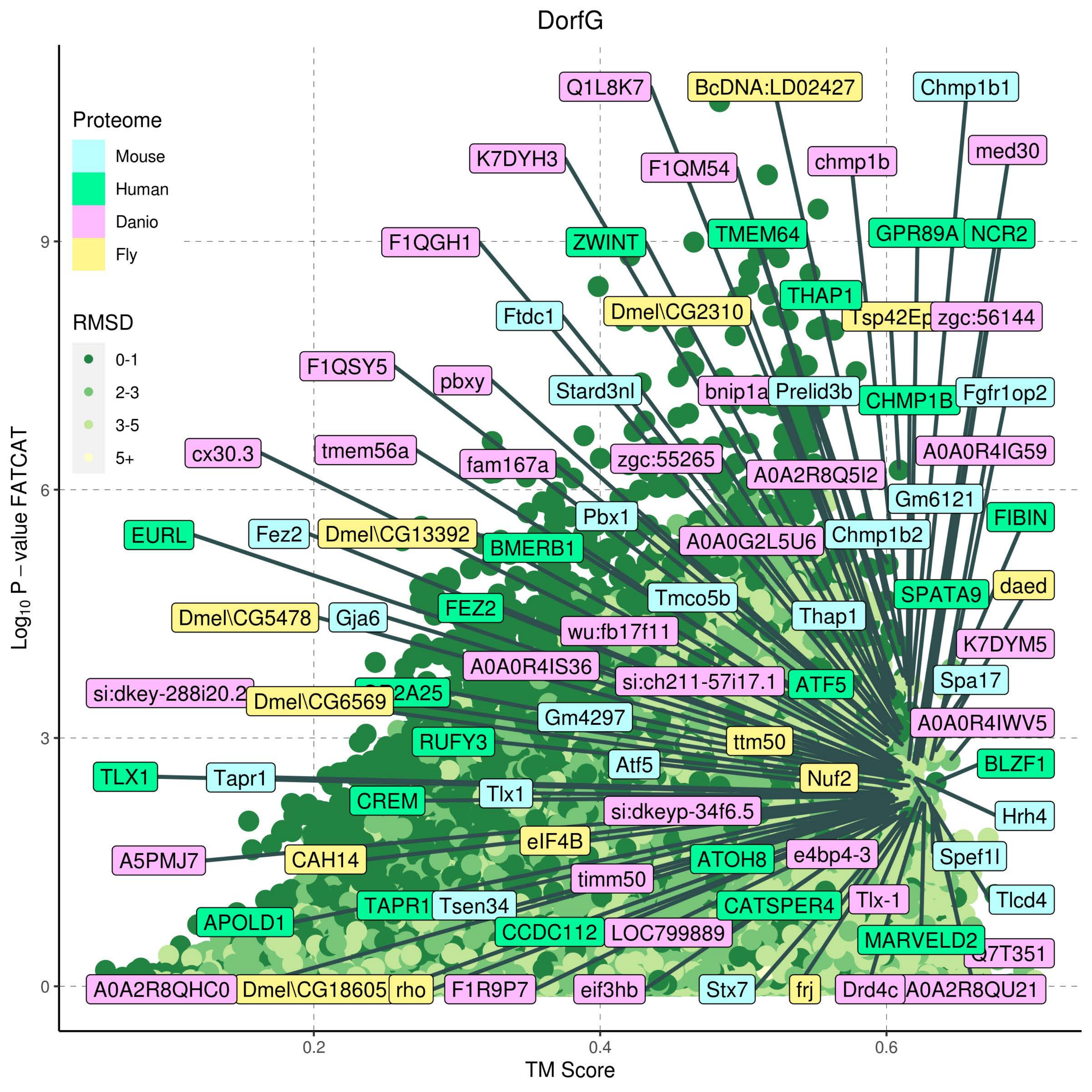
DorfG
Proteome
Mouse
Human
Danio
Fly
RMSD
0-1
2-3
3-5
5+
Log10 P − value FATCAT
TM Score
0
3
6
9
0.2
0.4
0.6
Q1L8K7
BcDNA:LD02427
Chmp1b1
K7DYH3
F1QM54
chmp1b
med30
F1QGH1
ZWINT
TMEM64
GPR89A
NCR2
THAP1
Ftdc1
Dmel\CG2310
zgc:56144
F1QSY5
pbxy
Stard3nl
bnip1a
Prelid3b
CHMP1B
Fgfr1op2
cx30.3
tmem56a
fam167a
zgc:55265
A0A2R8Q5I2
A0A0R4IG59
Gm6121
EURL
Fez2
Dmel\CG13392
Pbx1
BMERB1
A0A0G2L5U6
Chmp1b2
FIBIN
Tmco5b
SPATA9
daed
Dmel\CG5478
Gja6
FEZ2
Thap1
wu:fb17f11
K7DYM5
A0A0R4IS36
si:ch211-57i17.1
ATF5
Spa17
si:dkey-288i20.2
Dmel\CG6569
Gm4297
A0A0R4IWV5
RUFY3
ttm50
TLX1
Tapr1
Atf5
BLZF1
Nuf2
CREM
Tlx1
si:dkeyp-34f6.5
Hrh4
eIF4B
A5PMJ7
CAH14
ATOH8
e4bp4-3
Spef1l
timm50
TAPR1
Tsen34
CATSPER4
Tlx-1
Tlcd4
APOLD1
CCDC112
LOC799889
MARVELD2
Q7T351
A0A2R8QHC0
Dmel\CG18605
rho
F1R9P7
eif3hb
Stx7
frj
Drd4c
A0A2R8QU21

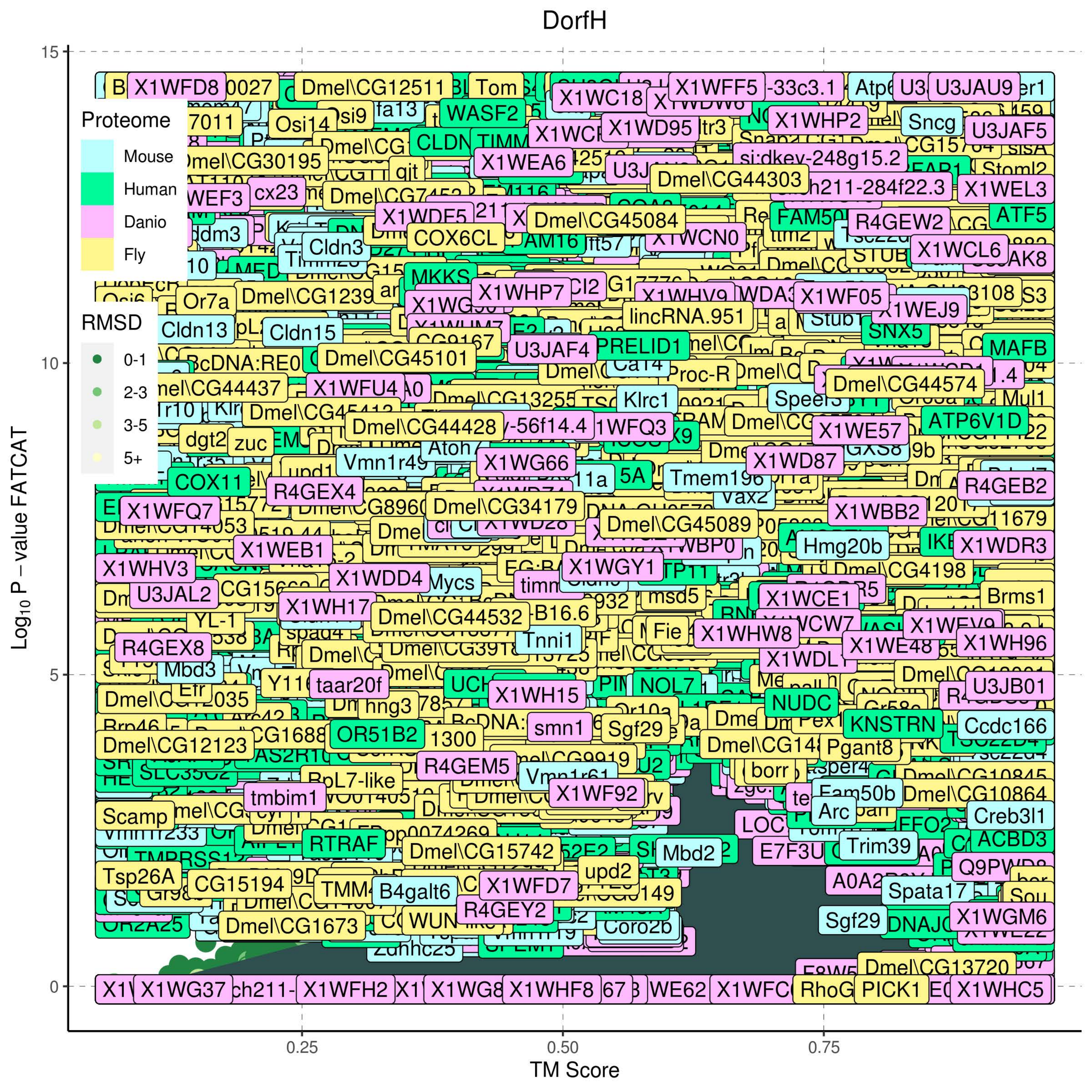
DorfH
Proteome
Mouse
Human
Danio
Fly
RMSD
0-1
2-3
3-5
5+
Log_{10} P – value FATCAT
TM Score
0
5
10
15
0.25
0.50
0.75

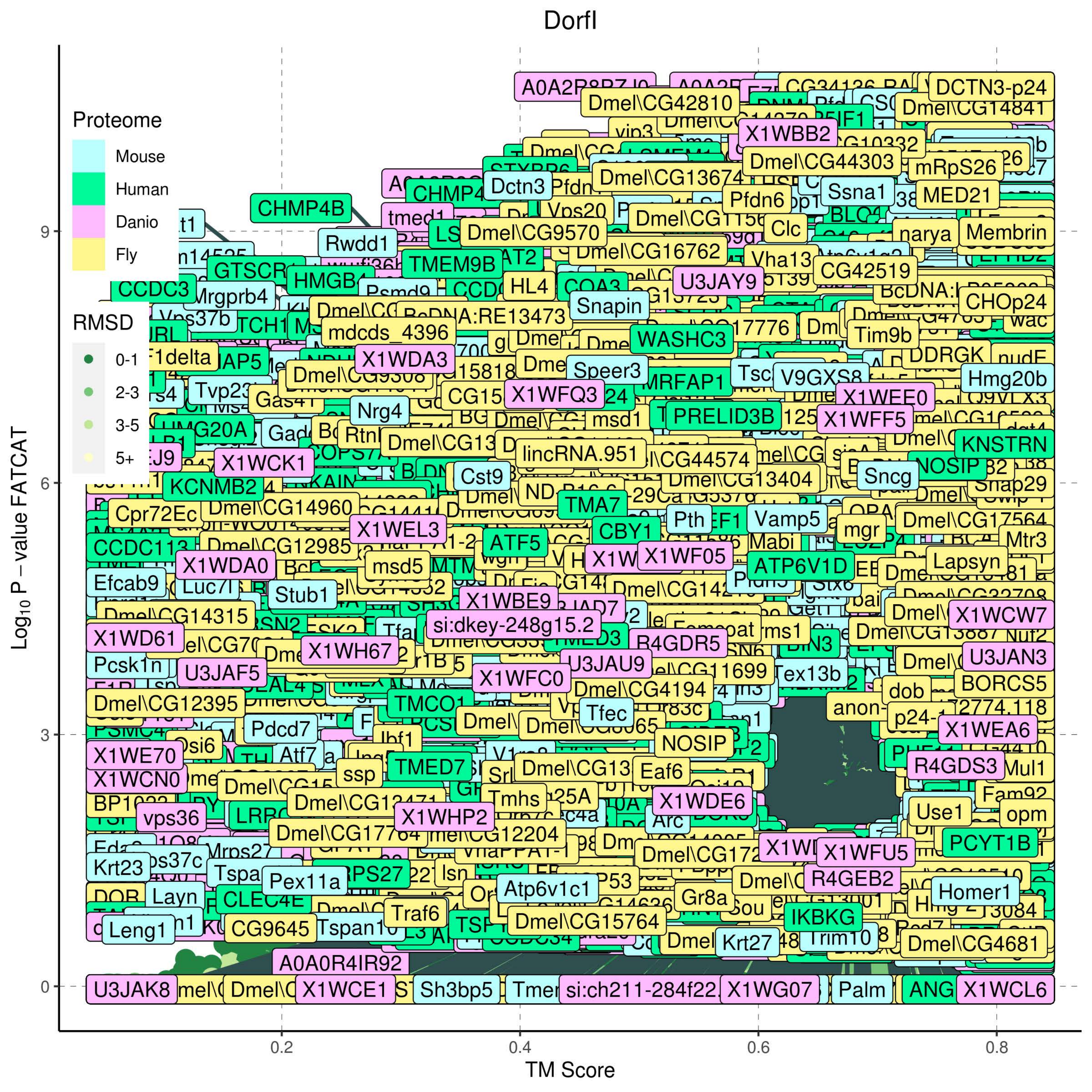

Dorfl
Proteome
Mouse
Human
Danio
Fly
RMSD
0-1
2-3
3-5
5+
Log_{10} P – value FATCAT
TM Score
0.2
0.4
0.6
0.8
0
3
6
9
DCTN3-p24
Dmel\CG42810
Dmel\CG14841
X1WBB2
Dmel\CG44303
mRpS26
Dmel\CG13674
Dctn3
Ssna1
MED21
CHMP4B
Dmel\CG9570
Clc
narya
Membrin
Rwdd1
Dmel\CG16762
Vha13
TMEM9B
HL4
U3JAY9
CG42519
GTSCR
CCDC3
Mrgprb4
Snapin
CHOp24
BcDNA:RE13473
mdcds_4396
WASHC3
Tim9b
X1WDA3
Speer3
DDRGK
Dmel\CG9308
Hmg20b
X1WFQ3
MRFAP1
V9GXS8
X1WEE0
Tvp23
Nrg4
PRELID3B
X1WFF5
HMG20A
lincRNA.951
KNSTRN
X1WCK1
Cst9
Sncg
NOSIP
KCNMB2
Dmel\CG13404
Snap29
Cpr72Ec
Dmel\CG14960
TMA7
Pth
Vamp5
Dmel\CG17564
X1WEL3
CBY1
mgr
CCDC113
Dmel\CG12985
ATF5
Mtr3
X1WDA0
X1WF05
Lapsyn
msd5
ATP6V1D
Efcab9
Stub1
X1WBE9
Dmel\CG14315
si:dkey-248g15.2
X1WCW7
X1WD61
X1WH67
R4GDR5
U3JAU9
Pcsk1n
U3JAF5
X1WFC0
Tex13b
U3JAN3
Dmel\CG4194
BORCS5
Dmel\CG12395
TMCO1
dob
Tfec
Pdcd7
X1WEA6
NOSIP
X1WE70
TMED7
Eaf6
R4GDS3
X1WCN0
Mul1
ssp
X1WDE6
vps36
Tmhs
Use1
opm
X1WHP2
Dmel\CG17764
PCYT1B
X1WFU5
Krt23
Pex11a
R4GEB2
Atp6v1c1
Gr8a
Homer1
Traf6
IKBKG
Leng1
CG9645
Dmel\CG15764
Krt27
Trim10
Dmel\CG4681
A0A0R4IR92
U3JAK8
X1WCE1
Sh3bp5
X1WG07
Palm
X1WCL6

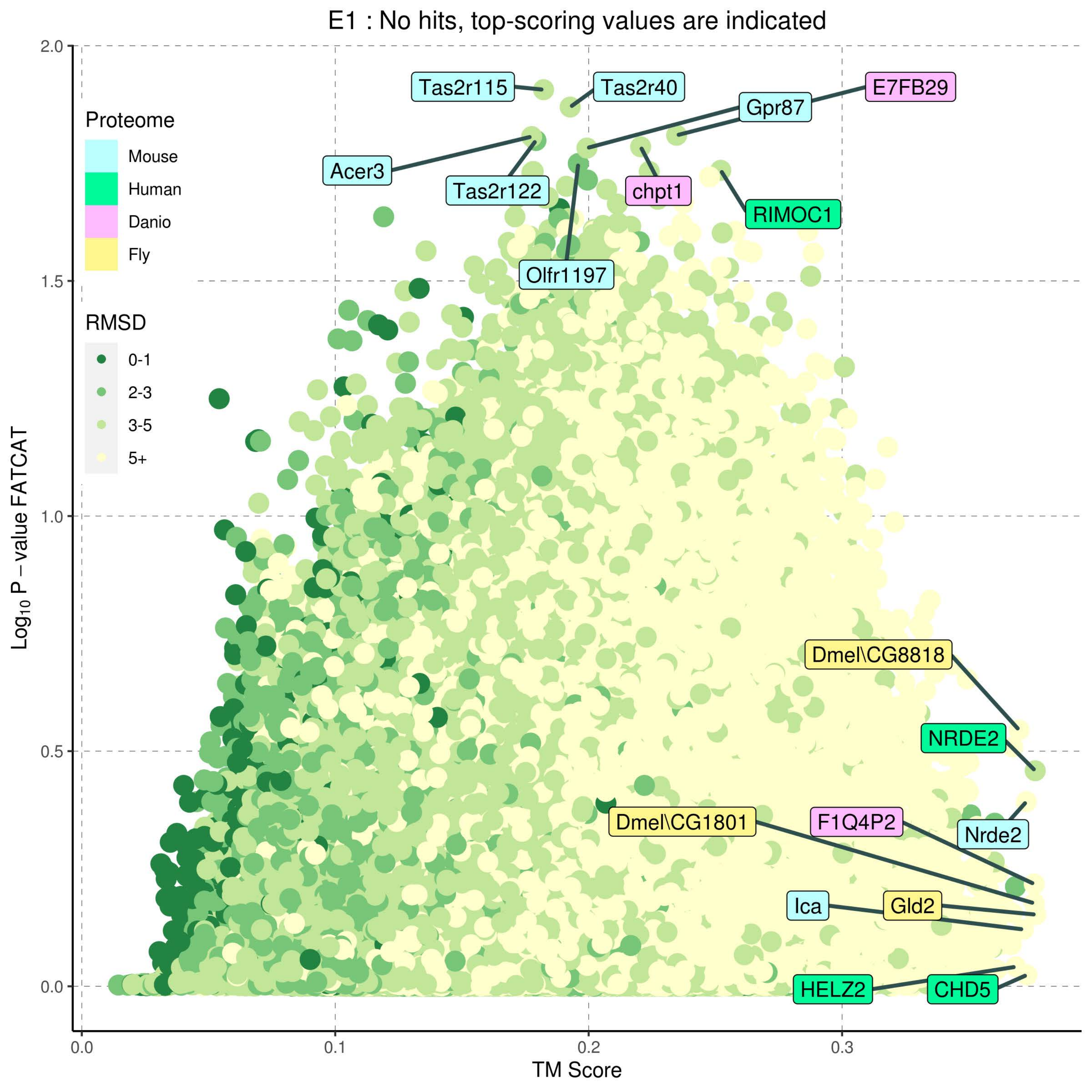
E1 : No hits, top-scoring values are indicated
Proteome
Mouse
Human
Danio
Fly
RMSD
0-1
2-3
3-5
5+
Log10 P – value FATCAT
TM Score
0.0
0.1
0.2
0.3
0.5
1.0
1.5
2.0
Tas2r115
Tas2r40
E7FB29
Gpr87
Acer3
Tas2r122
chpt1
RIMOC1
Olfr1197
Dmel\CG8818
NRDE2
Dmel\CG1801
F1Q4P2
Nrde2
Ica
Gld2
HELZ2
CHD5

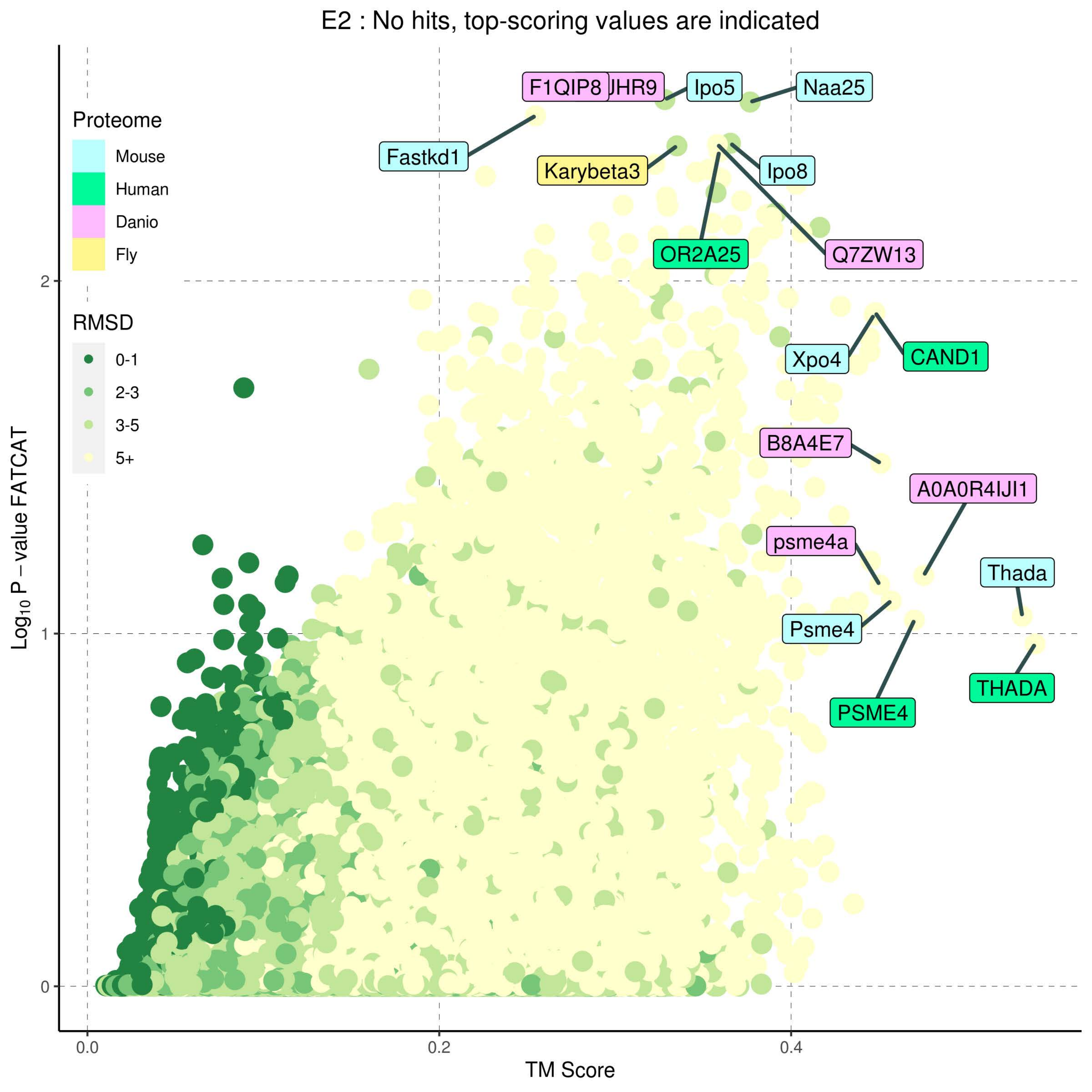
E2 : No hits, top-scoring values are indicated
Proteome
Mouse
Human
Danio
Fly
RMSD
0-1
2-3
3-5
5+
F1QIP8
JHR9
Ipo5
Naa25
Fastkd1
Karybeta3
Ipo8
OR2A25
Q7ZW13
Xpo4
CAND1
B8A4E7
A0A0R4IJI1
psme4a
Thada
Psme4
PSME4
THADA
Log10 P − value FATCAT
TM Score
0.0
0.2
0.4
0
1
2

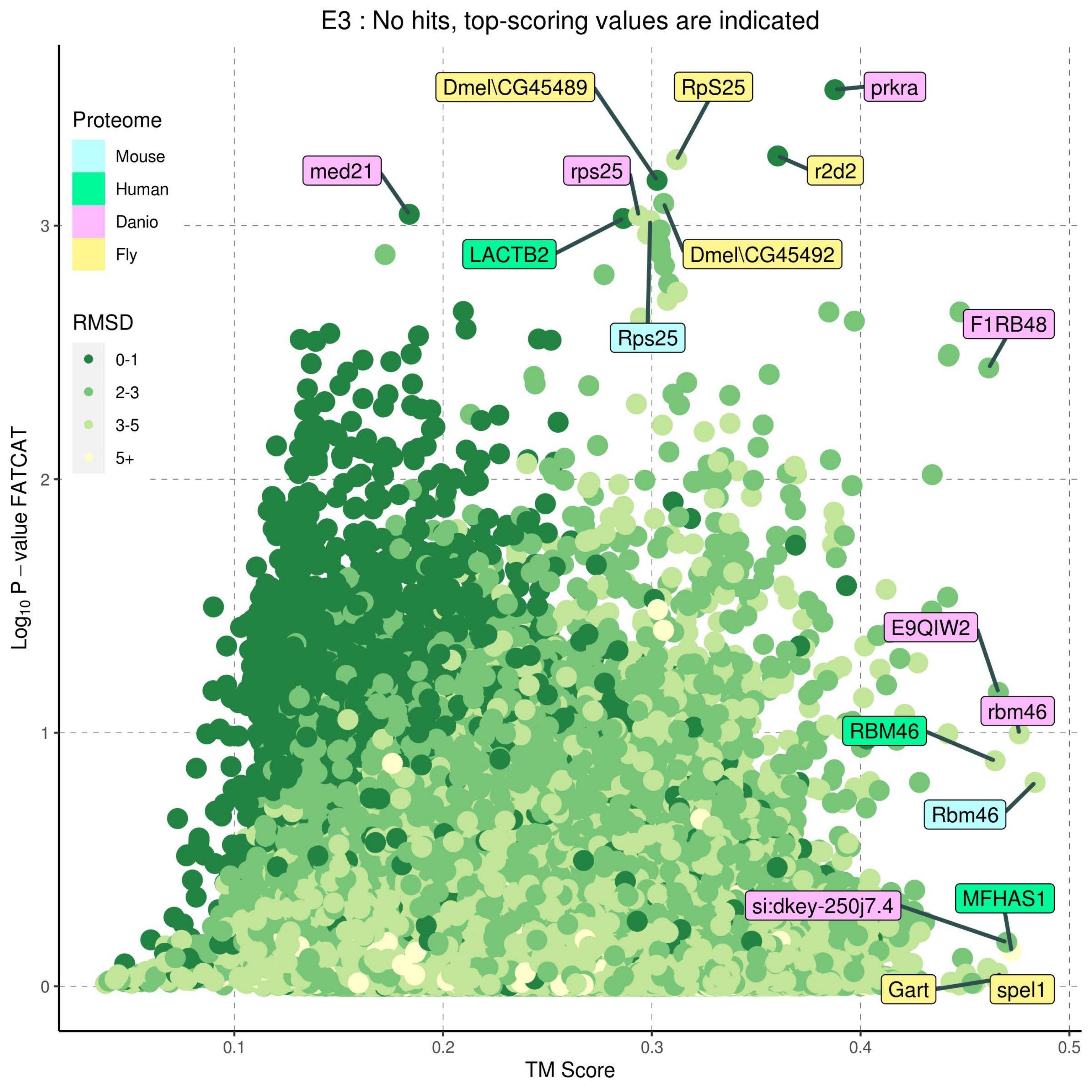

E3 : No hits, top-scoring values are indicated
Proteome
Mouse
Human
Danio
Fly
RMSD
0-1
2-3
3-5
5+
Dmel\CG45489
RpS25
prkra
med21
rps25
r2d2
LACTB2
Dmel\CG45492
Rps25
F1RB48
E9QIW2
rbm46
RBM46
Rbm46
MFHAS1
si:dkey-250j7.4
Gart
spel1
Log10 P − value FATCAT
TM Score
0
1
2
3
0.1
0.2
0.3
0.4
0.5

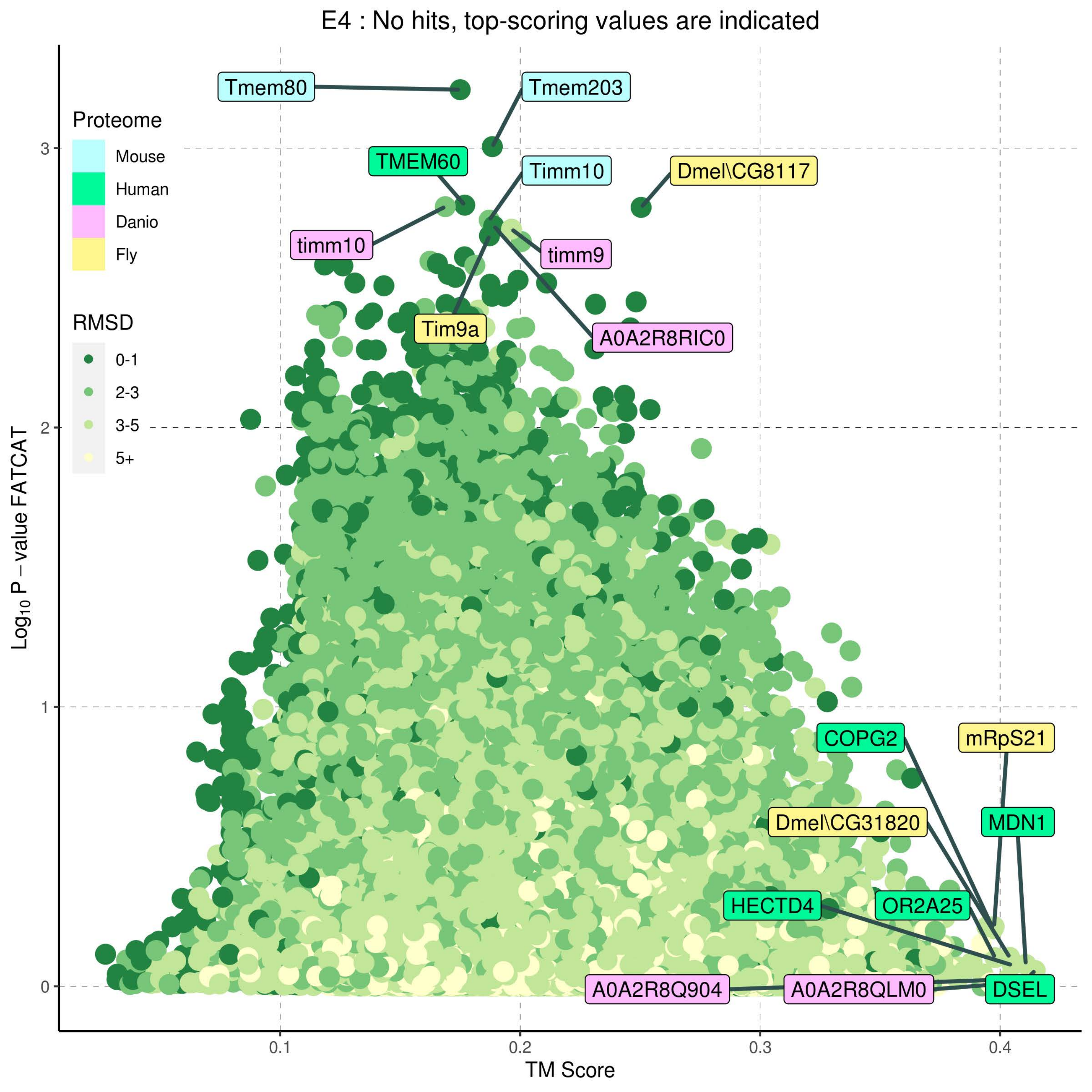
E4 : No hits, top-scoring values are indicated
Proteome
Mouse
Human
Danio
Fly
RMSD
0-1
2-3
3-5
5+
Tmem80
Tmem203
TMEM60
Timm10
Dmel\CG8117
timm10
timm9
Tim9a
A0A2R8RIC0
COPG2
mRpS21
Dmel\CG31820
MDN1
HECTD4
OR2A25
A0A2R8Q904
A0A2R8QLM0
DSEL
0
1
2
3
0.1
0.2
0.3
0.4
TM Score
Log10 P – value FATCAT

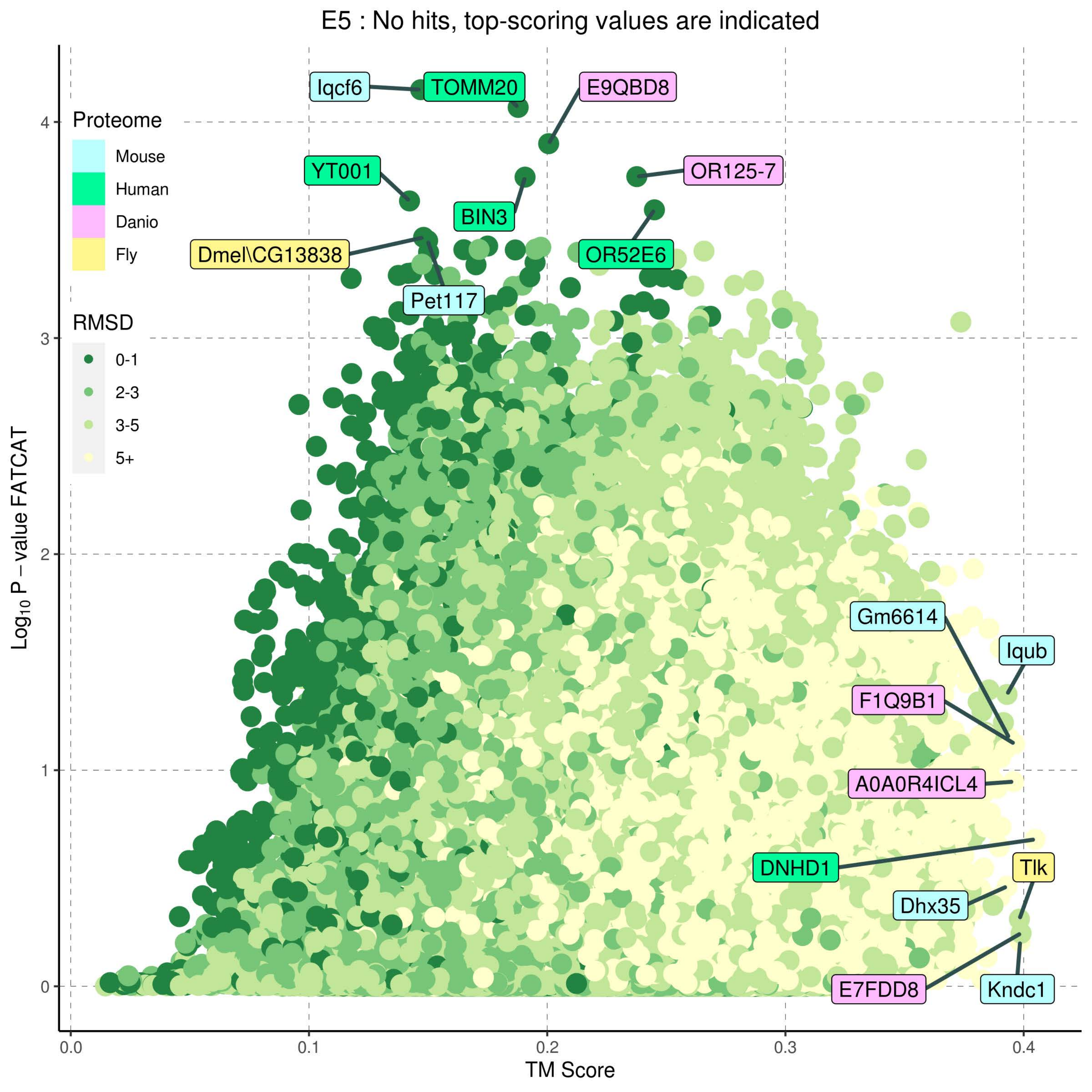

E5 : No hits, top-scoring values are indicated
Proteome
Mouse
Human
Danio
Fly
RMSD
0-1
2-3
3-5
5+
Iqcf6
TOMM20
E9QBD8
YT001
OR125-7
BIN3
Dmel\CG13838
OR52E6
Pet117
Gm6614
Iqub
F1Q9B1
A0A0R4ICL4
DNHD1
Tlk
Dhx35
E7FDD8
Kndc1
Log_{10} P − value FATCAT
TM Score
0
1
2
3
4
0.0
0.1
0.2
0.3
0.4

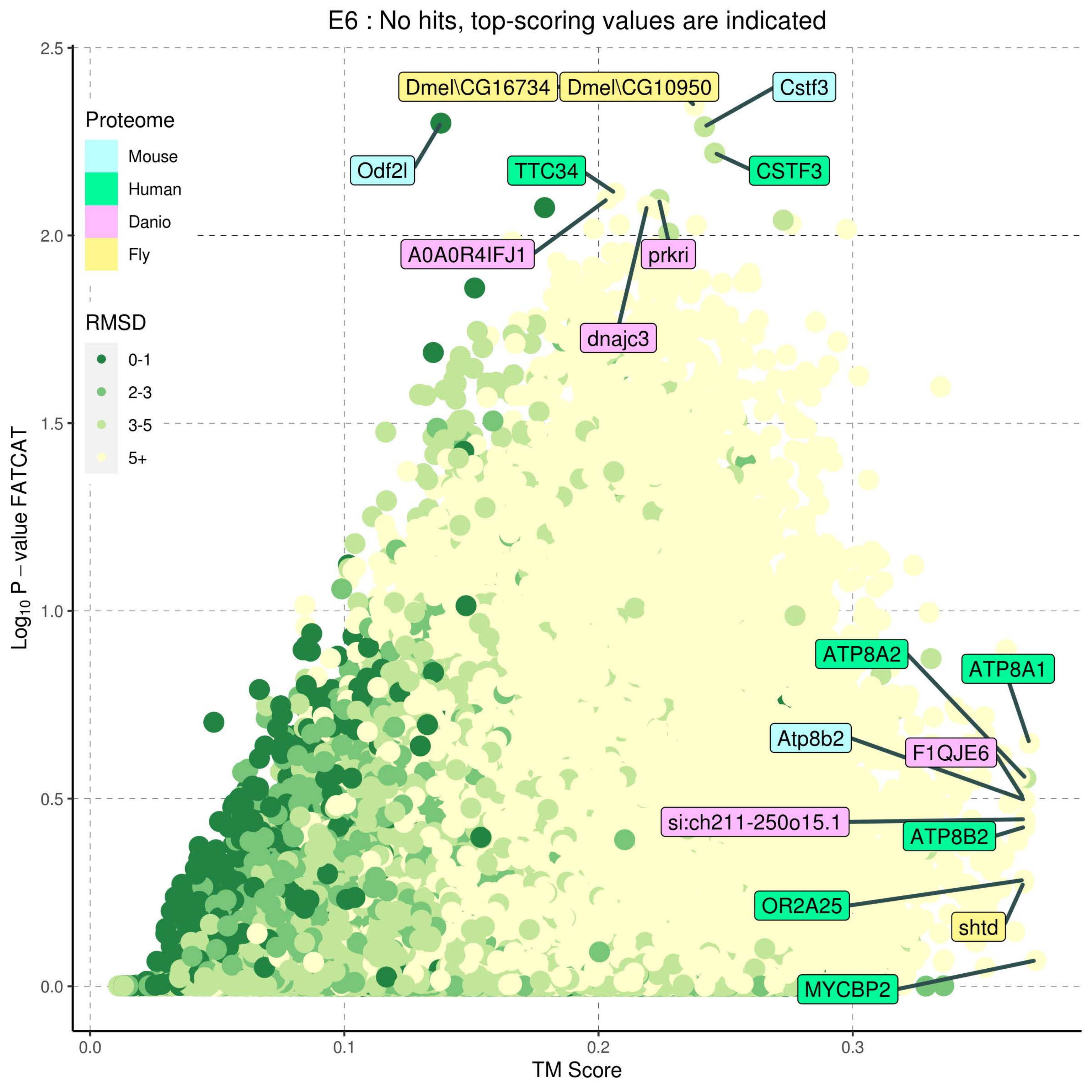

E6 : No hits, top-scoring values are indicated
Proteome
Mouse
Human
Danio
Fly
RMSD
0-1
2-3
3-5
5+
Dmel\CG16734
Dmel\CG10950
Cstf3
Odf2l
TTC34
CSTF3
A0A0R4IFJ1
prkri
dnajc3
ATP8A2
ATP8A1
Atp8b2
F1QJE6
si:ch211-250o15.1
ATP8B2
OR2A25
shtd
MYCBP2
0.0
0.5
1.0
1.5
2.0
2.5
0.0
0.1
0.2
0.3
Log10 P − value FATCAT
TM Score

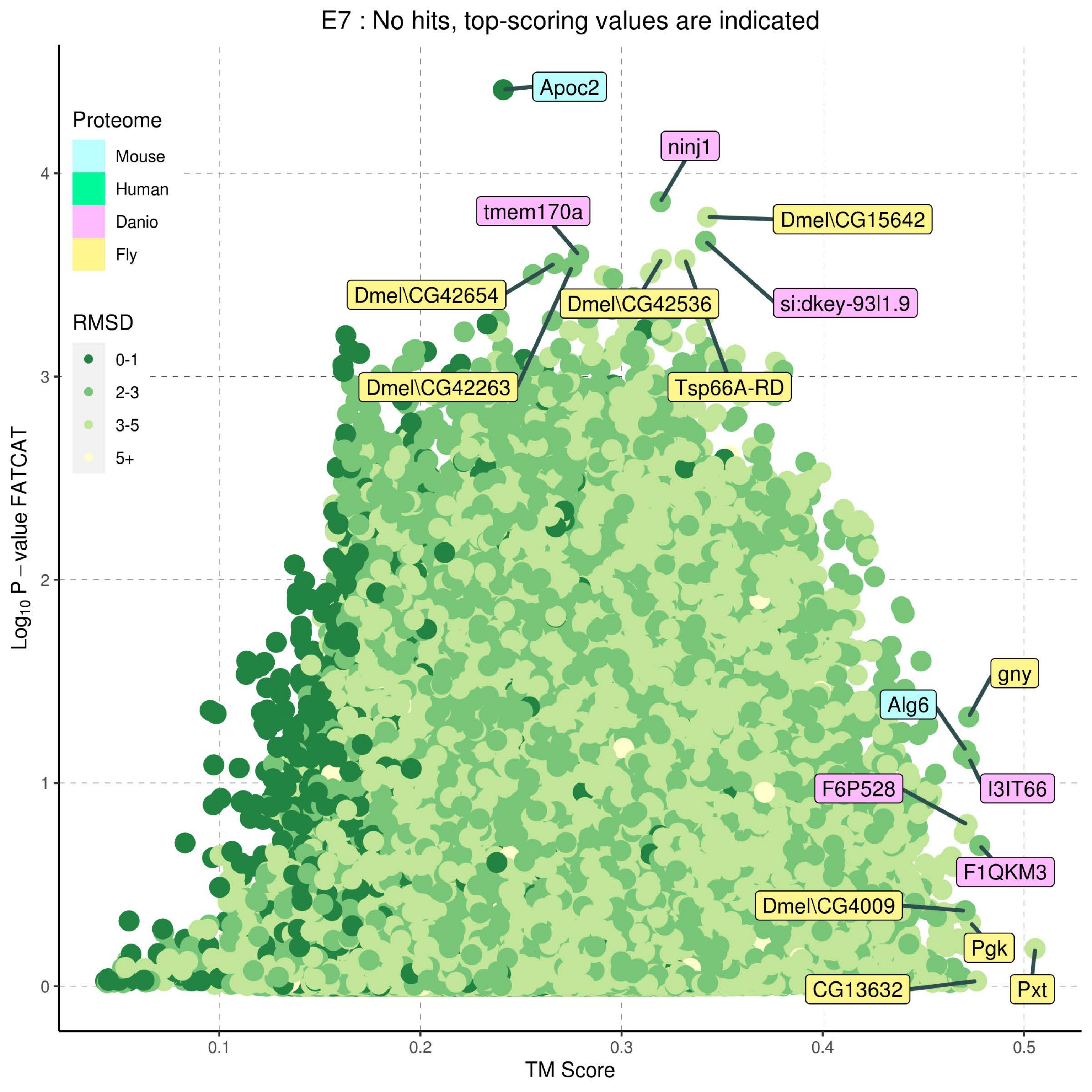
E7 : No hits, top-scoring values are indicated
Proteome
Mouse
Human
Danio
Fly
RMSD
0-1
2-3
3-5
5+
Apoc2
ninj1
tmem170a
Dmel\CG15642
Dmel\CG42654
Dmel\CG42536
si:dkey-93l1.9
Dmel\CG42263
Tsp66A-RD
gny
Alg6
I3IT66
F6P528
F1QKM3
Dmel\CG4009
Pgk
CG13632
Pxt
Log10 P – value FATCAT
TM Score
0
1
2
3
4
0.1
0.2
0.3
0.4
0.5

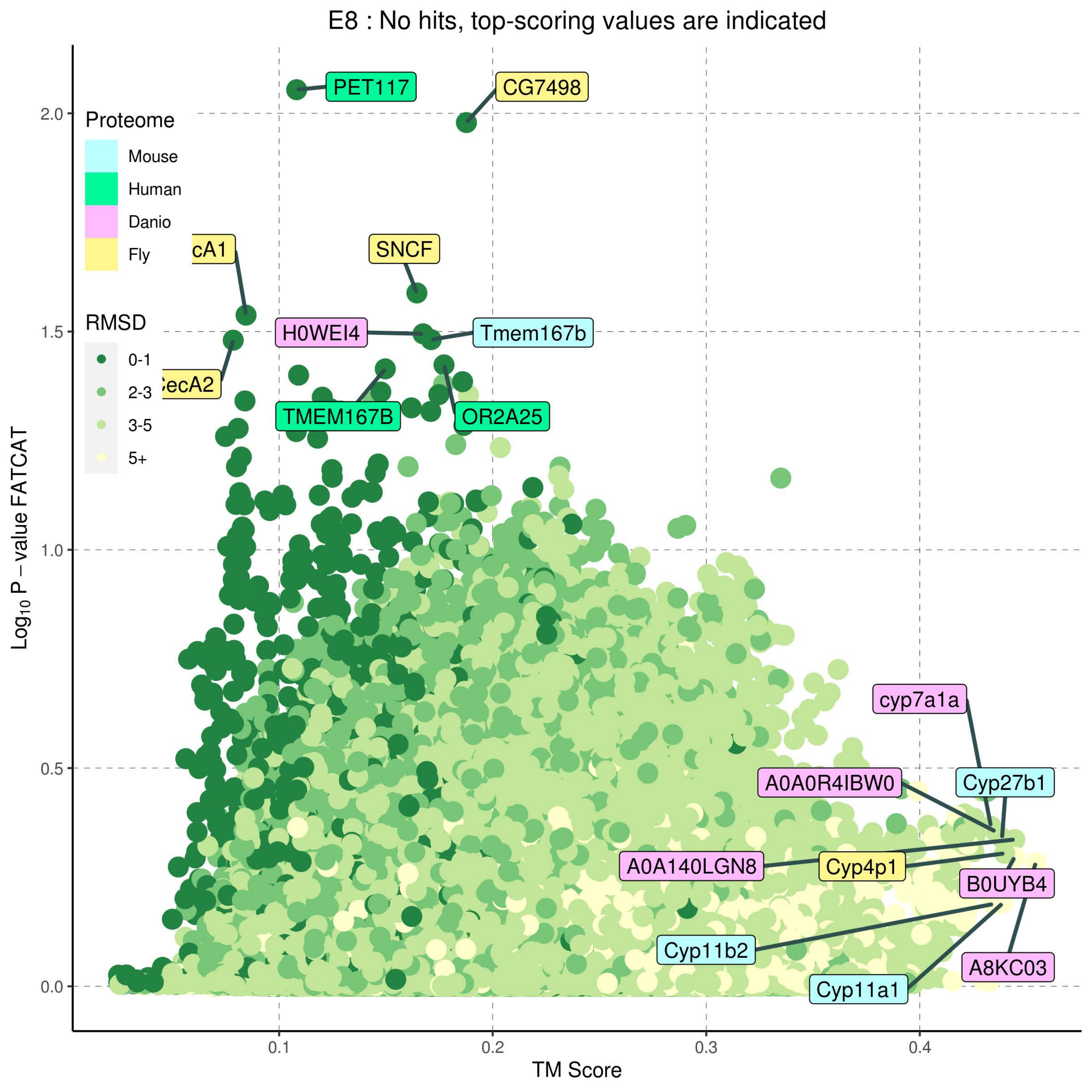

E8 : No hits, top-scoring values are indicated
Proteome
Mouse
Human
Danio
Fly
RMSD
0-1
2-3
3-5
5+
PET117
CG7498
cA1
SNCF
H0WEI4
Tmem167b
ecA2
TMEM167B
OR2A25
cyp7a1a
A0A0R4IBW0
Cyp27b1
A0A140LGN8
Cyp4p1
B0UYB4
Cyp11b2
A8KC03
Cyp11a1
Log_{10} P − value FATCAT
TM Score
0.0
0.5
1.0
1.5
2.0
0.1
0.2
0.3
0.4

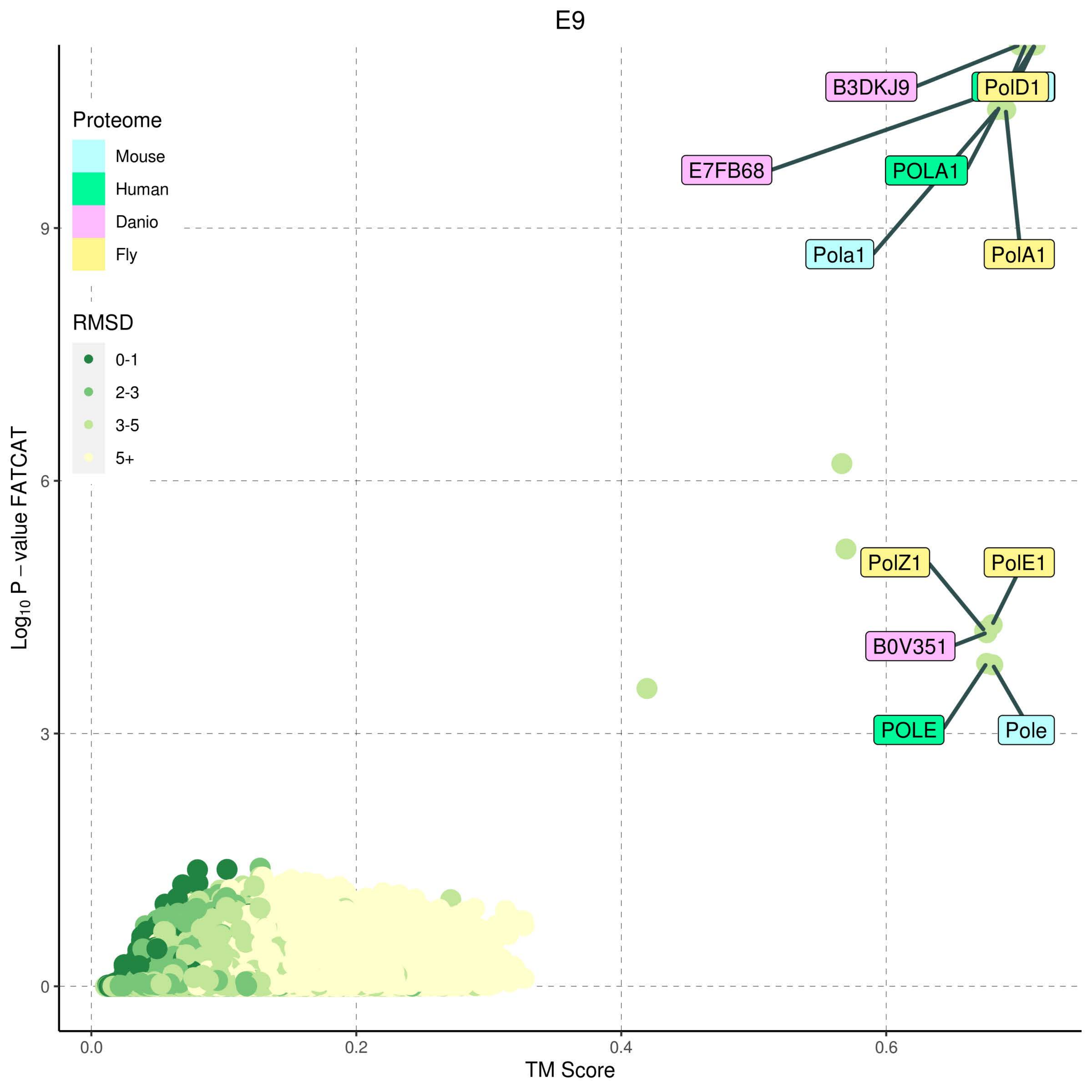

E9
Proteome
Mouse
Human
Danio
Fly
RMSD
0-1
2-3
3-5
5+
B3DKJ9
PolD1
E7FB68
POLA1
Pola1
PolA1
PolZ1
PolE1
B0V351
POLE
Pole
0
3
6
9
0.0
0.2
0.4
0.6
TM Score
Log_{10} P − value FATCAT

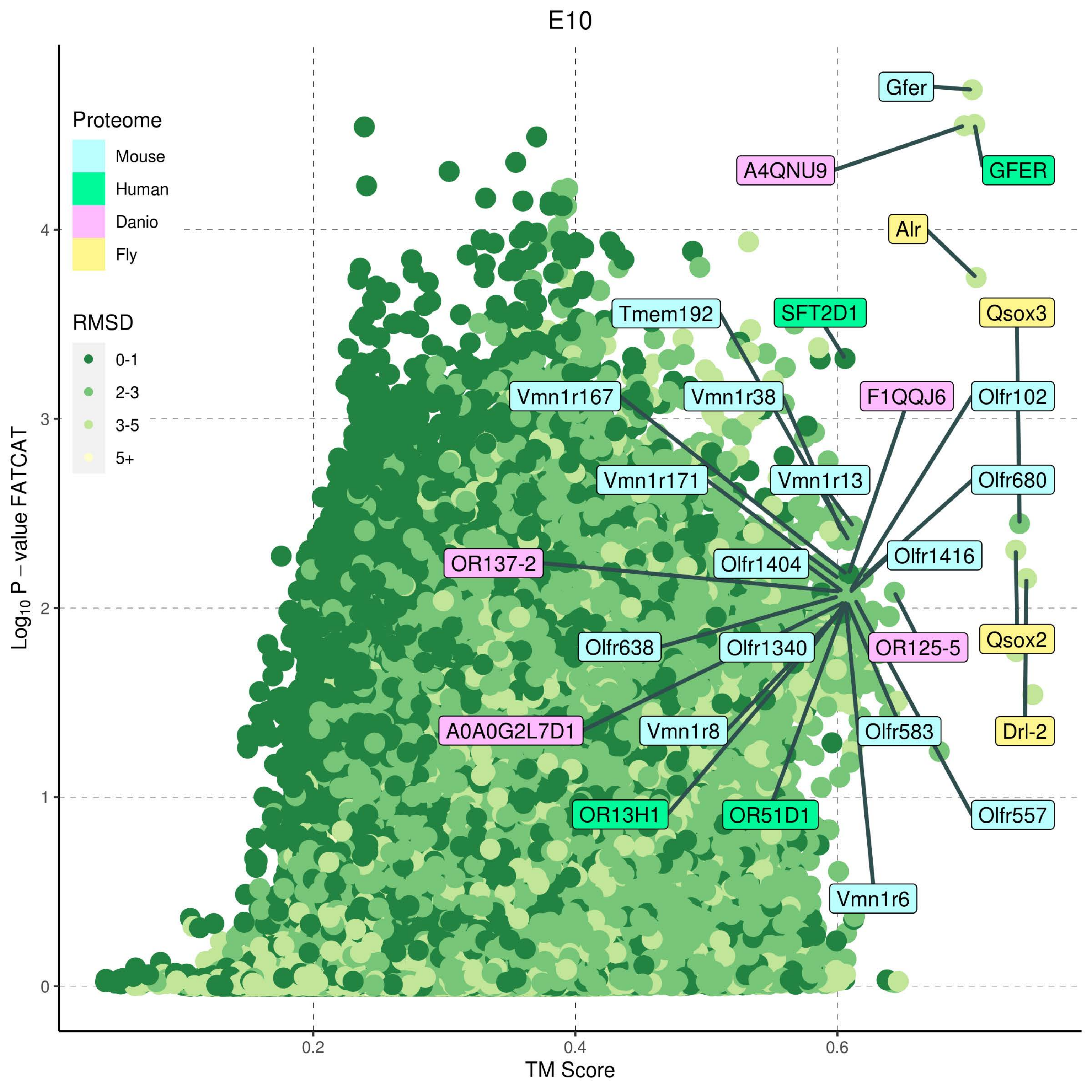

E10
Proteome
Mouse
Human
Danio
Fly
RMSD
0-1
2-3
3-5
5+
Gfer
A4QNU9
GFER
Alr
Tmem192
SFT2D1
Qsox3
Vmn1r167
Vmn1r38
F1QQJ6
Olfr102
Vmn1r171
Vmn1r13
Olfr680
OR137-2
Olfr1404
Olfr1416
Olfr638
Olfr1340
OR125-5
Qsox2
A0A0G2L7D1
Vmn1r8
Olfr583
Drl-2
OR13H1
OR51D1
Olfr557
Vmn1r6
Log_{10} P − value FATCAT
TM Score
0
1
2
3
4
0.2
0.4
0.6

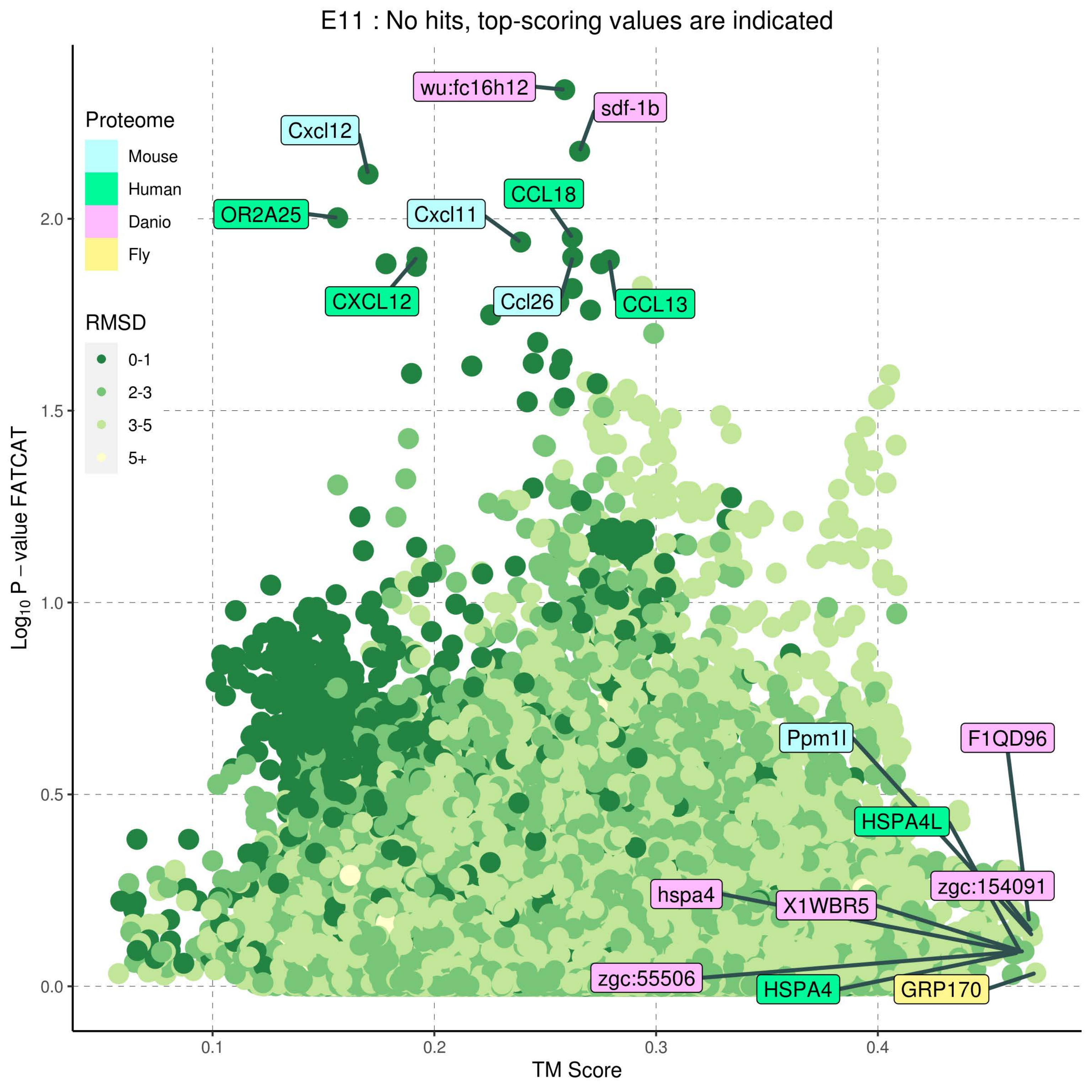

E11 : No hits, top-scoring values are indicated
Proteome
Mouse
Human
Danio
Fly
RMSD
0-1
2-3
3-5
5+
wu:fc16h12
sdf-1b
Cxcl12
OR2A25
Cxcl11
CCL18
CXCL12
Ccl26
CCL13
Ppm1l
F1QD96
HSPA4L
zgc:154091
hspa4
X1WBR5
zgc:55506
HSPA4
GRP170
Log_{10} P − value FATCAT
TM Score
0.0
0.5
1.0
1.5
2.0
0.1
0.2
0.3
0.4

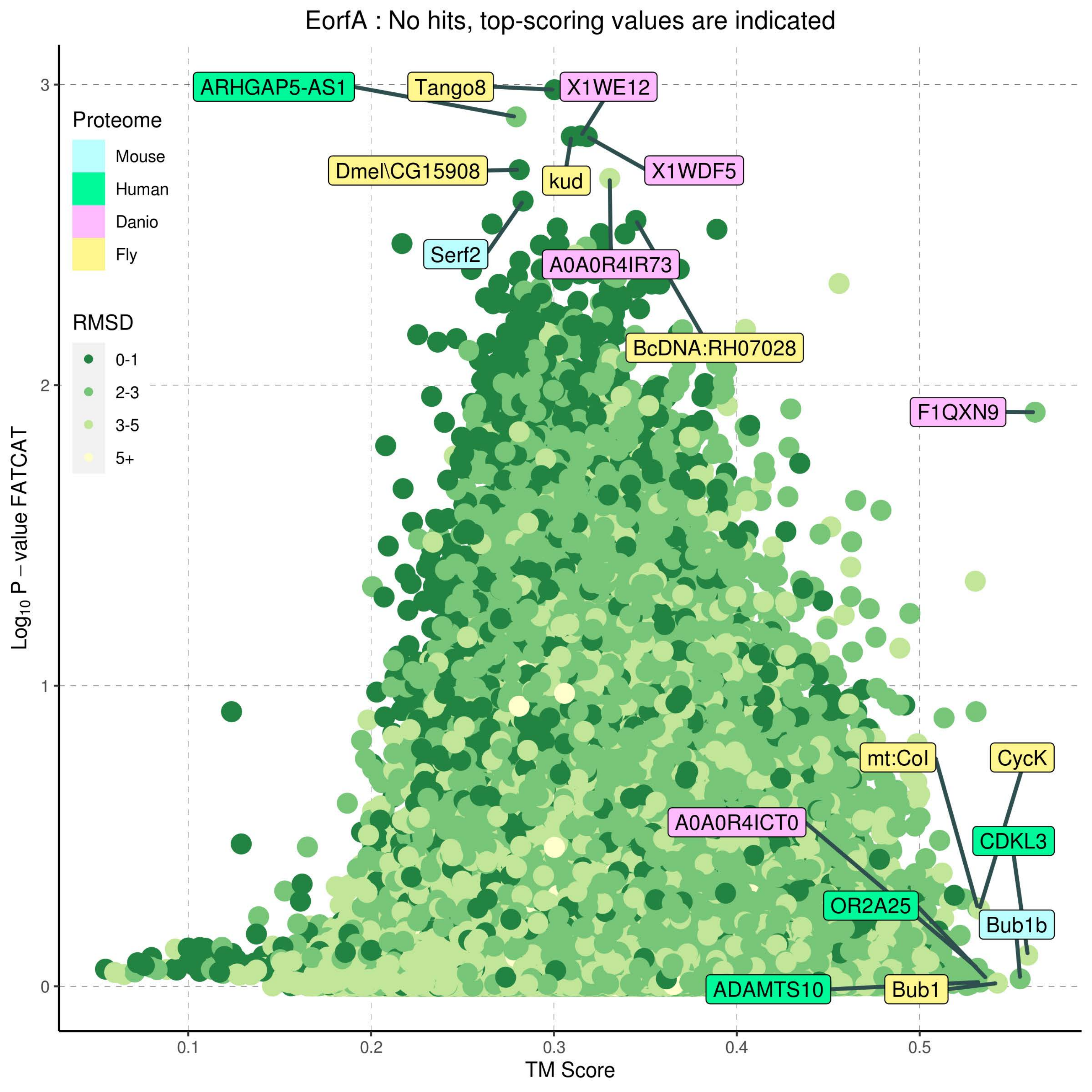
EorfA : No hits, top-scoring values are indicated
Proteome
Mouse
Human
Danio
Fly
RMSD
0-1
2-3
3-5
5+
ARHGAP5-AS1
Tango8
X1WE12
X1WDF5
kud
Dmel\CG15908
Serf2
A0A0R4IR73
BcDNA:RH07028
F1QXN9
mt:CoI
CycK
CDKL3
A0A0R4ICT0
OR2A25
Bub1b
ADAMTS10
Bub1
Log10 P − value FATCAT
TM Score
0
1
2
3
0.1
0.2
0.3
0.4
0.5

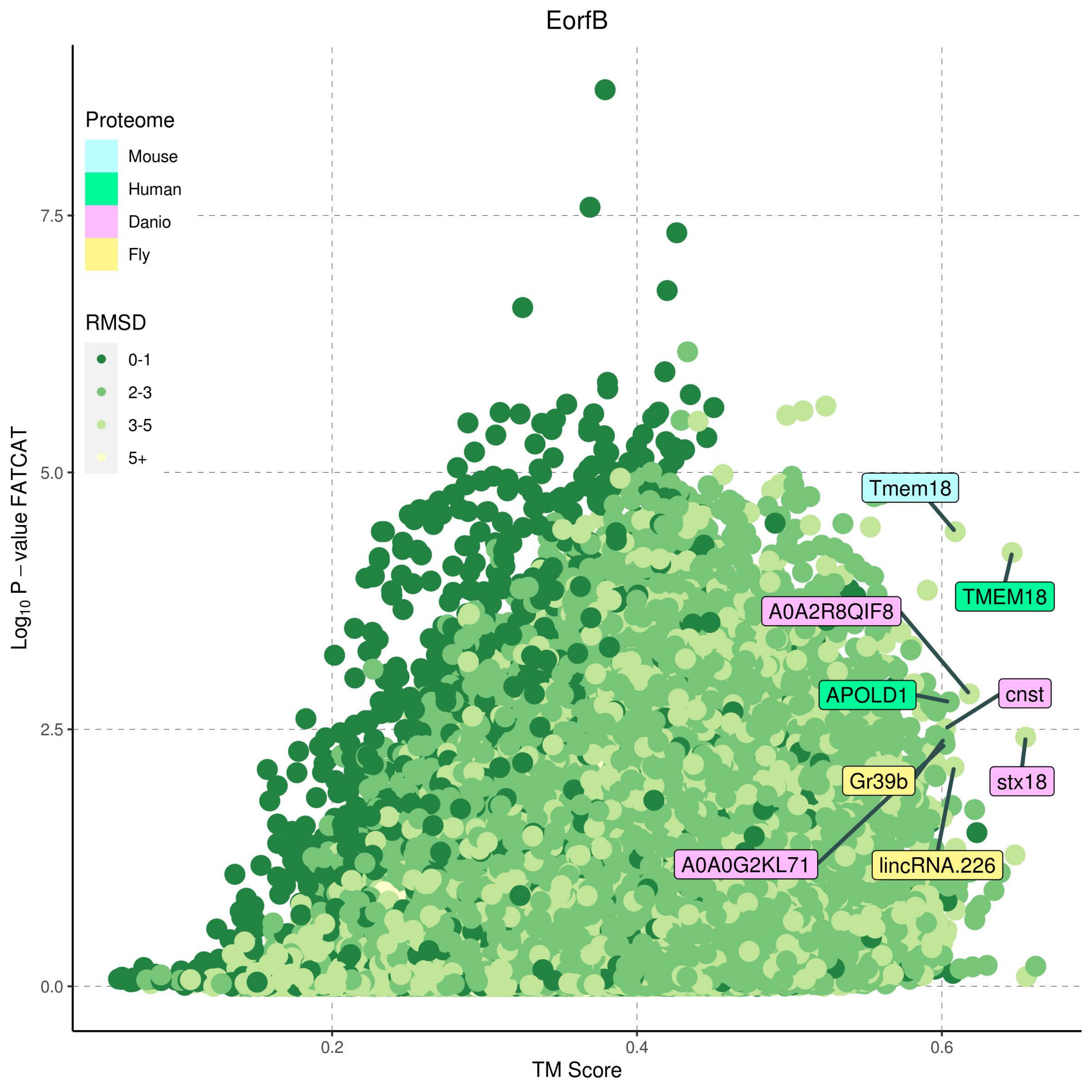
EorfB
Proteome
Mouse
Human
Danio
Fly
RMSD
0-1
2-3
3-5
5+
Tmem18
TMEM18
A0A2R8QIF8
APOLD1
cnst
Gr39b
stx18
A0A0G2KL71
lincRNA.226
7.5
5.0
2.5
0.0
0.2
0.4
0.6
TM Score
Log10 P – value FATCAT

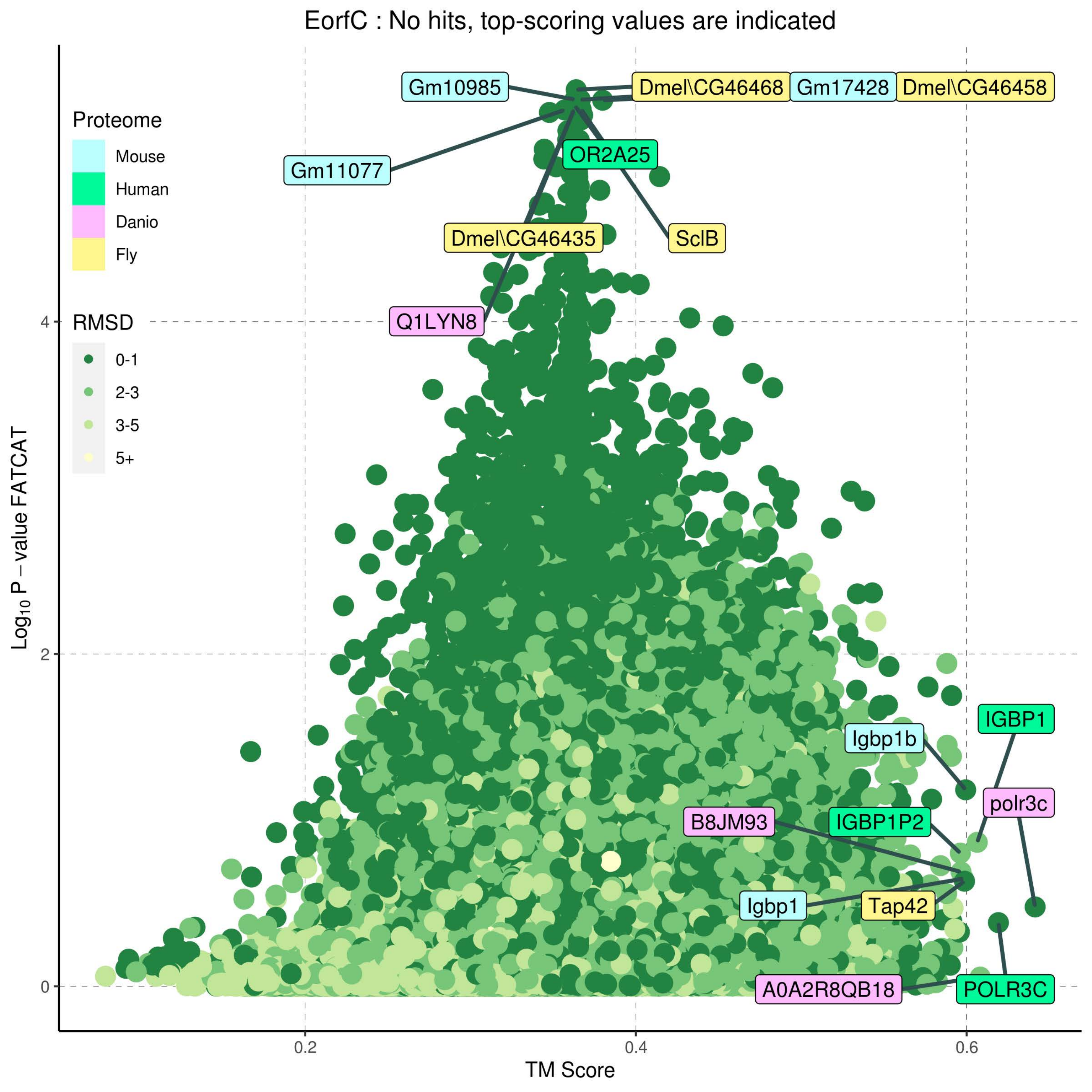
EorfC : No hits, top-scoring values are indicated
Proteome
Mouse
Human
Danio
Fly
RMSD
0-1
2-3
3-5
5+
Gm10985
Dmel\CG46468
Gm17428
Dmel\CG46458
OR2A25
Gm11077
Dmel\CG46435
SclB
Q1LYN8
IGBP1
Igbp1b
polr3c
B8JM93
IGBP1P2
Igbp1
Tap42
A0A2R8QB18
POLR3C
Log10 P – value FATCAT
TM Score
0.2
0.4
0.6
0
2
4

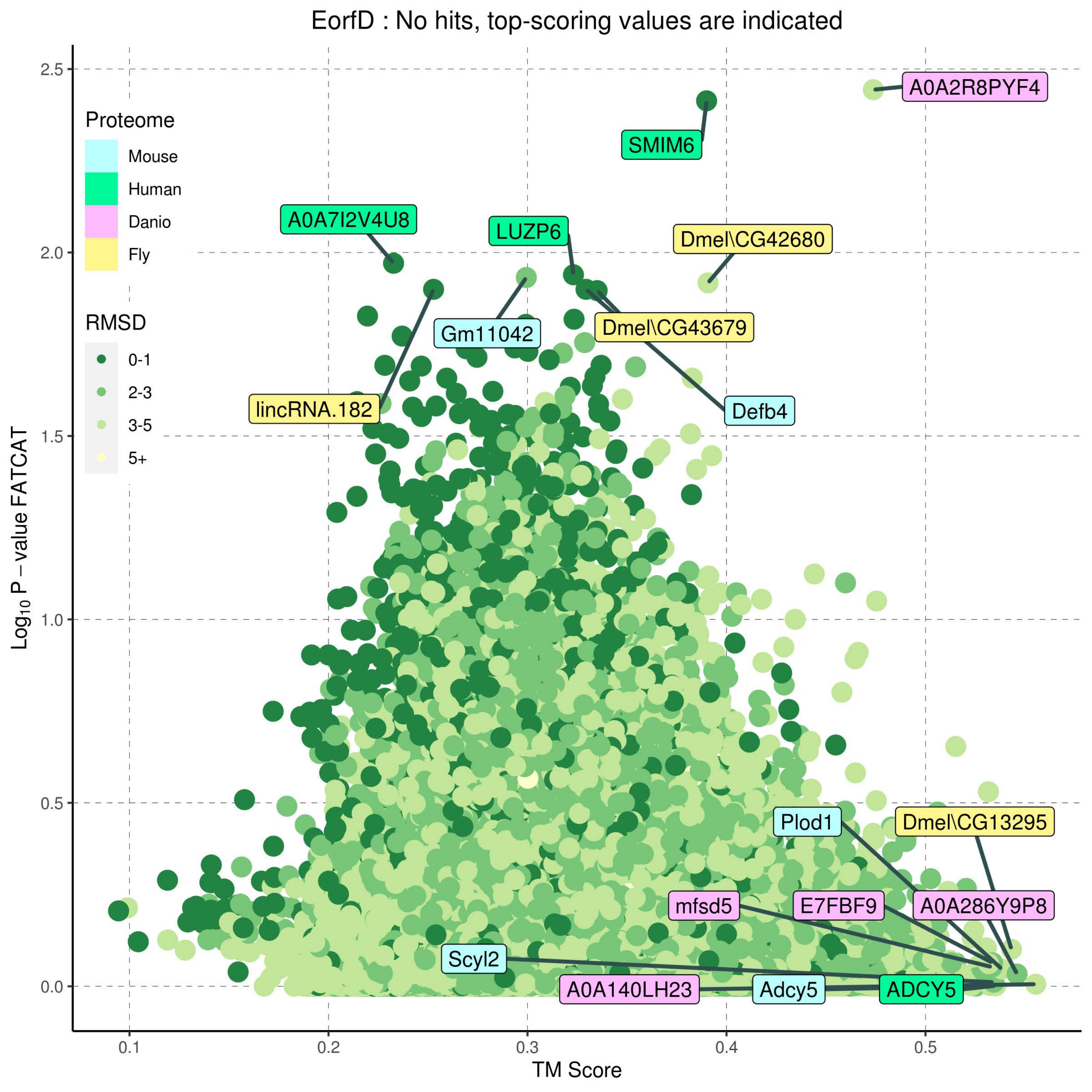

EorfD : No hits, top-scoring values are indicated
Proteome
Mouse
Human
Danio
Fly
RMSD
0-1
2-3
3-5
5+
A0A2R8PYF4
SMIM6
A0A7I2V4U8
LUZP6
Dmel\CG42680
Gm11042
Dmel\CG43679
lincRNA.182
Defb4
Plod1
Dmel\CG13295
mfsd5
E7FBF9
A0A286Y9P8
Scyl2
A0A140LH23
Adcy5
ADCY5
2.5
2.0
1.5
1.0
0.5
0.0
0.1
0.2
0.3
0.4
0.5
TM Score
Log10 P – value FATCAT

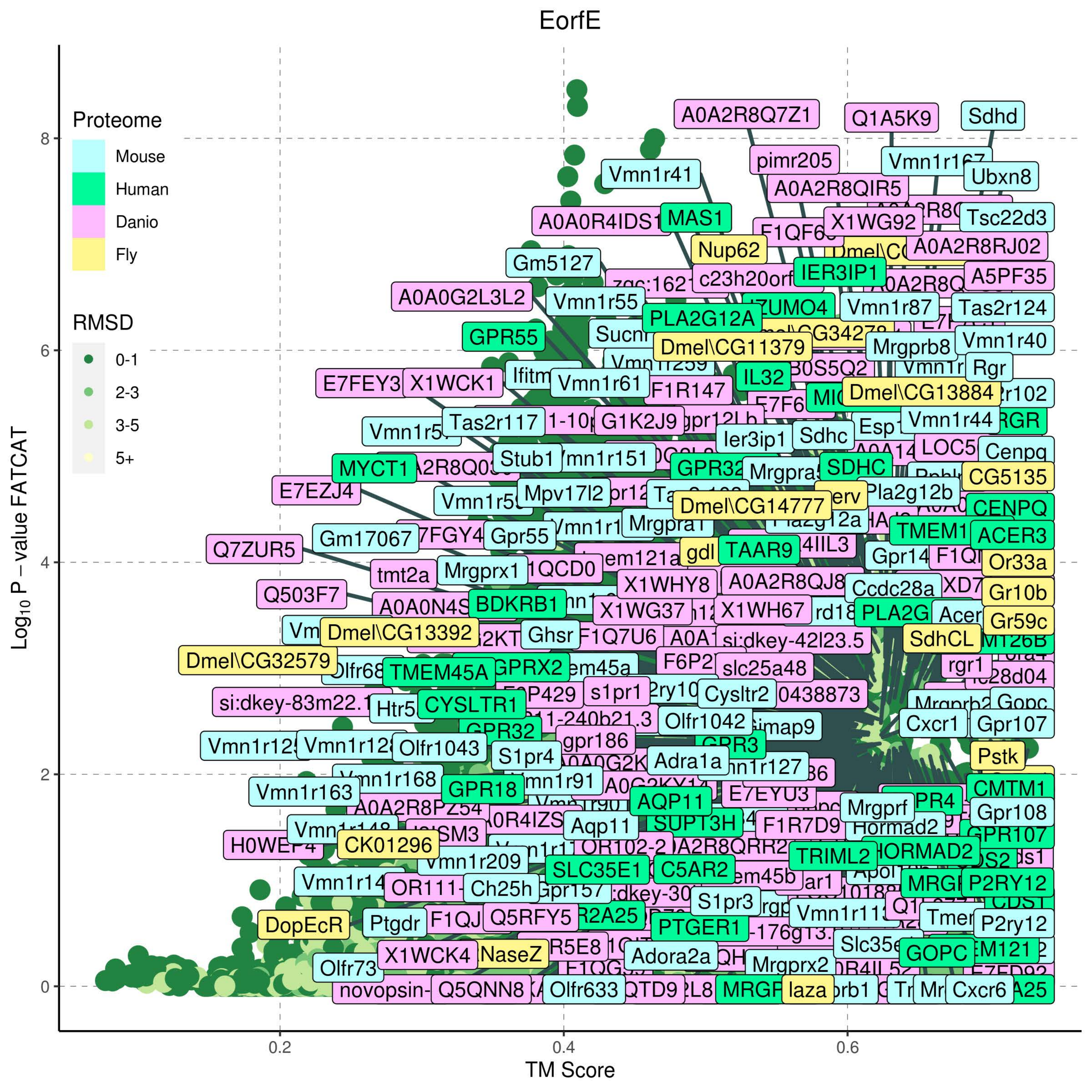
EorfE
Proteome
Mouse
Human
Danio
Fly
RMSD
0-1
2-3
3-5
5+
Log10 P − value FATCAT
TM Score
0
2
4
6
8
0.2
0.4
0.6
A0A2R8Q7Z1
Q1A5K9
Sdhd
pimr205
Vmn1r41
Ubxn8
A0A2R8QIR5
MAS1
A0A0R4IDS1
X1WG92
Tsc22d3
Nup62
A0A2R8RJ02
Gm5127
IER3IP1
A5PF35
A0A0G2L3L2
Vmn1r55
Vmn1r87
Tas2r124
PLA2G12A
GPR55
Dmel\CG11379
Mrgprb8
Vmn1r40
Rgr
E7FEY3
X1WCK1
Vmn1r61
IL32
F1R147
Dmel\CG13884
Tas2r117
G1K2J9
Vmn1r44
Ier3ip1
Sdhc
Stub1
MYCT1
SDHC
CG5135
E7EZJ4
Mpv17l2
Pla2g12b
Dmel\CG14777
CENPQ
ACER3
Gm17067
Gpr55
Q7ZUR5
TAAR9
Or33a
tmt2a
Mrgprx1
X1WHY8
Q503F7
Ccdc28a
Gr10b
BDKRB1
X1WG37
X1WH67
Gr59c
Dmel\CG13392
Ghsr
F1Q7U6
si:dkey-42l23.5
SdhCL
Dmel\CG32579
TMEM45A
GPRX2
slc25a48
rgr1
si:dkey-83m22.1
CYSLTR1
s1pr1
Cysltr2
Olfr1042
Cxcr1
Gpr107
Olfr1043
S1pr4
Adra1a
Pstk
Vmn1r168
Vmn1r163
GPR18
AQP11
E7EYU3
CMTM1
Mrgprf
Gpr108
Aqp11
SUPT3H
F1R7D9
Hormad2
CK01296
TRIML2
HORMAD2
Vmn1r209
SLC35E1
C5AR2
MRGPRD
P2RY12
Ch25h
S1pr3
DopEcR
Ptgdr
Q5RFY5
PTGER1
P2ry12
X1WCK4
NaseZ
Adora2a
Mrgprx2
GOPC
Olfr73
Q5QNN8
Olfr633
laza
Cxcr6

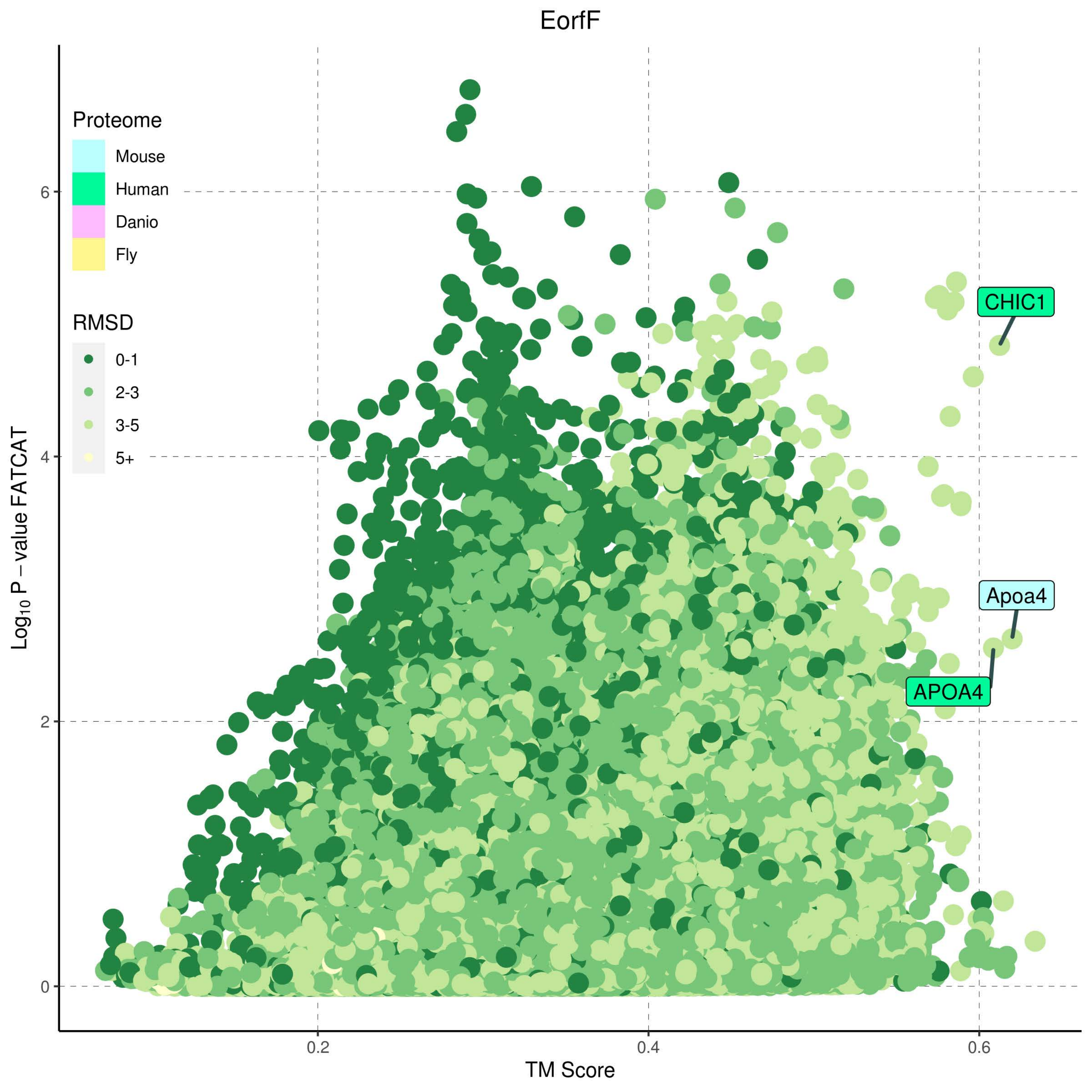
EorfF
Proteome
Mouse
Human
Danio
Fly
RMSD
0-1
2-3
3-5
5+
CHIC1
Apoa4
APOA4
Log10 P − value FATCAT
0
2
4
6
0.2
0.4
0.6
TM Score

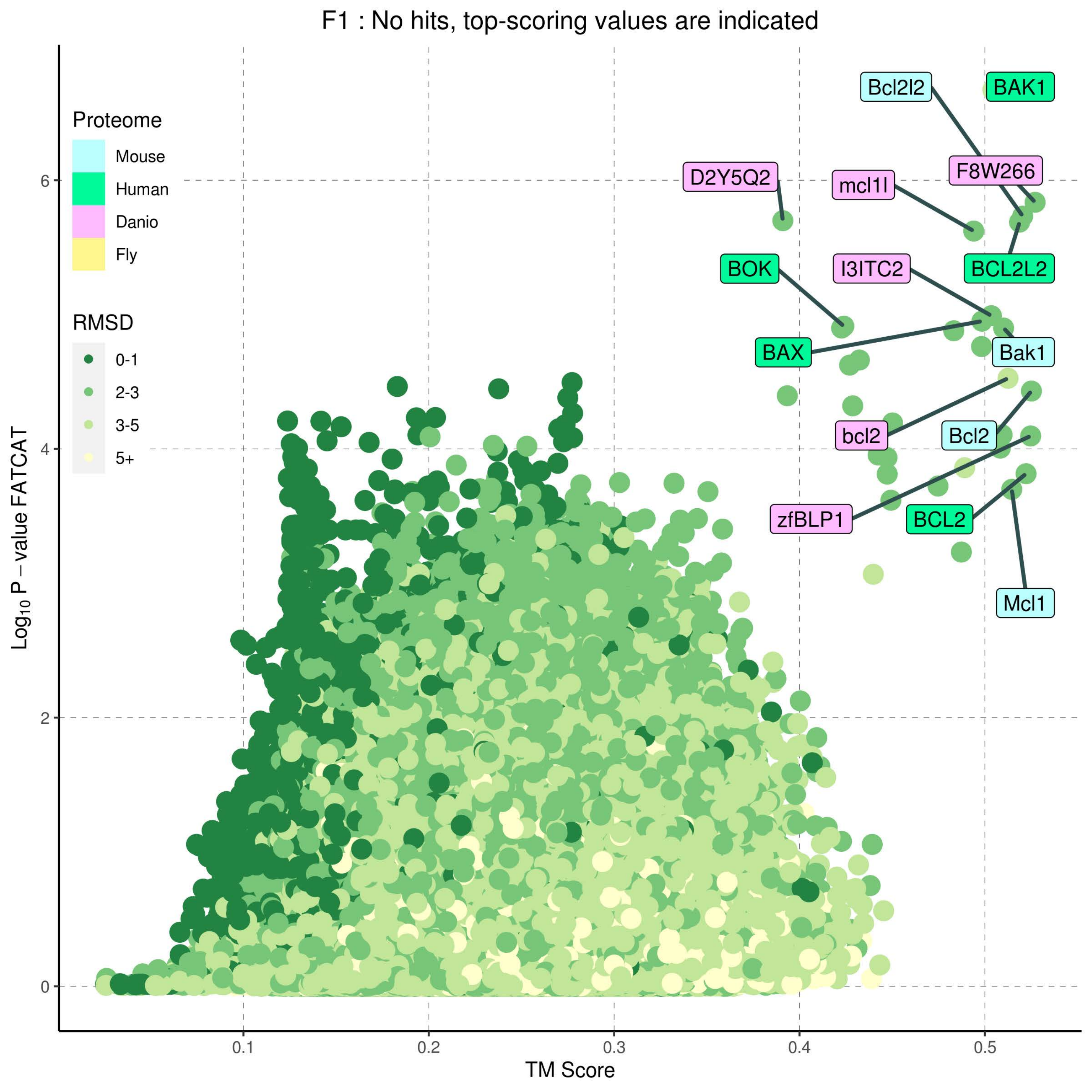

F1 : No hits, top-scoring values are indicated
Proteome
Mouse
Human
Danio
Fly
RMSD
0-1
2-3
3-5
5+
Bcl2l2
BAK1
D2Y5Q2
mcl1l
F8W266
BOK
I3ITC2
BCL2L2
BAX
Bak1
bcl2
Bcl2
zfBLP1
BCL2
Mcl1
Log_{10} P − value FATCAT
TM Score
0
2
4
6
0.1
0.2
0.3
0.4
0.5

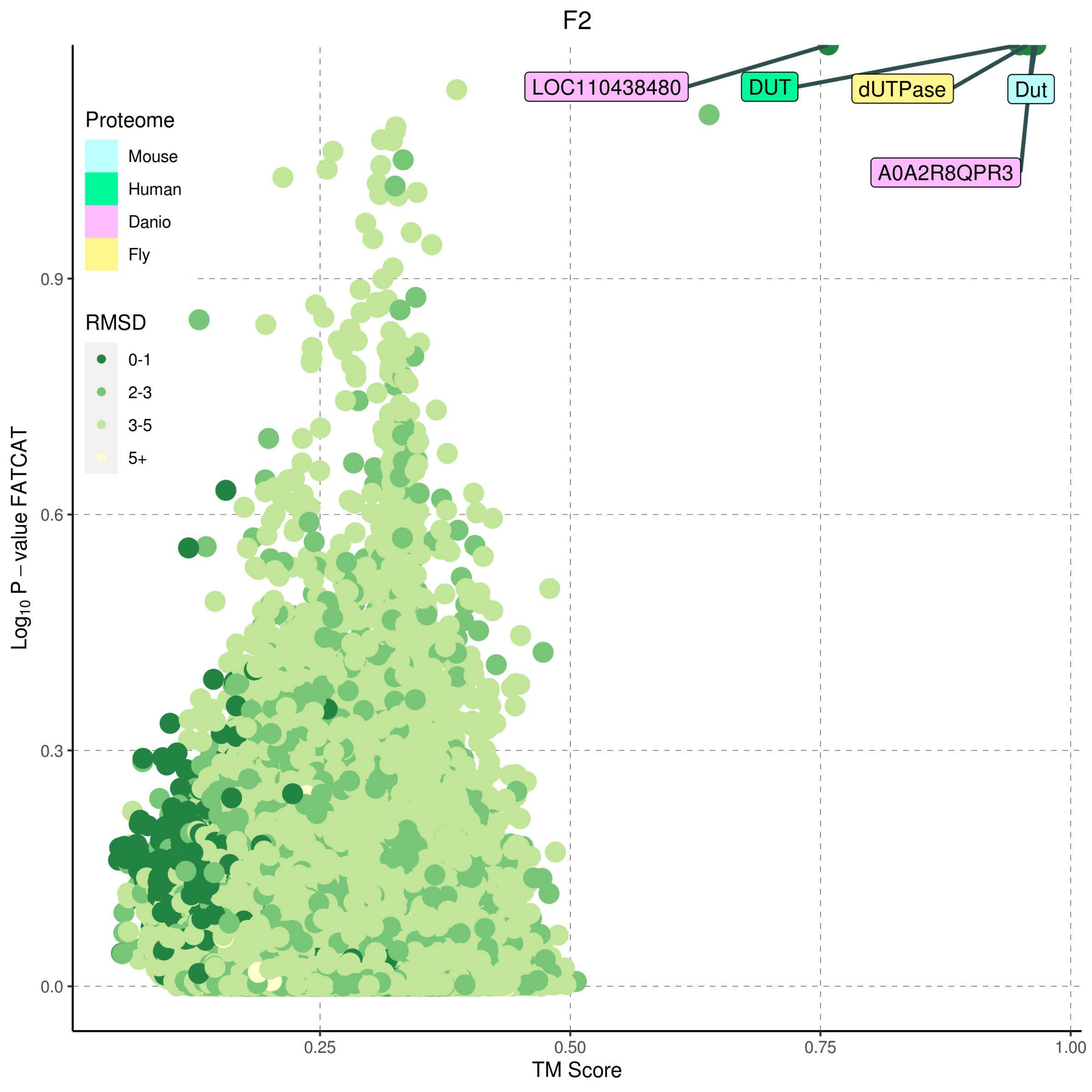
F2
LOC110438480
DUT
dUTPase
Dut
A0A2R8QPR3
Proteome
Mouse
Human
Danio
Fly
RMSD
0-1
2-3
3-5
5+
0.9
0.6
0.3
0.0
Log10 P − value FATCAT
0.25
0.50
0.75
1.00
TM Score

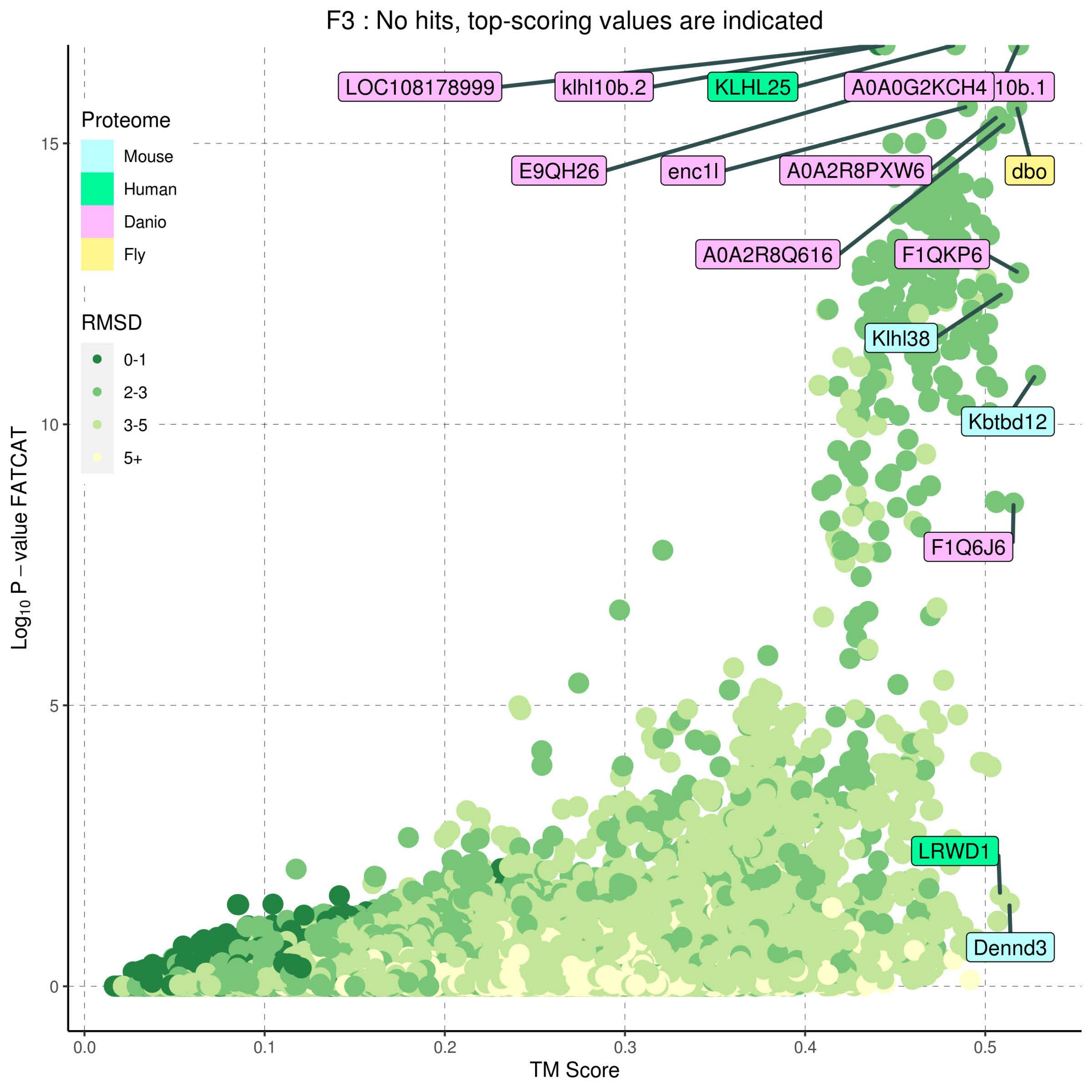
F3 : No hits, top-scoring values are indicated
Proteome
Mouse
Human
Danio
Fly
RMSD
0-1
2-3
3-5
5+
LOC108178999
klhl10b.2
KLHL25
A0A0G2KCH4
10b.1
E9QH26
enc1l
A0A2R8PXW6
dbo
A0A2R8Q616
F1QKP6
Klhl38
Kbtbd12
F1Q6J6
LRWD1
Dennd3
Log10 P – value FATCAT
TM Score
0
5
10
15
0.0
0.1
0.2
0.3
0.4
0.5

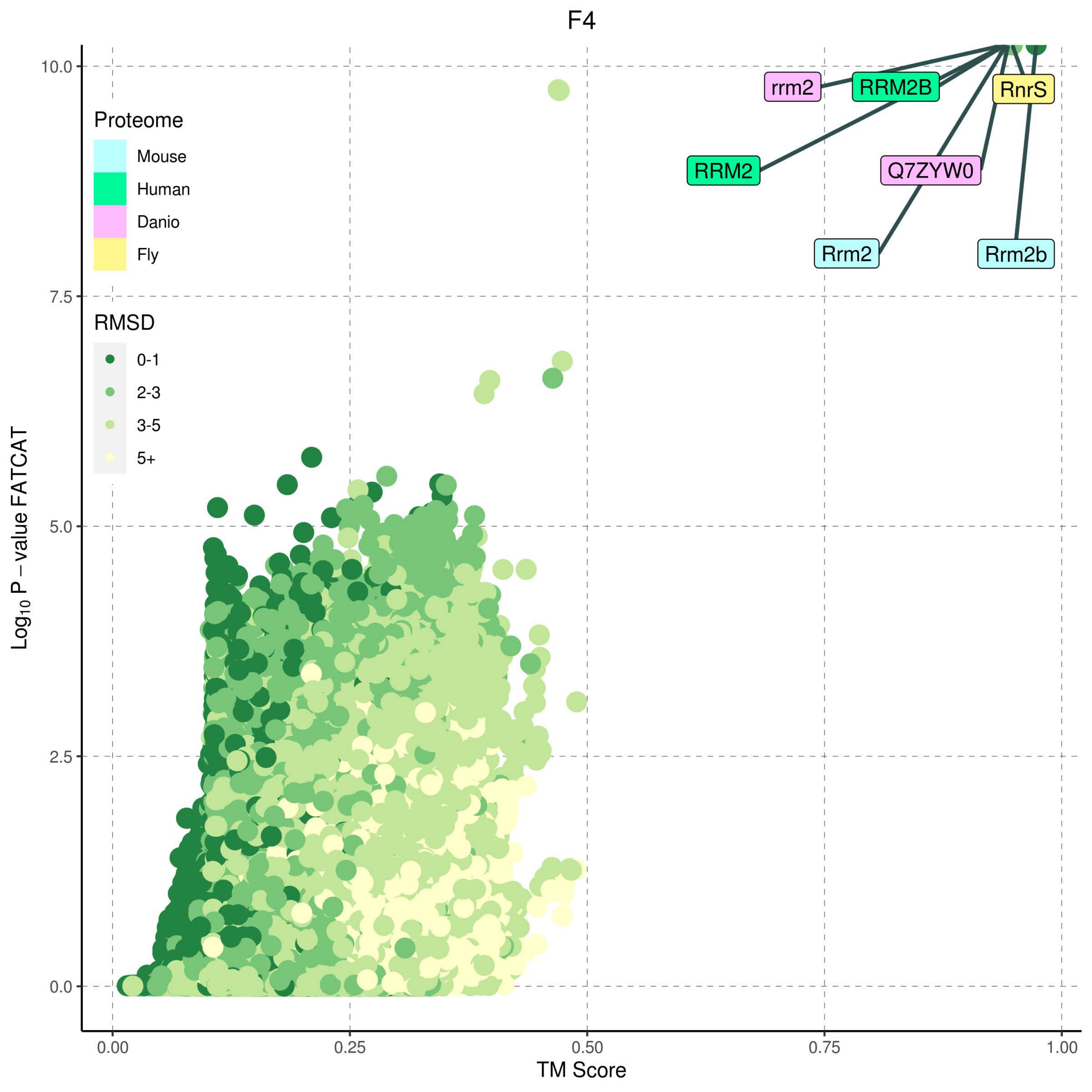
F4
Proteome
Mouse
Human
Danio
Fly
RMSD
0-1
2-3
3-5
5+
rrm2
RRM2B
RnrS
RRM2
Q7ZYW0
Rrm2
Rrm2b
Log10 P − value FATCAT
TM Score
10.0
7.5
5.0
2.5
0.0
0.00
0.25
0.50
0.75
1.00

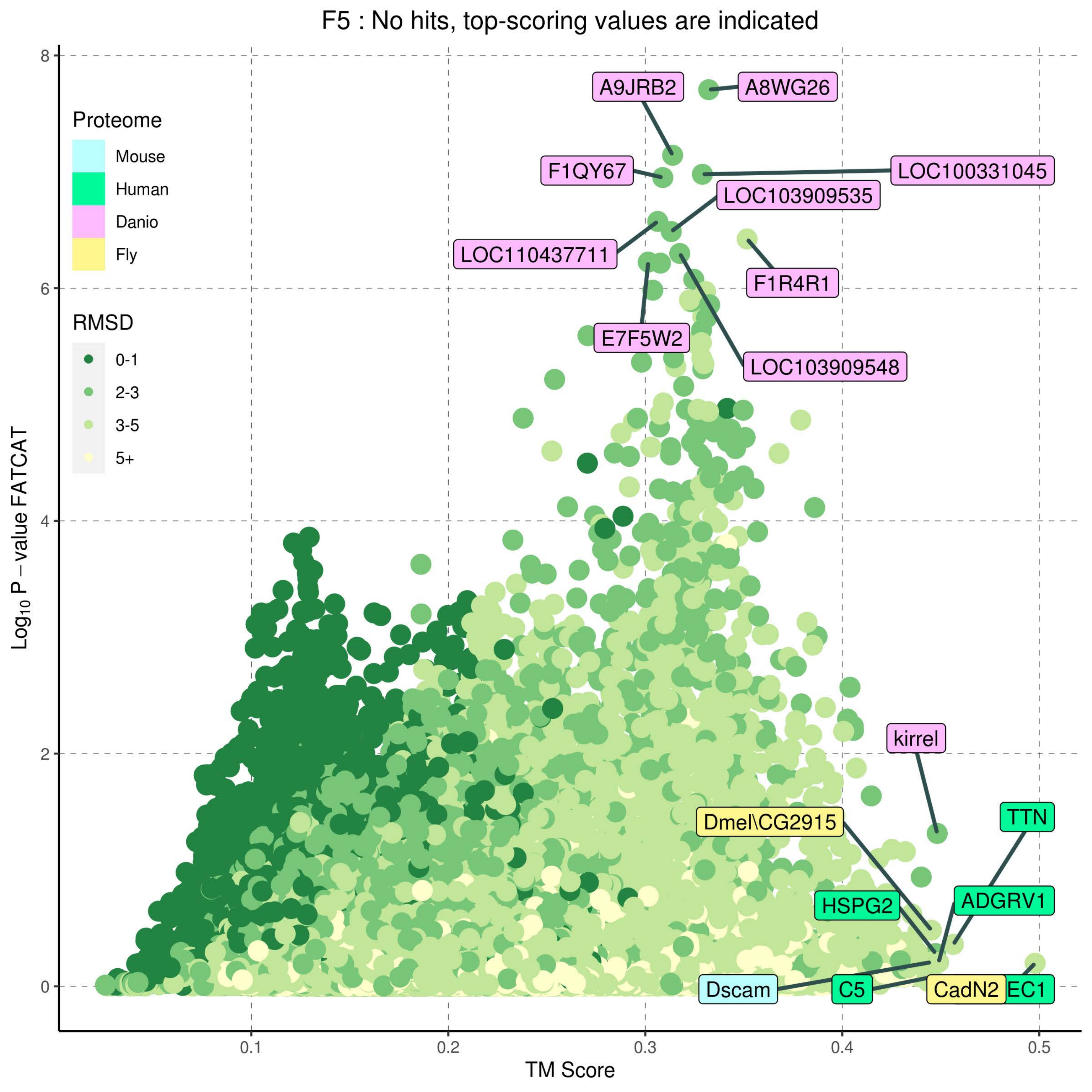

F5 : No hits, top-scoring values are indicated
Proteome
Mouse
Human
Danio
Fly
RMSD
0-1
2-3
3-5
5+
A9JRB2
A8WG26
F1QY67
LOC100331045
LOC103909535
LOC110437711
F1R4R1
E7F5W2
LOC103909548
kirrel
Dmel\CG2915
TTN
HSPG2
ADGRV1
Dscam
C5
CadN2
EC1
Log10 P – value FATCAT
TM Score
0
2
4
6
8
0.1
0.2
0.3
0.4
0.5

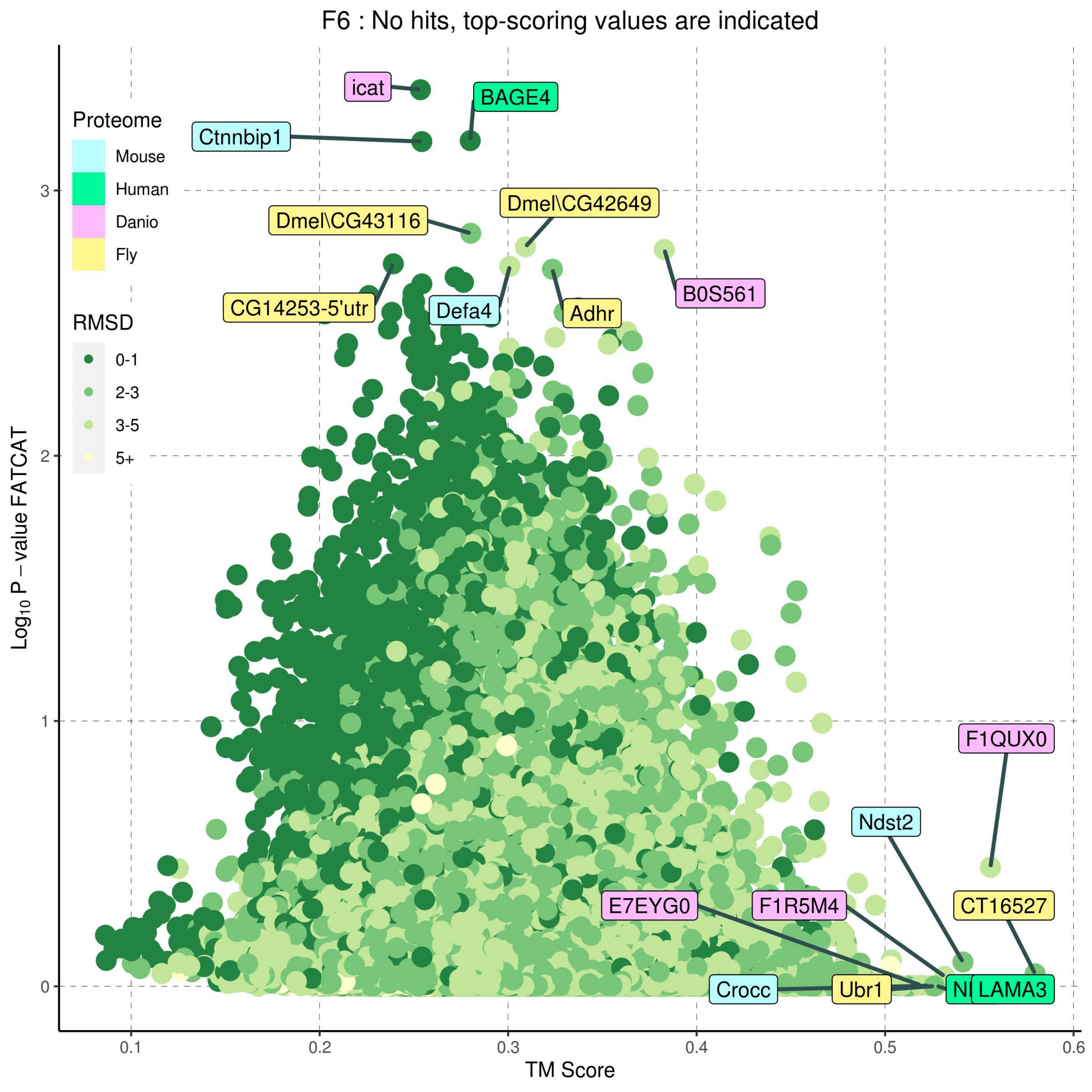

F6 : No hits, top-scoring values are indicated
Proteome
Mouse
Human
Danio
Fly
RMSD
0-1
2-3
3-5
5+
icat
BAGE4
Ctnnbip1
Dmel\CG43116
Dmel\CG42649
CG14253-5'utr
Defa4
Adhr
B0S561
F1QUX0
Ndst2
E7EYG0
F1R5M4
CT16527
Crocc
Ubr1
LAMA3
Log10 P – value FATCAT
TM Score
0
1
2
3
0.1
0.2
0.3
0.4
0.5
0.6

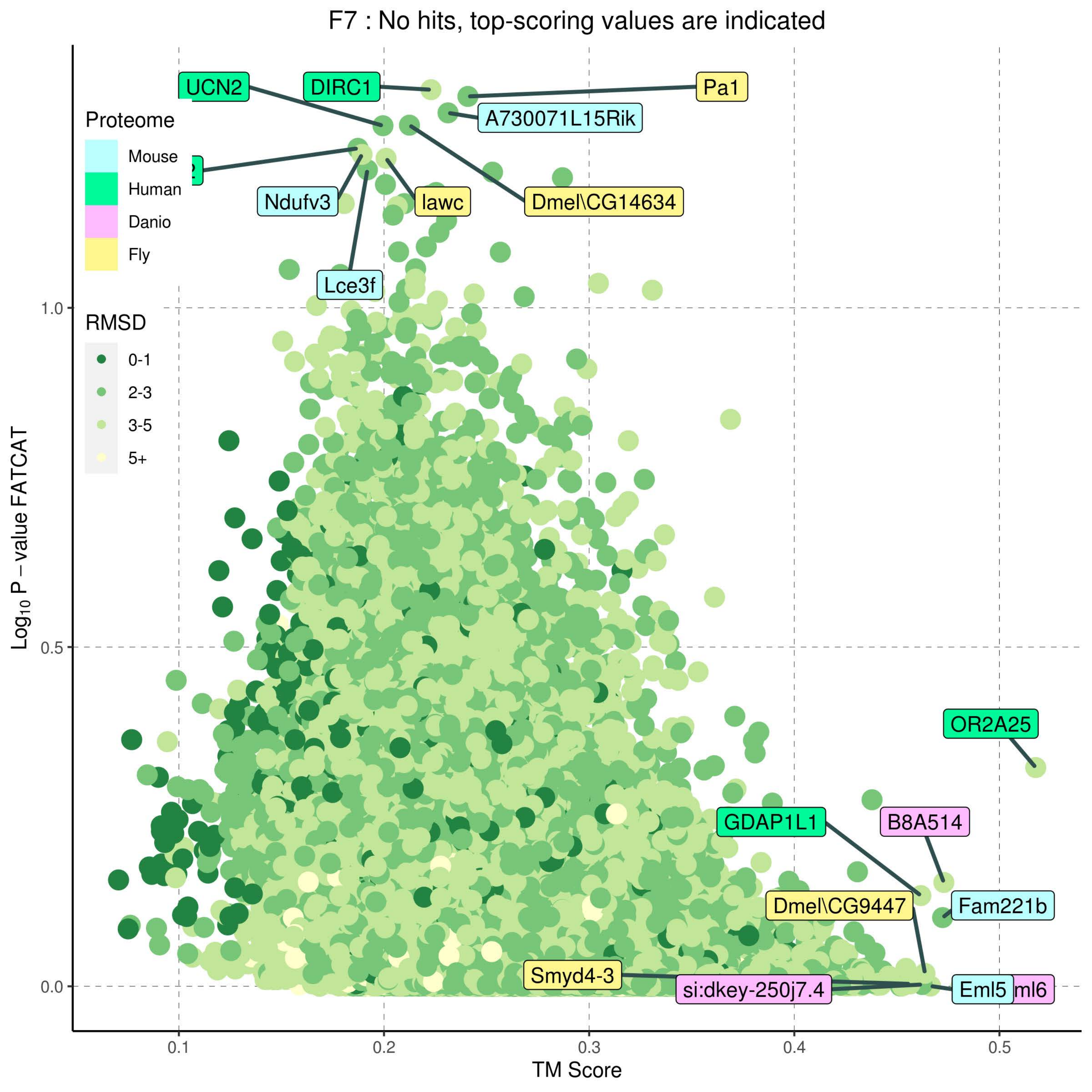
F7 : No hits, top-scoring values are indicated
Proteome
Mouse
Human
Danio
Fly
RMSD
0-1
2-3
3-5
5+
UCN2
DIRC1
Pa1
A730071L15Rik
Ndufv3
lawc
Dmel\CG14634
Lce3f
OR2A25
GDAP1L1
B8A514
Fam221b
Dmel\CG9447
Smyd4-3
si:dkey-250j7.4
Eml5
ml6
1.0
0.5
0.0
0.1
0.2
0.3
0.4
0.5
Log_{10} P − value FATCAT
TM Score

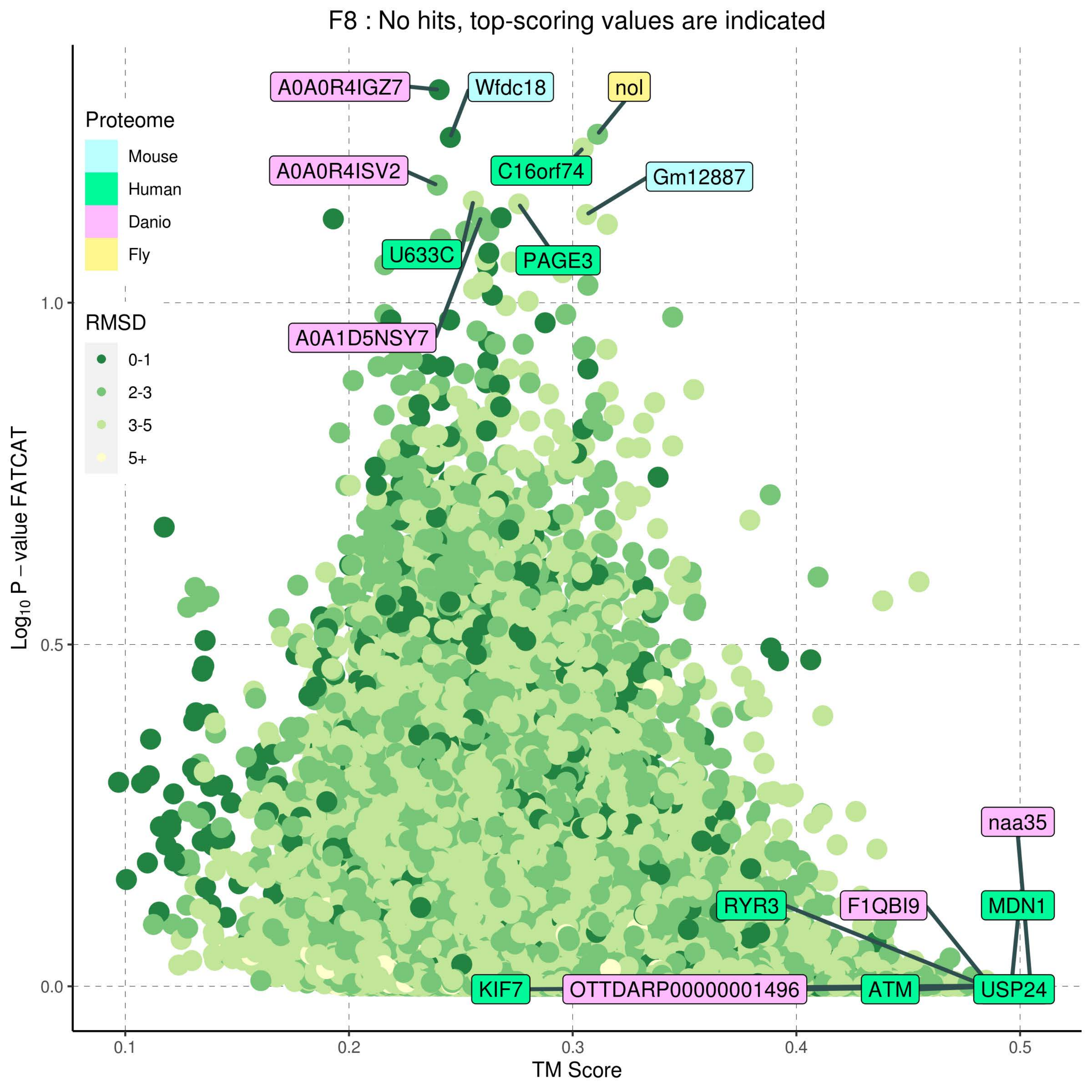

F8 : No hits, top-scoring values are indicated
Proteome
Mouse
Human
Danio
Fly
RMSD
0-1
2-3
3-5
5+
A0A0R4IGZ7
Wfdc18
nol
A0A0R4ISV2
C16orf74
Gm12887
U633C
PAGE3
A0A1D5NSY7
naa35
RYR3
F1QBI9
MDN1
KIF7
OTTDARP00000001496
ATM
USP24
1.0
0.5
0.0
0.1
0.2
0.3
0.4
0.5
TM Score
Log10 P – value FATCAT

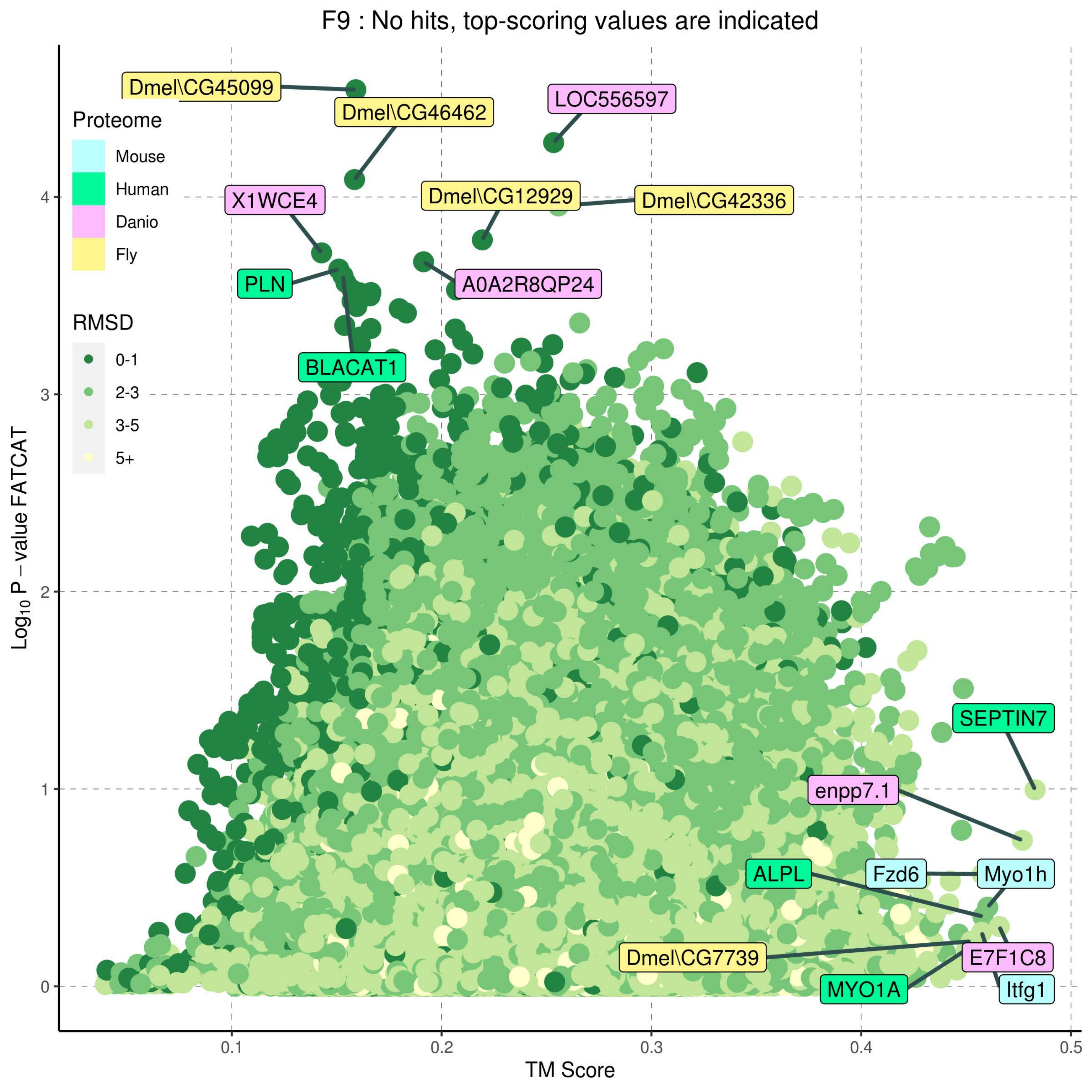
F9 : No hits, top-scoring values are indicated
Proteome
Mouse
Human
Danio
Fly
RMSD
0-1
2-3
3-5
5+
Dmel\CG45099
Dmel\CG46462
LOC556597
X1WCE4
Dmel\CG12929
Dmel\CG42336
PLN
A0A2R8QP24
BLACAT1
SEPTIN7
enpp7.1
ALPL
Fzd6
Myo1h
Dmel\CG7739
E7F1C8
MYO1A
Itfg1
Log10 P – value FATCAT
TM Score
0
1
2
3
4
0.1
0.2
0.3
0.4
0.5

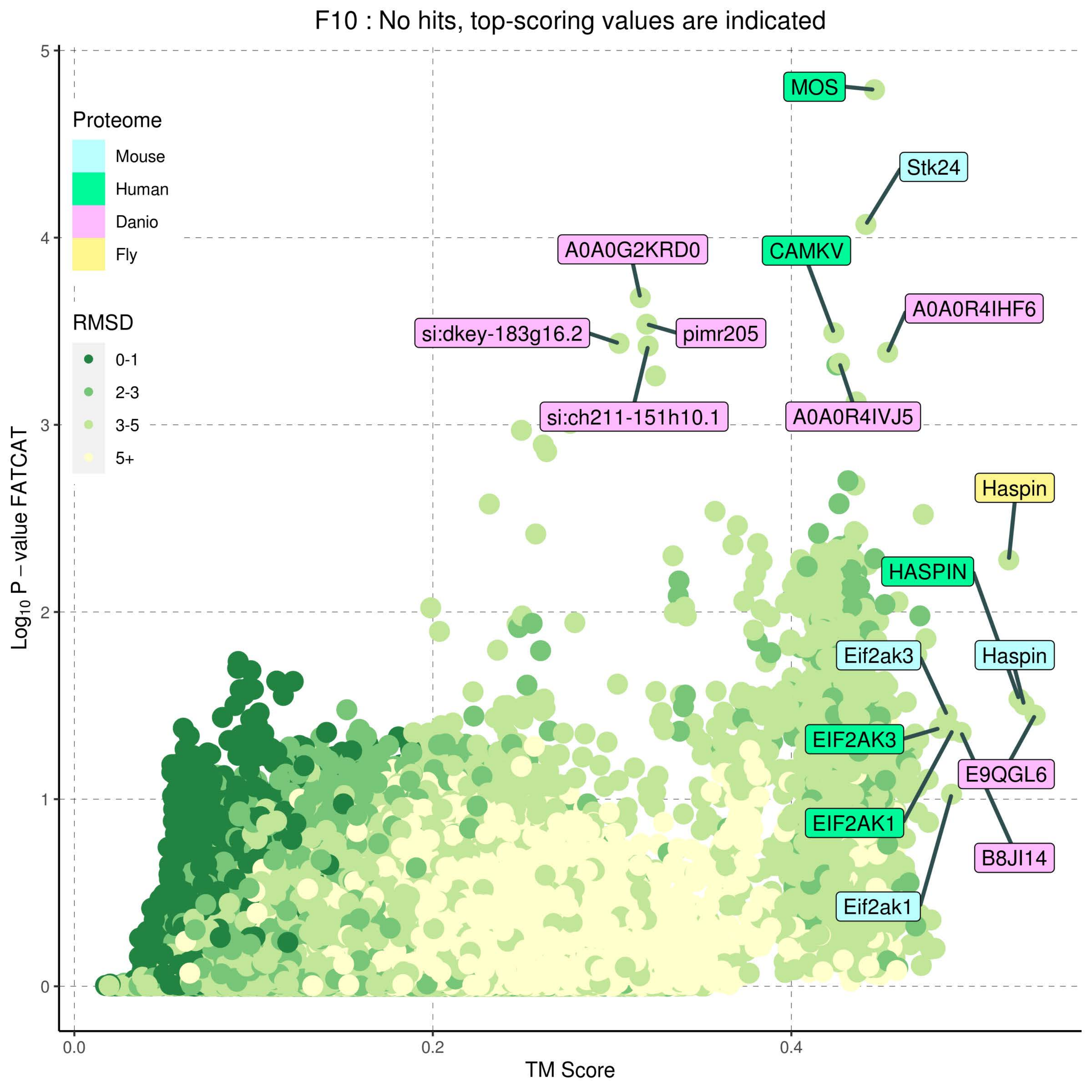

F10 : No hits, top-scoring values are indicated
Proteome
Mouse
Human
Danio
Fly
RMSD
0-1
2-3
3-5
5+
MOS
Stk24
A0A0G2KRD0
CAMKV
A0A0R4IHF6
si:dkey-183g16.2
pimr205
si:ch211-151h10.1
A0A0R4IVJ5
Haspin
HASPIN
Eif2ak3
Haspin
EIF2AK3
E9QGL6
EIF2AK1
B8JI14
Eif2ak1
Log10 P – value FATCAT
TM Score
0
1
2
3
4
5
0.0
0.2
0.4

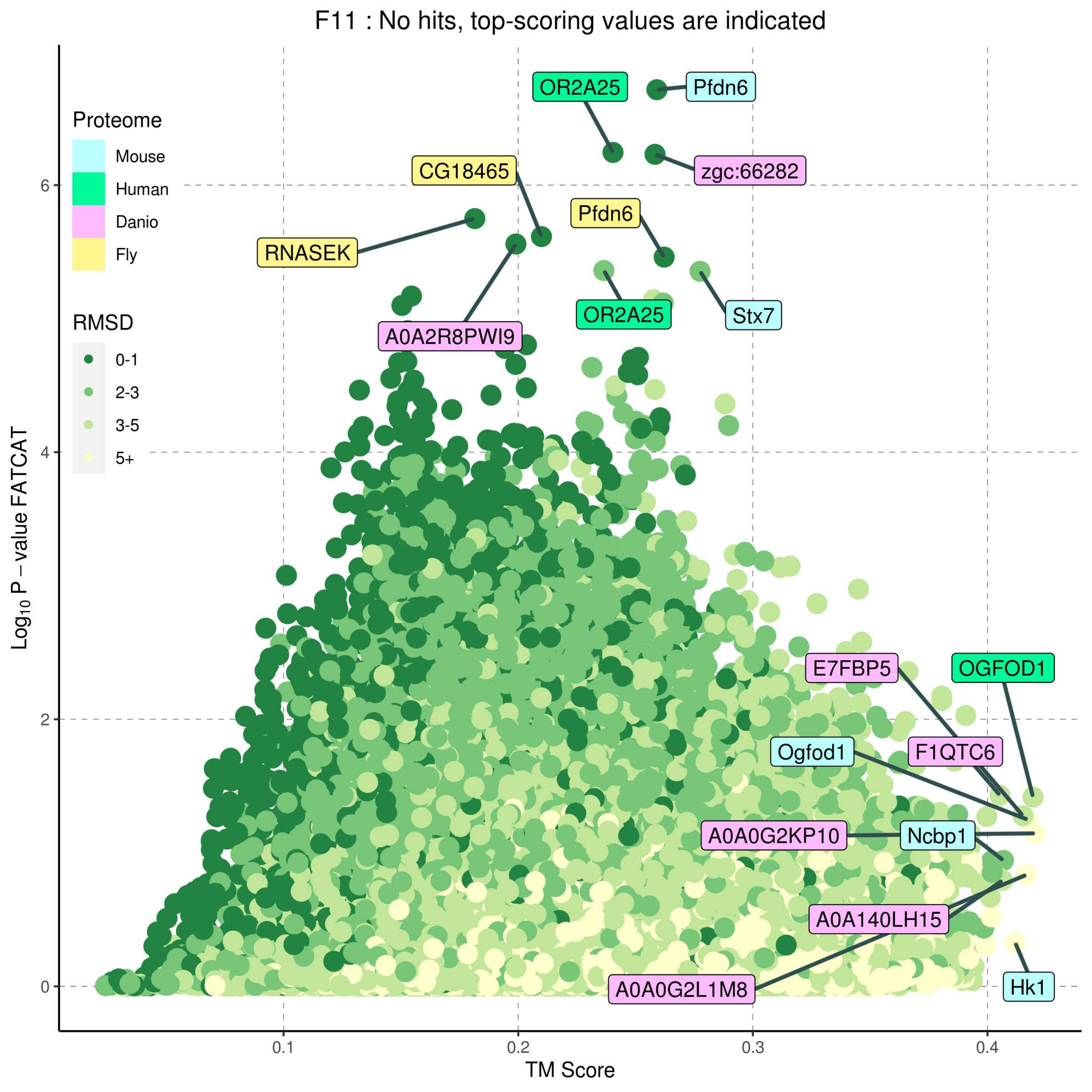

F11 : No hits, top-scoring values are indicated
Proteome
Mouse
Human
Danio
Fly
RMSD
0-1
2-3
3-5
5+
Log10 P – value FATCAT
TM Score
0.1
0.2
0.3
0.4
0
2
4
6
OR2A25
Pfdn6
zgc:66282
CG18465
Pfdn6
RNASEK
OR2A25
Stx7
A0A2R8PWI9
E7FBP5
OGFOD1
Ogfod1
F1QTC6
A0A0G2KP10
Ncbp1
A0A140LH15
A0A0G2L1M8
Hk1

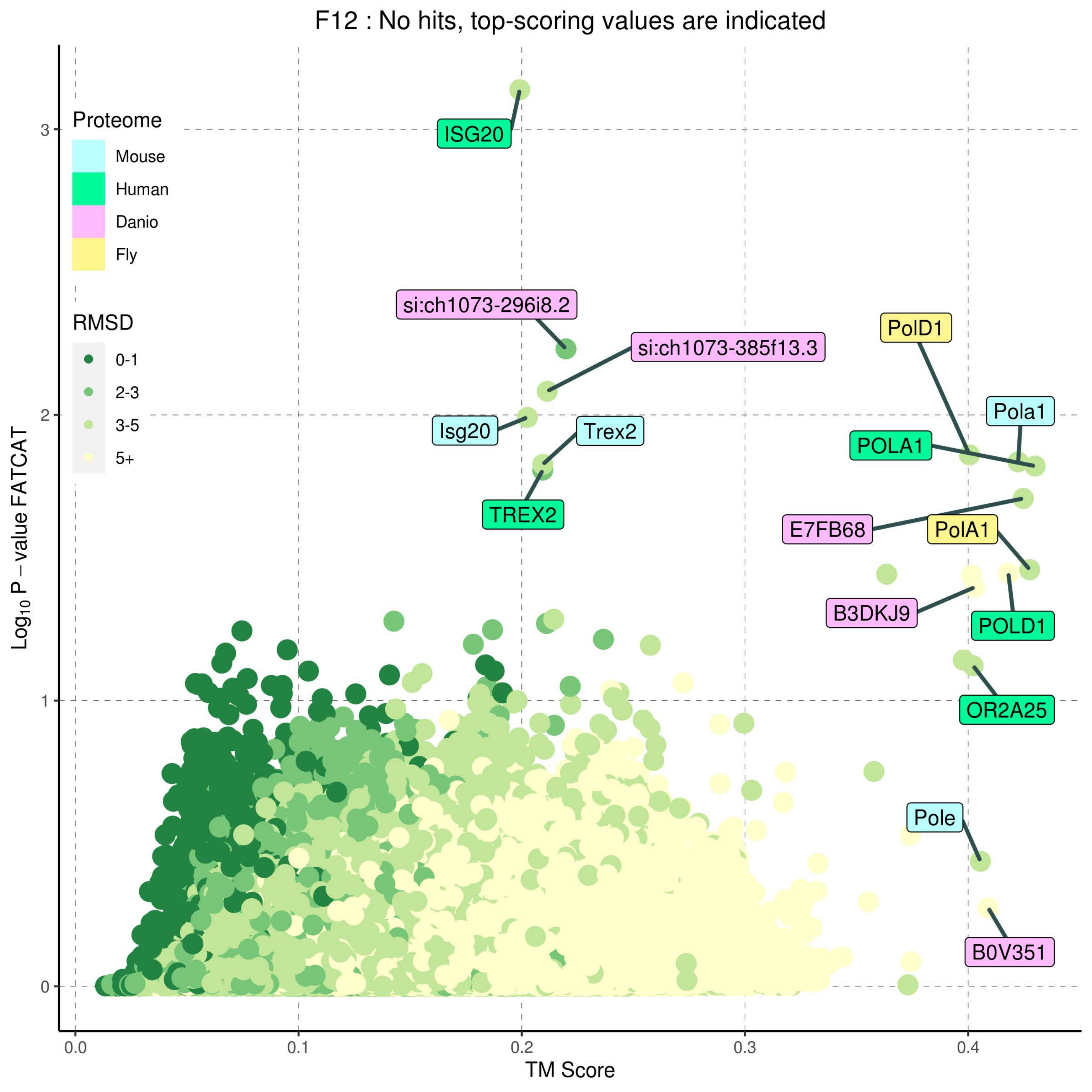

F12 : No hits, top-scoring values are indicated
Proteome
Mouse
Human
Danio
Fly
RMSD
0-1
2-3
3-5
5+
ISG20
si:ch1073-296i8.2
si:ch1073-385f13.3
Isg20
Trex2
TREX2
PolD1
Pola1
POLA1
E7FB68
PolA1
B3DKJ9
POLD1
OR2A25
Pole
B0V351
Log_{10} P − value FATCAT
TM Score
0
1
2
3
0.0
0.1
0.2
0.3
0.4

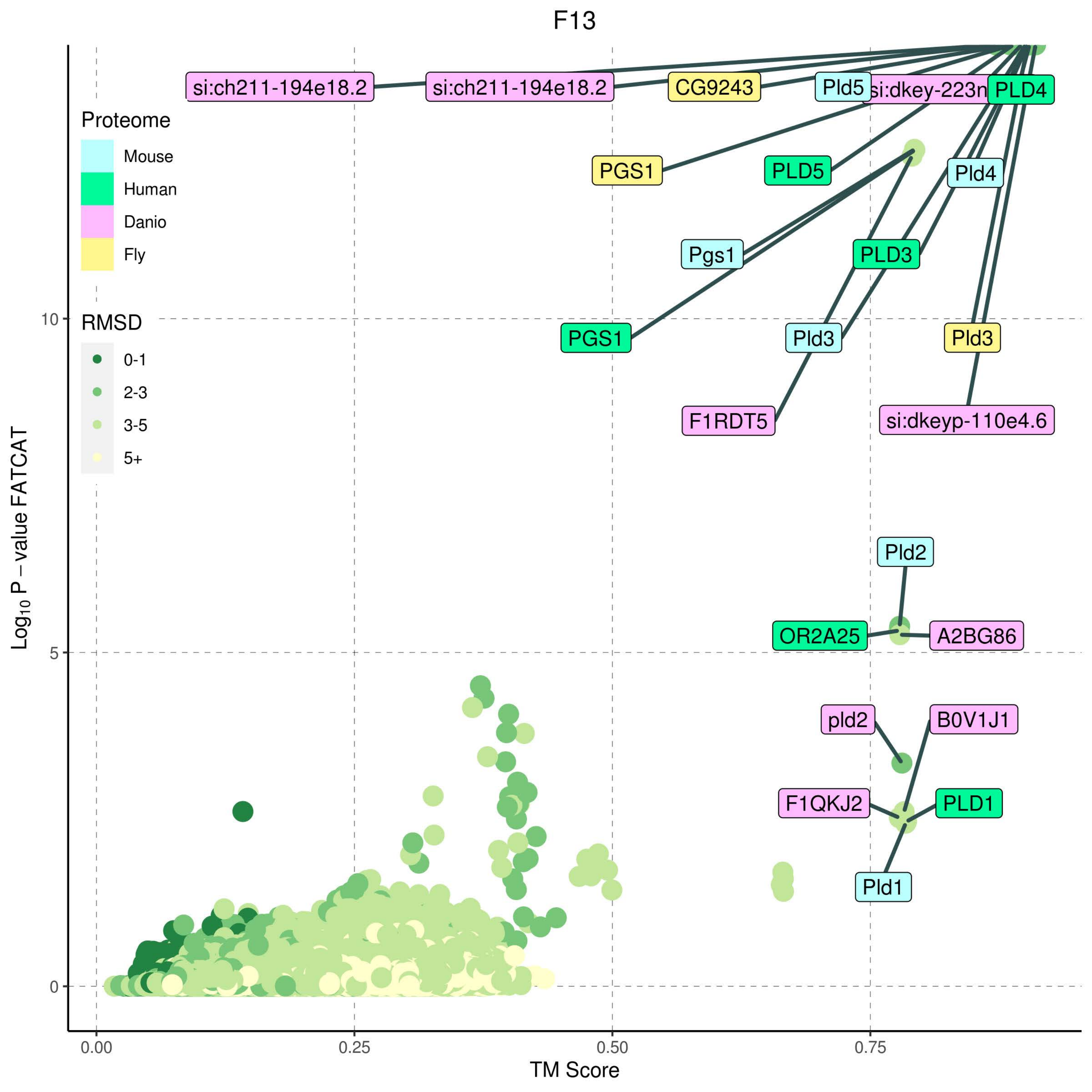
F13
Proteome
Mouse
Human
Danio
Fly
RMSD
0-1
2-3
3-5
5+
si:ch211-194e18.2
si:ch211-194e18.2
CG9243
Pld5
si:dkey-223n
PLD4
PGS1
PLD5
Pld4
Pgs1
PLD3
PGS1
Pld3
Pld3
F1RDT5
si:dkeyp-110e4.6
Pld2
OR2A25
A2BG86
pld2
B0V1J1
F1QKJ2
PLD1
Pld1
Log10 P − value FATCAT
TM Score
10
5
0
0.00
0.25
0.50
0.75

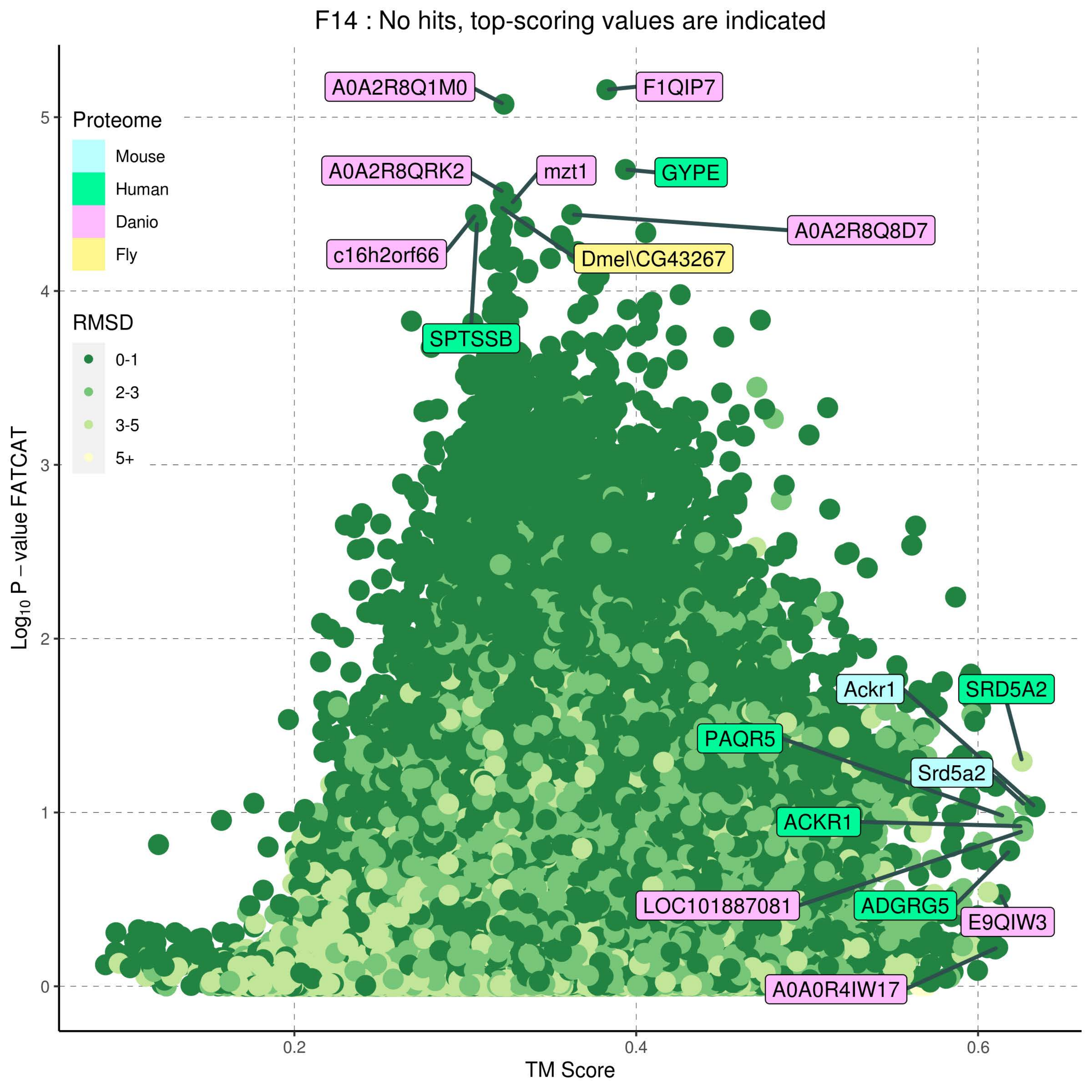

F14 : No hits, top-scoring values are indicated
Proteome
Mouse
Human
Danio
Fly
RMSD
0-1
2-3
3-5
5+
A0A2R8Q1M0
F1QIP7
A0A2R8QRK2
mzt1
GYPE
A0A2R8Q8D7
c16h2orf66
Dmel\CG43267
SPTSSB
Ackr1
SRD5A2
PAQR5
Srd5a2
ACKR1
LOC101887081
ADGRG5
E9QIW3
A0A0R4IW17
Log10 P − value FATCAT
TM Score
0
1
2
3
4
5
0.2
0.4
0.6

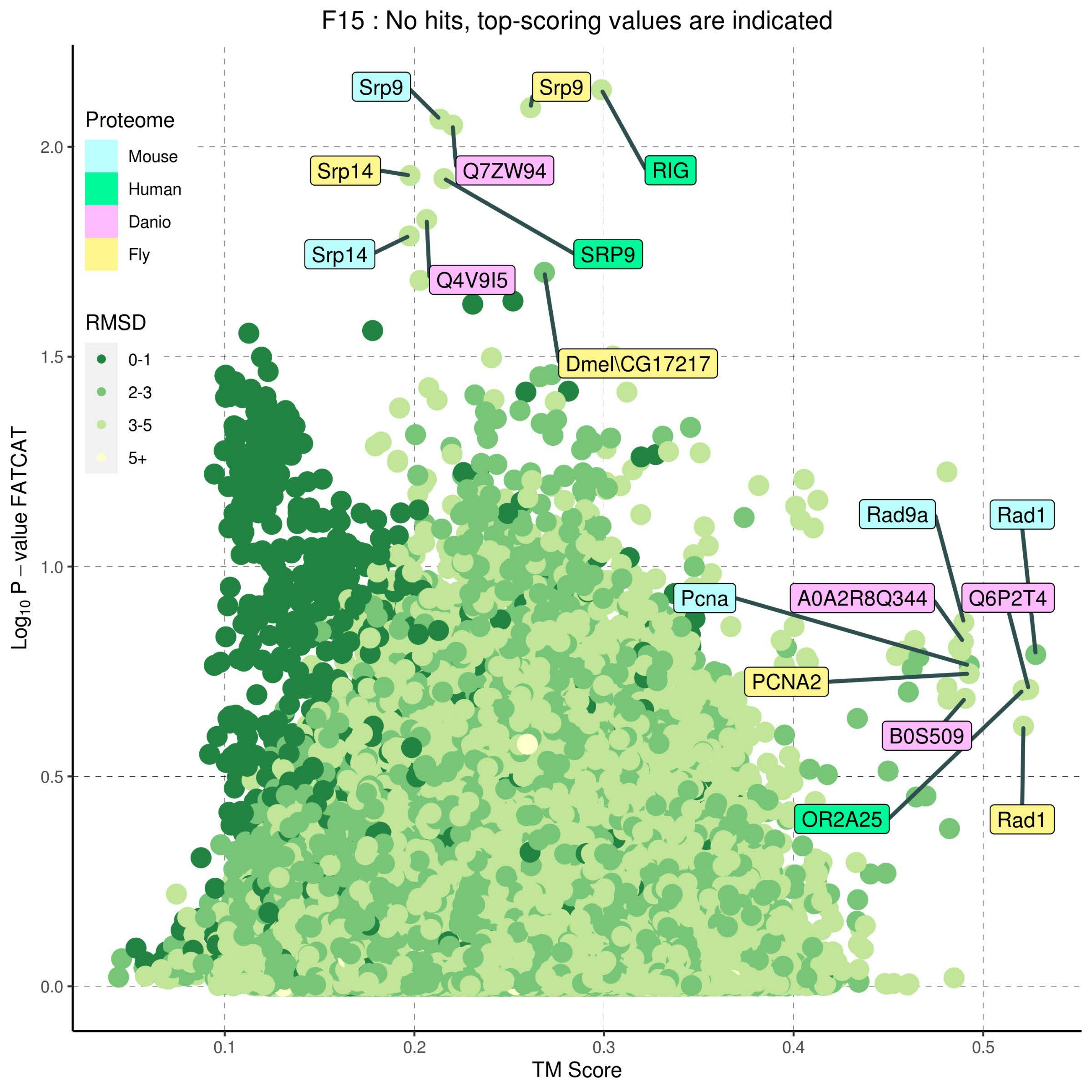
F15 : No hits, top-scoring values are indicated
Proteome
Mouse
Human
Danio
Fly
RMSD
0-1
2-3
3-5
5+
Srp9
Srp9
Q7ZW94
RIG
Srp14
SRP9
Srp14
Q4V9I5
Dmel\CG17217
Rad9a
Rad1
Pcna
A0A2R8Q344
Q6P2T4
PCNA2
B0S509
OR2A25
Rad1
$\mathrm{Log_{10}}$ P − value FATCAT
TM Score
0.0
0.5
1.0
1.5
2.0
0.1
0.2
0.3
0.4
0.5

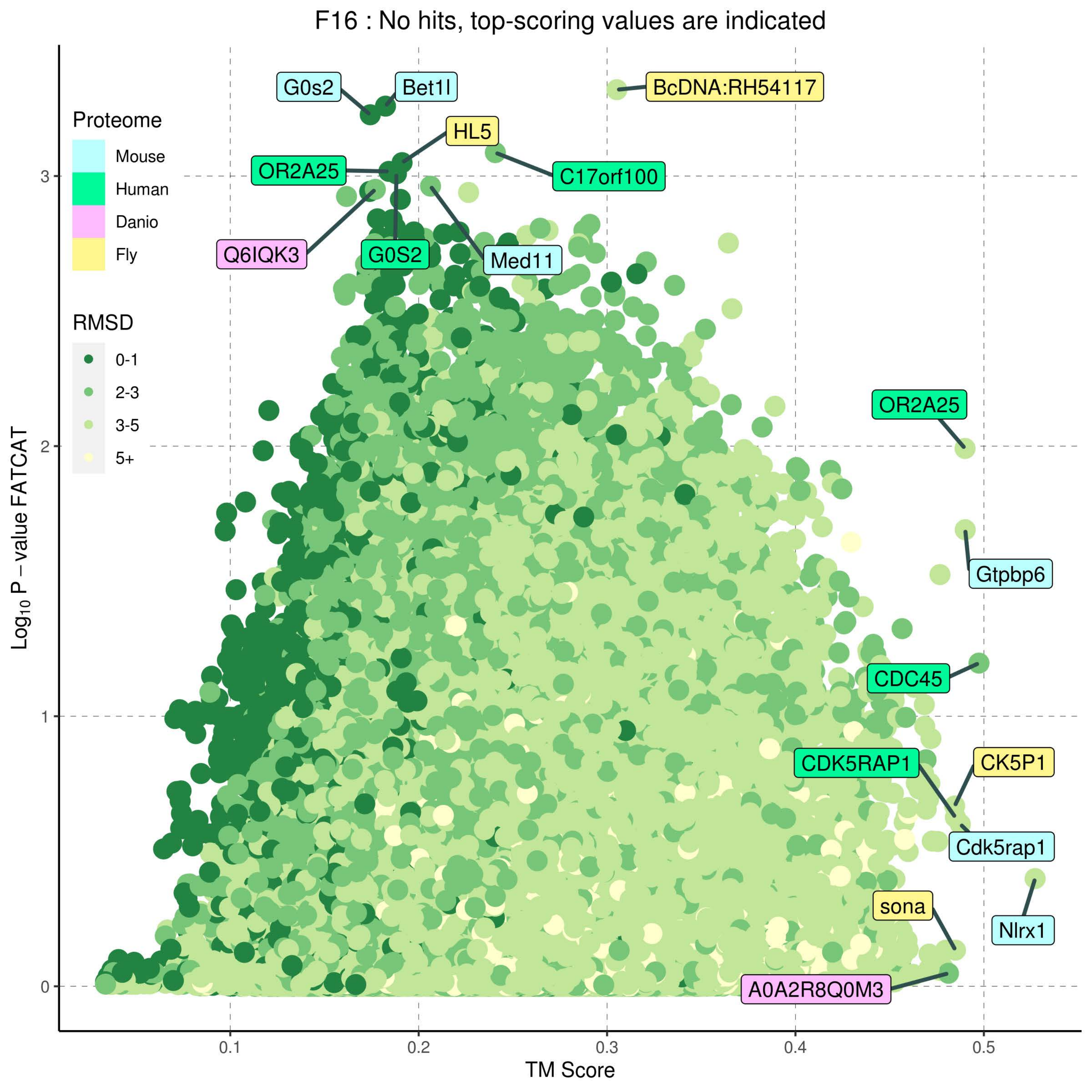
F16 : No hits, top-scoring values are indicated
Proteome
Mouse
Human
Danio
Fly
RMSD
0-1
2-3
3-5
5+
G0s2
Bet1l
BcDNA:RH54117
HL5
OR2A25
C17orf100
Q6IQK3
G0S2
Med11
OR2A25
Gtpbp6
CDC45
CDK5RAP1
CK5P1
Cdk5rap1
sona
Nlrx1
A0A2R8Q0M3
Log10 P – value FATCAT
TM Score
0
1
2
3
0.1
0.2
0.3
0.4
0.5

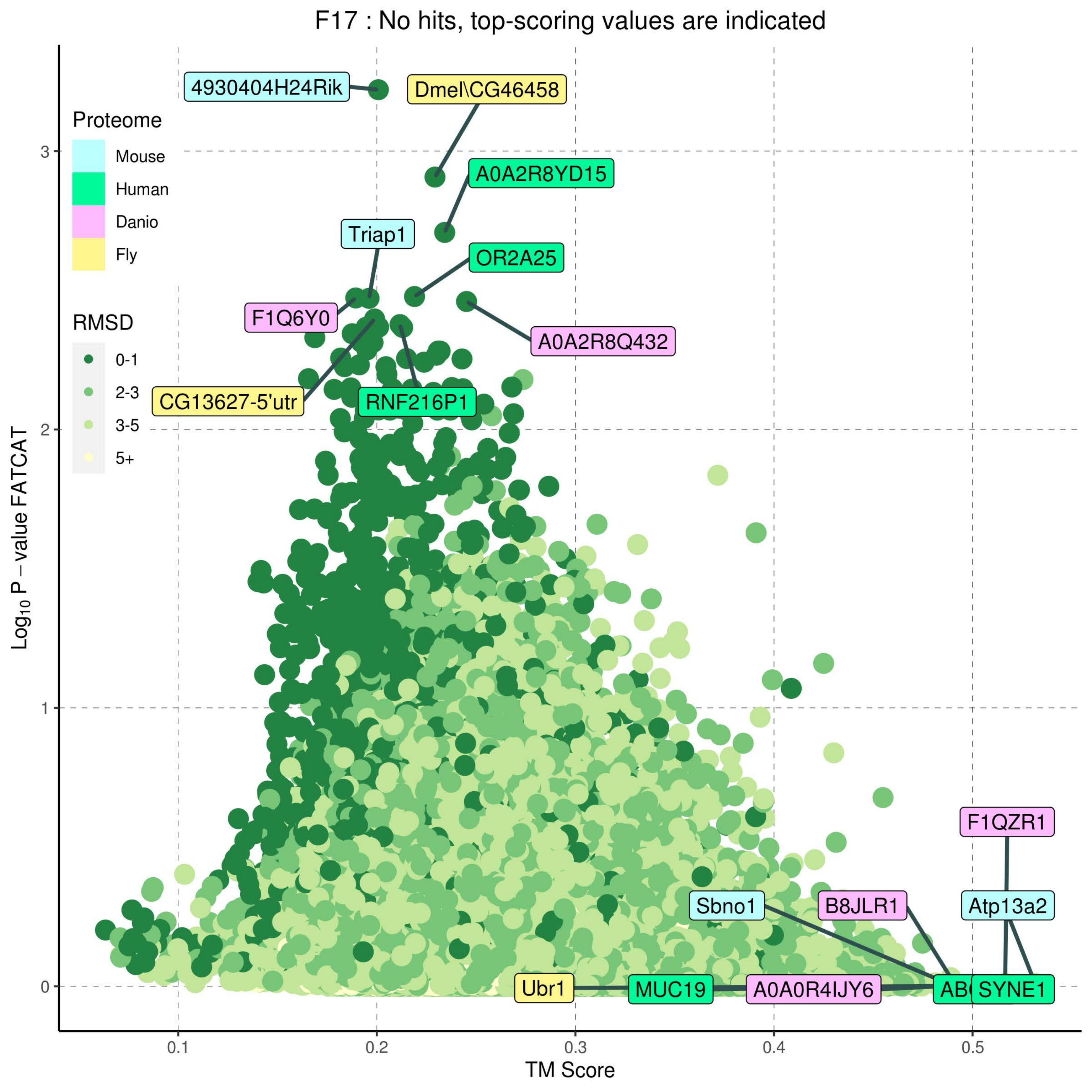

F17 : No hits, top-scoring values are indicated
Proteome
Mouse
Human
Danio
Fly
RMSD
0-1
2-3
3-5
5+
4930404H24Rik
Dmel\CG46458
A0A2R8YD15
Triap1
OR2A25
F1Q6Y0
A0A2R8Q432
CG13627-5'utr
RNF216P1
F1QZR1
Sbno1
B8JLR1
Atp13a2
Ubr1
MUC19
A0A0R4IJY6
SYNE1
Log_{10} P − value FATCAT
TM Score
0
1
2
3
0.1
0.2
0.3
0.4
0.5

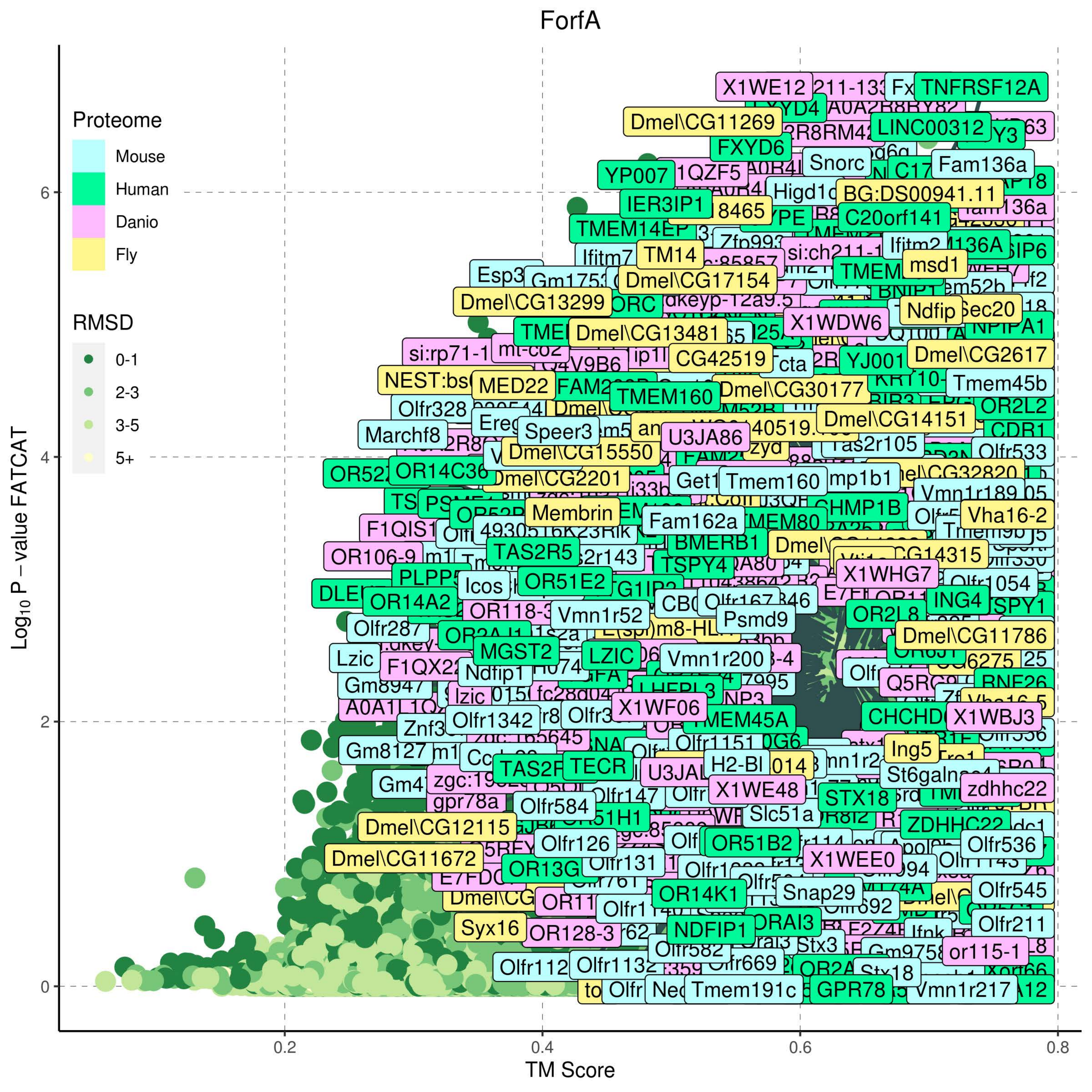
ForfA
Proteome
Mouse
Human
Danio
Fly
RMSD
0-1
2-3
3-5
5+
Log10 P − value FATCAT
0
2
4
6
0.2
0.4
0.6
0.8
TM Score

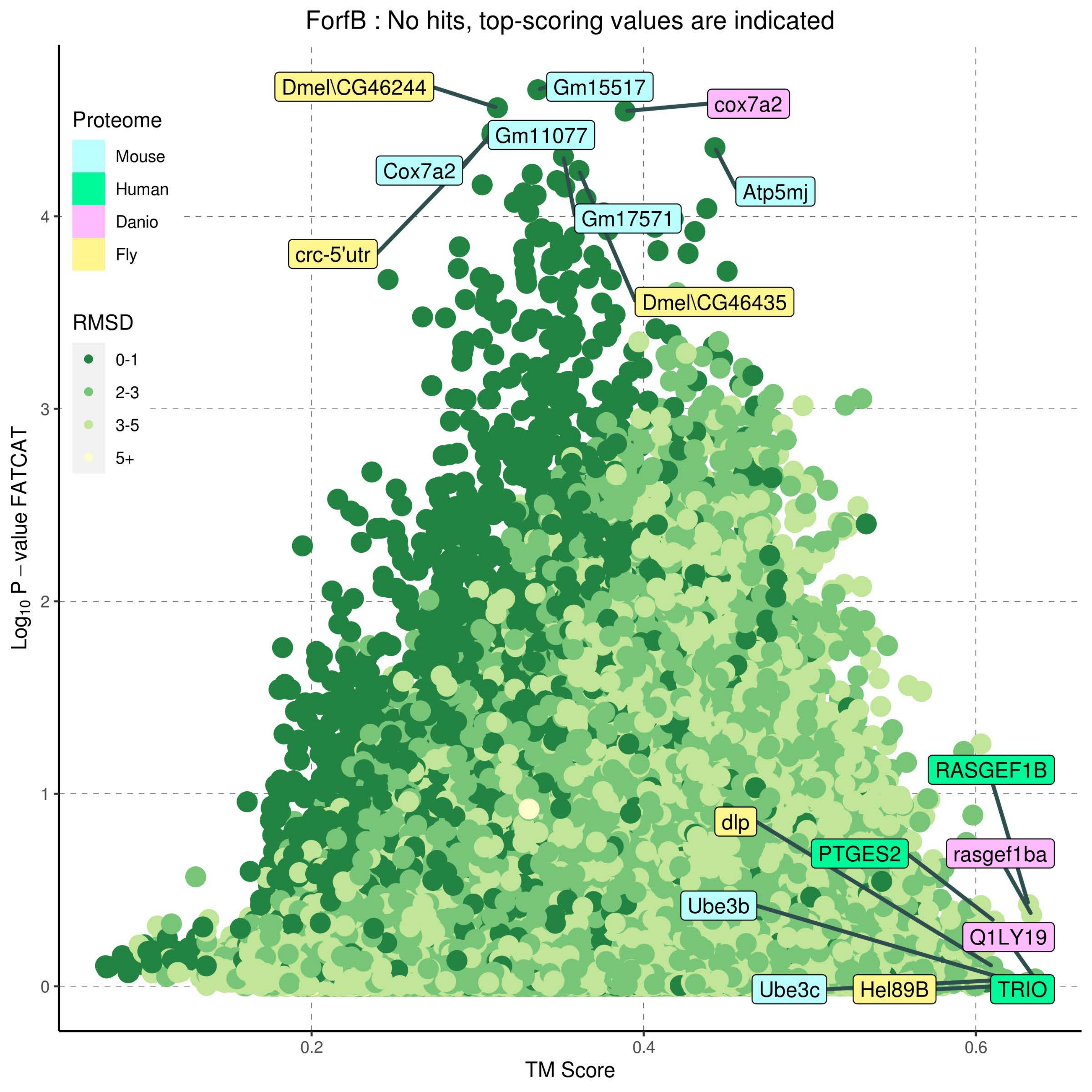
ForfB : No hits, top-scoring values are indicated
Proteome
Mouse
Human
Danio
Fly
RMSD
0-1
2-3
3-5
5+
Dmel\CG46244
Gm15517
cox7a2
Gm11077
Cox7a2
Atp5mj
Gm17571
crc-5'utr
Dmel\CG46435
RASGEF1B
dlp
PTGES2
rasgef1ba
Ube3b
Q1LY19
Ube3c
Hel89B
TRIO
Log10 P − value FATCAT
TM Score
0
1
2
3
4
0.2
0.4
0.6

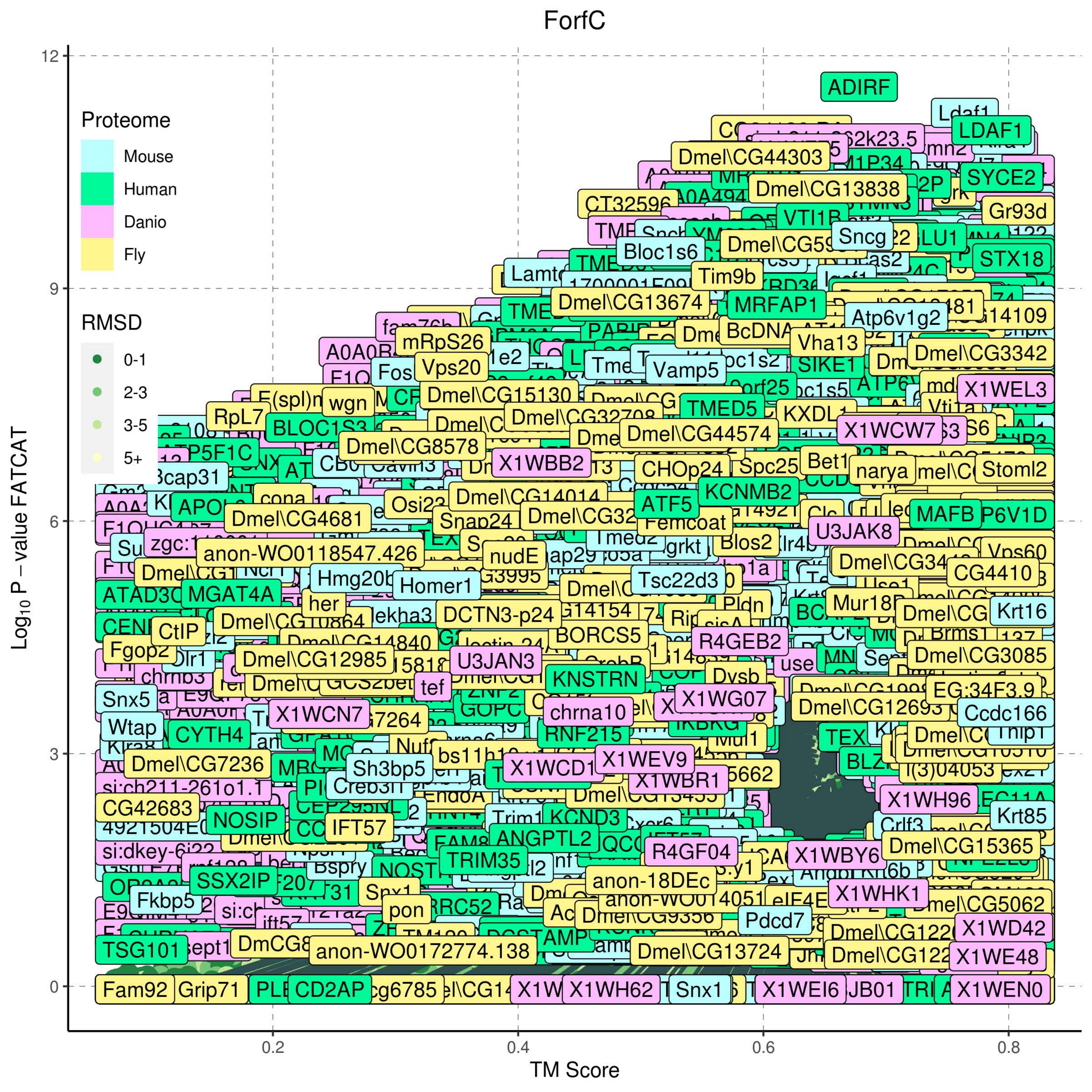
ForfC
Proteome
Mouse
Human
Danio
Fly
RMSD
0-1
2-3
3-5
5+
Log10 P – value FATCAT
TM Score
12
9
6
3
0
0.2
0.4
0.6
0.8
ADIRF
LDAF1
Dmel\CG44303
Dmel\CG13838
SYCE2
Gr93d
VTI1B
Sncg
STX18
Bloc1s6
Tim9b
Dmel\CG13674
MRFAP1
Atp6v1g2
mRpS26
Vha13
Dmel\CG3342
Vps20
Vamp5
X1WEL3
Dmel\CG15130
TMED5
RpL7
BLOC1S3
Dmel\CG44574
X1WCW7
Dmel\CG8578
X1WBB2
CHOp24
Spc25
Stoml2
KCNMB2
Dmel\CG4681
ATF5
Femcoat
MAFB
U3JAK8
anon-WO0118547.426
nudE
Tsc22d3
CG4410
Homer1
DCTN3-p24
BORCS5
R4GEB2
Krt16
Dmel\CG12985
U3JAN3
KNSTRN
Dmel\CG3085
X1WG07
Dmel\CG12693
Snx5
X1WCN7
chrna10
Ccdc166
CYTH4
RNF215
Dmel\CG7236
Sh3bp5
X1WEV9
X1WBR1
si:ch211-261o1.1
CG42683
NOSIP
IFT57
X1WH96
Krt85
ANGPTL2
TRIM35
R4GF04
X1WBY6
Dmel\CG15365
SSX2IP
anon-18DEc
X1WHK1
Fkbp5
Pdcd7
Dmel\CG5062
X1WD42
TSG101
anon-WO0172774.138
Dmel\CG13724
X1WE48
Fam92
Grip71
CD2AP
X1WH62
Snx1
X1WEI6
X1WEN0

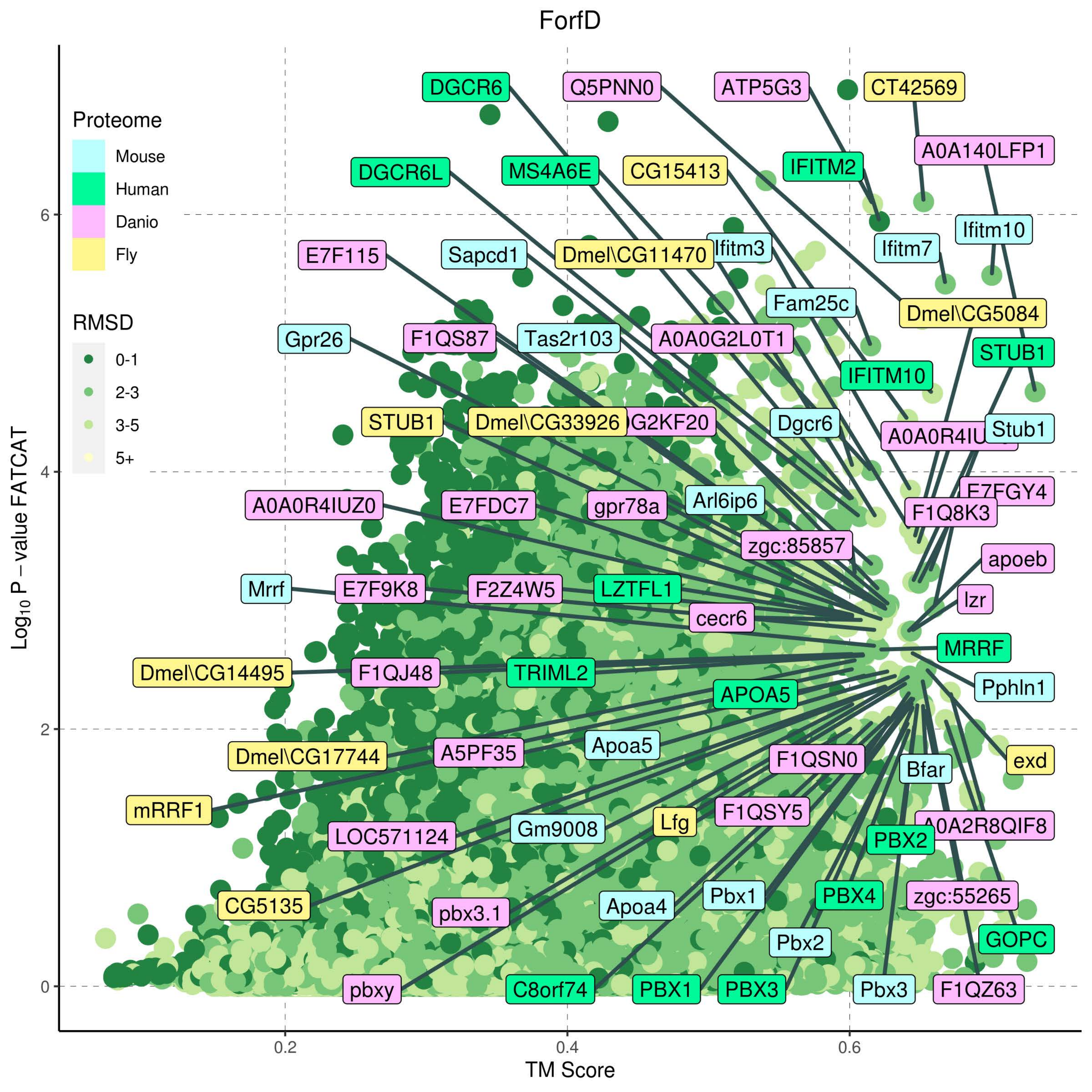
ForfD
Proteome
Mouse
Human
Danio
Fly
RMSD
0-1
2-3
3-5
5+
Log₁₀ P – value FATCAT
TM Score
0
2
4
6
0.2
0.4
0.6
DGCR6
Q5PNN0
ATP5G3
CT42569
A0A140LFP1
DGCR6L
MS4A6E
CG15413
IFITM2
Ifitm10
E7F115
Sapcd1
Dmel\CG11470
Ifitm3
Ifitm7
Fam25c
Dmel\CG5084
Gpr26
F1QS87
Tas2r103
A0A0G2L0T1
STUB1
IFITM10
STUB1
Dmel\CG33926
Dgcr6
Stub1
Arl6ip6
E7FGY4
A0A0R4IUZ0
E7FDC7
gpr78a
F1Q8K3
zgc:85857
apoeb
Mrrf
E7F9K8
F2Z4W5
LZTFL1
lzr
cecr6
MRRF
Dmel\CG14495
F1QJ48
TRIML2
Pphln1
APOA5
Apoa5
Dmel\CG17744
A5PF35
F1QSN0
Bfar
exd
mRRF1
F1QSY5
Gm9008
Lfg
A0A2R8QIF8
LOC571124
PBX2
Pbx1
PBX4
zgc:55265
CG5135
Apoa4
pbx3.1
GOPC
Pbx2
pbxy
C8orf74
PBX1
PBX3
Pbx3
F1QZ63

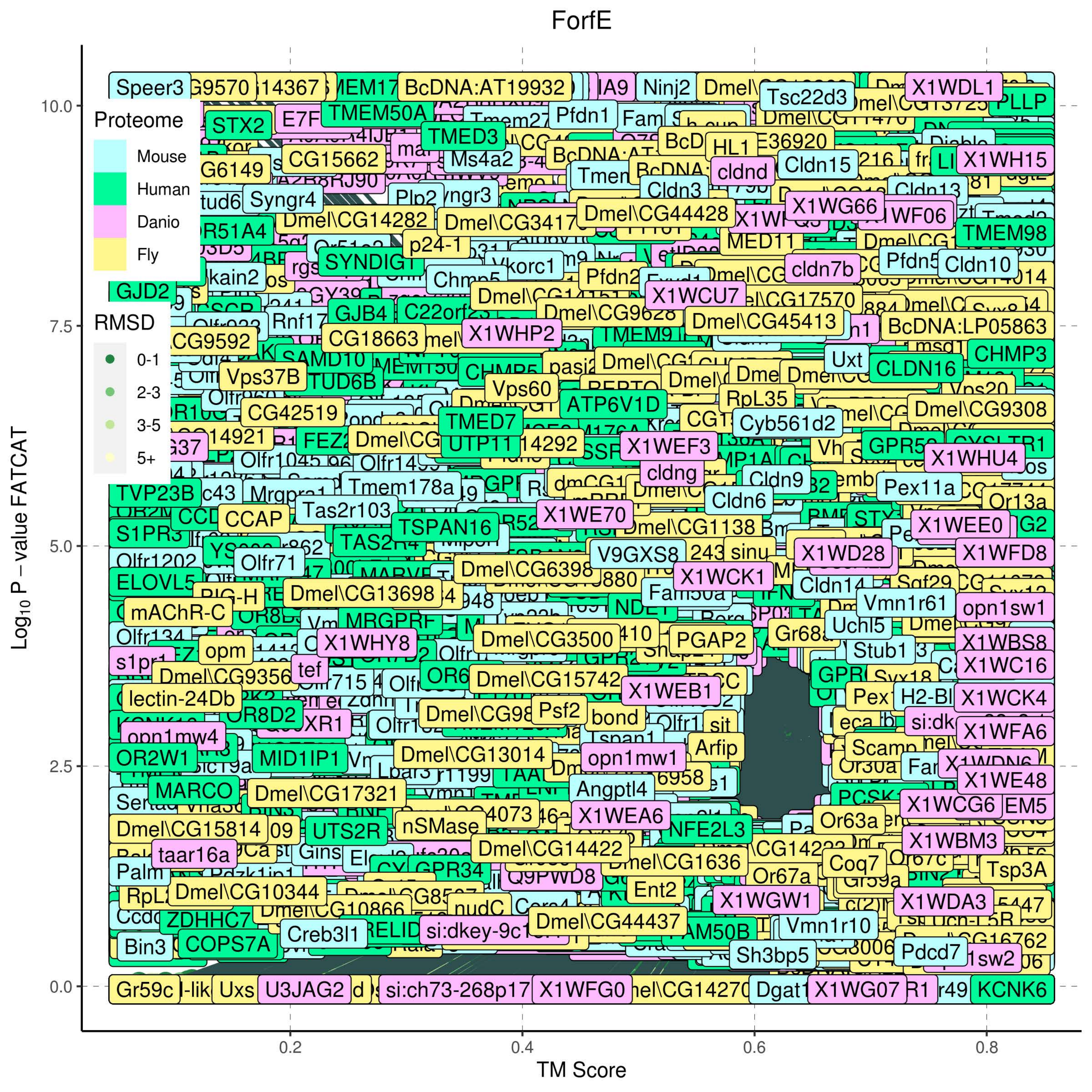

ForfE
Proteome
Mouse
Human
Danio
Fly
RMSD
0-1
2-3
3-5
5+
10.0
7.5
5.0
2.5
0.0
Log_{10} P − value FATCAT
0.2
0.4
0.6
0.8
TM Score

ForfF

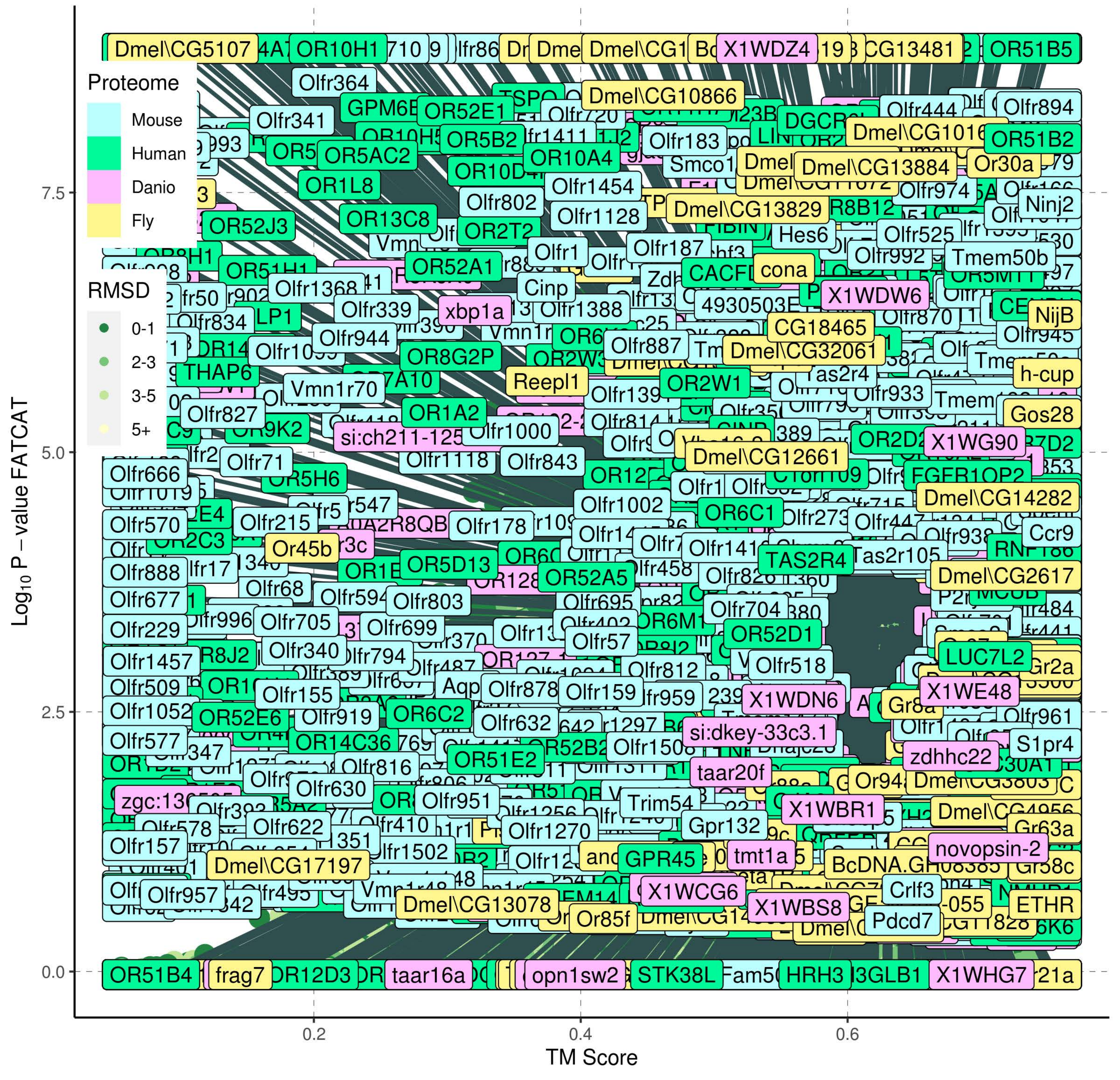

Proteome
Mouse
Human
Danio
Fly
RMSD
0-1
2-3
3-5
5+
Log_{10} P – value FATCAT
TM Score
0.0
2.5
5.0
7.5
0.2
0.4
0.6
Dmel\CG5107
OR10H1
X1WDZ4
CG13481
OR51B5
Olfr364
Dmel\CG10866
Olfr894
Olfr341
OR52E1
OR5AC2
OR5B2
OR10A4
Olfr183
OR51B2
Dmel\CG13884
Or30a
OR1L8
Olfr1454
Olfr802
Olfr974
Ninj2
OR52J3
OR13C8
Olfr1128
Dmel\CG13829
OR2T2
Hes6
Olfr525
Olfr1
Olfr187
Olfr992
Tmem50b
OR52A1
cona
Olfr1368
Cinp
X1WDW6
Olfr339
xbp1a
Olfr1388
CG18465
NijB
Olfr834
Olfr944
Olfr887
Dmel\CG32061
Olfr945
OR8G2P
THAP6
Reepl1
OR2W1
Olfr933
h-cup
Vmn1r70
Olfr827
OR1A2
Gos28
Olfr1000
X1WG90
Olfr71
Olfr1118
Olfr843
Dmel\CG12661
Olfr666
Dmel\CG14282
Olfr1002
OR6C1
Olfr570
Olfr215
Olfr178
Ccr9
Or45b
OR5D13
TAS2R4
Tas2r105
Olfr888
OR52A5
Dmel\CG2617
Olfr68
Olfr803
Olfr677
Olfr704
Olfr229
Olfr705
Olfr699
OR52D1
Olfr57
Olfr1457
Olfr340
LUC7L2
Olfr518
Olfr812
Olfr509
Olfr155
Olfr878
Olfr159
X1WDN6
X1WE48
Olfr1052
OR52E6
Olfr919
OR6C2
Olfr632
Olfr961
si:dkey-33c3.1
Olfr577
OR14C36
OR52B2
Olfr1508
S1pr4
zdhhc22
Olfr816
OR51E2
taar20f
Olfr630
Olfr951
Trim54
X1WBR1
Olfr622
Gpr132
Olfr1270
Gr63a
Olfr157
Dmel\CG17197
GPR45
tmt1a
novopsin-2
Olfr957
X1WCG6
X1WBS8
Crlf3
Dmel\CG13078
Or85f
Pdcd7
ETHR
OR51B4
frag7
taar16a
opn1sw2
STK38L
HRH3
X1WHG7

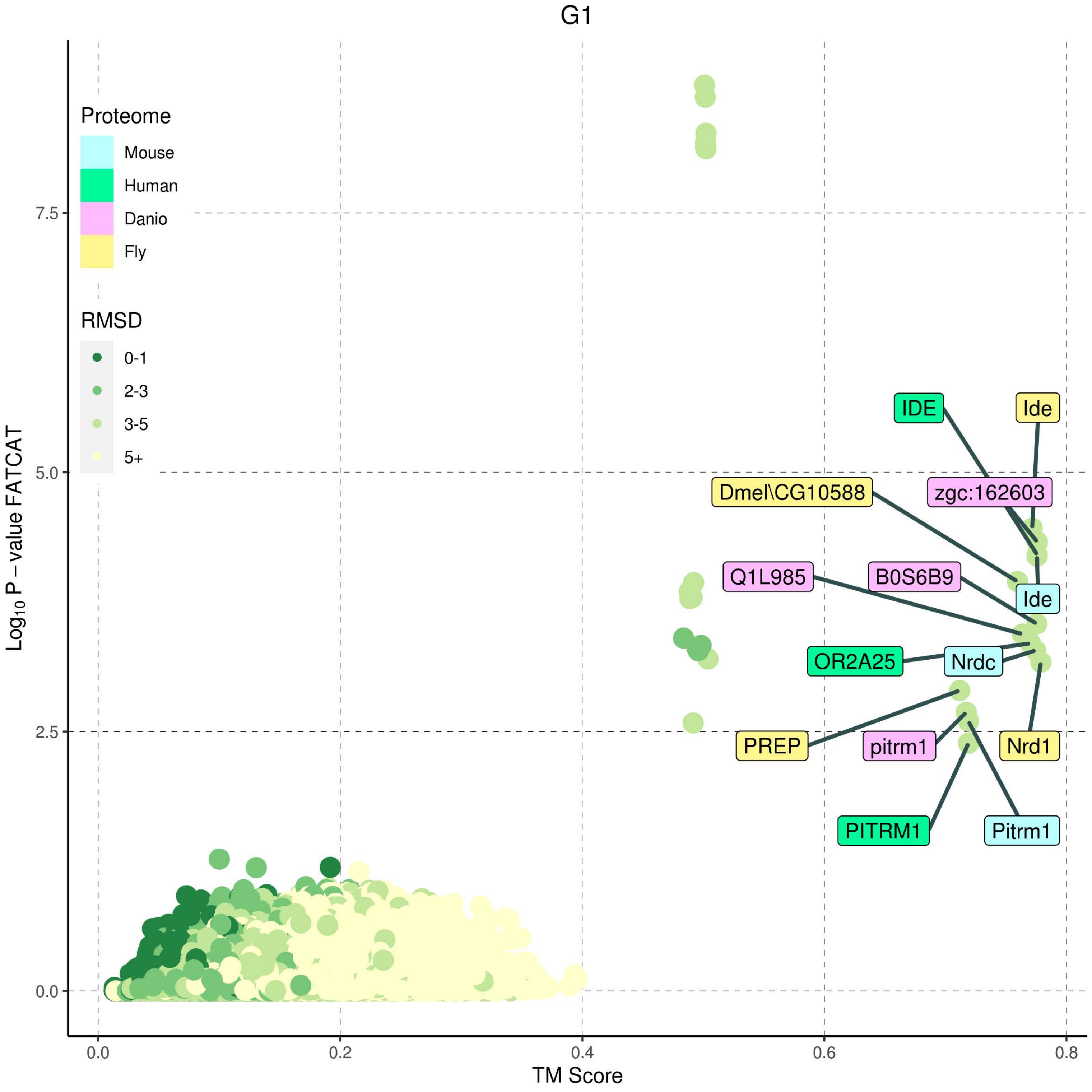
G1
Proteome
Mouse
Human
Danio
Fly
RMSD
0-1
2-3
3-5
5+
IDE
Ide
Dmel\CG10588
zgc:162603
Q1L985
B0S6B9
Ide
OR2A25
Nrdc
PREP
pitrm1
Nrd1
PITRM1
Pitrm1
7.5
5.0
2.5
0.0
0.0
0.2
0.4
0.6
0.8
Log_{10} P − value FATCAT
TM Score

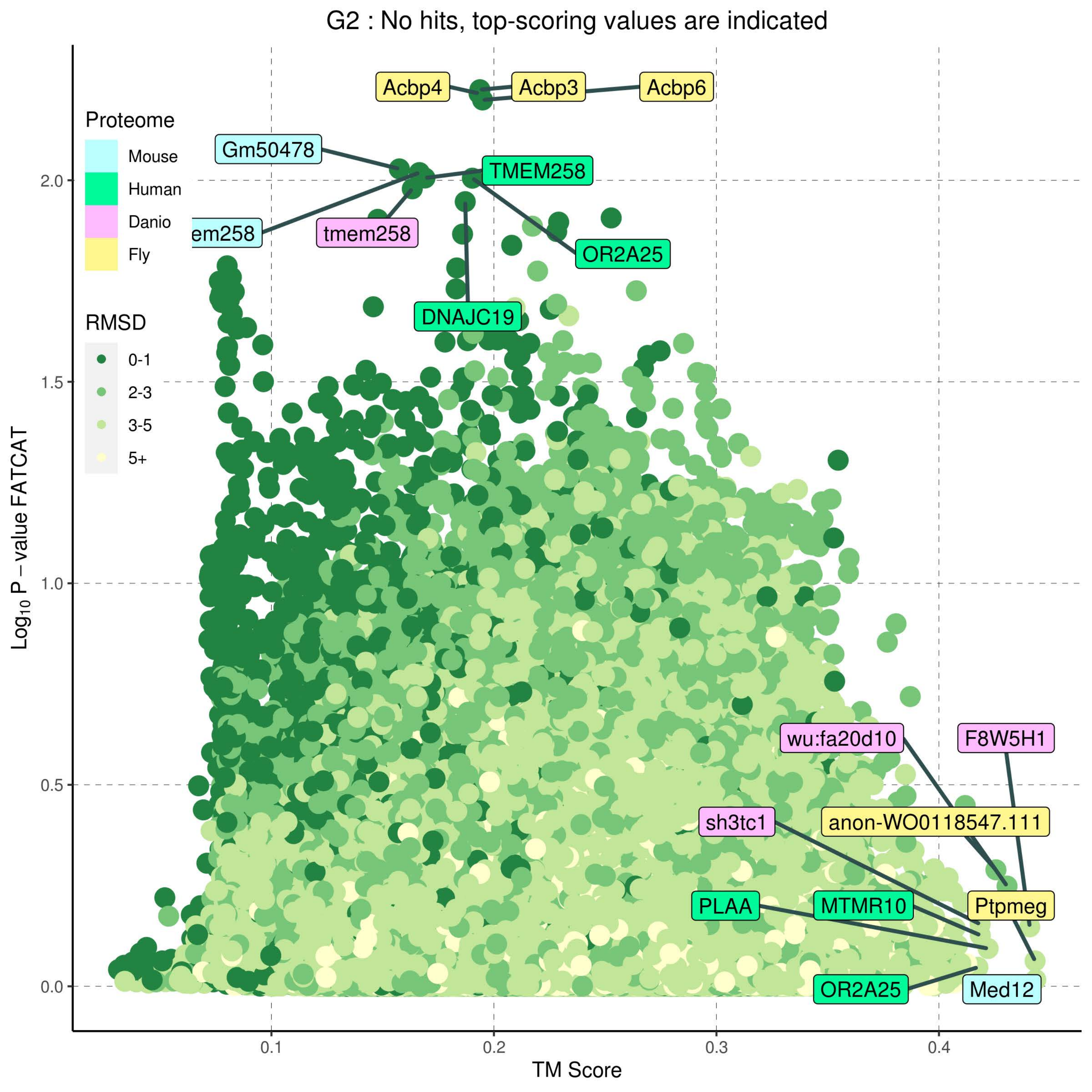

G2 : No hits, top-scoring values are indicated
Proteome
Mouse
Human
Danio
Fly
RMSD
0-1
2-3
3-5
5+
Acbp4
Acbp3
Acbp6
Gm50478
TMEM258
em258
tmem258
OR2A25
DNAJC19
wu:fa20d10
F8W5H1
sh3tc1
anon-WO0118547.111
PLAA
MTMR10
Ptpmeg
OR2A25
Med12
2.0
1.5
1.0
0.5
0.0
0.1
0.2
0.3
0.4
TM Score
Log10 P – value FATCAT

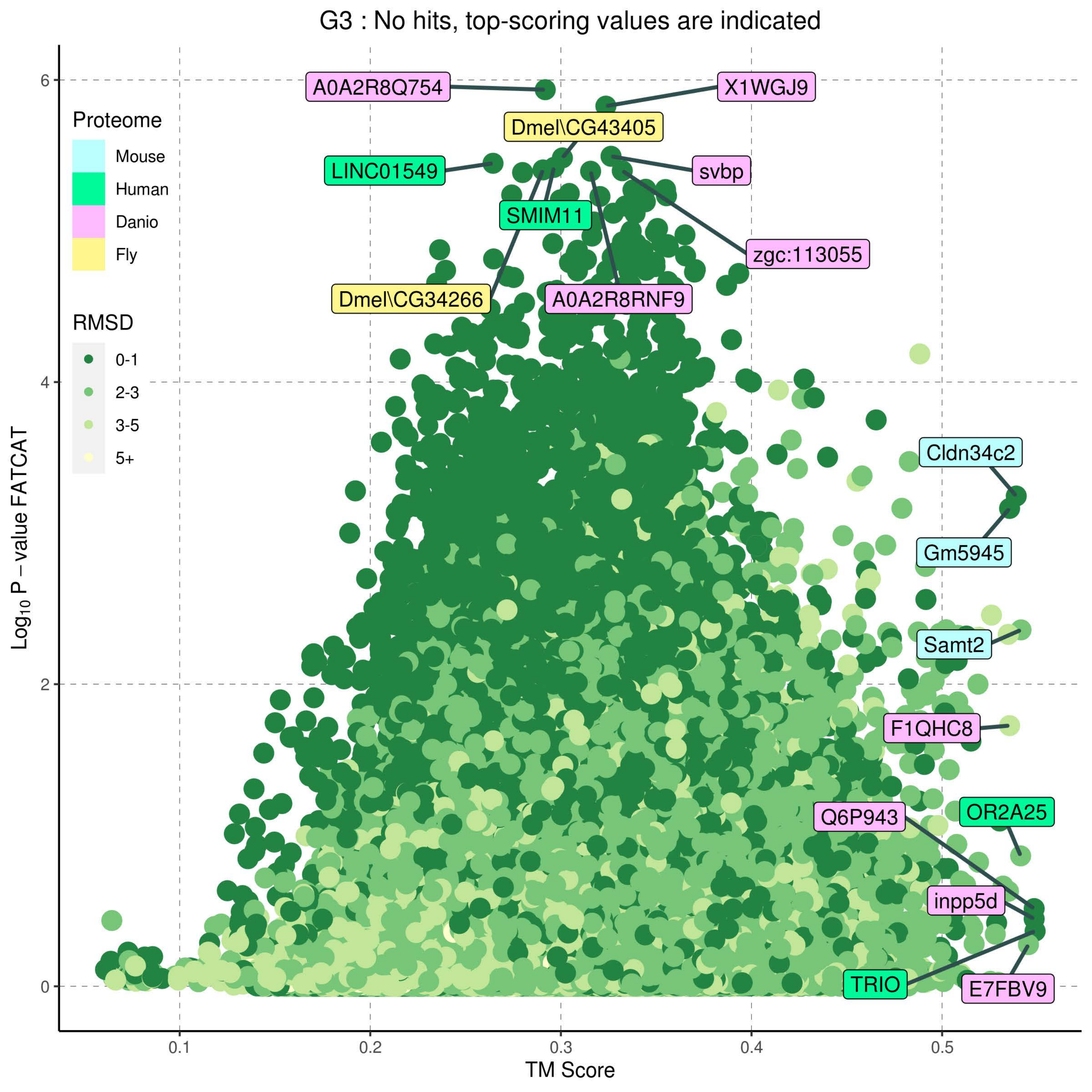
G3 : No hits, top-scoring values are indicated
Proteome
Mouse
Human
Danio
Fly
RMSD
0-1
2-3
3-5
5+
A0A2R8Q754
X1WGJ9
Dmel\CG43405
LINC01549
svbp
SMIM11
zgc:113055
Dmel\CG34266
A0A2R8RNF9
Cldn34c2
Gm5945
Samt2
F1QHC8
OR2A25
Q6P943
inpp5d
TRIO
E7FBV9
Log10 P − value FATCAT
TM Score
0
2
4
6
0.1
0.2
0.3
0.4
0.5

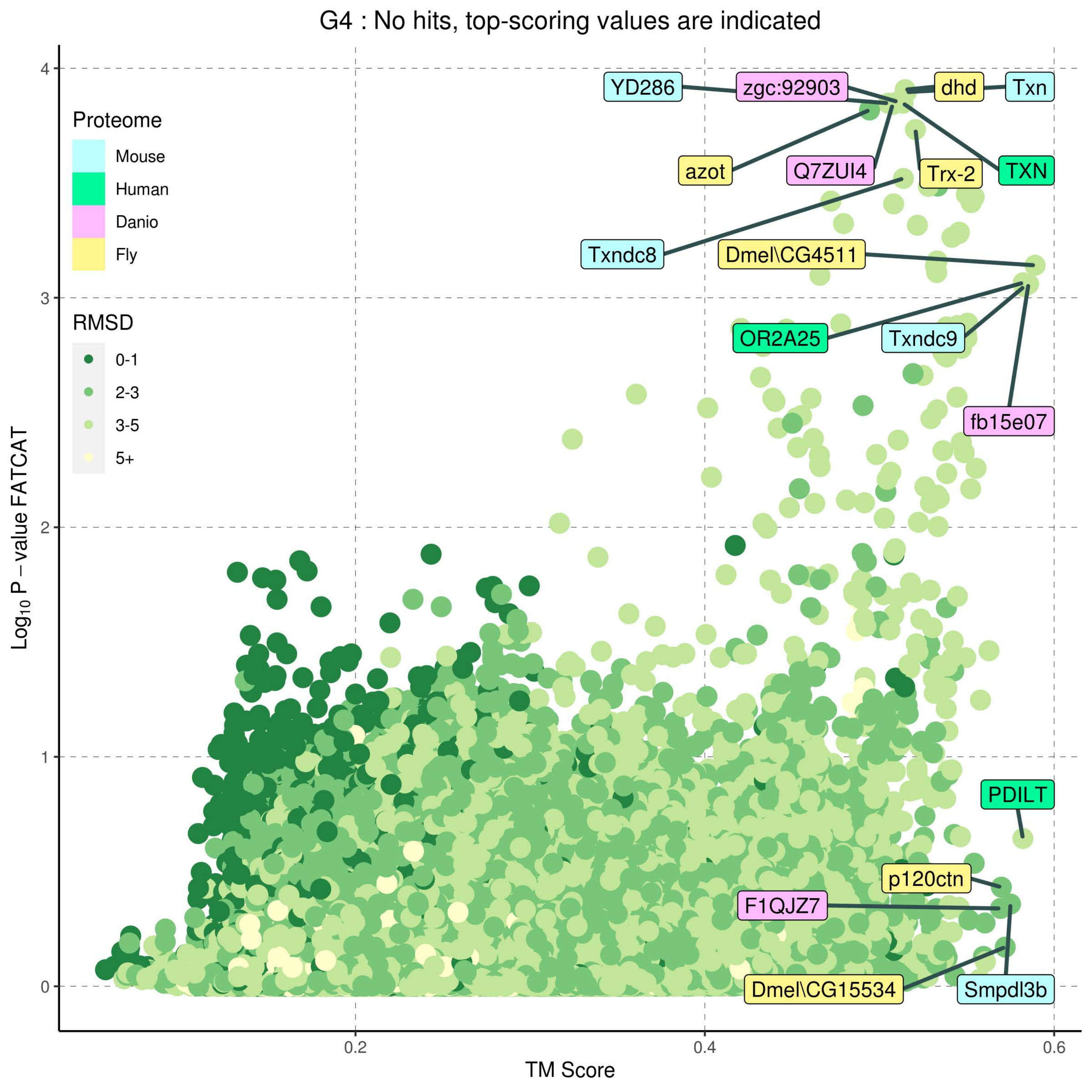

G4 : No hits, top-scoring values are indicated
Proteome
Mouse
Human
Danio
Fly
RMSD
0-1
2-3
3-5
5+
YD286
zgc:92903
dhd
Txn
azot
Q7ZUI4
Trx-2
TXN
Txndc8
Dmel\CG4511
OR2A25
Txndc9
fb15e07
PDILT
p120ctn
F1QJZ7
Dmel\CG15534
Smpdl3b
0
1
2
3
4
0.2
0.4
0.6
TM Score
Log10 P − value FATCAT

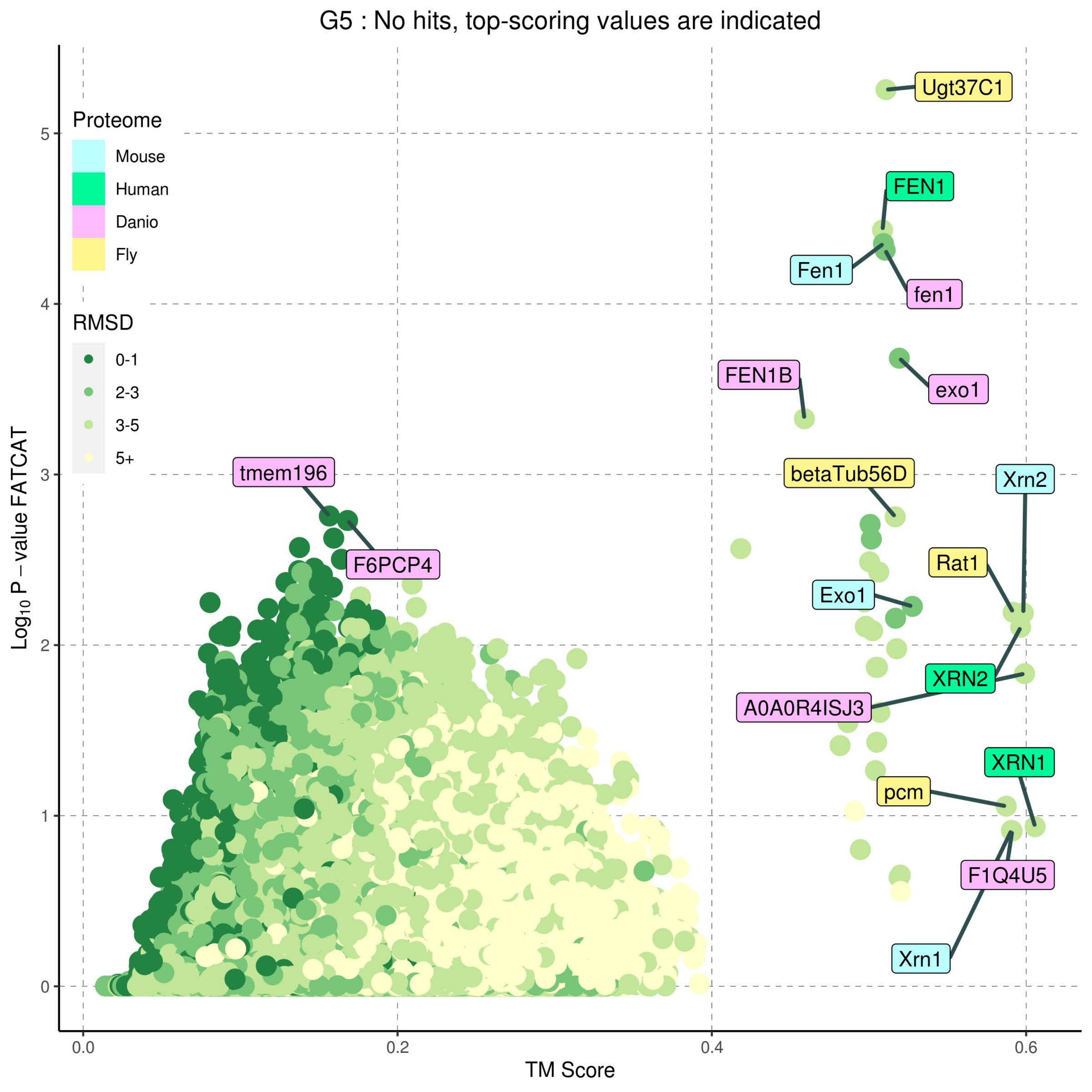
G5 : No hits, top-scoring values are indicated
Proteome
Mouse
Human
Danio
Fly
RMSD
0-1
2-3
3-5
5+
Log10 P − value FATCAT
TM Score
0.0
0.2
0.4
0.6
0
1
2
3
4
5
Ugt37C1
FEN1
Fen1
fen1
FEN1B
exo1
tmem196
F6PCP4
betaTub56D
Xrn2
Rat1
Exo1
XRN2
A0A0R4ISJ3
XRN1
pcm
F1Q4U5
Xrn1

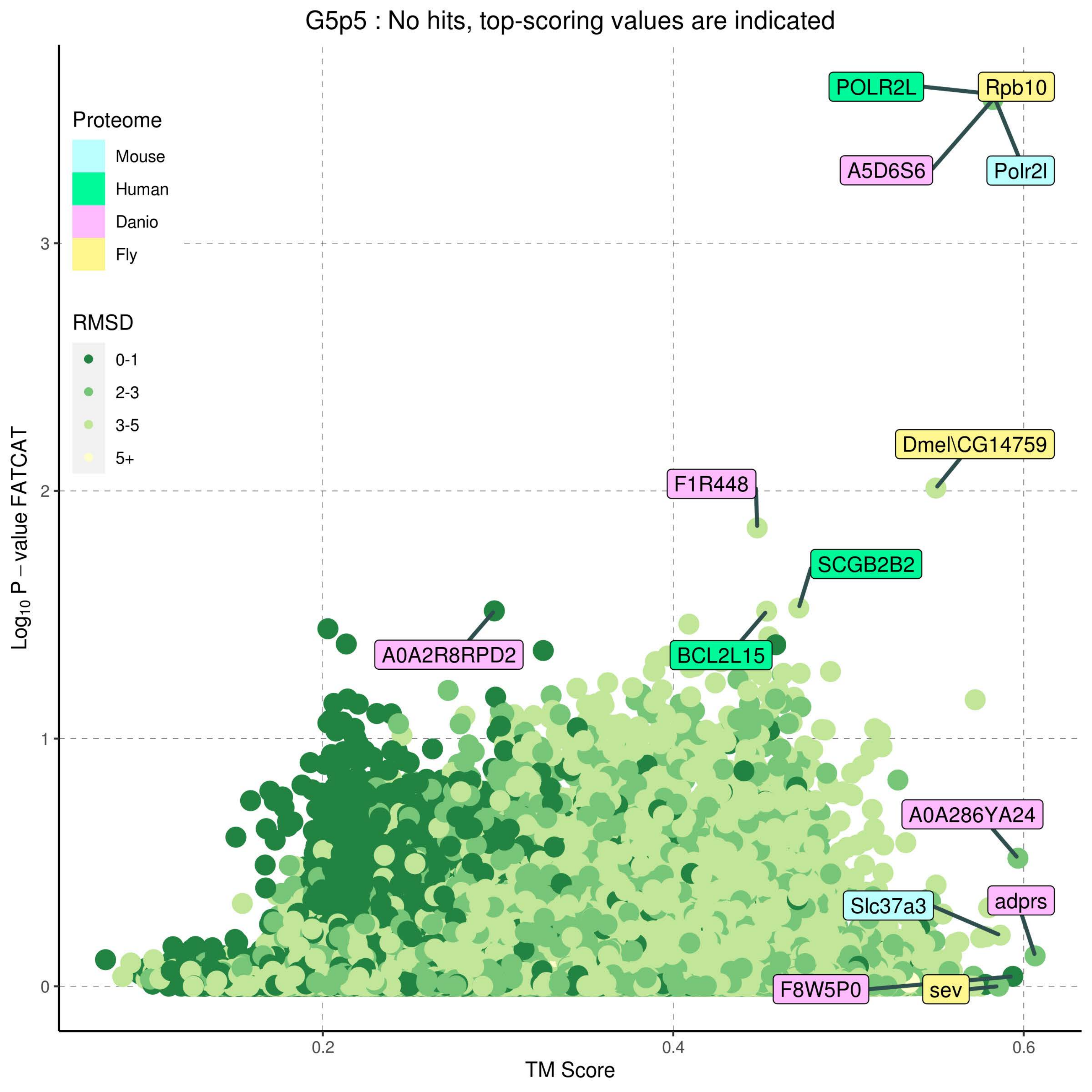

G5p5 : No hits, top-scoring values are indicated
Proteome
Mouse
Human
Danio
Fly
RMSD
0-1
2-3
3-5
5+
POLR2L
Rpb10
A5D6S6
Polr2l
Dmel\CG14759
F1R448
SCGB2B2
A0A2R8RPD2
BCL2L15
A0A286YA24
Slc37a3
adprs
F8W5P0
sev
Log10 P − value FATCAT
TM Score
0
1
2
3
0.2
0.4
0.6

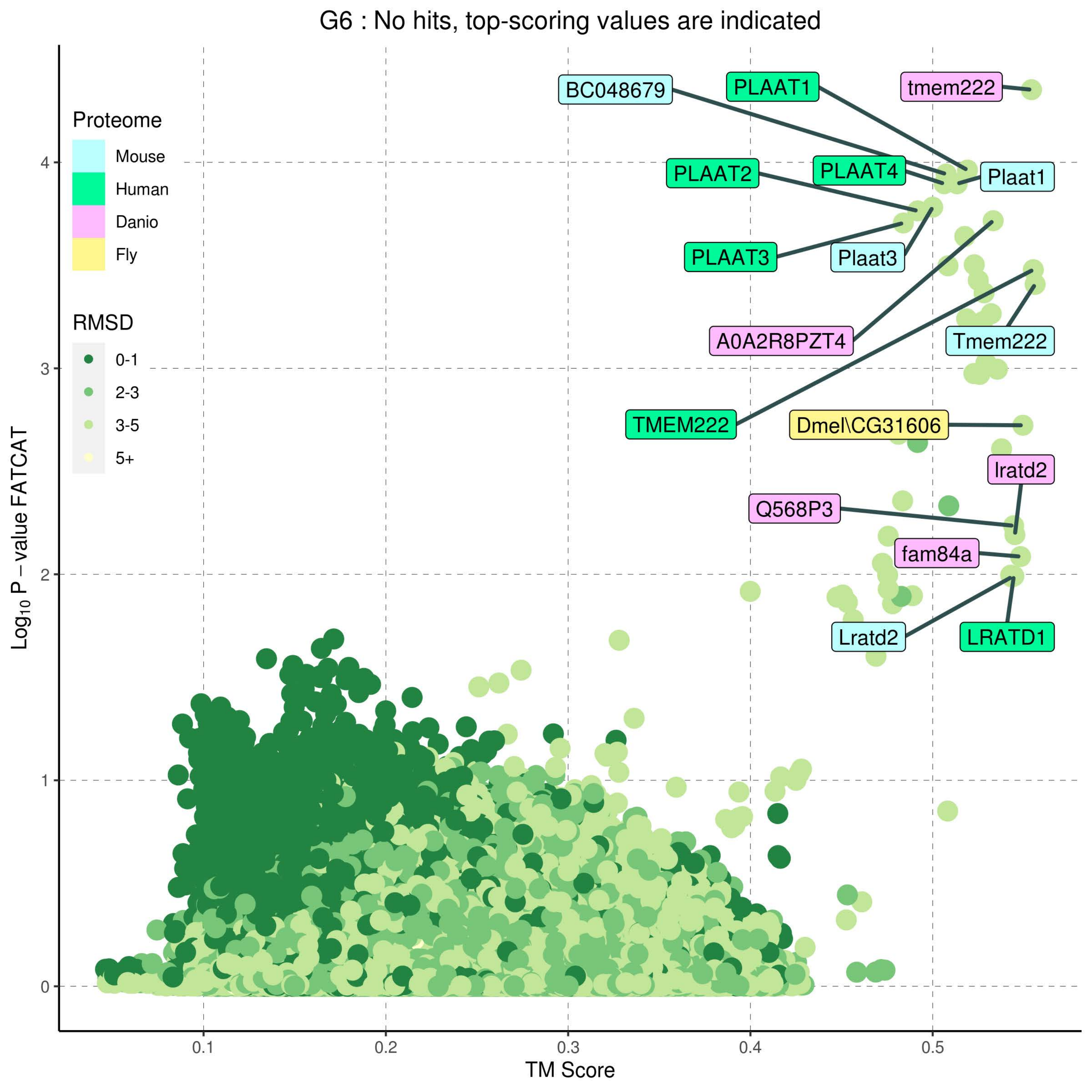
G6 : No hits, top-scoring values are indicated
Proteome
Mouse
Human
Danio
Fly
RMSD
0-1
2-3
3-5
5+
BC048679
PLAAT1
tmem222
PLAAT2
PLAAT4
Plaat1
PLAAT3
Plaat3
A0A2R8PZT4
Tmem222
TMEM222
Dmel\CG31606
lratd2
Q568P3
fam84a
Lratd2
LRATD1
Log10 P − value FATCAT
TM Score
0
1
2
3
4
0.1
0.2
0.3
0.4
0.5

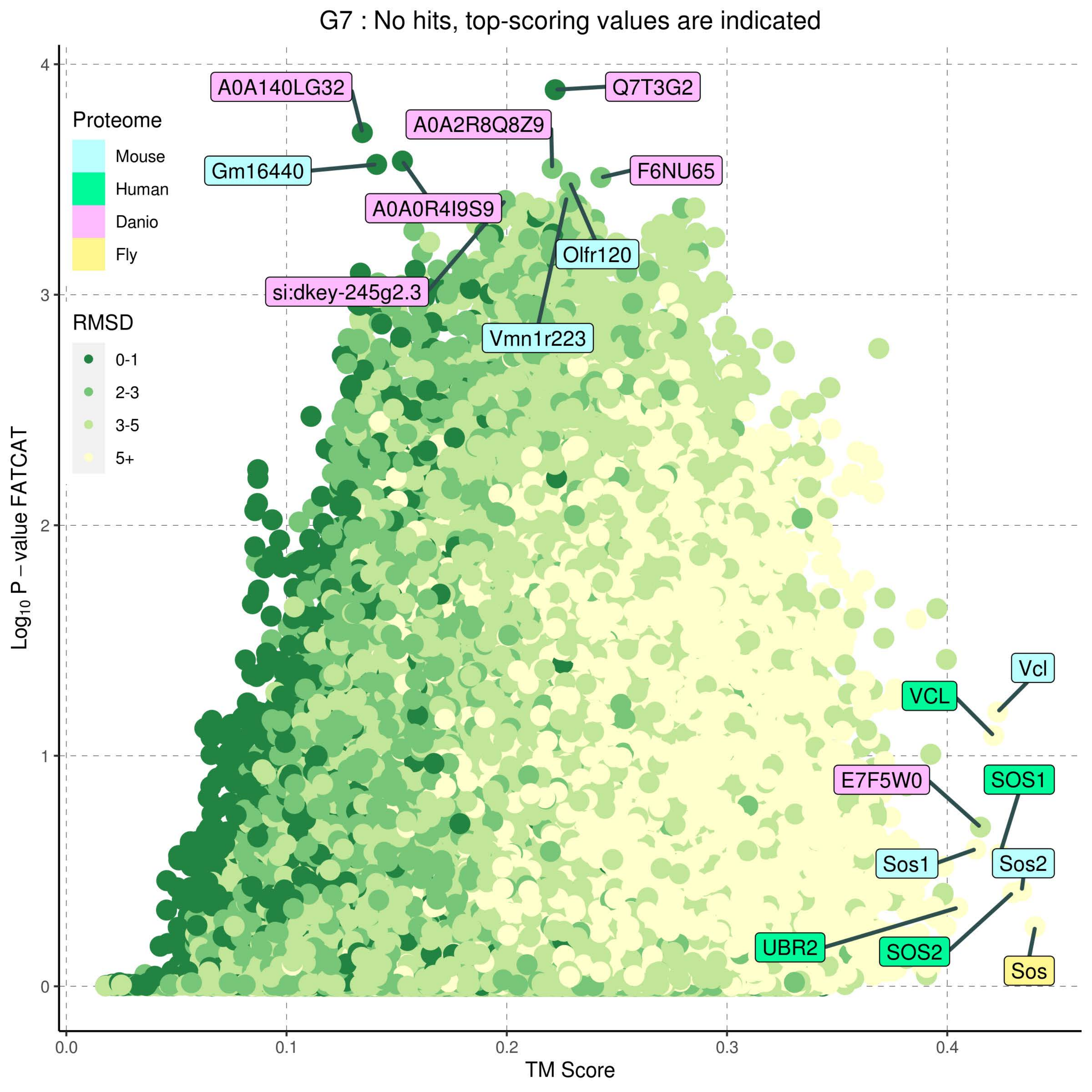
G7 : No hits, top-scoring values are indicated
Proteome
Mouse
Human
Danio
Fly
RMSD
0-1
2-3
3-5
5+
A0A140LG32
Q7T3G2
A0A2R8Q8Z9
Gm16440
F6NU65
A0A0R4I9S9
Olfr120
si:dkey-245g2.3
Vmn1r223
Vcl
VCL
E7F5W0
SOS1
Sos1
Sos2
UBR2
SOS2
Sos
Log_{10} P – value FATCAT
TM Score
0
1
2
3
4
0.0
0.1
0.2
0.3
0.4

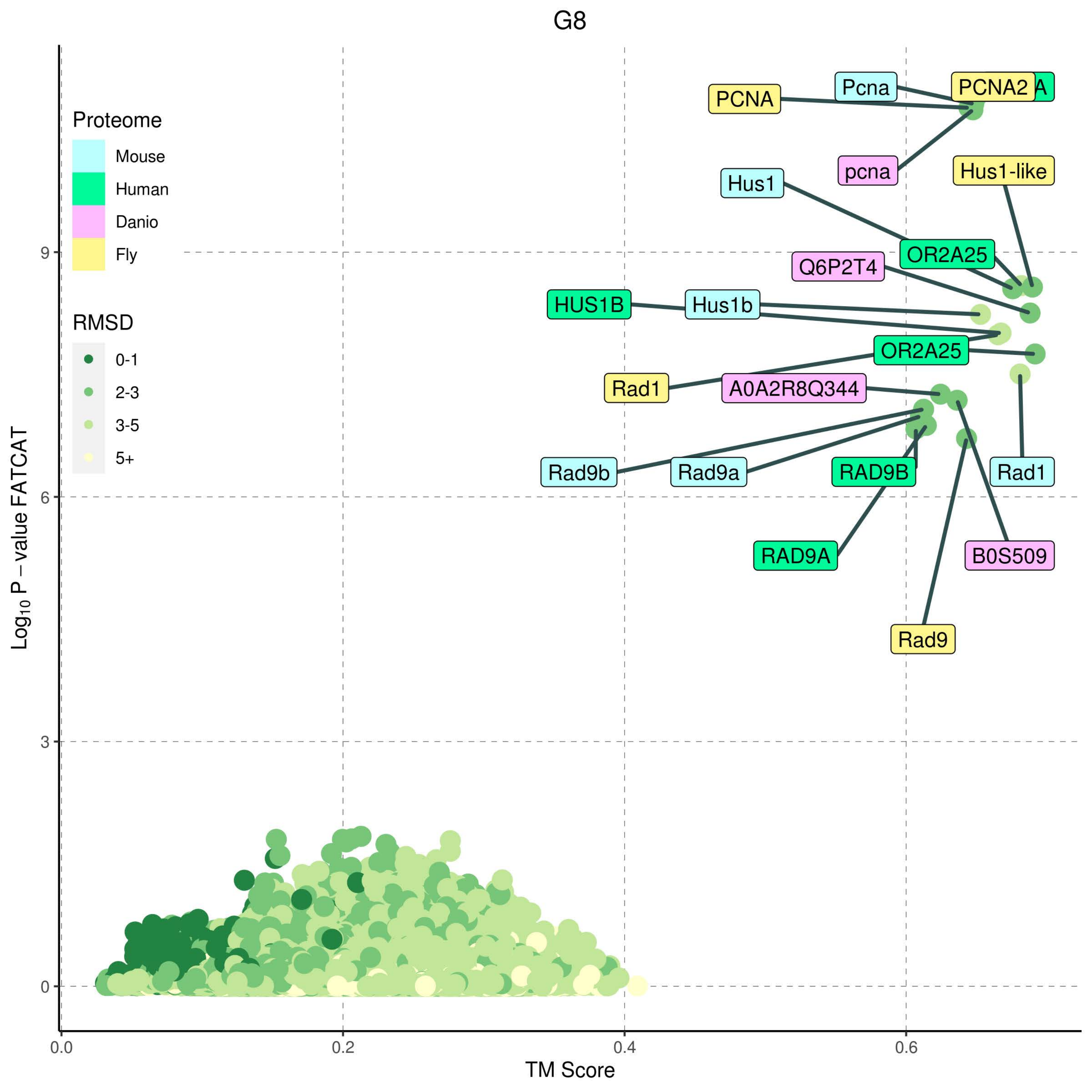
G8
Proteome
Mouse
Human
Danio
Fly
RMSD
0-1
2-3
3-5
5+
PCNA
Pcna
PCNA2
pcna
Hus1
Hus1-like
OR2A25
Q6P2T4
HUS1B
Hus1b
OR2A25
Rad1
A0A2R8Q344
Rad9b
Rad9a
RAD9B
Rad1
RAD9A
B0S509
Rad9
Log10 P − value FATCAT
TM Score
0
3
6
9
0.0
0.2
0.4
0.6

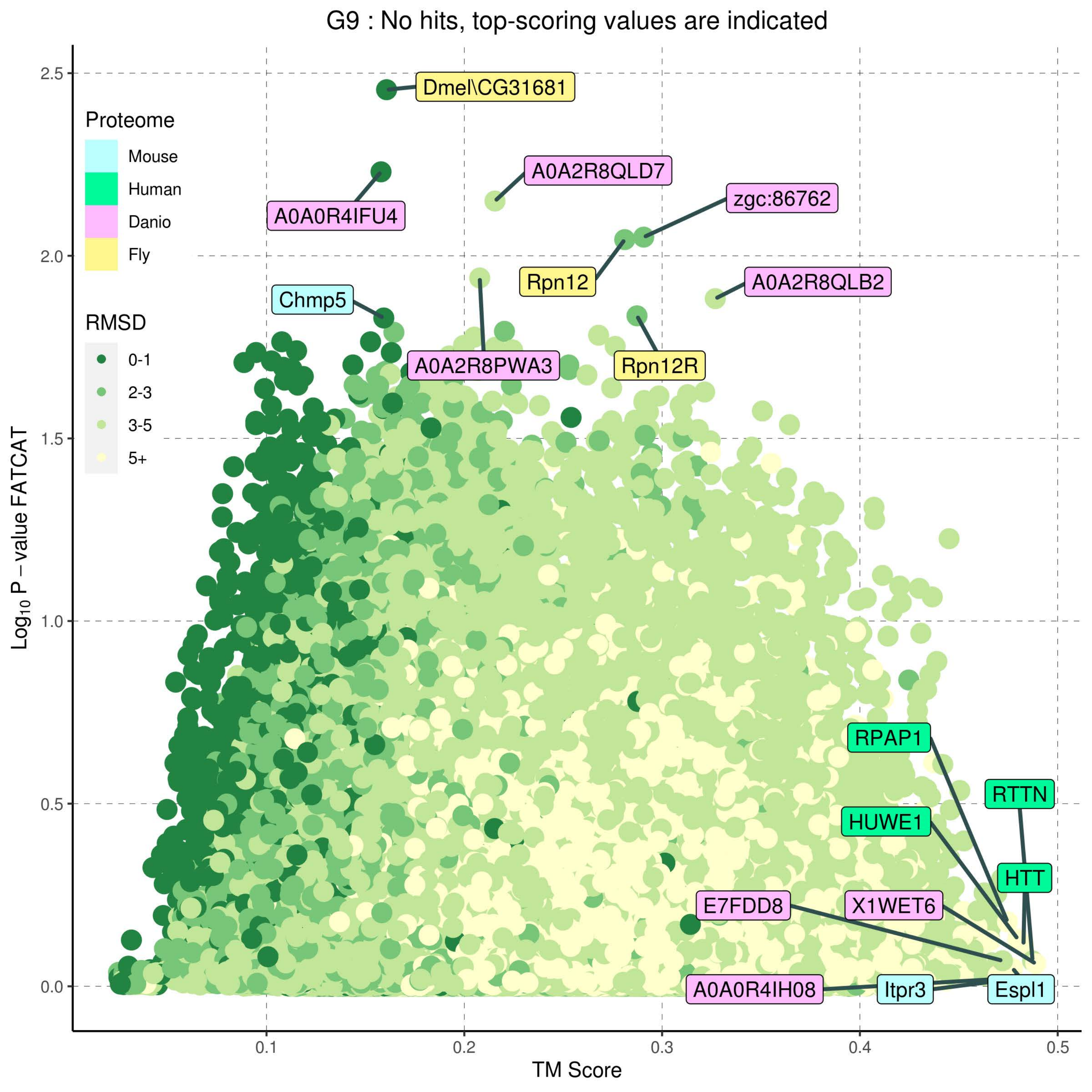
G9 : No hits, top-scoring values are indicated
Proteome
Mouse
Human
Danio
Fly
RMSD
0-1
2-3
3-5
5+
Dmel\CG31681
A0A2R8QLD7
A0A0R4IFU4
zgc:86762
Rpn12
A0A2R8QLB2
Chmp5
A0A2R8PWA3
Rpn12R
RPAP1
RTTN
HUWE1
HTT
E7FDD8
X1WET6
A0A0R4IH08
Itpr3
Espl1
Log_{10} P − value FATCAT
TM Score
0.0
0.5
1.0
1.5
2.0
2.5
0.1
0.2
0.3
0.4
0.5

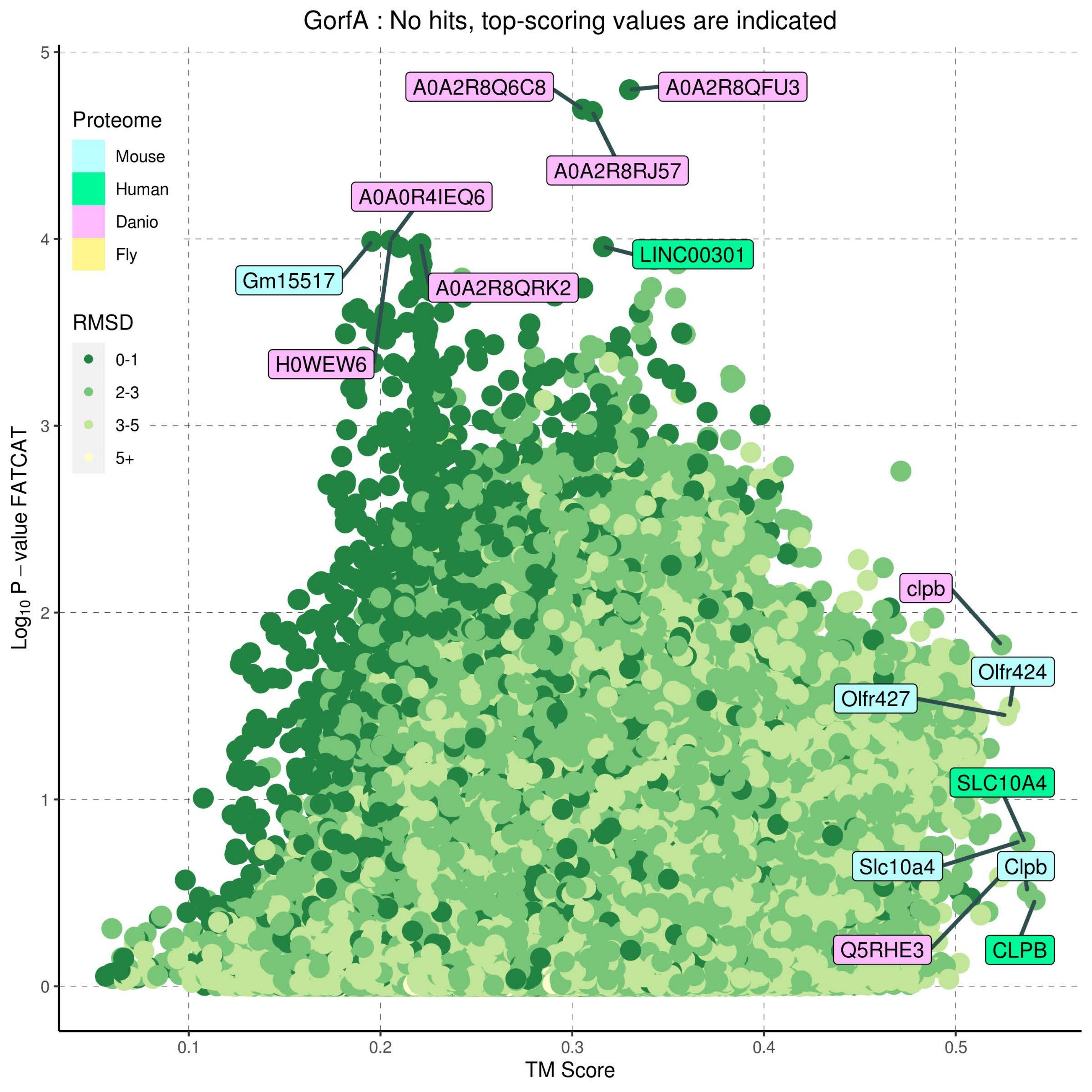
GorfA : No hits, top-scoring values are indicated
Proteome
Mouse
Human
Danio
Fly
RMSD
0-1
2-3
3-5
5+
A0A2R8Q6C8
A0A2R8QFU3
A0A2R8RJ57
A0A0R4IEQ6
Gm15517
LINC00301
A0A2R8QRK2
H0WEW6
clpb
Olfr424
Olfr427
SLC10A4
Slc10a4
Clpb
Q5RHE3
CLPB
Log10 P − value FATCAT
TM Score
0
1
2
3
4
5
0.1
0.2
0.3
0.4
0.5

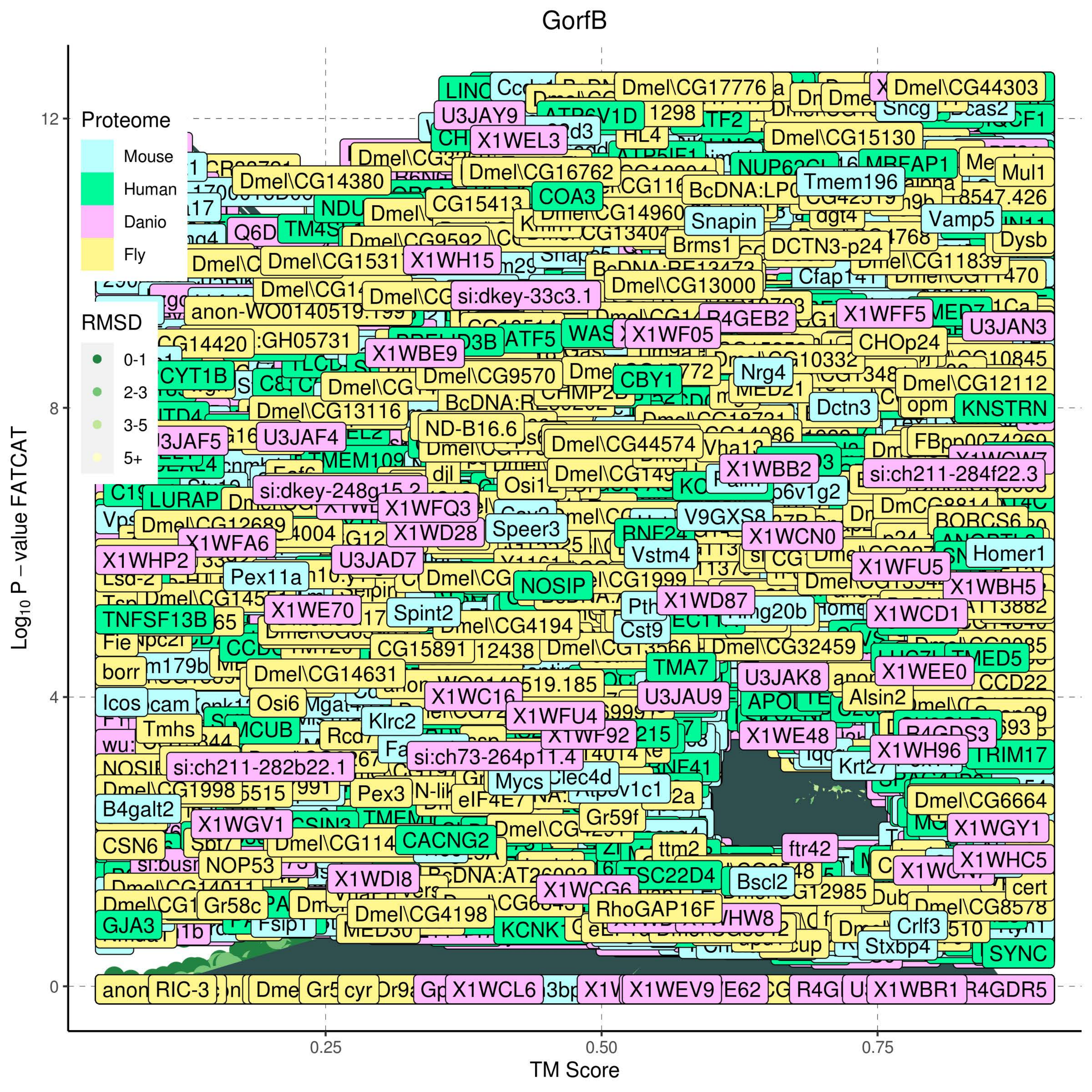

GorfB
Proteome
Mouse
Human
Danio
Fly
RMSD
0-1
2-3
3-5
5+
Log10 P – value FATCAT
TM Score
0
4
8
12
0.25
0.50
0.75
Dmel\CG17776
Dmel\CG44303
U3JAY9
X1WEL3
Dmel\CG15130
Dmel\CG14380
Dmel\CG16762
COA3
Tmem196
Mul1
Snapin
Vamp5
Q6D
Brms1
DCTN3-p24
Dysb
X1WH15
Dmel\CG13000
si:dkey-33c3.1
X1WFF5
U3JAN3
X1WBE9
X1WF05
CHOp24
Nrg4
Dmel\CG9570
CBY1
Dmel\CG12112
Dmel\CG13116
Dctn3
KNSTRN
U3JAF4
ND-B16.6
Dmel\CG44574
X1WBB2
si:ch211-284f22.3
LURAP
si:dkey-248g15.2
X1WFQ3
Dmel\CG12689
X1WD28
Speer3
V9GXS8
X1WCN0
X1WFA6
BORCS6
Homer1
X1WHP2
U3JAD7
Vstm4
X1WFU5
Pex11a
NOSIP
X1WBH5
X1WE70
Spint2
X1WD87
X1WCD1
TNFSF13B
Dmel\CG4194
Cst9
CG15891
Dmel\CG32459
TMA7
X1WEE0
Dmel\CG14631
U3JAK8
Icos
Osi6
X1WC16
U3JAU9
Alsin2
Tmhs
Klrc2
X1WFU4
MCUB
X1WF92
X1WE48
X1WH96
TRIM17
si:ch211-282b22.1
si:ch73-264p11.4
Krt27
Mycs
Dmel\CG1998
Pex3
B4galt2
eIF4E7
Dmel\CG6664
X1WGV1
Gr59f
X1WGY1
CSN6
CACNG2
ttm2
ftr42
NOP53
X1WHC5
X1WDI8
TSC22D4
Bscl2
X1WCG6
cert
Gr58c
Dmel\CG4198
RhoGAP16F
Dmel\CG8578
GJA3
Crlf3
Stxbp4
SYNC
RIC-3
cyr
X1WCL6
X1WEV9
X1WBR1
R4GDR5

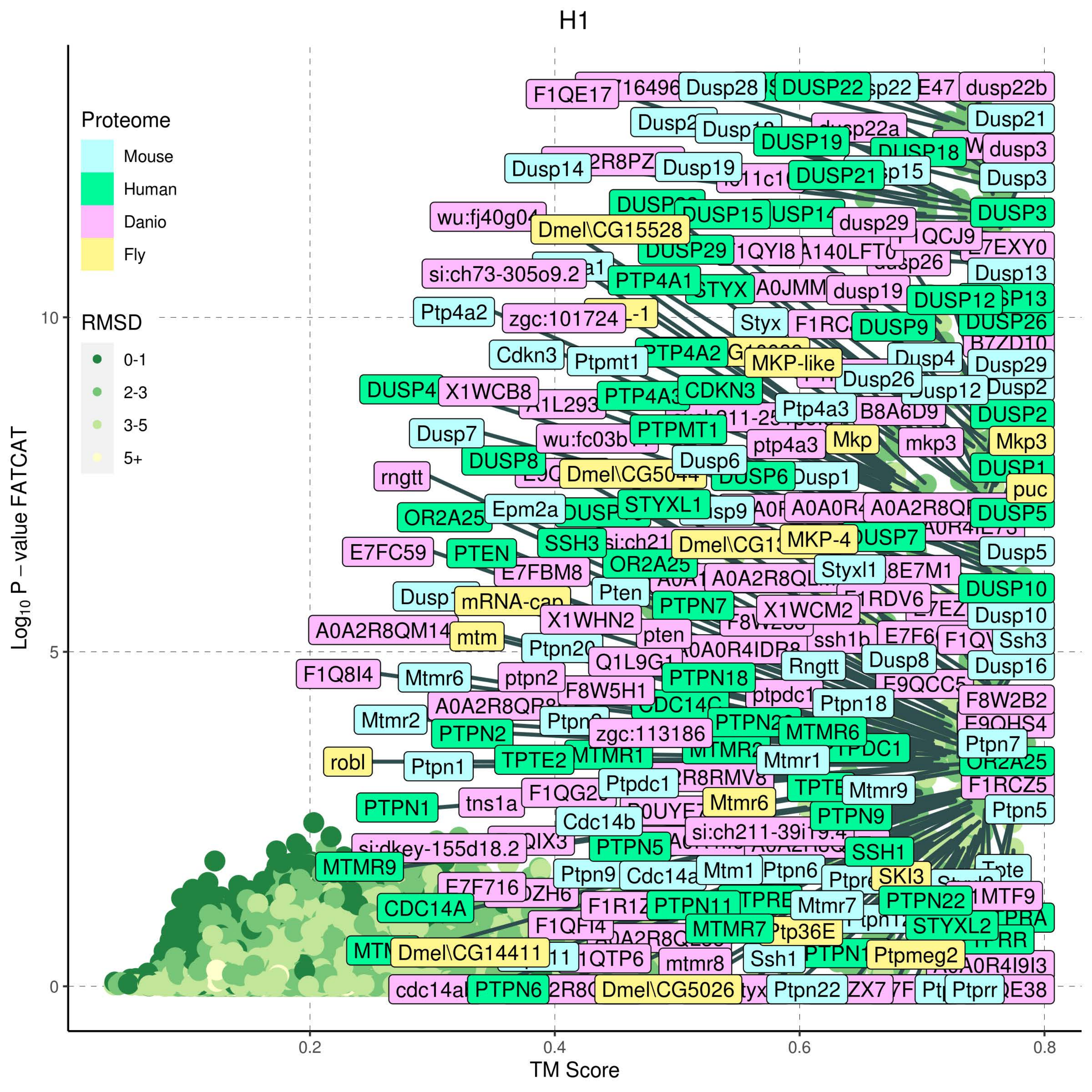
H1
Proteome
Mouse
Human
Danio
Fly
RMSD
0-1
2-3
3-5
5+
Log10 P − value FATCAT
TM Score
0
5
10
0.2
0.4
0.6
0.8
F1QE17
Dusp28
DUSP22
dusp22b
Dusp21
dusp3
Dusp3
DUSP3
Dusp14
Dusp19
DUSP19
DUSP21
DUSP15
dusp29
wu:fj40g04
Dmel\CG15528
DUSP29
STYX
Dusp13
si:ch73-305o9.2
PTP4A1
dusp19
DUSP12
DUSP26
Ptp4a2
zgc:101724
Styx
DUSP9
Cdkn3
Ptpmt1
PTP4A2
MKP-like
Dusp4
Dusp29
DUSP4
X1WCB8
CDKN3
Dusp26
Dusp12
Ptp4a3
B8A6D9
DUSP2
Dusp7
PTPMT1
ptp4a3
Mkp
mkp3
Mkp3
DUSP8
Dusp6
Dusp1
rngtt
DUSP6
puc
OR2A25
Epm2a
STYXL1
DUSP5
E7FC59
PTEN
SSH3
MKP-4
Dusp5
OR2A25
E7FBM8
Styxl1
Pten
PTPN7
DUSP10
X1WHN2
X1WCM2
Dusp10
A0A2R8QM14
mtm
pten
ssh1b
Ssh3
Ptpn20
Rngtt
Dusp8
Dusp16
F1Q8I4
Mtmr6
ptpn2
F8W5H1
PTPN18
Ptpn18
F8W2B2
Mtmr2
PTPN2
zgc:113186
MTMR6
Ptpn7
robl
Ptpn1
TPTE2
MTMR1
Mtmr1
Ptpdc1
Mtmr9
F1RCZ5
PTPN1
tns1a
Mtmr6
PTPN9
Ptpn5
Cdc14b
si:dkey-155d18.2
PTPN5
SSH1
MTMR9
Ptpn9
Cdc14a
Mtm1
Ptpn6
SKI3
E7F716
CDC14A
PTPN11
Mtmr7
PTPN22
STYXL2
MTMR7
Ptp36E
Dmel\CG14411
mtmr8
Ssh1
Ptpmeg2
cdc14ab
PTPN6
Dmel\CG5026
Ptpn22
Ptprr

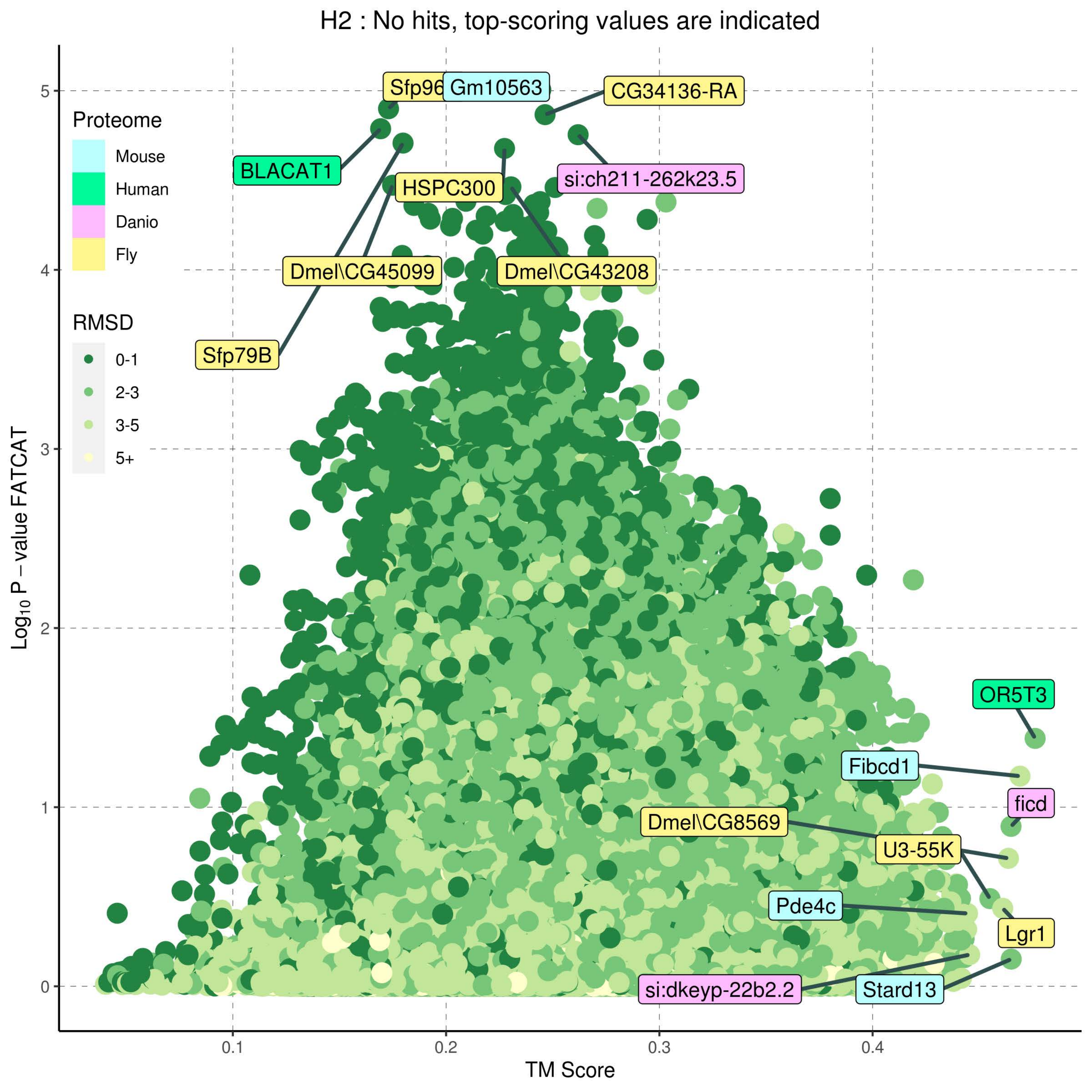

H2 : No hits, top-scoring values are indicated
Proteome
Mouse
Human
Danio
Fly
RMSD
0-1
2-3
3-5
5+
Sfp96
Gm10563
CG34136-RA
BLACAT1
si:ch211-262k23.5
HSPC300
Dmel\CG45099
Dmel\CG43208
Sfp79B
OR5T3
Fibcd1
ficd
Dmel\CG8569
U3-55K
Pde4c
Lgr1
si:dkeyp-22b2.2
Stard13
Log_{10} P – value FATCAT
TM Score
0
1
2
3
4
5
0.1
0.2
0.3
0.4

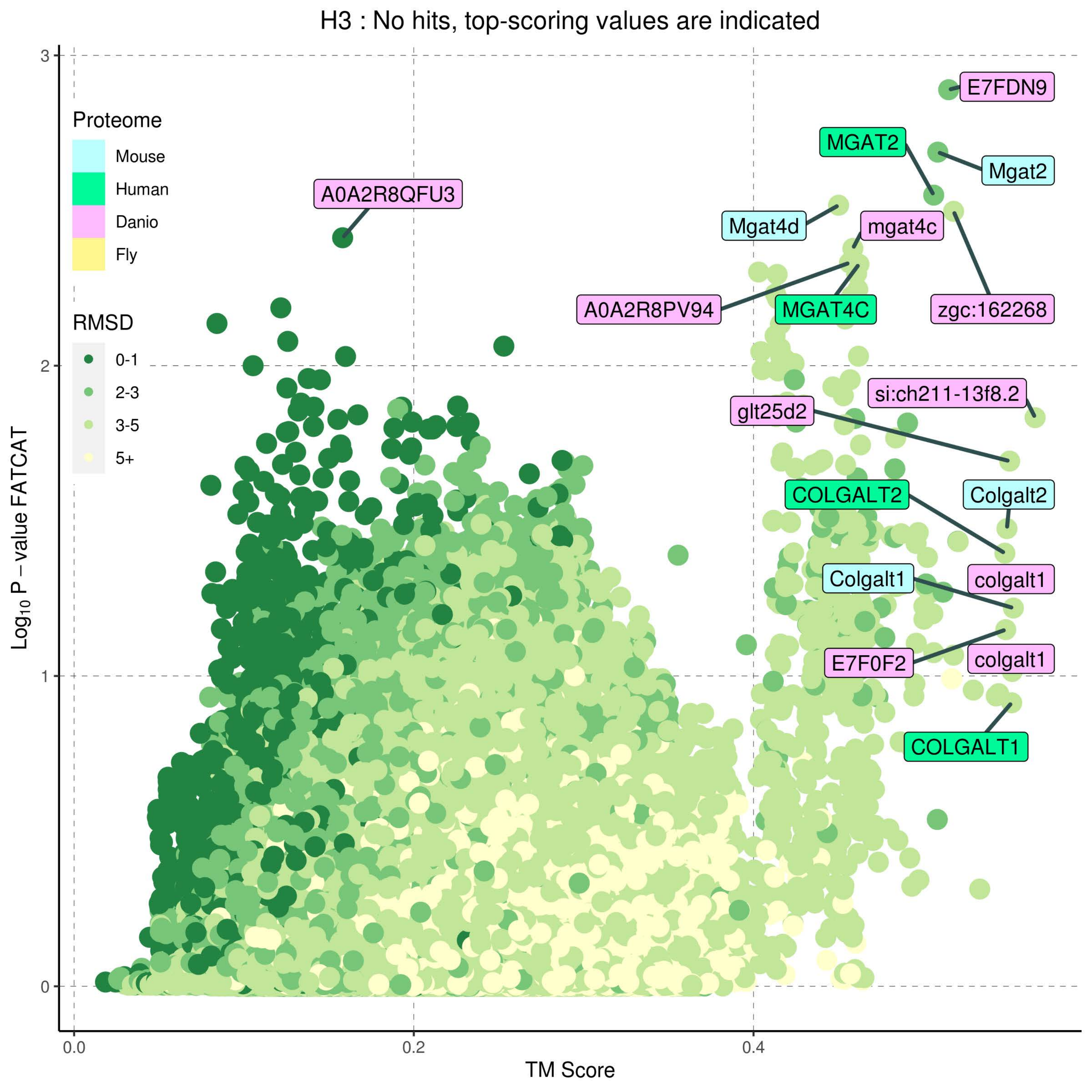
H3 : No hits, top-scoring values are indicated
Proteome
Mouse
Human
Danio
Fly
RMSD
0-1
2-3
3-5
5+
E7FDN9
MGAT2
Mgat2
A0A2R8QFU3
Mgat4d
mgat4c
A0A2R8PV94
MGAT4C
zgc:162268
si:ch211-13f8.2
glt25d2
COLGALT2
Colgalt2
Colgalt1
colgalt1
E7F0F2
colgalt1
COLGALT1
Log_{10} P – value FATCAT
TM Score
0
1
2
3
0.0
0.2
0.4

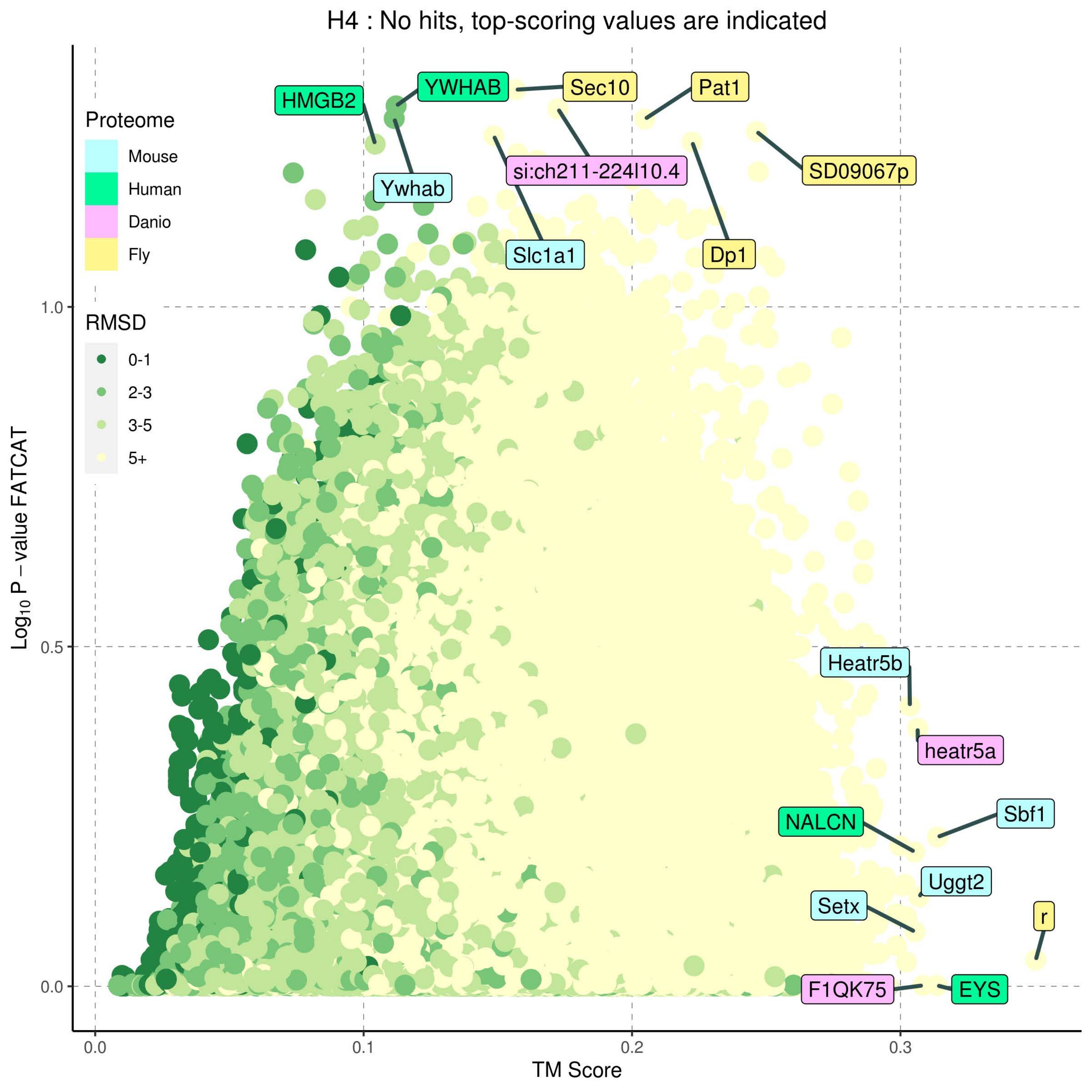

H4 : No hits, top-scoring values are indicated
Proteome
Mouse
Human
Danio
Fly
RMSD
0-1
2-3
3-5
5+
HMGB2
YWHAB
Sec10
Pat1
SD09067p
si:ch211-224l10.4
Ywhab
Slc1a1
Dp1
Heatr5b
heatr5a
Sbf1
NALCN
Uggt2
Setx
r
F1QK75
EYS
1.0
0.5
0.0
0.1
0.2
0.3
Log10 P − value FATCAT
TM Score

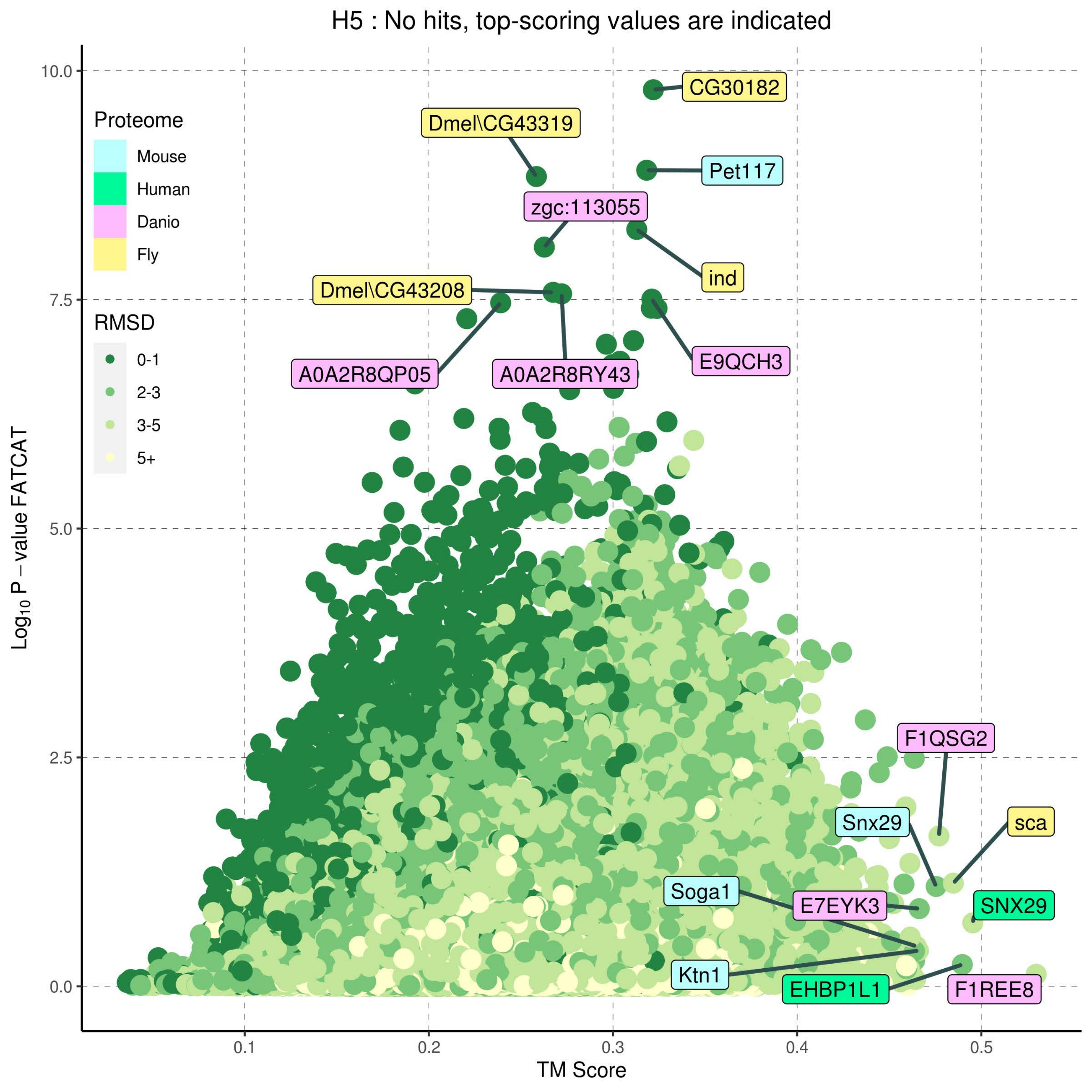

H5 : No hits, top-scoring values are indicated
Proteome
Mouse
Human
Danio
Fly
RMSD
0-1
2-3
3-5
5+
CG30182
Dmel\CG43319
Pet117
zgc:113055
ind
Dmel\CG43208
E9QCH3
A0A2R8QP05
A0A2R8RY43
F1QSG2
Snx29
sca
Soga1
E7EYK3
SNX29
Ktn1
EHBP1L1
F1REE8
10.0
7.5
5.0
2.5
0.0
0.1
0.2
0.3
0.4
0.5
Log_{10} P – value FATCAT
TM Score

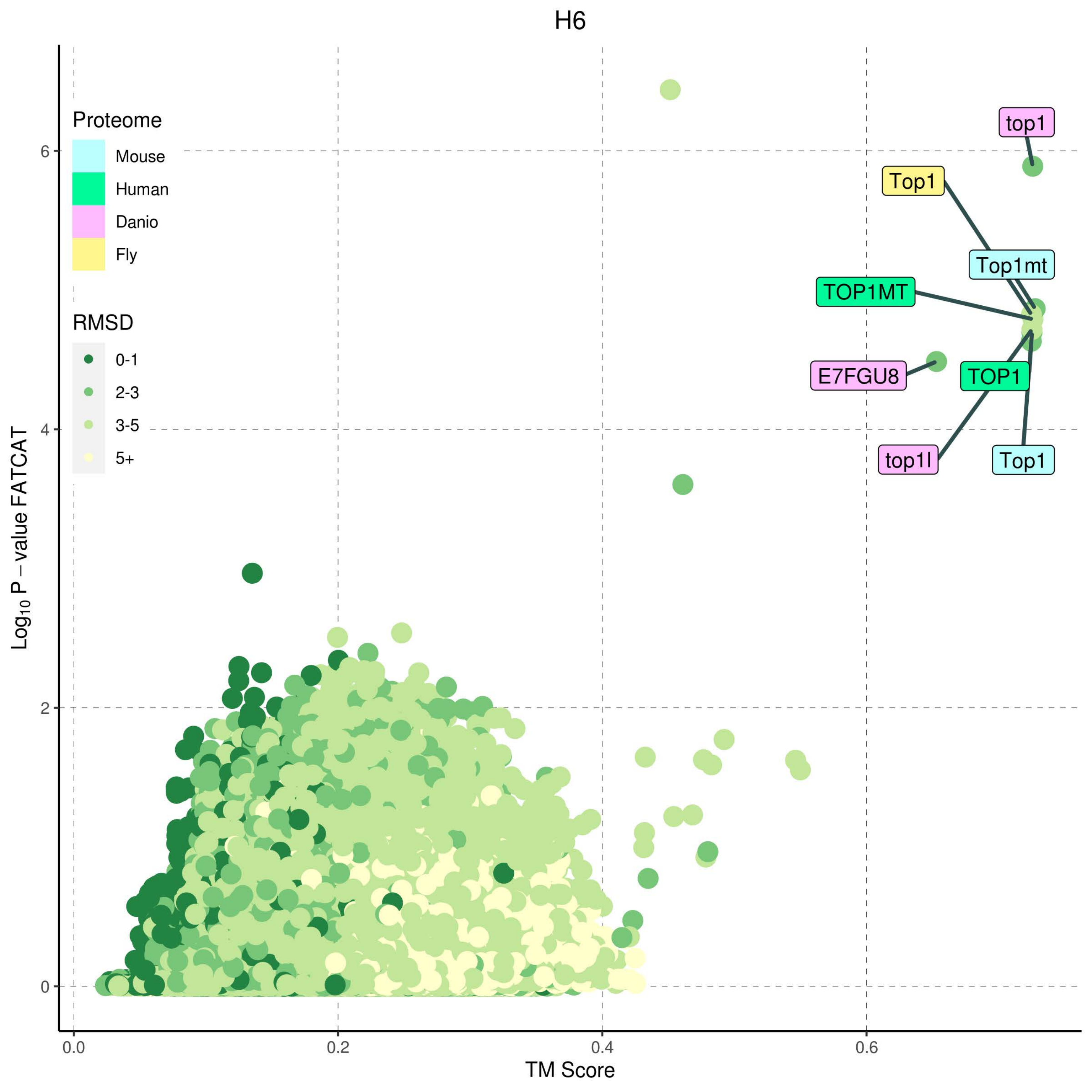

H6
Proteome
Mouse
Human
Danio
Fly
RMSD
0-1
2-3
3-5
5+
top1
Top1
Top1mt
TOP1MT
E7FGU8
TOP1
top1l
Top1
Log_{10} P − value FATCAT
TM Score
0
2
4
6
0.0
0.2
0.4
0.6

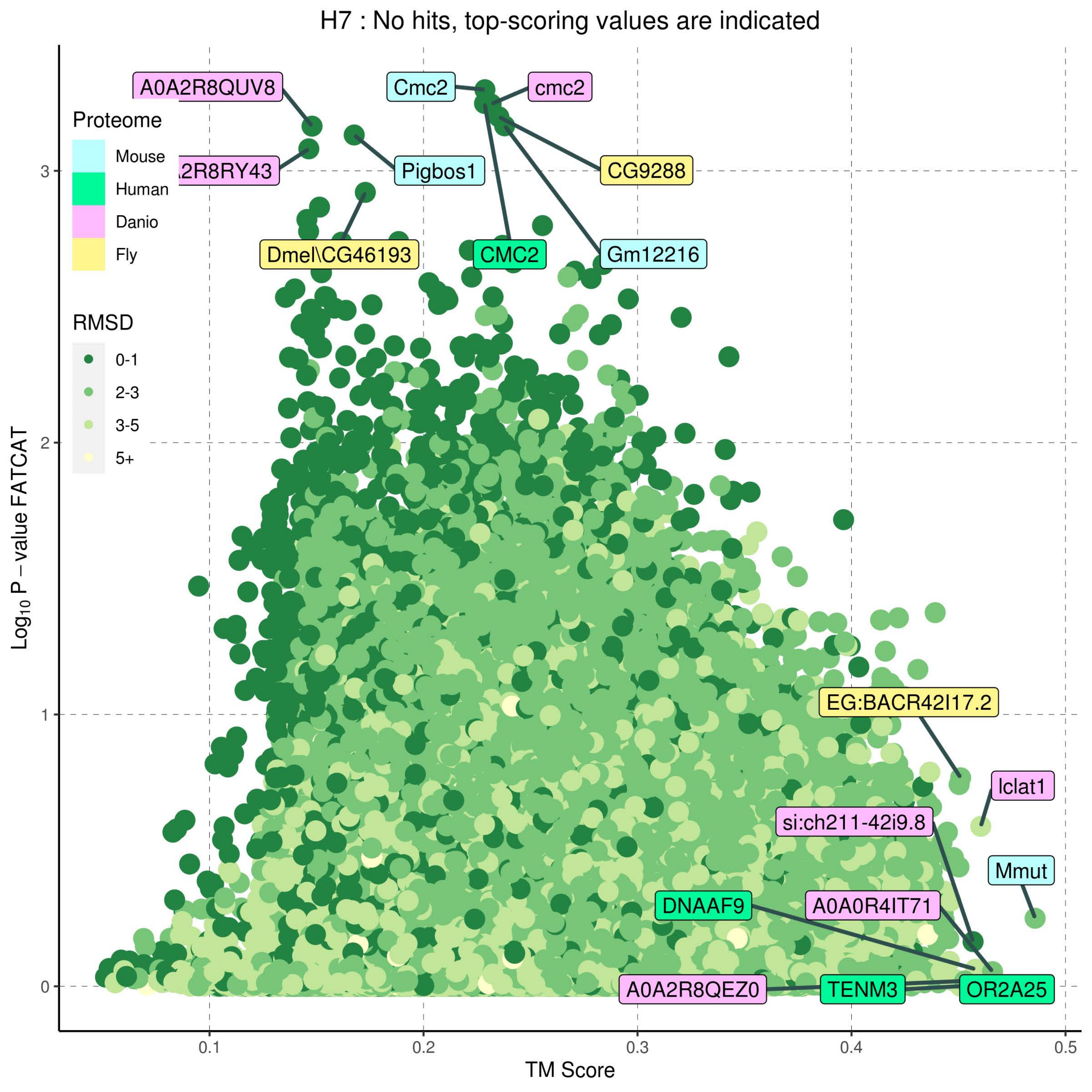
H7 : No hits, top-scoring values are indicated
Proteome
Mouse
Human
Danio
Fly
RMSD
0-1
2-3
3-5
5+
A0A2R8QUV8
Cmc2
cmc2
Pigbos1
CG9288
Dmel\CG46193
CMC2
Gm12216
EG:BACR42I17.2
lclat1
si:ch211-42i9.8
Mmut
DNAAF9
A0A0R4IT71
A0A2R8QEZ0
TENM3
OR2A25
Log10 P − value FATCAT
TM Score
0
1
2
3
0.1
0.2
0.3
0.4
0.5

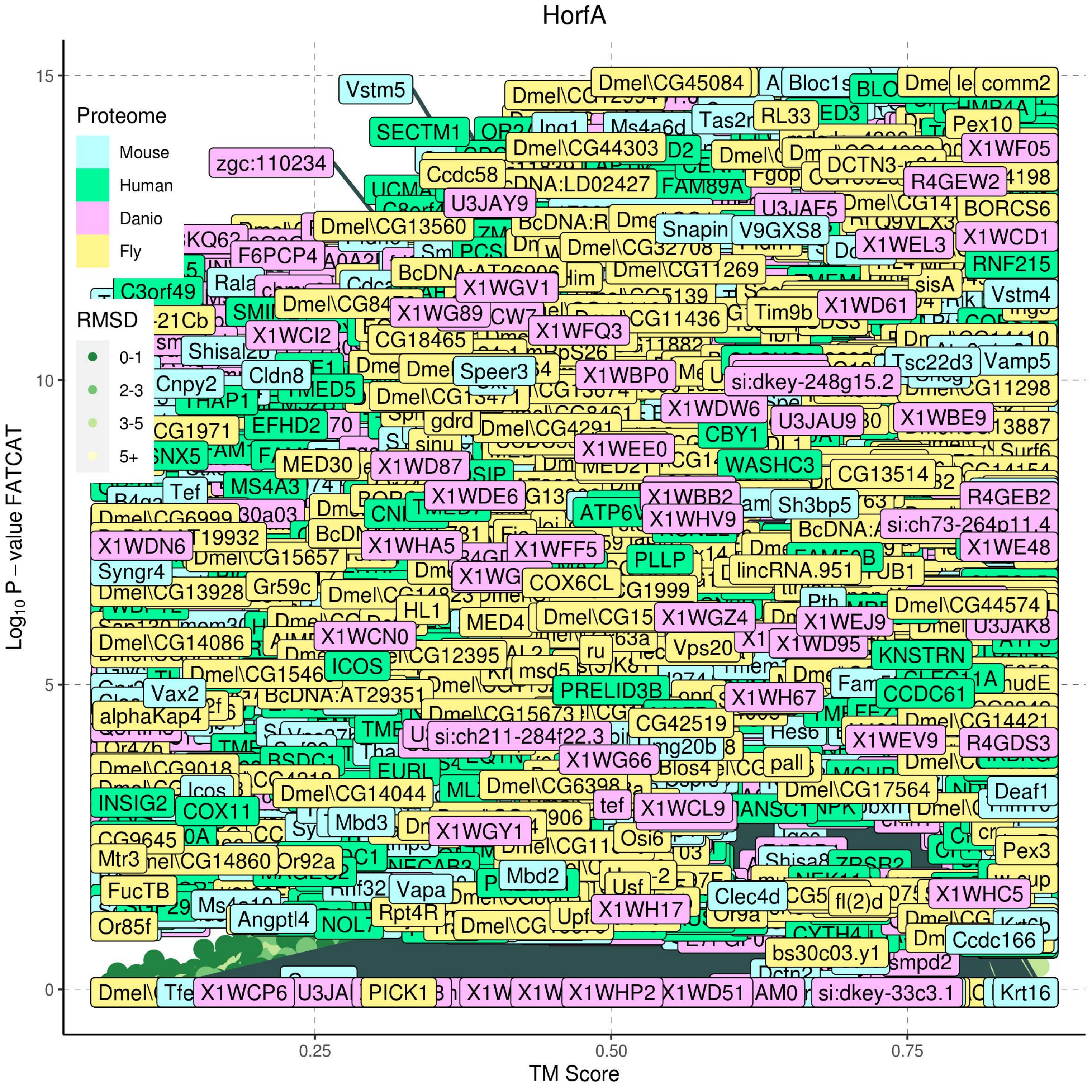

HorfA
Proteome
Mouse
Human
Danio
Fly
RMSD
0-1
2-3
3-5
5+
Log10 P − value FATCAT
TM Score
0
5
10
15
0.25
0.50
0.75
Vstm5
Dmel\CG45084
comm2
SECTM1
RL33
Pex10
zgc:110234
Dmel\CG44303
X1WF05
Ccdc58
FAM89A
R4GEW2
U3JAY9
U3JAF5
BORCS6
Dmel\CG13560
Snapin
V9GXS8
X1WEL3
X1WCD1
F6PCP4
Dmel\CG32708
Dmel\CG11269
RNF215
C3orf49
Rala
X1WGV1
sisA
Vstm4
X1WG89
X1WD61
Tim9b
Dmel\CG11436
X1WCI2
X1WFQ3
CG18465
Shisal2b
Tsc22d3
Vamp5
Cldn8
Speer3
X1WBP0
si:dkey-248g15.2
Cnpy2
TMED5
Dmel\CG11298
THAP1
X1WDW6
gdrd
EFHD2
U3JAU9
X1WBE9
CG1971
CBY1
SNX5
X1WEE0
MED30
X1WD87
WASHC3
CG13514
MS4A3
Tef
X1WDE6
R4GEB2
X1WBB2
Sh3bp5
X1WHV9
si:ch73-264p11.4
X1WDN6
X1WHA5
X1WFF5
X1WE48
Dmel\CG15657
PLLP
lincRNA.951
Syngr4
COX6CL
Dmel\CG13928
Gr59c
Dmel\CG44574
HL1
MED4
X1WGZ4
X1WEJ9
U3JAK8
Dmel\CG14086
X1WCN0
X1WD95
KNSTRN
Vps20
ICOS
msd5
Vax2
PRELID3B
X1WH67
CCDC61
BcDNA:AT29351
alphaKap4
CG42519
Dmel\CG14421
si:ch211-284f22.3
X1WEV9
R4GDS3
X1WG66
pall
Blos4
INSIG2
Dmel\CG14044
Dmel\CG6398
Dmel\CG17564
Deaf1
COX11
tef
X1WCL9
Mbd3
X1WGY1
Osi6
Pex3
Mtr3
Or92a
Mbd2
X1WHC5
FucTB
Vapa
Clec4d
fl(2)d
Angptl4
Rpt4R
X1WH17
Or85f
Ccdc166
bs30c03.y1
smpd2
X1WCP6
PICK1
X1WHP2
X1WD51
si:dkey-33c3.1
Krt16

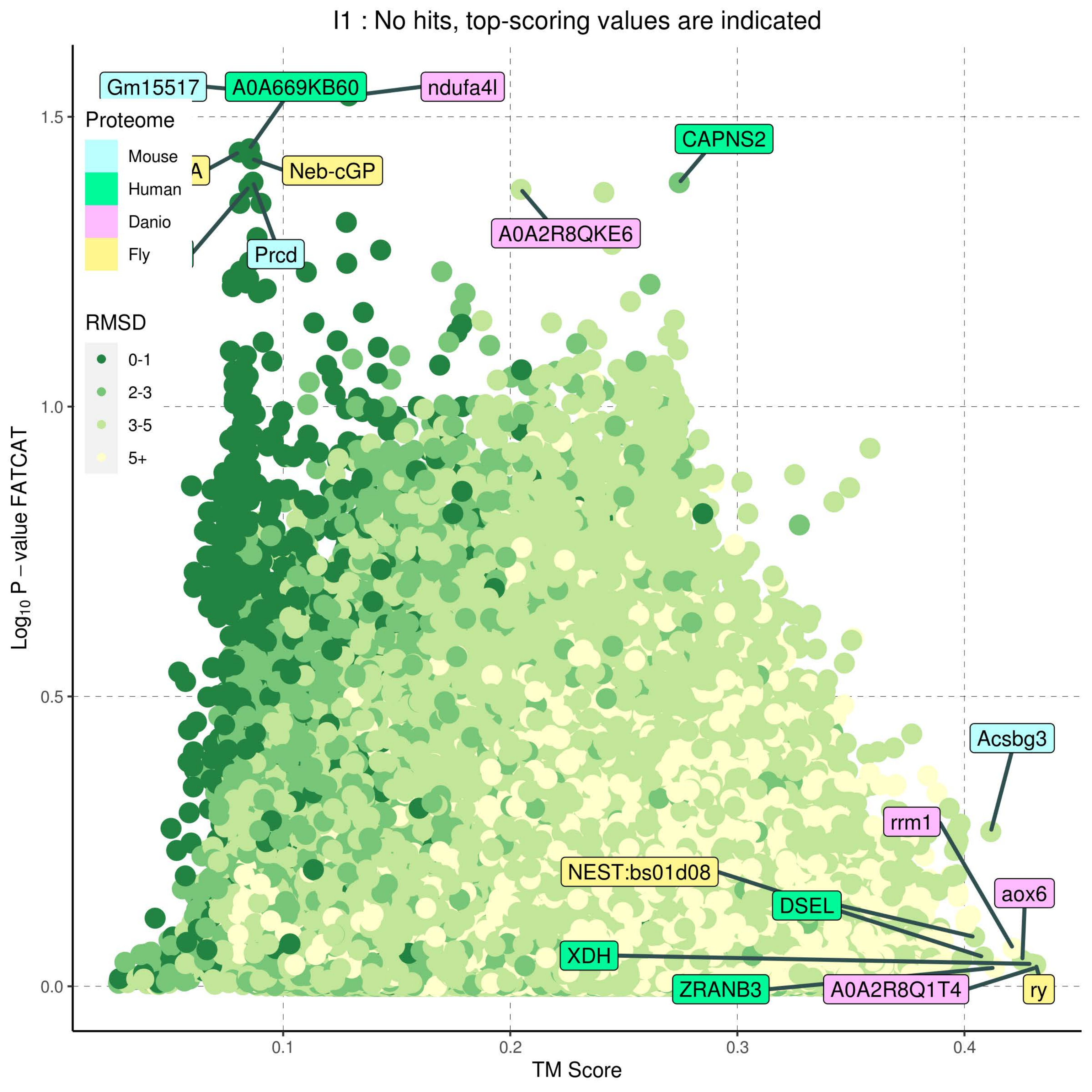
I1 : No hits, top-scoring values are indicated
Log10 P − value FATCAT
TM Score
0.0
0.5
1.0
1.5
0.1
0.2
0.3
0.4
Proteome
Mouse
Human
Danio
Fly
RMSD
0-1
2-3
3-5
5+
Gm15517
A0A669KB60
ndufa4l
Neb-cGP
Prcd
CAPNS2
A0A2R8QKE6
Acsbg3
rrm1
NEST:bs01d08
DSEL
aox6
XDH
ZRANB3
A0A2R8Q1T4
ry

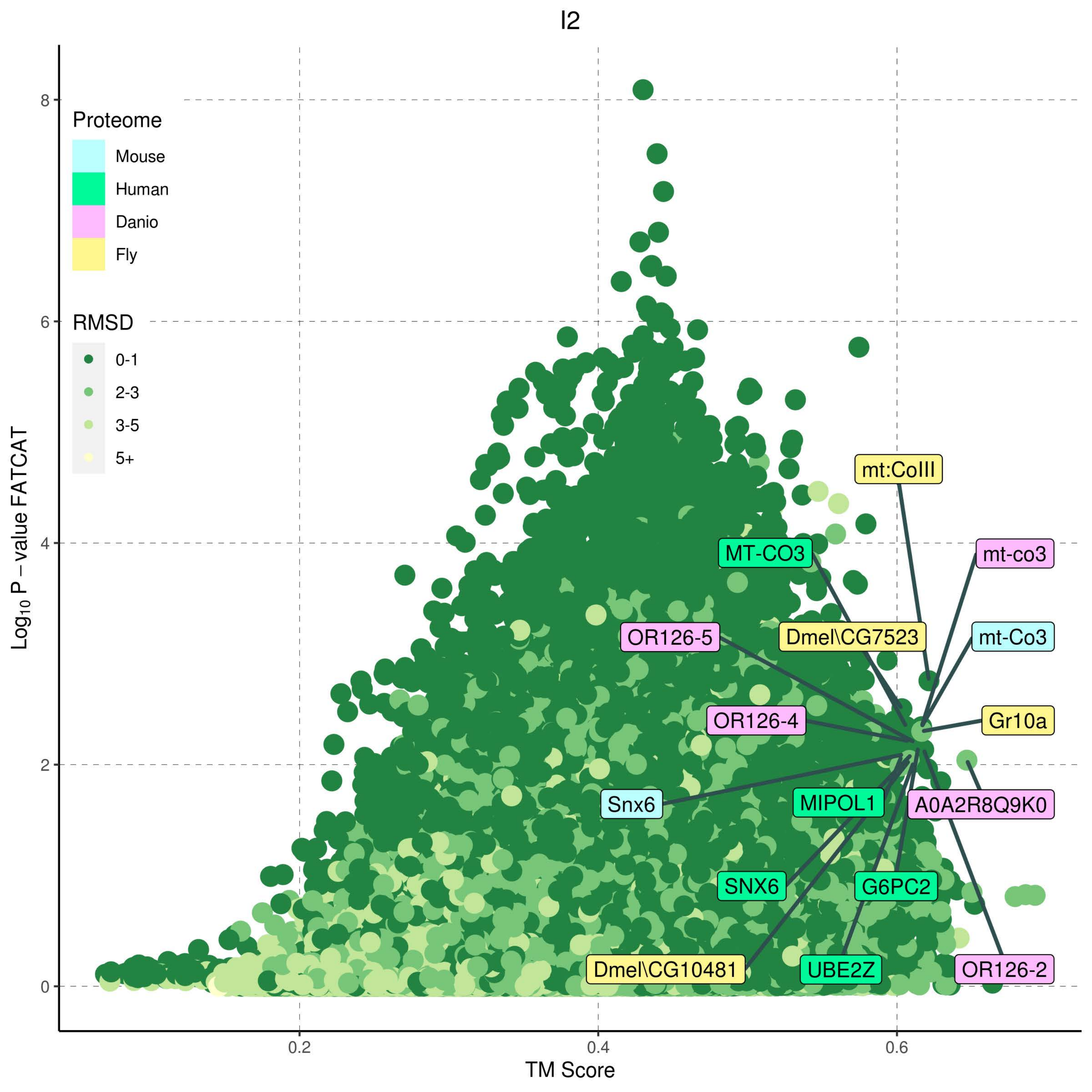

I2
Proteome
Mouse
Human
Danio
Fly
RMSD
0-1
2-3
3-5
5+
mt:CoIII
mt-co3
MT-CO3
mt-Co3
OR126-5
Dmel\CG7523
Gr10a
OR126-4
Snx6
MIPOL1
A0A2R8Q9K0
SNX6
G6PC2
Dmel\CG10481
UBE2Z
OR126-2
Log10 P – value FATCAT
TM Score
0
2
4
6
8
0.2
0.4
0.6

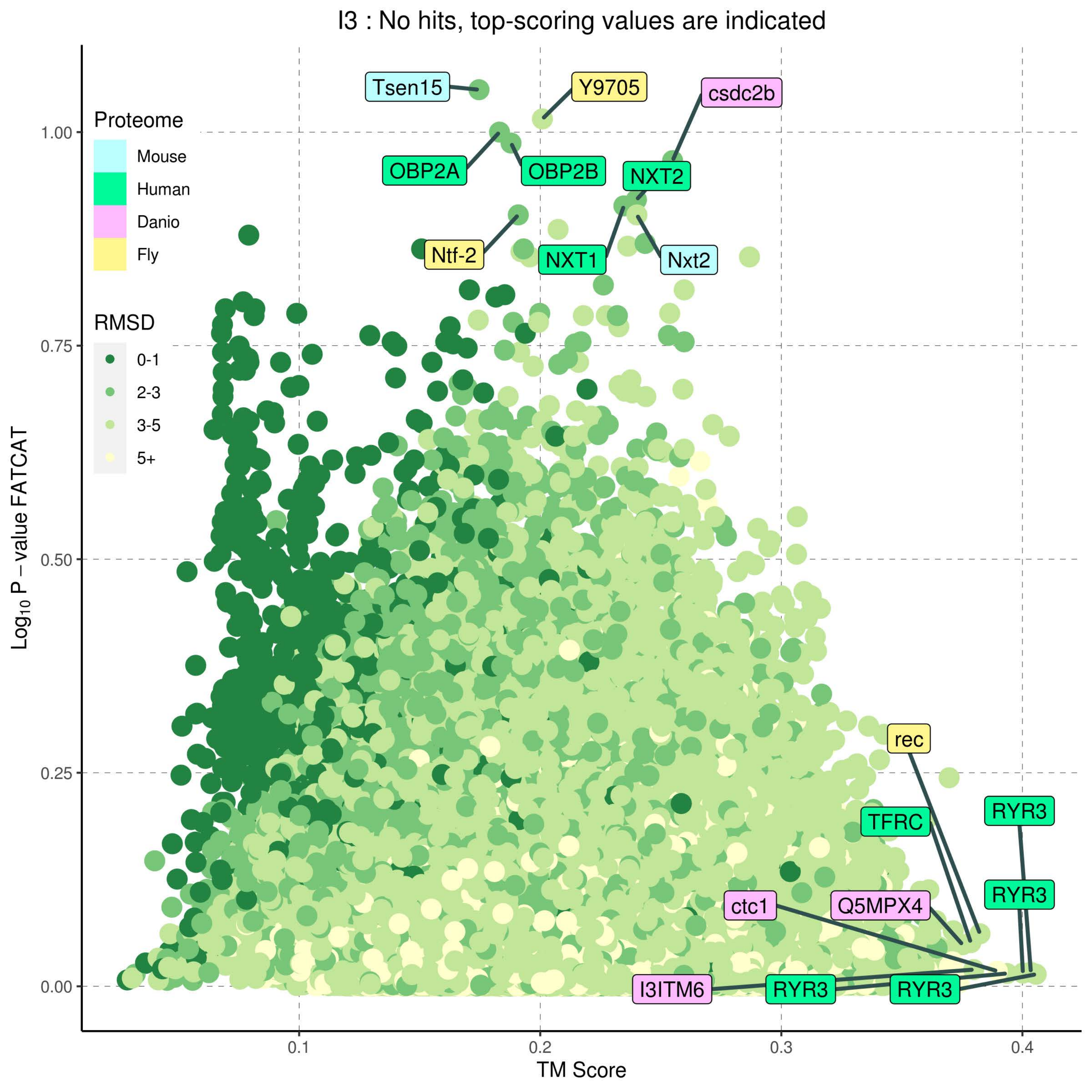

I3 : No hits, top-scoring values are indicated
Proteome
Mouse
Human
Danio
Fly
RMSD
0-1
2-3
3-5
5+
Log10 P − value FATCAT
TM Score
1.00
0.75
0.50
0.25
0.00
0.1
0.2
0.3
0.4
Tsen15
Y9705
csdc2b
OBP2A
OBP2B
NXT2
Ntf-2
NXT1
Nxt2
rec
TFRC
RYR3
RYR3
ctc1
Q5MPX4
I3ITM6
RYR3
RYR3

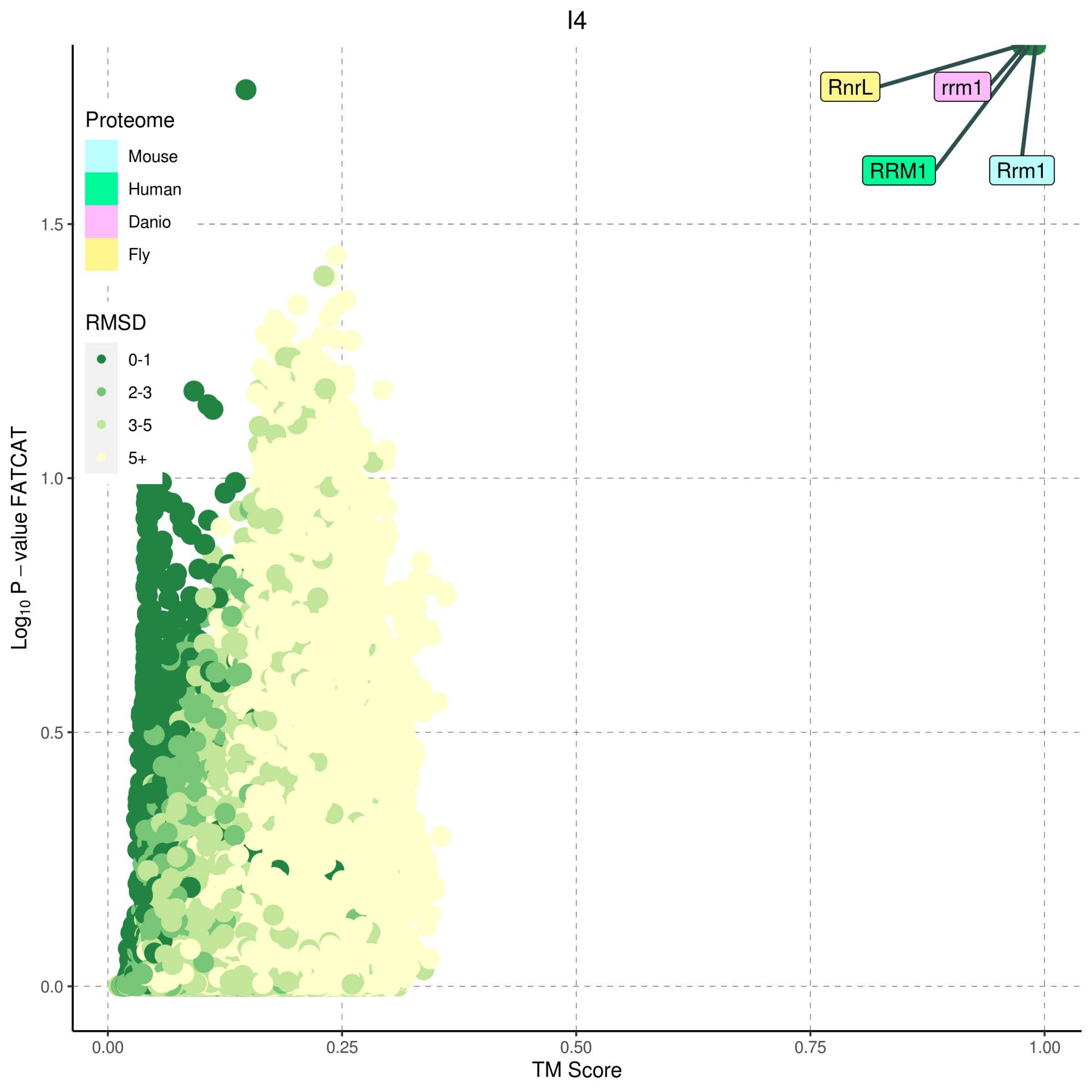

I4
RnrL
rrm1
RRM1
Rrm1
Proteome
Mouse
Human
Danio
Fly
RMSD
0-1
2-3
3-5
5+
1.5
1.0
0.5
0.0
Log10 P – value FATCAT
0.00
0.25
0.50
0.75
1.00
TM Score

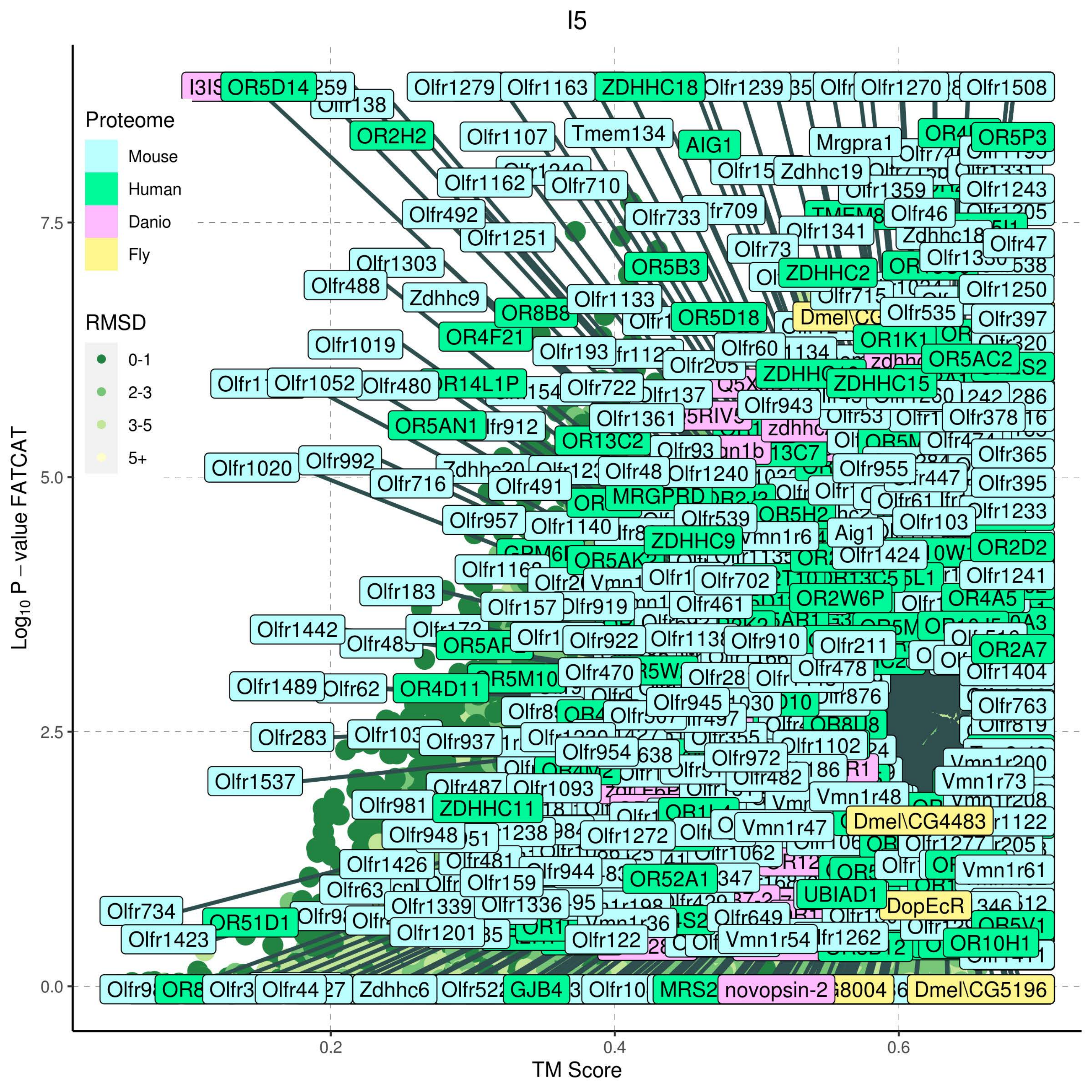

I5
Proteome
Mouse
Human
Danio
Fly
RMSD
0-1
2-3
3-5
5+
Log10 P − value FATCAT
TM Score
0.0
2.5
5.0
7.5
0.2
0.4
0.6
OR5D14
Olfr1279
Olfr1163
ZDHHC18
Olfr1239
Olfr1270
Olfr1508
Olfr138
OR2H2
Olfr1107
Tmem134
AIG1
Mrgpra1
OR5P3
Zdhhc19
Olfr1162
Olfr710
Olfr1359
Olfr1243
Olfr492
Olfr733
Olfr46
Olfr1251
Olfr1341
Olfr47
Olfr1303
Olfr73
OR5B3
ZDHHC2
Olfr488
Zdhhc9
Olfr1133
Olfr1250
OR8B8
OR5D18
Olfr535
Olfr397
OR4F21
OR1K1
Olfr1019
Olfr193
Olfr60
OR5AC2
Olfr1052
Olfr480
OR14L1P
Olfr722
Olfr205
ZDHHC15
Olfr943
Olfr378
OR5AN1
Olfr1361
Olfr53
OR13C2
Olfr93
Olfr365
Olfr1020
Olfr992
Olfr48
Olfr1240
Olfr955
Olfr447
Olfr716
Olfr491
MRGPRD
Olfr395
Olfr539
OR5H2
Olfr957
Olfr1140
Olfr103
Olfr1233
ZDHHC9
Aig1
OR2D2
Olfr1424
Olfr702
Olfr1241
Olfr183
OR2W6P
Olfr157
Olfr919
Olfr461
OR4A5
Olfr1442
Olfr922
Olfr910
Olfr211
OR2A7
Olfr470
Olfr478
Olfr1404
Olfr1489
Olfr62
OR4D11
Olfr945
Olfr876
Olfr763
Olfr283
Olfr937
Olfr954
Olfr972
Olfr1102
Olfr1537
Olfr482
Vmn1r73
Olfr1093
Vmn1r48
Olfr981
ZDHHC11
Vmn1r47
Dmel\CG4483
Olfr948
Olfr1272
Olfr1062
Olfr1426
Olfr481
Olfr944
OR52A1
Vmn1r61
Olfr159
UBIAD1
Olfr1339
Olfr1336
Olfr649
DopEcR
Olfr734
OR51D1
Olfr1201
Vmn1r36
Olfr1423
Olfr122
Vmn1r54
OR10H1
Zdhhc6
GJB4
novopsin-2
Dmel\CG5196

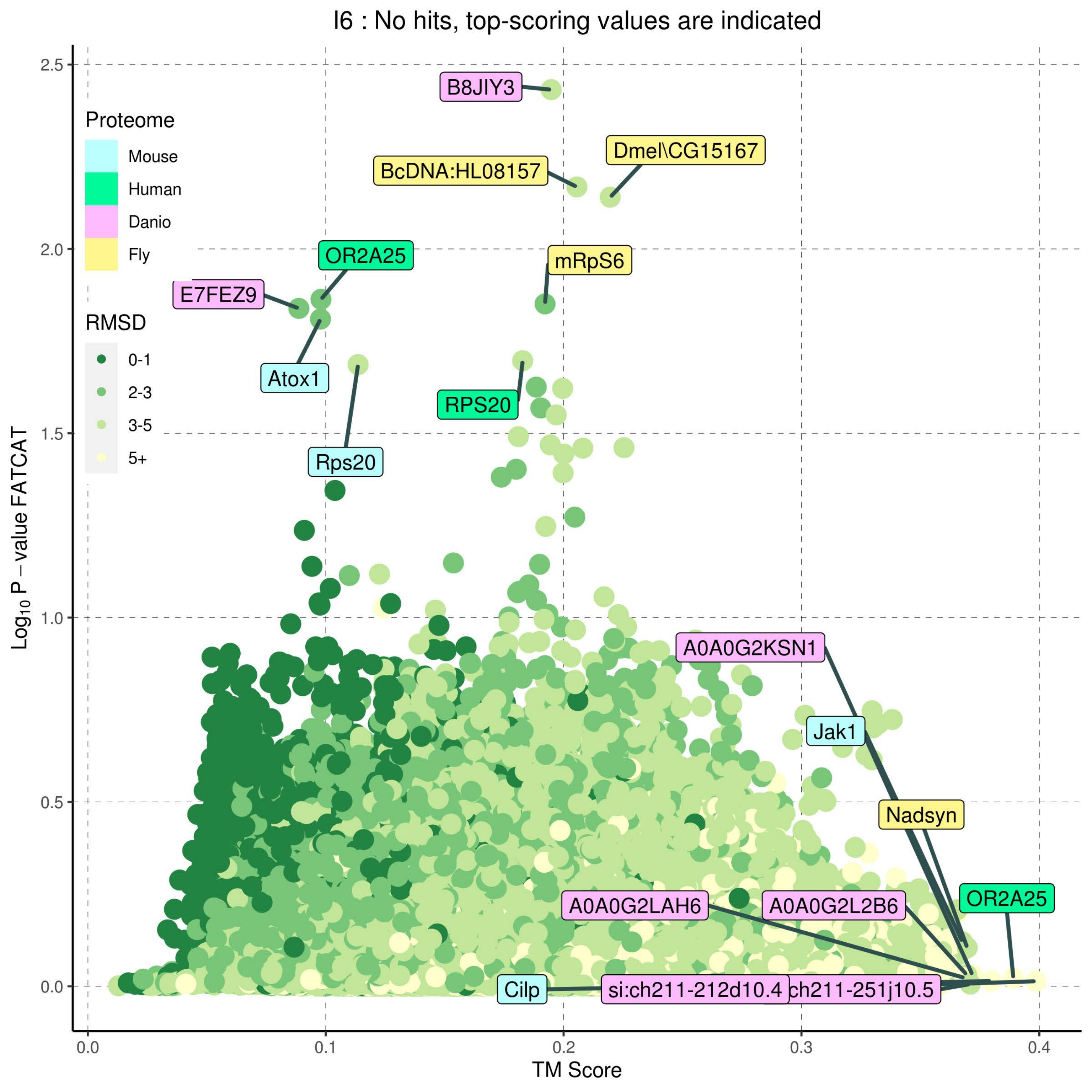

I6 : No hits, top-scoring values are indicated
Proteome
Mouse
Human
Danio
Fly
RMSD
0-1
2-3
3-5
5+
B8JIY3
Dmel\CG15167
BcDNA:HL08157
OR2A25
mRpS6
E7FEZ9
Atox1
RPS20
Rps20
A0A0G2KSN1
Jak1
Nadsyn
OR2A25
A0A0G2LAH6
A0A0G2L2B6
Cilp
si:ch211-212d10.4
ch211-251j10.5
Log10 P – value FATCAT
TM Score
0.0
0.1
0.2
0.3
0.4
0.0
0.5
1.0
1.5
2.0
2.5

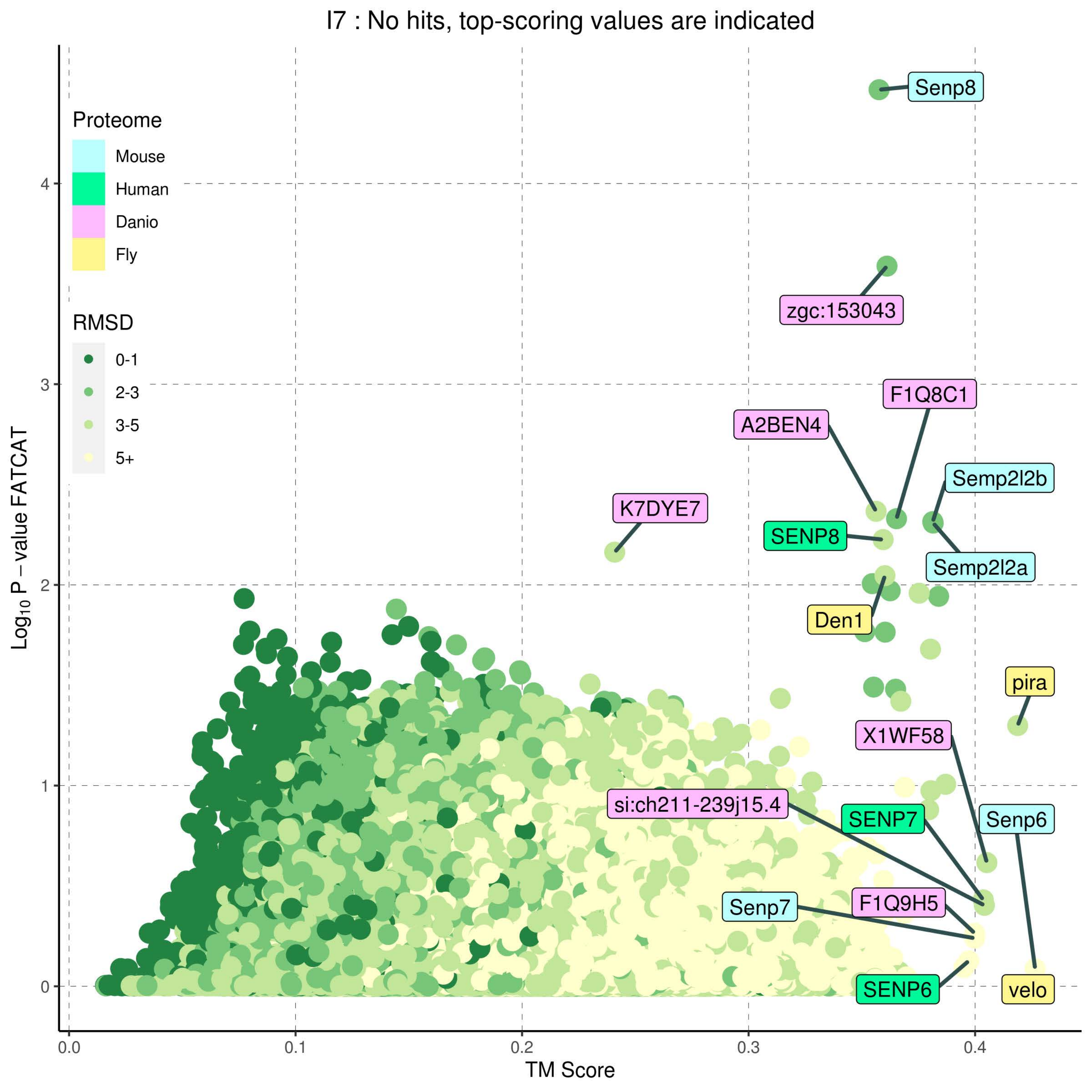

I7 : No hits, top-scoring values are indicated
Proteome
Mouse
Human
Danio
Fly
RMSD
0-1
2-3
3-5
5+
Senp8
zgc:153043
F1Q8C1
A2BEN4
Semp2l2b
K7DYE7
SENP8
Semp2l2a
Den1
pira
X1WF58
si:ch211-239j15.4
SENP7
Senp6
Senp7
F1Q9H5
SENP6
velo
Log_{10} P – value FATCAT
TM Score
0
1
2
3
4
0.0
0.1
0.2
0.3
0.4

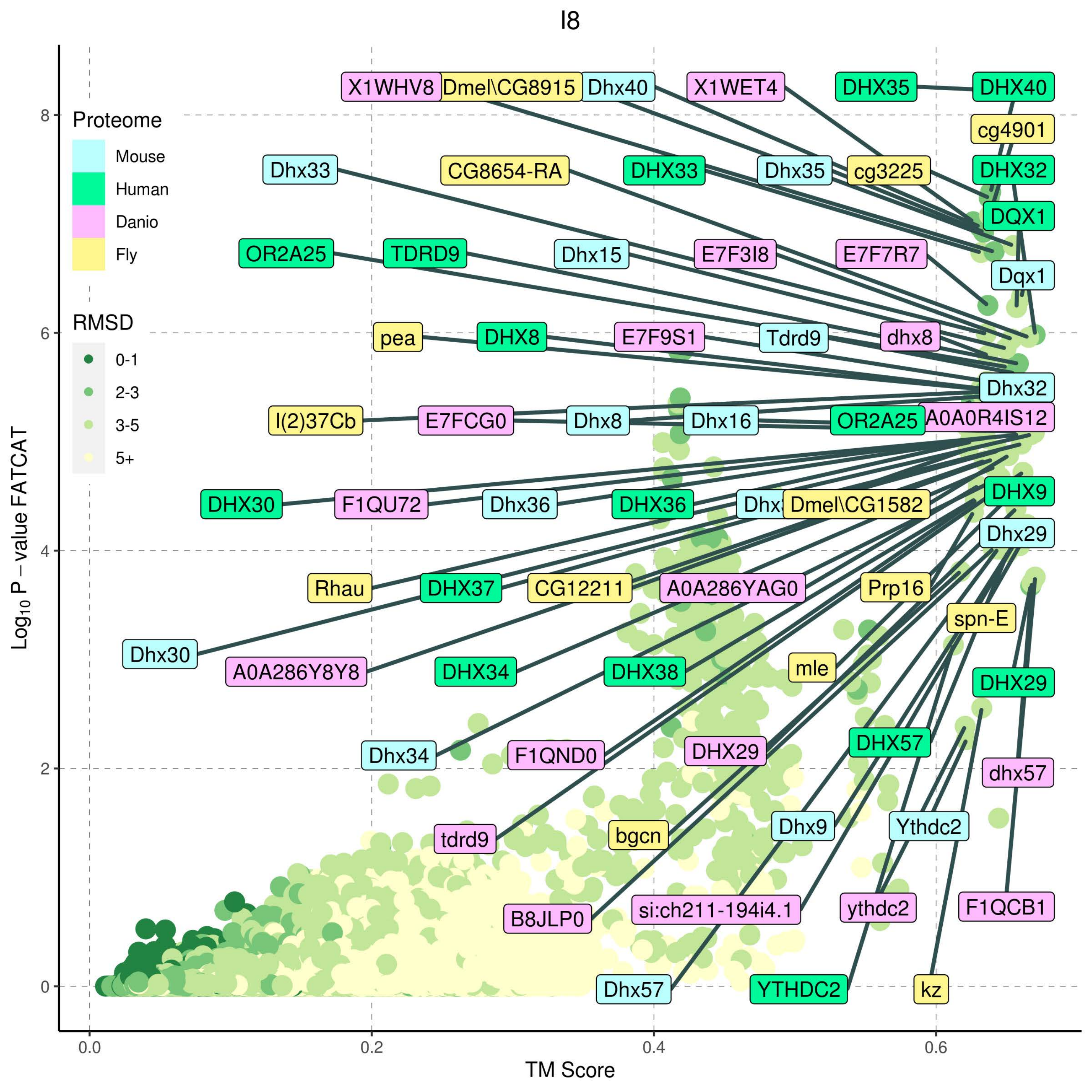
I8
Proteome
Mouse
Human
Danio
Fly
RMSD
0-1
2-3
3-5
5+
Log10 P − value FATCAT
TM Score
X1WHV8
Dmel\CG8915
Dhx40
X1WET4
DHX35
DHX40
cg4901
Dhx33
CG8654-RA
DHX33
Dhx35
cg3225
DHX32
DQX1
OR2A25
TDRD9
Dhx15
E7F3I8
E7F7R7
Dqx1
pea
DHX8
E7F9S1
Tdrd9
dhx8
Dhx32
l(2)37Cb
E7FCG0
Dhx8
Dhx16
OR2A25
A0A0R4IS12
DHX30
F1QU72
Dhx36
DHX36
Dmel\CG1582
DHX9
Dhx29
Rhau
DHX37
CG12211
A0A286YAG0
Prp16
spn-E
Dhx30
A0A286Y8Y8
DHX34
DHX38
mle
DHX29
Dhx34
F1QND0
DHX29
DHX57
dhx57
tdrd9
bgcn
Dhx9
Ythdc2
B8JLP0
si:ch211-194i4.1
ythdc2
F1QCB1
Dhx57
YTHDC2
kz

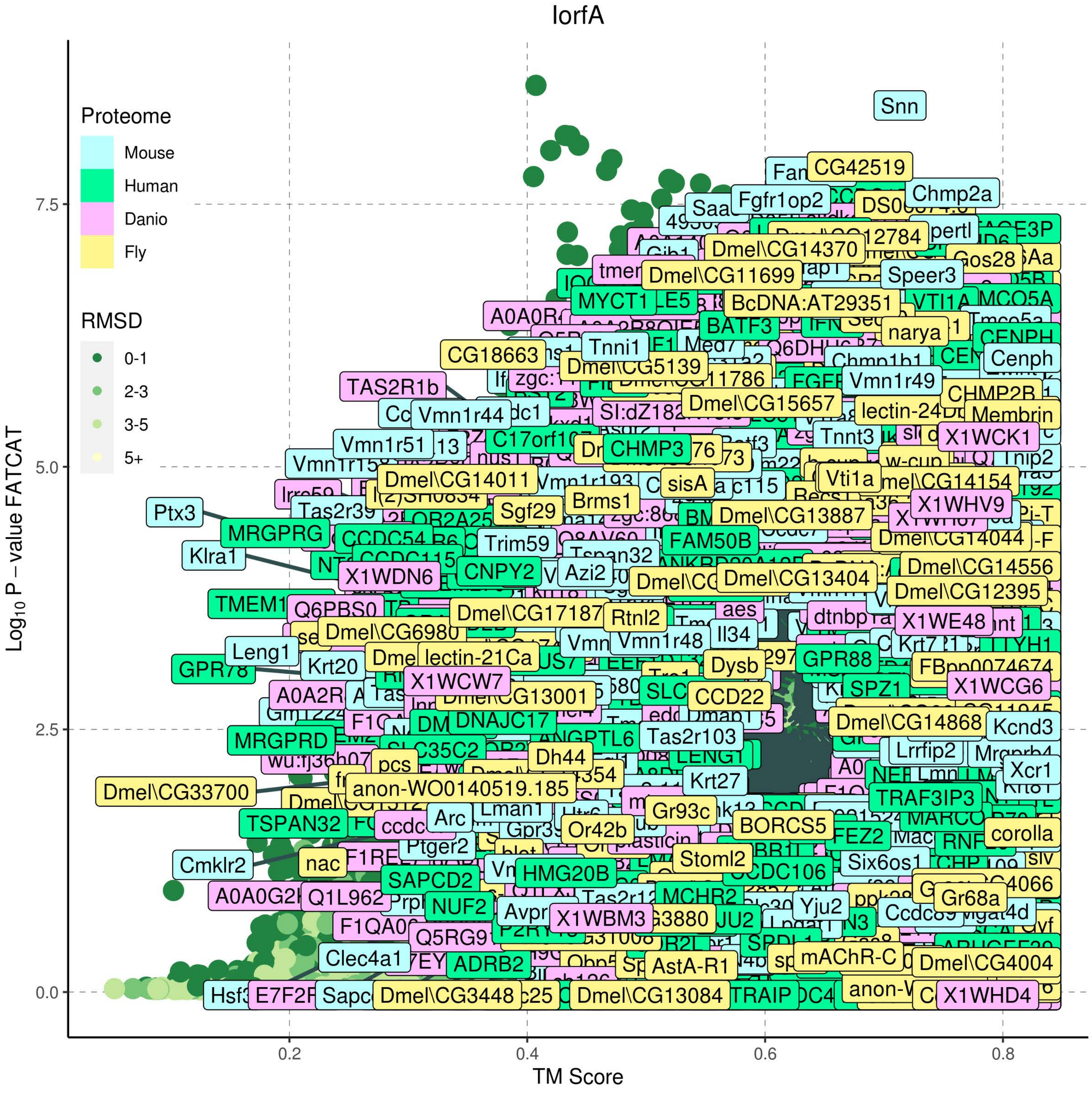

lorfA
Proteome
Mouse
Human
Danio
Fly
RMSD
0-1
2-3
3-5
5+
7.5
5.0
2.5
0.0
Log_{10} P − value FATCAT
0.2
0.4
0.6
0.8
TM Score

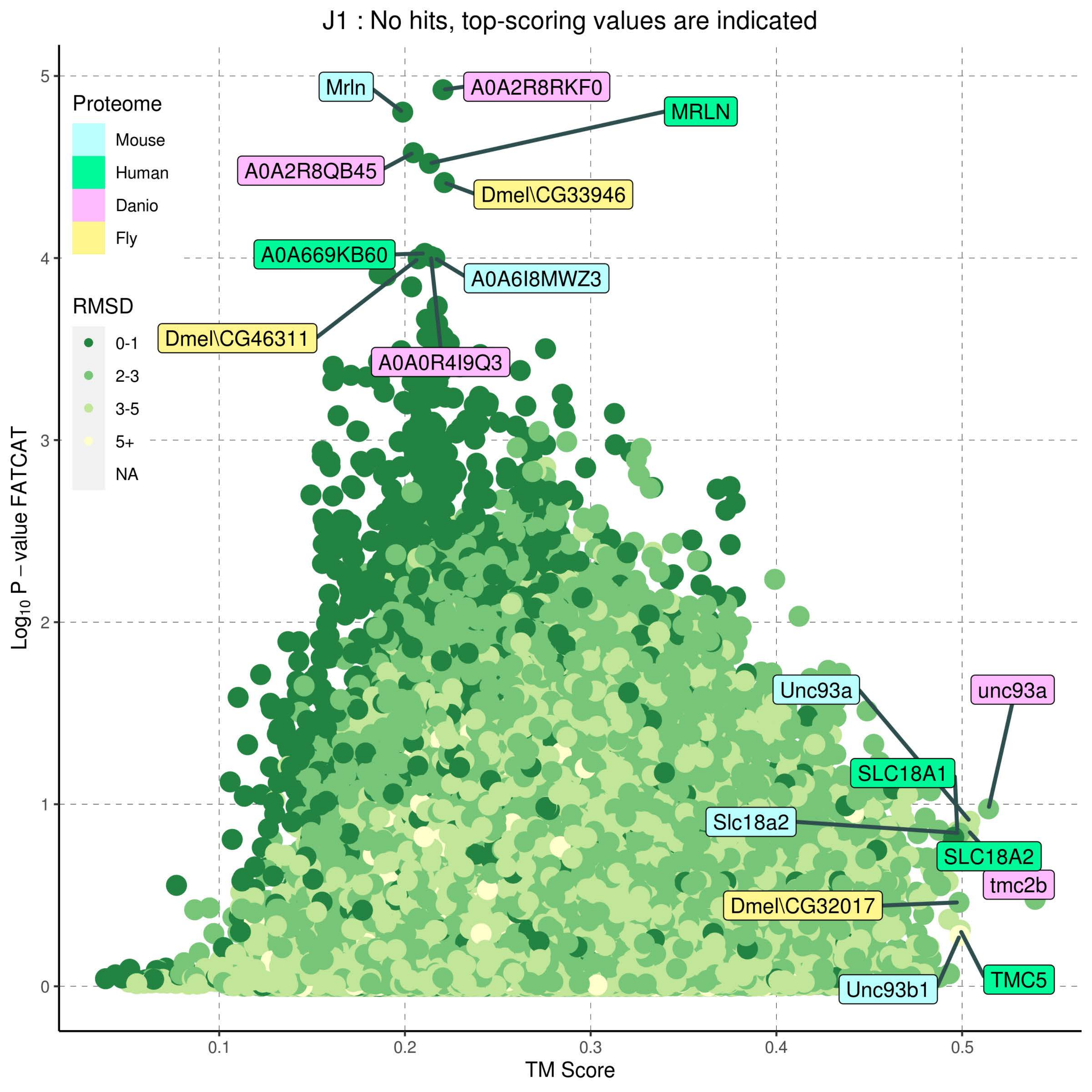
J1 : No hits, top-scoring values are indicated
Proteome
Mouse
Human
Danio
Fly
RMSD
0-1
2-3
3-5
5+
NA
Mrln
A0A2R8RKF0
MRLN
A0A2R8QB45
Dmel\CG33946
A0A669KB60
A0A6I8MWZ3
Dmel\CG46311
A0A0R4I9Q3
Unc93a
unc93a
SLC18A1
Slc18a2
SLC18A2
tmc2b
Dmel\CG32017
TMC5
Unc93b1
Log10 P − value FATCAT
TM Score
0
1
2
3
4
5
0.1
0.2
0.3
0.4
0.5

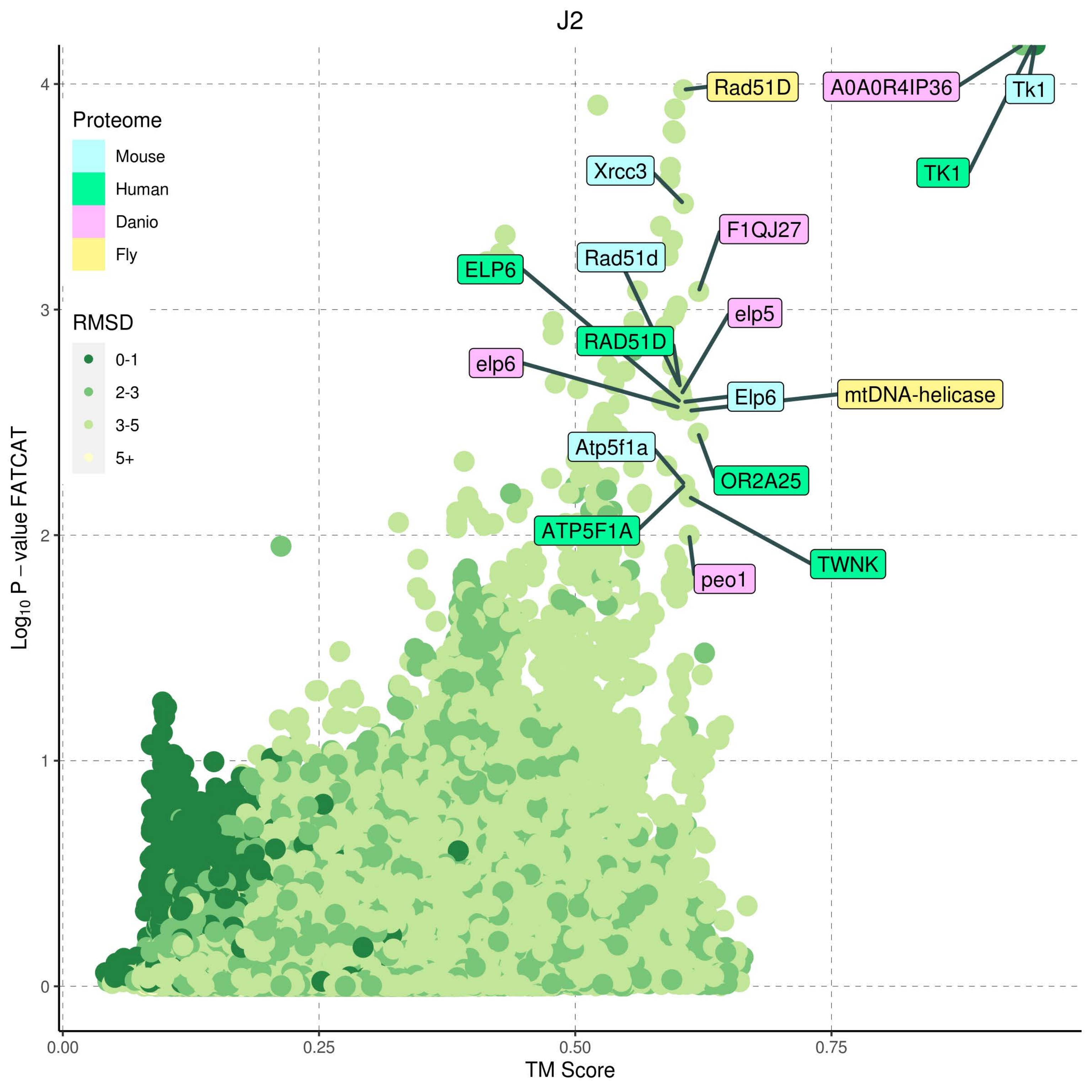
J2
Proteome
Mouse
Human
Danio
Fly
RMSD
0-1
2-3
3-5
5+
Rad51D
A0A0R4IP36
Tk1
TK1
Xrcc3
F1QJ27
Rad51d
ELP6
elp5
RAD51D
elp6
Elp6
mtDNA-helicase
Atp5f1a
OR2A25
ATP5F1A
TWNK
peo1
Log10 P − value FATCAT
TM Score
0
1
2
3
4
0.00
0.25
0.50
0.75

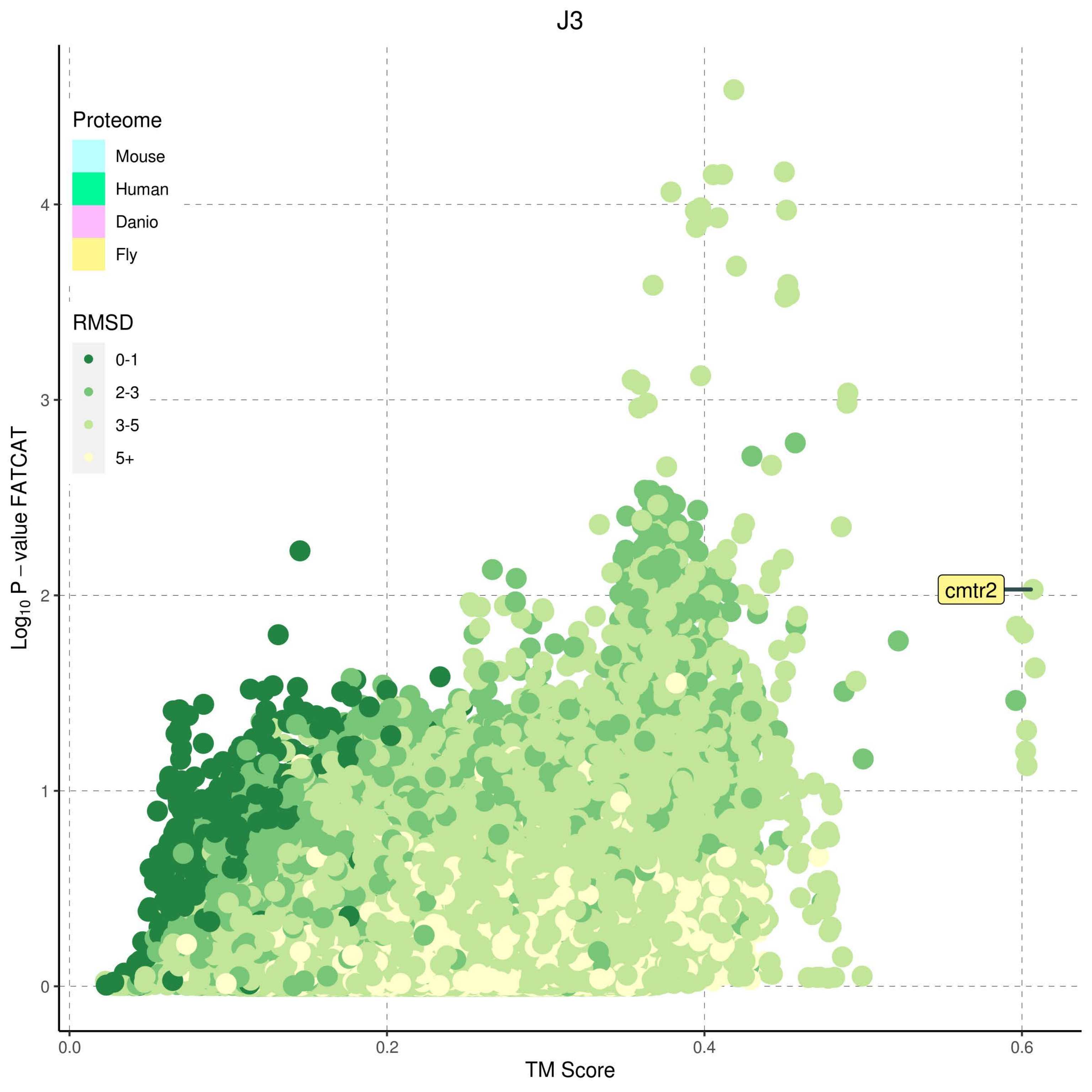

J3
Proteome
Mouse
Human
Danio
Fly
RMSD
0-1
2-3
3-5
5+
cmtr2
Log10 P − value FATCAT
TM Score
0
1
2
3
4
0.0
0.2
0.4
0.6

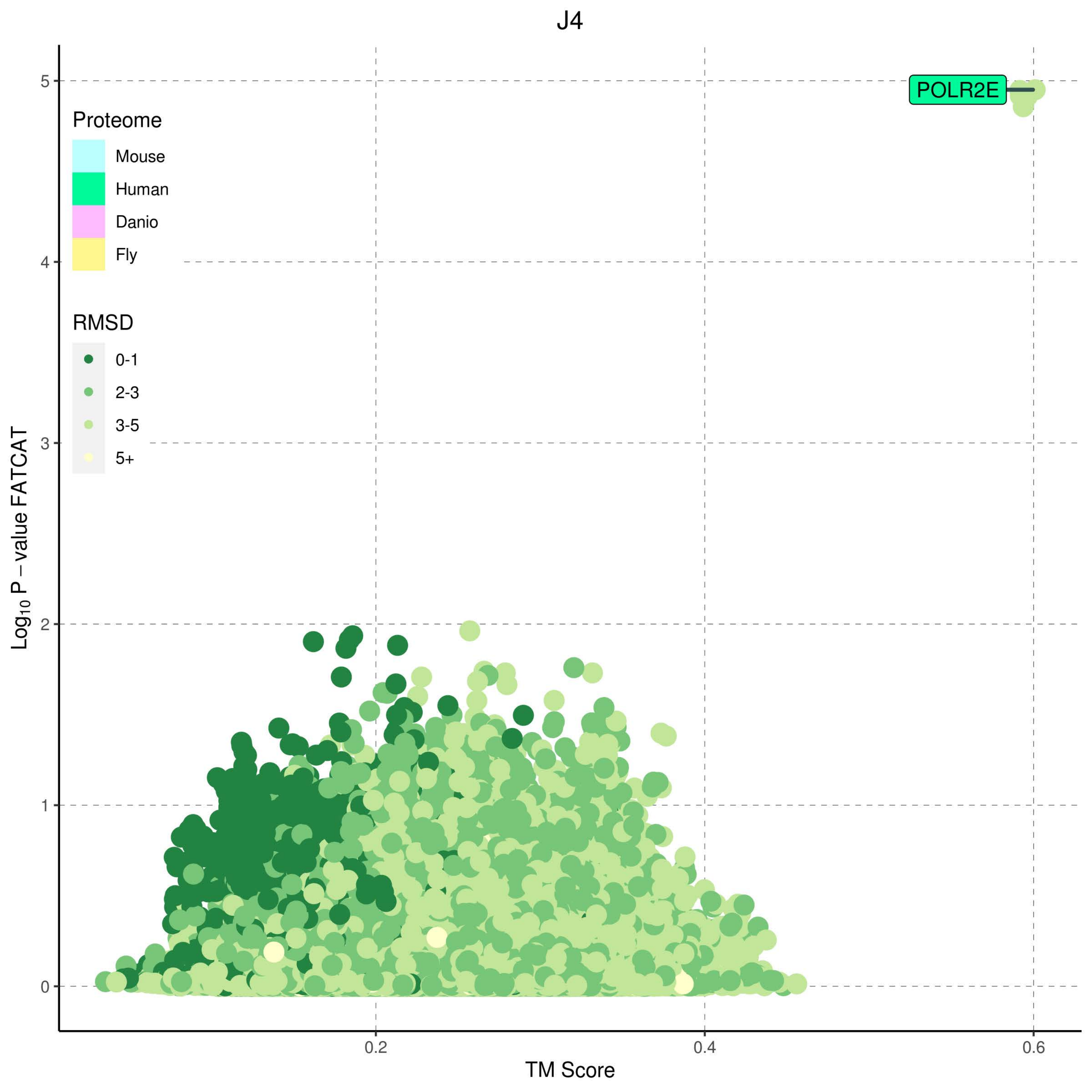
J4
POLR2E
Proteome
Mouse
Human
Danio
Fly
RMSD
0-1
2-3
3-5
5+
Log10 P – value FATCAT
0
1
2
3
4
5
0.2
0.4
0.6
TM Score

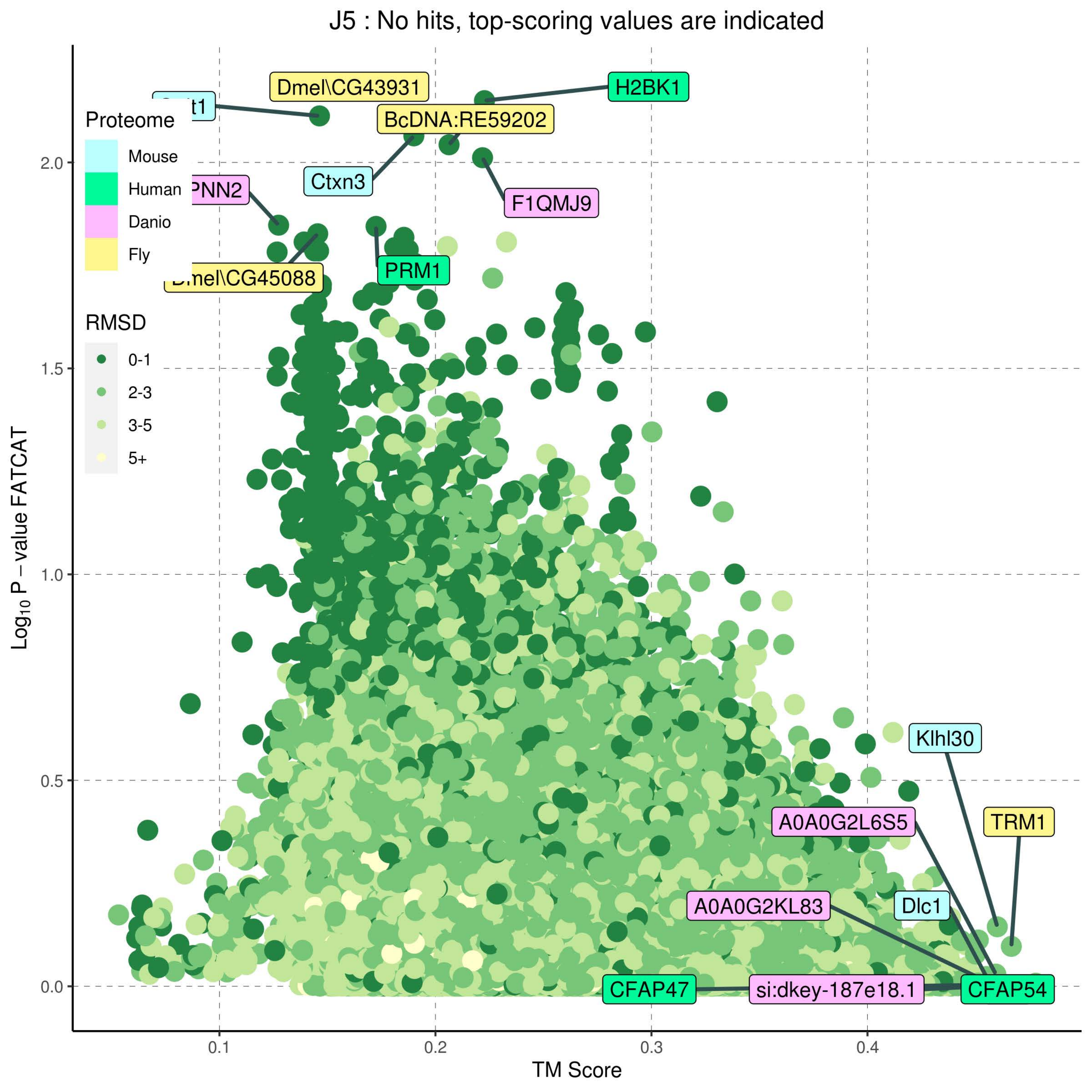

J5 : No hits, top-scoring values are indicated
Proteome
Mouse
Human
Danio
Fly
RMSD
0-1
2-3
3-5
5+
Dmel\CG43931
H2BK1
BcDNA:RE59202
Ctxn3
F1QMJ9
PNN2
PRM1
Dmel\CG45088
Klhl30
A0A0G2L6S5
TRM1
A0A0G2KL83
Dlc1
CFAP47
si:dkey-187e18.1
CFAP54
Log_{10} P – value FATCAT
TM Score
0.0
0.5
1.0
1.5
2.0
0.1
0.2
0.3
0.4

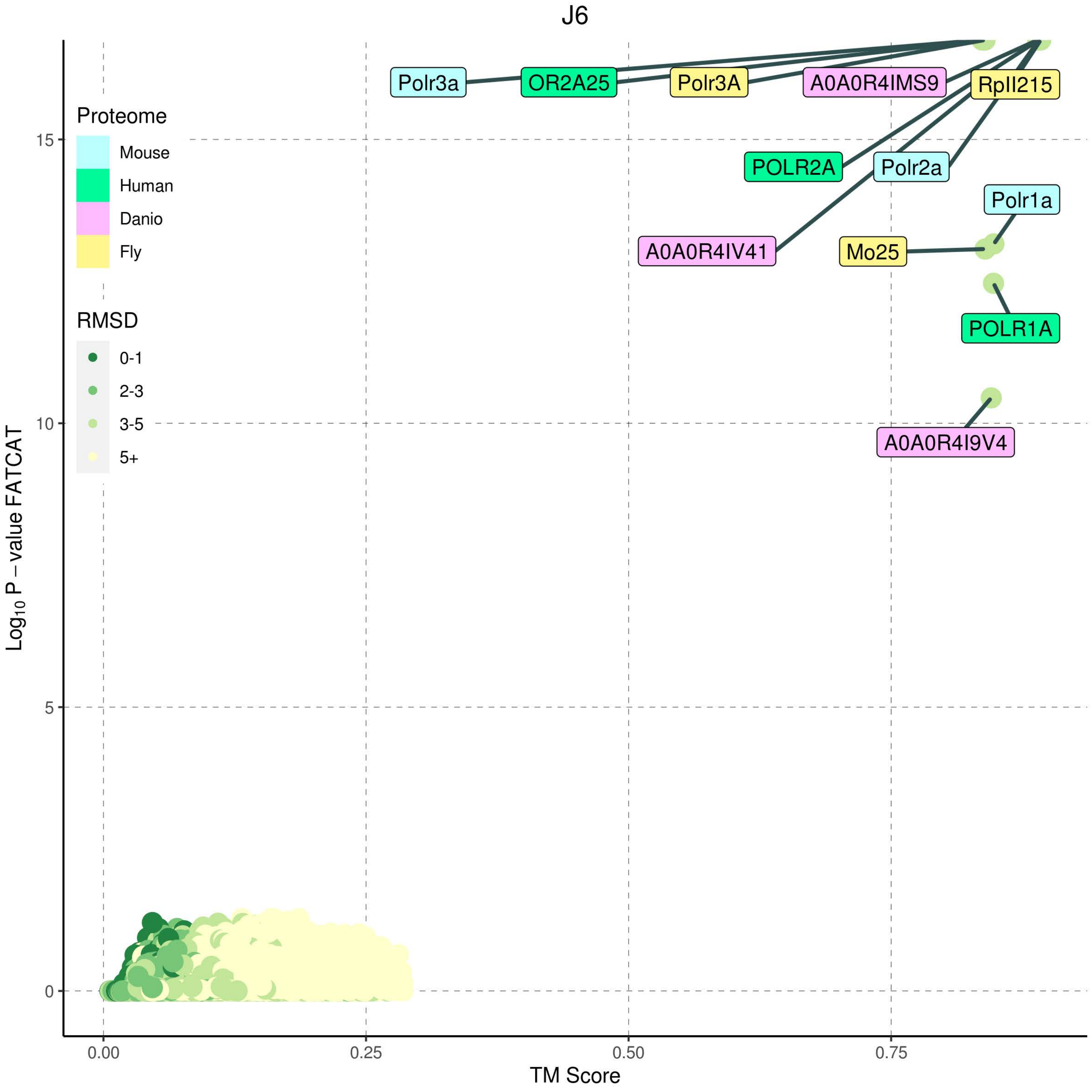

J6
Polr3a
OR2A25
Polr3A
A0A0R4IMS9
RpII215
Proteome
Mouse
Human
Danio
Fly
POLR2A
Polr2a
Polr1a
A0A0R4IV41
Mo25
POLR1A
RMSD
0-1
2-3
3-5
5+
A0A0R4I9V4
15
10
5
0
Log_{10} P − value FATCAT
0.00
0.25
0.50
0.75
TM Score

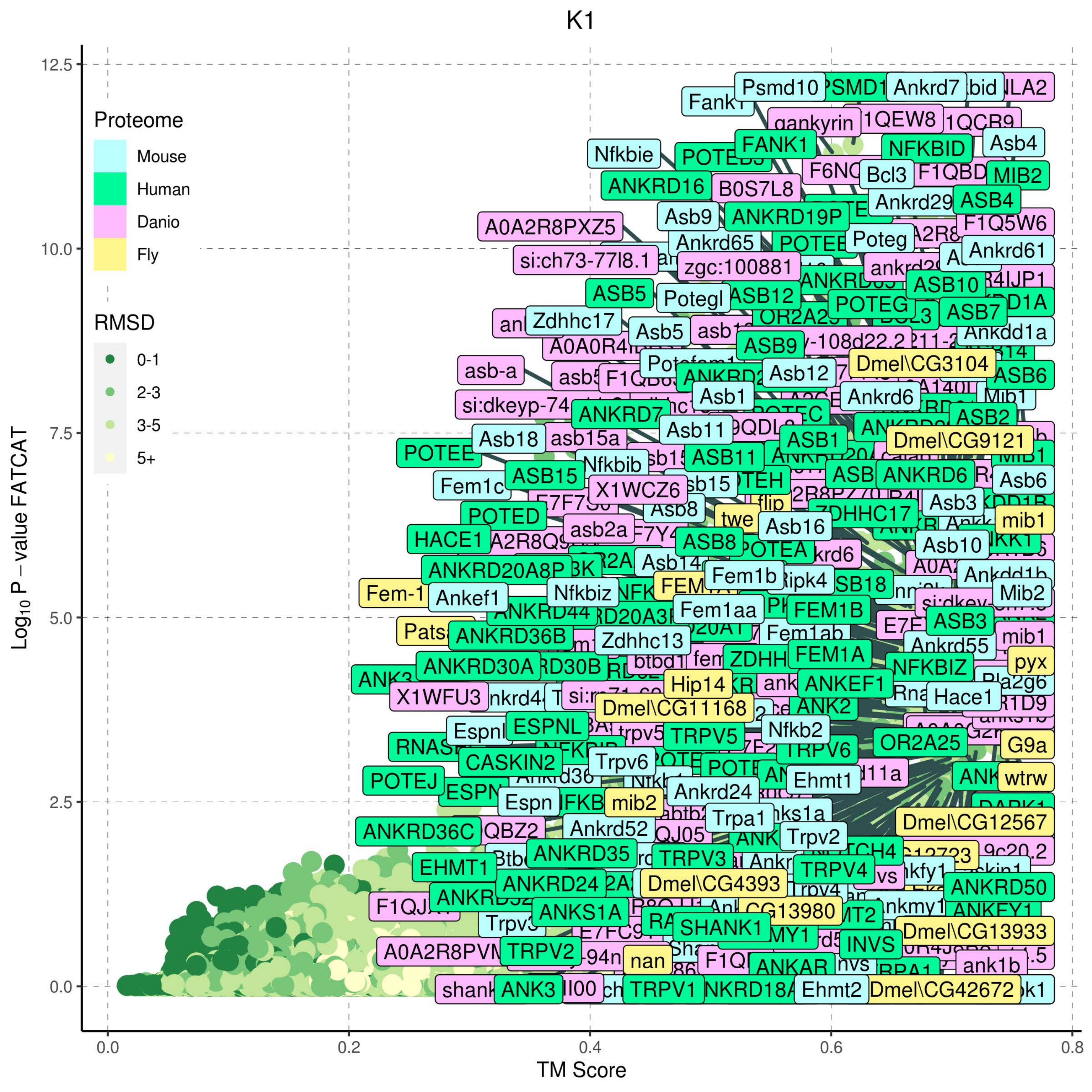
K1
Proteome
Mouse
Human
Danio
Fly
RMSD
0-1
2-3
3-5
5+
Log$_{10}$ P − value FATCAT
TM Score
0.0
2.5
5.0
7.5
10.0
12.5
0.0
0.2
0.4
0.6
0.8
Psmd10
Ankrd7
gankyrin
NFKBID
Asb4
FANK1
Nfkbie
Bcl3
MIB2
ANKRD16
B0S7L8
Ankrd29
ASB4
Asb9
ANKRD19P
A0A2R8PXZ5
F1Q5W6
Ankrd65
Poteg
Ankrd61
si:ch73-77l8.1
zgc:100881
ASB10
ASB5
Potegl
ASB12
POTEG
ASB7
Zdhhc17
Asb5
Ankdd1a
ASB9
Dmel\CG3104
asb-a
Asb12
ASB6
Asb1
Ankrd6
ANKRD7
ASB2
Asb11
ASB1
Dmel\CG9121
Asb18
asb15a
ASB11
Nfkbib
ASB15
ANKRD6
Asb6
Fem1c
X1WCZ6
Asb3
POTED
Asb8
twe
Asb16
mib1
HACE1
asb2a
ASB8
POTEA
Asb10
ANKRD20A8P
Asb14
Fem1b
Ripk4
Ankdd1b
Fem-1
Ankef1
Nfkbiz
Mib2
ANKRD44
Fem1aa
FEM1B
ASB3
ANKRD36B
Zdhhc13
Fem1ab
mib1
Ankrd55
ANKRD30A
FEM1A
NFKBIZ
pyx
X1WFU3
Hip14
ANKEF1
Hace1
Dmel\CG11168
ANK2
Espnl
ESPNL
Nfkb2
TRPV5
OR2A25
G9a
CASKIN2
TRPV6
Trpv6
POTEJ
Ehmt1
wtrw
Espn
mib2
Ankrd24
Trpa1
Dmel\CG12567
ANKRD36C
Ankrd52
Trpv2
ANKRD35
TRPV3
EHMT1
TRPV4
ANKRD24
Dmel\CG4393
ANKRD50
ANKS1A
CG13980
SHANK1
Dmel\CG13933
TRPV2
INVS
nan
ANKAR
ank1b
ANK3
TRPV1
Ehmt2
Dmel\CG42672

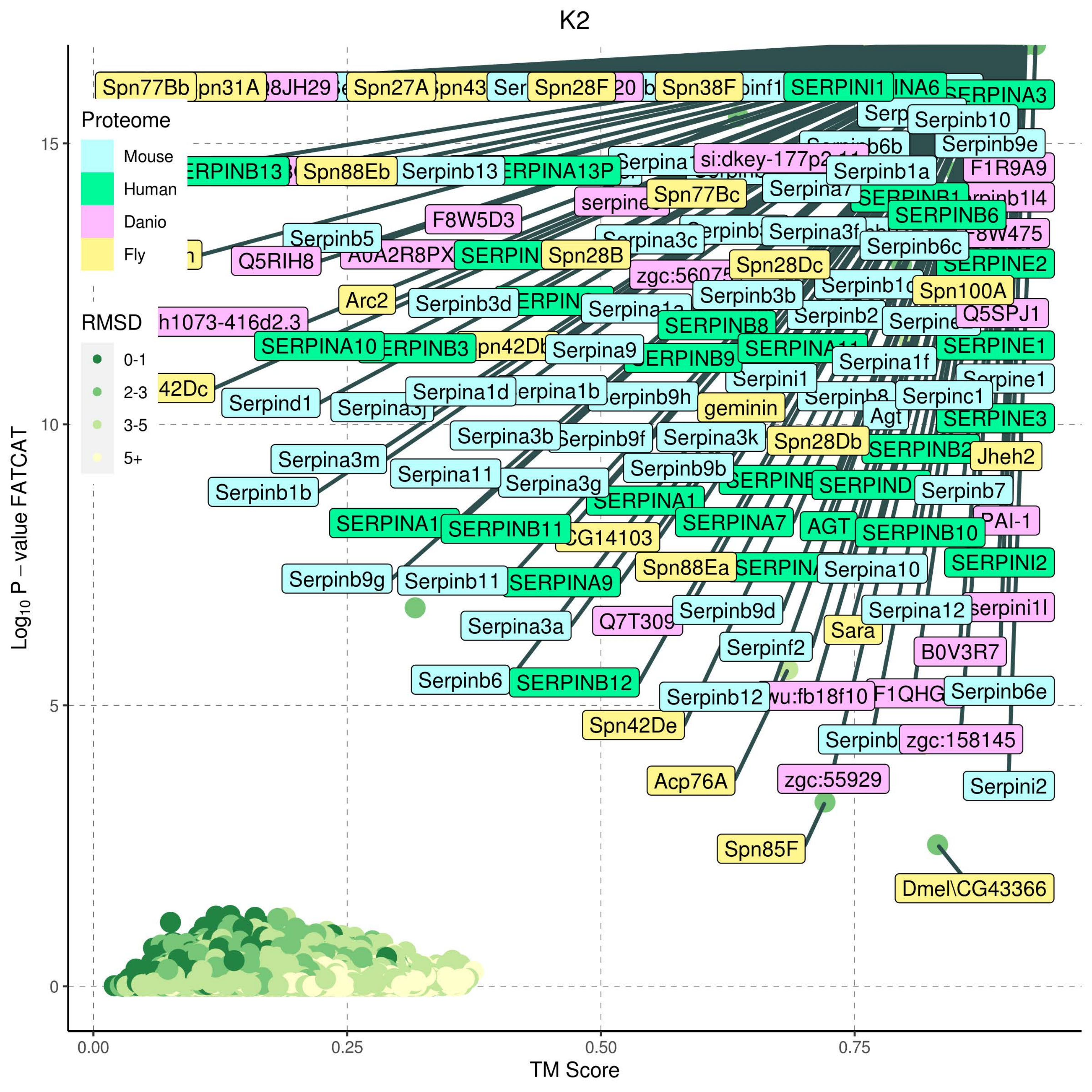

K2
Proteome
Mouse
Human
Danio
Fly
RMSD
0-1
2-3
3-5
5+
Log10 P − value FATCAT
TM Score
0.00
0.25
0.50
0.75
0
5
10
15
Spn77Bb
Spn27A
Spn28F
Spn38F
SERPINI1
SERPINA3
Serpinb10
Serpinb9e
Spn88Eb
Serpinb13
si:dkey-177p2
Serpinb1a
F1R9A9
Spn77Bc
SERPINB6
F8W5D3
Serpinb5
Serpina3c
Serpina3f
Serpinb6c
Q5RIH8
Spn28B
SERPINE2
zgc:56075
Spn28Dc
Arc2
Serpinb3d
Serpinb3b
Spn100A
Serpinb2
Q5SPJ1
SERPINB8
SERPINA10
Serpina9
SERPINB9
SERPINE1
Serpina1f
Serpini1
Serpind1
Serpina1d
Serpinc1
geminin
Agt
SERPINE3
Serpina3b
Serpinb9f
Serpina3k
Spn28Db
Jheh2
Serpina3m
Serpina11
Serpinb9b
Serpina3g
Serpinb7
Serpinb1b
SERPINB11
SERPINA7
AGT
SERPINB10
PAI-1
Spn88Ea
Serpina10
SERPINI2
Serpinb9g
Serpinb11
SERPINA9
Serpinb9d
Serpina12
serpini1l
Serpina3a
Sara
Serpinf2
B0V3R7
Serpinb6
SERPINB12
Serpinb12
wu:fb18f10
Serpinb6e
Spn42De
zgc:158145
Acp76A
zgc:55929
Serpini2
Spn85F
Dmel\CG43366

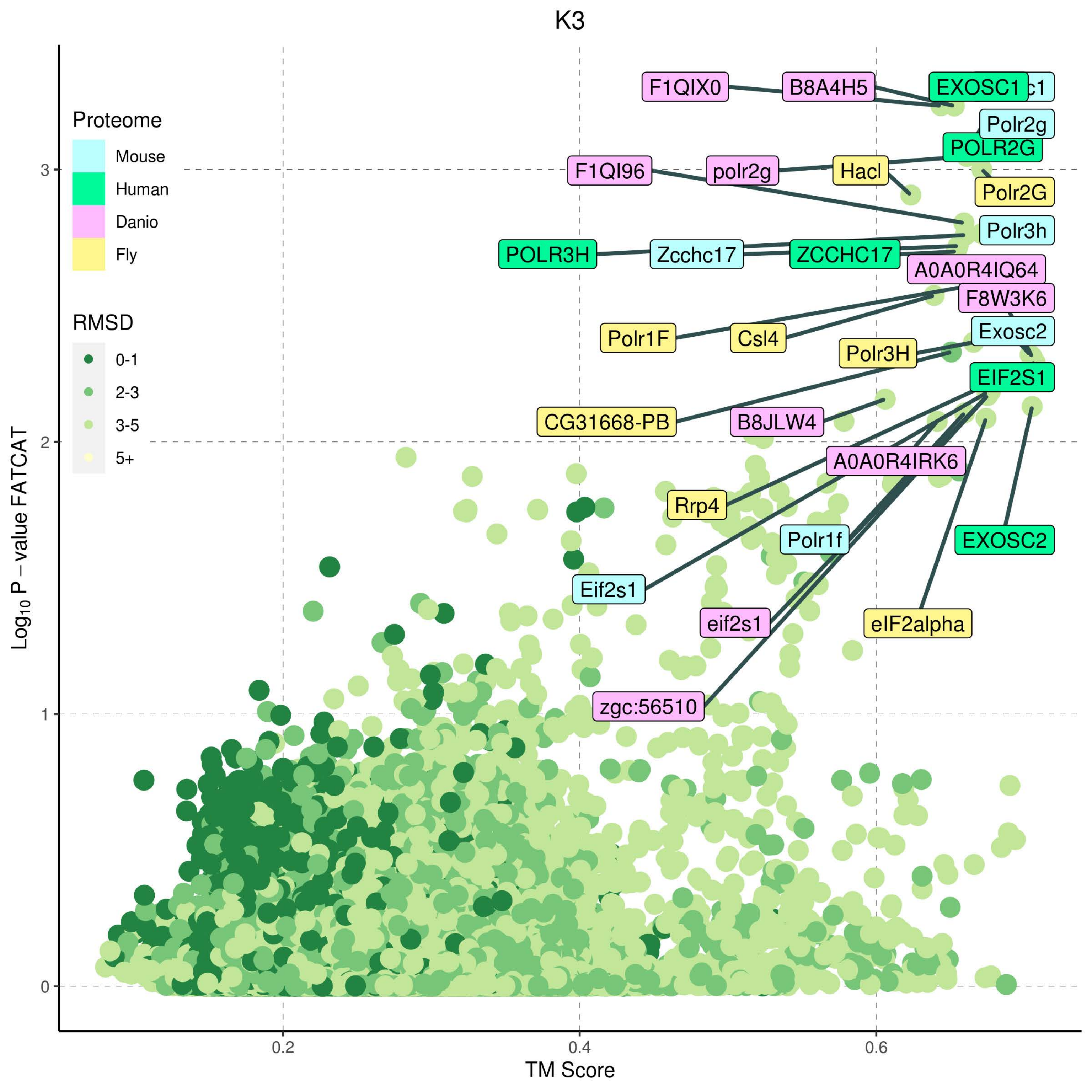

K3
Proteome
Mouse
Human
Danio
Fly
RMSD
0-1
2-3
3-5
5+
F1QIX0
B8A4H5
EXOSC1
Polr2g
POLR2G
F1QI96
polr2g
Hacl
Polr2G
Polr3h
POLR3H
Zcchc17
ZCCHC17
A0A0R4IQ64
F8W3K6
Polr1F
Csl4
Exosc2
Polr3H
EIF2S1
CG31668-PB
B8JLW4
A0A0R4IRK6
Rrp4
Polr1f
EXOSC2
Eif2s1
eif2s1
eIF2alpha
zgc:56510
Log10 P – value FATCAT
TM Score
0
1
2
3
0.2
0.4
0.6

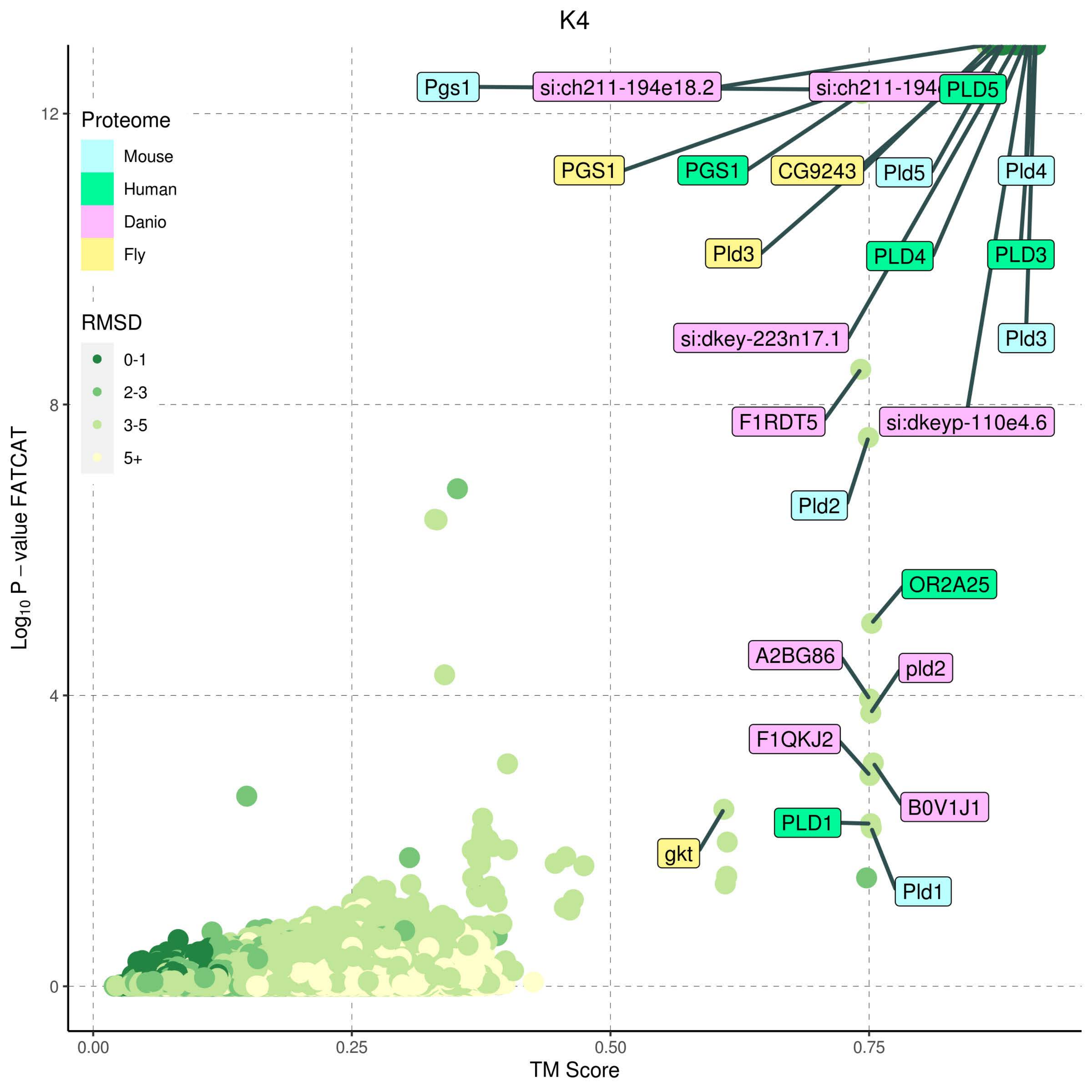

K4
Proteome
Mouse
Human
Danio
Fly
RMSD
0-1
2-3
3-5
5+
Pgs1
si:ch211-194e18.2
si:ch211-194
PLD5
PGS1
PGS1
CG9243
Pld5
Pld4
Pld3
PLD4
PLD3
si:dkey-223n17.1
Pld3
F1RDT5
si:dkeyp-110e4.6
Pld2
OR2A25
A2BG86
pld2
F1QKJ2
B0V1J1
PLD1
gkt
Pld1
Log10 P – value FATCAT
TM Score
0
4
8
12
0.00
0.25
0.50
0.75

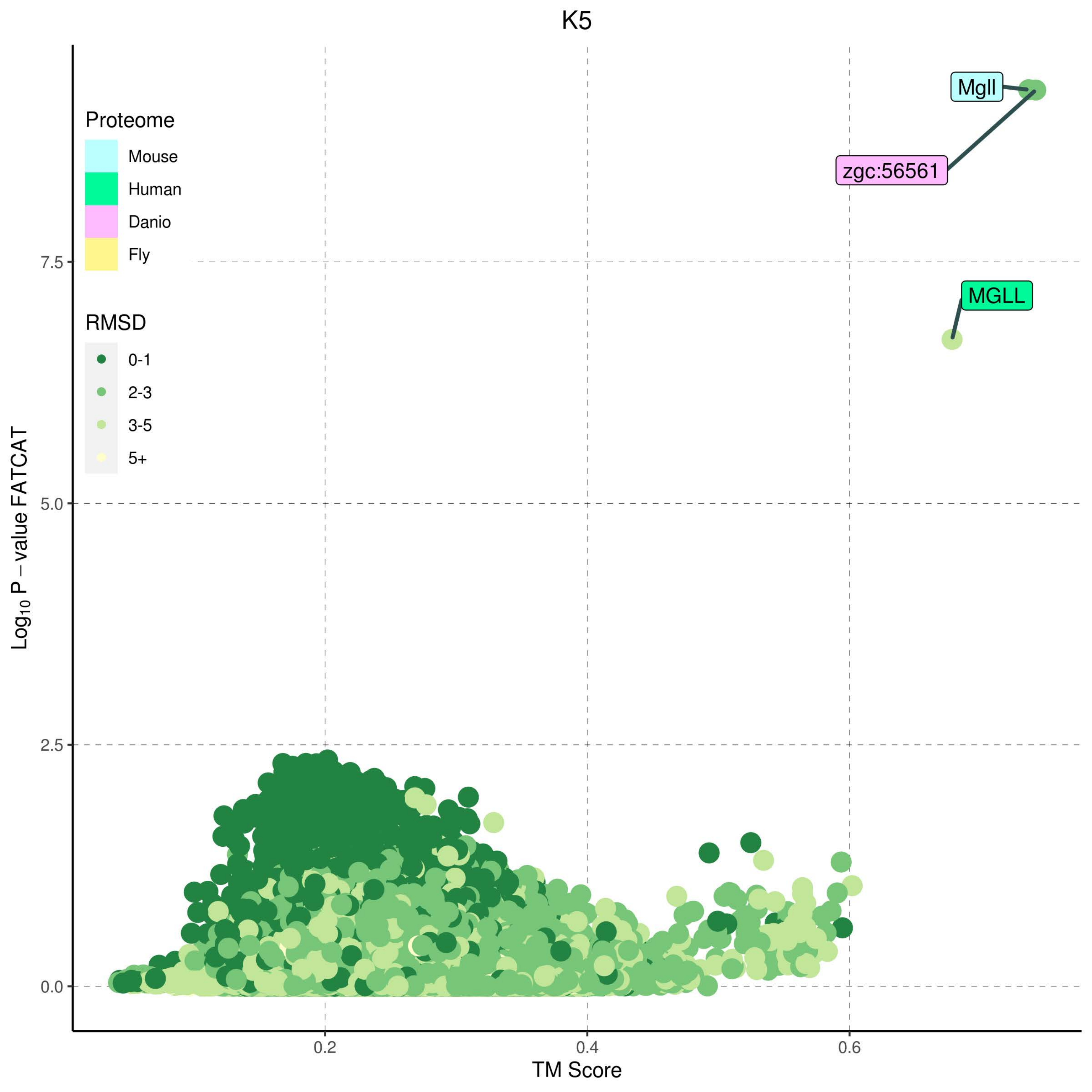
K5
Proteome
Mouse
Human
Danio
Fly
RMSD
0-1
2-3
3-5
5+
Mgll
zgc:56561
MGLL
7.5
5.0
2.5
0.0
0.2
0.4
0.6
TM Score
Log10 P – value FATCAT

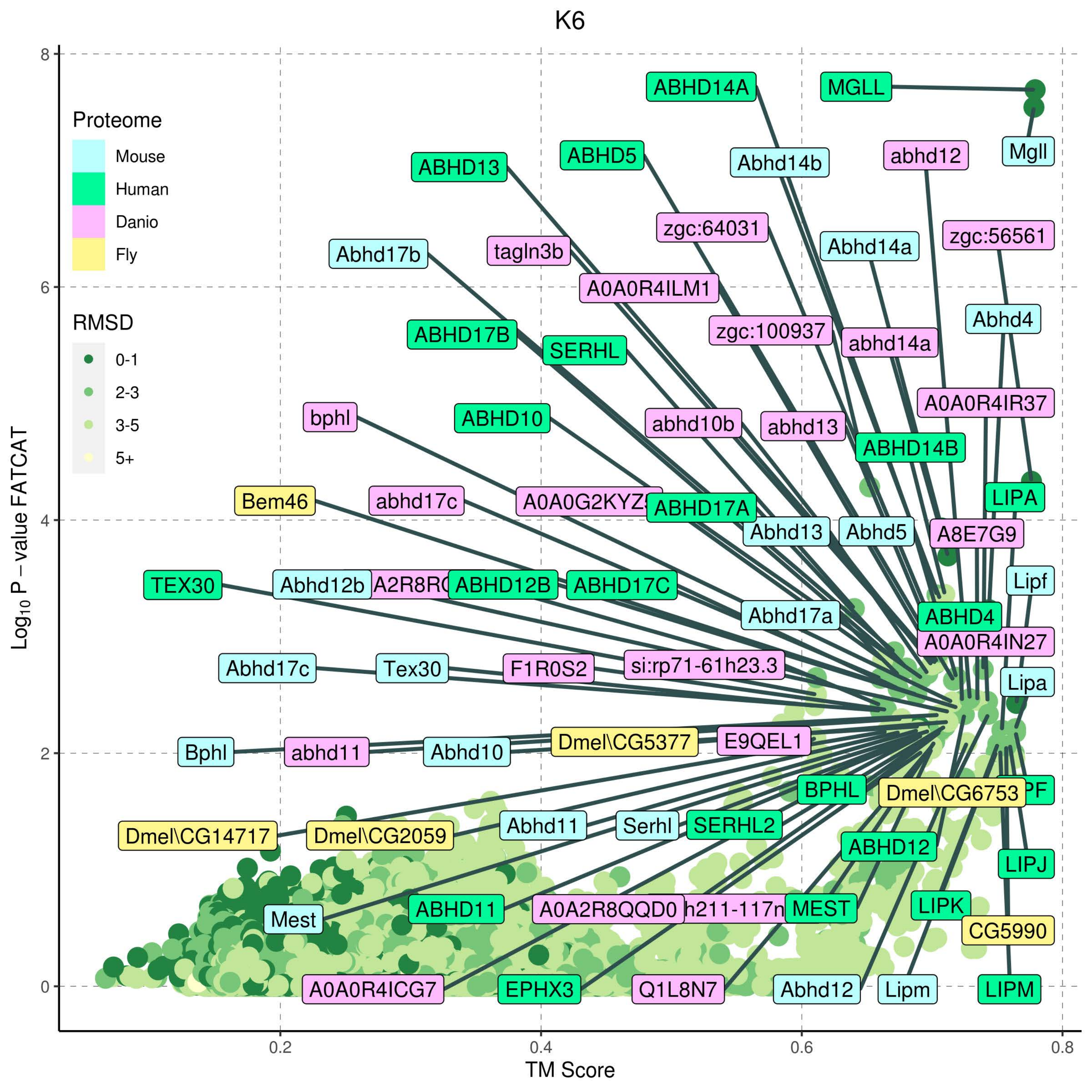

K6
Proteome
Mouse
Human
Danio
Fly
RMSD
0-1
2-3
3-5
5+
ABHD14A
MGLL
Mgll
ABHD13
ABHD5
Abhd14b
abhd12
zgc:64031
Abhd14a
zgc:56561
Abhd17b
tagln3b
A0A0R4ILM1
Abhd4
zgc:100937
abhd14a
ABHD17B
SERHL
A0A0R4IR37
bphl
ABHD10
abhd10b
abhd13
ABHD14B
Bem46
abhd17c
ABHD17A
LIPA
Abhd13
Abhd5
A8E7G9
TEX30
Abhd12b
ABHD12B
ABHD17C
Lipf
Abhd17a
ABHD4
A0A0R4IN27
Abhd17c
Tex30
F1R0S2
si:rp71-61h23.3
Lipa
Bphl
abhd11
Abhd10
Dmel\CG5377
E9QEL1
BPHL
Dmel\CG6753
Dmel\CG14717
Dmel\CG2059
Abhd11
Serhl
SERHL2
ABHD12
LIPJ
Mest
ABHD11
A0A2R8QQD0
MEST
LIPK
CG5990
A0A0R4ICG7
EPHX3
Q1L8N7
Abhd12
Lipm
LIPM
Log10 P – value FATCAT
TM Score
0
2
4
6
8
0.2
0.4
0.6
0.8

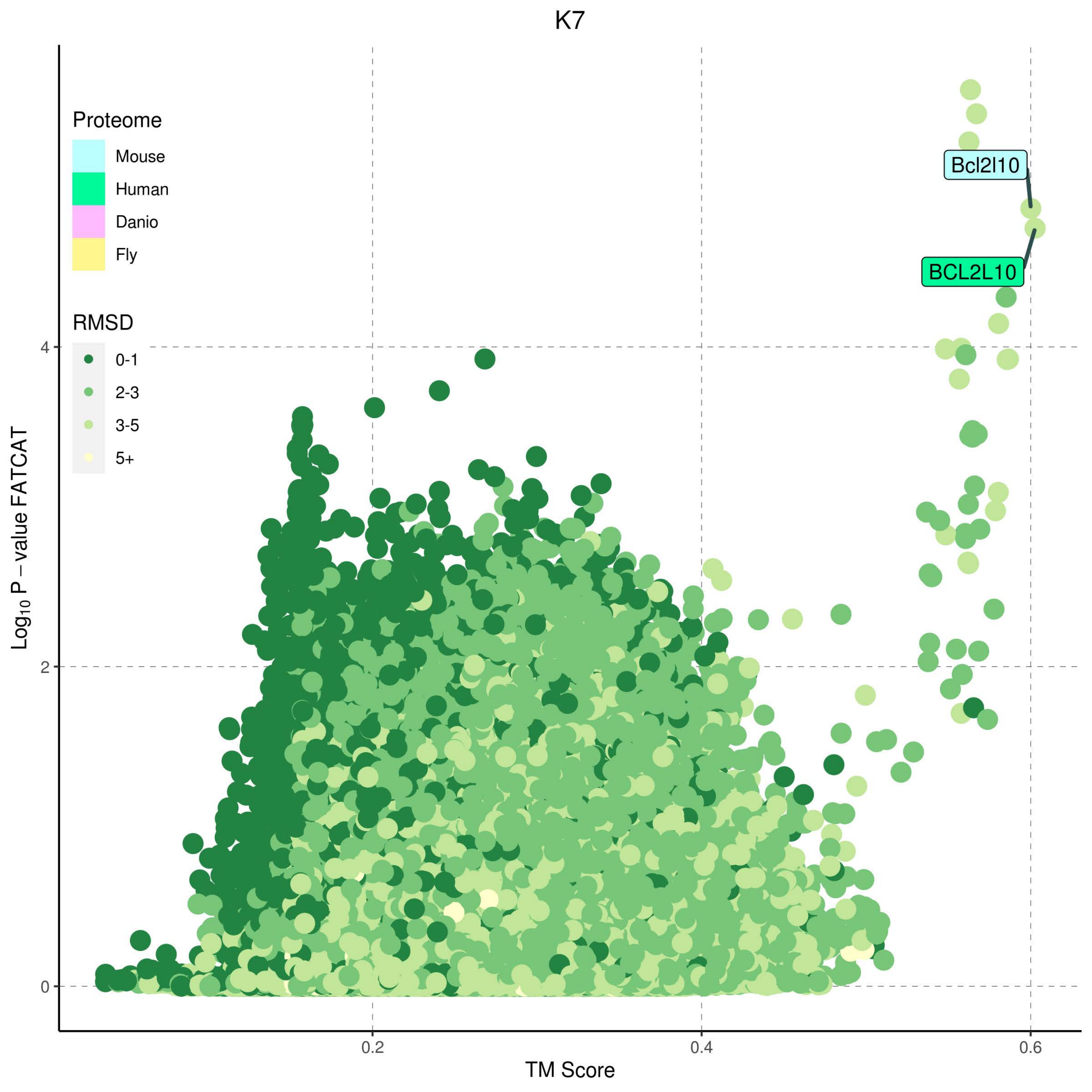

K7
Proteome
Mouse
Human
Danio
Fly
RMSD
0-1
2-3
3-5
5+
Bcl2l10
BCL2L10
Log10 P − value FATCAT
TM Score
0
2
4
0.2
0.4
0.6

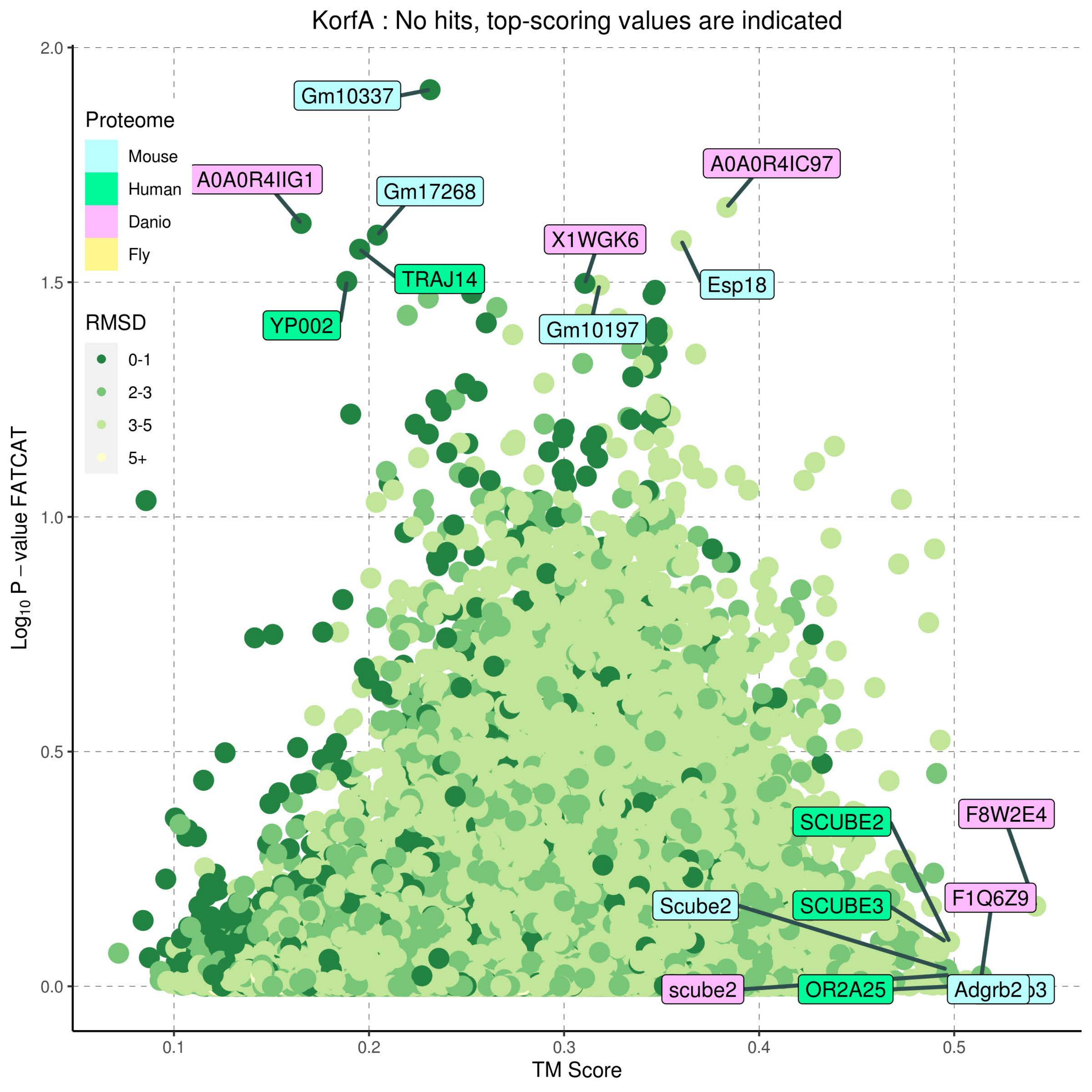

KorfA : No hits, top-scoring values are indicated
Proteome
Mouse
Human
Danio
Fly
RMSD
0-1
2-3
3-5
5+
Log10 P − value FATCAT
TM Score
2.0
1.5
1.0
0.5
0.0
0.1
0.2
0.3
0.4
0.5
Gm10337
A0A0R4IIG1
Gm17268
A0A0R4IC97
X1WGK6
Esp18
TRAJ14
YP002
Gm10197
SCUBE2
F8W2E4
Scube2
SCUBE3
F1Q6Z9
scube2
OR2A25
Adgrb2

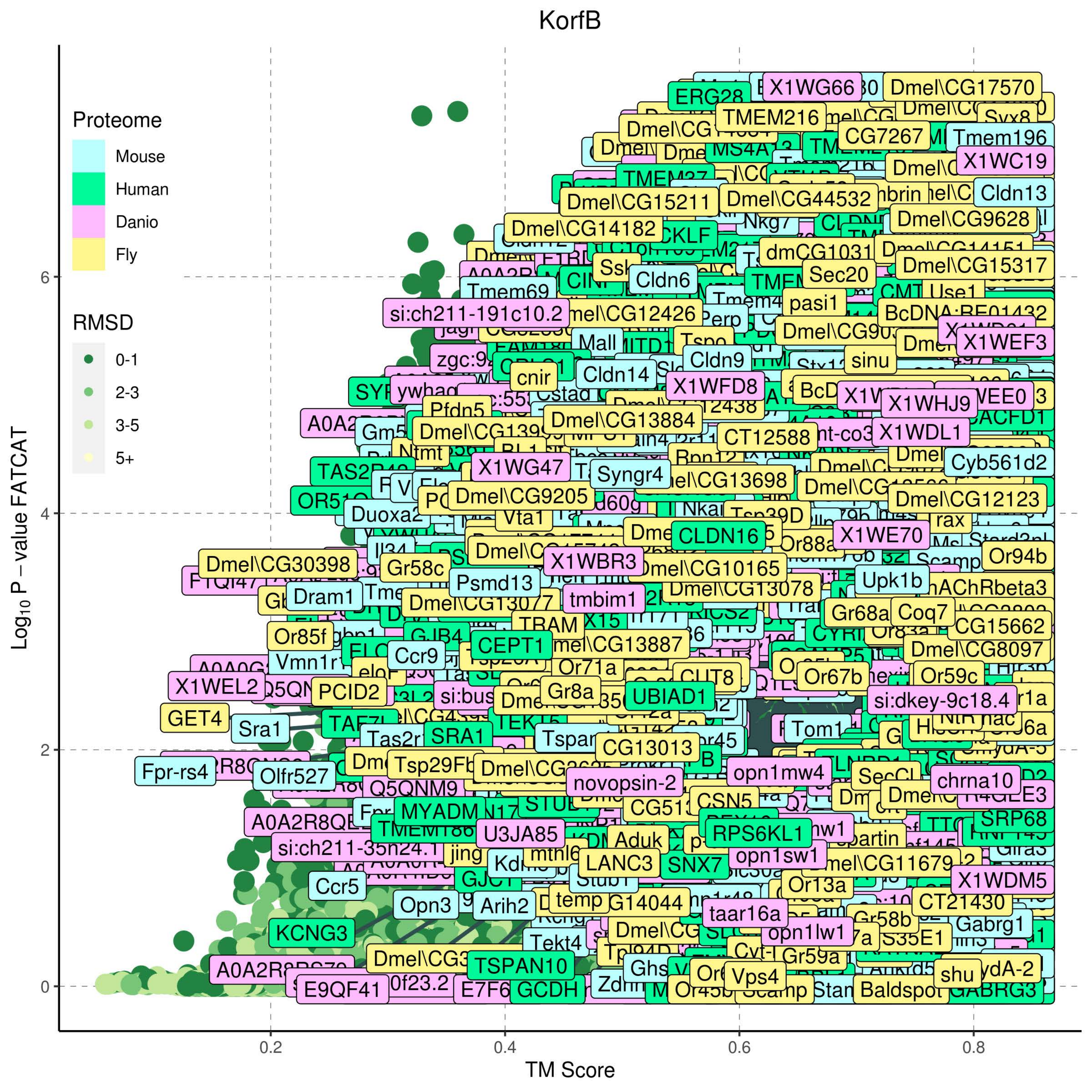

KorfB
Proteome
Mouse
Human
Danio
Fly
RMSD
0-1
2-3
3-5
5+
Log10 P − value FATCAT
TM Score
0
2
4
6
0.2
0.4
0.6
0.8
X1WG66
Dmel\CG17570
ERG28
TMEM216
CG7267
Tmem196
X1WC19
Cldn13
Dmel\CG15211
Dmel\CG44532
Dmel\CG9628
Dmel\CG14182
Nkg7
Dmel\CG15317
Cldn6
Sec20
pasi1
si:ch211-191c10.2
Mall
Cldn9
sinu
X1WEF3
Cldn14
X1WFD8
X1WHJ9
Dmel\CG13884
CT12588
X1WDL1
Pfdn5
X1WG47
Syngr4
Cyb561d2
Dmel\CG9205
Dmel\CG12123
Duoxa2
Vta1
CLDN16
X1WE70
Or94b
Dmel\CG30398
Gr58c
X1WBR3
Psmd13
Upk1b
Dram1
tmbim1
Gr68a
Coq7
CG15662
Or85f
CEPT1
Ccr9
Dmel\CG8097
X1WEL2
PCID2
Or67b
Or59c
Gr8a
UBIAD1
si:dkey-9c18.4
GET4
Sra1
SRA1
Tom1
CG13013
Fpr-rs4
Olfr527
Q5QNM9
novopsin-2
opn1mw4
chrna10
MYADM
SRP68
U3JA85
RPS6KL1
Aduk
si:ch211-35n24.1
LANC3
SNX7
opn1sw1
X1WDM5
Ccr5
Or13a
Opn3
Arih2
CT21430
KCNG3
taar16a
Gabrg1
Tekt4
opn1lw1
A0A2R8QE
TSPAN10
Vps4
shu
E9QF41
GCDH
Baldspot
GABRG3

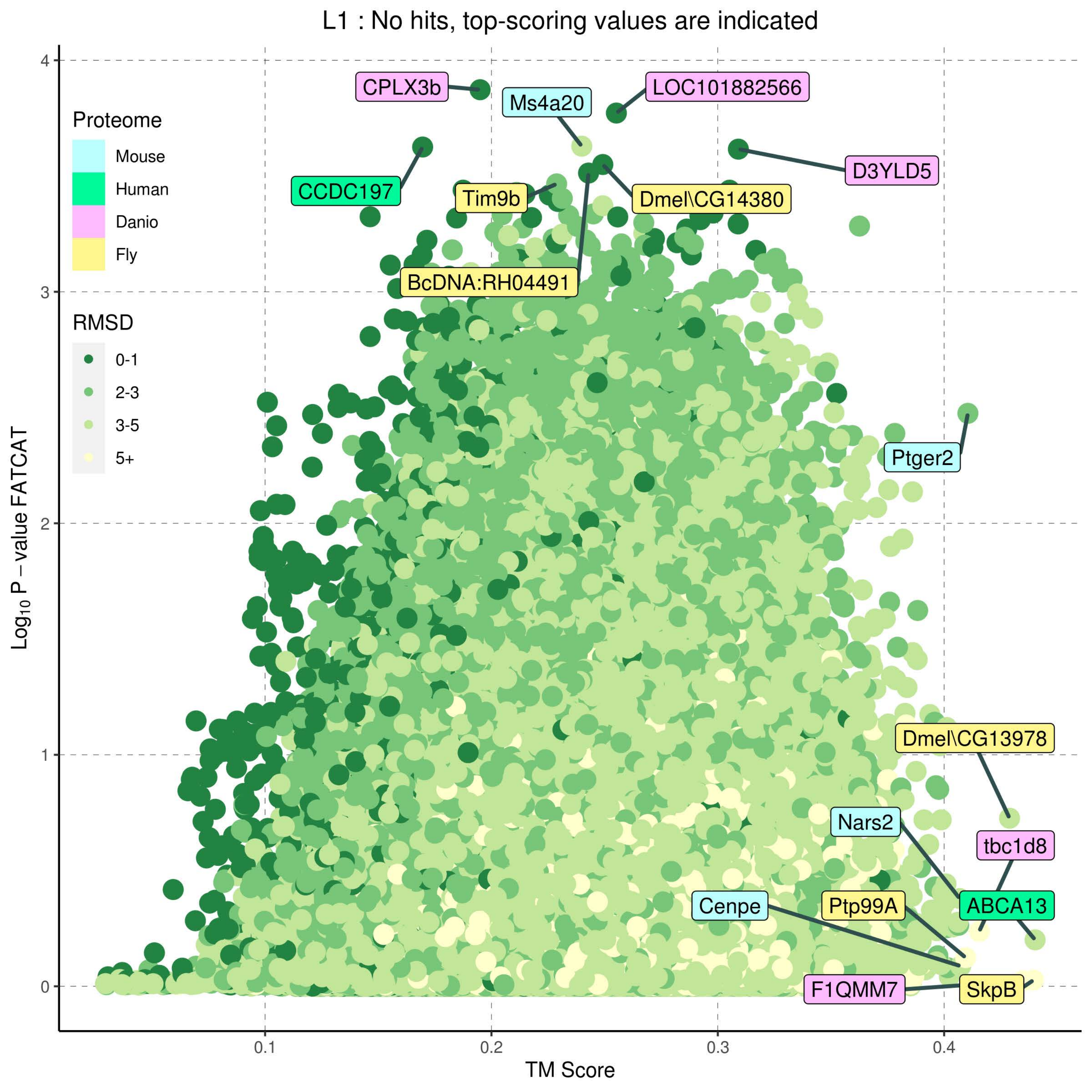

L1 : No hits, top-scoring values are indicated
Proteome
Mouse
Human
Danio
Fly
RMSD
0-1
2-3
3-5
5+
Log10 P − value FATCAT
TM Score
0
1
2
3
4
0.1
0.2
0.3
0.4
CPLX3b
Ms4a20
LOC101882566
CCDC197
Tim9b
Dmel\CG14380
D3YLD5
BcDNA:RH04491
Ptger2
Dmel\CG13978
Nars2
tbc1d8
Cenpe
Ptp99A
ABCA13
F1QMM7
SkpB

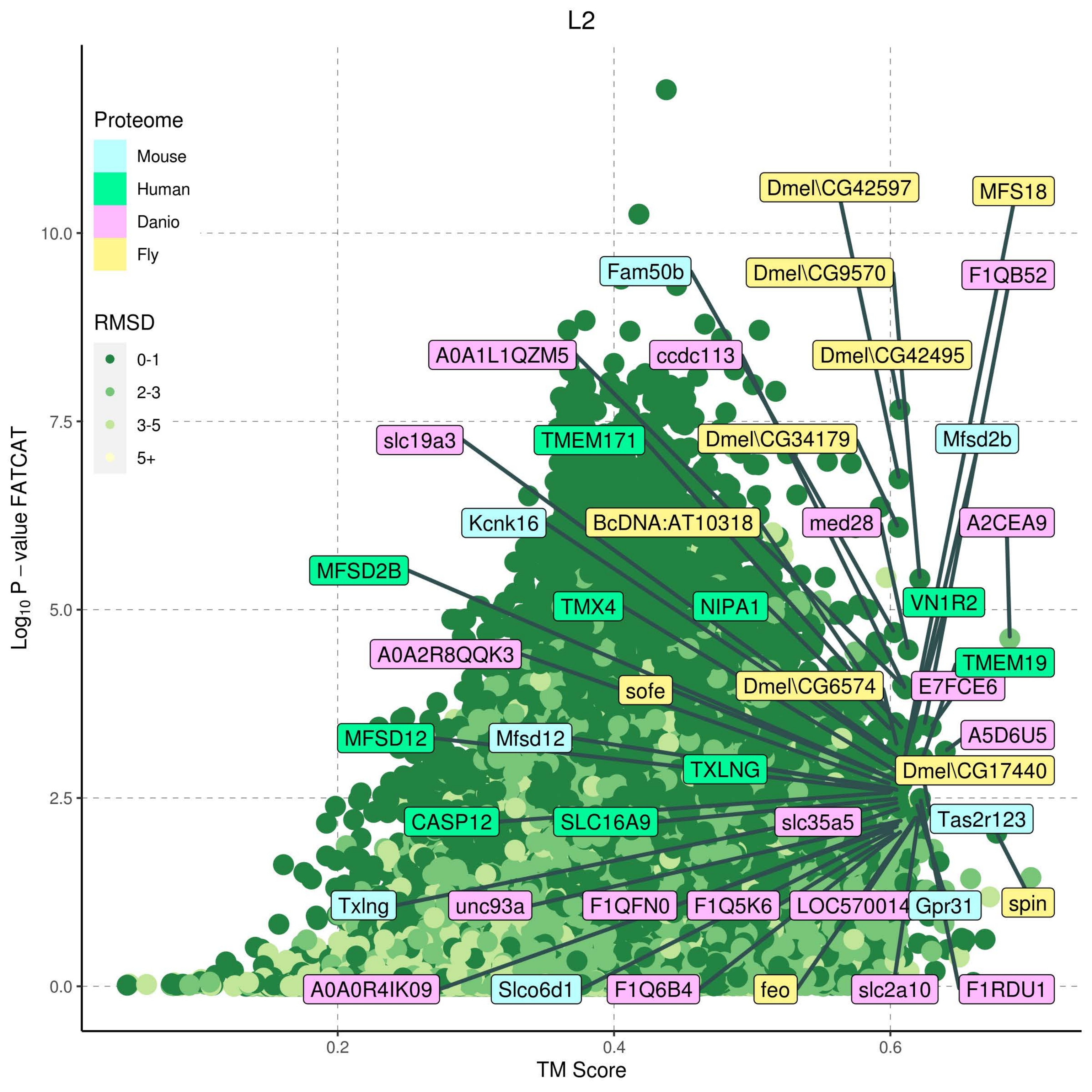

L2
Proteome
Mouse
Human
Danio
Fly
RMSD
0-1
2-3
3-5
5+
Log10 P − value FATCAT
TM Score
0.0
2.5
5.0
7.5
10.0
0.2
0.4
0.6
Dmel\CG42597
MFS18
Fam50b
Dmel\CG9570
F1QB52
A0A1L1QZM5
ccdc113
Dmel\CG42495
slc19a3
TMEM171
Dmel\CG34179
Mfsd2b
Kcnk16
BcDNA:AT10318
med28
A2CEA9
MFSD2B
TMX4
NIPA1
VN1R2
A0A2R8QQK3
TMEM19
sofe
Dmel\CG6574
E7FCE6
A5D6U5
MFSD12
Mfsd12
TXLNG
Dmel\CG17440
CASP12
SLC16A9
slc35a5
Tas2r123
Txlng
unc93a
F1QFN0
F1Q5K6
LOC570014
Gpr31
spin
A0A0R4IK09
Slco6d1
F1Q6B4
feo
slc2a10
F1RDU1

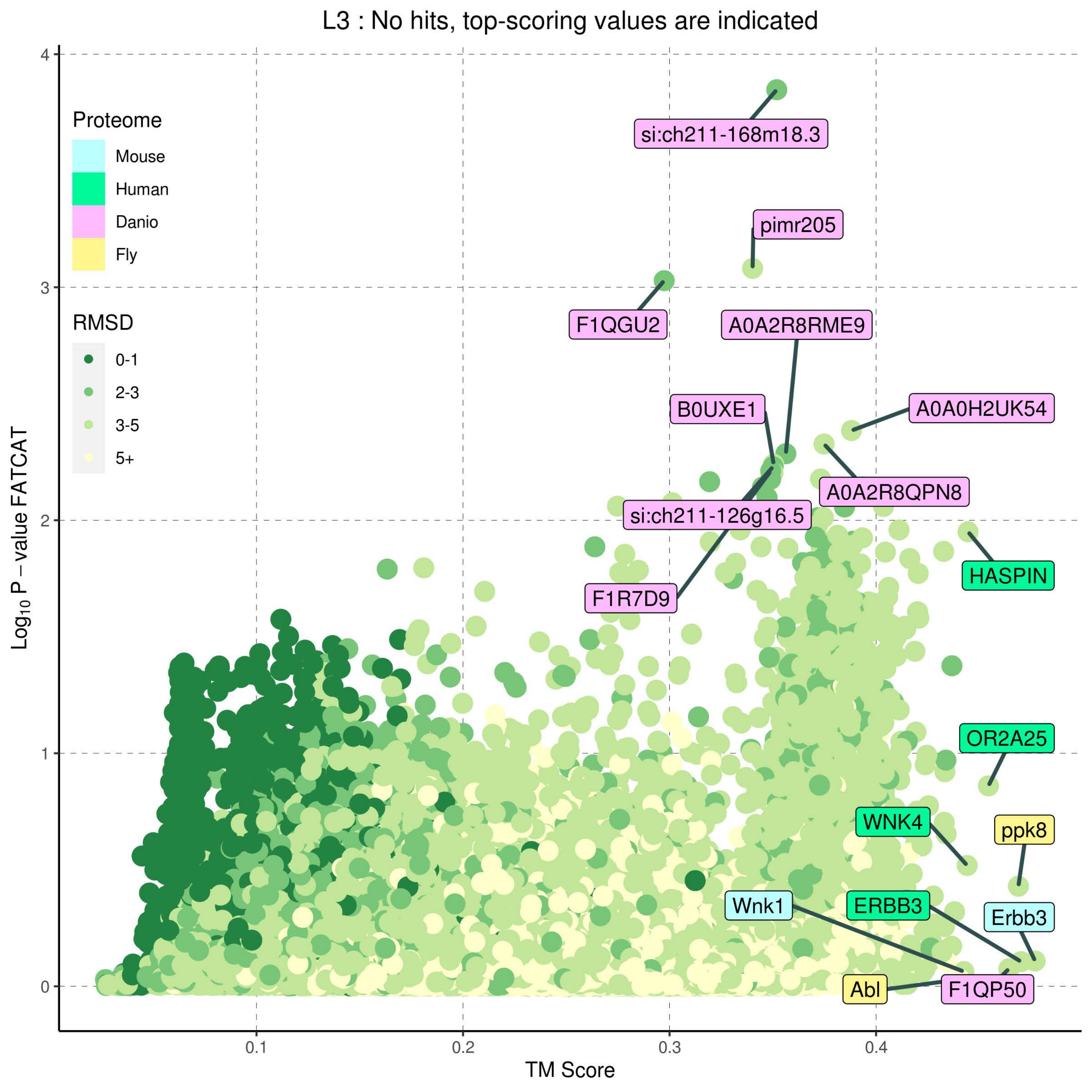
L3 : No hits, top-scoring values are indicated
Proteome
Mouse
Human
Danio
Fly
RMSD
0-1
2-3
3-5
5+
si:ch211-168m18.3
pimr205
F1QGU2
A0A2R8RME9
B0UXE1
A0A0H2UK54
A0A2R8QPN8
si:ch211-126g16.5
HASPIN
F1R7D9
OR2A25
WNK4
ppk8
Wnk1
ERBB3
Erbb3
Abl
F1QP50
Log10 P − value FATCAT
TM Score
0
1
2
3
4
0.1
0.2
0.3
0.4

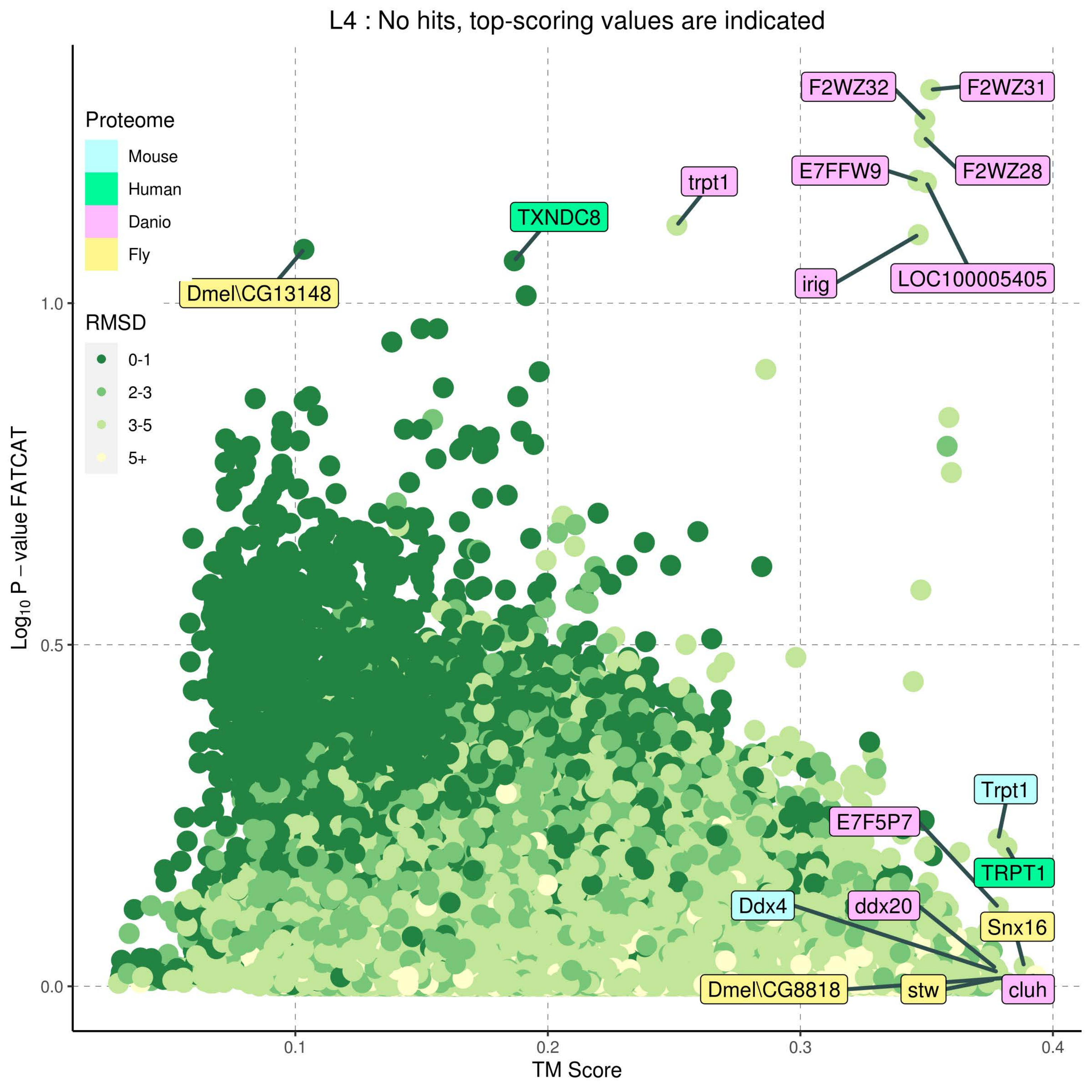
L4 : No hits, top-scoring values are indicated
Proteome
Mouse
Human
Danio
Fly
RMSD
0-1
2-3
3-5
5+
Log10 P − value FATCAT
TM Score
0.0
0.5
1.0
0.1
0.2
0.3
0.4
F2WZ32
F2WZ31
F2WZ28
E7FFW9
trpt1
TXNDC8
Dmel\CG13148
irig
LOC100005405
Trpt1
E7F5P7
TRPT1
Ddx4
ddx20
Snx16
Dmel\CG8818
stw
cluh

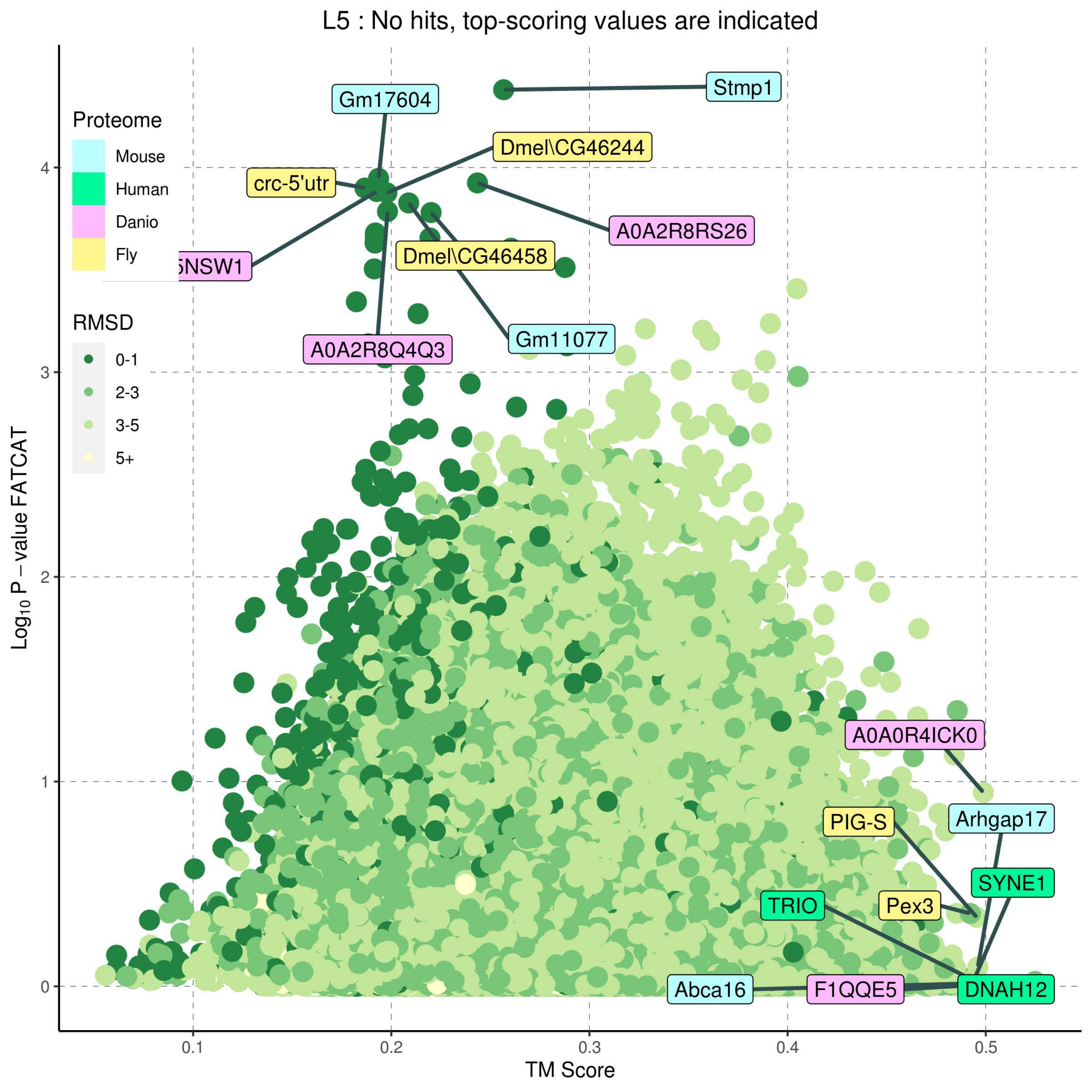

L5 : No hits, top-scoring values are indicated
Proteome
Mouse
Human
Danio
Fly
RMSD
0-1
2-3
3-5
5+
Stmp1
Gm17604
Dmel\CG46244
crc-5'utr
A0A2R8RS26
5NSW1
Dmel\CG46458
Gm11077
A0A2R8Q4Q3
A0A0R4ICK0
PIG-S
Arhgap17
SYNE1
TRIO
Pex3
Abca16
F1QQE5
DNAH12
Log10 P – value FATCAT
TM Score
0
1
2
3
4
0.1
0.2
0.3
0.4
0.5

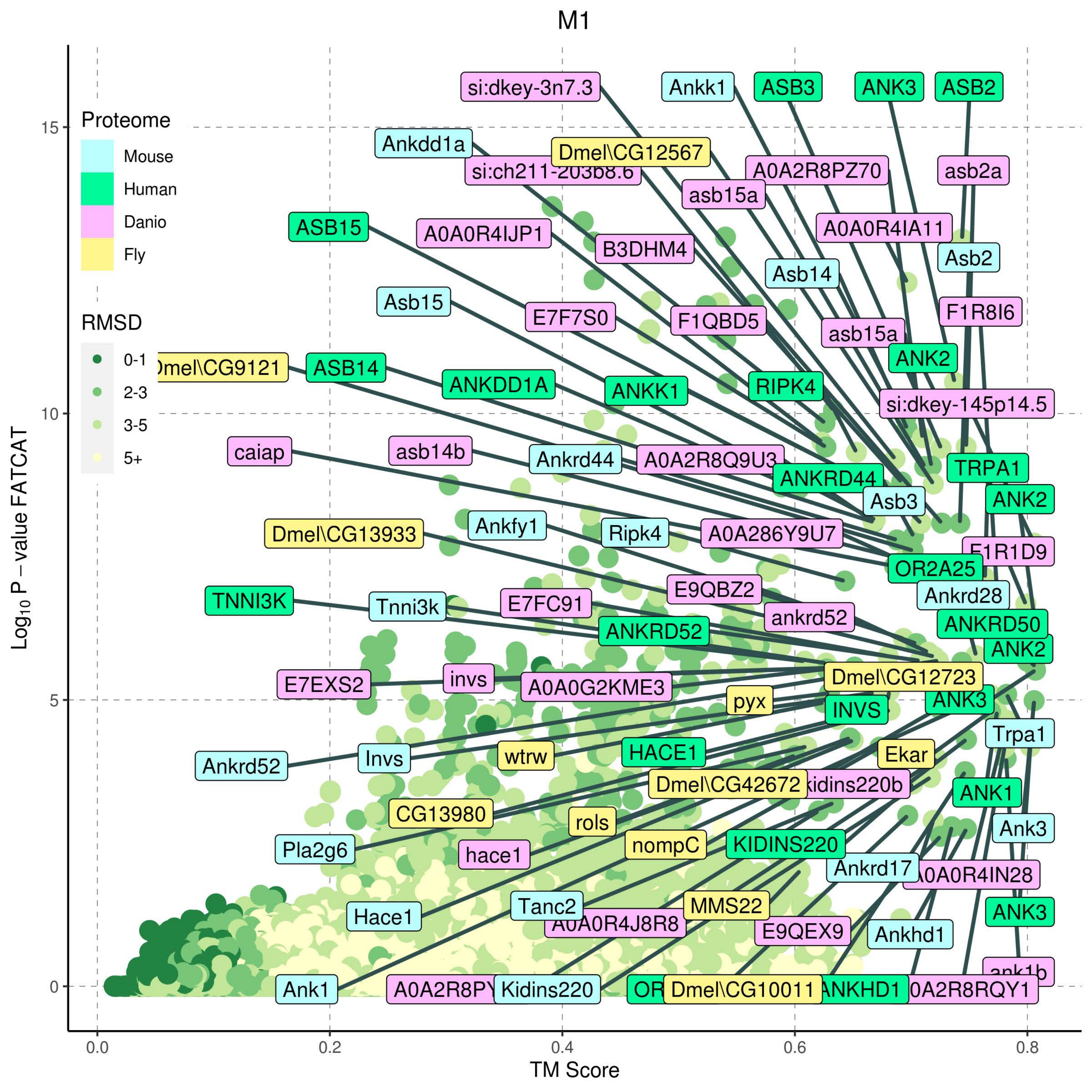
M1
Proteome
Mouse
Human
Danio
Fly
RMSD
0-1
2-3
3-5
5+
Log10 P − value FATCAT
TM Score
0
5
10
15
0.0
0.2
0.4
0.6
0.8
si:dkey-3n7.3
Ankk1
ASB3
ANK3
ASB2
Ankdd1a
Dmel\CG12567
A0A2R8PZ70
asb2a
asb15a
ASB15
A0A0R4IJP1
B3DHM4
A0A0R4IA11
Asb2
Asb14
Asb15
E7F7S0
F1QBD5
F1R8I6
asb15a
ASB14
ANK2
ANKDD1A
ANKK1
RIPK4
si:dkey-145p14.5
caiap
asb14b
Ankrd44
ANKRD44
TRPA1
Asb3
ANK2
Ankfy1
Dmel\CG13933
Ripk4
A0A286Y9U7
OR2A25
E9QBZ2
TNNI3K
Tnni3k
E7FC91
Ankrd28
ankrd52
ANKRD50
ANKRD52
ANK2
E7EXS2
invs
A0A0G2KME3
Dmel\CG12723
pyx
ANK3
INVS
Trpa1
Ankrd52
Invs
wtrw
HACE1
Ekar
Dmel\CG42672
ANK1
CG13980
rols
Ank3
Pla2g6
hace1
nompC
KIDINS220
Ankrd17
ANK3
Hace1
Tanc2
MMS22
E9QEX9
Ankhd1
Ank1
Kidins220
Dmel\CG10011
ANKHD1

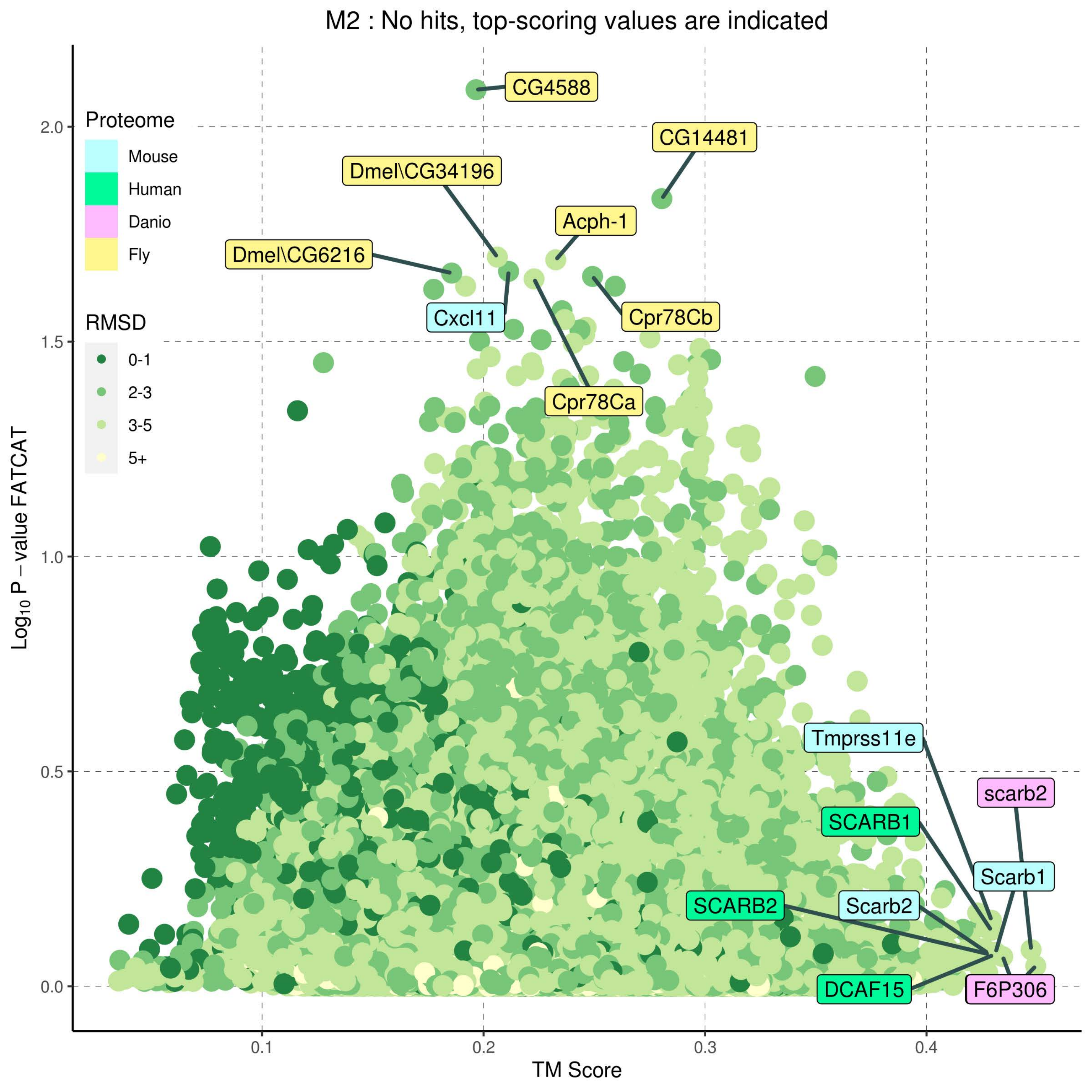
M2 : No hits, top-scoring values are indicated
Proteome
Mouse
Human
Danio
Fly
RMSD
0-1
2-3
3-5
5+
CG4588
CG14481
Dmel\CG34196
Acph-1
Dmel\CG6216
Cxcl11
Cpr78Cb
Cpr78Ca
Tmprss11e
scarb2
SCARB1
Scarb1
SCARB2
Scarb2
DCAF15
F6P306
Log10 P – value FATCAT
TM Score
0.0
0.5
1.0
1.5
2.0
0.1
0.2
0.3
0.4

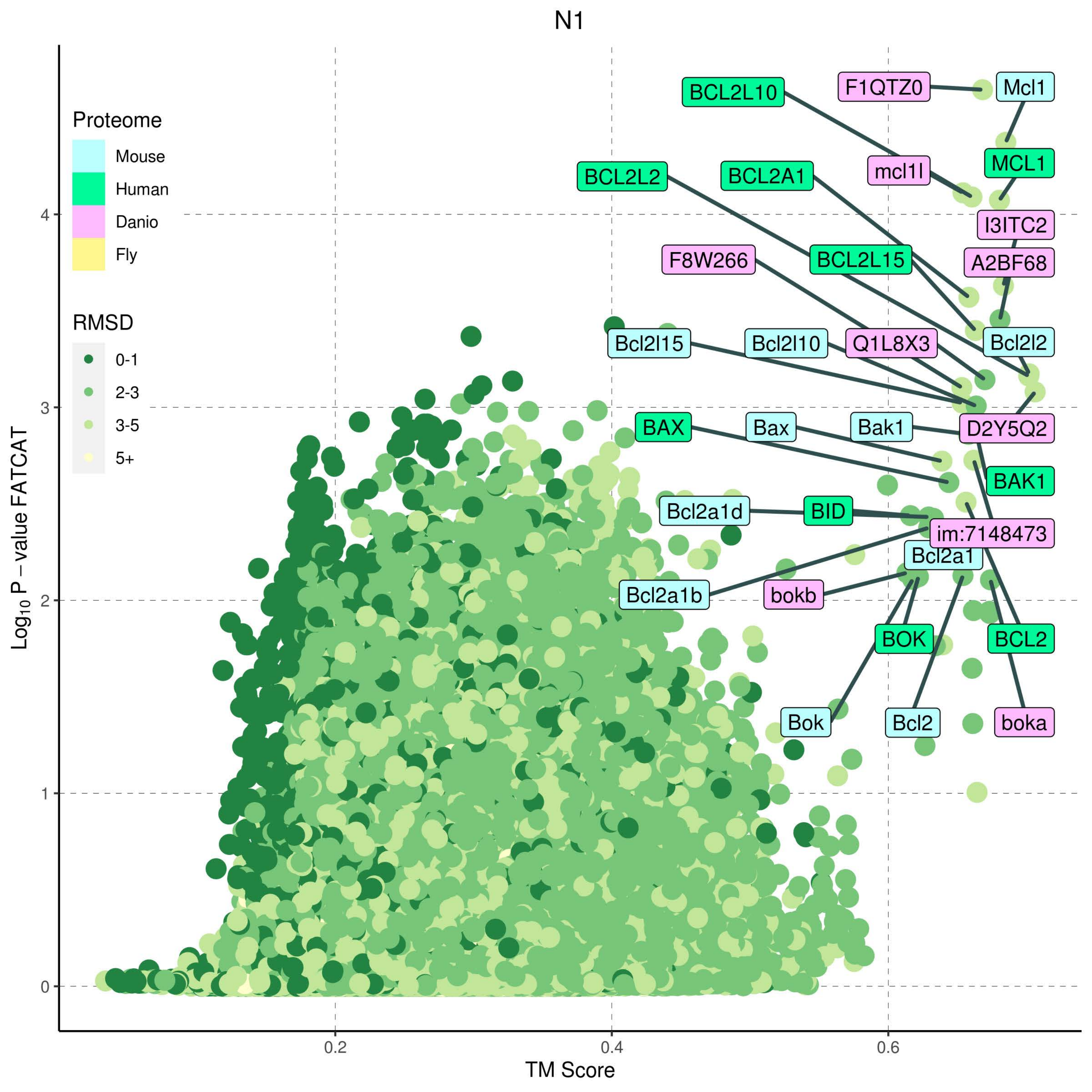
N1
Proteome
Mouse
Human
Danio
Fly
RMSD
0-1
2-3
3-5
5+
F1QTZ0
Mcl1
BCL2L10
mcl1l
MCL1
BCL2L2
BCL2A1
I3ITC2
A2BF68
F8W266
BCL2L15
Bcl2l15
Bcl2l10
Q1L8X3
Bcl2l2
BAX
Bax
Bak1
D2Y5Q2
BAK1
Bcl2a1d
BID
im:7148473
Bcl2a1
Bcl2a1b
bokb
BOK
BCL2
Bok
Bcl2
boka
0
1
2
3
4
0.2
0.4
0.6
Log10 P − value FATCAT
TM Score

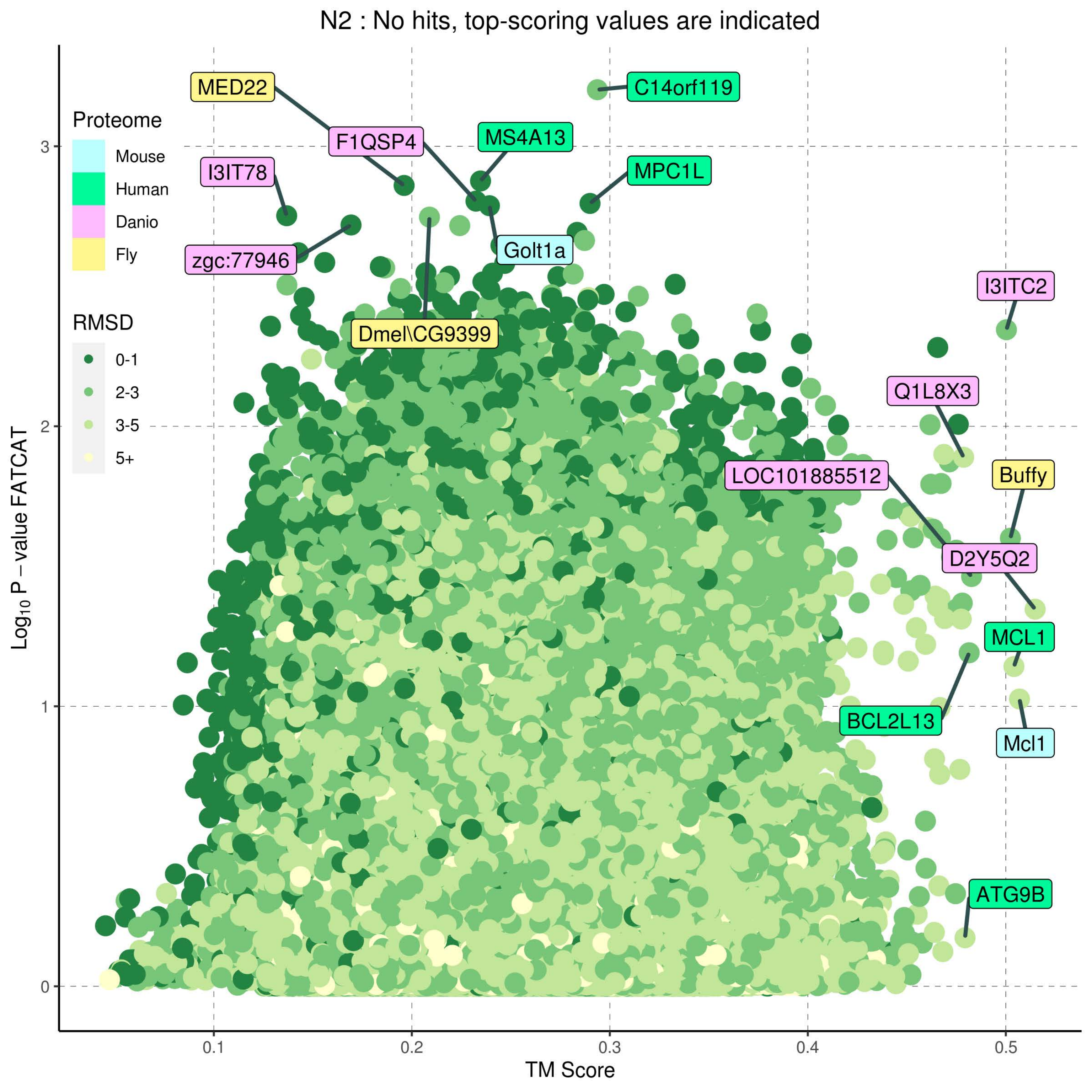
N2 : No hits, top-scoring values are indicated
Proteome
Mouse
Human
Danio
Fly
RMSD
0-1
2-3
3-5
5+
MED22
C14orf119
F1QSP4
MS4A13
I3IT78
MPC1L
zgc:77946
Golt1a
Dmel\CG9399
I3ITC2
Q1L8X3
LOC101885512
Buffy
D2Y5Q2
MCL1
BCL2L13
Mcl1
ATG9B
Log10 P − value FATCAT
TM Score
0
1
2
3
0.1
0.2
0.3
0.4
0.5

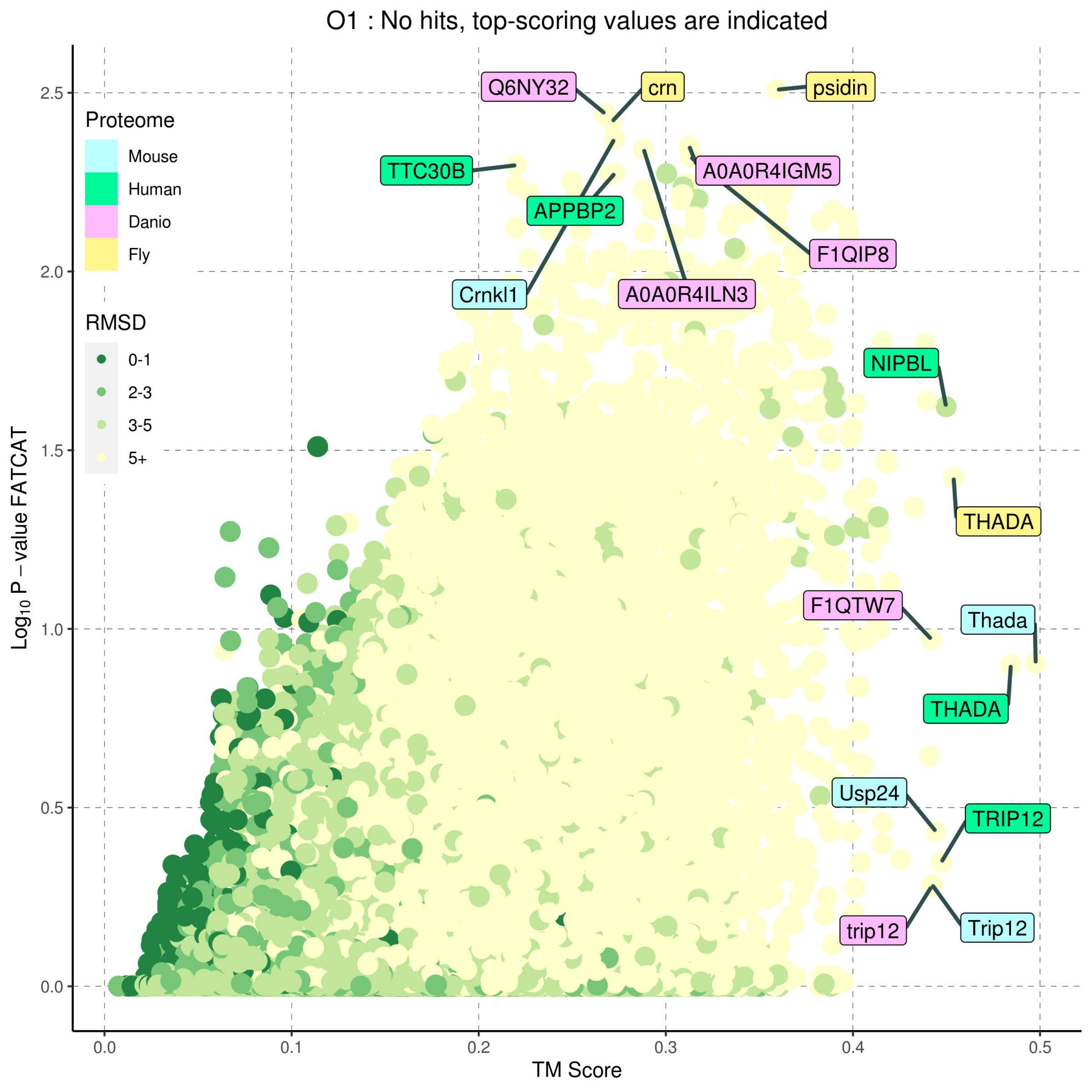

O1 : No hits, top-scoring values are indicated
Proteome
Mouse
Human
Danio
Fly
RMSD
0-1
2-3
3-5
5+
Q6NY32
crn
psidin
TTC30B
A0A0R4IGM5
APPBP2
F1QIP8
Crnkl1
A0A0R4ILN3
NIPBL
THADA
F1QTW7
Thada
THADA
Usp24
TRIP12
trip12
Trip12
Log10 P − value FATCAT
TM Score
0.0
0.5
1.0
1.5
2.0
2.5
0.0
0.1
0.2
0.3
0.4
0.5

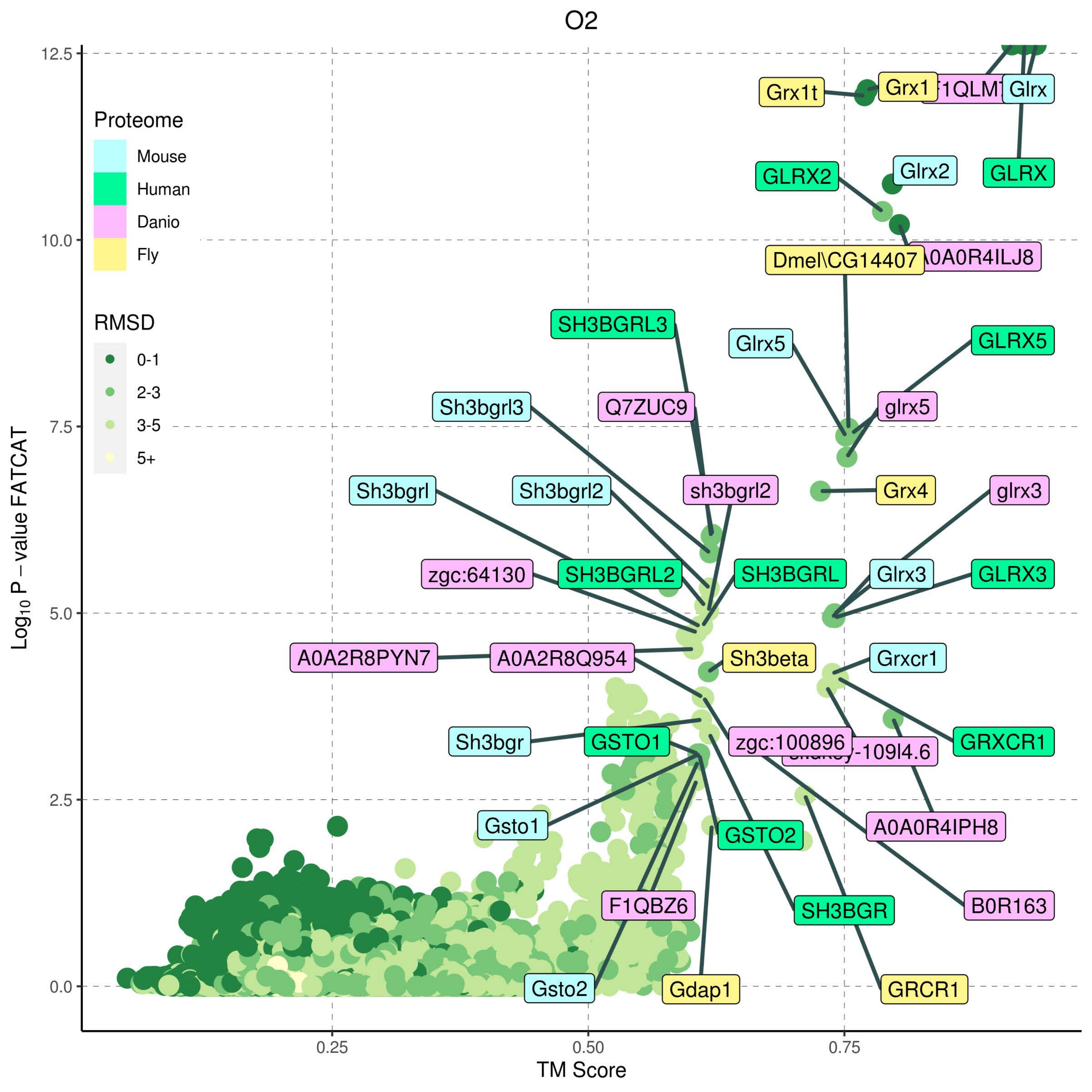

O2
Proteome
Mouse
Human
Danio
Fly
RMSD
0-1
2-3
3-5
5+
Log10 P − value FATCAT
TM Score
0.25
0.50
0.75
0.0
2.5
5.0
7.5
10.0
12.5
Grx1t
Grx1
Glrx
GLRX2
Glrx2
GLRX
Dmel\CG14407
SH3BGRL3
Glrx5
GLRX5
Sh3bgrl3
Q7ZUC9
glrx5
Sh3bgrl
Sh3bgrl2
sh3bgrl2
Grx4
glrx3
zgc:64130
SH3BGRL2
SH3BGRL
Glrx3
GLRX3
A0A2R8PYN7
A0A2R8Q954
Sh3beta
Grxcr1
Sh3bgr
GSTO1
zgc:100896
GRXCR1
Gsto1
GSTO2
A0A0R4IPH8
F1QBZ6
SH3BGR
B0R163
Gsto2
Gdap1
GRCR1

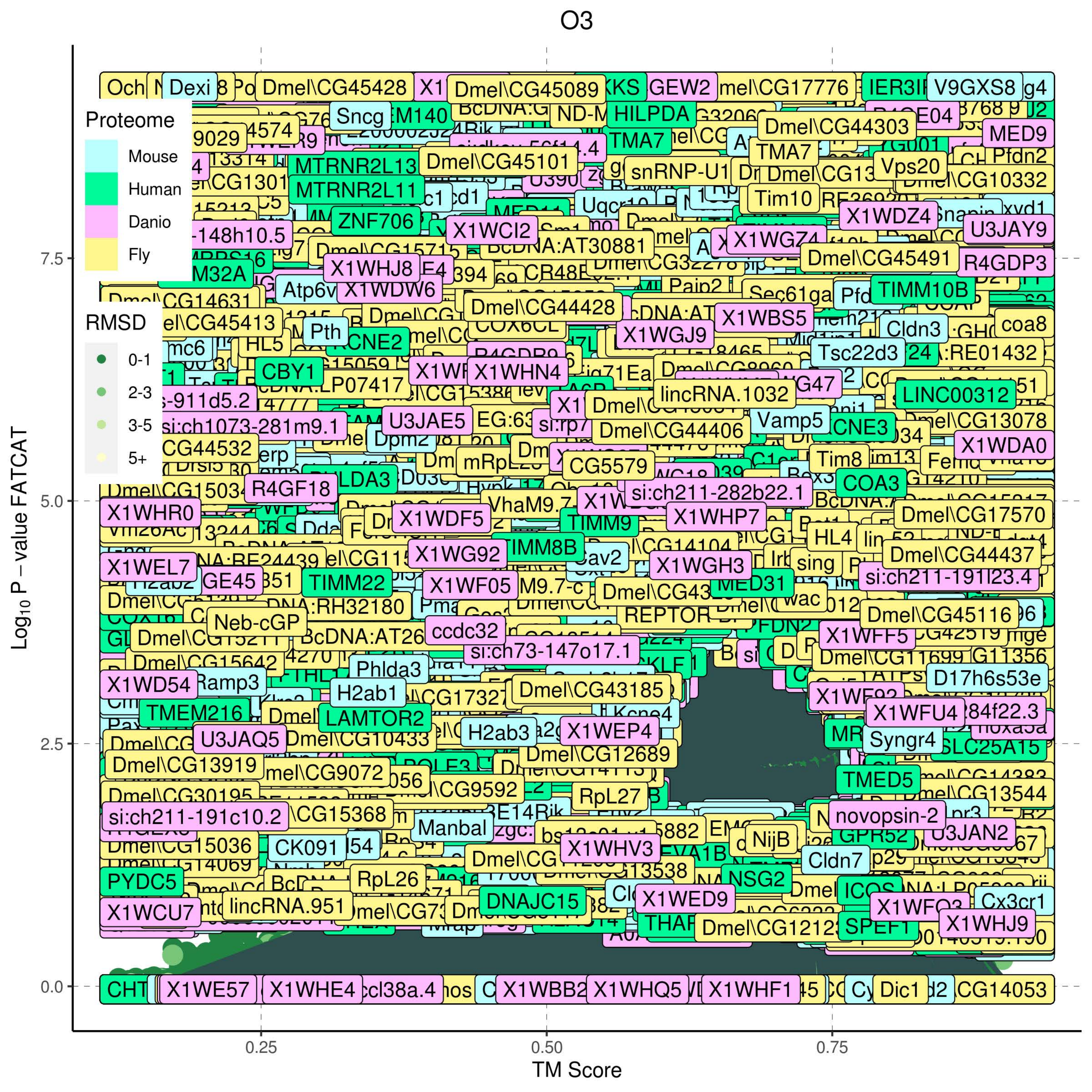
O3
Proteome
Mouse
Human
Danio
Fly
RMSD
0-1
2-3
3-5
5+
Log_{10} P − value FATCAT
TM Score
0.0
2.5
5.0
7.5
0.25
0.50
0.75
Dmel\CG45428
Dmel\CG45089
V9GXS8
HILPDA
Dmel\CG44303
MED9
MTRNR2L13
Dmel\CG45101
MTRNR2L11
ZNF706
X1WCI2
X1WDZ4
U3JAY9
X1WHJ8
X1WDW6
Dmel\CG45491
R4GDP3
TIMM10B
Dmel\CG44428
X1WBS5
X1WGJ9
Cldn3
coa8
Tsc22d3
CBY1
X1WHN4
lincRNA.1032
LINC00312
U3JAE5
Dmel\CG44406
Vamp5
Dmel\CG13078
X1WDA0
CG5579
COA3
R4GF18
si:ch211-282b22.1
X1WHR0
X1WDF5
X1WHP7
Dmel\CG17570
TIMM9
X1WG92
TIMM8B
X1WGH3
Dmel\CG44437
X1WEL7
TIMM22
X1WF05
si:ch211-191l23.4
Neb-cGP
Dmel\CG45116
ccdc32
X1WFF5
si:ch73-147o17.1
X1WD54
Ramp3
Phlda3
H2ab1
Dmel\CG43185
D17h6s53e
TMEM216
LAMTOR2
X1WFU4
H2ab3
X1WEP4
Syngr4
U3JAQ5
Dmel\CG10433
Dmel\CG12689
Dmel\CG13919
TMED5
RpL27
si:ch211-191c10.2
novopsin-2
Manbal
U3JAN2
CK091
X1WHV3
NijB
Cldn7
PYDC5
RpL26
NSG2
lincRNA.951
DNAJC15
X1WED9
X1WCU7
X1WFQ3
Cx3cr1
X1WHJ9
SPEF1
X1WE57
X1WHE4
X1WBB2
X1WHQ5
X1WHF1
Dic1
CG14053

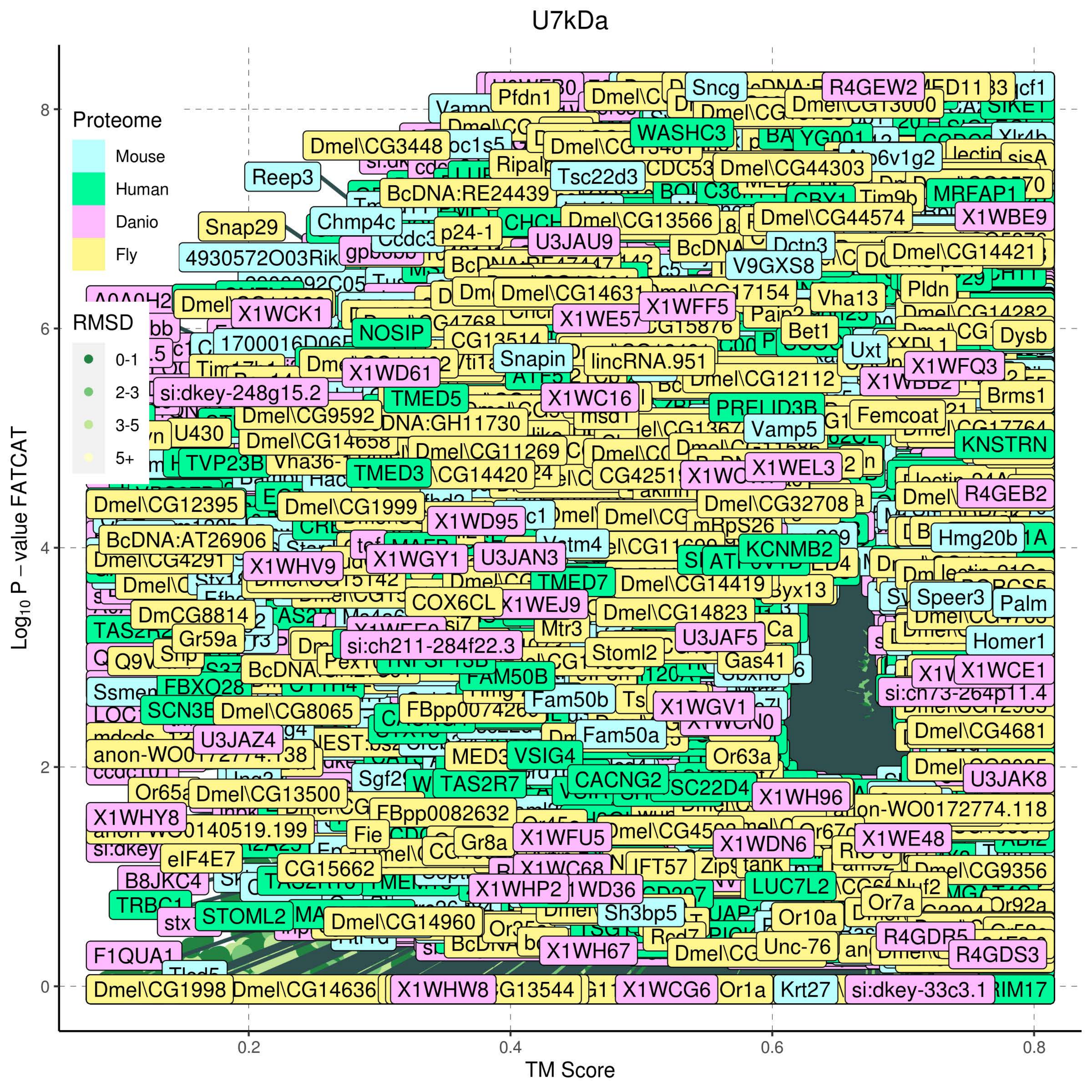

U7kDa
Proteome
Mouse
Human
Danio
Fly
RMSD
0-1
2-3
3-5
5+
Log_{10} P − value FATCAT
TM Score
0
2
4
6
8
0.2
0.4
0.6
0.8
Sncg
R4GEW2
Pfdn1
Dmel\CG3448
Reep3
Tsc22d3
Dmel\CG44303
BcDNA:RE24439
MRFAP1
Chmp4c
Snap29
Dmel\CG13566
Dmel\CG44574
X1WBE9
U3JAU9
Dmel\CG14421
4930572O03Rik
V9GXS8
Dmel\CG14631
X1WFF5
X1WCK1
Vha13
Pldn
NOSIP
Bet1
Uxt
Dysb
Snapin
lincRNA.951
X1WFQ3
X1WD61
si:dkey-248g15.2
TMED5
X1WC16
Dmel\CG12112
Brms1
Dmel\CG9592
Femcoat
Vamp5
U430
Dmel\CG14658
KNSTRN
Dmel\CG11269
TVP23B
TMED3
X1WEL3
Dmel\CG12395
Dmel\CG1999
Dmel\CG32708
R4GEB2
X1WD95
BcDNA:AT26906
Hmg20b
KCNMB2
X1WHV9
X1WGY1
U3JAN3
Dmel\CG4291
TMED7
Dmel\CG14419
COX6CL
X1WEJ9
Speer3
Palm
DmCG8814
Dmel\CG14823
Gr59a
si:ch211-284f22.3
U3JAF5
Homer1
Stoml2
Gas41
X1WCE1
FAM50B
FBXO28
si:ch73-264p11.4
Fam50b
SCN3B
Dmel\CG8065
X1WGV1
Dmel\CG4681
U3JAZ4
Fam50a
VSIG4
Or63a
CACNG2
TAS2R7
U3JAK8
Dmel\CG13500
X1WH96
FBpp0082632
X1WHY8
X1WFU5
X1WDN6
X1WE48
eIF4E7
Gr8a
IFT57
CG15662
Dmel\CG9356
B8JKC4
X1WHP2
LUC7L2
TRBC1
STOML2
Sh3bp5
Or7a
Dmel\CG14960
Or10a
R4GDR5
F1QUA1
X1WH67
Unc-76
R4GDS3
Dmel\CG1998
Dmel\CG14636
X1WHW8
X1WCG6
Or1a
Krt27
si:dkey-33c3.1

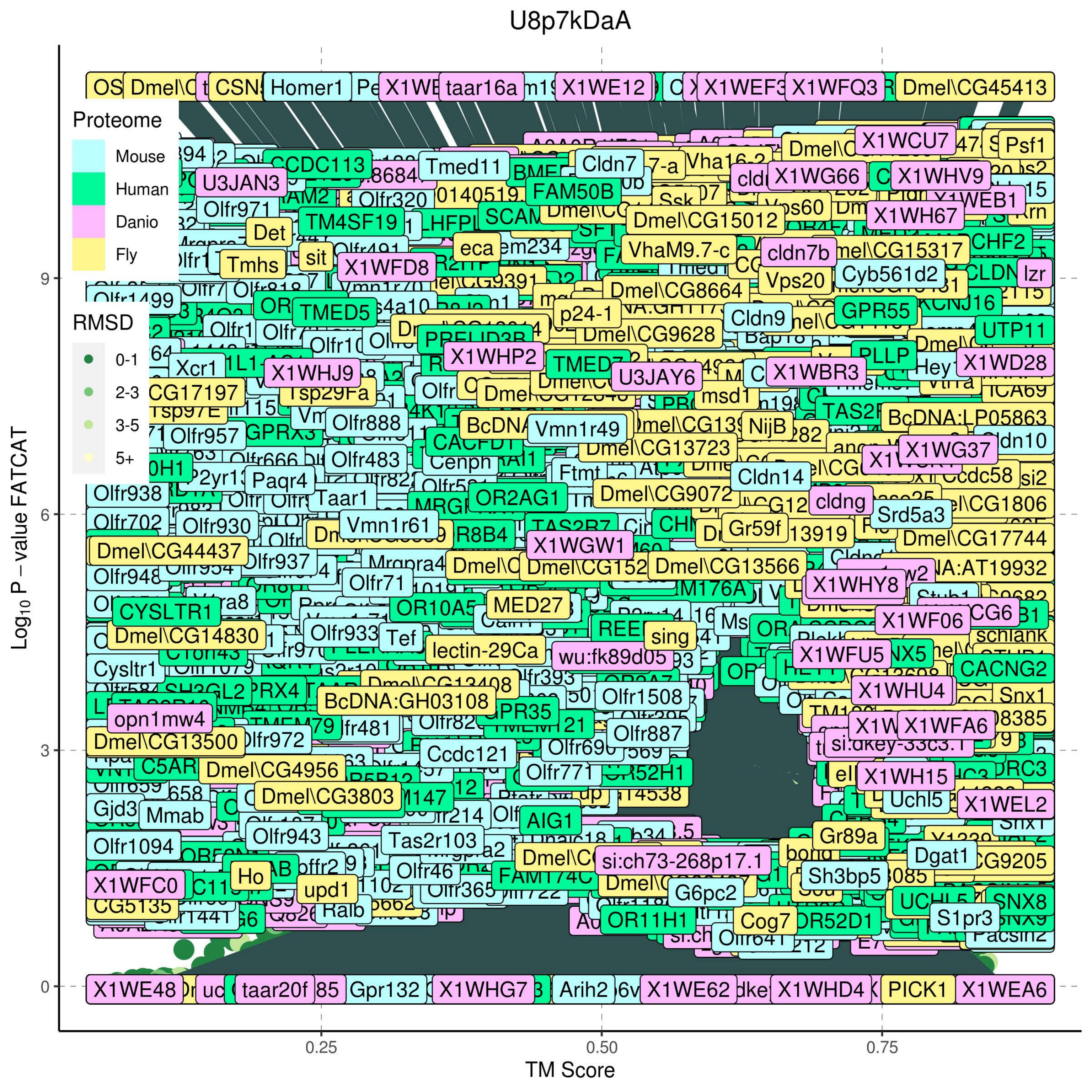

U8p7kDaA
Proteome
Mouse
Human
Danio
Fly
RMSD
0-1
2-3
3-5
5+
Log10 P − value FATCAT
TM Score
0.25
0.50
0.75
0
3
6
9

GSDM alignment

>M35027.1:c153690-152956 Vaccinia virus Copenhagen complete genome
----
MGNKNIKPSKENRLSILSKDKMDSFKRGSWATSSFREKSRATIQRFSSLRREHIKVDH--
PDKFLELKRGIYEIIQKSSSIDVDKRTKLMSNIKTMM--INPFMIEGLMTSLE-----------------NLD--
PDNKMSYSSVMILGEFDIINISDNE-------AAFEFINSLLKSLLLLNTRQLKLLEYSI-SNDLLYAHINA---
LEYIIKNTFNVPER------QLILRGQYLT---PIFSDLLKYAGLTIK--SNILMWNK--QFIKPVSDLYTSIRLLYCVTVX--
----
>AY484669.1:c159516-158782 Rabbitpox virus complete genome

MGNKNIKPSKENRLSILSKDKMDSFKRGSWATSSFREKSRATIQRFSSLRREHIKVDH--
PDKFLELKRGIYEIIQKSSSIDVDKRTKLMSNIKTMM--INPFMIEGLMTSLE-----------------NLD--
PDNKMSYSSVMILGEFDIINISDNE-------AAFEFINSLLKSLLLLNTRQLKLLEYSI-SNDLLYAHINA---
LEYIIKNTFNVPER------QLILRGQYLT---PIFSDLLKYAGLTIK--SNILMWNK--QFIKPVSDLYTSIRLLHCVTVX--
----
>KY349117.1:c166272-165538 Horsepox virus strain MNR complete genome
----
MGNKNIKPSKENRLSILSKDKMDSFKRGSWATSSFREKSRATIQRFSSLRREHIKVDH--
PDKFLELKRGIYEIIQKSSSIDVDKRTKLMSNIKTMM--INPFMIEGLMTSLE-----------------NLD--
PDNKISYSSVMILGEFDIINISDNE-------AAFEFINSLLKSLLLLNTRQLKLLEYSI-SNDLLYAHINA---
LEYIIKNTFNVPER------QLILRGQYLT---PIFSDLLKYAGLTIK--SNILMWNK--QFIKPVSDLYTSIRLLHCVTVX--
----
>MG599038.1:c157906-157148 Buffalopox virus isolate Karachi 2005 complete genome
----
MGNKNIKPSKENRLSILSKDKMDSFKRGSWATSSFREKSRATIQRFSSLRREHIKVDH--
PDKFLELKRGIYEIIQKSSSIDVDKRTKLMSNIKTMM--INPFMIEGLMTSLE-----------------NLD--
PDNKMSYSSVMILGEFDIINISDNE-------AAFEFINSLLKSLLLLNTRQLKLLEYSI-SNDLLYAHINA---
LEYIIKNTFNVPER------QLILRGQYLT---PIFSDLLKYAGLTIK--SNILMWNK--
QFIKPVSDLYTSMRLLHCVTESYKVIGMGX--

>AY243312.1:c154675-153917 Vaccinia virus WR complete genome

MGNKNIKPSKENRLSILSKDKMDSFKRGSWATSSFREKSRATIQRFSSLRREHIKVDH--
PDKFLELKRGIYKIIQKSSSIDVDKRTKLMSNIKTMM--INPFMIEGLMTSLE----------------NLD--
PDNKMSYSSVMILGEFDIINISDNE-------AAFEFINSLLKSLLLLNTRQLKLLEYSI-SNDLLYAHINA---
LEYIIKNTFNVPER------QLILRGQYLT---PIFSDLLKYAGLTIK--SNILMWNK--
QFIKPVSDLYTSMRLLHCVTESYKVIGMGX--

>KC813500.1:c153312-152578 Cowpox virus strain MonKre08/4 complete genome
----
MGNKNIKPSKENRLSILSKDKMDSFKRGSWATSSFREKSHATIQRFSSLRREHIKVDH--
PDKFLELKREIYEIIQKSSSIDVDKRTKLMSNIKTMM--INPFMIEGLMTSLE----------------NLD--
PDNKMSYSSVMILGEFDIINISDNE-------AAFEFINSLLKSLLLLNTRQLKLLEYSI-SNDLLYAHINA---

LEYIIKNTFNVPER------QLILRGQYLT---PIFSDLLKYAGLTIK--SNILMWNK--
KFIKPVSDLYTSMRLLHCVTVX---

>NC 055231.1:c163765-163031 Orthopoxvirus Abatino complete genome
----
MGNKHIKPSKENRLSILSKDKMDSFKRGSWATSSFREKSHATIQRFSSLRREHVKVDH--
PDKFLELKREIYEIIQKSSSIDVDKRTKLMSNIKTMM--INPFMIESLMTSLE-----------------NLD--
PDNKMSYSSVMILGEFDIINISDNE-------AAFEFINSLLKSLLLLNTRQLKLLEYSI-SNDLLYAHINA---
LEYIIKNTFNVPER------QLILRGQYLT---PIFSDLLKYAGLTIK--SNILMWNK--
KFIKPVSDLYTSMRLLHCVTVX---

>MN912466.1:c156293-155559 Ectromelia virus WH complete genome
----
MGNKNIKPSKENRLSILSKDKMDSFKRGSCATSSFREKSHATIQRFSSLRREHIKVDH--
PDKFLVLKREIYEIIQKSSSIDVDKRTKLMSNIKTMM--INPFMIEGLMTSLE-----------------NLD--
PDNKMSYSSAMILGEFDIINISDNE-------ATFEFINSLLKSLLLLNTRQLKLLEYSI-SNDLLYANINA---
LEYIIKNTFNVPER------QLILRGQYLT---PIFSDLLKYAGLTIK--SNILMWNK--
KFIKPVSDLYTSMRLLHCVTVX---

>MH607143.1:c167386-166652 Akhmeta virus isolate Vani 2010 complete genome
----
MGNKHIKPSKENRLSILYKDRMDSFKRGSWATSSFREKSHATIQRFSSLRREHVKVDH--
PDKFLELKREIYVIIQKSASIDVDKRTKLMSNIKTMM--INPFMIEGLMSSLE-----------------NLD--
PDNKMSYSSVMILGEFDIINISDNE-------KAFEFINSLLKSLLLLNTRQLKLLEYSI-SNDLLYAHINA---
LEYIIKNTFNVPER------QLILRGQYLT---PIFSDLLNYAGLTIK--SNILMWNK--
KFIKPVSDLYTSMRLLHCVTVX---

>DQ437594.1:c153307-152573 Taterapox virus strain Dahomey 1968 complete genome
----
MGNKNIKPSKENRLSILYKDRMDSFKRGSWVTSSFREKSHATIQRFSSLRREHVKVDH--
PDKFLELKREIYAIIQKSSSIDVDKRTKLMSNIKTMM--INPFMIEGLMTSLE-----------------SLD--
PDNKMSYSSVMILGEFDIINISDNE-------AAFEFINSLLKSLLLLNTSQLKLLEYSI-SNNLLYTHINA---
LEYIIKNTFNVPER------QLILRGQYLT---PFFSDLLKYAGLTIK--SNILMWNK--
KFIKPVSDLYTSMRLLHCVTVX---

>KY358055.1:c145385-144651 Variola virus strain VD21 17th century complete genome
----
MGNKNIKPSKENRLSILYKDRMDSFKRGSWATSSFREKSHATIQRFSSLRREHVKVDH--
PDKFLELKREIYAIIQKSSSIDVDKRTKLMSNIKTMM--INPFMIEGLMTSLE-----------------SLD--
PDNKMSYSSVMILGEFDIINISDNK-------AAFEFINSLLKSLLLLNTSQLKLLEYSI-SNDLLYTHINA---
LEYIIKNTFNVPER------QLILRCQYLT---PIFSDLLKYAGLTIK--SNILMWNK--KFIKPVSDLYTSMRLLHCVTVX-
----
>DQ441432.1:c146026-145292 Variola virus strain Korea 1947 (Lee Masterseed) complete genome

----
MGNKNIKPSKENRLSILYKDRMDSFKRGSWATSSFREKSHATIQRFSSLRREHVKVDH--
PDKFLELKREIYAIIQKSSSIDVDKRTKLMSNIKTMM--INPFMIEGLMTSLE-----------------SLD--
PDNKMSYSSVMILGEFDIINISDNK-------AAFEFINSVLKSLLLLNTSQLKLLEYSI-SNDLLYTHINA---
LEYIIKNTFNVPER------QLILRCQYLT---PIFSDLLKYAGLTIK--SNILMWNK--
KFIKPVSDLYTSMQLLHCVTVX--

>DQ441437.1:c146904-146170 Variola virus strain Sierra Leone 1969 (V68-258) complete genome

----
MGNKNIKPSKENRLSILYKDRMDSFKRGSWATSSFREKSHATIQRFSSLRREHVKVDH--
PDKFLELKREIYAIIQKSSSIDVDKRTKLMSNIKTMM--INPFMIEGLMTSLE-----------------SLD--
PDNKMSYSSVMILGEFDIINISDNK-------AAFEFINSLLKSLLLLNTSQLKLLEYSI-SNDLLYTHINA---
LEYIIKNTFNVPER------QLILRCQYLT---PIFSDLLKYAGLTIK--SNILMWNK--
KFIKPVSDLYTSMQLLHCVTVX--

>MN240300.1:c166555-165827 Alaskapox virus complete genome

MGNKNGKPFTENIQFLKDKRDSFKRGTWATSSFREKSHATIQRFSSLRRENVKVDQ--
HDKYMELKREIYAIIQKSSSIDINKRTKLLSNIKMMM--INPFMIEGLMTSLE-----------------NLD--
PDNKMSYSSVMILGEFDIINISDNE-------TAFEFINNLLKSLLLLNNRQLKLLEYSI-SNDLLYAHINA---
LEYIIKNTFNVPER------QLILRGQYLT---PIFSDLLKYAGITIK--SNILMWNK--KFIKPVSDLYTSMRLLHCVTVX-
----

>KU749310.1:c167631-166873 Skunkpox virus strain WA complete genome

----
MGNNIKPSKEDKQLSSKGNRQSILFKDRMDSFKRGSWATSSFREKSVTTIQKFSSLRREHTKIEQ-
PDSKFIELKREIYEIINKSSSIDIDKRLMLIYNIKKMM--VQPFMLEGLMNSLE-----------------KMD--
PEDMISYSSVMILGEFDIVNIYDNK-------STFQFINGLLKSLHALNSKQLKLLEYSI-SNDLLYNQVTA---
LEYVIKNTFDVPER------QLILRGQYLT---PIFNDLLKYSGLTIK--SNILMWNK--KFIKPVSDLYTAIRLLYCVTIX--
----

>KU749311.1:c169057-168281 Volepox virus strain CA complete genome

----
MGNNIKPSKENRQSLSKENRQSLKKNRQSFLFNDGMDTFKRGSWATSSFREKSASTIHKFSSLRREHTKIDQP
-DSKFIELKREIYEIINKSSSIDIDKRIMLVYNIKKMM--VNPFMIEGLMNSLE-----------------KMD--
PEDVISYSSVMIFGEFDMVNIHNK--------SAFQFINSLLKSLHALNNKQLKLLEYSI-SNDLLYNQVTA---
LEYVIKNTFDVPER------QLILRGQYLT---PIFNDLLKYSGLTIK--SNILMWNK--KFIKPISDLYTAIRLLYCVTVX--
----

>KU749309.1:c145369-144644 Raccoonpox virus strain 85A complete genome

----
MGNNIKSSKENRQSGLFKDRMDSFKRGSWATSSFREKSVPTIHKFSSLRRERVTVQP--
DSKFIELKREIYAIINKSSSIDIDKRIILISNIKKMM--VNPFMIEGLMNSLE-----------------KMD--
PEDMISYSSVMILGEFDINMNDA---------PSFQFIISLLKSLHALNNKQLKLLEYSI-GNDLLYSQVTA---
LEYVIKNTFDVPER------QLILRGKYLT---PIFNDLLKYSGLTIK--SNILMWNK--KFIKPVSDLYIAIRLLYCMTIX--
----

>AY009089.1:c154402-154034 Camelpox virus CMS complete genome
----
-------------MM--INPFMIEGLMTSLE-----------------SLD--PDNKMSYSSMMILGEFDIINISDNE-------
VAFEFIHSLLKSLLLLNTSQLKLLEYSI-SNDLLYTHINA---LEYIIKNTFNVPER------QLILRGQYLT---
PIFSDLLKYAGLTIKX--

>MK910851.1:c154204-153836 Camelpox virus strain Negev2016 complete genome
----
-------------MM--INPFIIEGLMTSLE-----------------SLD--PDNKMSYSSMMILGEFDIINISDNE-------
VAFEFIHSLLKSLLLLNTSQLKLLEYSI-SNDLLYTHINA---LEYIIKNTFNVPER------QLILRGQYLT---
PIFSDLLKYAGLTIKX--

>MF001305.1:c159640-158771 NY 014 poxvirus strain 2013 complete genome
---
MGNKHSEPVFSESTISYTKRSMRVPCNEKPSTELKTGFNRLSLREKTVSSLQRGFNRLSLNEKTFSLSRGFNNR
LSLNEKTFSLQRGFSLRRFNSVRGES-SNSKLMELKKEIYETIQQANDMDIDKRIKLLYNIKSMM--
GNSFMLKGLIDSLE-----------------TLE--PTDGISYSSVMILGEFSILDQYTDGGK-----
STFKFVINLLKSLDSLNHHQQKLIEYAV-NNDILYDQITM---IEYIMKNNLDMENR------QFILKGKYTT---
PLFIDLLKHTGINV--ISNVLIWNK--KYTKSISNLYTAMRLLYSITVX--

>MF001304.1:c160603-159728 Murmansk poxvirus strain LEIV-11411 complete genome
MGNKHSEPVFSASTISYTKRSSIRRVPCNENPSTELKTGEFNRLSLREKTVSSLQRGFNRLSLNEKTFSLHRGFN
NRLSLNEKTFSLQRGFSLRRFNSVRGES--SSKLMELKKEIHETIQQANNMDIDKRIKLLYNIKSMM--
GNSFMLKGLIDSLE-----------------TLE--PTDGISYSSVMILGEFSILDQYTDGGK-----
STFKFVINLLKSLDSLNHHQQKLIEYAV-NNDMLYDQITM---LEYIMKNNLDMENR------QFILKGKYTT---
PLFIDLLKHTGINV--ISNVLMWNK--KYTKSISNLYTAMRLLYSITVX--

>HQ849551.1:c134784-134035 Yoka poxvirus strain DakArB 4268 complete genome
----
MGNKNSDPVFTSTSCNNTTSLKRGQSLHLHRDLDKLSLNENTLFNRNFSFRRVNSMRIKPSNYSKLIELKKEIF
ETIKKANDMDIDKRIKLLHNIKSII--TNPFVLKGLIESLE-----------------TFE--
SVDGIKYSSVMILGEFNILDQYTGT-------STFQFVIDLLKSLYVLNNKQQKIIEYAI-NNDMLYEQIEM---
IEYIMTNVLDIDNY------HFILKGKYVT---PLFVELLKNTGINV-ISNNIIIWNK--KYTKNISNLYKAIRLLHSITVX--
----
>KU749310.1:172437-172985 Skunkpox virus strain WA complete genome
----
MQNSYIGQIYTRCHAILLIPDEYKHKFIKSLTHLI--GYNGRTYNRCT-------------------------
RDAITELTDIANEIDNT---------------DVKLITTFIISSIYKITRFQSFLVELAM-YHKVLDKIQHI---
MEYIIDNHSRIDNNGFTPYSDNDPDFNQVIA--
CIIYNLLRSSGMEINYSKYKAWWNPRHDYVDTFGLLYVIFTILSIIDDNX---

>KU749309.1:150174-150722 Raccoonpox virus strain 85A complete genome
----
MRDSYICQIYTRCHSILSMPDDYKYKFIKSLNHLI--RYNGRTYNRCT--------------------------

RDAITELSDIANSIDDT---------------DIKIITTFIISSIYKITRFQSFLVELAM-HHKVLDKVQHI---
MEYIIDNHSHSNTNGFTPYSIDDPDFNQVVA--
CIIYNLLRSSGMDINYSKCKAWWNPRHDYVDTFGLLYVIFTILSIIGDKX--

>KU749311.1:173861-174445 Volepox virus strain CA complete genome
----
MLNDYIGQIYIRCRAILSIPDDYKHKFIKSLTHLI--RYNGRTYNRCT------------------------
RDAITELSDIANNIGNN---------------DVKIITTFIISSIYKITRFQSFLVELAM-YHKVLDKIQHI---
MEYIIENHSHNDTNGFTPYSTDDTNFNQVIA--
CIIYNLLKSSGMEINYSECKAWWNPRHDYVDTFGLLYIIVTILLIIDGDQYNFYNKLEKNKX----------------------
----
>MF001304.1:c165763-165224 Murmansk poxvirus strain LEIV-11411 complete genome
--M--
LSVPSQYKDLLQEYYDIITSVPDEYRDIFVEYLIYLI--EYDGRISNKRT------------------------
RDIKKELINIVGKVSDKT--------------RLMNFISFITESICRLTRFQSFLLELAS-YHNVLDEVQEI---
MGDIIENHCRMEIIGFTTYDINSIVL-------SIIYNMLKTSGMRVDYDTSKAWWNP--
EYRDMFGSLYMIVTTLTILKNIX--

>MF001305.1:c164808-164269 NY 014 poxvirus strain 2013 complete genome
----
MLSVPSKYKDLLQEYYDIITSVPDEYRDIFVQYLIYLI--EYDGRISN---------------------------
KRTRDIKKELINIVGKVSDKT-----------RLMNFISFITESICRLTRFQSFLLELAS-YHDVLDEVQEI---
MGDIIKNHCRMEIIGFTAYDINSIVL-------SIIYNMLKTSGMRVDYDTSKVWWNP--
EYSDMFGSLYMIVTTLTILKNIX--

>NP 051846.1 m132L Myxoma virus
---MGNLLYCCFTV--
EKTVPSIQRYVNGQVNLDNFLARHRKRSILLYLLKVI--RDDKLLNEVC----------------------------
RVKKKYSVYLYGCKNPNA-----------EVVNFINYILVYLNALSCTQKRLLYIII-AENMISSFIGY---
INTVFTSNAVKFKDSE-----------------RCIEELFIETGLYI---SPQGSYKKHTEVMEELIYLYILARLFKQLL-----------
----
>NP 052021.1 gp132L Rabbit fibroma virus
---MGNLLYCCFVV--
DVTTPSIQRYVNEQVNLDKFLSRRKKKSILLYLLKVI--RNDKLLTEVYC------------------------------
SKKYSELYGCKNPNT-----------EVVNFINYILIYLNALSYTQKRLLYIII-AENMITSFIEY---
INAVFTSSSTVKFKDSE----------------RCIEELFIETGLYISSSQGYNKQHT--EVMEELIYLYILAKLFKQLL---------
----
>YP 009408097.1 Immunoprevalent protein Eptesipox virus
---MGNSTSVSKSCNNAVTHVSANSIRQHIL--
FNNFETLHKDIQSKIDLVNTFTPQTKNLIFRNLLIVI--TNSYHLQNLLDALE-----------------
QLEPMYVTDAYSEAILNEIGLCDKGIPNL--------SSIHFMIYLVSGLTKLTTKQSKILMEIVTDAKIFCHHVNV---
LEYIIKKNVEKLETVTSTLLEKYTKL-------PLEVTLFKESGLKIQ--GNTYIWDP--EHKKSICNLYTVIKIMSYIM----
----

>ref|NP 835465.2| chromosome 17 organism Homo sapiens gcode 1 chromosome 17 map 17q21.1 gasdermin-A Homo sapiens
---GDV--
HEGFRTLKEEVQRETQQVEKLSRVGQSSLLSSLSKLL--GKKKELQDLELALEG----------------ALD--
KGHEVTLEALPKDVLLSK--------------EAVGAILYFVGALTELSEAQQKLLVKSM-EKKILPVQLKL---
VESTMEQNFLLDKEGVFPLQPELLSSLGDEEL-TLTEALVGLSGLEVQRSGPQYMWDP--
DTLPRLCALYAGLSLLQQLTKAS--

>ref|XP 015154998.2| location chromosome chromosome 27 completeness complete organism Gallus gallus isolate bGalGal1 gcode 1 chromosome 27 sex female tissue type blood country USA: Fayetteville lat ls
--DGFA--
HTEVEALEEEVKEKCGILCQLAGELNAIVLKTIKAVM--RDRNLLEELSGKMEAVLD-DPDNCE------
LTTQSPDLEVLLSTLKGSPRCDLL-------------SLTEAISYTLDAFSELTEDQLLLLLESL-EVEIVPQQLKL---
VRSILEQSAELGAA------AGLLSSFSEQQQQLTTAVLELSGVELQGSGAAVCTE---
ESFPSLSALYAALHALHCLVGAGGRSARAD---

>ref|XP 024836266.1| location chromosome chromosome 19 completeness complete organism Bos taurus isolate L1 Dominette 01449 registration number 42190680 breed Hereford gcode 1 chromosome 19 sex fem...
---GAV--
HEDFRTLKEEVQRETQEVEKLSPEGKSSLLSSLSKLL--GKKKELQDLELTLEE----------------ALG--
KGHEETLEALPKNVLLSK--------------GAMDAILYFLGALAELSEAQQKLLVKSM-EKKILPVQLKL---
VESTMEQNFLQDKEGVFPLRPELLSSLGEEEL-TLTEALVGLSGLEVQRSGPQYTWDP--
DTLPRLCALYAGLSLLRLLTKAS--

>ref|XP 014975037.1| location chromosome chromosome 16 completeness complete organism Macaca mulatta isolate AG07107 bio material Coriell:AG07107 gcode 1 chromosome 16 sex female tissue type fibroblaa
---GDV--
HEGFGTLKEEVQRETQQVERLSQAGQSSLLSSLSKLL--GKKKELQDLELALEG----------------ALD--
KGHEVTLEALPKDVLLSK--------------EAVGAILYFVGALTELSEVQQKLLVKSM-EKKILPVQLKL---
VESTMEQNFLQDKEGVFPLHPELLSSLGEEEL-TLTEALVGLSGLEVQRSGPQYMWDP--
DTLPRLCALYAGLSLLQQLTKAS--

>ref|XP 001171222.1| location chromosome chromosome 17 completeness complete organism Pan troglodytes isolate Yerkes chimp pedigree *C0471 (Clint) gcode 1 chromosome 17 sex male tissue type blood devs
---GDV--
HEGFRTLKEEVQRETQQVEKLSRVGQSSLLSSLSKLL--GKKKELQDLELALEG----------------ALD--
KGHEVTLEALPKDVLLSK--------------EAMGAILYFVGALTELSEAQQKLLVKSM-EKKILPVQLKL---
VESTMEQNFLLDKEGVFPLQPELLSSLGDEEL-TLTEALVGLSGLEVQRSGPQYMWDP--
DTLPRLCALYAGLSLLQQLTKAS--

>ref|XP 024429158.1| chromosome Unknown completeness complete organism Desmodus rotundus isolate DRU21DN04 gcode 1 chromosome Unknown sex male tissue type muscle/skin sample dev stage adult country Us
--GEV--
HQDFRMLKEEIQRETQEVEKLSRMGQSSLLTSLSKLL--GKKKELQDLELTLEG----------------ALD--
KGHEVALEALPKDVLLSK--------------EVVGAILYFLGALTELSEAQQKLLVKSM-EKKILPVQLKL---
VESIMEQNFLLDKEGVFPLQPNLLSCLGEEEL-TFTEALVGLSGLEVQRSGPQYMWDP--
DTLPDLCALYAGLSFLQLLTKAS--

>ref|XP 016020091.2| chromosome Unknown completeness complete organism Rousettus aegyptiacus isolate mRouAeg1 gcode 1 chromosome Unknown sex male tissue type muscle dev stage adult country USA: Berkes
--GEV--
HEDFRTLKEEIQRETQEVEKLSHLGQSSLLTSLSKLL--GNRKELQDLELTLEG----------------ALG--
KGHEVTLEALPKDVLLSK--------------EALGAILYFLGALTELSEVQQKLLVKSM-EKKILPVQLKL---
VESTMEQNFLQDKEGVFPLQPDLLSSLGEEDL-TLTEALVGLSGLEVQRSGPQYTWDP--
DTLPHLCALYAGLSFLQLLTKAS--

>ref|XP 006924750.1| chromosome Unknown completeness complete organism Pteropus alecto gcode 1 chromosome Unknown sex male tissue type kidney country Australia: Anglican Church Grammar School East Bro
--GEV--
HEDFRTLKEEIQRETQEVEKLSHVGRSSLLTSLSKLL--GNRKELQDLELTLEG----------------ALG--
KGHEVTLEALPKDVLLSK--------------EALGAILYFLGALTELSEVQQKLLVKSM-EKKILPVQLKL---
VESTMEQNFLQDKESVFPLQPDLLSSLGEEDL-TLTEALVGLSGLEVQRSGPQYTWDP--
DTLPCLCALYAGLSLLQLLTKAS--

>ref|XP 035935879.1| chromosome Unknown completeness complete organism Halichoerus grypus isolate 241Hg ecotype Northwest Atlantic gcode 1 chromosome Unknown sex male tissue type blood dev stage molts
--KEE--
HGDFKTLKEEVQREIQEVKKLSRVGQSSLLTSLSKLL--GKKKELQDLELMLEG----------------ALD--
KGHEVTPEALPKDVLVSK--------------EAMGGILYFLGALIELSEAQQKLLIKSV-EKKILPMQLKL---
VESTMEQSFLQDKDGVFPLQPDLLSSLGEEEL-TLTEALVGLSGLEVQRSGPQYTWDP--
DTLPRLCALYAGLSLLQLLTKAS--

>ref|XP 004633937.1| chromosome Unknown completeness complete organism Octodon degus isolate 3935 gcode 1 chromosome Unknown sex female gasdermin-A Octodon degus
--GEV--
HEDFKTLKEEVRRETQEVDKLSRAGQGSLLSSLSNLL--GKKKELQDLELTLEG----------------ALD--
KGHEVTLEALPKDVLLSK--------------ESMGAILYFLGALTVLSEAQQKLLVKSV-EKKLLPVQLKL---
VESTLEQNFLQDKEGVFPLRPDLLSSLGEEEL-TLTEALVGLSGLEVQRSGPQYTWDP--
DTLPRLCALYASLSLLQLLARSP--

>ref|XP 029428328.1| location chromosome chromosome 12 completeness complete organism Rhinatrema bivittatum gcode 1 chromosome 12 gasdermin-A Rhinatrema bivittatum
--DYLRILENF----
QEGFPAVMAQIEKECVEFTLLSPEVAEQFLAAFLLLI--KDPKTSADVESRLEQSLY-C-SARESKA---HPE--
PSIARLLGYLGLSSRPVVI-------------PLVRAVLFFLDAVSELDEDGTSLLVESV-ERQDTGQQLKR---
VQKVLEEELFSVRQGSL------------------------AGDEAAGGVSPEGWSP--AVQSCAMALYAALYALHVLSSC--
----
>XP 032891479.1 gasdermin-A Amblyraja radiata
---SLFC--
QDTANVNGDEVFEICCELQCLDKGLKQRLLGLVCQII--EDSELLSILSDVLCEA-C-A-GTDYTLSELEKLD--
KKRRECGENLLAIWSEDQLKNAVED-------NLLKAVSILFAALEDLPPTTLPVLIQSL-KMQILPQQLTL---
VTGILKDLDHSQVDQALKVETE---SFTEDAF-GITAEMLTDVGLHLERDSVQKTREP--S----
LHELSVALYCLNALSSN--

>sp|Q5Y4Y6|GSDA3 MOUSE Gasdermin-A3 OS Mus musculus OX 10090 GN Gsdma3 PE 1 SV 1
---GEM--
HEDFKTLKEEVQRETQEVEKLSPVGRSSLLTSLSHLL--GKKKELQDLEQKLEG----------------ALD--
KGQKVTLEALPKDVLLSK--------------DAMDAILYFLGALTELTEEQLKILVKSL-EKKILPVQLKL---
VESTLEQNFLQDKEGVFPLQPDLLSSLGEEEL-TLTEALVGLSGLEVQRSGPQYAWDP--
DTRHNLCALYAGLSLLHLLSRKSNALTYCALS---

>sp|Q9EST1|GSDMA MOUSE Gasdermin-A OS Mus musculus OX 10090 GN Gsdma PE 2 SV 1
---GEM--
HEDFKTLKEEVQRETQEVEKLSPVGRSSLLTSLSHLL--GKKKELQDLEQTLEG----------------ALD--
KGHEVTLEALPKDVLLSK--------------DAMDAILYFLGALTVLSEAQQKLLVKSL-EKKILPVQLKL---
VESTMEKNFLQDKEGVFPLQPDLLSSLGEEEL-ILTEALVGLSGLEVQRSGPQYTWDP--
DTLPHLCALYAGLSLLQLLSKNS---

>sp|Q32M21|GSDA2 MOUSE Gasdermin-A2 OS Mus musculus OX 10090 GN Gsdma2 PE 2 SV 1
---GEM--
HEDFKTLKKEVQQETQEVEKLSPVGRSSLLTSLSHLL--GKKKELQDLEQMLEG----------------ALD--
KGHEVTLEALPKDVLLLK--------------DAMDAILYFLGALTELSEEQLKILVKSL-ENKVLPVQLKL---
VESILEQNFLQDKEDVFPLRPDLLSSLGEEDQ-ILTEALVGLSGLEVQRSGPQYTWNP--
DTCHNLCALYAGLSLLHLLSRDS---

>ref|NP 001159430.1| chromosome 17 organism Homo sapiens gcode 1 chromosome 17 map 17q21.1 gasdermin-B isoform 3 Homo sapiens
----
-----DVLNSLAKCL--GKEDIRQDLEQRVSEVLI-S-G---------ELHMEDPDKPLLSSLFNAAGVLVE-------------
ARAKAILDFLDALLELSEEQQ-FVAEAL-EKGTLPLLKDQ---VKSVMEQNWD----------------------------------
ELASSPPDMDYDP---EARILCALYVVVSILLELAEGPTSVSS---

>ref|NP 001231146.1| chromosome 19 organism Bos taurus gcode 1 chromosome 19 map 19 gasdermin-B Bos taurus

----
-----EALNCFTKCL--SSEEELQDLEQRVSEVQC-S-G---------ELQMNSPANSLISSLFNAAGALIE-------------
ARAETIWDVLDALMELSEYRQ-FVAEVL-DKGELRPVKDK---VESILEEN------------------------
RCEGPLDVSCD---------------PEARTLCALYVVVSILLQLSEKPTSVSS--

>ref|XP 009430521.1| location chromosome chromosome 17 completeness complete organism Pan troglodytes isolate Yerkes chimp pedigree *C0471 (Clint) gcode 1 chromosome 17 sex male tissue type blood devs
----
-----DVLNSLAKCL--GKEEIRQDLEQRVSEVLISR-----------ELHMEDPDKPLLSSLFNAAGVLVE-------------
VRAKAILDFLDALLELSEEQQ-FVAEAL-EKGTLPLLKDQ---VKSVMEQNWD---------------------------------
ELASSPPDMDCDP---EARILCALYVVVSVLLELAEGPTSVSS---

>ref|XP 023358160.1| location chromosome chromosome 4 completeness complete organism Sarcophilus harrisii gcode 1 chromosome 4 cell line 91H gasdermin-B Sarcophilus harrisii
----
-----AVLDLLTRLL---QDEALGEMEGKVAMALF-T-E---------ELS--DPENPLLSVLFDASGSSMQ-------------
NQAEGILDFLEALTELKEEQQKILPKVV-EKGLLSETMKL---VEDLLRQSETFSLKAD----------------
SITHTLVGICDPEGKLEQP-VTWDS--ETCHSLCALYTVLSVLMLLAQGSVSVP-----------------------------------
----
>ref|XP 024897515.1| chromosome Unknown completeness complete organism Pteropus alecto gcode 1 chromosome Unknown sex male tissue type kidney country Australia: Anglican Church Grammar School East Bro
----
-----DALSCLTKCL--TKDEQLQDLEQRMSEVLISG-----------ELQMDSPAGPLISSLFNAAGILVE-------------
TRTEAIMDFLDAVMELFE-EKELVGEAL-EKGTLPLLKEQ---VESVLDQNGGEQPE------------------------------
-------CVGCDP---EAQILHALYVAVSILLQLSEKPASASS--

>ref|XP 036078993.1| chromosome Unknown completeness complete organism Rousettus aegyptiacus isolate mRouAeg1 gcode 1 chromosome Unknown sex male tissue type muscle dev stage adult country USA: Berkes
----
-----DALSCLSKCL--TKDEQLQDLEQRVSEILISG-----------ELQMDSPAGLLINSLFNASGILVE-------------
TRTEAIMDFLDALMELSE-EKELVAEAL-EKGTLPLLKE----------QNCGEQPE------------------------------------
YVGWDP---EAQVLRTIYAAVSILLLLSEKPASVSC---

>ref|XP 024429114.1| chromosome Unknown completeness complete organism Desmodus rotundus isolate DRU21DN04 gcode 1 chromosome Unknown sex male tissue type muscle/skin sample dev stage adult country Us
----
-----NVLSCLTKCL--TSDGELQALEERVSEVLV-S-E---------ELQVEGPADPLISSLFNAAGILVE-------------
ACIEAILDFLDALKELSEEKE-LVAETL-EKGTLPLLKDQSGGLKSVLEQEWGEQPRG------------------------------
-------VGRDP---EAQTVCTLYLVVSILLQLSEKPAPASSC--

>ref|XP 014975035.1| location chromosome chromosome 16 completeness complete organism Macaca mulatta isolate AG07107 bio material Coriell:AG07107 gcode 1 chromosome 16 sex female tissue type fibroblaa
----
-----DVLNFLTKCL--GREELWQDLEQKISEVLI-F-G---------ELQMEDPVNPLLSSLFNAAGILVG------------
ARAEAILDFLDALLELSEEQH-LVAEAL-EKGTLPLLKDQ---VKPIMEQNWD----------------------------------
ELASSPHDMDCDP---EARIPCALYVVVSVLLELAEGTTSVSS---

>ref|XP 035935913.1| chromosome Unknown completeness complete organism Halichoerus grypus isolate 241Hg ecotype Northwest Atlantic gcode 1 chromosome Unknown sex male tissue type blood dev stage molts
----
DVLSCLRGQLPLSAKVFEVLISGSRHLEMEGLVGLL--ISSLFNAAGFLVK-
------------AHTESILDVLDAL--ITSEEQHLVAEAL-EKGTLPLLKDQ---VRSVLEQNWSEQPH---------------------
-----------------DVGWDP---DAXLLCALYVALSLLLQLGEKPTSVPS---

>ref|NP 113603.1| chromosome 8 organism Homo sapiens gcode 1 chromosome 8 map 8q24.21 gasdermin-C Homo sapiens
----
LLPSFHTISPTLFNASSNDMKLKPELFLTQQFLSGHLPKYEQVHILPVG-RIEEPF----
WQNFKHLQEEVFQKIKTLAQLSKDVQDVMFYSILAML--RDRGALQDLMNMLELDSS-G-----------HLD--
GPGGAILKKLQQDSNHAWF-------------NPKDPILYLLEAIMVLSDFQHDLLACSM-EKRILLQQQEL---
VRSILEPNFRYPWSIPFTLKPELLAPLQSEGL-AITYGLLEECGLRMELDNPRSTWDV--
EAKMPLSALYGTLSLLQQLAEA--

>ref|XP 028708741.1| location chromosome chromosome 8 completeness complete organism Macaca mulatta isolate AG07107 bio material Coriell:AG07107 gcode 1 chromosome 8 sex female tissue type fibroblasta
----
WLPSFHTISPTLFNASPKDMKLKPELFLKPKFLRGHLPKYEQVCILPVG-RIEEPF----
WQNFKHLQEEVFQKIKTLAQLSKDVQDVVFYSILAML--GDRGALQDLMDMLELDSS-G-----------HLD--
GPGGAILKKLQQYSNRAWF-------------NPKDPILYLLEAIMVLNDIQHDLLACSV-EMRILLQQQEL---
VRSILEPNFRYPWSIPFTLKPELLAPLQSEGL-AITYGLLEECGLRMELDNPRSTWDL--
EAKIPLSALYGTLSLLQQLAEA--

>ref|NP 001039469.1| chromosome 14 organism Bos taurus breed L1 Hereford gcode 1 chromosome 14 map 14 gasdermin-C Bos taurus
----
ILSISDDNKRKAFFVGKVQRQSAMGTKRRPILPIGRIQEPI--
SRDFKSLQNEVSREIEAVAELPRDIRDALFHTILAKL--KDQGALQDLTDMLDGNLW-----------------
DHTGSFLSEMREDSRNVWL-------------ESRPCVIYLLEALLVLSDIQHELLAWSM-EKRILPQQREL---
VESILEPNFRYPWNIPFTLDPKLLASLQDEGL-AVTFGLLQECGLRVAPDNPKGTWDL--
EAKKPLSALYGSLSVLQQLAEA---

>ref|XP 031803207.1| location chromosome chromosome 1 completeness complete organism Sarcophilus harrisii gcode 1 chromosome 1 cell line 91H gasdermin-C Sarcophilus harrisii
----
HTEIGLGSMRGMEEKLEWKDYPKLSLFLETIEETQQGFGWKQQDRNNCQR--
VRDFQRLLEEVTFQKKDLVRMPGKQRTTLLCALHDLL--GNRTALQDLEDKLLHVLD-SGIWG-------KME--
GPGNTILSNLQDSSDSPVN-------------GYITGFLYLLEALAALSDNQHDLLAQSL-EKKILSQQLKL---
VKSILETNFNQFHS-TFILPPEILSLIDEEL--NLSLELVEDCGLQLQRTEQMLILDP--
EGKNPLCALYGALSCLLSLSED---

>ref|XP 036088919.1| chromosome Unknown completeness complete organism Rousettus aegyptiacus isolate mRouAeg1 gcode 1 chromosome Unknown sex male tissue type muscle dev stage adult country USA: Berkes
--FRLQLQSFTGGREIQPKSGECEIQILSGRAQRSC-
-WLDFGRLQEEVS-WYVKKAQLLEDMGAVVFSSILAML--GDWEALEGLEDKLEQKPL-G-----------HLD--
GPGGVILNALRESSSYQWF-------------NSEDSILYLLEAINGLSDLQHDLLAWSM-EKKVLLQQRDL---
VRSILEPNFKYPWNIPFTMKPELLAPLRGEAL-AITYGLLEECGLTMEPNSPRSTWDL--
EAKKPLSALYAALSVLQQLAEA---

>ref|XP 024427836.1| chromosome Unknown completeness complete organism Desmodus rotundus isolate DRU21DN04 gcode 1 chromosome Unknown sex male tissue type muscle/skin sample dev stage adult country Us
----
DFKRLHEEVSKRVIAAAELSKDIKGVVFSNILAML--GDRGALQDLVHMLEQEPLKG-----------HLN--
GPGGTILNELQKDSSFAWN-------------GSQYLILYLLEAMMVLSDVQLCLLAPSM-EKKILSQQRDL---
VRSILEPNFKYPWSIPFTLKPELLAPLQGEGL-AITYGLLDECGLKMELHSPRSTWDL--
EAKKPLSALYGTLSVLQQLAEA---

>ref|XP 019678683.1| location chromosome chromosome F2 completeness complete organism Felis catus isolate Cinnamon breed Abyssinian gcode 1 chromosome F2 sex female gasdermin-C Felis catus
----
AVNNGPLYTMSVSDVRKPLDLVLHSPGRIQRVL--WQDFKQLHEEIFQEMEALAQLSKDTQDSIFHTILNML--
GNREALQDLTDTLDGSPL-D-----------LLD--DFGGTILNEMQPDTRDLWI-------------
QARFHIIYLLEVIMVLSDTQHDLLAQSM-EKRILLQQQKL---VRSILEPNFKYCWDIPFTLKPELLAPLQGEDL-
AITYGLLEECGLKMELNSPRSTWDL--ETKQPLSALYGTLSLLQQLTGSPERPGPTPVYAESVPDELGGV---------
----
>ref|XP 001153860.1| location chromosome chromosome 8 completeness complete organism Pan troglodytes isolate Yerkes chimp pedigree *C0471 (Clint) gcode 1 chromosome 8 sex male tissue type blood dev ss
----
LLPSFHTISPTLFNASSNDMKLKPELFLTQQFLSGHLPKYEQVHILPVG-RIEERF----
WQNFKHLQEEVFQKIKTLAQLSKDVQNVVFYSILAML--RDRGALQDLMNMLELDSS-G-----------HLD--
GPGGAILKKLQQDSNHAWF-------------NPKDPILYLLEAIMVLSDFQHDLLACSV-EKRILLQQQEL---
VRSILEPNFRYPWSIPFTLKPELLAPLQSEGL-AITYGLLEECGLRMELDNPRSTWDV--

EAKMPLSALYGTLSLLQQLAEA--

>ref|XP 021073018.1| location chromosome chromosome 17 completeness complete organism Mus pahari gcode 1 chromosome 17 sex female tissue type tail gasdermin-C Mus pahari
---RPMYLSGHFEILRHHEEIIRIGSRIEYISPIGTIEEPT--
HLDFQCLQNEVSQKTMLLAELSKDVQEVVFSSFLHMLCEGDSEVLYDLMKMLELNQL-E-----------HMD--
GPGGKILEELQKDRSPSWI-------------DLKDLILNLLQALMVLSNTQLSLLAQSV-EMRLLLQQQEL---
VKSILQPNYKYPWHIPFTLQPQLLAPLQGEGL-AITYELLEECGLKMELNNPRSTWHL--
EVKMPLSALYASLSLLQQLSEA---

>ref|NP 081236.1| chromosome 15 organism Mus musculus strain C57BL/6 gcode 1 chromosome 15 map 15 gasdermin-D Mus musculus
---DG-IDEEELIE--
AADFQGLYAEVKACSSELESLEMELRQQILVNIGKIL--QDQPSMEALEASLGQGLC-SGGQVE------PLD--
GPAGCILECLVLDSGELVP-------------ELAAPIFYLLGALAVLSETQQQLLAKAL-ETTVLSKQLEL---
VKHVLEQSTPWQEQSSVSLPTVLLGDCWDEK--NPTWVLLEECGLRLQVESPQVHWEP--
TSLIPTSALYASLFLLSSLGQKPC--

>ref|NP 079012.3| chromosome 8 organism Homo sapiens gcode 1 chromosome 8 map 8q24.3 gasdermin-D Homo sapiens
---DGVPAEGAF----
TEDFQGLRAEVETISKELELLDRELCQLLLEGLEGVL--RDQLALRALEEALEQGQS-L-GPVE------PLD--
GPAGAVLECLVLSSGMLVP-------------ELAIPVVYLLGALTMLSETQHKLLAEAL-ESQTLLGPLEL---
VGSLLEQSAPWQERSTMSLPPGLLGNSWGEG--APAWVLLDECGLELGEDTPHVCWEP--
QAQGRMCALYASLALLSGLSQEPH---

>ref|NP 001039625.1| chromosome 14 organism Bos taurus breed Hereford gcode 1 chromosome 14 map 14 gasdermin-D Bos taurus
---DG-STEHWLAT--
TDDYQGLRAEVKAWAMGLEGLSKGLCGQLLGGLGQVL--RDEPALQALEDSLEQGLC-S-GPVE------PRD--
GPVGAILESLVLPSGQLET-------------ELAGPVFYLLQALAVLSEAQHVLLAEVL-EMGALSGTFWL---
VESLVEQSSPWQEHRAVSLPPERLGDSWGSE--APGWALLEACGLEPQVGTPQVCWEP--
GARGCACALYACLALLLRLSQLC---

>ref|XP 015001615.2| location chromosome chromosome 8 completeness complete organism Macaca mulatta isolate AG07107 bio material Coriell:AG07107 gcode 1 chromosome 8 sex female tissue type fibroblasta
---DGVPAEGTF----
TEDFQGLREEVEAISKELELLDRELCQLLLEGLEGVL--RDQLALRALEEALEQGLS-L-GPVE------SLD--
GPAGAVLECLVLESRMLVP-------------ELAVPVVYLLGALTMLSETQHKLLAEAL-ESQTLLGPLEL---
VGSLLEQSAPWQERSTVSLPPGLLGSSWGEG--APAWVLLEECGLELGEDTPHVCWEP--
QAQGRMCALYASLALLSGLSQEPH---

>ref|XP 006913014.1| chromosome Unknown completeness complete organism Pteropus alecto gcode 1 chromosome Unknown sex male tissue type kidney country Australia: Anglican Church Grammar School East Bro
---DG-PTENCLVP--
TEDFQGLQVEVKAWASALENGSATLCGQLLGGLGQVL--RDEPALQALEESLEQGLC-S-GRAE------PLH--
GPAGAVLECLVLHCRTLVE-------------ELARPVFYLLEALAALSETQHMLLAEVL-ETGALPESVEL---
VETLLEQSTPWQEREAISLSPRLLGSNWGSE--APAWVLLEECGLKLQVGAPQVCWEP--
EAQGPTCALYACLALLLKLSQLR---

>ref|XP 036089037.1| chromosome Unknown completeness complete organism Rousettus aegyptiacus isolate mRouAeg1 gcode 1 chromosome Unknown sex male tissue type muscle dev stage adult country USA: Berkes
---DG-PAEHWLVP--
TEDFQGLQMEVRAWARALEDASATLCGQLLGALGQVL--WDEPALQALEESLEQGLC-S-GRAE------PLH--
GPAGAVLECLVLRCGTLVE-------------ELAGPVFYLLGALAALSETQHVLLAKAL-ETGALPEWVKL---
VETLLEQSTPWQEREAISLPSELLESSWGSE--APAWVLLEECGLKLQVGAPQVCWEP--
EAQGPTCALYACLALLLRLSQLR---

>ref|XP 024428237.1| chromosome Unknown completeness complete organism Desmodus rotundus isolate DRU21DN04 gcode 1 chromosome Unknown sex male tissue type muscle/skin sample dev stage adult country Us
---DG-LDEDQVVP--
TEDFQGLQAEVQVWAGPLESLSKTLCEQLLRGLAQVL--RDELALQTLEGSLEQGLC-C-GRAE------PLQ--
GPAGHVLECLVLPCRTLVN-------------ELAGPVTYLLGALAALSEAQRMLLAEVL-EASALPEPFKL---
VEKLLEQSSPWQECRALSLPPGLLGGSWGPE--VPAWILLEACGLELQVDAPQVSWKP--
EAQGCTCALYACLALLSKLSQLC---

>ref|XP 009454389.2| location chromosome chromosome 8 completeness complete organism Pan troglodytes isolate Yerkes chimp pedigree *C0471 (Clint) gcode 1 chromosome 8 sex male tissue type blood dev ss
---DGVPAEGAF----
TEDFQGLRVEVETISKELERLDRELCQLLLKGLEGVL--RDQLALRALEEALEQGQS-L-GPVE------PLD--
GPAGAVLECLVFSSGVLVL-------------ELAIPVVYLLGALTMLSETQHKLLAEAL-ESQTLLGPLEL---
VGSLLEQSAPWQERSTMSLPPGLLGSSWGEG--APAWVLLDECGLELGEDTPHVCWEP--
QAQGRMCALYASLALLSGLSQEPH---

>ref|XP 023104116.1| location chromosome chromosome F2 completeness complete organism Felis catus isolate Cinnamon breed Abyssinian gcode 1 chromosome F2 sex female gasdermin-D Felis catus
---DG-DIEDQMPV--
TEDFQGLKVEVSVHADGLKGLSGELCGQILAGLMKVL--REEPALESLQEELEQGLC-C-GWVA------SPD--
APGGAILECLVQSSGKVEE-------------ELARPILYLVQALTELNETQRVLLAEAL-ETGDLSGQSRL---
VQSVLEQSSPWKEHRAVSLPQELLGSSWDSE--APAWVLLEECGLELRVDVPQVHWQP--

DAQGRTSALYACLVLLPHLSQDQLNETQRVLLAEALETGDLSGQSRLVQSVLEQSSPWKEHRAVSLPQELLGS
SWDSEAPAWVLLEECGLELRVDVPQVHWQPDAQGRTSALYACLVLLPHLSQDRA
>ref|NP 001120925.1| chromosome 7 organism Homo sapiens gcode 1 chromosome 7 map 7p15.3 gasdermin-E isoform a Homo sapiens
--DAAHGISSQDGP--
LSVLKQATLLLERNFHPFAELPEPQQTALSDIFQAVL--FDDELLMVLEPVCDDLVS-GLSPTVAVLG--ELK--
PRQQQDLVAFLQLVGCSLQGGCPGPED-AGSKQLFMTAYFLVSALAEMPDSAAALLGTCC-KLQIIPTLCHL--
-LRALSDDGVSDLED------PTLTPLKDTERF-
GIVQRLFASADISLERLKSSVKAVILKDSKVFPLLLCITLNGLCALGREHS-----------------------------------
----
>ref|NP 061239.1| chromosome 6 organism Mus musculus strain C57BL/6 gcode 1 chromosome 6 map 6 gasdermin-E precursor Mus musculus
--DGGQGISSQDGP--
LRVVKQATLHLERSFHPFAVLPAQQQRALFCVLQKIL--FDEELLRALEQVCDDVAG-GLWSSQAVLAMEELT-
-DSQQQDLTAFLQLVGYRIQGEHPGPQDEVSNQKLFATAYFLVSALAEMPDNATVFLGTCC-KLHVISSLCCL-
--LHALSDDSVCDFHN------PTLAPLRDTERF-
GIVQRLFASADIALERMQFSAKATILKDSCIFPLILHITLSGLSTLSKEHEEELCQSGHATGQD-----------------
----
>ref|NP 001006361.1| chromosome 2 organism Gallus gallus breed Leghorn gcode 1 chromosome 2 map 2 gasdermin-E Gallus gallus
---FLFHPDAVDNGMYSGAENPVPSHAS--
LSVLKKDLSQLKAQFQPFVKLPEDKQRALYKSLCELL--LHEETLTALEDVLDALCT-GDKPDLK-----ELK--
PAQQQDLADFLQLVGCRVQDELLLQNYHLQDEELLSAAHLLISAISELSDYTLVLLRACS-DLQVVPALCCL---
PYIASADGTLALSS------PSVATLTDRRRF-
DVAQRLFASANINLEKTETSVKAVTMREPRFFPLVLYVALCGLHSLGGTVEELF-----------------------------
----
>ref|NP 001180041.1| chromosome 4 organism Bos taurus breed Hereford gcode 1 chromosome 4 map 4 gasdermin-E Bos taurus
--DAGQGLPAQDKP--
LSVLKQATLLLERNFHPFMELPEQQQIALNDVLQAVL--LDEELLVVLEQVCDDIVS-SLSPSLVALG--ELK--
PSQKHNLTTFLRLVGCSVQGERLGSQDVVDNQKLFSTAFFLVSALAEMPDNAAALLGTCC-KLQIIPLLYHL---
LRALSHDGVSDLED------PALAPLKDREKF-
GIVQRLFAATDINLERMQSSVKAASREDPNVLPLILYISLSGLCALGRAQ------------------------------------
----
>ref|XP 002933445.2| location chromosome chromosome 6 completeness complete organism Xenopus tropicalis strain Nigerian gcode 1 chromosome 6 sex female tissue type liver and blood dev stage adult nots
---VLFDWDVVDGSKEAFVRKCILRSAP--
LCLLREGIAAHEKHFSAWKQLPDVQCLELYTLLCQIL--YDGQALSKLHAVVEDLCS-KRKPTQAAFD--ELM--
PSQRIIAENILYLSGYDMPNGKFLHAANR---ELLVALHILTSALNELSDSALAVLGTCC-ELQLLPVFSAL---
MNMSSDEGLCSTTE------PALMDFLDQERF-
YVSQKLFALFNIELEIKEDFIYAATAEDPGFLPLILFIVITGLQLLKRD---

>ref|XP 001096213.2| location chromosome chromosome 3 completeness complete organism Macaca mulatta isolate AG07107 bio material Coriell:AG07107 gcode 1 chromosome 3 sex female tissue type fibroblasta
---DAAHGISSQDGP--
LSVLKQVTPLLERNFHPFVELPEPQQTALSDIFQAVL--FDDELLMVLEPVCDDLVS-GLSPPMAVLG--ELK--
LQQQQDLEAFLQLVGCSLHGGCPGPED-TGSKQLFMTAYFLVSALSEMPDNAAALLGTCC-KLQIIPTLCHL---
LRVLSDDGVSDLED------PTLAPLKDTERF-GIVQRLFASADISLERLKSSVKAVILKDSKVFP-
LLCIALNGLCALGREHS--

>ref|XP 006912063.1| chromosome Unknown completeness complete organism Pteropus alecto gcode 1 chromosome Unknown sex male tissue type kidney country Australia: Anglican Church Grammar School East Bro
---DAWQGPPASDKP--
LSVLKEVTLPLEKSFRPFAELPEQQQTALSGILQAVL--FDDELLLALEQVCDDVFS-DLSPPRVIPG--ELK--
PSQQQDLMAFLHLVGYSMQSGCPGPEDAVSNQKLFSTAYFLVSALAEMPDNAAALLGTCC-KLQIIPTLCHL-
--LHVLSADGESDLED------AALAPLRDTERF-
EIVQRLLAAADINLERQQSSVKAVTCQVPNVSPLVLYISLNGLWALGRMHQ------------------------------------
----
>ref|XP 031796450.1| location chromosome chromosome 5 completeness complete organism Sarcophilus harrisii gcode 1 chromosome 5 cell line 91H gasdermin-E Sarcophilus harrisii
---DSVDGKSEAVKLTPVEGP--
LSALKHGMLVLERNFRPFVELSGHHRAALCEILWEVL--LDDELVVALDTVLSDVLAGGRPRLWALP---GLS--
PGQQQQLAAFLGLAGLHVQEDSRVRRDARAPRELLSTAHFLVSALAEMPDDTVALMGVCC-
QLHLIPTLCHL---PQVTSADGVSELGD------PSLAPLSDAGSF-
EVVQKLFALSHISLERREAHVEAVIMKRPQYLPLVLYITLHGLCALEGKAE---

>ref|XP 027689547.1| location chromosome chromosome 2 completeness complete organism Chelonia mydas isolate rCheMyd1 gcode 1 chromosome 2 sex male tissue type blood dev stage adult country Israel: Mes
---FLYQPDAVDSDRRSGDRNIVPSDAS--
LSVLKQDLLLLKMQFQPFVKLPEDKQGALNKIFYELL--HHEEIVIALEEVLDSIFT-GEEPDFTDLK--ELK--
PPQQQNITDFLQLVGYSLQNELLQKDQLHYT-GLFSAAHILISAVAELSDYTLALLRACC-DIQIVPILCYL---
PNVASADGTLTLRD------PALAPLTDTGRF-
HIVQRLFALSNINLEMMESSMKAVTMKEPSFFPFILYIALYGFYALGGTI--

>ref|XP 040208817.1| location chromosome chromosome 5 completeness complete organism Rana temporaria gcode 1 chromosome 5 gasdermin-E Rana temporaria
---FDWDMVDLSRNISVDGKAVPSNYS--
LSAIKSDILEITKPFSVLQDLPEAHRGEIYNGLFEIL--HDGQTVTQLQAVVEEICL-EKRPGLTFLS--ELK--
SPERAWVQKILHLIGYDMLNQKLIEPFKK---DLLSAVHLLTSALDEMPDTALAILGACC-KLHLLPALWAL---
TNITSDEGLCSRTE------PALTDLIHQGRF-QIAQSLFSFFNMRLELNEKNIAVTS-
EDPSLLPHILYIAISGFHALKNNLKI---

>ref|XP 023962559.1| location chromosome chromosome 2 completeness complete organism Chrysemys picta bellii isolate RCT428 sub species bellii gcode 1 chromosome 2 sex female country USA: Grant Co. ...
--FLYQPDAVDSDRHSGDRNIIPSGAS--
LSVLKQDLLLLKMQFQPFVKLPEDKQGALNKIFYEFL--HHEEMVTALEEVLDSICT-GEEPDFTDLK--ELK--
PPQQQNITDFLQLVGYSLQNELLLQNDQLHYKGLFSAAHILISAVAELSDYTLALLRACC-DIQIIPALCCL---
PNVASADGTLTLRD------PALAPLTDTGRF-
DIVQRLFALSNINLEMMESSVKAVTMKEPSFFPFILYIALYGFYALGGTI---

>ref|XP 029444983.1| location chromosome chromosome 2 completeness complete organism Rhinatrema bivittatum gcode 1 chromosome 2 gasdermin-E isoform X1 Rhinatrema bivittatum
---YSWDVVDSGDRSMMNRNVVPSDAP-
-LSLLKQDVLQQTKIFQSFLELPEEKRSVLNKLLCEIL--SHGETLTLLEDVLDDICT-EHKPNLTALN--ELK--
VEQQQNIKDLWPLLGLGLENELLLQPEKLQEKELLLATHVLVSALEEMGDSPLAILRACC-ELHLMPVLCHL---
LNSLSDDGTSMRDS--------
LLSIFNDKGRFQIAQKLFSLSNISLELTEDNIRAVTKSEPGFLPLILSIILSGFHVLAGTF-----------------------------
----
>ref|XP 004626528.1| chromosome Unknown completeness complete organism Octodon degus isolate 3935 gcode 1 chromosome Unknown sex female gasdermin-E Octodon degus
--DAKHGMLSKDGL--
LSVLKQATLPLERNFHPFVELPAQQQQALHNLLQAVL--LDEELLVVLEQVCDDMAG-
SVWSPQTALAMRALK--
APQQQDLTAFLQLVGCSIQGECPSPEDEVSNQKLFATAYFLVSALAEMPDNAAALLGICC-KLQIIPTLYHM---
LRALSDDGVCDLED------PTLAPLRDTERF-
GIVQRLFVATDINLEQEQASVKAVILKDPHIFPLILYITLNGLCALGREH--

>ref|XP 021046698.1| location chromosome chromosome 2 completeness complete organism Mus pahari gcode 1 chromosome 2 sex female tissue type tail gasdermin-E Mus pahari
--DGGQGISSQDGP--
LRVVKQATLHLERSFHPFAVLPAQQQRALFCVLQKIL--FDEELLRALEQVCDDVAG-GLWSSQAISAMEELT--
DSQQQDLTAFLQLVGYRIQGEHPGPQDAVSNQKLFATAYFLVSALAEMPDNATVFLGTCC-KLHVISSLCCL--
-LHALSDDSVCDFQD------PALAPLRDTERF-
GIVQRLFASADIALERMQFSAKATILKDSCIFPLILHITLSGLSTLSKEHEEQLCQSEGHATGQD------------------
----
>ref|XP 009324004.1| chromosome Unknown completeness complete organism Pygoscelis adeliae isolate BGI AS28 gcode 1 sex male country Antarctica chromosome Unknown PREDICTED: non-syndromic hearing impae
--FLYQPDAVDNEMYSGAKNRIPSDAS--
LSVLKQDLSWLKTQFQPFVKLPEDKQRALYKTLCELL--LHEEMVAALEDLLDDICT-GDKPDLK-----ELK--
PAQQQDLLGFLQLLGCSLQSELLLQKYQPQDEELLSAAHLLISAISELPDYTLVLLRACC-DLQVVPALCCL---
PNIASADGTLALSS------PLVATLTDRGRF-
DVVQRLFASSNITLEMTVSSVKAVTMKEPRFFPLVLYVALYGFYALGGNV--------------------------------------
----

>ref|XP 024418892.1| chromosome Unknown completeness complete organism Desmodus rotundus isolate DRU21DN04 gcode 1 chromosome Unknown sex male tissue type muscle/skin sample dev stage adult country Us
---DAGQGPSAPDEP--
LSVLKQATLPLERAFHPFGQLPEQEQTALSGVLQVVL--FDEELLVVLEQVCDDMVG-GLPPSLAVLG--ELK--
PPQKQNLMAFLQLVGCSMQGECLGPQGVVSNQKLFSTAYFLVSALAEMPDNAAALLGTCC-
KLQIIPALCHL---LRALSAGGEPDLED------TALAPLRDTERF-
GIVQCLLATASLDLERRQSSVKAVTCQVPNVSPLVLYVSLSGLHALGRAHQ---

>ref|XP 016015046.2| chromosome Unknown completeness complete organism Rousettus aegyptiacus isolate mRouAeg1 gcode 1 chromosome Unknown sex male tissue type muscle dev stage adult country USA: Berkes
---DAWQRPSASDKP--
LSVLKQVSLPLERSFRPFAELPQQQQTALSGILQAVL--FDEELLLALEQVCDDVFS-GLSPPRAMPG--ELK--
PSQQQDLAAFLRLVGYSVQSGCPGPEDAVSNQKLFSTAYFLVSALAEMPDNAAALLGTCC-KLQMIPTLCHL-
--LRALSADGESDLED------AALAPLRDTERF-
EIVQRLLAAADIRLERRQSSVKAVTYPVPDVSQLVLYVSLNGLWALARMHQ---------------------------------------
----
>ref|XP 032903802.1| location chromosome chromosome 2 completeness complete organism Amblyraja radiata isolate CabotCenter1 gcode 1 chromosome 2 sex male tissue type testis liver country USA: Gulf ofa
---PLSCVDGAHKCQLSALEMDVPSETS--
LIILKPAIEEANQQFQPFHELAEEKCLQLFRLYCEFL--YHEEVISFLENALDELSS-TEQPNLLGLQ--ELD--
STQAQRVKELLQILGYSCPNEQGSLKKDITTPEILTAAFYLVSALAGMSEESLAVLGICC-ETQILPTLHYL---
INNVPDDGIVPVDN------PNMLPLQEEDNF-
YIAHRLFALSNICLEVRETAIKVITDDQPGMAPVLLCIVLQGFAILSGIKRNTSDPKTQT-----------------------------
----
>ref|XP 001159685.3| location chromosome chromosome 7 completeness complete organism Pan troglodytes isolate Yerkes chimp pedigree *C0471 (Clint) gcode 1 chromosome 7 sex male tissue type blood dev ss
---DAAHGISSQDGP--
LSVLKQATLLLERNFHPFAELPEPQQTALSDIFQAVL--FDDELLMVLEPVCDDLVS-GLSPTVAVLG--ELK--
PRQQQDLVAFLQLVGCSLQGGCPGPED-AGSKQLFMTAYFLVSALAEMPDNAAALLGTCC-KLQIIPTLCHL--
-LRALSDDGVSDLED------PILTPLKDAERF-
GIVQRLFASADISLERLKSSVKAVILKDSKVFPLLLCITLNGLCALGREHS--

PYD Alignment
>sp|Q8WXC3|PYDC1 HUMAN Pyrin domain-containing protein 1 OS=Homo sapiens OX=9606 GN=PYDC1 PE=1 SV=1
---------------------MGTKREAIL-KVLENLTPEELKKFKMKLGTVPLREGFE----RIPRGALGQLDIV-DLTDKLVAS-
-YYEDYAAELVVAVLRDMR-----------MLEEAAR-LQRAA-----------
>NP 660183.1:1-88 apoptosis-associated speck-like protein containing a CARD isoform b Homo sapiens

--------------------MGRARDAIL-DALENLTAEELKKFKLKLLSVPLREGYG----RIPRGALLSMDAL-DLTDKLVSF-
-YLETYGAE-LTANVLRDM-----------GLQEMAG-QLQAA-----------
>NP 001104231.1 uncharacterized protein LOC796649 Danio rerio
--------------------MASTKTEIL-DVLDELRERE---FKD---FKWRLSNKETLENSIPRGKLENADRQ-
DVVDYMEQH--FGTSEAGK-VAVRL--LH-----------SMNQNNL-AEQLKKTLAVVSKV--
>q919n6 danio asc
-------------------MAESFKEHLQ-EAFEDLGADN---LRK---FKSKLGDRRQEP-
RVTKSAIEKLKDEIDLADLMVGV--FTSKDAVS-VTVEI--LR-----------AIKCNAV-ADDLLRNTGQS-----
>q56p42 pydc2 human
-----------------MASSAELDFNLQ-ALLEQLSQDELSKFKS---LIRTISLGK----ELQTVPQTEVDKA-N-GKQLVEI--
FTSHSCS--YWAGMAAIQ-----------VFEKMNQ-THLSGRADEHCVM---
>q91vj1 pycard mouse
-------------------MESEYREMLLLTGLDHITEEE---LKR---FKYFALTEF----QIARSTLDVADRT-ELADHLIQS--
AGAASAVTKAINIFQKLN-----------YMHIANA-LEEKKKE---------
>o35368 ifi203 mouse
--------------------MAEYKNIVLLKGLENMEDYQ---FRT---VKSLLRKEL----KLTKKMQEDYDRI-
QLADWMEDK--FPK---DA-GLDKL--IK-----------VCEHIKD-LKDLAKKLKTEKAK--
>q504j1 caspb danio
---------------------------LS-DVLEDLVESELKQFTR---QLWIGVKPGVE--PIPRGKLENKDRQ-DVVDSMVQQ--
YSED-AGTITVQTL--RK-----------IKQNERA-----------------
>q919l7 caspa danio
--------------------------HLQ-DALSNIGADN---LRR---FQSRLGDRKQEP-RVRKSTIEKLKDEIDLVDLLVNT--
FTSD-AVS-VTVDI--LR-----------GIKCNAV-AE--------------
>tr|B2CWB9|B2CWB9 9POXV M013 OS=Myxoma virus OX=10273 GN=m013L PE=4 SV=1
---------------------MEHRGVII-TVLENLSDYQ---FKM---FIYLAMEDL----YIERAEKEKIDRI-DLAHKISEQ--
YLGTDYIE-FMKRV--TD-----------FIPNKVY-VDSLLARAEADA----
>NP 051902.1 Hypothetical protein SFV s013L Rabbit fibroma virus
---------------------MEHRGVII-TVLENLTDYQ---FKM---FLYLVTEDL----RINPVEKEKIDRI-DLAYKISEL--
YPGHSYIE-FMKQV--TG-----------YIPNKVY-VDSLLKNAEENT----
>YP 227401.1 hypothetical protein DpV83gp024 Deerpox virus W-848-83
---------------------MELRSAII-AVLENINRYQ---FKM---VIFIVQDEL----YIEEEEKLTMDRI-DLAEKLIKK--YKD--
-IR-SLYFL--LN-----------IFKEMPD-TEFVKKQLSDYIKRF-
>NP 073403.1 18L protein Yaba-like disease virus
-----------------MLYTMRIKSAII-FSLEDVTHYQ---FKI---LIFLTKDEL----NISDEEKQILDRV-DFAEKLFQT--
YPG---IK-SLYFL--EK-----------AISMVPN-AKYARSNINRLISDL-
>ABQ43490.1 pyrin domain Tanapox virus
-----------------MLYNMRIKSAII-FSLEDVTHYQ---FKI---LIFLTKDEL----NINDEEKQILDRV-DFAEKLFQA--
YPG---IK-SLYFL--EK-----------AISMVPN-AKYARSNINRLISDL-
>NP 570174.1 Hypothetical protein SWPVgp014 Swinepox virus
---------------------MELRYTII-SVLERLTPYQ---FKT---LLFLIQDDI----NISNDDINVLDRV-DLAIKIMNK--YNN-
--YR-AIYFL--YK-----------VILRIHN-TEYISGTLQRSIQNI-
>Vaccinia
-----MVKNNKISNSCRMIMSTNPNNILM-RHLKNLTDDE---FKC---IIHRSSDFL----YLSDSDYTSITKE-
TLVSEIVEE--YPDD-CNK-ILAII--FL-----------VLDKDID-VDIET-----------

>Buffalopox
-----MVKNNKISNSCLMIMSTNPNNILM-RHLKNLTDDE---FKC---IIHRSSDFL----YLSDSDYTSITKE-TLVSEIVEE--YPDD-CNK-ILAII--FL-----------VLDKDID-VDIET-----------
>Cowpox
MVKSNKIQKNKISNSCRMIMSTNPNNILM-RHLKNLTDDE---FKC---IIHRSSDFL----YLSDRDYTSITKE-TLVSEIVEE--YPDD-CNK-ILAII--FL-----------VLDKDID-VDIET-----------
>Rabbitpox
-----MVKNNKISNSCLMIMSTDPNNILM-RHLKNLTDDE---FKC---IIHRSSDFL----YLSDRDYTSITKE-TLVSEIVEE--YPDD-CNK-ILAII--FL-----------VLDKDID-VDIET-----------
>Horsepox
------MVKNKISNSCLMIMSTDPNNILM-RHLKNLTDDE---FKC---IIHRSSDFL----YLSNRDYTSITKE-TLVSEIVEE--YPDD-CNK-ILAII--FL-----------VLDKDID-VDIKT-----------
>Orthopox abatino
MVKNNKIQKIKTSNSYRDVIMTNPNNILM-RHLKNLTDDE---FKC---IVNRSSDFI----YLSDRDYTSITKE-TLVSEIVEE--YPDD-CNK-ILAII--FL-----------VLGKDVD-IETET-----------
>Akhmeta virus
MVKSNKIQKNKISNSCRYMIMNTNTNILM-RHLKNLTDDE---FKC---IIHRSSDFL----YLSDRDYTSITKE-TLVSEIVEE--YQDD-CNK-ILAII--FL-----------VLDKDID-VDIET-----------
>Variola Virus
-----------------MIMSTDPNNILM-RYLKNLTDDK---FKC---IIHQSSDFL----YLSDRDYTSITKE-TLVSEIVEE--YPDD-CNK-ILAII--FL-----------VLDKDID-VDIET-----------
>Monkeypox
-----------------MIMRTDTNNILM-RHLKNLTDDE---FKC---IIHRSSDFL----YLSDRDYTSITKE-TLVSEIVEE--YPDD-CNK-ILAII--FL-----------VLDKDID-VDIET-----------
>Alaskapox
-----------------------MNTILM-RHFKNLTDDE---FKC---IIHRSSDFI----YLSDRDYTSITKE-TLVSEIVEE--YPDD-CDK-ILAII--FL-----------VLGKDIE-VDVKT-----------
>Volepox
---------------------MANKNRLV-SYLKNLTDDE---FKC---IIYRSSDFI----YLSDSDHDTITKE-KLVKEIIEE--YPDD-CNK-ILAII--FL-----------VLDKDID-IDIET-----------
>Skunkpox
----------------------MNKNRLV-KYLKNLTDDE---FKC---IIYRSSDFI----YLSDSDHDNITRE-TLVKEIMEE--YPDD-CNK-ILAII--FL-----------VLDKDID-LDIET-----------
>Raccoonpox
---------------------MDVKNRIV-KYLKNLTDDE---FRY---IIYRSSDFI----YLSDGDHNTITKE-TLVNEIIEE--YPDD-CNK-IFAII--FL-----------VLDIDID-LDTET-----------
>NY 014 poxvirus
------------------------MDKIM-CYLNNITDEE---FET---VIDRSSNFL----YLSDDDHTNITRE-SLALEIVEE--YPND-CER-ILAII--LL-----------SLDKYIS-FESTI-----------
>Murmansk poxvirus
------------------------MDKIT-HYLNNITDGE---FET---VIDRSSNFL----YLSDDDHTNITRE-SLALEIVEE--YPND-CER-ILAIL--LL-----------SLDKYIS-FESTI-----------
>Yoka poxvirus

-----------------------MNKIL-NYFNSITDDE---FKT---IISISSDFL----YLSDNDNINITKE-SLALEILEQYPYPND-YEK-ILSML--LL-----------ALGKYVF-FETII-----------
>QDJ94995.1 IFN-inducible protein Hypsugopox virus
------------------MDSYTKGSIL-YALKNLTFQQ---LKE---FMFISSEQL----YLSNKDRLNFDVV-DLADRIIIV--CHDN-ALK-AFKLI--KE-----------ILTMMES-VDFVINYMNKCIENTC
>QHG62576.1 hypothetical protein Cetacean poxvirus 1
-----------MIDSKSLNVEIEVKIALM-RALDELSSYQ---LKV---LAFLTSNRV----CVIHRSMSNVDRV-DIAKRINTT--LP---CVC-SLTYLRDLLKNVKHTESVRDILCEDINYIESIMRNNNEIKRFTC
>YP 009407969.1 IFN-inducible protein Eptesipox virus
------------------MDAFTKGSTL-YALKNLTFNQ---LKE---FMFISSDGL----YLTHKNRLEFDVV-DLADRIIIV--NNNN-PLK-VFKHI--KE-----------TLCLMDD-VEFVLKYIDKCIENCA

GSDM Tree

(((((((((((('AY484669.1:c159516-158782 Rabbitpox virus complete genome':0.00000632,'KY349117.1:c166272-165538 Horsepox virus strain MNR complete genome':0.00396044)0.5300:0.00000632,'M35027.1:c153690-152956 Vaccinia virus Copenhagen complete genome':0.00396094)0.6900:0.00396040,('MG599038.1:c157906-157148 Buffalopox virus isolate Karachi 2005 complete genome':0.00000632,'AY243312.1:c154675-153917 Vaccinia virus WR complete genome':0.00384602)0.7600:0.00398762)0.8500:0.01196656,'KC813500.1:c153312-152578 Cowpox virus strain MonKre08/4 complete genome':0.00000632)0.2000:0.00001264,'MN912466.1:c156293-155559 Ectromelia virus WH complete genome':0.01989595)0.6200:0.00395345,'NC 055231.1:c163765-163031 Orthopoxvirus Abatino complete genome':0.00793905)0.5300:0.00641072,('MH607143.1:c167386-166652 Akhmeta virus isolate Vani 2010 complete genome':0.02387728,((('AY009089.1:c154402-154034 Camelpox virus CMS complete genome':0.00000632,'MK910851.1:c154204-153836 Camelpox virus strain Negev2016 complete genome':0.00808054)0.9200:0.02440740,'DQ437594.1:c153307-152573 Taterapox virus strain Dahomey 1968 complete genome':0.01191498)0.3300:0.00000632,('KY358055.1:c145385-144651 Variola virus strain VD21 17th century complete genome':0.00000632,('DQ441432.1:c146026-145292 Variola virus strain Korea 1947 (Lee Masterseed) complete genome':0.00395560,'DQ441437.1:c146904-146170 Variola virus strain Sierra Leone 1969 (V68-258) complete genome':0.00000632)0.7700:0.00395750)0.8700:0.00793967)0.6500:0.01266543)0.3200:0.00554384)0.5300:0.03253804,'MN240300.1:c166555-165827 Alaskapox virus complete genome':0.11139866)0.9700:0.13966158,('KU749309.1:c145369-144644 Raccoonpox virus strain 85A complete genome':0.10647955,('KU749310.1:c167631-166873 Skunkpox virus strain WA complete genome':0.07403330,'KU749311.1:c169057-168281 Volepox virus strain CA complete genome':0.09493255)0.5900:0.02370722)0.4600:0.07236375)1.0000:0.50096918,('HQ849551.1:c134784-134035 Yoka poxvirus strain DakArB 4268 complete genome':0.41854758,('MF001305.1:c159640-158771 NY 014 poxvirus strain 2013 complete genome':0.10644996,'MF001304.1:c160603-159728 Murmansk poxvirus strain LEIV-11411

complete
genome':0.06453801)0.8000:0.16035801)0.4100:0.07538004)1.0000:1.10608085,YP_00940809
7.1_Immunoprevalent_protein_Eptesipox_virus:0.88065310)0.5900:0.32061200,((NP_051846.
1_m132L_Myxoma_virus:0.09720761,NP_052021.1_gp132L_Rabbit_fibroma_virus:0.24063256
)1.0000:1.72365406,(('MF001304.1:c165763-165224 Murmansk poxvirus strain LEIV-11411
complete genome':0.04040716,'MF001305.1:c164808-164269 NY 014 poxvirus strain 2013
complete genome':0.11589372)0.8900:0.43947697,('KU749311.1:173861-174445 Volepox virus
strain CA complete genome':0.03751514,('KU749310.1:172437-172985 Skunkpox virus strain
WA complete genome':0.06828167,'KU749309.1:150174-150722 Raccoonpox virus strain 85A
complete
genome':0.09772203)0.6700:0.06086908)0.9400:0.42301996)1.0000:1.32178979)0.4300:0.417
93913)0.6600:0.74188710,((((((((ref|NP_113603.1|_chromosome_8_organism_Homo_sapiens
_gcode_1_chromosome_8_map_8q24.21_gasdermin-C_Homo_sapiens:0.00648950,'ref|XP
001153860.1| location chromosome chromosome 8 completeness complete organism Pan
troglodytes isolate Yerkes chimp pedigree *C0471 (Clint) gcode 1 chromosome 8 sex male
tissue type blood dev ss':0.01016408)0.7300:0.02356312,'ref|XP 028708741.1| location
chromosome chromosome 8 completeness complete organism Macaca mulatta isolate
AG07107 bio material Coriell:AG07107 gcode 1 chromosome 8 sex female tissue type
fibroblasta':0.05208806)1.0000:0.26393153,ref|XP_019678683.1|_location_chromosome_chro
mosome_F2_completeness_complete_organism_Felis_catus_isolate_Cinnamon_breed_Abyssi
nian_gcode_1_chromosome_F2_sex_female_gasdermin-
C_Felis_catus:0.41823708)0.1300:0.05011136,('ref|XP 036088919.1| chromosome Unknown
completeness complete organism Rousettus aegyptiacus isolate mRouAeg1 gcode 1
chromosome Unknown sex male tissue type muscle dev stage adult country USA:
Berkes':0.32082568,ref|XP_024427836.1|_chromosome_Unknown_completeness_complete_o
rganism_Desmodus_rotundus_isolate_DRU21DN04_gcode_1_chromosome_Unknown_sex_ma
le_tissue_type_muscle/skin_sample_dev_stage_adult_country_Us:0.13881323)0.6000:0.09111
937)0.1400:0.07902331,ref|NP_001039469.1|_chromosome_14_organism_Bos_taurus_breed
_L1_Hereford_gcode_1_chromosome_14_map_14_gasdermin-
C_Bos_taurus:0.55125872)0.3600:0.07430343,ref|XP_021073018.1|_location_chromosome_c
hromosome_17_completeness_complete_organism_Mus_pahari_gcode_1_chromosome_17_s
ex_female_tissue_type_tail_gasdermin-
C_Mus_pahari:0.36366499)0.9800:0.59858814,ref|XP_031803207.1|_location_chromosome_c
hromosome_1_completeness_complete_organism_Sarcophilus_harrisii_gcode_1_chromosome
_1_cell_line_91H_gasdermin-C_Sarcophilus_harrisii:0.73800521)0.4200:0.14672251,((('ref|XP
015154998.2| location chromosome chromosome 27 completeness complete organism Gallus
gallus isolate bGalGal1 gcode 1 chromosome 27 sex female tissue type blood country USA:
Fayetteville lat
ls':0.89302167,ref|XP_029428328.1|_location_chromosome_chromosome_12_completeness_
complete_organism_Rhinatrema_bivittatum_gcode_1_chromosome_12_gasdermin-
A_Rhinatrema_bivittatum:1.13451927)0.3600:0.10801623,XP_032891479.1_gasdermin-
A_Amblyraja_radiata:1.35621246)0.1500:0.21106408,(ref|NP_081236.1|_chromosome_15_or
ganism_Mus_musculus_strain_C57BL/6_gcode_1_chromosome_15_map_15_gasdermin-
D_Mus_musculus:0.37973081,(((ref|NP_079012.3|_chromosome_8_organism_Homo_sapiens

_gcode_1_chromosome_8_map_8q24.3_gasdermin-D_Homo_sapiens:0.00490946,'ref|XP 009454389.2| location chromosome chromosome 8 completeness complete organism Pan troglodytes isolate Yerkes chimp pedigree *C0471 (Clint) gcode 1 chromosome 8 sex male tissue type blood dev ss':0.03154543)0.9800:0.02755997,'ref|XP 015001615.2| location chromosome chromosome 8 completeness complete organism Macaca mulatta isolate AG07107 bio material Coriell:AG07107 gcode 1 chromosome 8 sex female tissue type fibroblasta':0.02541537)1.0000:0.24655309,(ref|XP_023104116.1|_location_chromosome_chromosome_F2_completeness_complete_organism_Felis_catus_isolate_Cinnamon_breed_Abyssinian_gcode_1_chromosome_F2_sex_female_gasdermin-D_Felis_catus:0.38688835,(ref|NP_001039625.1|_chromosome_14_organism_Bos_taurus_breed_Hereford_gcode_1_chromosome_14_map_14_gasdermin-D_Bos_taurus:0.18906395,(ref|XP_024428237.1|_chromosome_Unknown_completeness_complete_organism_Desmodus_rotundus_isolate_DRU21DN04_gcode_1_chromosome_Unknown_sex_male_tissue_type_muscle/skin_sample_dev_stage_adult_country_Us:0.22927157,('ref|XP 006913014.1| chromosome Unknown completeness complete organism Pteropus alecto gcode 1 chromosome Unknown sex male tissue type kidney country Australia: Anglican Church Grammar School East Bro':0.05855083,'ref|XP 036089037.1| chromosome Unknown completeness complete organism Rousettus aegyptiacus isolate mRouAeg1 gcode 1 chromosome Unknown sex male tissue type muscle dev stage adult country USA: Berkes':0.07916446)0.9600:0.08309248)0.6600:0.09456772)0.6500:0.07865023)0.8200:0.11484867)0.5700:0.05693998)1.0000:0.51206201)0.1000:0.19500033)0.0200:0.03306529)0.0000:0.15081324,((sp|Q5Y4Y6|GSDA3_MOUSE_Gasdermin-A3_OS_Mus_musculus_OX_10090_GN_Gsdma3_PE_1_SV_1:0.02992407,sp|Q32M21|GSDA2_MOUSE_Gasdermin-A2_OS_Mus_musculus_OX_10090_GN_Gsdma2_PE_2_SV_1:0.08079961)0.5000:0.01350323,(sp|Q9EST1|GSDMA_MOUSE_Gasdermin-A_OS_Mus_musculus_OX_10090_GN_Gsdma_PE_2_SV_1:0.01547699,((('ref|XP 016020091.2| chromosome Unknown completeness complete organism Rousettus aegyptiacus isolate mRouAeg1 gcode 1 chromosome Unknown sex male tissue type muscle dev stage adult country USA: Berkes':0.00923641,'ref|XP 006924750.1| chromosome Unknown completeness complete organism Pteropus alecto gcode 1 chromosome Unknown sex male tissue type kidney country Australia: Anglican Church Grammar School East Bro':0.01784865)0.9500:0.03854298,ref|XP_024429158.1|_chromosome_Unknown_completeness_complete_organism_Desmodus_rotundus_isolate_DRU21DN04_gcode_1_chromosome_Unknown_sex_male_tissue_type_muscle/skin_sample_dev_stage_adult_country_Us:0.07730313)0.4600:0.00685917,(ref|XP_035935879.1|_chromosome_Unknown_completeness_complete_organism_Halichoerus_grypus_isolate_241Hg_ecotype_Northwest_Atlantic_gcode_1_chromosome_Unknown_sex_male_tissue_type_blood_dev_stage_molts:0.09006814,((ref|XP_024836266.1|_location_chromosome_chromosome_19_completeness_complete_organism_Bos_taurus_isolate_L1_Dominette_01449_registration_number_42190680_breed_Hereford_gcode_1_chromosome_19_sex_fem...:0.07277097,ref|XP_004633937.1|_chromosome_Unknown_completeness_complete_organism_Octodon_degus_isolate_3935_gcode_1_chromosome_Unknown_sex_female_gasdermin-A_Octodon_degus:0.08986357)0.0600:0.00538989,('ref|XP 014975037.1| location chromosome chromosome 16 completeness complete organism Macaca

mulatta isolate AG07107 bio material Coriell:AG07107 gcode 1 chromosome 16 sex female tissue type
fibroblaa':0.03818418,(ref|NP_835465.2|_chromosome_17_organism_Homo_sapiens_gcode_1_chromosome_17_map_17q21.1_gasdermin-A_Homo_sapiens:0.00536674,'ref|XP 001171222.1| location chromosome chromosome 17 completeness complete organism Pan troglodytes isolate Yerkes chimp pedigree *C0471 (Clint) gcode 1 chromosome 17 sex male tissue type blood
devs':0.00000632)0.5100:0.01070020)0.9800:0.04354841)0.1500:0.00623461)0.1800:0.00553476)0.8000:0.06299807)0.7900:0.04239040)0.9900:0.44258489,((((((('ref|XP 024897515.1| chromosome Unknown completeness complete organism Pteropus alecto gcode 1 chromosome Unknown sex male tissue type kidney country Australia: Anglican Church Grammar School East Bro':0.06586777,'ref|XP 036078993.1| chromosome Unknown completeness complete organism Rousettus aegyptiacus isolate mRouAeg1 gcode 1 chromosome Unknown sex male tissue type muscle dev stage adult country USA:
Berkes':0.09991046)1.0000:0.11539316,ref|XP_024429114.1|_chromosome_Unknown_completeness_complete_organism_Desmodus_rotundus_isolate_DRU21DN04_gcode_1_chromosome_Unknown_sex_male_tissue_type_muscle/skin_sample_dev_stage_adult_country_Us:0.21763352)0.4000:0.02806263,ref|XP_035935913.1|_chromosome_Unknown_completeness_complete_organism_Halichoerus_grypus_isolate_241Hg_ecotype_Northwest_Atlantic_gcode_1_chromosome_Unknown_sex_male_tissue_type_blood_dev_stage_molts:0.34581838)0.2500:0.03004080,ref|NP_001231146.1|_chromosome_19_organism_Bos_taurus_gcode_1_chromosome_19_map_19_gasdermin-B_Bos_taurus:0.26530564)0.4800:0.09159899,('ref|XP 014975035.1| location chromosome chromosome 16 completeness complete organism Macaca mulatta isolate AG07107 bio material Coriell:AG07107 gcode 1 chromosome 16 sex female tissue type fibroblaa':0.11006455,(ref|NP_001159430.1|_chromosome_17_organism_Homo_sapiens_gcode_1_chromosome_17_map_17q21.1_gasdermin-
B_isoform_3_Homo_sapiens:0.01869987,'ref|XP 009430521.1| location chromosome chromosome 17 completeness complete organism Pan troglodytes isolate Yerkes chimp pedigree *C0471 (Clint) gcode 1 chromosome 17 sex male tissue type blood
devs':0.01689996)0.9400:0.05518121)1.0000:0.07184298)1.0000:0.50473782,ref|XP_023358160.1|_location_chromosome_chromosome_4_completeness_complete_organism_Sarcophilus_harrisii_gcode_1_chromosome_4_cell_line_91H_gasdermin-
B_Sarcophilus_harrisii:0.56088280)0.9600:0.33170458,('ref|XP 032903802.1| location chromosome chromosome 2 completeness complete organism Amblyraja radiata isolate CabotCenter1 gcode 1 chromosome 2 sex male tissue type testis liver country USA: Gulf
ofa':0.57159834,(((ref|XP_002933445.2|_location_chromosome_chromosome_6_completeness_complete_organism_Xenopus_tropicalis_strain_Nigerian_gcode_1_chromosome_6_sex_female_tissue_type_liver_and_blood_dev_stage_adult_nots:0.58041056,ref|XP_040208817.1|_location_chromosome_chromosome_5_completeness_complete_organism_Rana_temporaria_gcode_1_chromosome_5_gasdermin-
E_Rana_temporaria:0.43530056)0.7400:0.37086991,ref|XP_029444983.1|_location_chromosome_chromosome_2_completeness_complete_organism_Rhinatrema_bivittatum_gcode_1_chromosome_2_gasdermin-
E_isoform_X1_Rhinatrema_bivittatum:0.30113784)0.5100:0.13675007,(((ref|NP_001006361.1

|_chromosome_2_organism_Gallus_gallus_breed_Leghorn_gcode_1_chromosome_2_map_2_
gasdermin-E_Gallus_gallus:0.14607108,'ref|XP 009324004.1| chromosome Unknown
completeness complete organism Pygoscelis adeliae isolate BGI AS28 gcode 1 sex male country
Antarctica chromosome Unknown PREDICTED: non-syndromic hearing
impae':0.06555493)1.0000:0.18747693,('ref|XP 027689547.1| location chromosome
chromosome 2 completeness complete organism Chelonia mydas isolate rCheMyd1 gcode 1
chromosome 2 sex male tissue type blood dev stage adult country Israel:
Mes':0.07982534,'ref|XP 023962559.1| location chromosome chromosome 2 completeness
complete organism Chrysemys picta bellii isolate RCT428 sub species bellii gcode 1
chromosome 2 sex female country USA: Grant Co.
...':0.01125944)0.9900:0.09327687)0.9600:0.15059215,(ref|XP_031796450.1|_location_chrom
osome_chromosome_5_completeness_complete_organism_Sarcophilus_harrisii_gcode_1_chr
omosome_5_cell_line_91H_gasdermin-E_Sarcophilus_harrisii:0.49835971,(((('ref|XP
006912063.1| chromosome Unknown completeness complete organism Pteropus alecto gcode
1 chromosome Unknown sex male tissue type kidney country Australia: Anglican Church
Grammar School East Bro':0.04540917,'ref|XP 016015046.2| chromosome Unknown
completeness complete organism Rousettus aegyptiacus isolate mRouAeg1 gcode 1
chromosome Unknown sex male tissue type muscle dev stage adult country USA:
Berkes':0.06765941)1.0000:0.10802648,ref|XP_024418892.1|_chromosome_Unknown_compl
eteness_complete_organism_Desmodus_rotundus_isolate_DRU21DN04_gcode_1_chromosom
e_Unknown_sex_male_tissue_type_muscle/skin_sample_dev_stage_adult_country_Us:0.1214
4140)0.9300:0.07802127,ref|NP_001180041.1|_chromosome_4_organism_Bos_taurus_breed
_Hereford_gcode_1_chromosome_4_map_4_gasdermin-
E_Bos_taurus:0.16311047)0.7200:0.04030548,(((ref|NP_001120925.1|_chromosome_7_organi
sm_Homo_sapiens_gcode_1_chromosome_7_map_7p15.3_gasdermin-
E_isoform_a_Homo_sapiens:0.00453124,'ref|XP 001159685.3| location chromosome
chromosome 7 completeness complete organism Pan troglodytes isolate Yerkes chimp pedigree
*C0471 (Clint) gcode 1 chromosome 7 sex male tissue type blood dev
ss':0.00911979)0.9900:0.01107332,'ref|XP 001096213.2| location chromosome chromosome 3
completeness complete organism Macaca mulatta isolate AG07107 bio material
Coriell:AG07107 gcode 1 chromosome 3 sex female tissue type
fibroblasta':0.05555503)1.0000:0.14905206,(ref|XP_004626528.1|_chromosome_Unknown_c
ompleteness_complete_organism_Octodon_degus_isolate_3935_gcode_1_chromosome_Unkn
own_sex_female_gasdermin-
E_Octodon_degus:0.14112392,(ref|NP_061239.1|_chromosome_6_organism_Mus_musculus_
strain_C57BL/6_gcode_1_chromosome_6_map_6_gasdermin-
E_precursor_Mus_musculus:0.01643603,ref|XP_021046698.1|_location_chromosome_chrom
osome_2_completeness_complete_organism_Mus_pahari_gcode_1_chromosome_2_sex_fem
ale_tissue_type_tail_gasdermin-
E_Mus_pahari:0.04506946)1.0000:0.24424411)0.8800:0.06822000)0.6100:0.02349932)1.0000:
0.29867710)1.0000:0.32476703)0.9400:0.23585091)0.6400:0.34461861)1.0000:1.77119903)0.
0100:0.08220955);

PYD Tree

(sp_Q8WXC3_PYDC1_HUMAN:0.0000021986,NP_660183.1_1-
88:0.8189957255,((((NP_001104231.1:0.5582152076,(q919n6_danio:0.1596274241,q919l7_cas
pa:0.1464068014)100:1.6604787690)78:0.3649286491,q504j1_caspb:0.8779213839)75:0.3248
494404,q91vj1_pycard:1.3275505794)53:0.3488003785,(q56p42_pydc2:2.4248812488,((((o353
68_ifi203:1.9252008714,(tr_B2CWB9_B2CWB9_9POXV:0.1873719086,NP_051902.1:0.1151924
580)98:0.8588394249)30:0.1685723872,QHG62576.1_hypothetical:1.3381578798)25:0.020581
3423,((YP_227401.1:0.4922697513,((NP_073403.1:0.0000024848,ABQ43490.1_pyrin:0.032263
9322)100:0.5536693021,NP_570174.1:0.6631629338)89:0.1050133382)91:0.2980680604,(QDJ
94995.1_IFN-
inducible:0.0322861746,YP_009407969.1:0.3451439803)100:1.1424169120)56:0.1652826846)
38:0.2909839033,((((((Vaccinia:0.0000024478,(Cowpox:0.0000024085,((Orthopox_abatino:0.10
55672911,Alaskapox:0.0953186299)96:0.0427250561,Akhmeta_virus:0.0648530371)92:0.0274
133695)92:0.0511067176)53:0.0098506539,Buffalopox:0.0000023745)51:0.0083698252,(Rabbi
tpox:0.0000027502,((Horsepox:0.0531890168,Variola_Virus:0.0358970206)28:0.0000020161,
Monkeypox:0.0242749897)26:0.0000027502)32:0.0115162760)97:0.2153206803,(Volepox:0.0
403124363,Skunkpox:0.0547150846)81:0.0431585044)36:0.0492731175,Raccoonpox:0.084855
2751)84:0.2158394416,((NY_014:0.0278797725,Murmansk_poxvirus:0.0311930000)97:0.1567
232511,Yoka_poxvirus:0.3046051547)97:0.3533709024)96:0.7863255573)26:0.1931065783)79
:0.4863747229)100:0.9712551055);

```
#!/bin/bash

#checks to ensure that matching files are present in both folders

for tmfile in TM/*; do
	name=${tmfile##*/}

	if [ -f "FATCAT/$name" ]; then
		echo "$name"
	else
		echo "$name is MISSING in FATCAT folder"
	fi
done


for fatfile in FATCAT/*; do
	name=${fatfile##*/}

	if [ -f "TM/$name" ]; then
		echo "$name"
	else
		echo "$name is MISSING in TM folder"
	fi
done
```

AlphaFold parameters

```
# explicit run of run_alphafold pointing to the appropriate databases on the RAM disk, for file passed in as $1
singularity exec --nv /uufs/chpc.utah.edu/sys/installdir/alphafold/2.1.2/alphafold_2.1.2.sif /app/run_alphafold.sh --data_dir=/scratch/general/lustre/app-repo/alphafold --uniref90_database_path=/scratch/general/lustre/app-repo/alphafold/uniref90/uniref90.fasta --mgnify_database_path=/scratch/general/lustre/app-repo/alphafold/mgnify/mgy_clusters_2018_12.fa --uniclust30_database_path=/dev/shm/af/uniclust30_2018_08 --bfd_database_path=/dev/shm/af/bfd_metaclust_clu_complete_id30_c90_final_seq.sorted_opt --pdb70_database_path=/dev/shm/af/pdb70 --template_mmcif_dir=/scratch/general/nfs1/app-repo/alphafold/pdb_mmcif/mmcif_files --obsolete_pdbs_path=/scratch/general/nfs1/app-repo/alphafold/pdb_mmcif/obsolete.dat --fasta_paths=$1 --max_template_date=2020-05-14 --output_dir=temp_module --use_gpu_relax
```

Example FATCAT call for file passed in as $1

```
FATCATSearch.pl $1 /scratch/general/vast/user_directory/drome_v2/chain_list-drome-fatcat  -i
/scratch/general/vast/user_directory/drome_v2/ -q > $1.drosophila.FATCAT.txt &
```

Example TM-align call for file passed in as $FILE

```
TMalign -dir1 /home/ian/AlphaDat/Proteomes/human_v2/
/home/ian/AlphaDat/Proteomes/human_v2/chain_list $FILE -outfmt 2 -fast > $FILE.human.txt
&
```

```
#!/bin/bash

#this file extracts hit name, score, opt-rmsd, align-len, and P-value from a fatcat file in a format
suitable for further processing
#IB 3.21.2022

for FILE in *FATCAT.txt
do
	outfile="$FILE.scores.txt"
	echo "Model Score RMSD Aligned P-value" >> $outfile
	reading=0 #0 before read, 1 when alignment found
	while read line
	do
		if [[ ${line:0:5} == "Align" ]] #we found a new entry
		then
			reading=1
			string="${line#*with *}"
			desired="${string%.pdb*}"
			echo -n $desired >> $outfile
		else
			if ((reading == 1))
			then
				string="${line#*Score *}"
				desired="${string% align*}"
				echo -n " $desired" >> $outfile

				string="${line#*opt-rmsd *}"
				desired="${string% chain*}"
				echo -n " $desired" >> $outfile
```

```
                                string="${line#*align-len *}"
                                desired="${string% gaps*}"
                                echo -n " $desired" >> $outfile
                                reading=2
                else
                        if ((reading == 2))
                        then
                                string="${line#*P-value *}"
                                desired="${string% Afp*}"
                                echo " $desired" >> $outfile
                                reading=0
                        fi
                        fi
                fi
        done < $FILE
done
```

```
#FATCAT and TMalign data visualization v0.2
#IB 11.28.2022
#Requires properly-formatted TM-align + FATCAT datafiles
#Requires a "working_prot.txt" list of input files
#change working directory as appropriate
#This script has been adapted to produce a figure suitable for import into illustrator as an SVG

#next: work on limiting "too many hit" plots

library(ggplot2)
library(ggimage)
library(ggrepel)
library(ggnewscale)
library(svglite)


protList <-read.table("/home/ian/homology_search/fcresults/working_prot.txt")
protList <- unlist(protList)

cols <- c("Mouse" = "paleturquoise1", "Human" = "mediumspringgreen", "Danio" = "plum1",
"Fly" = "khaki1")

for (protein in protList) {
  setwd("/home/ian/homology_search/fcresults/fat_tm_merge/converted")
  hasHits <- 0
```

```
  mouseData <- read.table(header = TRUE, text=gsub(" ", "\t",
readLines(paste(protein,".pdb.mouse.txt",sep=""))))
  humanData <- read.table(header = TRUE, text=gsub(" ", "\t",
readLines(paste(protein,".pdb.human.txt",sep=""))))
  danioData <- read.table(header = TRUE, text=gsub(" ", "\t",
readLines(paste(protein,".pdb.danio.txt",sep=""))))
  flyData   <- read.table(header = TRUE, text=gsub(" ", "\t",
readLines(paste(protein,".pdb.drosophila.txt",sep=""))))

  #append source genome name - used later when annotating graphs
  mouseData$Proteome <- "Mouse"
  humanData$Proteome <- "Human"
  danioData$Proteome <- "Danio"
  flyData$Proteome <- "Fly"

  testData <- merge(humanData, mouseData, all = TRUE)
  testData <- merge(testData, danioData, all = TRUE)
  testData <- merge(testData, flyData, all = TRUE)

  #Hits defined as TM score > 0.6 AND FATCAT P-value < 0.01
  hits <- testData[testData$TM2 > 0.6, ]
  hits <- hits[-log10(hits$P.value)>2, ]

  #if no hits, instead label the top .01% of results from each tool
  if (nrow(hits)>0){
    finalLabel <- hits
    hasHits <- 1
  } else {
    topP <- quantile(-log10(testData$P.value), probs = .9999)
    topTM <- quantile(testData$TM2, probs = .9999)
    toLabel <- testData[testData$TM2>topTM, ]
    toLabel2 <- testData[-log10(testData$P.value)>topP, ]
    toLabel3 <- rbind(toLabel, toLabel2)
    finalLabel <- unique(toLabel3)
  }

  if(hasHits) {
    graphTitle<-protein
  } else {
    graphTitle<-paste(protein, ": No hits, top-scoring values are indicated")
  }


  p <- ggplot(testData, aes(x=TM2, y=(-log10(P.value)))) +
```

```
   ggtitle(graphTitle) +
   xlab("TM Score") +
   ylab(bquote(Log[10]~P-value~FATCAT)) +
   geom_point(data = humanData, aes(x=TM2, y=(-log10(P.value)), size=1,
color=factor(cut(RMSD.1, c(0,2,3,5,Inf))))) +
   geom_point(data = mouseData, aes(x=TM2, y=(-log10(P.value)), size=1,
color=factor(cut(RMSD.1, c(0,2,3,5,Inf))))) +
   geom_point(data = danioData, aes(x=TM2, y=(-log10(P.value)), size=1,
color=factor(cut(RMSD.1, c(0,2,3,5,Inf))))) +
   geom_point(data = flyData,   aes(x=TM2, y=(-log10(P.value)), size=1,
color=factor(cut(RMSD.1, c(0,2,3,5,Inf))))) +
   labs(color="RMSD")+
   scale_color_brewer(palette="YlGn", labels = c("0-1","2-3","3-5","5+"), direction=-1,
drop=FALSE) +
   new_scale_color() +
   new_scale_fill() +
   geom_label_repel(data = finalLabel, aes(label=Gene, fill=Proteome),
                #color = rep(c("green","red","yellow","blue")[as.factor(finalLabel$Proteome)],times
= 2),
                size = 6,
                seed = 2, box.padding = 1, nudge_x = 0, min.segment.length = 0, segment.size = 1,
segment.color="darkslategrey", max.overlaps = Inf)+ #Inf overlaps leads to issues
   scale_fill_manual(values = cols,
                 labels = c("Mouse", "Human", "Danio", "Fly")) +

   guides(fill = guide_legend(override.aes = aes(color = NA))) +
   guides(size = "none") +

   theme(
         panel.background = element_blank(),
         panel.border = element_blank(),
         panel.grid.major = element_line(size = 0.1, color = "black", linetype = "dashed"),
         panel.grid.minor = element_blank(),
         axis.line = element_line(color="black"),
         axis.text = element_text(size = 22),
         axis.title = element_text(size = 30),
         plot.title = element_text(hjust = 0.5, size = 30),
         legend.text = element_text(size = 22),
         legend.title = element_text(size = 22),
         legend.position = c(0.15, 0.8)
     )

 setwd("/home/ian/homology_search/fcresults")
 #change to SVG to import into illustrator, etc
```

```
  ggsave(filename = paste(protein,".png",sep=""), plot = p, width = 8, height = 8)
}
```

```
#FATCAT and TMalign data visualization v0.2
#IB 12.14.22
#Requires properly-formatted TM-align + FATCAT datafiles
#Requires a "working_prot.txt" list of input files
#change working directory as appropriate

#next: work on limiting "too many hit" plots

library(ggplot2)
library(ggimage)
library(ggrepel)
library(ggnewscale)


protList <-read.table("/home/ian/homology_search/fcresults/working_prot.txt")
protList <- unlist(protList)

cols <- c("Mouse" = "paleturquoise1", "Human" = "mediumspringgreen", "Danio" = "plum1",
"Fly" = "khaki1")

for (protein in protList) {
  setwd("/home/ian/homology_search/fcresults/fat_tm_merge/converted")
  hasHits <- 0
  mouseData <- read.table(header = TRUE, text=gsub(" ", "\t",
readLines(paste(protein,".pdb.mouse.txt",sep=""))))
  humanData <- read.table(header = TRUE, text=gsub(" ", "\t",
readLines(paste(protein,".pdb.human.txt",sep=""))))
  danioData <- read.table(header = TRUE, text=gsub(" ", "\t",
readLines(paste(protein,".pdb.danio.txt",sep=""))))
  flyData   <- read.table(header = TRUE, text=gsub(" ", "\t",
readLines(paste(protein,".pdb.drosophila.txt",sep=""))))

  #append source genome name - used later when annotating graphs
  mouseData$Proteome <- "Mouse"
  humanData$Proteome <- "Human"
  danioData$Proteome <- "Danio"
  flyData$Proteome <- "Fly"

  testData <- merge(humanData, mouseData, all = TRUE)
  testData <- merge(testData, danioData, all = TRUE)
```

```
  testData <- merge(testData, flyData, all = TRUE)

  #Hits defined as TM score > 0.6 AND FATCAT P-value < 0.01
  hits <- testData[testData$TM2 > 0.6, ]
  hits <- hits[-log10(hits$P.value)>2, ]

  #if no hits, instead label the top .01% of results from each tool

    topP <- quantile(-log10(testData$P.value), probs = .9999)
    topTM <- quantile(testData$TM2, probs = .9999)
    toLabel <- testData[testData$TM2>topTM, ]
    toLabel2 <- testData[-log10(testData$P.value)>topP, ]
    toLabel3 <- rbind(toLabel, toLabel2)
    finalLabel <- unique(toLabel3)

  if(hasHits) {
    graphTitle<-protein
  } else {
    graphTitle<-paste(protein, ": No hits, top-scoring values are indicated")
  }


  p <- ggplot(testData, aes(x=TM2, y=(-log10(P.value)))) +
    ggtitle(graphTitle) +
    xlab("TM Score") +
    ylab(bquote(Log[10]~P-value~FATCAT)) +
    geom_point(data = humanData, aes(x=TM2, y=(-log10(P.value)), size=1,
color=factor(cut(RMSD.1, c(0,2,3,5,Inf))))) +
    geom_point(data = mouseData, aes(x=TM2, y=(-log10(P.value)), size=1,
color=factor(cut(RMSD.1, c(0,2,3,5,Inf))))) +
    geom_point(data = danioData, aes(x=TM2, y=(-log10(P.value)), size=1,
color=factor(cut(RMSD.1, c(0,2,3,5,Inf))))) +
    geom_point(data = flyData,   aes(x=TM2, y=(-log10(P.value)), size=1,
color=factor(cut(RMSD.1, c(0,2,3,5,Inf))))) +
    labs(color="RMSD")+
    scale_color_brewer(palette="YlGn", labels = c("0-1","2-3","3-5","5+"), direction=-1,
drop=FALSE) +
    new_scale_color() +
    new_scale_fill() +
    geom_label_repel(data = finalLabel, aes(label=Entry, fill=Proteome),
                  #color = rep(c("green","red","yellow","blue")[as.factor(finalLabel$Proteome)],times
= 2),
                  seed = 2, box.padding = 1, nudge_x = 0, min.segment.length = 0, segment.size = 1,
segment.color="darkslategrey", max.overlaps = Inf)+ #Inf overlaps leads to issues
```

```
  scale_fill_manual(values = cols,
            labels = c("Mouse", "Human", "Danio", "Fly")) +

  guides(fill = guide_legend(override.aes = aes(color = NA))) +
  guides(size = "none") +

  theme(
      panel.background = element_blank(),
      panel.border = element_blank(),
      panel.grid.major = element_line(size = 0.1, color = "black", linetype = "dashed"),
      panel.grid.minor = element_blank(),
      axis.line = element_line(color="black"),

      plot.title = element_text(hjust = 0.5),
      legend.position = c(0.06, 0.75)
    )

 setwd("/home/ian/homology_search/fcresults")
 ggsave(filename = paste(protein,"_toponly_uniprot.png",sep=""), plot = p, width = 8, height =
8)
}
```

```
#FATCAT and TMalign data visualization v0.2
#IB 10.11.2022
#Requires properly-formatted TM-align + FATCAT datafiles
#Requires a "working_prot.txt" list of input files
#change working directory as appropriate

library(ggplot2)
library(ggimage)
library(ggrepel)
library(ggnewscale)


protList <-read.table("/home/ian/homology_search/fcresults/working_prot.txt")
protList <- unlist(protList)

cols <- c("Mouse" = "paleturquoise1", "Human" = "mediumspringgreen", "Danio" = "plum1",
"Fly" = "khaki1")

for (protein in protList) {
```

```
}

  setwd("/home/ian/homology_search/fcresults/fat_tm_merge/converted")
  hasHits <- 0
  mouseData <- read.table(header = TRUE, text=gsub(" ", "\t",
readLines(paste(protein,".pdb.mouse.txt",sep=""))))
  humanData <- read.table(header = TRUE, text=gsub(" ", "\t",
readLines(paste(protein,".pdb.human.txt",sep=""))))
  danioData <- read.table(header = TRUE, text=gsub(" ", "\t",
readLines(paste(protein,".pdb.danio.txt",sep=""))))
  flyData   <- read.table(header = TRUE, text=gsub(" ", "\t",
readLines(paste(protein,".pdb.drosophila.txt",sep=""))))

  #append source genome name - used later when annotating graphs
  mouseData$Proteome <- "Mouse"
  humanData$Proteome <- "Human"
  danioData$Proteome <- "Danio"
  flyData$Proteome <- "Fly"

  testData <- merge(humanData, mouseData, all = TRUE)
  testData <- merge(testData, danioData, all = TRUE)
  testData <- merge(testData, flyData, all = TRUE)

  #Hits defined as TM score > 0.6 AND FATCAT P-value < 0.01
  hits <- testData[testData$TM2 > 0.6, ]
  hits <- hits[-log10(hits$P.value)>2, ]

  #if no hits, instead label the top .01% of results from each tool
  if (nrow(hits)>0){
    finalLabel <- hits
    hasHits <- 1
  } else {
    topP <- quantile(-log10(testData$P.value), probs = .9999)
    topTM <- quantile(testData$TM2, probs = .9999)
    toLabel <- testData[testData$TM2>topTM, ]
    toLabel2 <- testData[-log10(testData$P.value)>topP, ]
    toLabel3 <- rbind(toLabel, toLabel2)
    finalLabel <- unique(toLabel3)
  }

  if(hasHits) {
    graphTitle<-protein
  } else {
    graphTitle<-paste(protein, ": No hits, top-scoring values are indicated")
  }
```

```
  p <- ggplot(testData, aes(x=TM2, y=(-log10(P.value)))) +
    ggtitle(graphTitle) +
    xlab("TM Score") +
    ylab(bquote(Log[10]~P-value~FATCAT)) +
    geom_point(data = humanData, aes(x=TM2, y=(-log10(P.value)), size=1,
color=factor(cut(RMSD.1, c(0,2,3,5,Inf))))) +
    geom_point(data = mouseData, aes(x=TM2, y=(-log10(P.value)), size=1,
color=factor(cut(RMSD.1, c(0,2,3,5,Inf))))) +
    geom_point(data = danioData, aes(x=TM2, y=(-log10(P.value)), size=1,
color=factor(cut(RMSD.1, c(0,2,3,5,Inf))))) +
    geom_point(data = flyData,   aes(x=TM2, y=(-log10(P.value)), size=1,
color=factor(cut(RMSD.1, c(0,2,3,5,Inf))))) +
    labs(color="RMSD")+
    scale_color_brewer(palette="YlGn", labels = c("0-1","2-3","3-5","5+"), direction=-1,
drop=FALSE) +
    new_scale_color() +
    new_scale_fill() +
    geom_label_repel(data = finalLabel, aes(label=Gene, fill=Proteome),
                  #color = rep(c("green","red","yellow","blue")[as.factor(finalLabel$Proteome)],times
= 2),
                  seed = 2, box.padding = 1, nudge_x = 0, min.segment.length = 0, segment.size = 1,
segment.color="darkslategrey", max.overlaps = Inf)+ #Inf overlaps leads to issues
    scale_fill_manual(values = cols,
                   labels = c("Mouse", "Human", "Danio", "Fly")) +

    guides(fill = guide_legend(override.aes = aes(color = NA))) +
    guides(size = "none") +

    theme(
          panel.background = element_blank(),
          panel.border = element_blank(),
          panel.grid.major = element_line(size = 0.1, color = "black", linetype = "dashed"),
          panel.grid.minor = element_blank(),
          axis.line = element_line(color="black"),

          plot.title = element_text(hjust = 0.5),
          legend.position = c(0.06, 0.75)
      )

  setwd("/home/ian/homology_search/fcresults")
  ggsave(filename = paste(protein,".png",sep=""), plot = p, width = 8, height = 8)
}
```

```
#!/bin/bash
#Finalized by Ian Boys ~10.2022
#Merges FATCAT and TM-align search results for visualization and analysis in R
#Make sure to QC FATCAT files first to ensure that runs completed
#Requires FATCAT and TM-align search results in separate "FATCAT" and "TM" folders in root
directory
#Requires UNIPROT gene name/accession ID conversion lists under "IDs" in root directory
#Naming conventions are those used in FATCAT CHPC scripts and local TM-align scripts. Modify
below if required

#set up directories
#rootdir=$(pwd)
rootdir="$( cd -- "$(dirname "$0")" >/dev/null 2>&1 ; pwd -P )"
mkdir TM/TMconvert
mkdir res
mkdir final
mkdir final/converted
echo $rootdir

#remove "pound" and CPU time report from TMalign results
cd "$rootdir/TM"
for file in *.txt; do
	#sed -i '$ d' $file #remove the last line
	sed '1s/^.//' $file > TMconvert/$file #get rid of the first character (#)
done
cd $rootdir

#rename FATCAT files to use same naming as TMalign
for fatfile in FATCAT/*.FATCAT.txt.scores.txt; do
	mv -- "$fatfile" "${fatfile%.FATCAT.txt.scores.txt}.txt"
done
cd $rootdir

#join FATCAT and TMalign files
for file in TM/TMconvert/*; do
	name=${file##*/}

	if [ -f "FATCAT/$name" ]; then
		paste "$file" "FATCAT/$name" > "res/$name"
	fi
done
cd $rootdir
```

```
#append gene names to existing tables
for file in res/*; do
	name=${file##*/}

	case $name in
		*"human"*)
			paste "IDs/humanIDs.tsv" "res/$name" > "final/$name";;
		*"drosophila"*)
			paste "IDs/flyIDs.tsv" "res/$name" > "final/$name";;
		*"danio"*)
			paste "IDs/danioIDs.tsv" "res/$name" > "final/$name";;
		*"mouse"*)
			paste "IDs/mouseIDs.tsv" "res/$name" > "final/$name";;

	esac
done

#removes "short match" (ie failed) TM-align results, as these interfere w/ downstream analysis
cd $rootdir/final
for file in *.txt; do
	grep -v "Short match" $file > converted/$file
done
```

```
#!/bin/bash
#check for expected last ID within fatcat file, echo problem files to log.txt

for file in *.txt; do
	name=${file##*/}

	case $name in
		*"human"*)
			if ! grep -q "X6R8D5" $file; then echo $file >> log.txt ; fi ;;
		*"drosophila"*)
			if ! grep -q "X4YX01" $file; then echo $file >> log.txt ; fi ;;
		*"danio"*)
			if ! grep -q "Z4YHZ5" $file; then echo $file >> log.txt ; fi ;;
		*"mouse"*)
			if ! grep -q "W8DXL4" $file; then echo $file >> log.txt ; fi ;;
	esac
done
```

```
#!/bin/bash

outfile="secondary_structures.txt"
numss=0

for FILE in *.pdb
do
	stride $FILE > $FILE.stride
done

for FILE in *.stride
do
	echo -n $FILE >> $outfile
	while read line
	do
		if [[ ${line:0:3} == 'LOC' ]]
		then
			((numss++))
		fi
	done < $FILE
	echo -n " " >> $outfile
	echo $numss >> $outfile
	numss=0
done
```
