# Supplementary material for: Structural homology screens reveal poxvirus-encoded proteins impacting inflammasome-mediated defenses
Source: bioRxiv. 2023 Apr 3:2023.02.26.529821. Originally published 2023 Feb 27. Preprint. [Version 2] doi: 10.1101/2023.02.26.529821 (PMC10002665; doi:10.1101/2023.02.26.529821)
Supplement: Supplement 2 [file NIHPP2023.02.26.529821v2-supplement-2.pdf]

bioRxiv preprint doi: https://doi.org/10.1101/2023.02.26.529821; this version posted April 3, 2023. The copyright holder for this preprint (which was not certified by peer review) is the author/funder, who has granted bioRxiv a license to display the preprint in perpetuity. It is made available under aCC-BY 4.0 International license.

Supplemental Figures

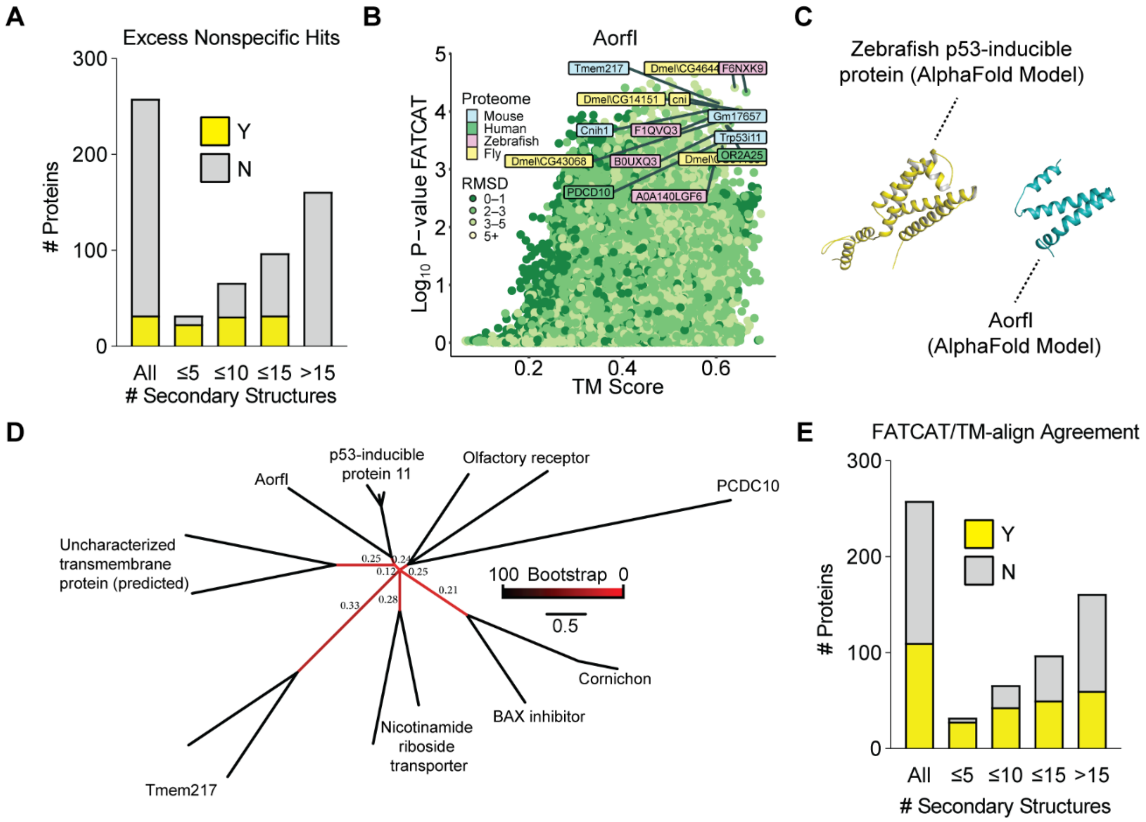

Figure S1. Related to Figure 1

a. Tabulation of nonspecific hits by number of protein secondary structures as determined by STRIDE.
b. FATCAT and TM-align results for A orf I. Only a subset of the 100+ “hits” are indicated.
c. Comparison of AlphaFold models of A orf I and danio p53-inducible protein 11.
d. Maximum likelihood tree of the hits indicated in Figure S1A. Select bootstrap values from 100 replicates are indicated.
e. Tabulation of proteins for which TM-align and FATCAT converged by number of protein secondary structures determined by STRIDE.

bioRxiv preprint doi: https://doi.org/10.1101/2023.02.26.529821; this version posted April 3, 2023. The copyright holder for this preprint (which was not certified by peer review) is the author/funder, who has granted bioRxiv a license to display the preprint in perpetuity. It is made available under aCC-BY 4.0 International license.

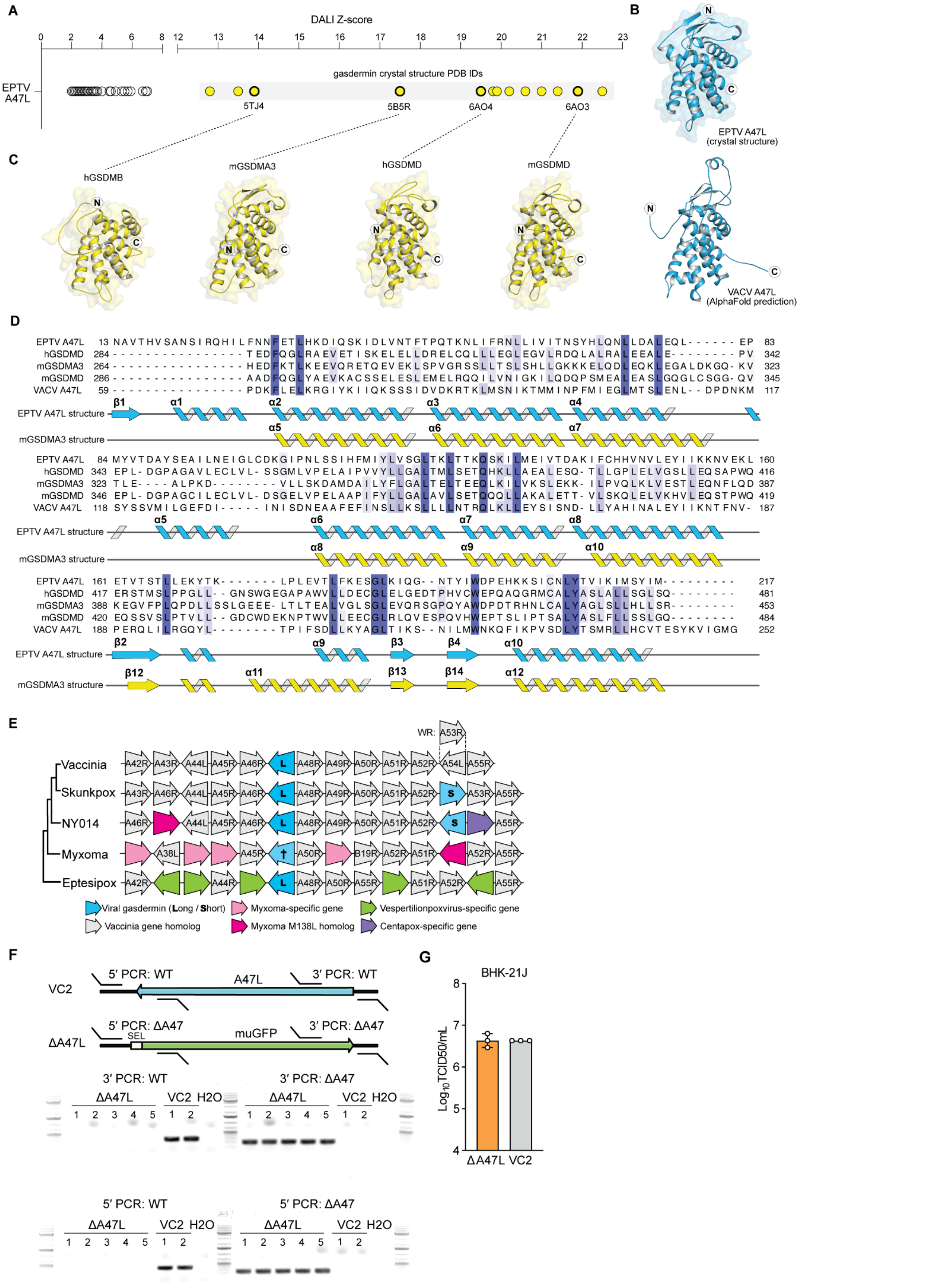

bioRxiv preprint doi: https://doi.org/10.1101/2023.02.26.529821; this version posted April 3, 2023. The copyright holder for this preprint (which was not certified by peer review) is the author/funder, who has granted bioRxiv a license to display the preprint in perpetuity. It is made available under aCC-BY 4.0 International license.

Figure S2. Related to Figure 2

a. DALI Z-scores from searching EPTV gasdermin structure against the full Protein Data Bank (PDB). Results for multiple chains were manually removed. PDB entries that represent gasdermin crystal structures in the PDB were highlighted yellow, and select PDB entries and the structures of four select structures are show in panel c.
b. Experimental and predicted structures of poxvirus gasdermin proteins reveal gasdermin homology. Top, crystal structure of the Eptesipox virus (EPTV) gasdermin. Bottom, AlphaFold predicted structure of vaccinia virus (VACV) gasdermin. The N- and C-termini are indicated with the circled letters N and C.
c. Representative published crystal structures of human (h) or mouse (m) gasdermins from the top EPTV A47L DALI hits shown in panel a. Shown structures are of the hGSDMB CTD (PDB ID 5TJ4), mGSDMA3 (PDB ID 5B5R, residues 264-453), hGSDMD CTD (PDB ID 6AO4), and mGSDMD (PDB ID 6AO3). The N- and C-termini are indicated with the circled letters N and C.
d. Structure-based sequence alignment of the EPTV gasdermin crystal structure and structures from panels b and c. The hGSDMB structure was excluded due to large gaps in alignment, as were the first 58 residues of the VACV gasdermin AlphaFold predicted structure. The secondary structure of mGSDMA3 is based on the numbering scheme used previously [28].
e. Synteny analysis of viral gasdermins. When applicable, genes are labeled based on their vaccinia homologs. †: The evolutionary history of leporipox gasdermin is ambiguous (see main text).
f. Top: schematic of A47 locus for VC2 and ΔA47L recombinant virus with PCR genotyping primers indicated. Bottom: PCR genotyping of recombinant ΔA47L virus. VC2 (wild-type) is included as a control. ΔA47L clonal stock 1 was used for experiments.
g. BHK-21J cells were infected with wild-type (VC2) or A47L-deficient vaccinia virus at an MOI of 1 and viral titer was quantified by TCID50 24 hours post-infection. n = 3 biological replicates.

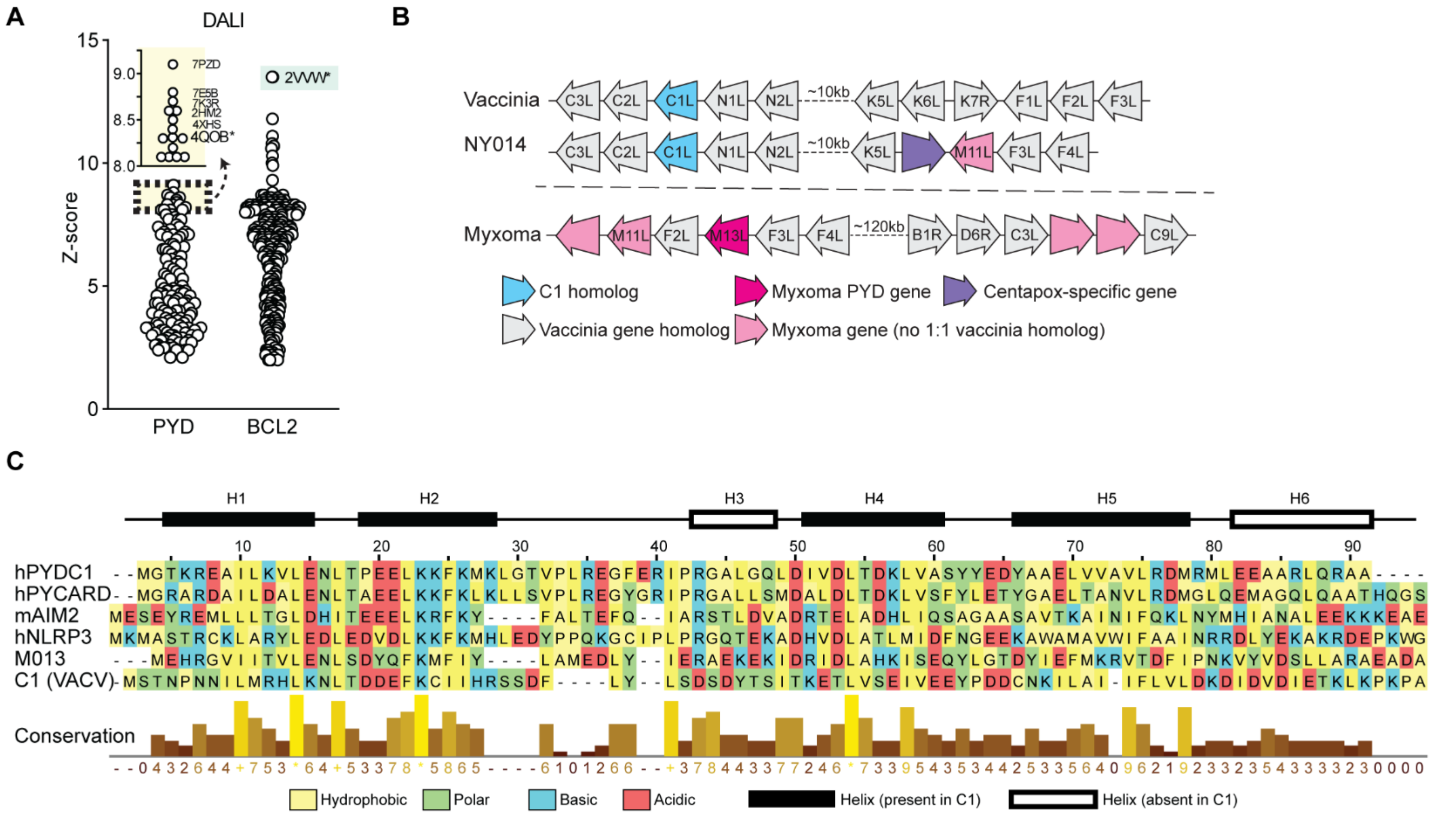

Figure S3. Related to Figure 3

a. DALI Z-scores from searching C1 subdomain AlphaFold models against the full Protein Data Bank (PDB).
b. Synteny analysis of C1 and M013 families. When applicable, genes are labeled based on their vaccinia homologs.
c. Amino acid alignment of select pyrin domains. Helices denoted are those present in ASC [71].

bioRxiv preprint doi: https://doi.org/10.1101/2023.02.26.529821; this version posted April 3, 2023. The copyright holder for this preprint (which was not certified by peer review) is the author/funder, who has granted bioRxiv a license to display the preprint in perpetuity. It is made available under aCC-BY 4.0 International license.

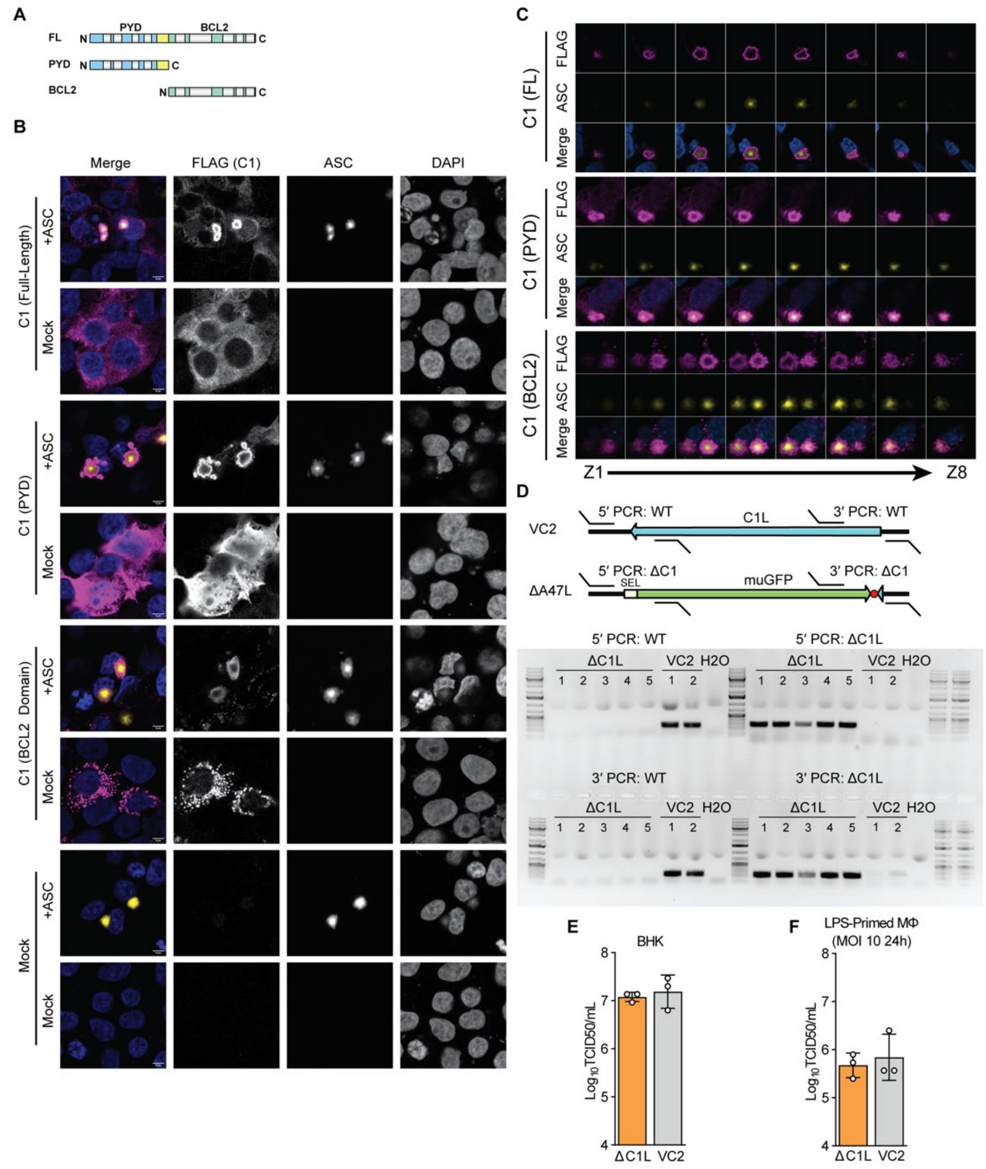

Figure S4. Related to Figure 4

a. C1 truncations used in this study. Helices (grey boxes) and the PYD-Bcl-2 linker (yellow) are indicated.
b. 293T cells were co-transfected with the indicated plasmids and either ASC-GFP [1] or an empty vector. 24 hours post-transfection, cells were fixed and stained for FLAG (C1) and subsequently imaged. Representative image from n=3 independent experiments. Images for full-length C1 protein are the same as in Figure 4B. Scalebar: 5µm.
c. Z stacks of ASC specks in 293T cells co-transfected with ASC-GFP and the indicated FLAG-tagged C1-expressing constructs. Z stacks are from the same experiment shown in Figure 4B.
d. Top: schematic of C1L locus for VC2 and ΔC1L recombinant virus with PCR genotyping primers indicated. Red octagon represents the stop codon introduced in-frame to preserve the coding sequence of N1L (see methods). Bottom: PCR genotyping of recombinant ΔC1L virus. VC2 (wild-type) is included as a control. ΔC1L clonal stock 2 was used for experiments.
e. BHK-21J cells were infected with wild-type (VC2) or C1L-deficient vaccinia virus at an MOI of 1 and viral titer was quantified by TCID50 24 hours post-infection. n = 3 biological replicates.
f. LPS-primed murine macrophages were infected with wild-type (VC2) or C1L-deficient vaccinia virus at an MOI of 10 and viral titer was quantified by TCID50 24 hours post-infection. n = 3 biological replicates. Samples are from the same experiment as Figure 4G.

bioRxiv preprint doi: https://doi.org/10.1101/2023.02.26.529821; this version posted April 3, 2023. The copyright holder for this preprint (which was not certified by peer review) is the author/funder, who has granted bioRxiv a license to display the preprint in perpetuity. It is made available under aCC-BY 4.0 International license.

List of Supplemental Files

Table S1: Vaccinia homology screen results
Table S2: Accession numbers used in this study
Table S3: Oligos and recombinant DNA used or generated in this study
Table S4: Crystallographic statistics
File S1: All vaccinia virus AlphaFold models
File S2: All vaccinia screen results (images)
File S3: Alignments and trees used for phylogenetic analyses
File S4: Scripts used in this study
